# Supplementary material for: Energy stress promotes P-bodies formation via lysine-63-linked polyubiquitination of HAX1
Source: EMBO J. 2024 May 20;43(13):11. doi: 10.1038/s44318-024-00120-6 (PMC11217408; doi:10.1038/s44318-024-00120-6)
Supplement: Supplementary file 1 — Appendix [file 44318_2024_120_MOESM1_ESM.pdf]

APPENDIX

Zhan *et al.*

Table of contents

Appendix Figures

Appendix Figure S1, related to Figure 6……………………………………….2

Appendix Figure S2, related to Figure 8……………………………………….4

Appendix Figure S3, related to Figure 8……………………………………….6

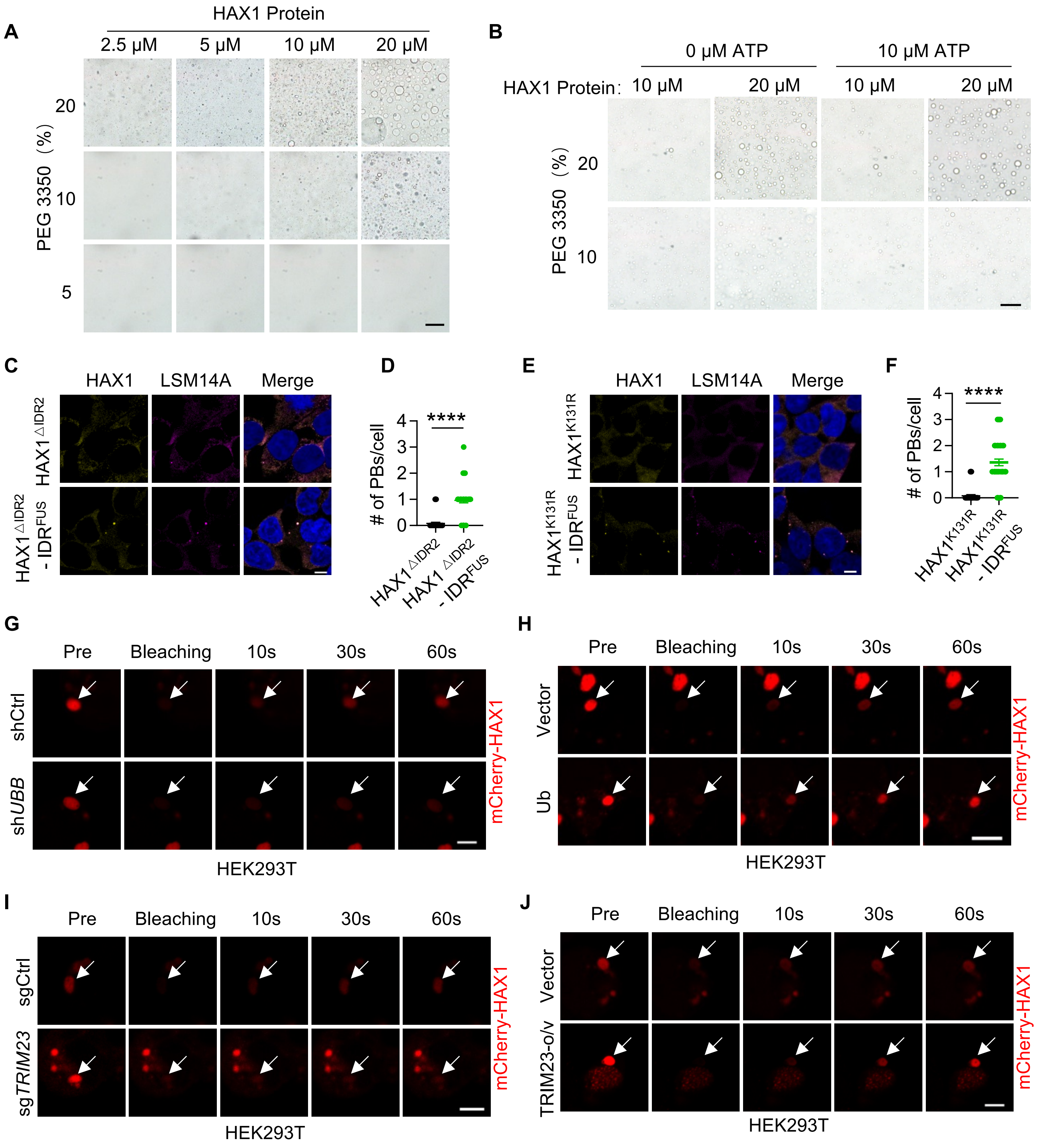

Appendix Figure S1 - Figure legend on the next page

Appendix Figure S1. Phase separation of HAX1.

(**A**) LLPS of recombinant HAX1 proteins in the presence of 150 mM NaCl and 5%-20% (w/v) PEG3350. Scale bar, 25 μm.

(**B**) LLPS of recombinant HAX1 proteins in 150 mM NaCl and 10%-20% (w/v) PEG3350 with or without the addition of 10 μM ATP. Scale bar, 25 μm.

(**C-F**) *HAX1* KO HEK293T cells were transfected with mCherry-HAX1$^{\Delta IDR2}$ or mCherry-HAX1$^{\Delta IDR2}$-IDR^{FUS} (C, D), or mCherry-HAX1^{K131R} or mCherry-HAX1^{K131R}-IDR^{FUS} (E, F) and stained for LSM14A. Representative images are shown in (C, E). The number of P-bodies within each cell is plotted in (D, F) (n = 50). Scale bar, 5 μm. Error bars indicate SEM. ****P < 0.0001 (Student's t test).

(**G-J**) FRAP analysis of HAX1-mCherry in *UBB* knockdown (G), Ub overexpression (H), *TRIM23* knockout (I), or TRIM23 overexpression (J) HEK293T cells. Scale bar, 5 μm.

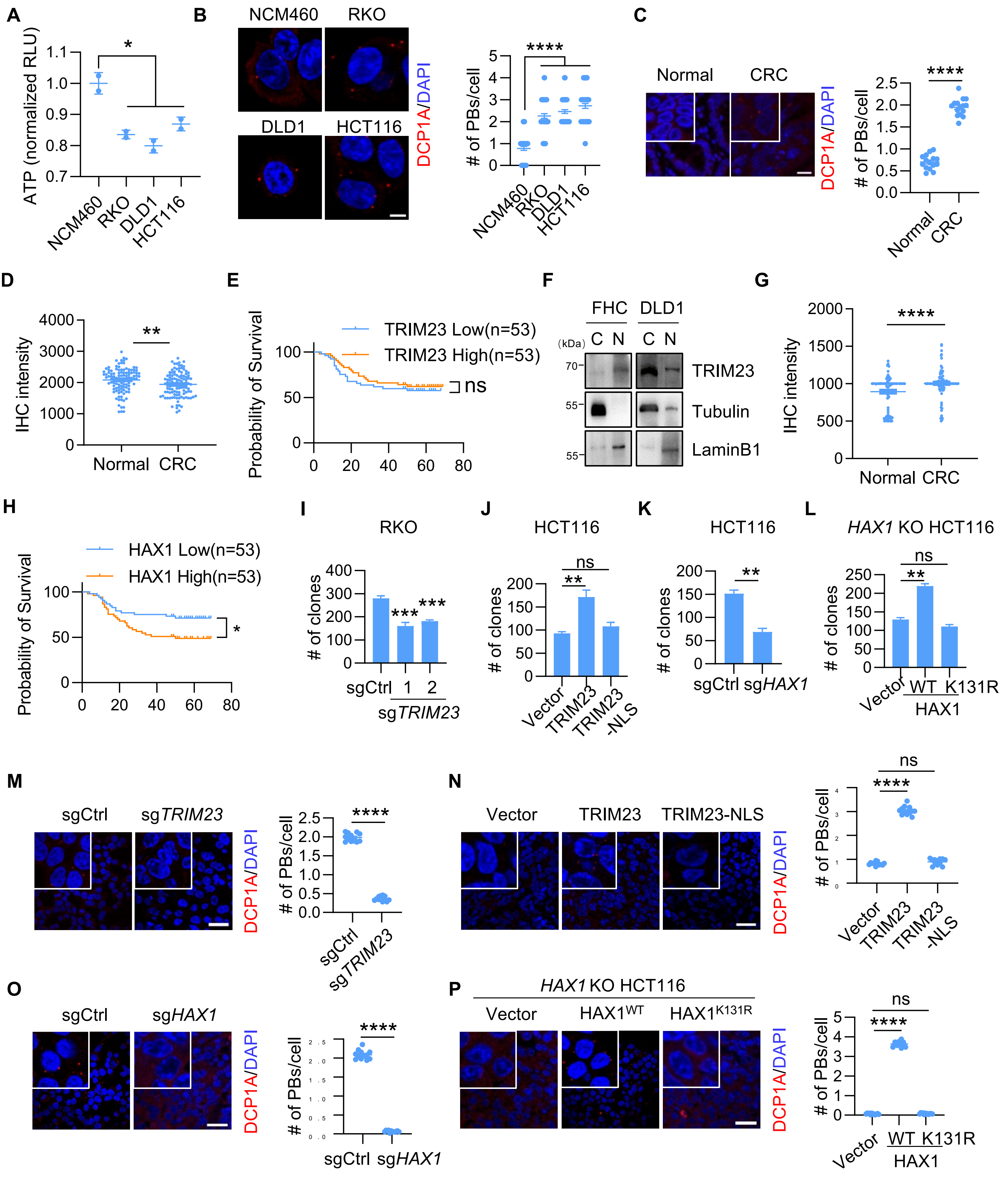

Appendix Figure S2 - Figure legend on the next page

Appendix Figure S2. TRIM23 and HAX1 promote colorectal cancer progression and are associated with poor prognosis in tumor patients.

(**A**) ATP levels in NCM460, RKO, DLD1 and HCT116 cells were detected. Statistical Error bars indicate SD. *P < 0.05 (Student's t test).

(**B**) NCM460, RKO, DLD1 and HCT116 cells were stained for the P-body marker DCP1A (left). The number of P-bodies within each cell is plotted (right) (n = 50). Scale bar, 5 μm. Statistical Error bars indicate SEM. ****P < 0.0001 (Student's t test).

(**C**) Immunofluorescence staining of P-bodies in normal adjacent tissues and CRC tissues. The number of P-bodies within each cell from 15 patients is plotted (right). Scale bars, 20 μm (inset: 10 μm). Statistical Error bars indicate SEM. ****P < 0.0001 (Student's t test).

(**D**) Quantitative immunohistochemical staining of TRIM23 in normal adjacent tissues and CRC tissues. Statistical Error bars indicate SEM. **P < 0.01 (Student's t test).

(**E**) Kaplan-Meier survival analysis of CRC patients with high TRIM23 expression and low TRIM23 expression. ns: no significance (Log-rank test).

(**F**) The expression levels of TRIM23 in the cytoplasm (C) and nucleus (N) were determined via karyoplasmic separation in normal intestinal epithelial cells (FHC) and DLD1 cells.

(**G**) Quantitative immunohistochemical staining of HAX1 in normal adjacent tissues and CRC tissues. Statistical Error bars indicate SEM. **P < 0.01 (Student's t test).

(**H**) Kaplan-Meier survival analysis of CRC patients with high and low HAX1 expression. *P < 0.05 (Log-rank test).

(**I-L**) Colony formation assay in the indicated CRC cells. At least two biological replicates are plotted as the mean ± SD. Ns: no significance, **P < 0.01, ***P < 0.001 (Student's t test).

(**M-P**) Immunofluorescence staining of P-bodies in xenograft tumors from Fig. 8J-M. The number of P-bodies represented by DCP1A within each cell from 12 mice are plotted (right). Scale bars: 20 μm (inset: 10 μm). Error bars indicate SEM. ns: no significance, ****P < 0.0001 (Student's t test).

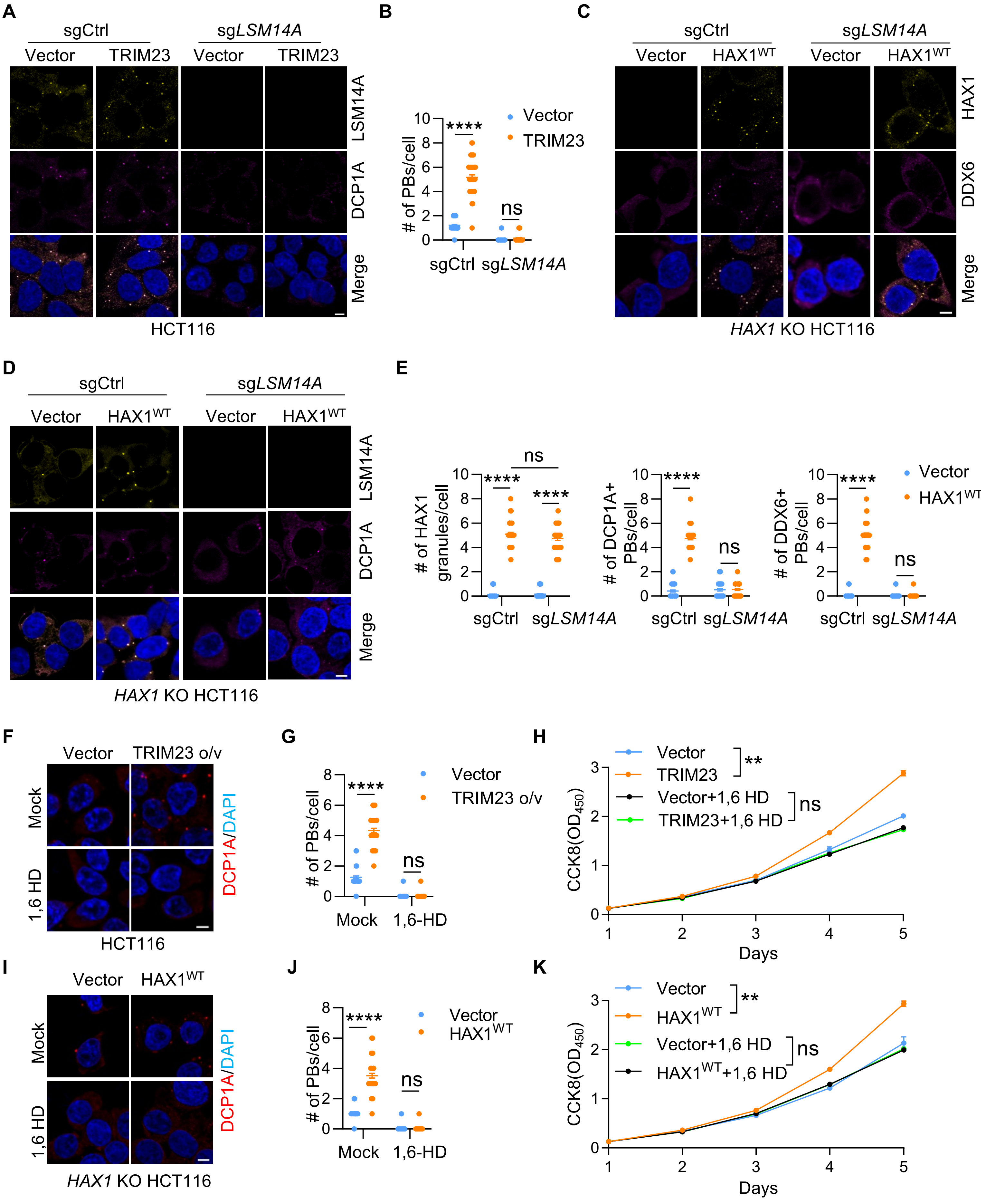

Appendix Figure S3 - Figure legend on the next page

Appendix Figure S3. The effect of TRIM23/HAX1 on the tumorigenicity of CRC cells was dependent on P-bodies.

(**A, B**) Control or TRIM23-overexpressing HCT116 cells were transduced with control or *LSM14A* sgRNA. Cells were stained for LSM14A and DCP1A and imaged (A). The number of P-bodies labeled with DCP1A within each cell is plotted in (B) (n = 50). Scale bar, 5 μm. Error bars indicate SEM. Ns: no significance, ****P < 0.0001 (Two-way ANOVA).

(**C-E**) *HAX1* KO HCT116 cells expressing control or HAX1 were transduced with control or *LSM14A* sgRNA. Cells were stained for DDX6 and HAX1 (C) or for LSM14A and DCP1A (D). The number of HAX1 granules or P-bodies labeled with DCP1A or DDX6 within each cell are plotted in (E) (n = 50). Scale bar, 5 μm. Error bars indicate SEM. Ns: no significance, ****P < 0.0001 (Two-way ANOVA).

(**F-H**) Control or TRIM23-overexpressing HCT116 cells were treated with or without 1,6-HD (3%, 5 min). Cells were stained for DCP1A and imaged (F) (Scale bar, 5 μm), and the number of P-bodies within each cell is plotted in (G) (n = 50, Error bars indicate SEM). The proliferation of the indicated cells was determined by CCK-8 assay (H) (n = 3, Error bars indicate SD). ns: no significance, **P < 0.01, **P < 0.01 (Two-way ANOVA).

(**I-K**) *HAX1* KO HCT116 cells expressing control or HAX1 were treated with or without 1,6-HD (3%, 5 min). Cells were stained for DCP1A and imaged (I) (Scale bar, 5 μm), and the number of P-bodies within each cell is plotted in (J) (n = 50, Error bars indicate SEM). The proliferation of the indicated cells was determined by CCK-8 assay (K) (n = 3, Error bars indicate SD). ns: no significance, **P < 0.01, **P < 0.01 (Two-way ANOVA).
