# Supplementary figures and images for: Energy stress promotes P-bodies formation via lysine-63-linked polyubiquitination of HAX1 (part 1 of 4)
Source: EMBO J. 2024 May 20;43(13):11. doi: 10.1038/s44318-024-00120-6 (PMC11217408; doi:10.1038/s44318-024-00120-6)

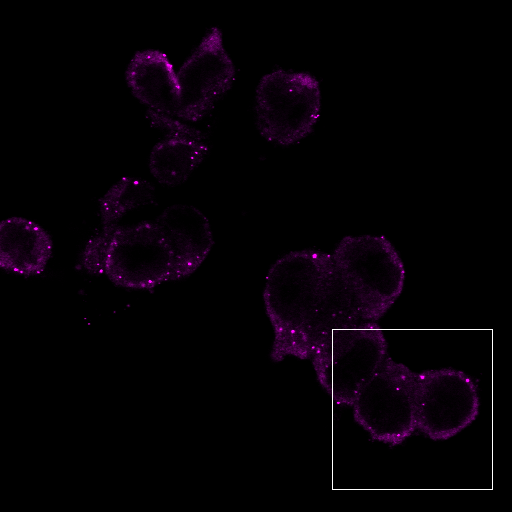

Supplement: Supplementary file 4 — Source data Fig. 1 [file 44318_2024_120_MOESM4_ESM.zip › Figure 1/1A/2-DG/DCP1A.tif]

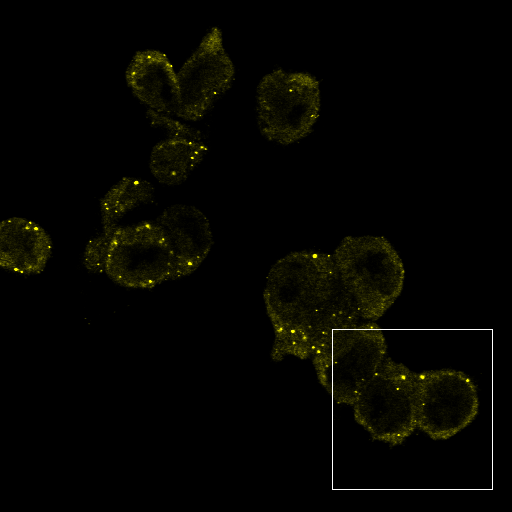

Supplement: Supplementary file 4 — Source data Fig. 1 [file 44318_2024_120_MOESM4_ESM.zip › Figure 1/1A/2-DG/LSM14A.tif]

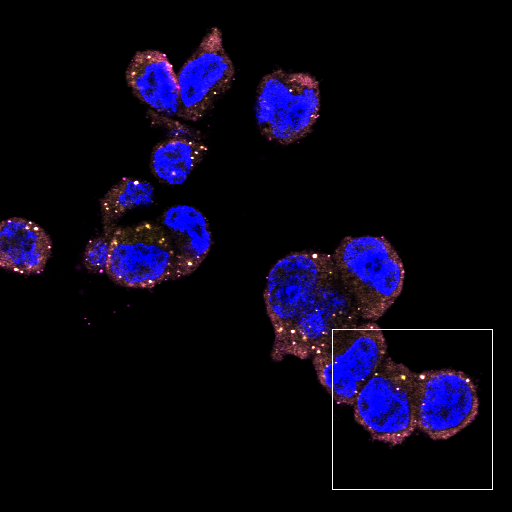

Supplement: Supplementary file 4 — Source data Fig. 1 [file 44318_2024_120_MOESM4_ESM.zip › Figure 1/1A/2-DG/Merge.tif]

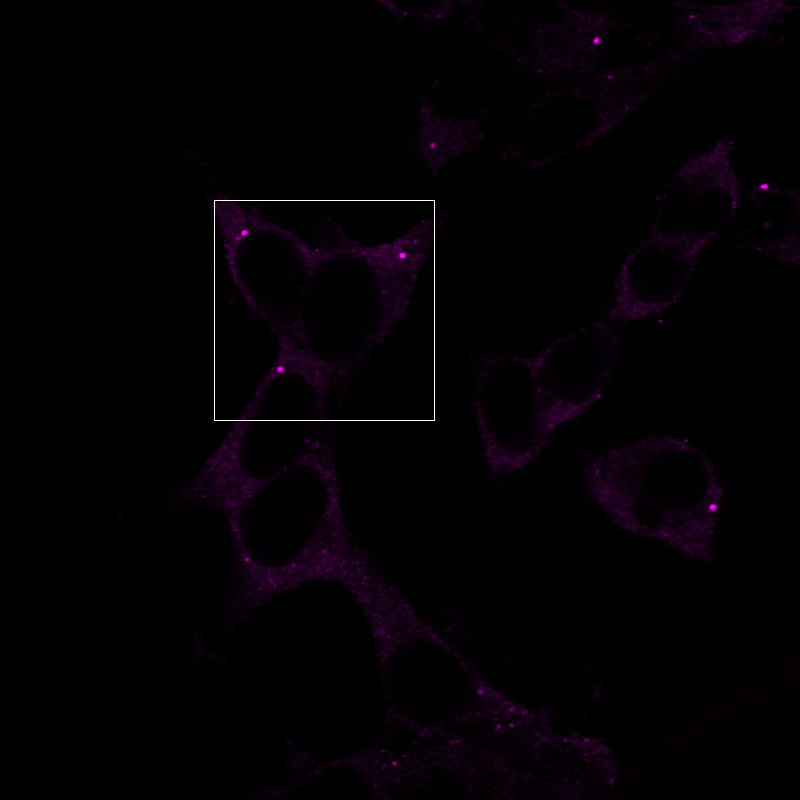

Supplement: Supplementary file 4 — Source data Fig. 1 [file 44318_2024_120_MOESM4_ESM.zip › Figure 1/1A/Mock/DCP1A.tif]

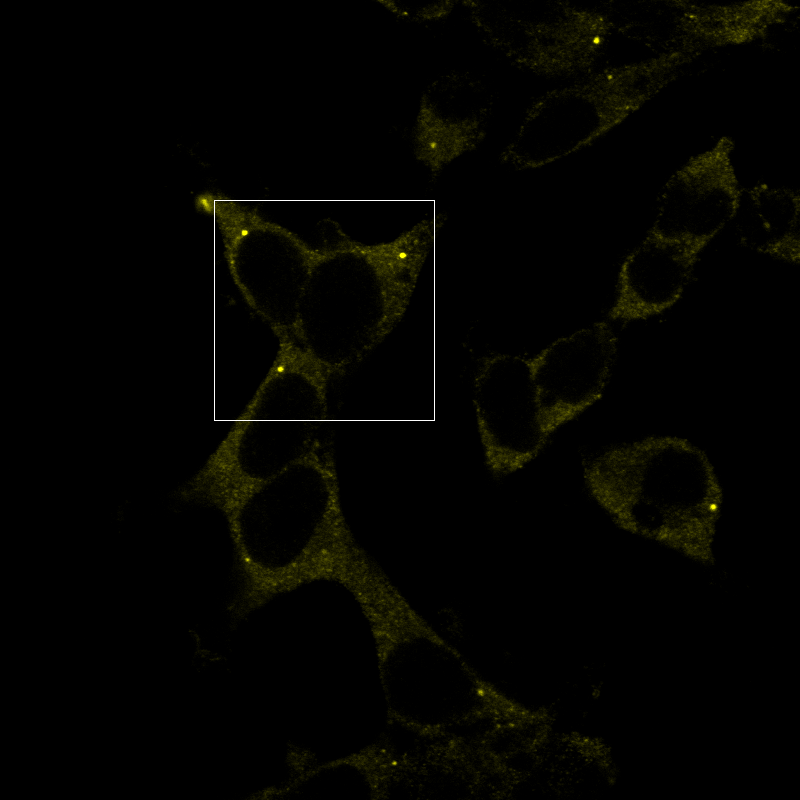

Supplement: Supplementary file 4 — Source data Fig. 1 [file 44318_2024_120_MOESM4_ESM.zip › Figure 1/1A/Mock/LSM14A.tif]

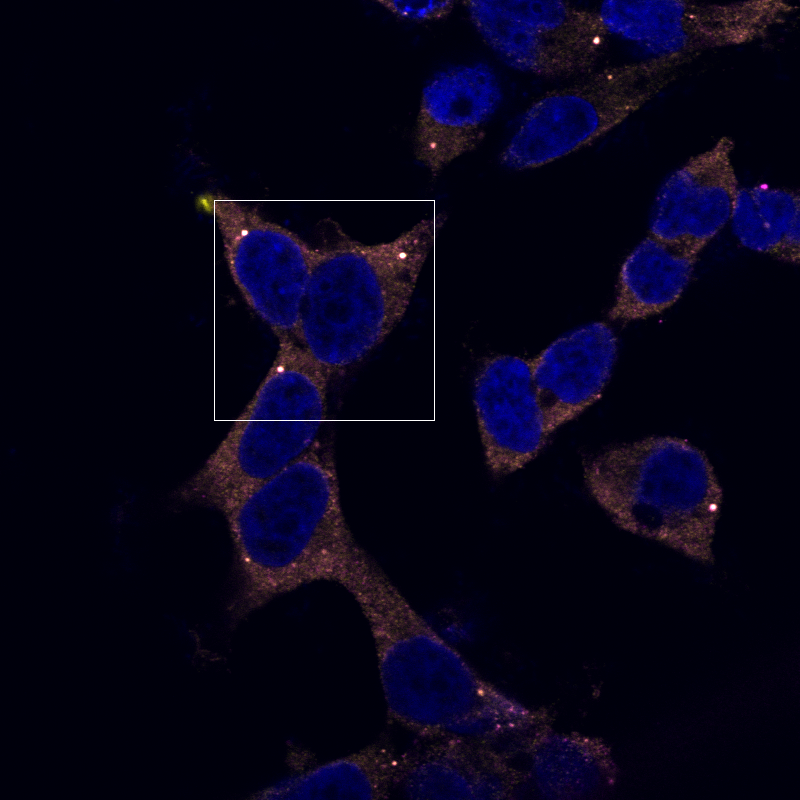

Supplement: Supplementary file 4 — Source data Fig. 1 [file 44318_2024_120_MOESM4_ESM.zip › Figure 1/1A/Mock/Merge.tif]

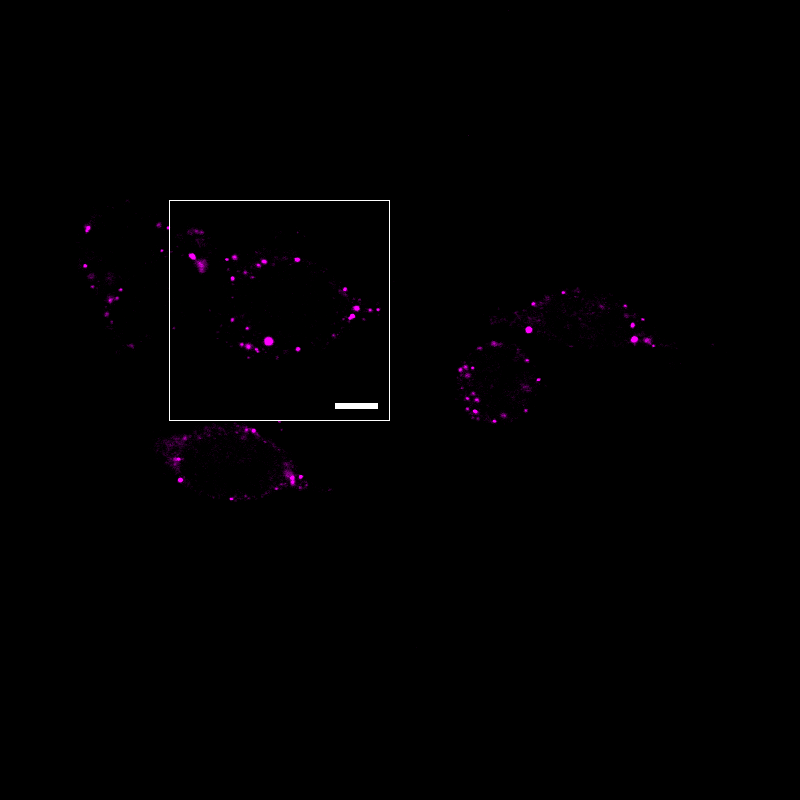

Supplement: Supplementary file 4 — Source data Fig. 1 [file 44318_2024_120_MOESM4_ESM.zip › Figure 1/1A/2-DG+Oligomycin/DCP1A.tif]

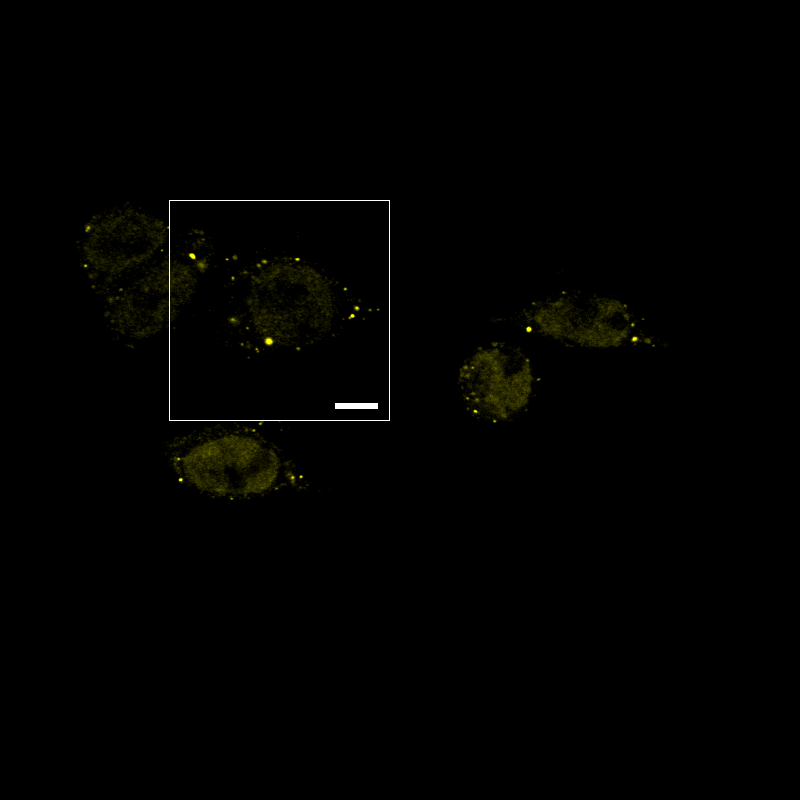

Supplement: Supplementary file 4 — Source data Fig. 1 [file 44318_2024_120_MOESM4_ESM.zip › Figure 1/1A/2-DG+Oligomycin/LSM14A.tif]

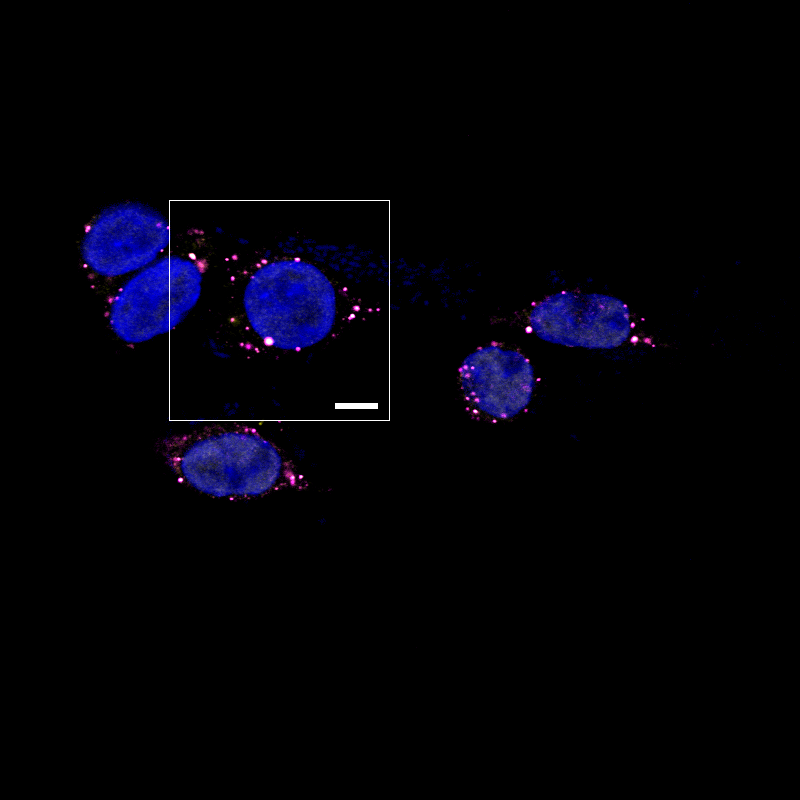

Supplement: Supplementary file 4 — Source data Fig. 1 [file 44318_2024_120_MOESM4_ESM.zip › Figure 1/1A/2-DG+Oligomycin/Merge.tif]

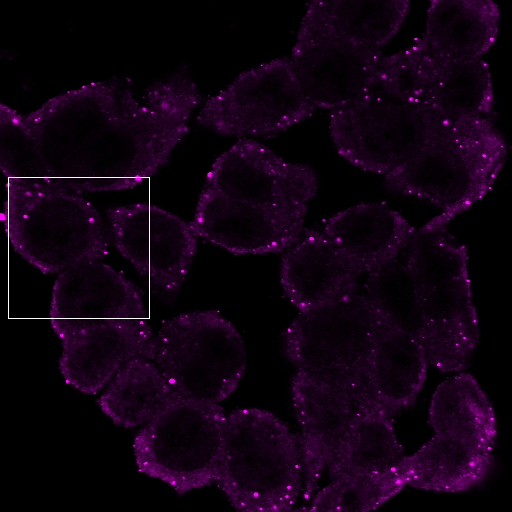

Supplement: Supplementary file 4 — Source data Fig. 1 [file 44318_2024_120_MOESM4_ESM.zip › Figure 1/1A/Oligomycin/DCP1A.tif]

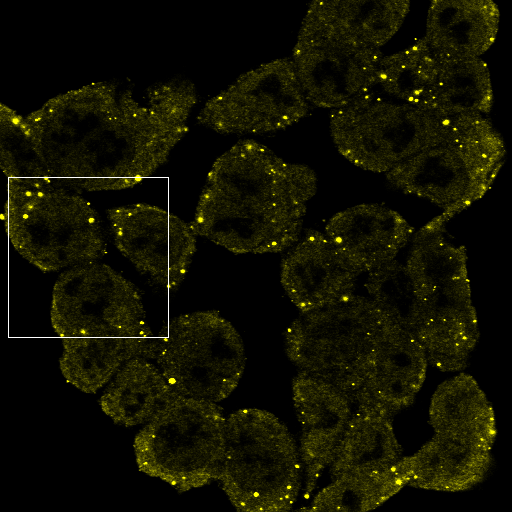

Supplement: Supplementary file 4 — Source data Fig. 1 [file 44318_2024_120_MOESM4_ESM.zip › Figure 1/1A/Oligomycin/LSM14A.tif]

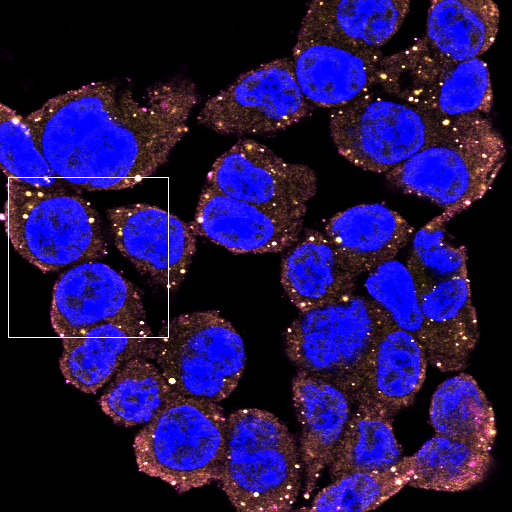

Supplement: Supplementary file 4 — Source data Fig. 1 [file 44318_2024_120_MOESM4_ESM.zip › Figure 1/1A/Oligomycin/Merge.tif]

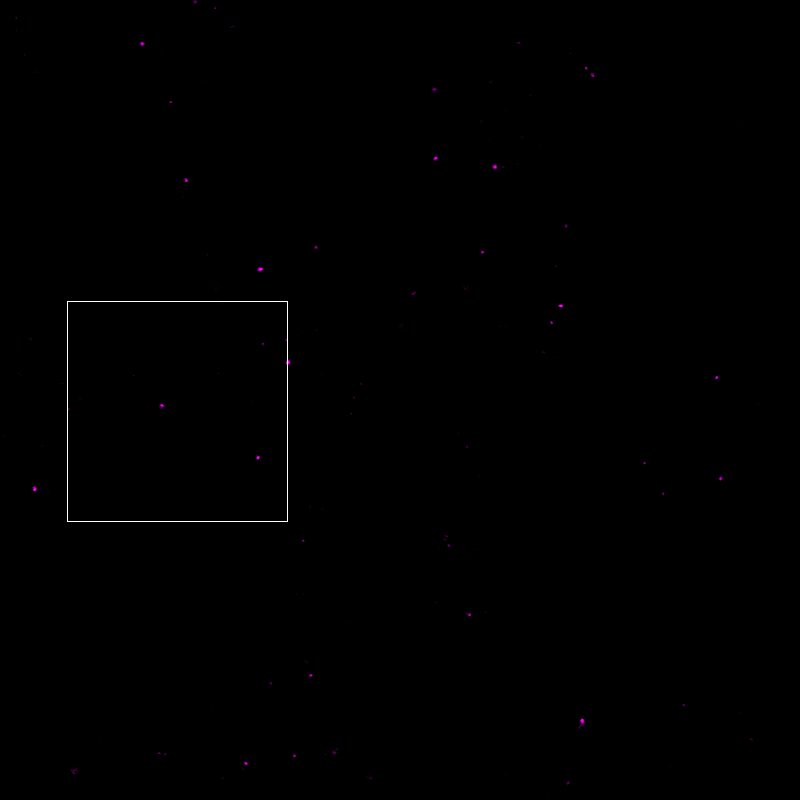

Supplement: Supplementary file 4 — Source data Fig. 1 [file 44318_2024_120_MOESM4_ESM.zip › Figure 1/1G/Oligomycin+ATPsome/DCP1A.tif]

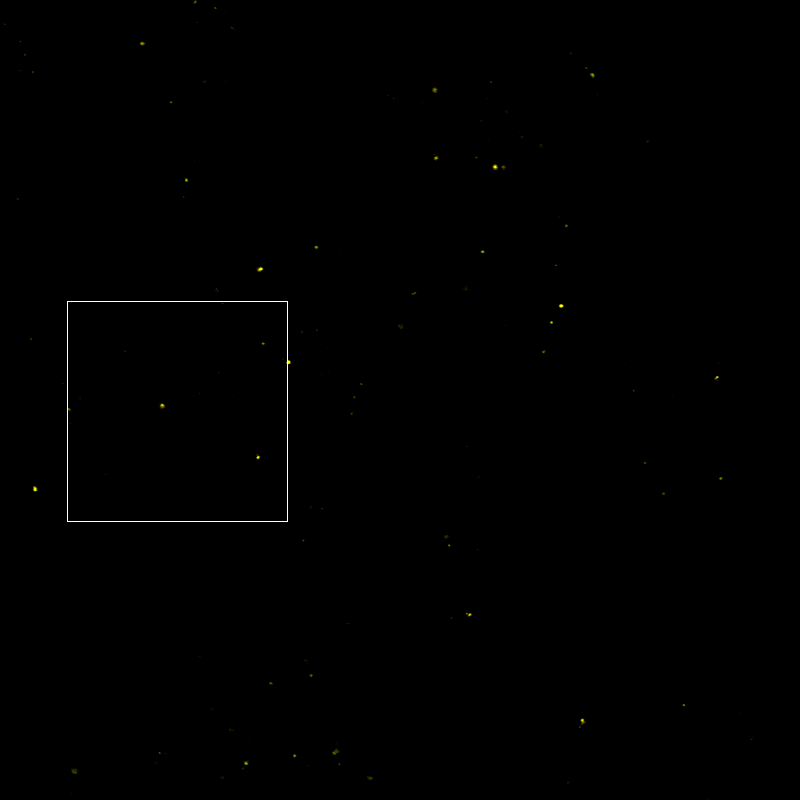

Supplement: Supplementary file 4 — Source data Fig. 1 [file 44318_2024_120_MOESM4_ESM.zip › Figure 1/1G/Oligomycin+ATPsome/LSM14A.tif]

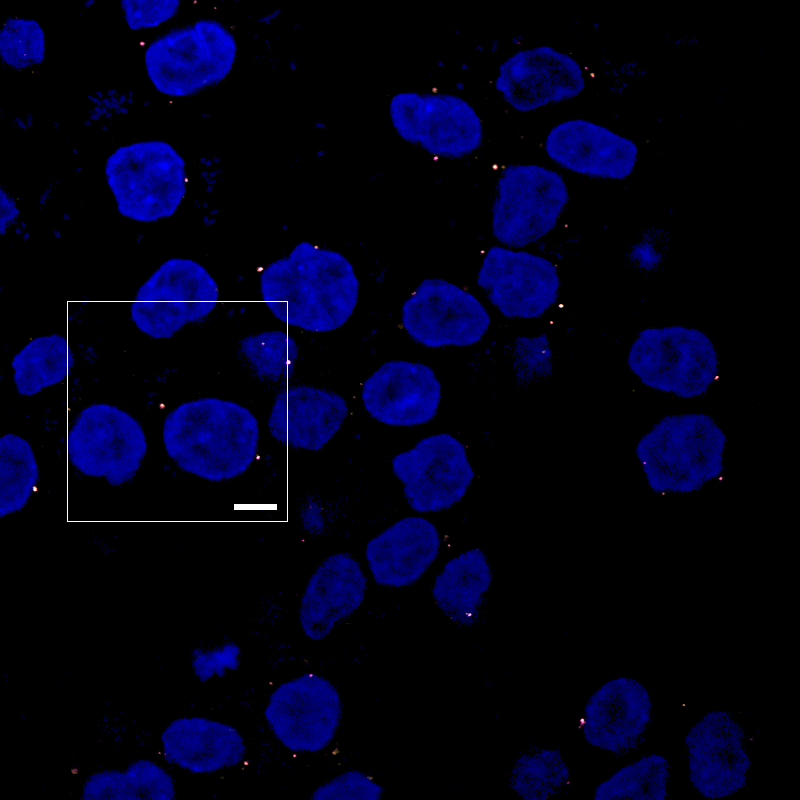

Supplement: Supplementary file 4 — Source data Fig. 1 [file 44318_2024_120_MOESM4_ESM.zip › Figure 1/1G/Oligomycin+ATPsome/Merge.tif]

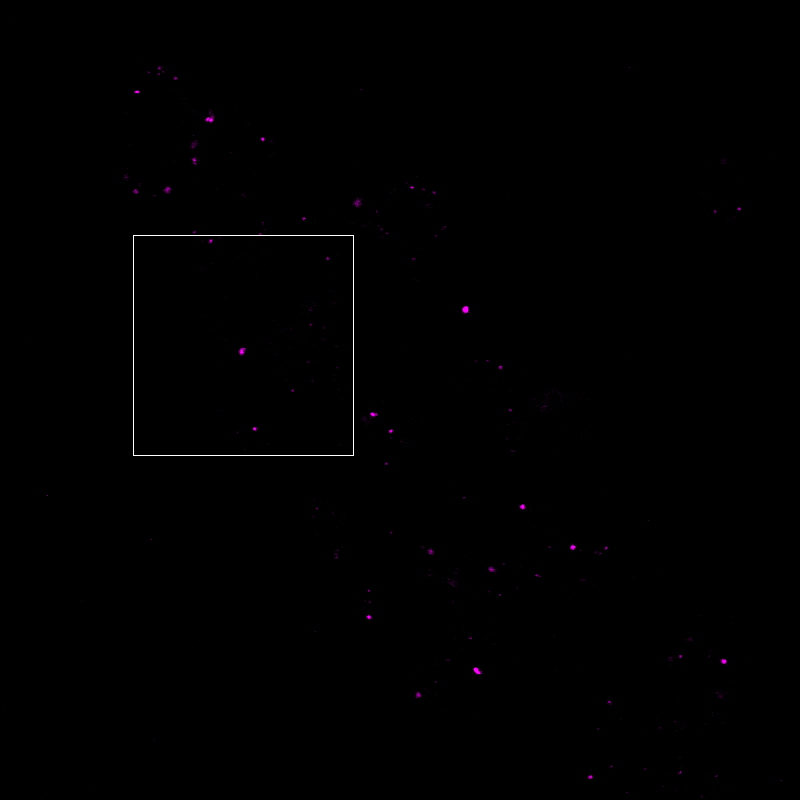

Supplement: Supplementary file 4 — Source data Fig. 1 [file 44318_2024_120_MOESM4_ESM.zip › Figure 1/1G/Liposome/DCP1A.tif]

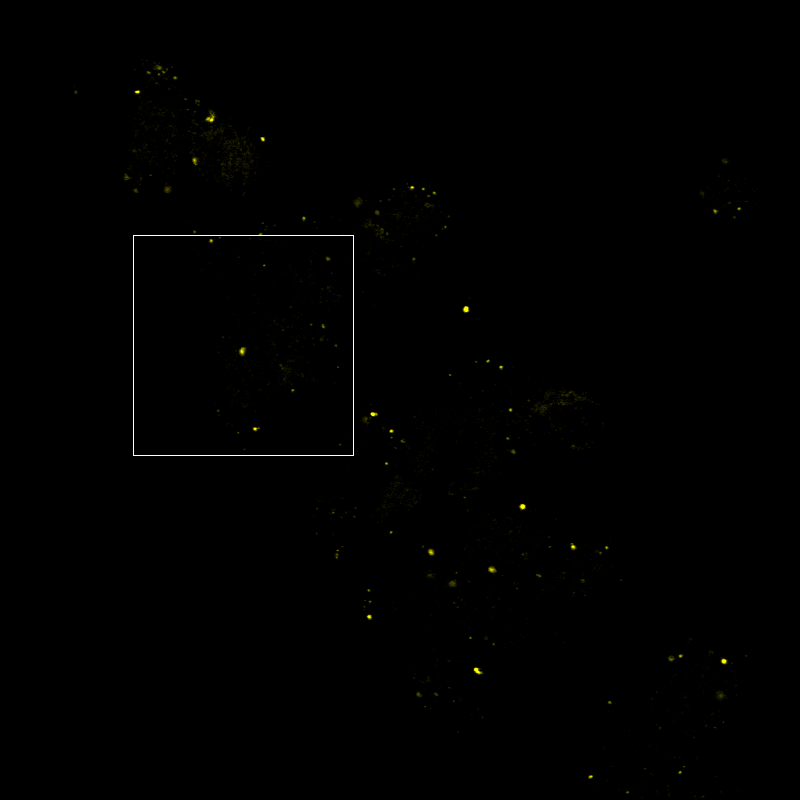

Supplement: Supplementary file 4 — Source data Fig. 1 [file 44318_2024_120_MOESM4_ESM.zip › Figure 1/1G/Liposome/LSM14A.tif]

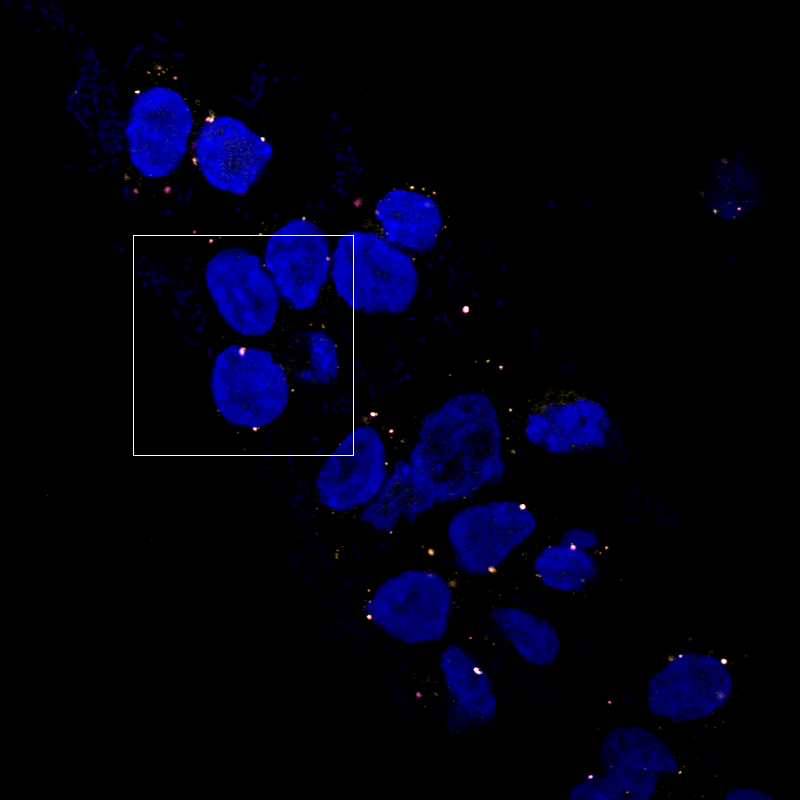

Supplement: Supplementary file 4 — Source data Fig. 1 [file 44318_2024_120_MOESM4_ESM.zip › Figure 1/1G/Liposome/Merge.tif]

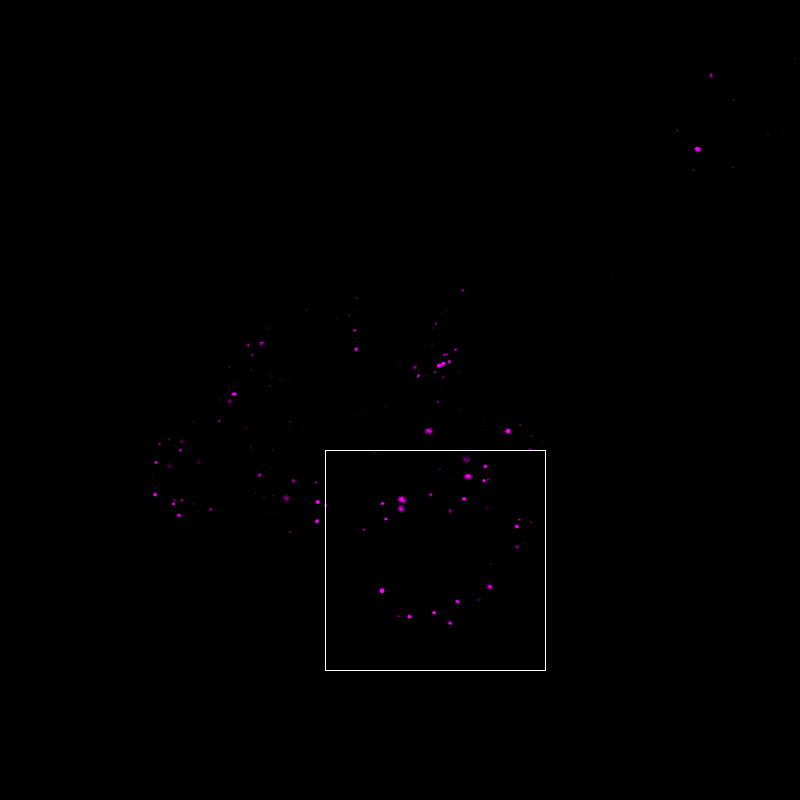

Supplement: Supplementary file 4 — Source data Fig. 1 [file 44318_2024_120_MOESM4_ESM.zip › Figure 1/1G/Oligomycin+Liposome/DCP1A.tif]

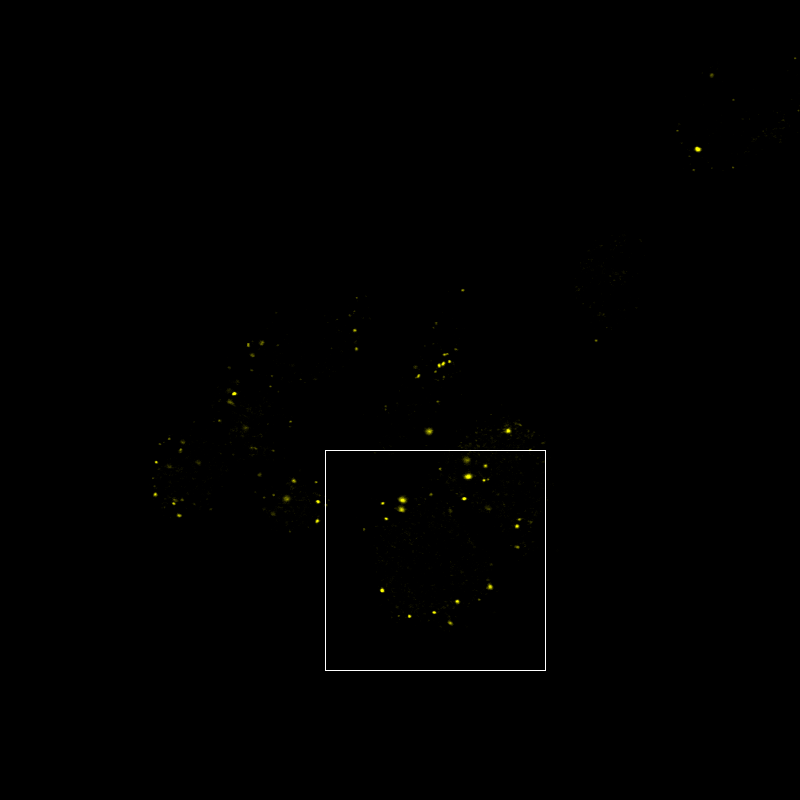

Supplement: Supplementary file 4 — Source data Fig. 1 [file 44318_2024_120_MOESM4_ESM.zip › Figure 1/1G/Oligomycin+Liposome/LSM14A.tif]

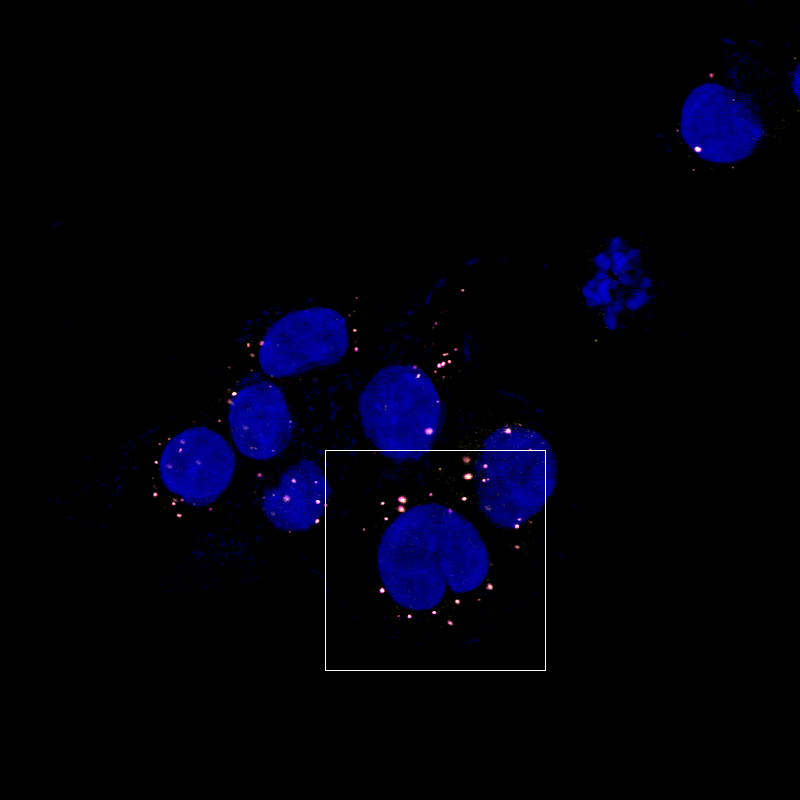

Supplement: Supplementary file 4 — Source data Fig. 1 [file 44318_2024_120_MOESM4_ESM.zip › Figure 1/1G/Oligomycin+Liposome/Merge.tif]

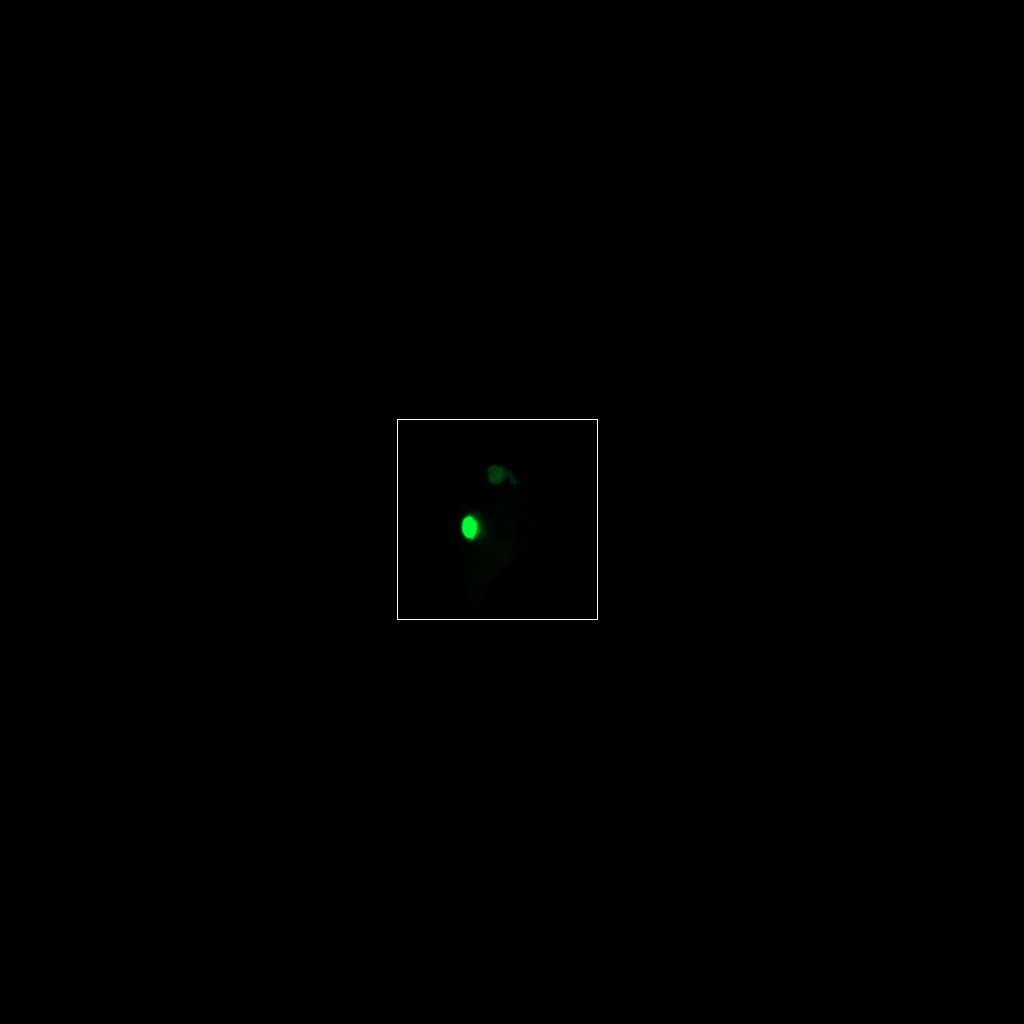

Supplement: Supplementary file 4 — Source data Fig. 1 [file 44318_2024_120_MOESM4_ESM.zip › Figure 1/1I/Mock/30s.tif]

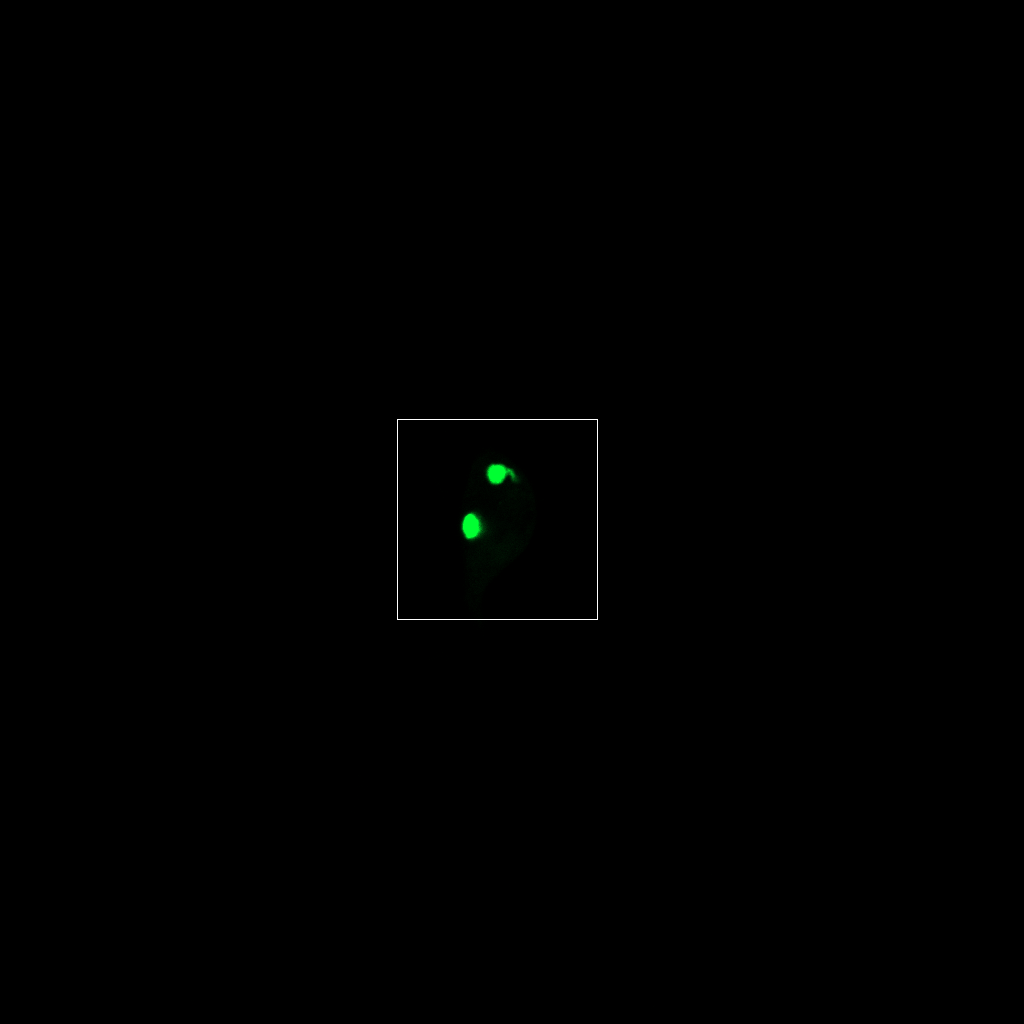

Supplement: Supplementary file 4 — Source data Fig. 1 [file 44318_2024_120_MOESM4_ESM.zip › Figure 1/1I/Mock/Pre.tif]

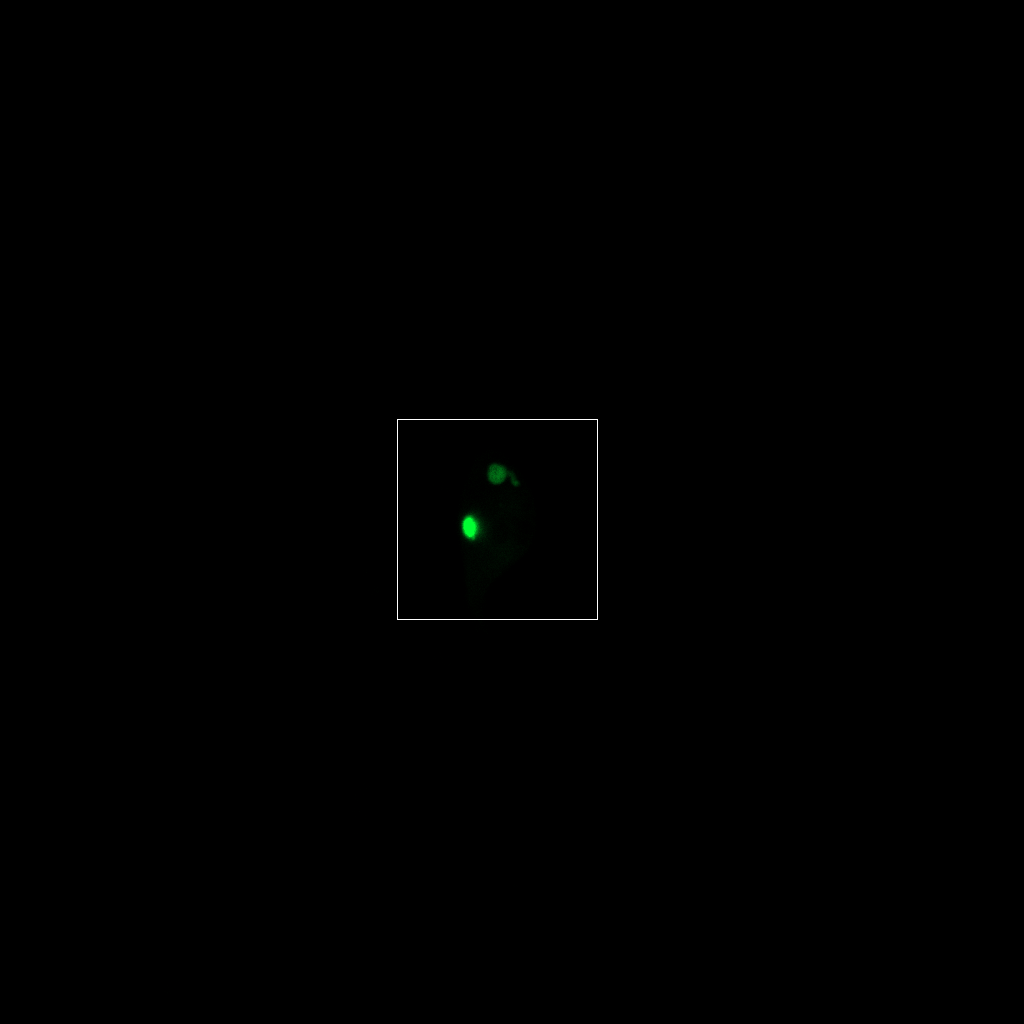

Supplement: Supplementary file 4 — Source data Fig. 1 [file 44318_2024_120_MOESM4_ESM.zip › Figure 1/1I/Mock/60s.tif]

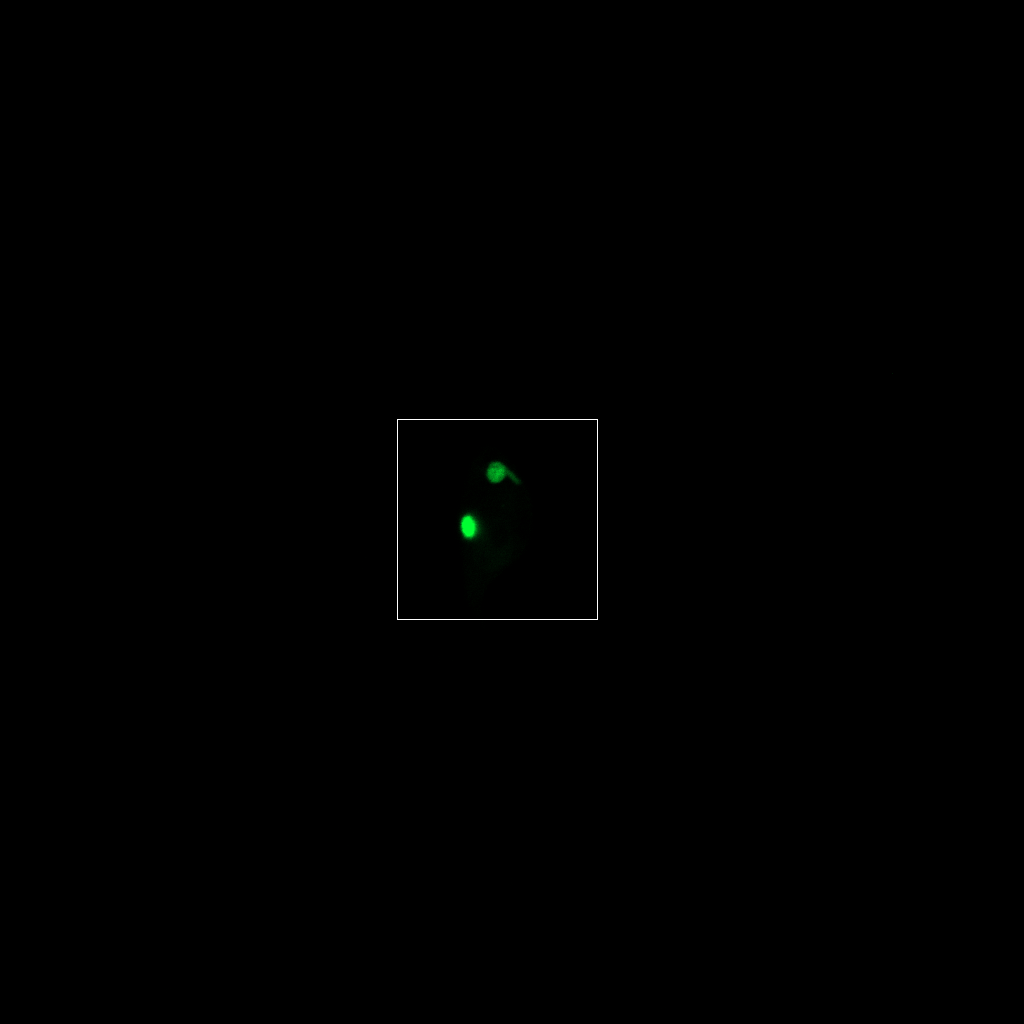

Supplement: Supplementary file 4 — Source data Fig. 1 [file 44318_2024_120_MOESM4_ESM.zip › Figure 1/1I/Mock/120s.tif]

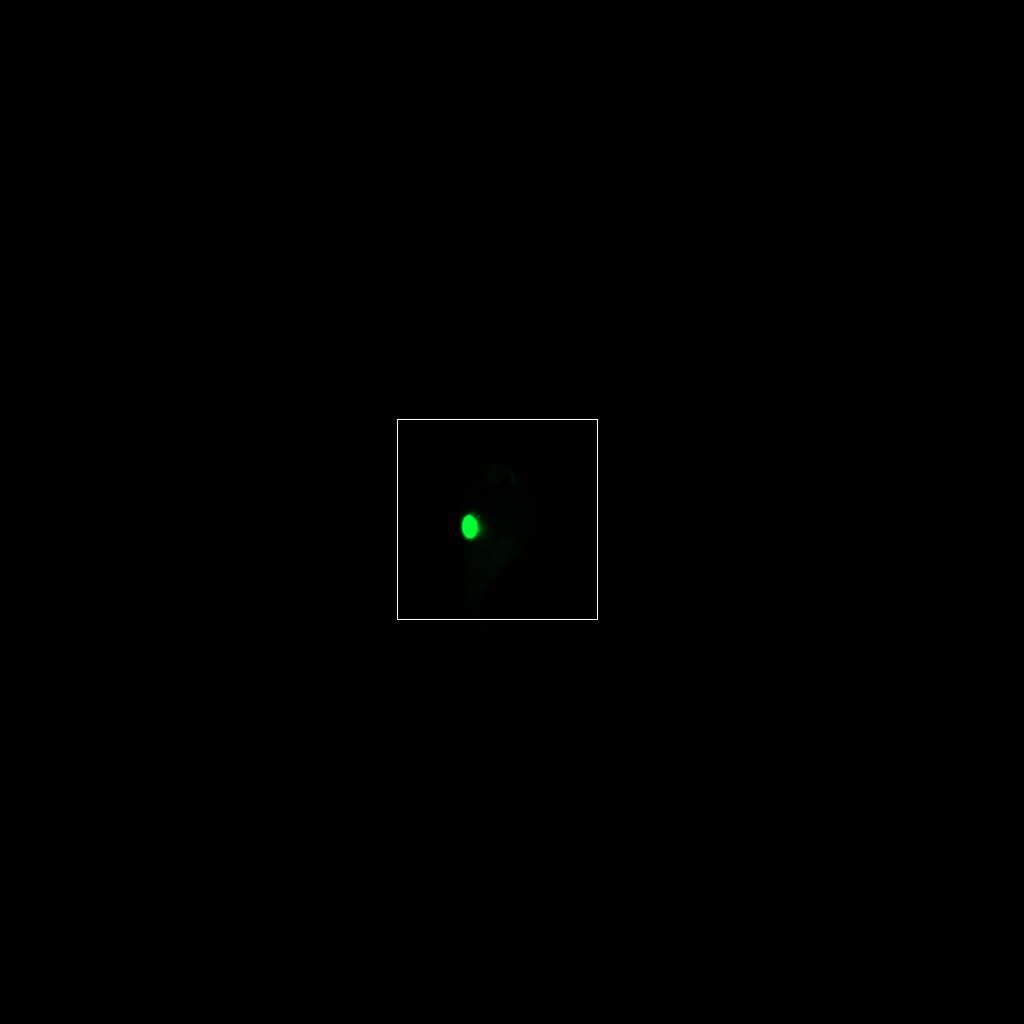

Supplement: Supplementary file 4 — Source data Fig. 1 [file 44318_2024_120_MOESM4_ESM.zip › Figure 1/1I/Mock/Bleaching.tif]

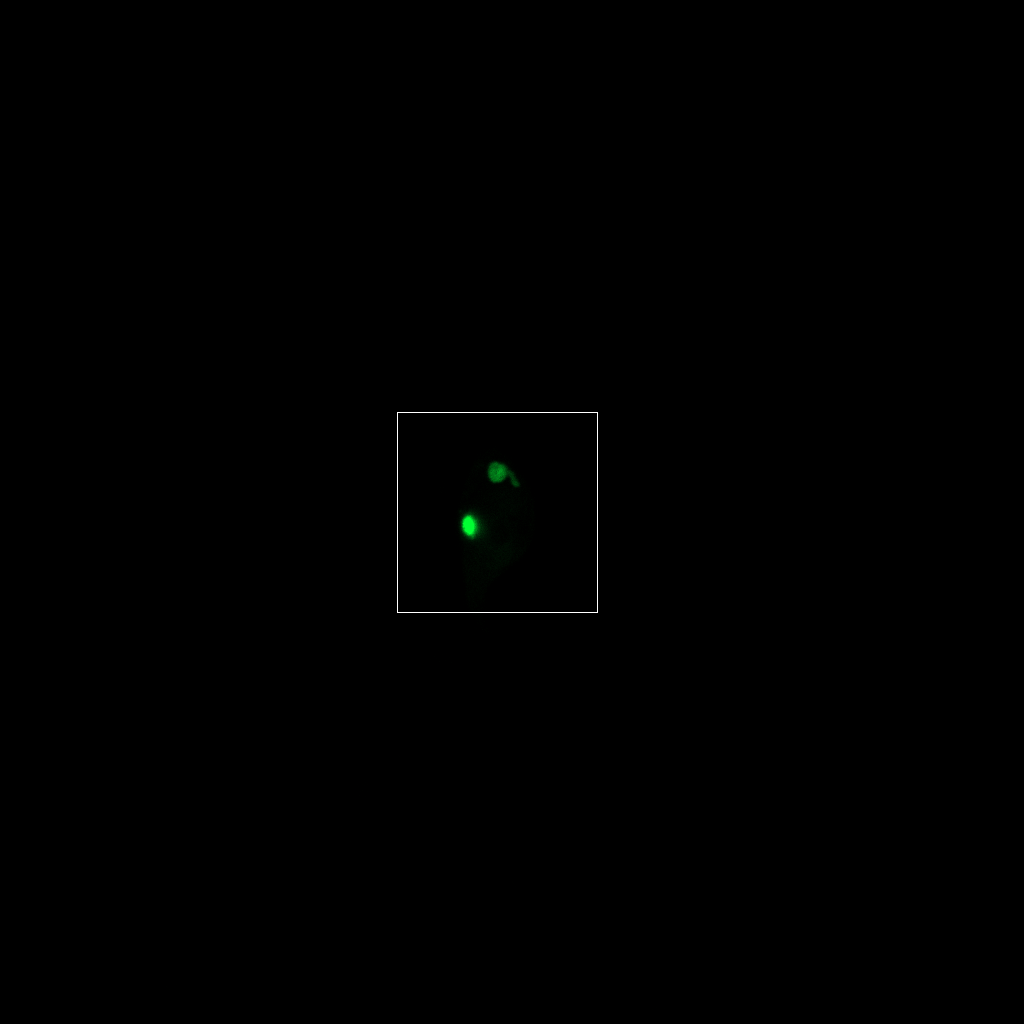

Supplement: Supplementary file 4 — Source data Fig. 1 [file 44318_2024_120_MOESM4_ESM.zip › Figure 1/1I/Mock/90s.tif]

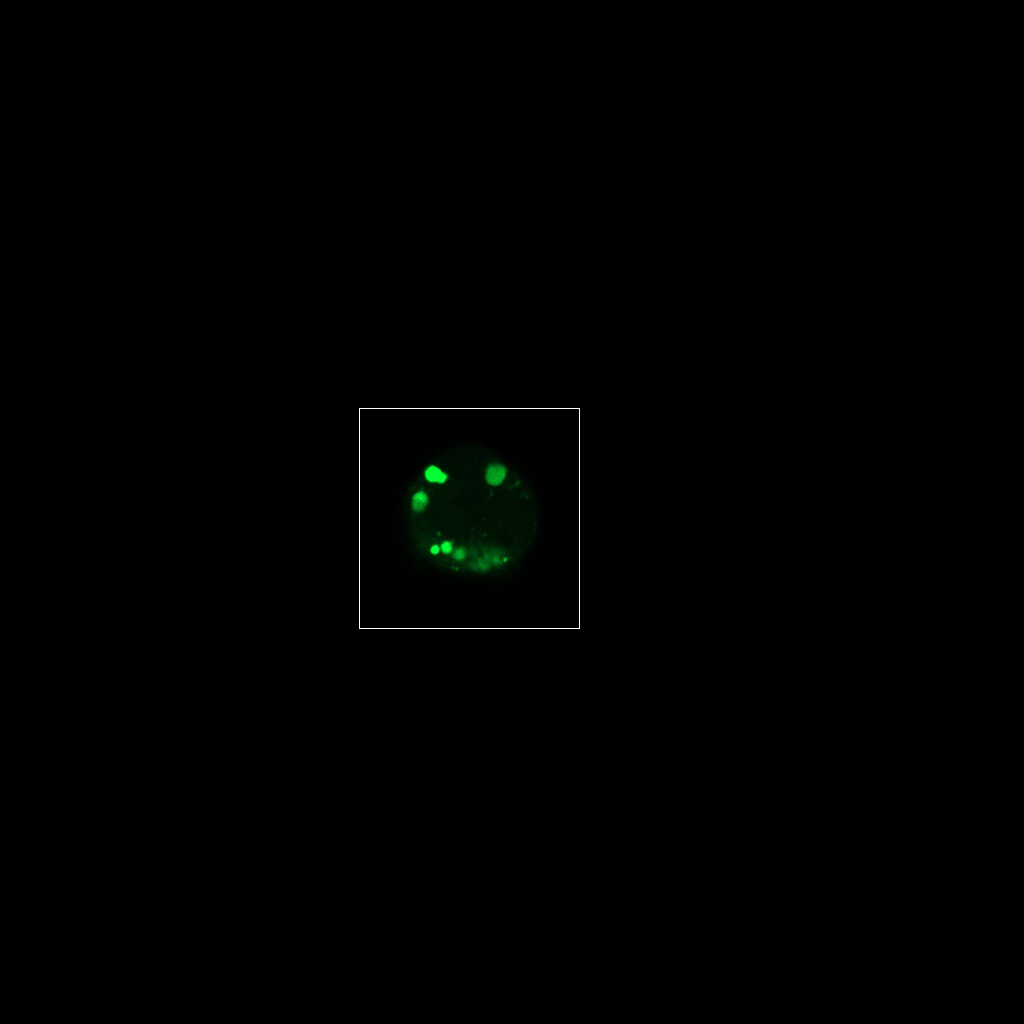

Supplement: Supplementary file 4 — Source data Fig. 1 [file 44318_2024_120_MOESM4_ESM.zip › Figure 1/1I/Oligomycin/30s.tif]

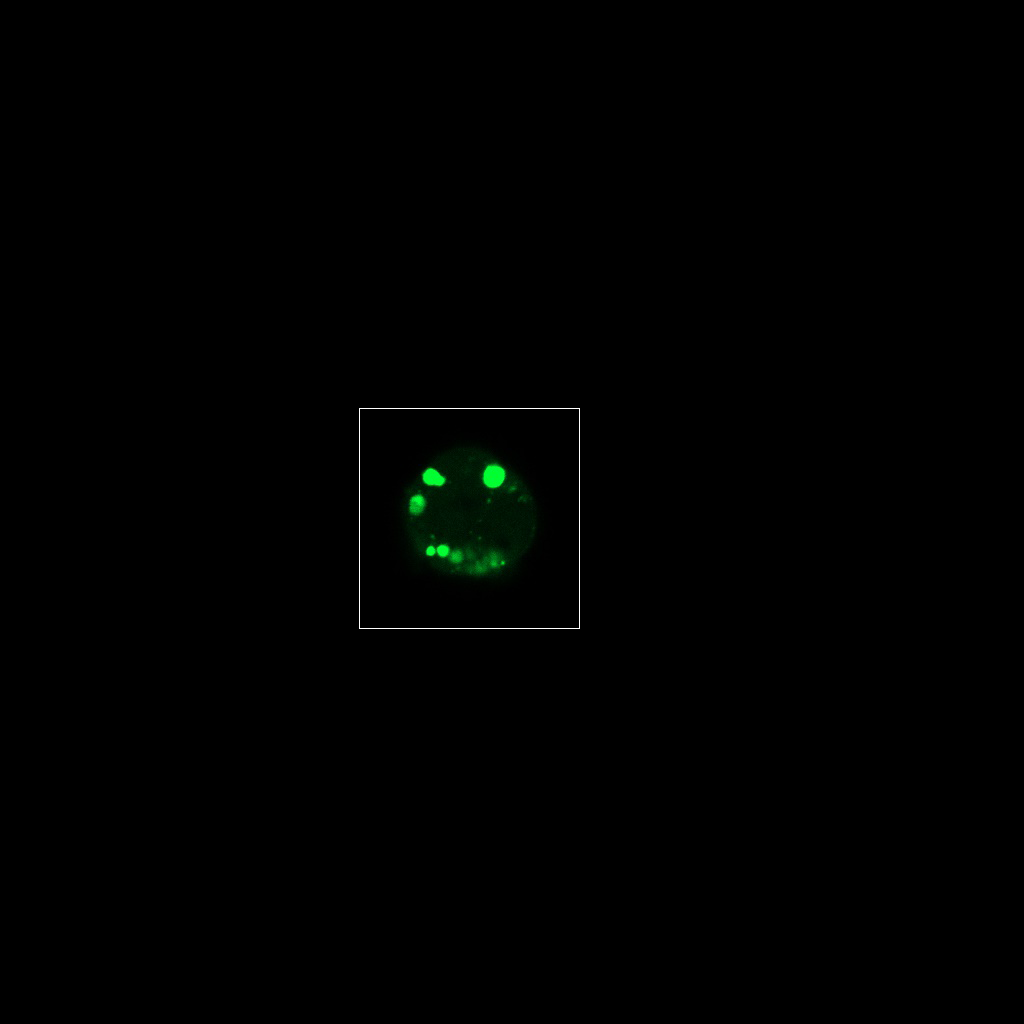

Supplement: Supplementary file 4 — Source data Fig. 1 [file 44318_2024_120_MOESM4_ESM.zip › Figure 1/1I/Oligomycin/Pre.tif]

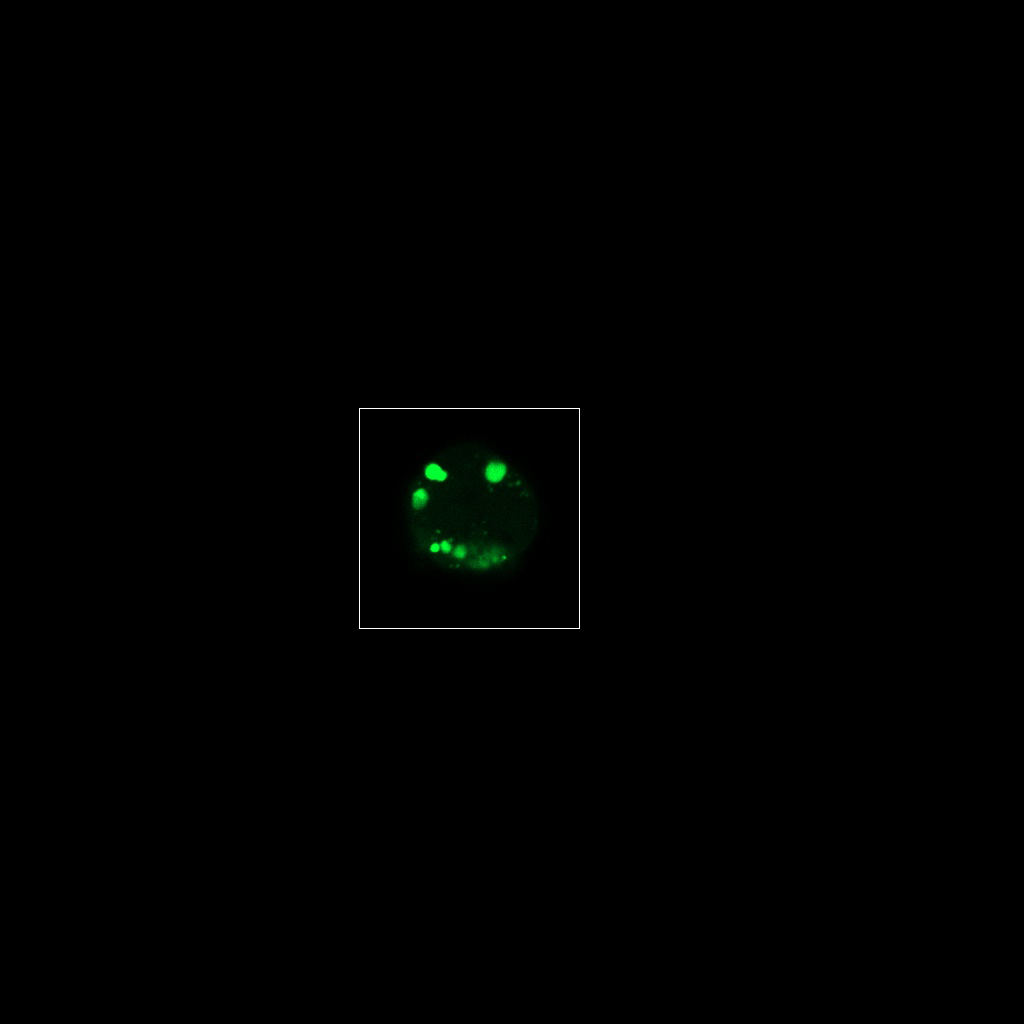

Supplement: Supplementary file 4 — Source data Fig. 1 [file 44318_2024_120_MOESM4_ESM.zip › Figure 1/1I/Oligomycin/60s.tif]

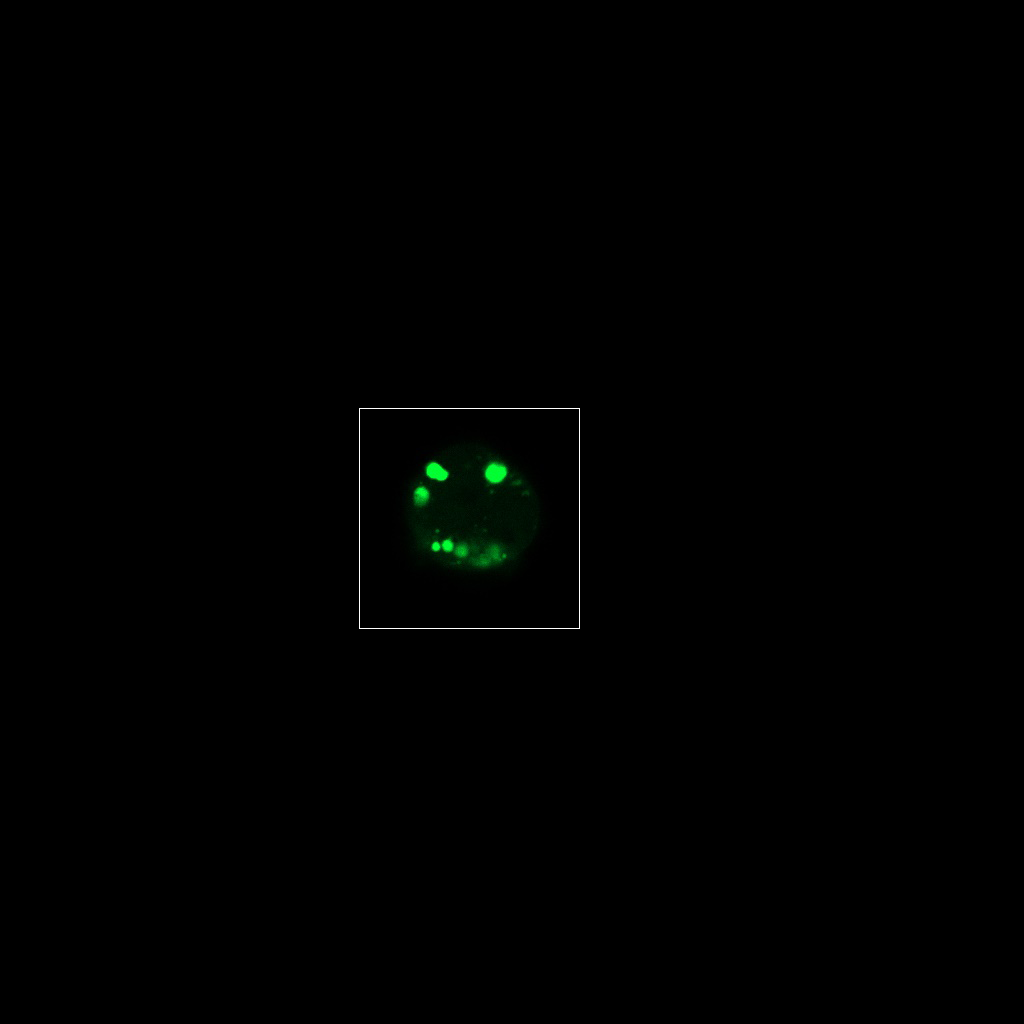

Supplement: Supplementary file 4 — Source data Fig. 1 [file 44318_2024_120_MOESM4_ESM.zip › Figure 1/1I/Oligomycin/120s.tif]

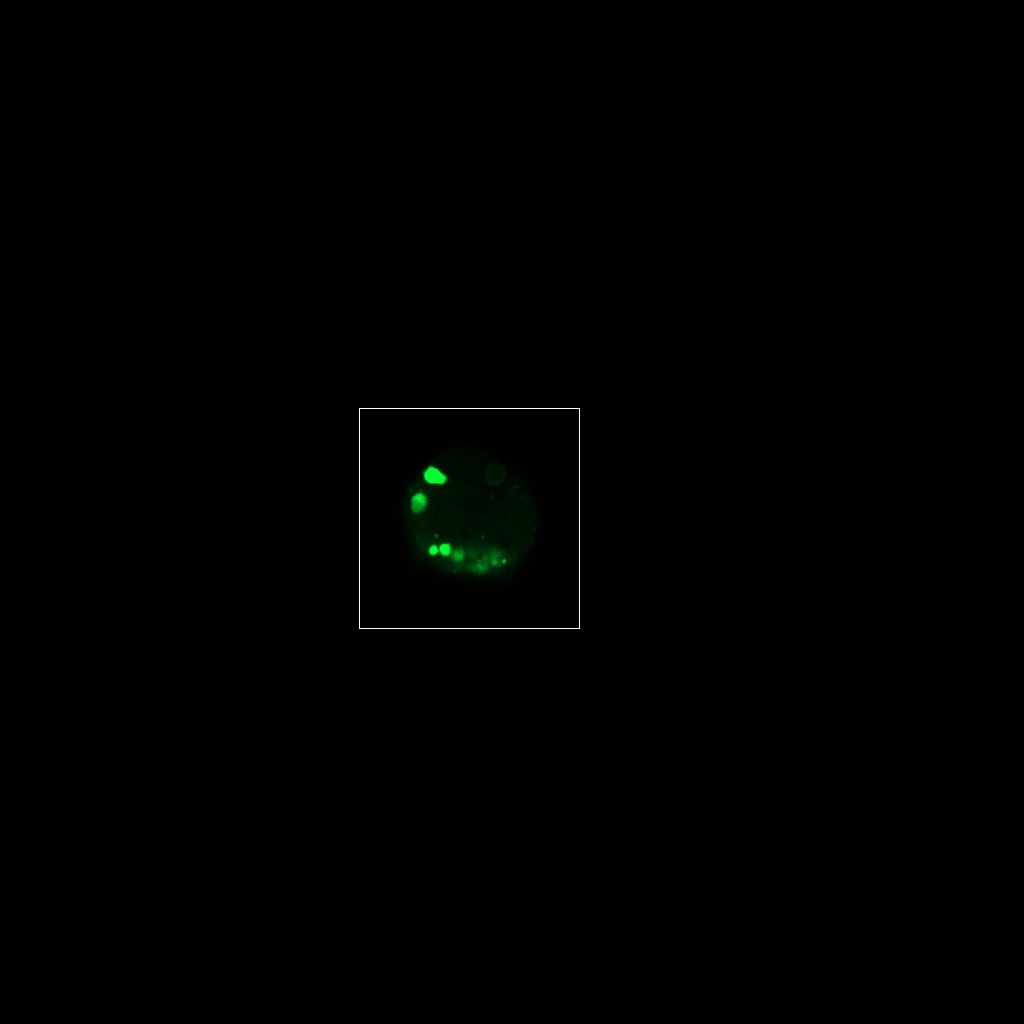

Supplement: Supplementary file 4 — Source data Fig. 1 [file 44318_2024_120_MOESM4_ESM.zip › Figure 1/1I/Oligomycin/Bleaching.tif]

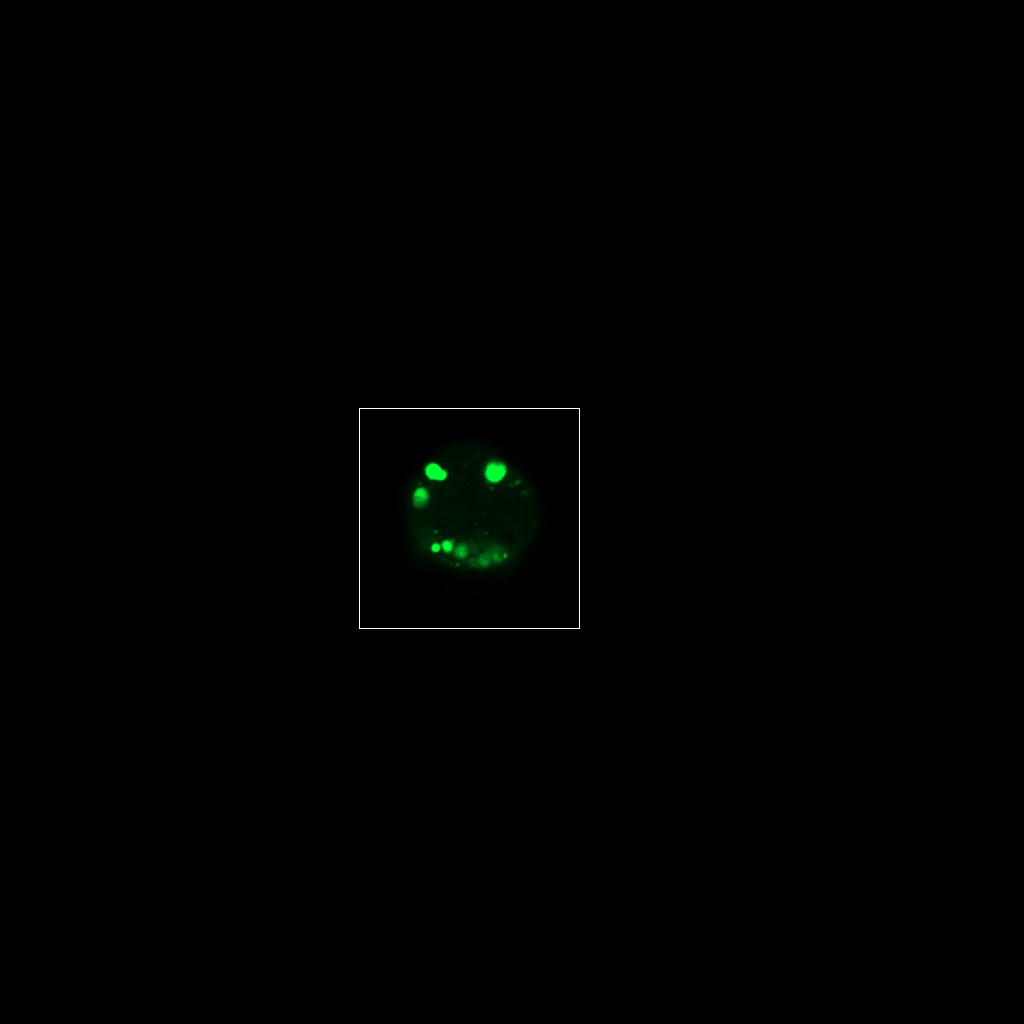

Supplement: Supplementary file 4 — Source data Fig. 1 [file 44318_2024_120_MOESM4_ESM.zip › Figure 1/1I/Oligomycin/90s.tif]

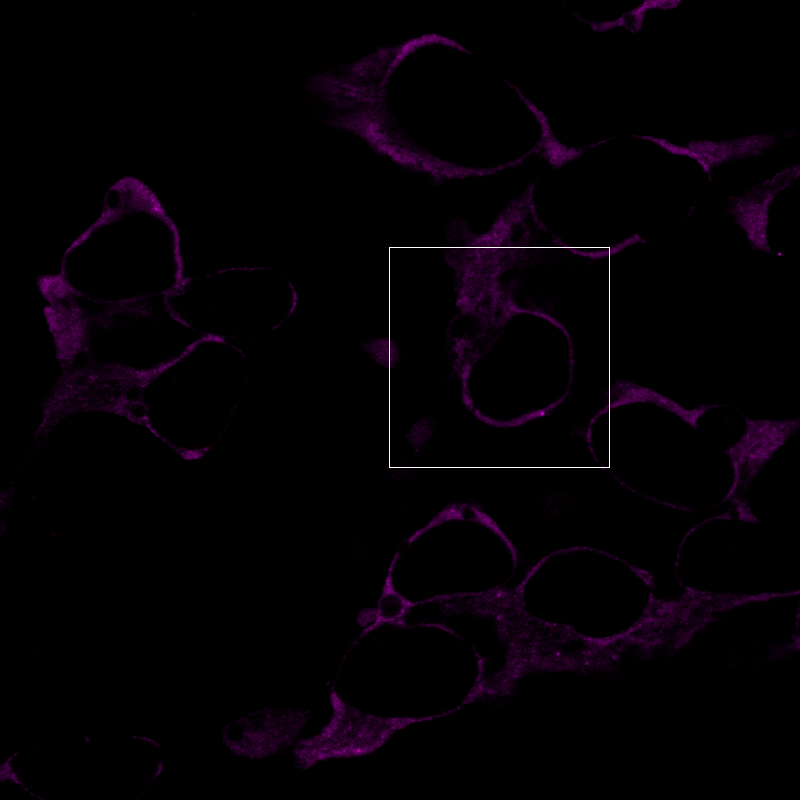

Supplement: Supplementary file 4 — Source data Fig. 1 [file 44318_2024_120_MOESM4_ESM.zip › Figure 1/1E/Heat shock/ATPsome/DCP1A.tif]

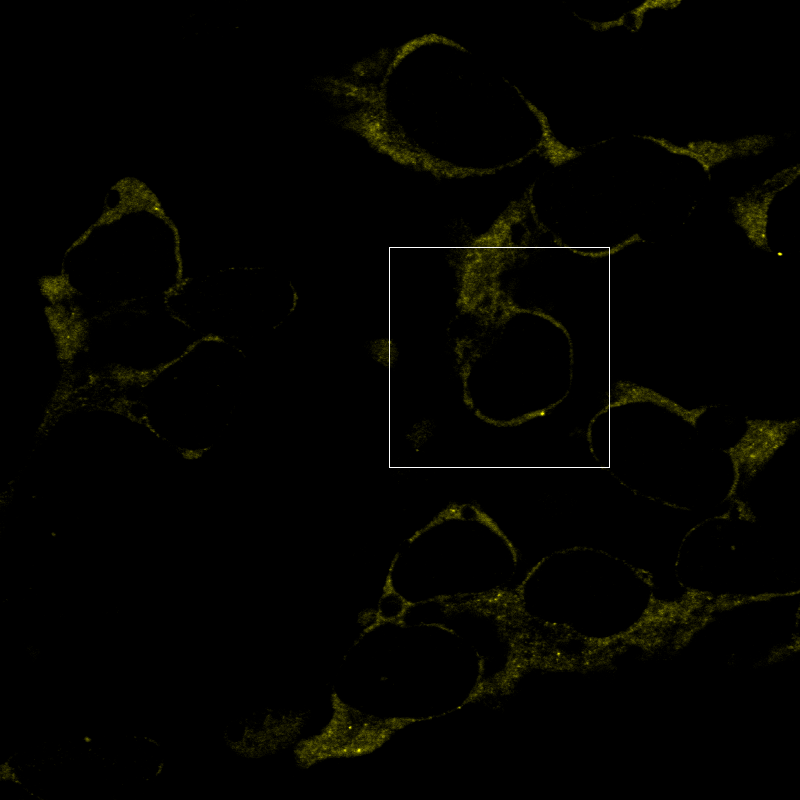

Supplement: Supplementary file 4 — Source data Fig. 1 [file 44318_2024_120_MOESM4_ESM.zip › Figure 1/1E/Heat shock/ATPsome/LSM14A.tif]

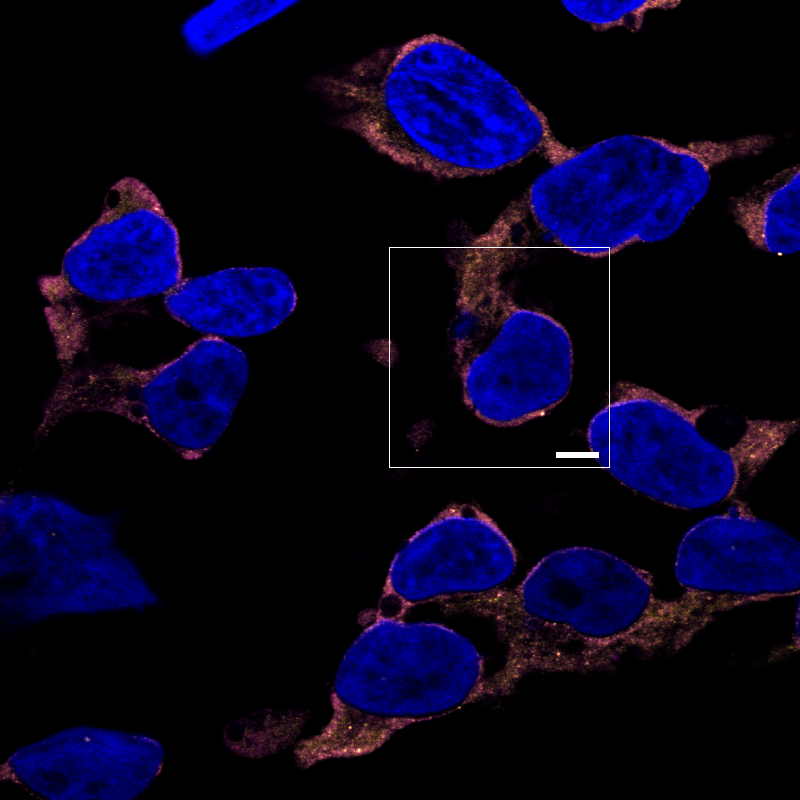

Supplement: Supplementary file 4 — Source data Fig. 1 [file 44318_2024_120_MOESM4_ESM.zip › Figure 1/1E/Heat shock/ATPsome/Merge.tif]

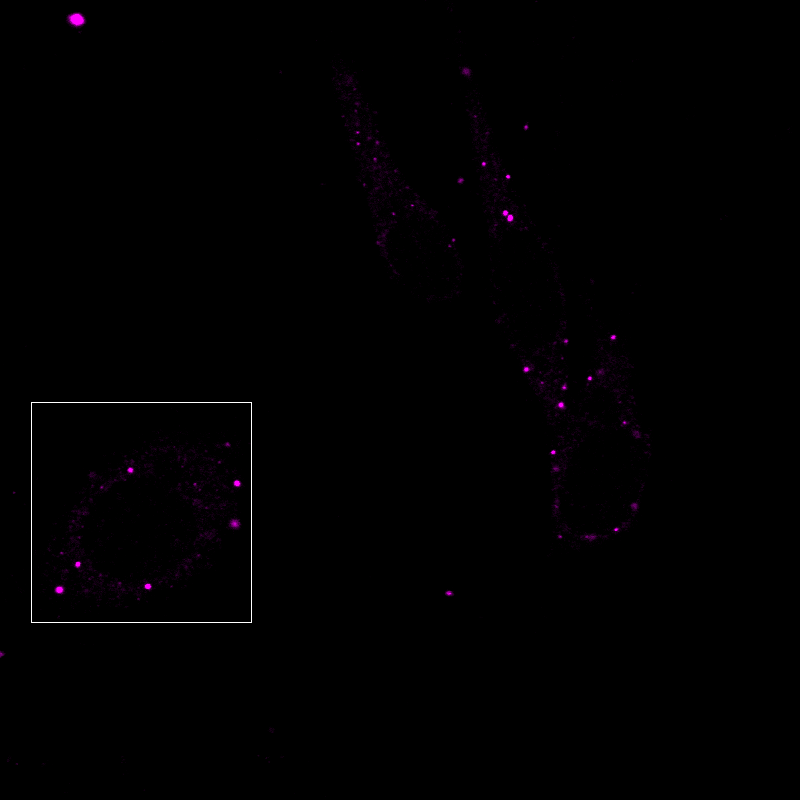

Supplement: Supplementary file 4 — Source data Fig. 1 [file 44318_2024_120_MOESM4_ESM.zip › Figure 1/1E/Heat shock/Liposome/DCP1A.tif]

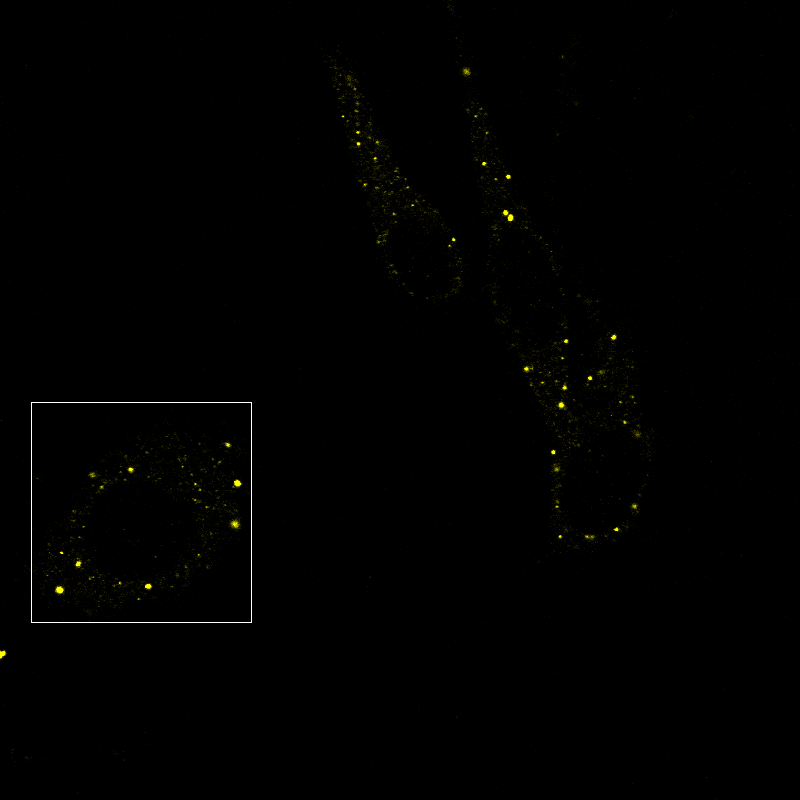

Supplement: Supplementary file 4 — Source data Fig. 1 [file 44318_2024_120_MOESM4_ESM.zip › Figure 1/1E/Heat shock/Liposome/LSM14A.tif]

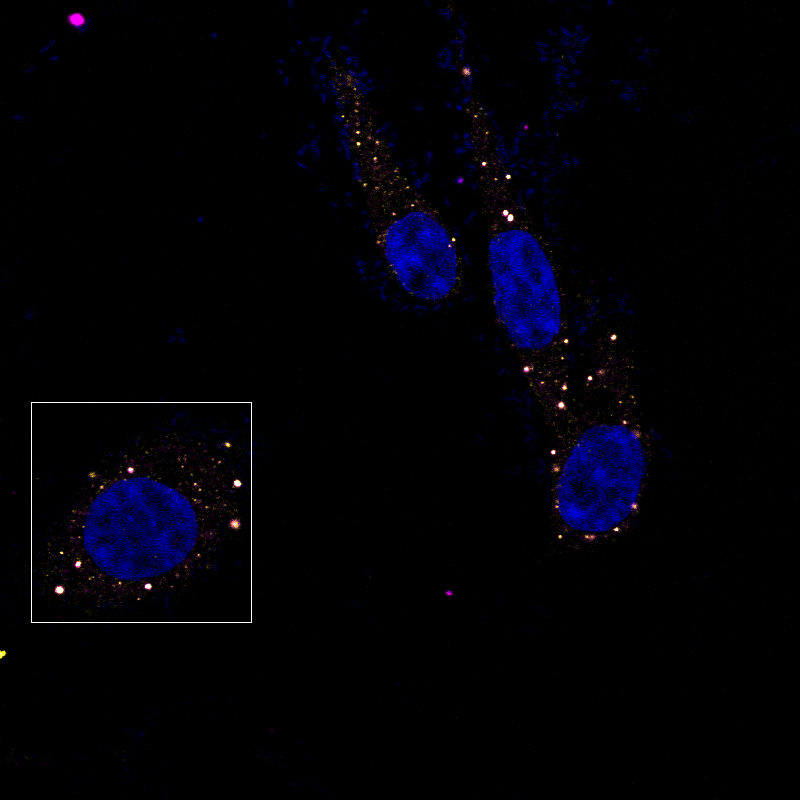

Supplement: Supplementary file 4 — Source data Fig. 1 [file 44318_2024_120_MOESM4_ESM.zip › Figure 1/1E/Heat shock/Liposome/Merge.tif]

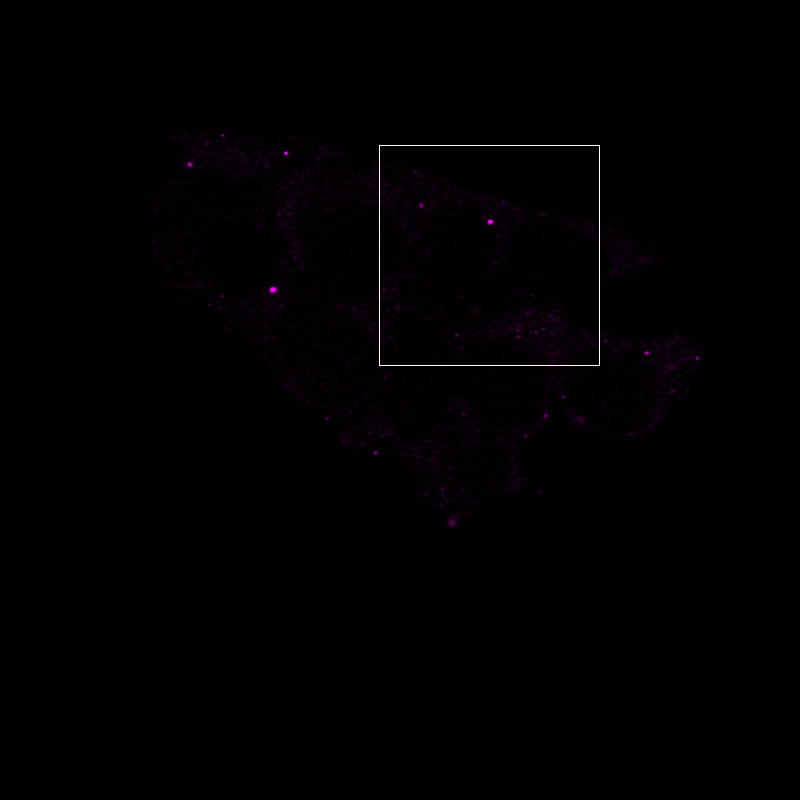

Supplement: Supplementary file 4 — Source data Fig. 1 [file 44318_2024_120_MOESM4_ESM.zip › Figure 1/1E/Ctrl/ATPsome/DCP1A.tif]

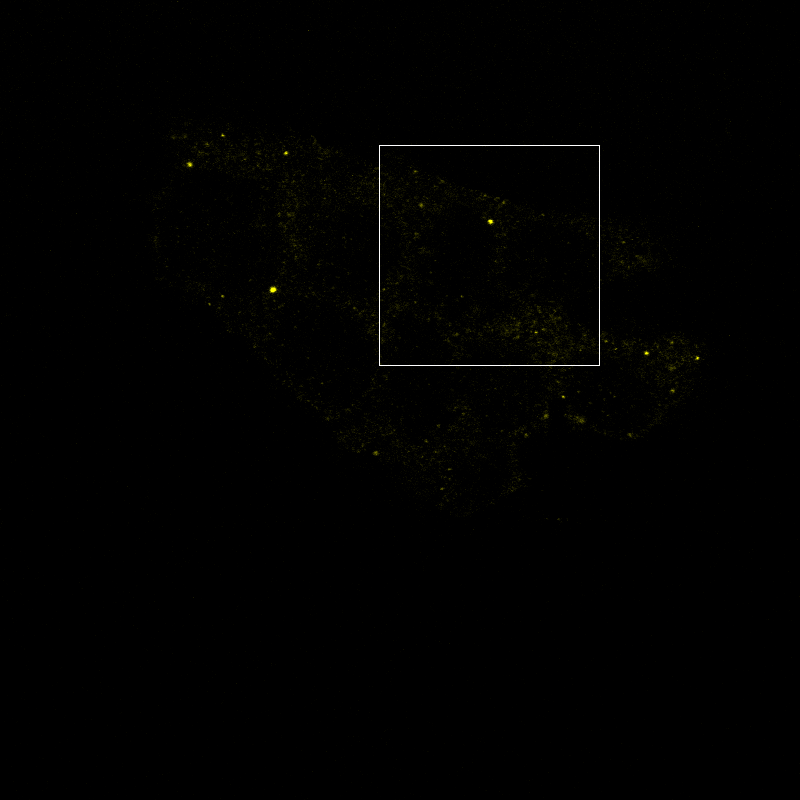

Supplement: Supplementary file 4 — Source data Fig. 1 [file 44318_2024_120_MOESM4_ESM.zip › Figure 1/1E/Ctrl/ATPsome/LSM14A.tif]

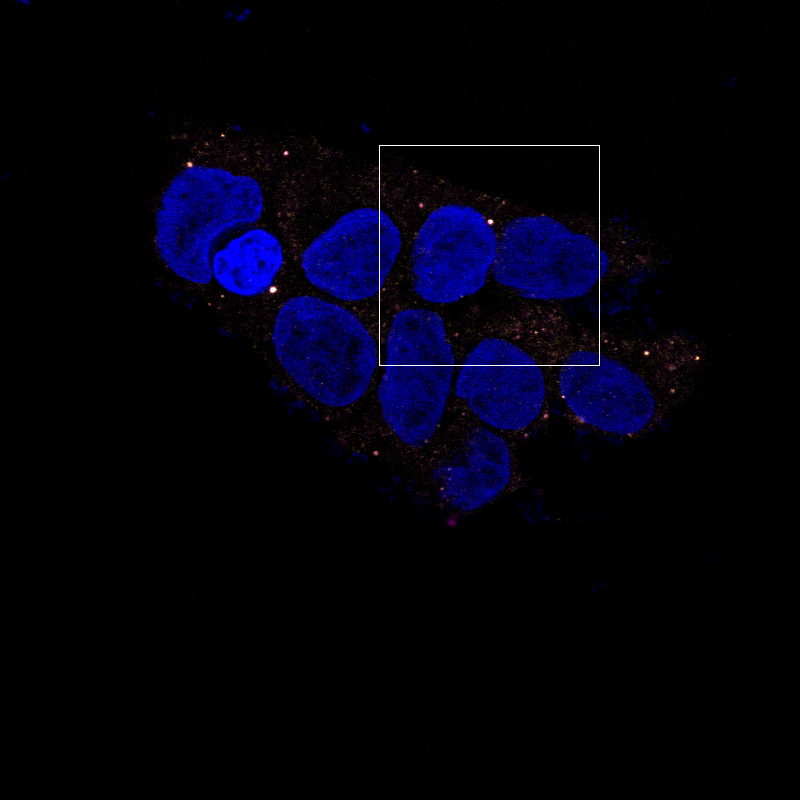

Supplement: Supplementary file 4 — Source data Fig. 1 [file 44318_2024_120_MOESM4_ESM.zip › Figure 1/1E/Ctrl/ATPsome/Merge.tif]

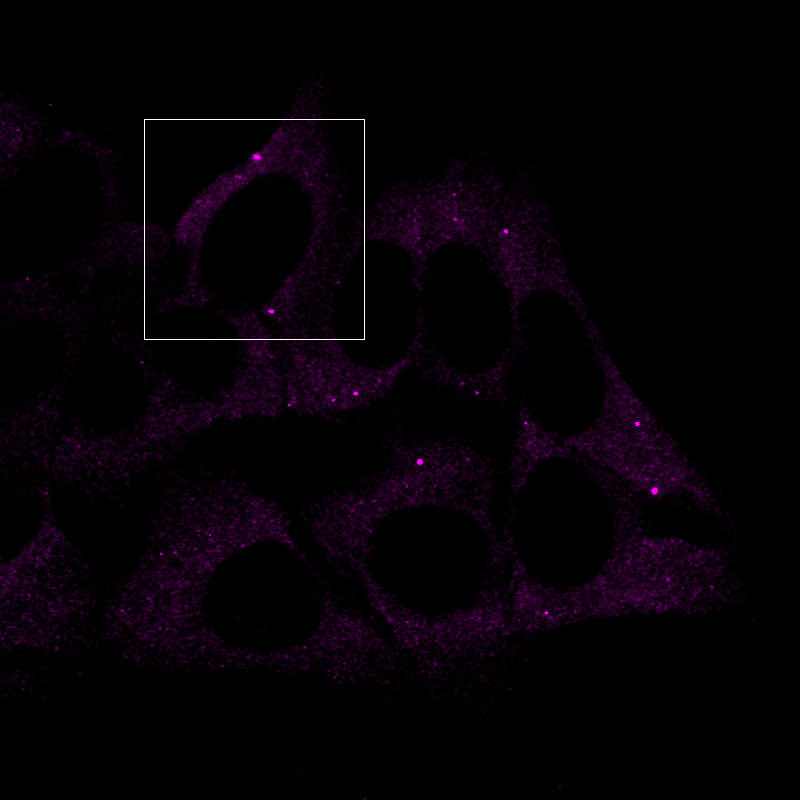

Supplement: Supplementary file 4 — Source data Fig. 1 [file 44318_2024_120_MOESM4_ESM.zip › Figure 1/1E/Ctrl/Liposome/DCP1A.tif]

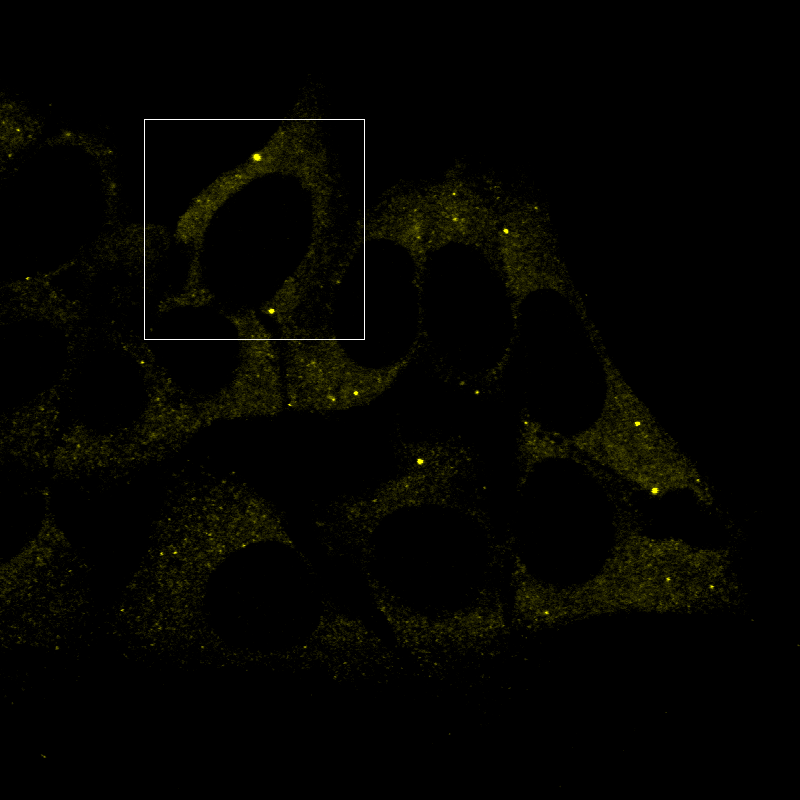

Supplement: Supplementary file 4 — Source data Fig. 1 [file 44318_2024_120_MOESM4_ESM.zip › Figure 1/1E/Ctrl/Liposome/LSM14A.tif]

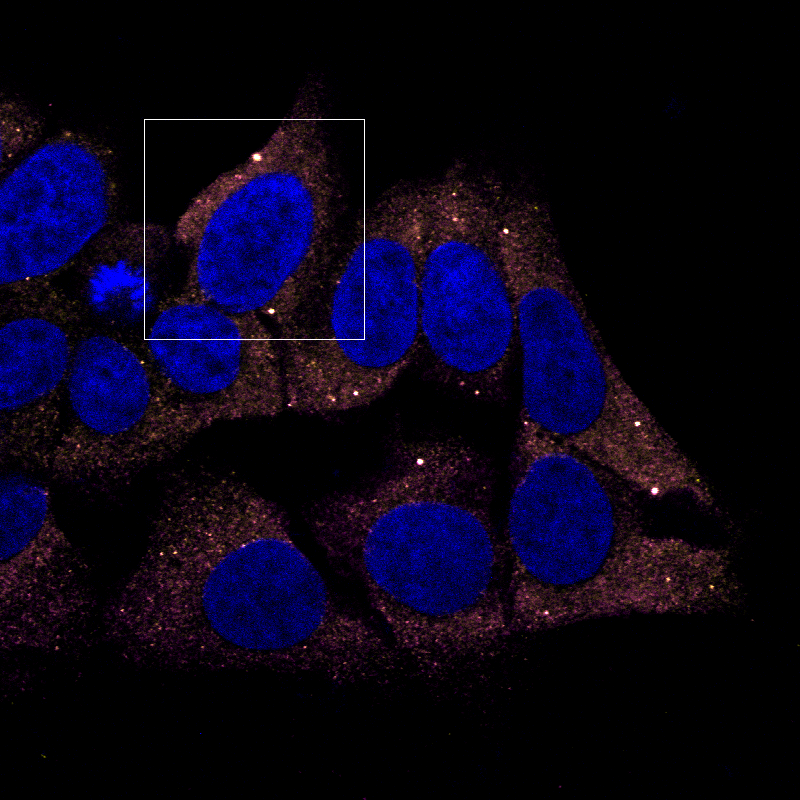

Supplement: Supplementary file 4 — Source data Fig. 1 [file 44318_2024_120_MOESM4_ESM.zip › Figure 1/1E/Ctrl/Liposome/Merge.tif]

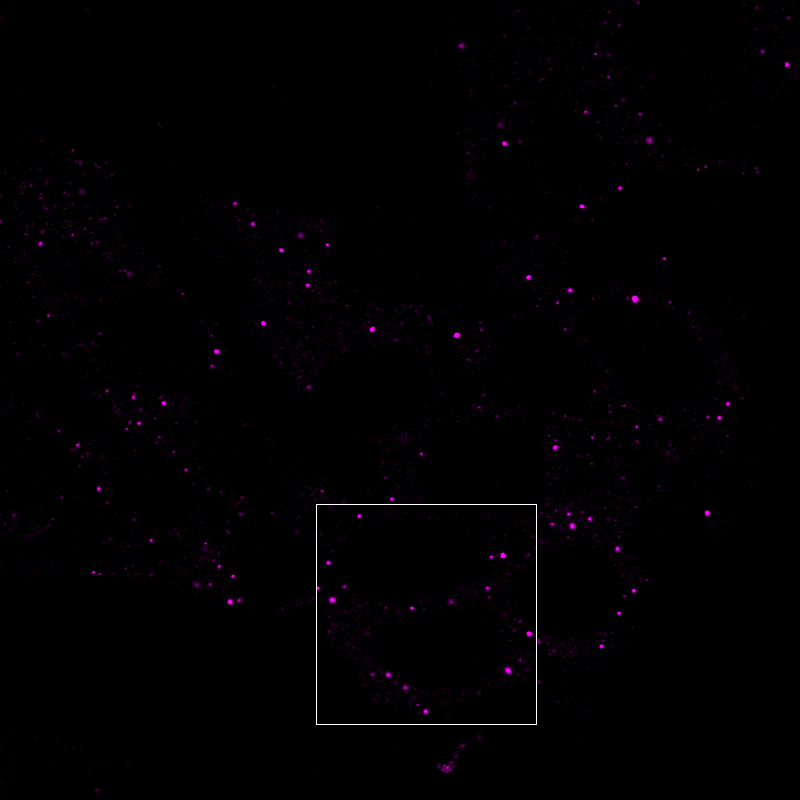

Supplement: Supplementary file 4 — Source data Fig. 1 [file 44318_2024_120_MOESM4_ESM.zip › Figure 1/1C/Heat shock/Mock/DCP1A.tif]

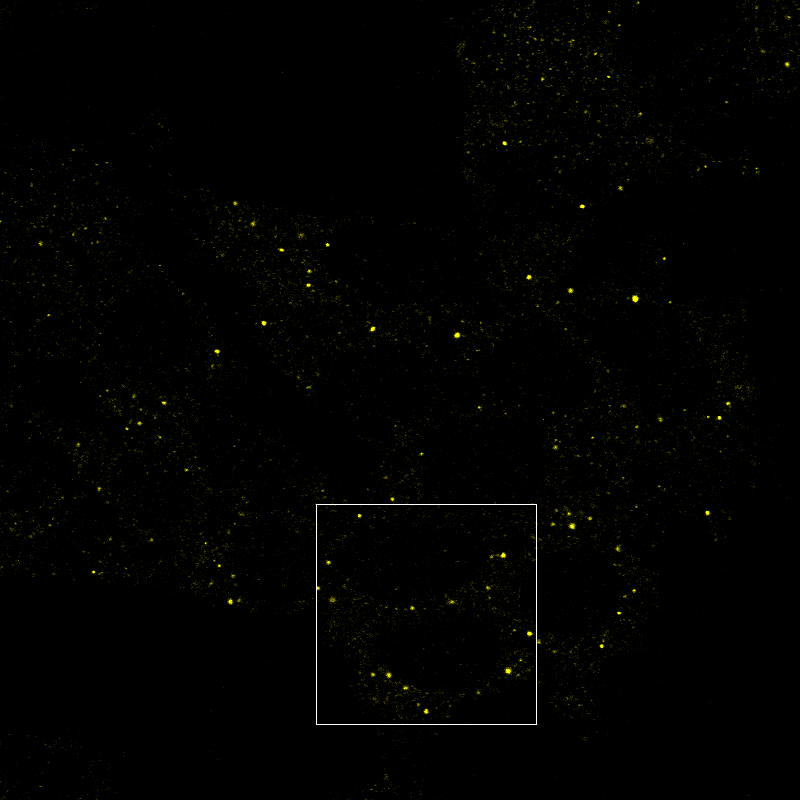

Supplement: Supplementary file 4 — Source data Fig. 1 [file 44318_2024_120_MOESM4_ESM.zip › Figure 1/1C/Heat shock/Mock/LSM14A.tif]

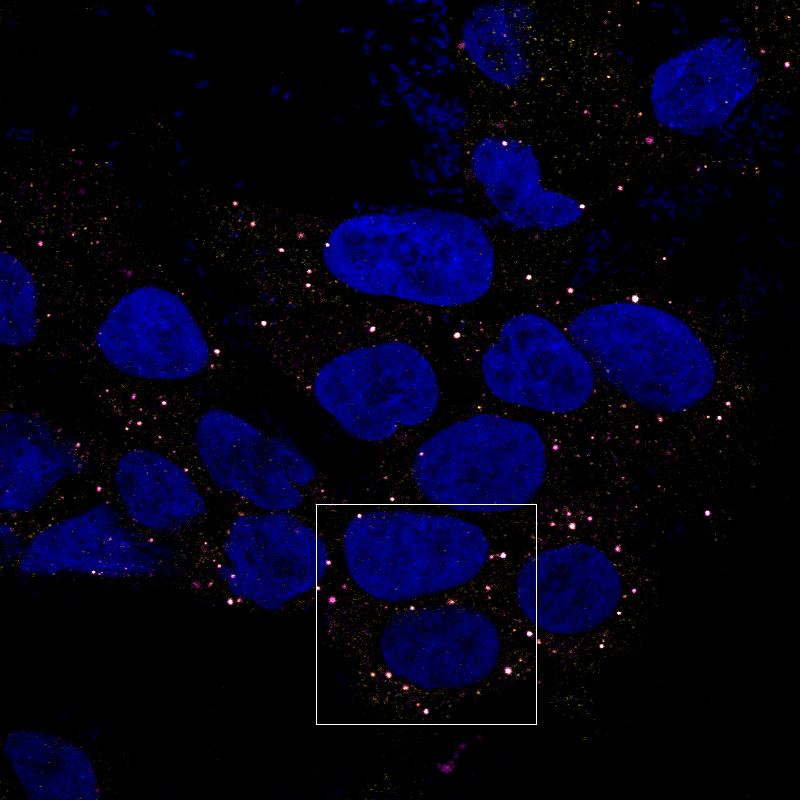

Supplement: Supplementary file 4 — Source data Fig. 1 [file 44318_2024_120_MOESM4_ESM.zip › Figure 1/1C/Heat shock/Mock/Merge.tif]

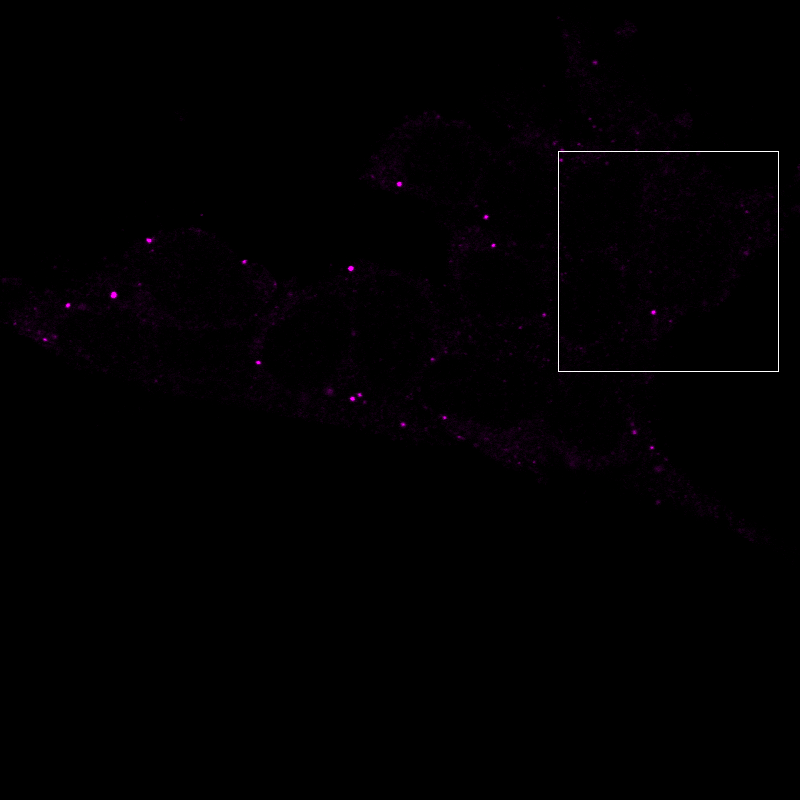

Supplement: Supplementary file 4 — Source data Fig. 1 [file 44318_2024_120_MOESM4_ESM.zip › Figure 1/1C/Heat shock/NADH+CoQ10/DCP1A.tif]

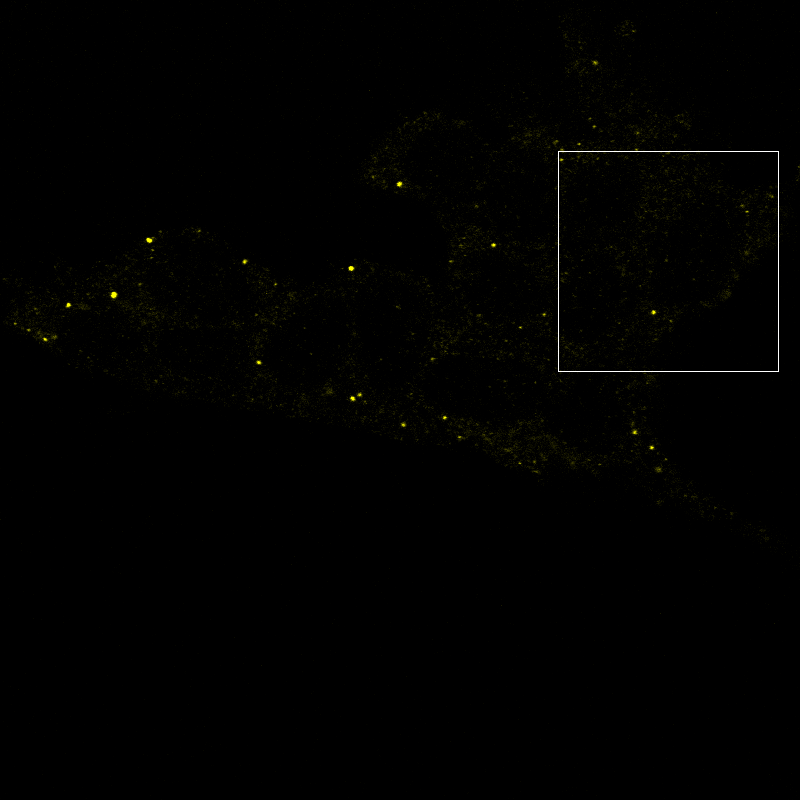

Supplement: Supplementary file 4 — Source data Fig. 1 [file 44318_2024_120_MOESM4_ESM.zip › Figure 1/1C/Heat shock/NADH+CoQ10/LSM14A.tif]

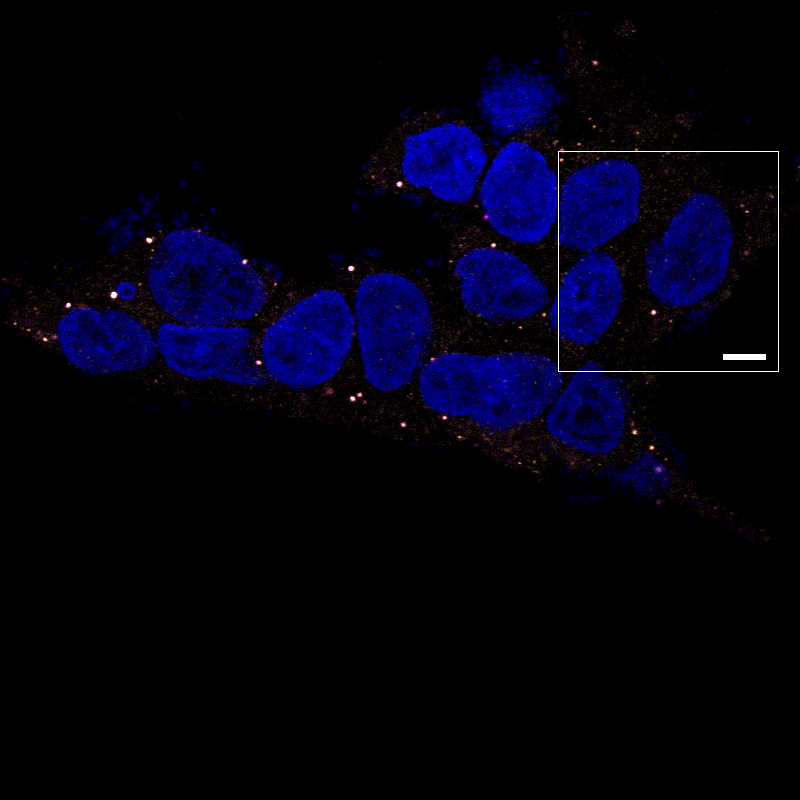

Supplement: Supplementary file 4 — Source data Fig. 1 [file 44318_2024_120_MOESM4_ESM.zip › Figure 1/1C/Heat shock/NADH+CoQ10/Merge.tif]

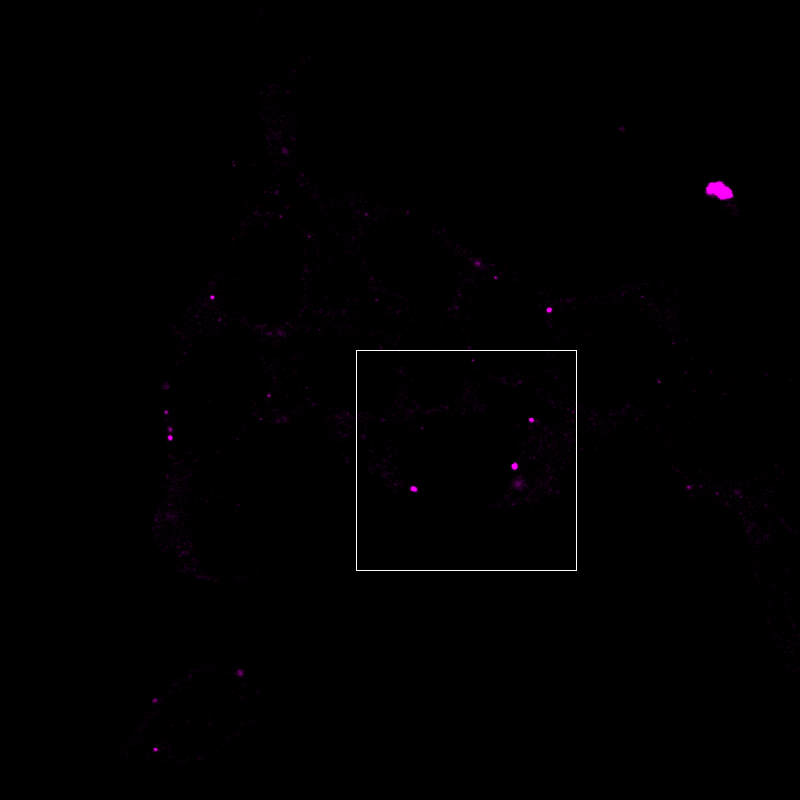

Supplement: Supplementary file 4 — Source data Fig. 1 [file 44318_2024_120_MOESM4_ESM.zip › Figure 1/1C/Ctrl/Mock/DCP1A.tif]

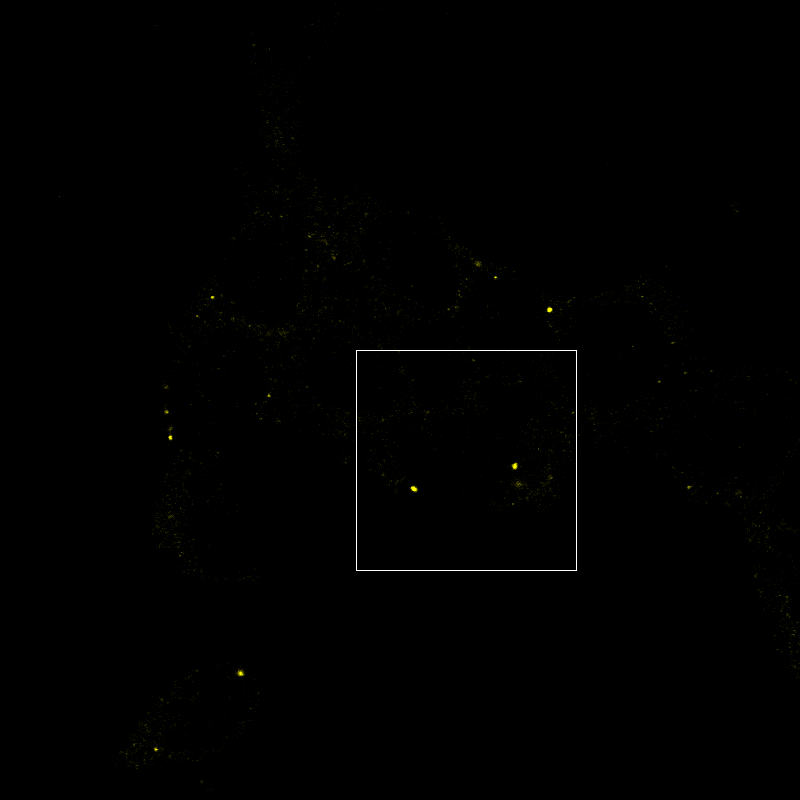

Supplement: Supplementary file 4 — Source data Fig. 1 [file 44318_2024_120_MOESM4_ESM.zip › Figure 1/1C/Ctrl/Mock/LSM14A.tif]

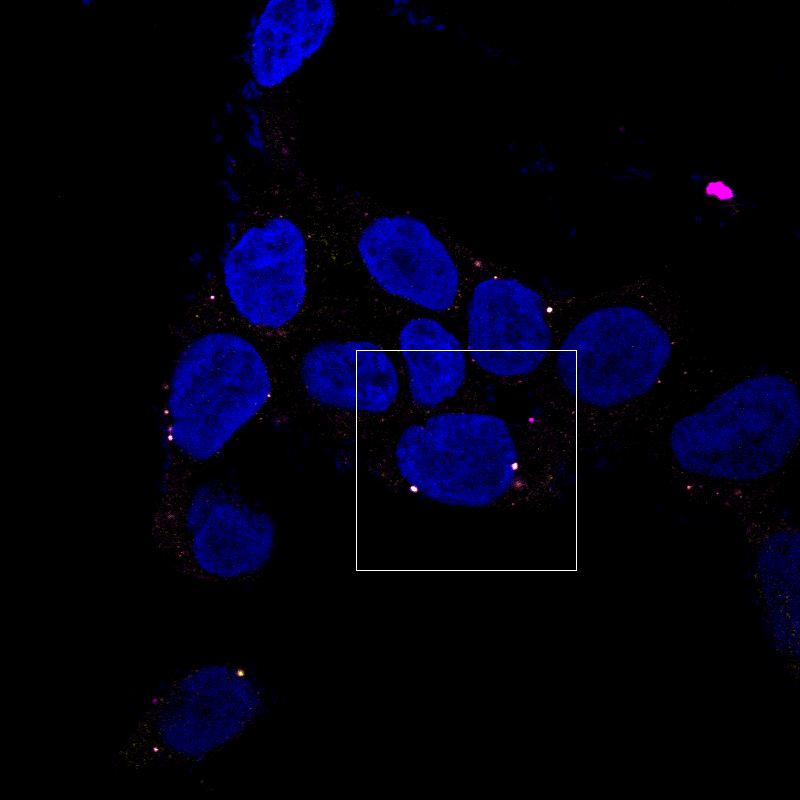

Supplement: Supplementary file 4 — Source data Fig. 1 [file 44318_2024_120_MOESM4_ESM.zip › Figure 1/1C/Ctrl/Mock/Merge.tif]

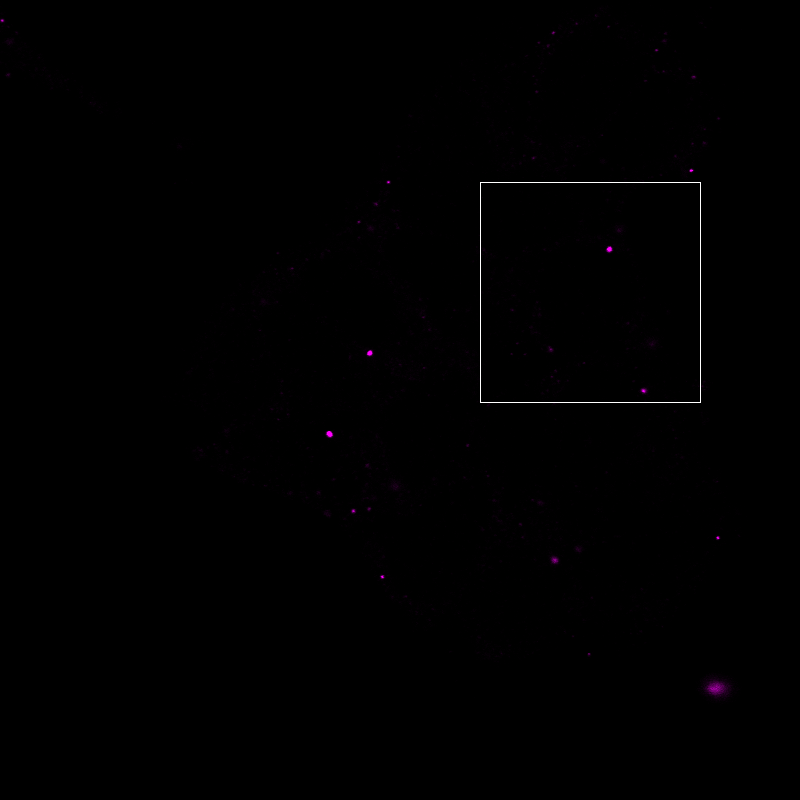

Supplement: Supplementary file 4 — Source data Fig. 1 [file 44318_2024_120_MOESM4_ESM.zip › Figure 1/1C/Ctrl/NADH+CoQ10/DCP1A.tif]

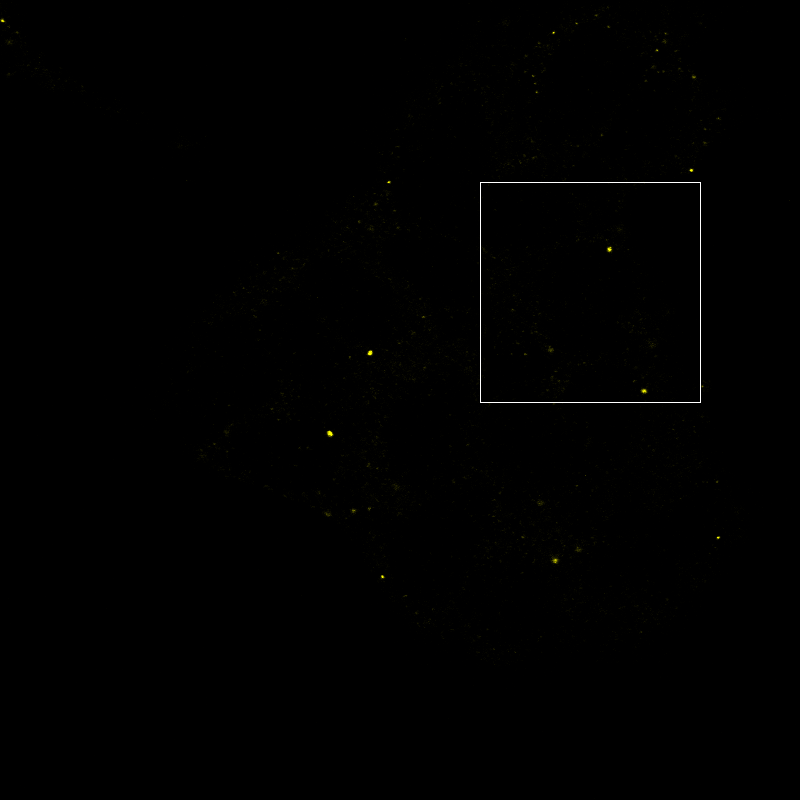

Supplement: Supplementary file 4 — Source data Fig. 1 [file 44318_2024_120_MOESM4_ESM.zip › Figure 1/1C/Ctrl/NADH+CoQ10/LSM14A.tif]

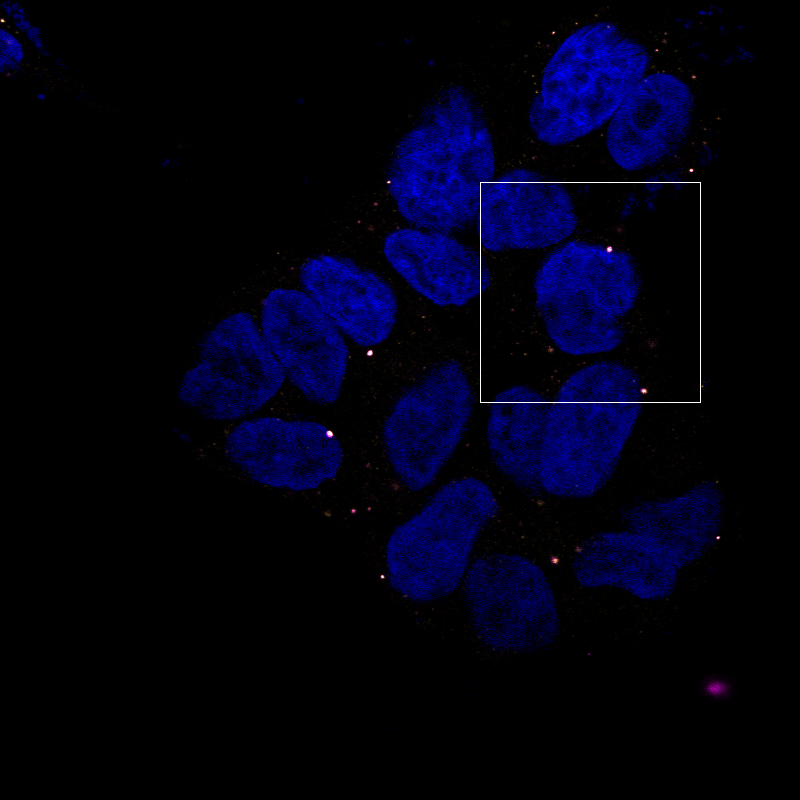

Supplement: Supplementary file 4 — Source data Fig. 1 [file 44318_2024_120_MOESM4_ESM.zip › Figure 1/1C/Ctrl/NADH+CoQ10/Merge.tif]

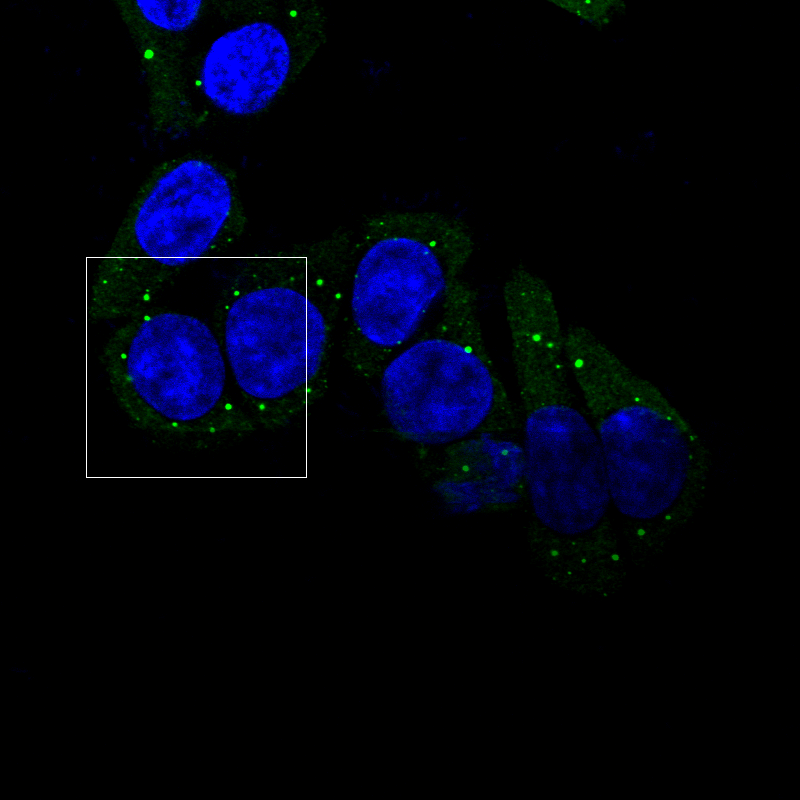

Supplement: Supplementary file 5 — Source data Fig. 2 [file 44318_2024_120_MOESM5_ESM.zip › Figure 2/2I/Ub-K48R.tif]

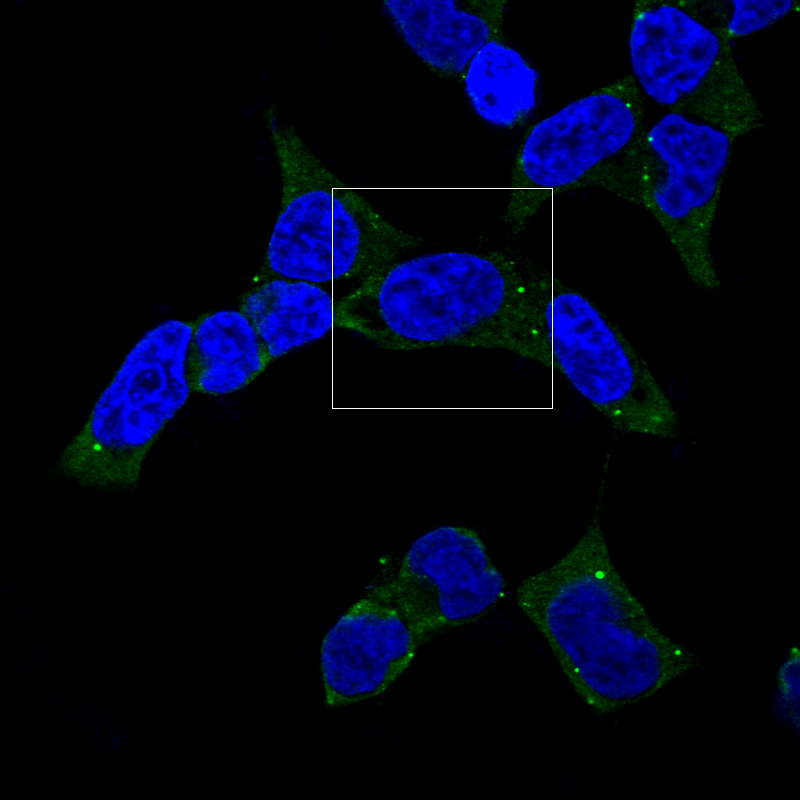

Supplement: Supplementary file 5 — Source data Fig. 2 [file 44318_2024_120_MOESM5_ESM.zip › Figure 2/2I/vector.tif]

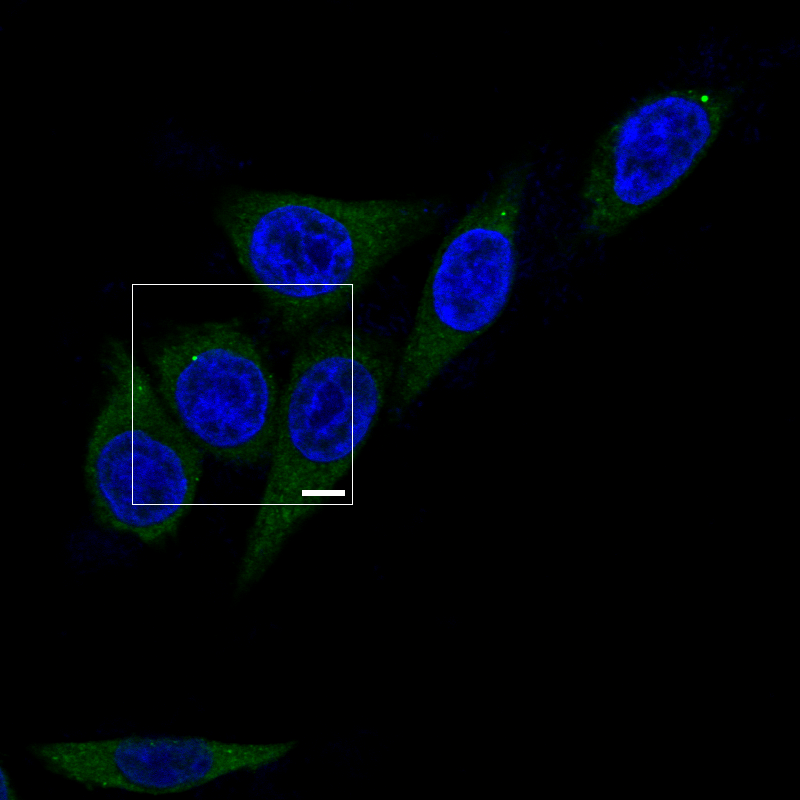

Supplement: Supplementary file 5 — Source data Fig. 2 [file 44318_2024_120_MOESM5_ESM.zip › Figure 2/2I/Ub-K63R.tif]

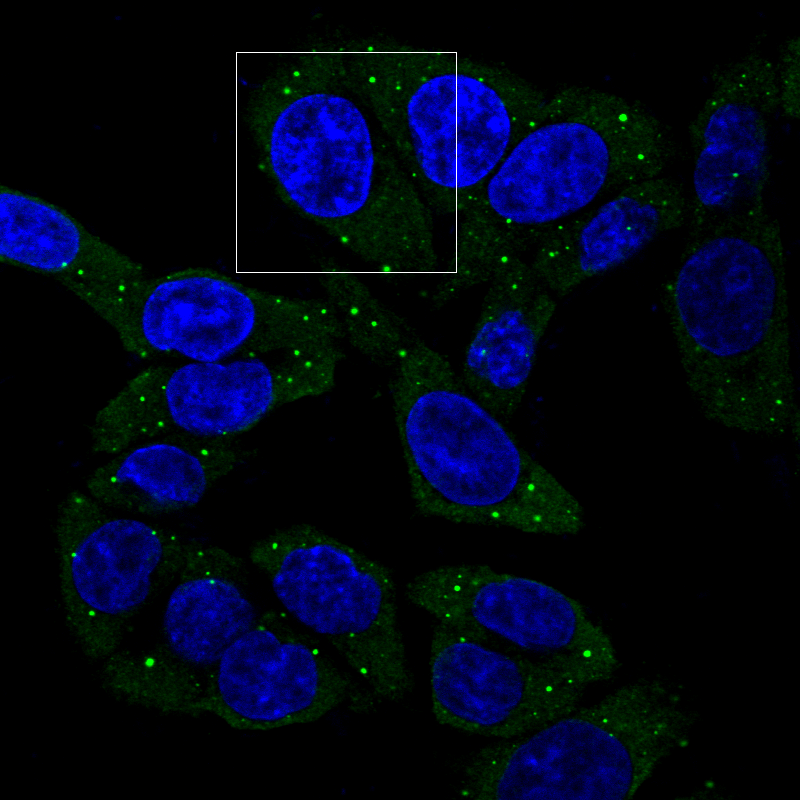

Supplement: Supplementary file 5 — Source data Fig. 2 [file 44318_2024_120_MOESM5_ESM.zip › Figure 2/2I/Ub.tif]

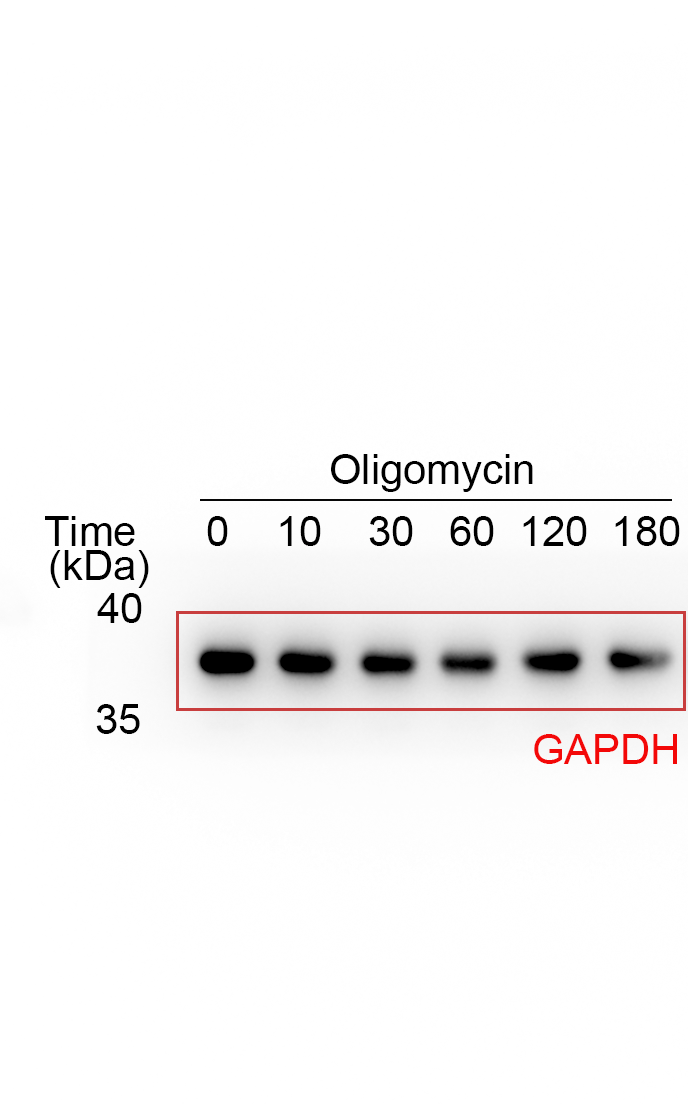

Supplement: Supplementary file 5 — Source data Fig. 2 [file 44318_2024_120_MOESM5_ESM.zip › Figure 2/2A/western-GAPDH.Tif]

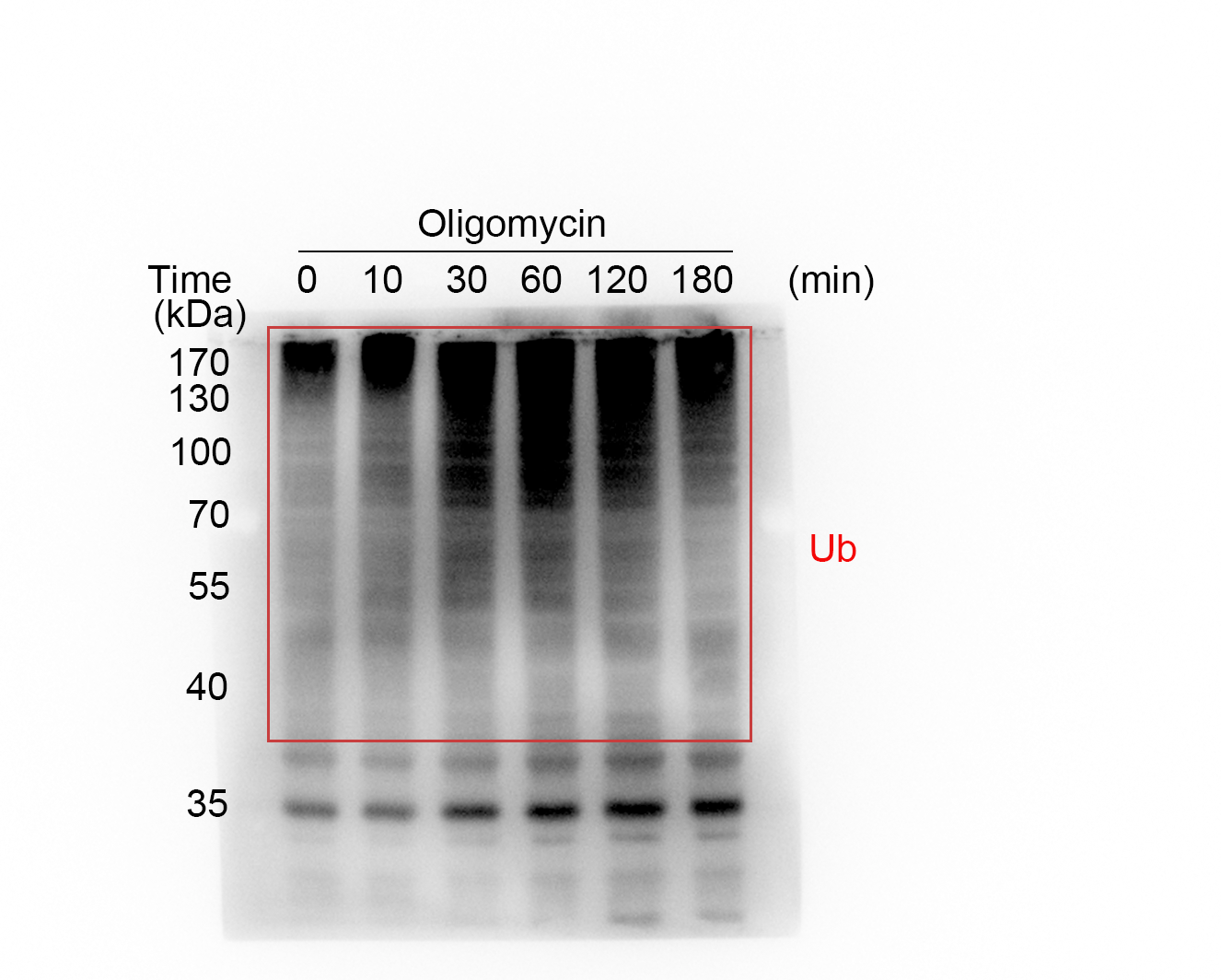

Supplement: Supplementary file 5 — Source data Fig. 2 [file 44318_2024_120_MOESM5_ESM.zip › Figure 2/2A/western-Ub-proteins.Tif]

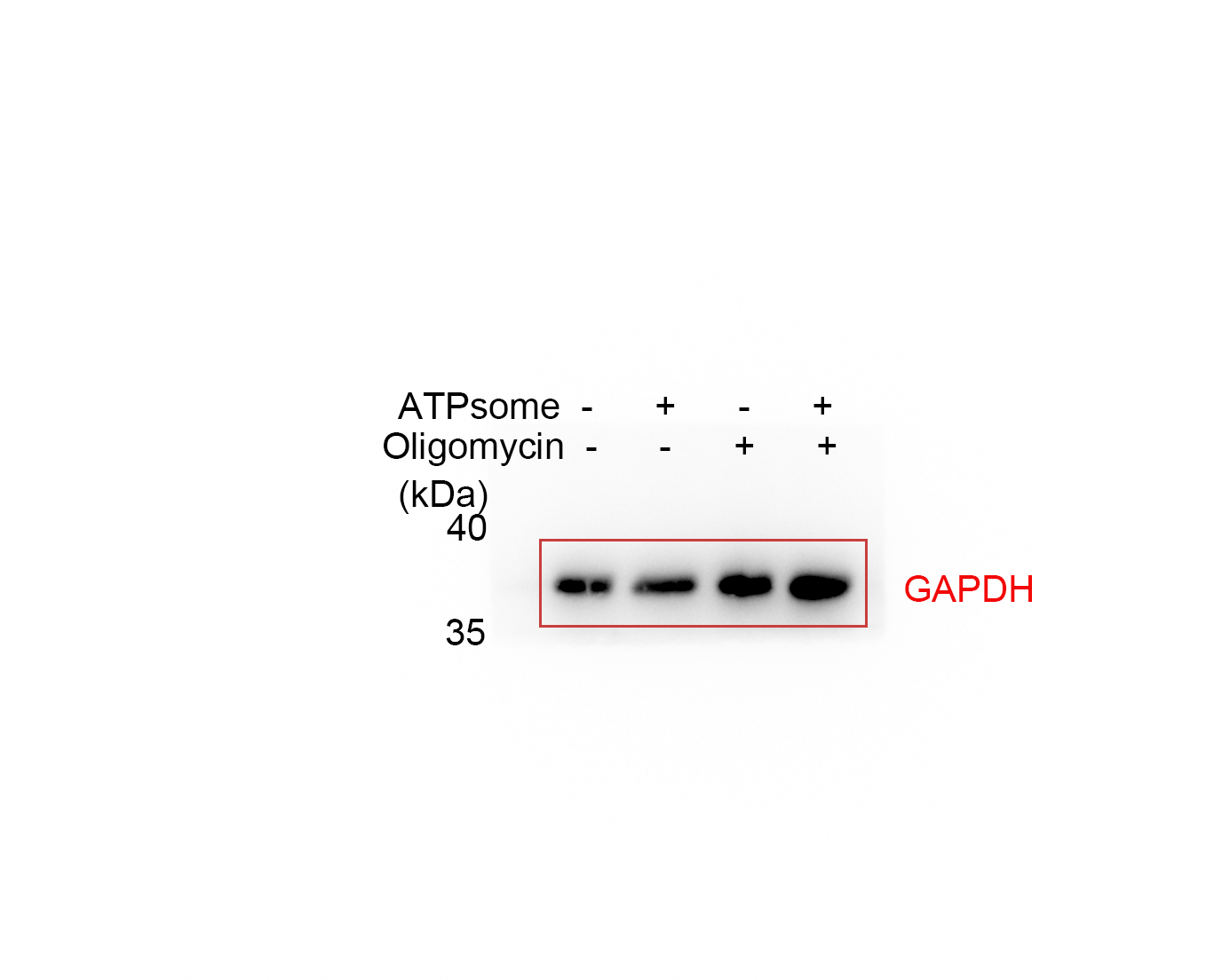

Supplement: Supplementary file 5 — Source data Fig. 2 [file 44318_2024_120_MOESM5_ESM.zip › Figure 2/2B/western-GAPDH.Tif]

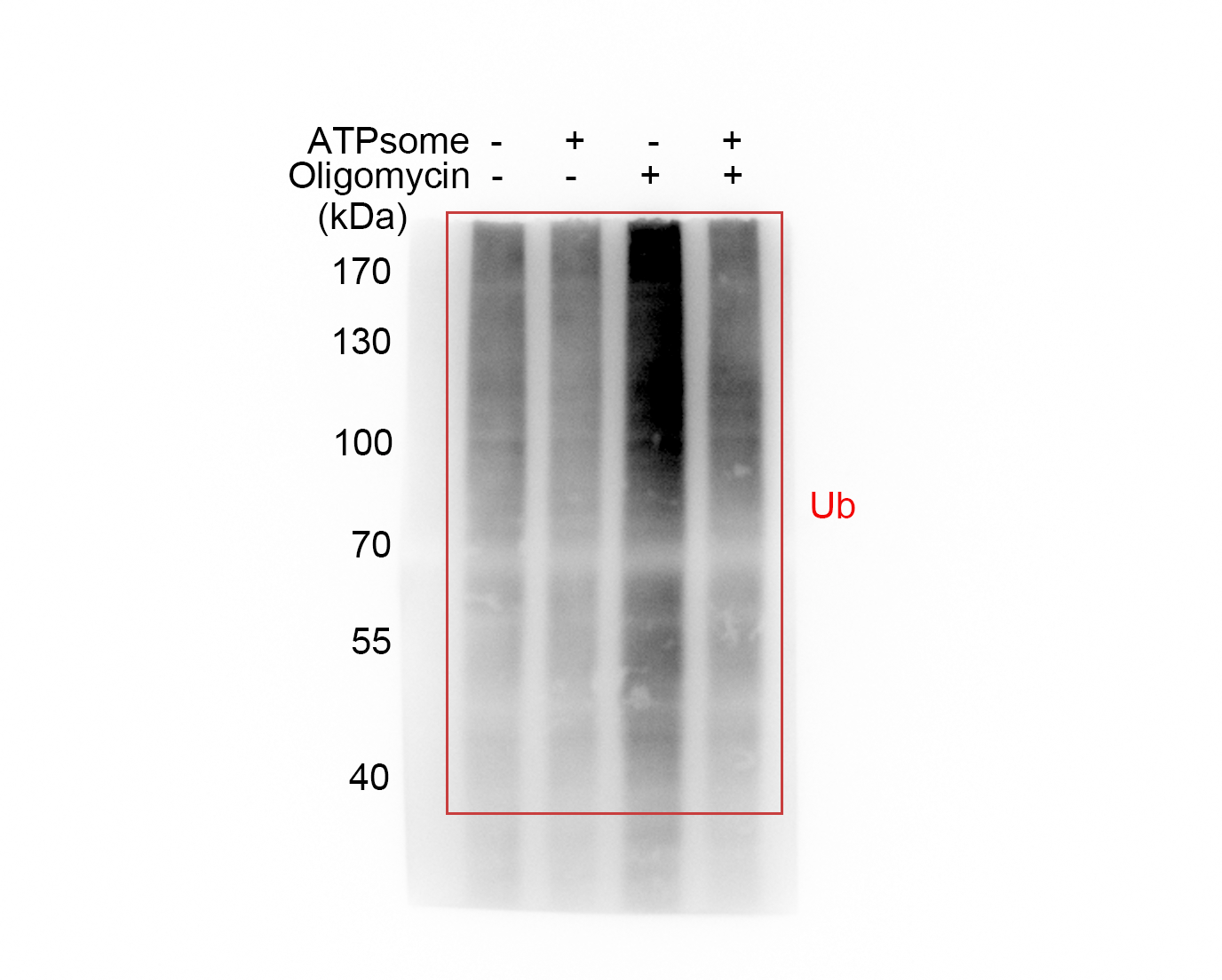

Supplement: Supplementary file 5 — Source data Fig. 2 [file 44318_2024_120_MOESM5_ESM.zip › Figure 2/2B/western-Ub-proteins.Tif]

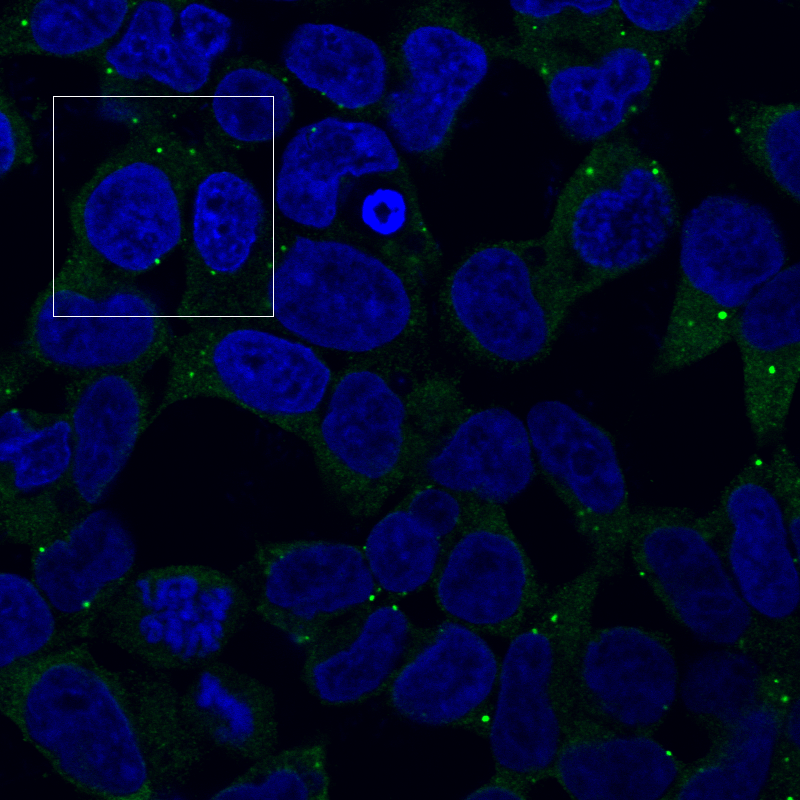

Supplement: Supplementary file 5 — Source data Fig. 2 [file 44318_2024_120_MOESM5_ESM.zip › Figure 2/2G/Mock/shCtrl.tif]

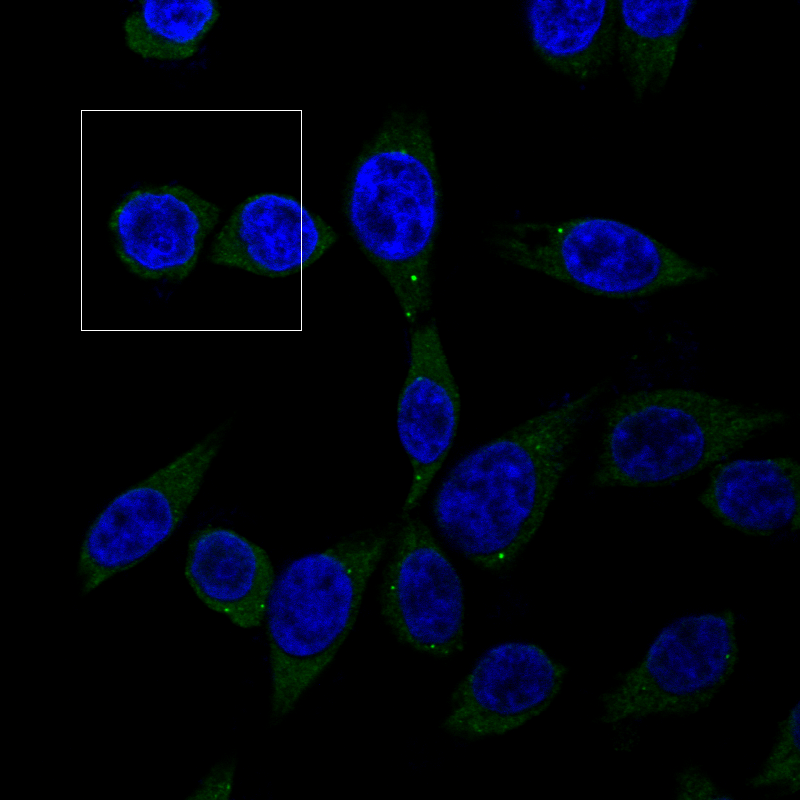

Supplement: Supplementary file 5 — Source data Fig. 2 [file 44318_2024_120_MOESM5_ESM.zip › Figure 2/2G/Mock/shUBB.tif]

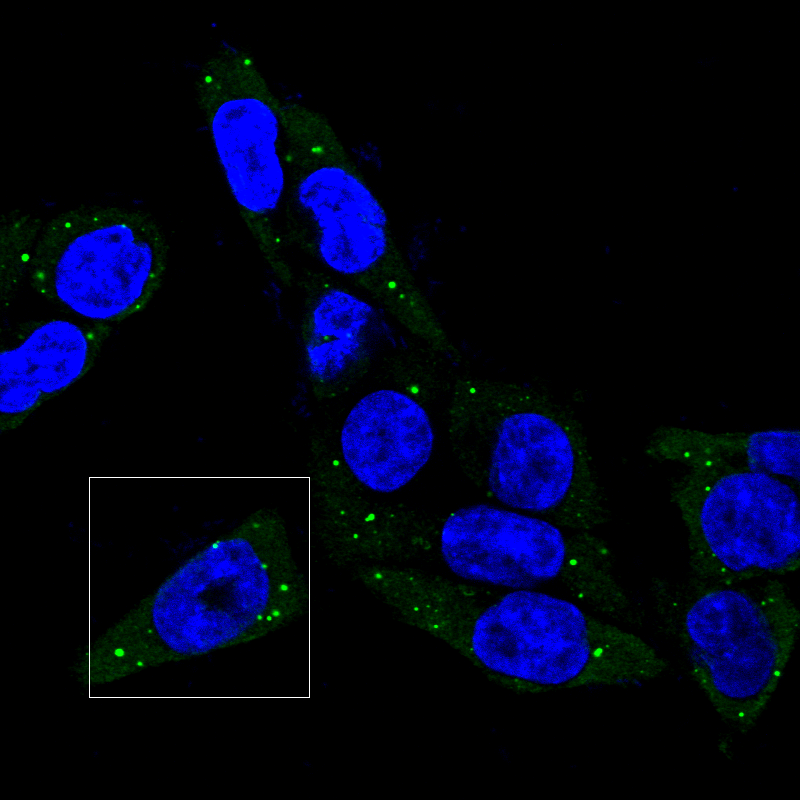

Supplement: Supplementary file 5 — Source data Fig. 2 [file 44318_2024_120_MOESM5_ESM.zip › Figure 2/2G/Oligomycin/shCtrl.tif]

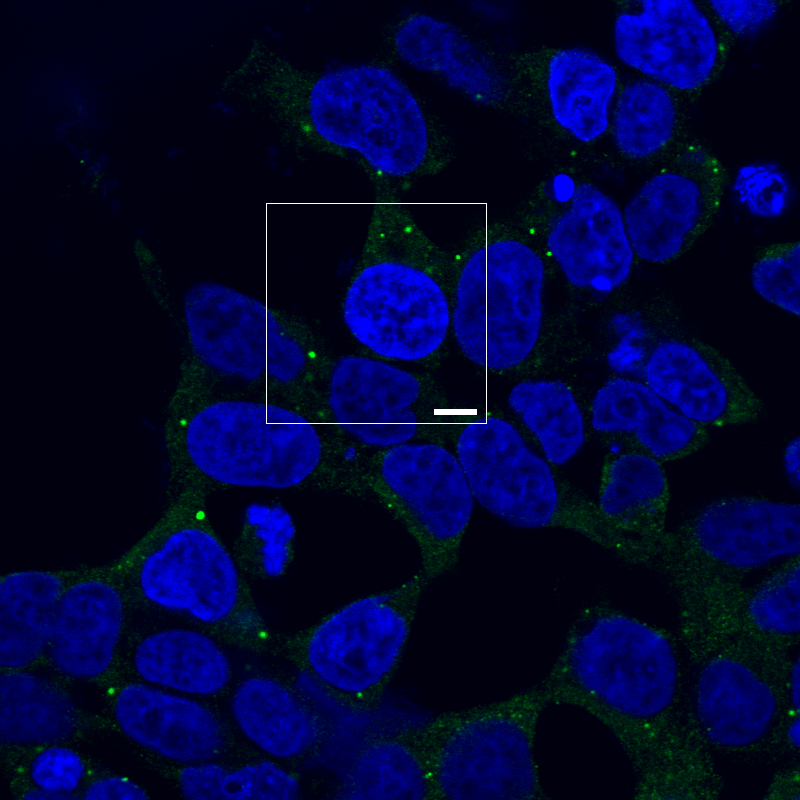

Supplement: Supplementary file 5 — Source data Fig. 2 [file 44318_2024_120_MOESM5_ESM.zip › Figure 2/2G/Oligomycin/shUBB.tif]

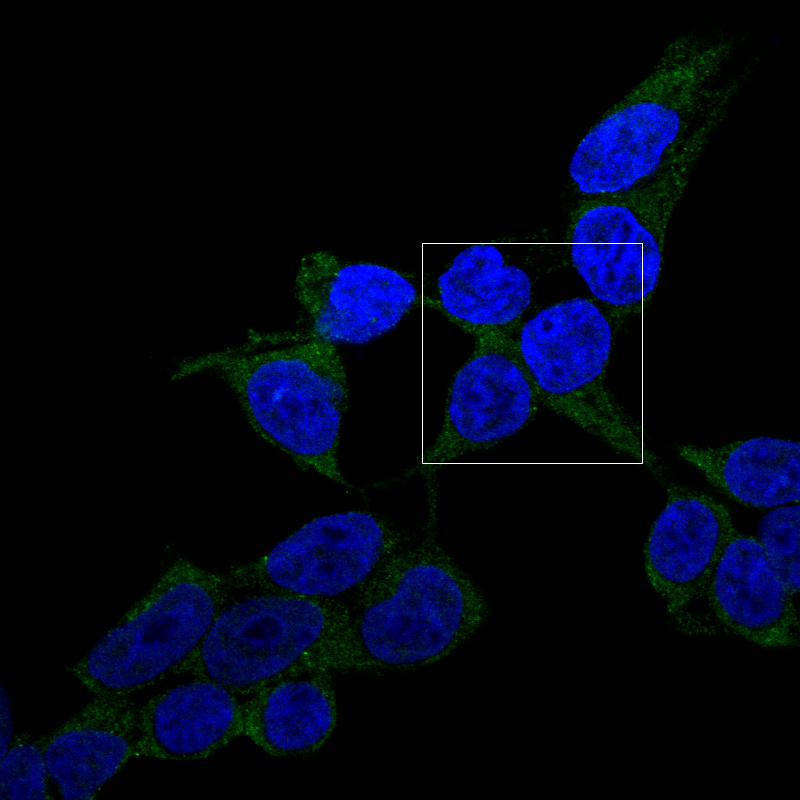

Supplement: Supplementary file 5 — Source data Fig. 2 [file 44318_2024_120_MOESM5_ESM.zip › Figure 2/2C/Mock/TAK-43.tif]

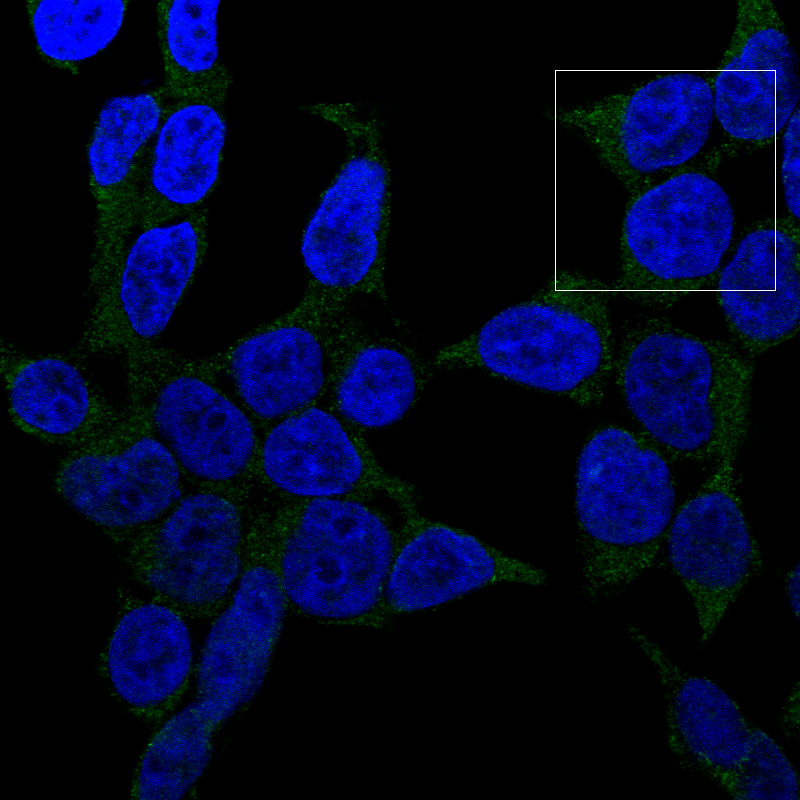

Supplement: Supplementary file 5 — Source data Fig. 2 [file 44318_2024_120_MOESM5_ESM.zip › Figure 2/2C/Mock/PYR-41.tif]

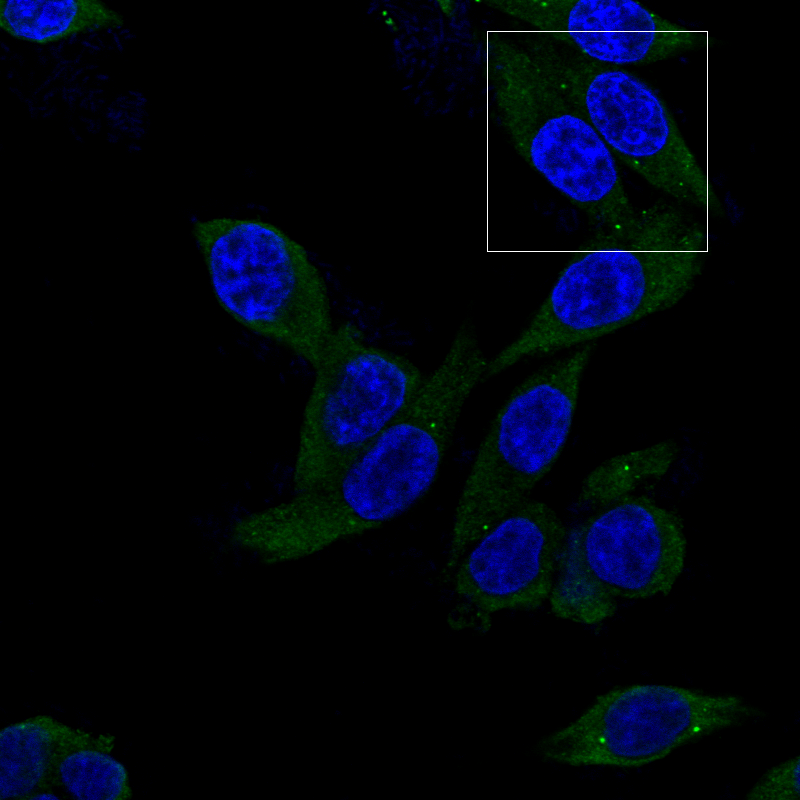

Supplement: Supplementary file 5 — Source data Fig. 2 [file 44318_2024_120_MOESM5_ESM.zip › Figure 2/2C/Mock/Mock.tif]

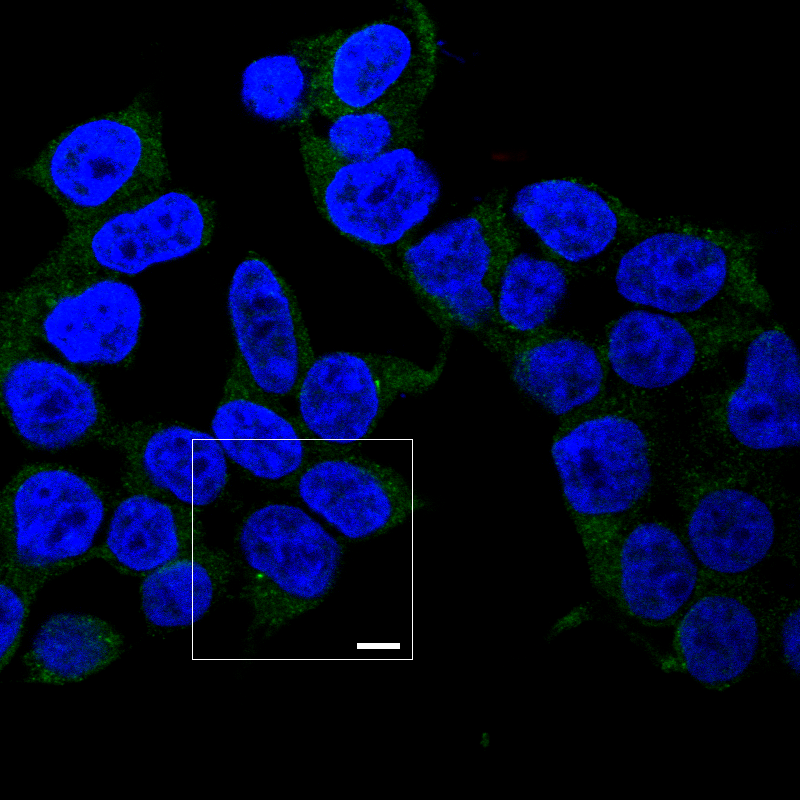

Supplement: Supplementary file 5 — Source data Fig. 2 [file 44318_2024_120_MOESM5_ESM.zip › Figure 2/2C/Oligomycin/PYR-41.tif]

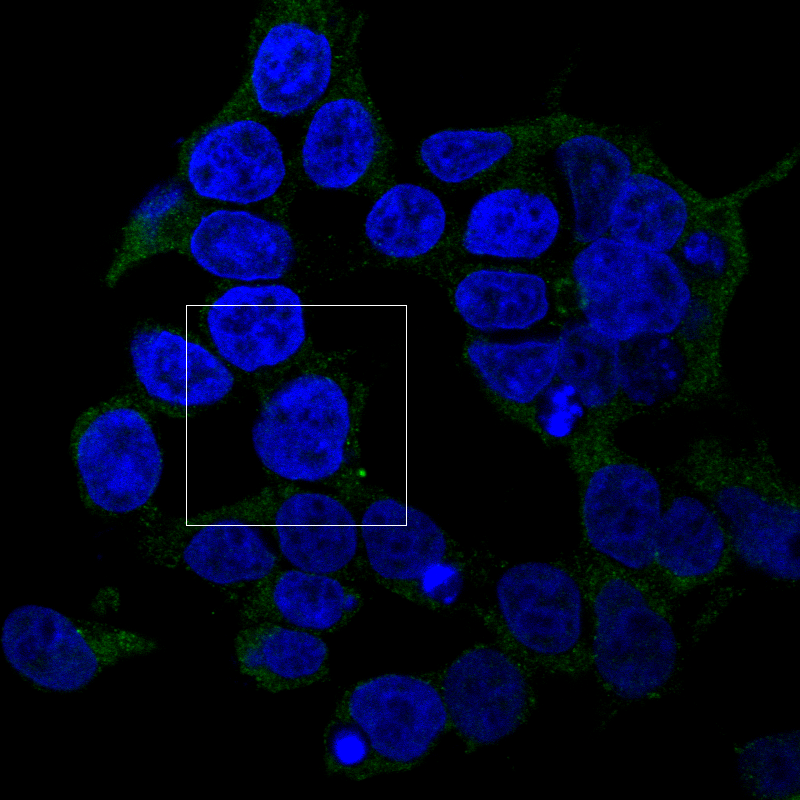

Supplement: Supplementary file 5 — Source data Fig. 2 [file 44318_2024_120_MOESM5_ESM.zip › Figure 2/2C/Oligomycin/TAK-243.tif]

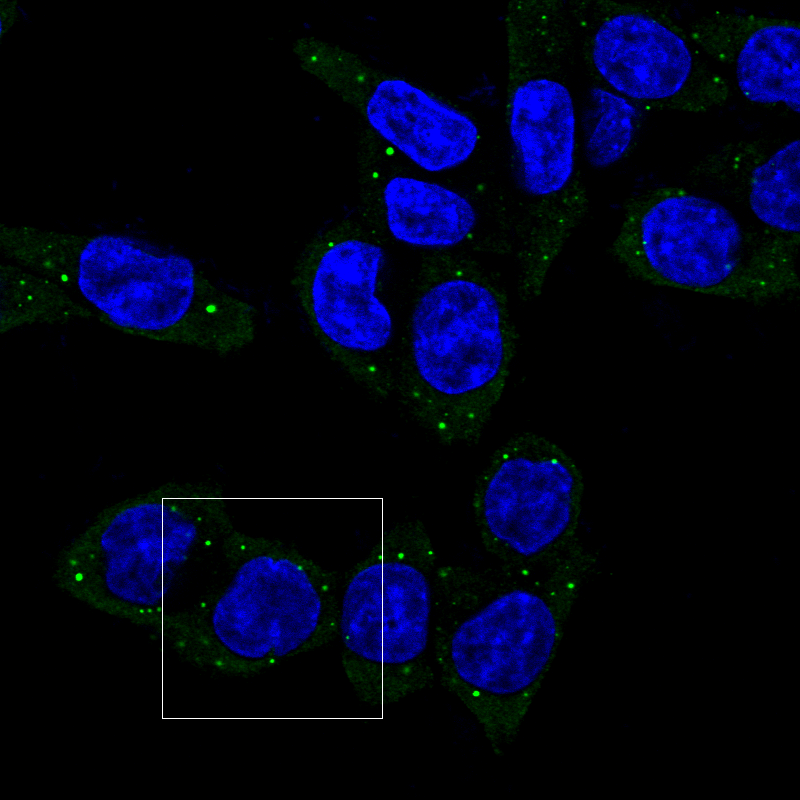

Supplement: Supplementary file 5 — Source data Fig. 2 [file 44318_2024_120_MOESM5_ESM.zip › Figure 2/2C/Oligomycin/Mock.tif]

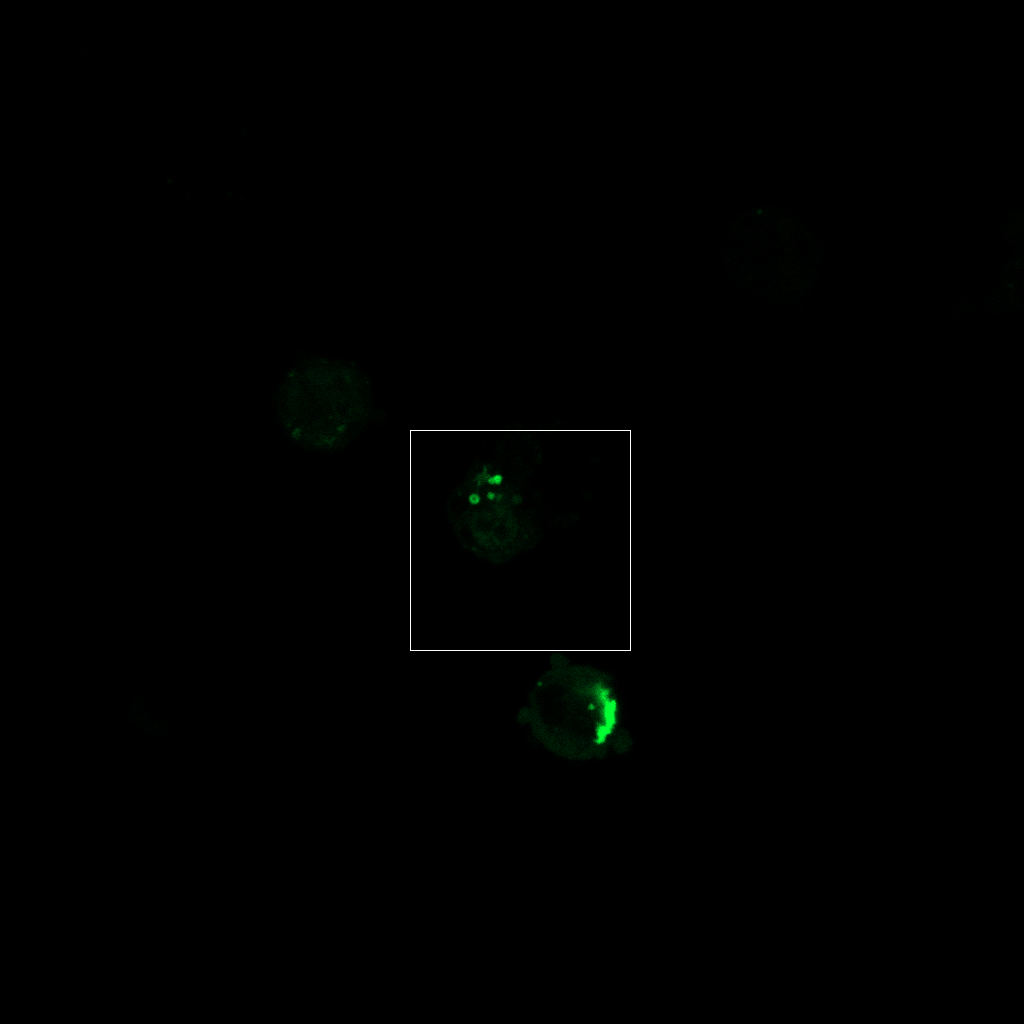

Supplement: Supplementary file 5 — Source data Fig. 2 [file 44318_2024_120_MOESM5_ESM.zip › Figure 2/2M/shUBB/30s.tif]

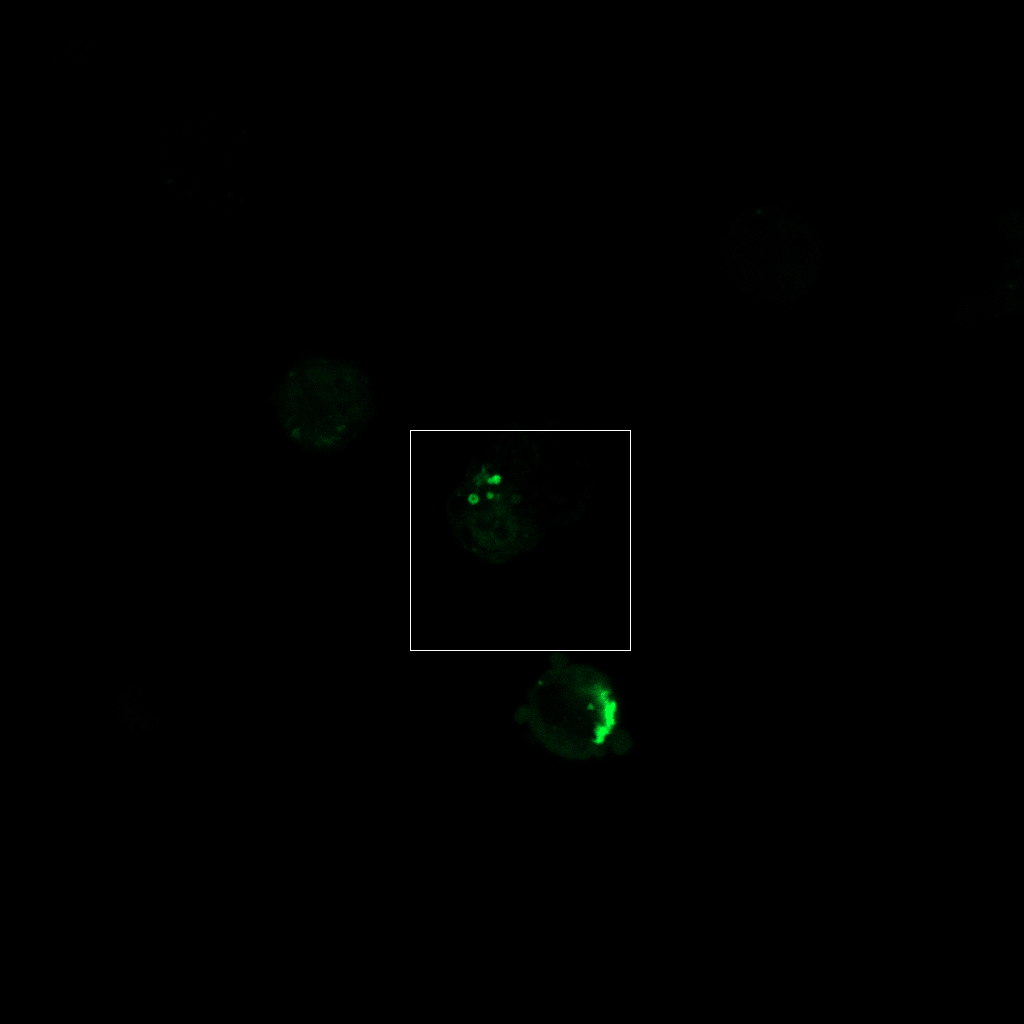

Supplement: Supplementary file 5 — Source data Fig. 2 [file 44318_2024_120_MOESM5_ESM.zip › Figure 2/2M/shUBB/10s.tif]

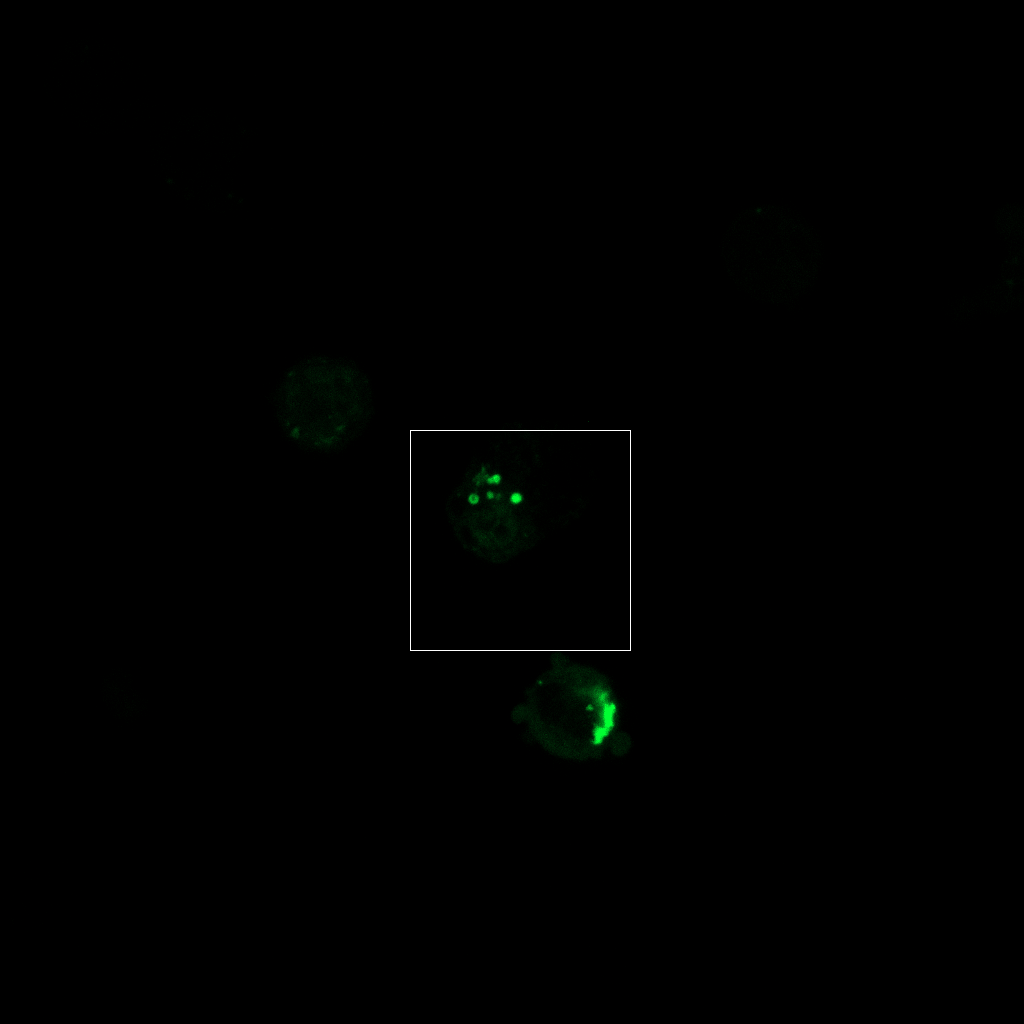

Supplement: Supplementary file 5 — Source data Fig. 2 [file 44318_2024_120_MOESM5_ESM.zip › Figure 2/2M/shUBB/Pre.tif]

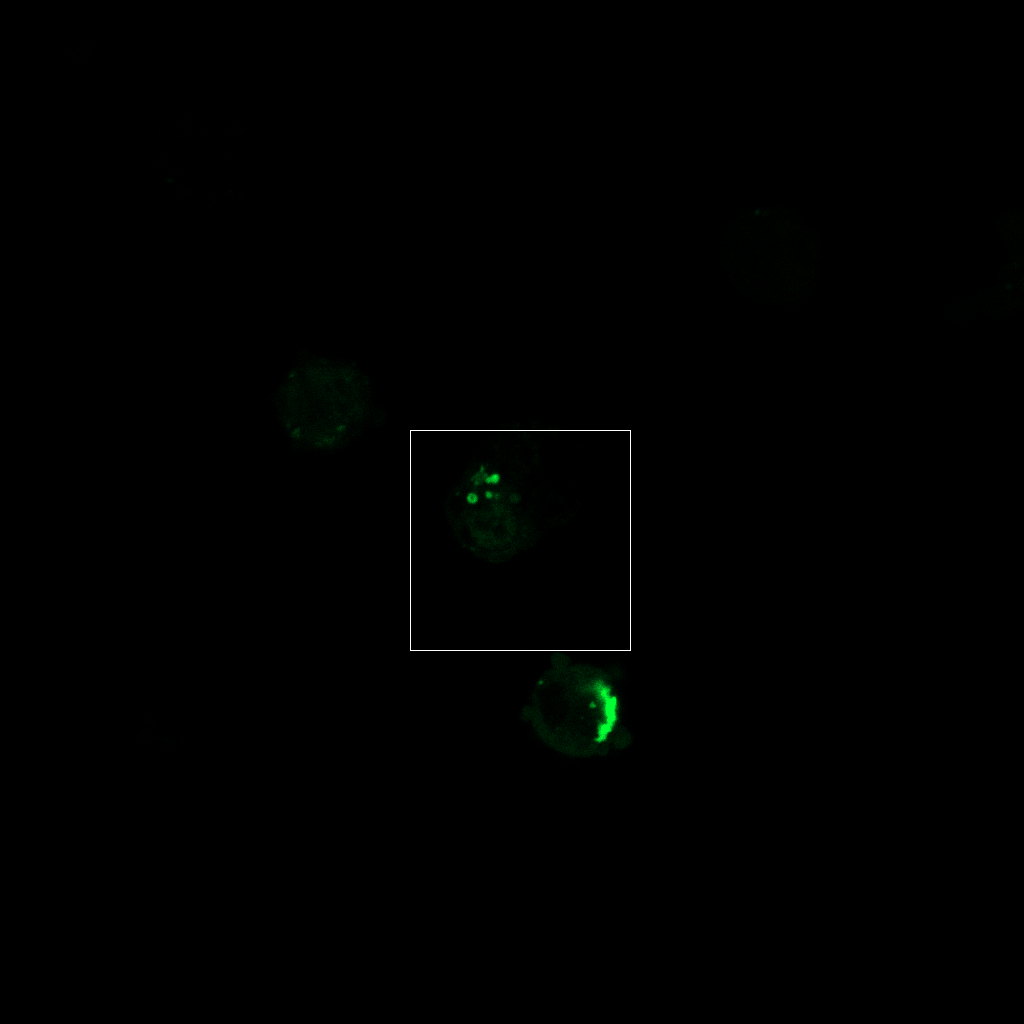

Supplement: Supplementary file 5 — Source data Fig. 2 [file 44318_2024_120_MOESM5_ESM.zip › Figure 2/2M/shUBB/60s.tif]

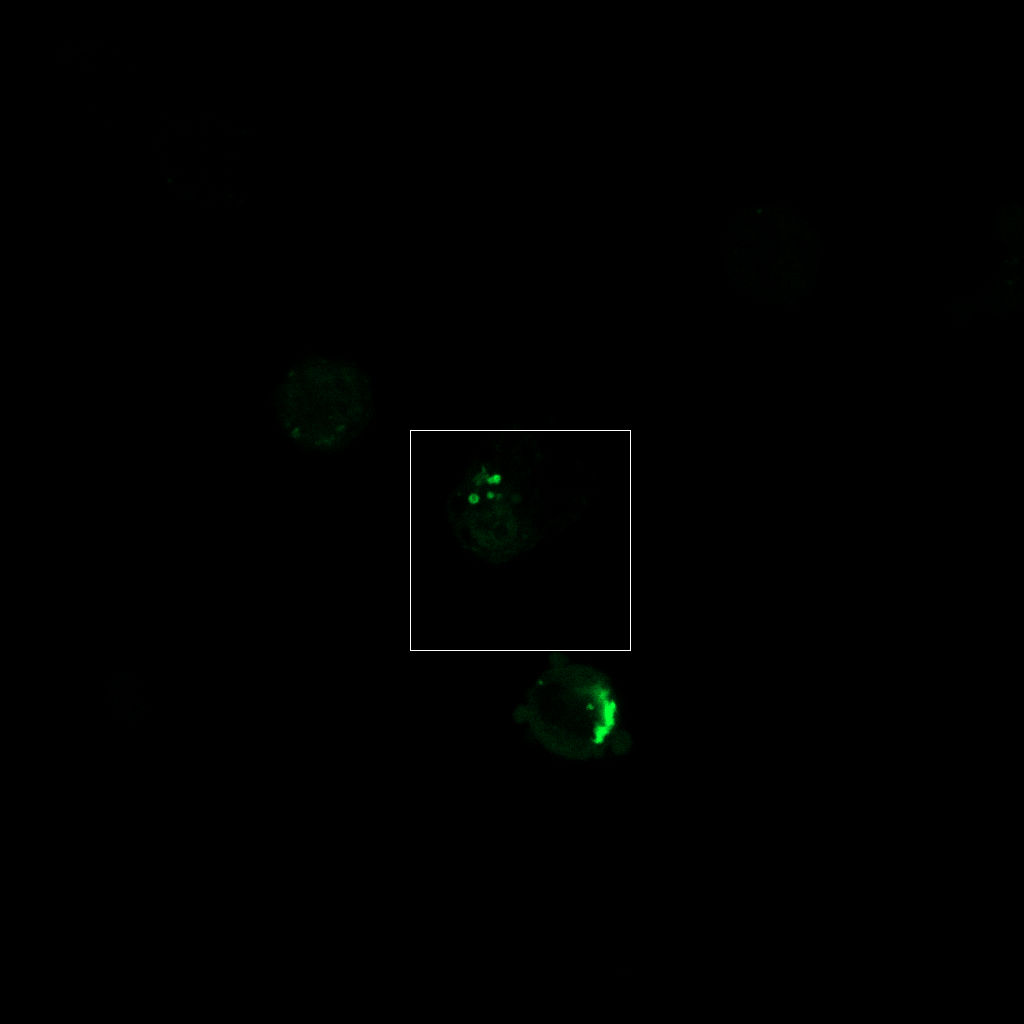

Supplement: Supplementary file 5 — Source data Fig. 2 [file 44318_2024_120_MOESM5_ESM.zip › Figure 2/2M/shUBB/Bleaching.tif]

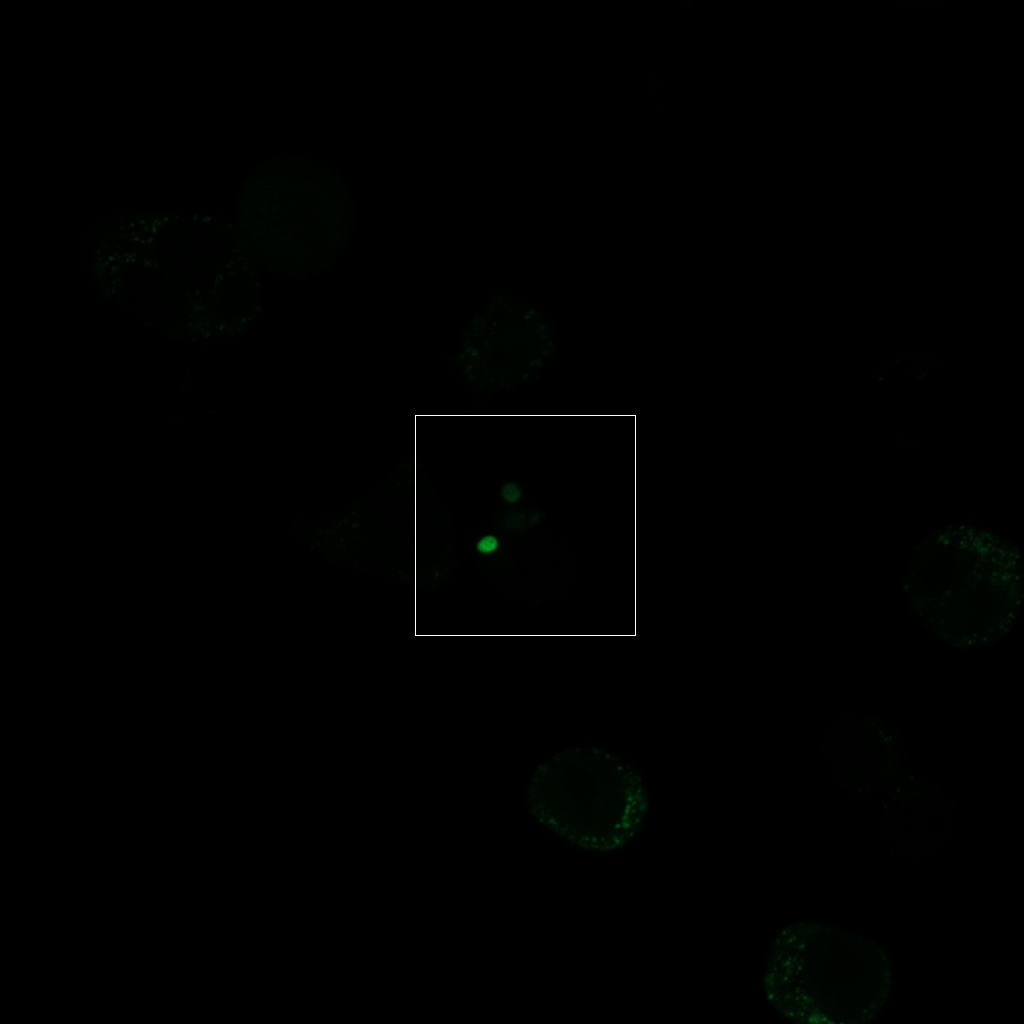

Supplement: Supplementary file 5 — Source data Fig. 2 [file 44318_2024_120_MOESM5_ESM.zip › Figure 2/2M/shCtrl/30s.tif]

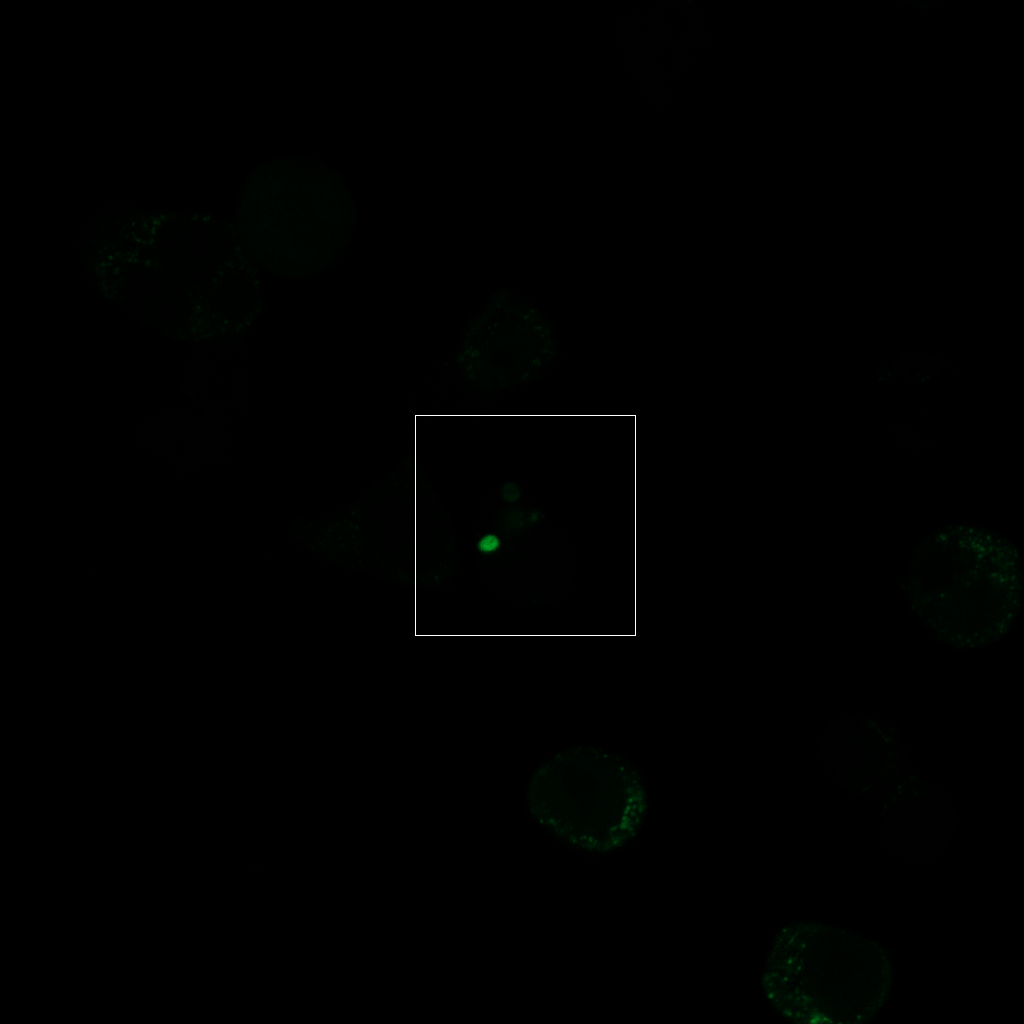

Supplement: Supplementary file 5 — Source data Fig. 2 [file 44318_2024_120_MOESM5_ESM.zip › Figure 2/2M/shCtrl/10s.tif]

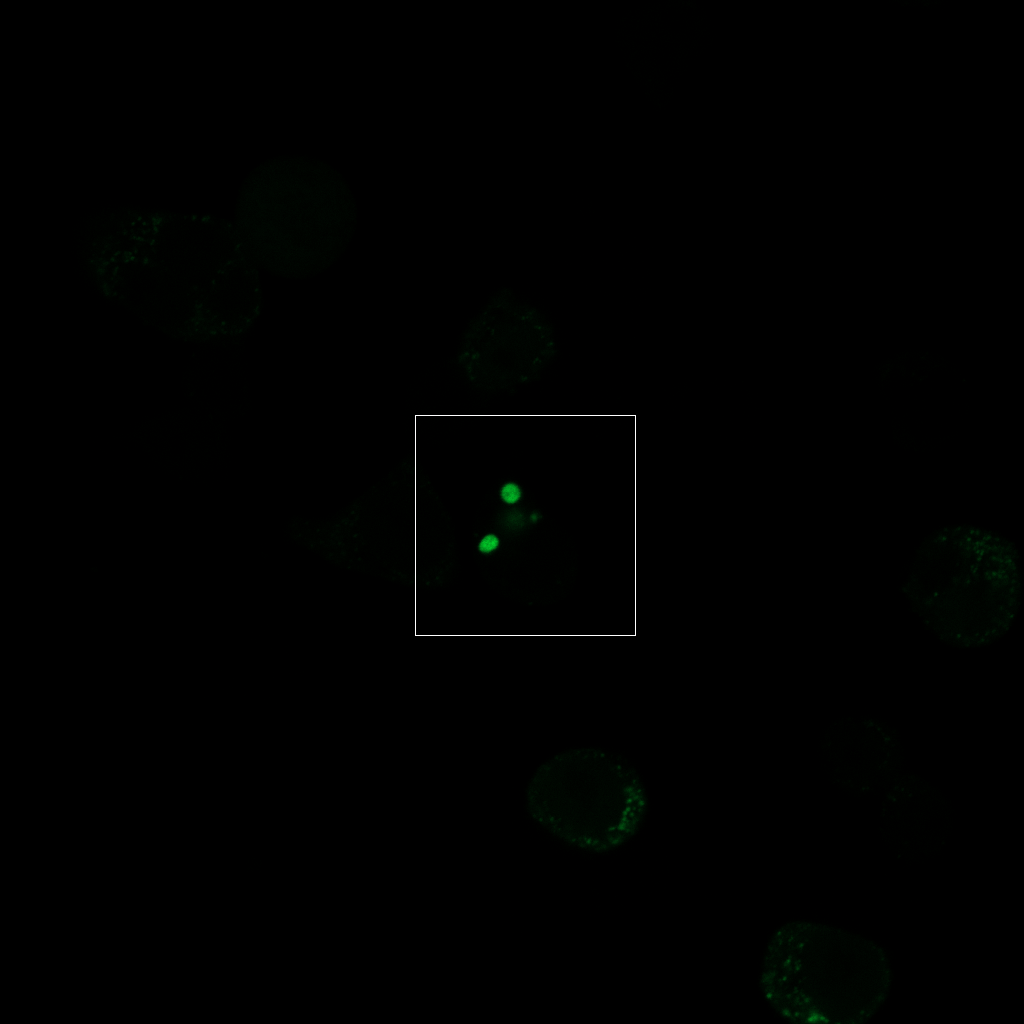

Supplement: Supplementary file 5 — Source data Fig. 2 [file 44318_2024_120_MOESM5_ESM.zip › Figure 2/2M/shCtrl/Pre.tif]

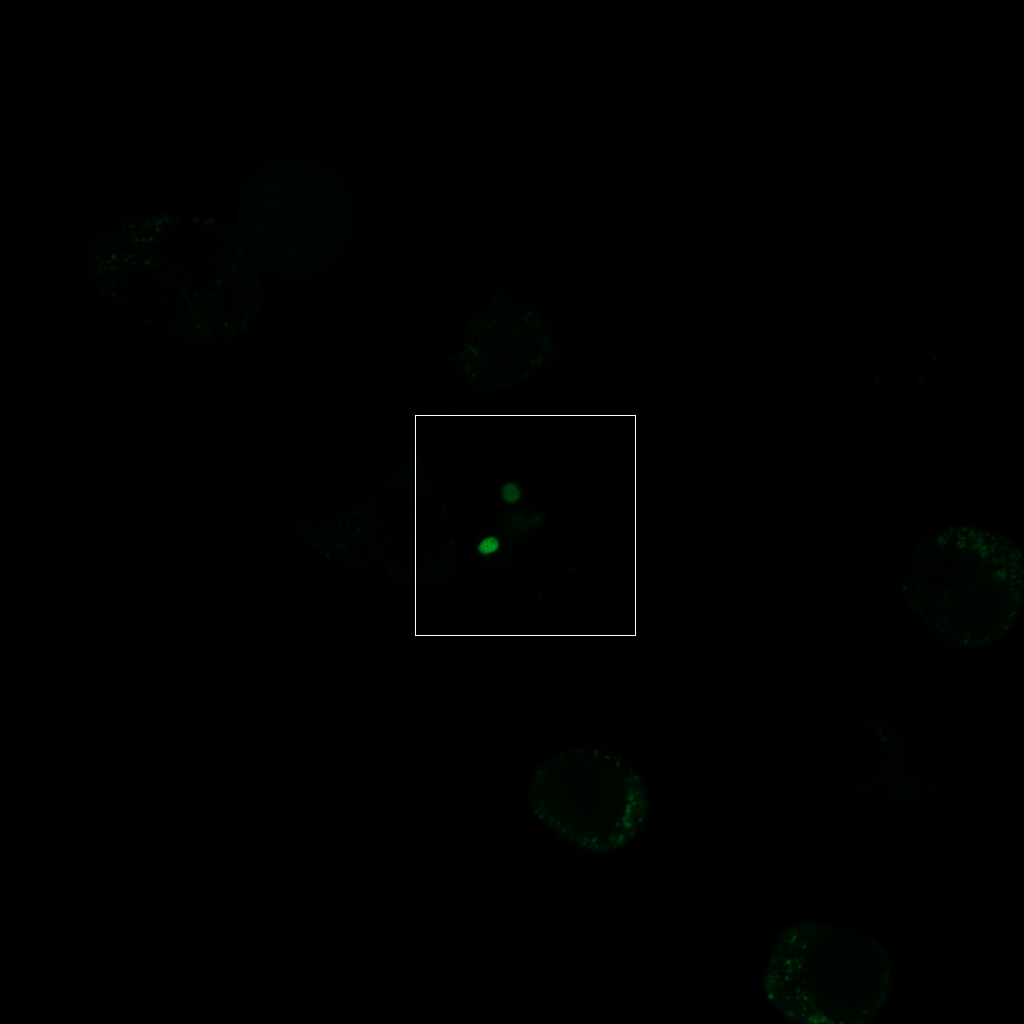

Supplement: Supplementary file 5 — Source data Fig. 2 [file 44318_2024_120_MOESM5_ESM.zip › Figure 2/2M/shCtrl/60s.tif]

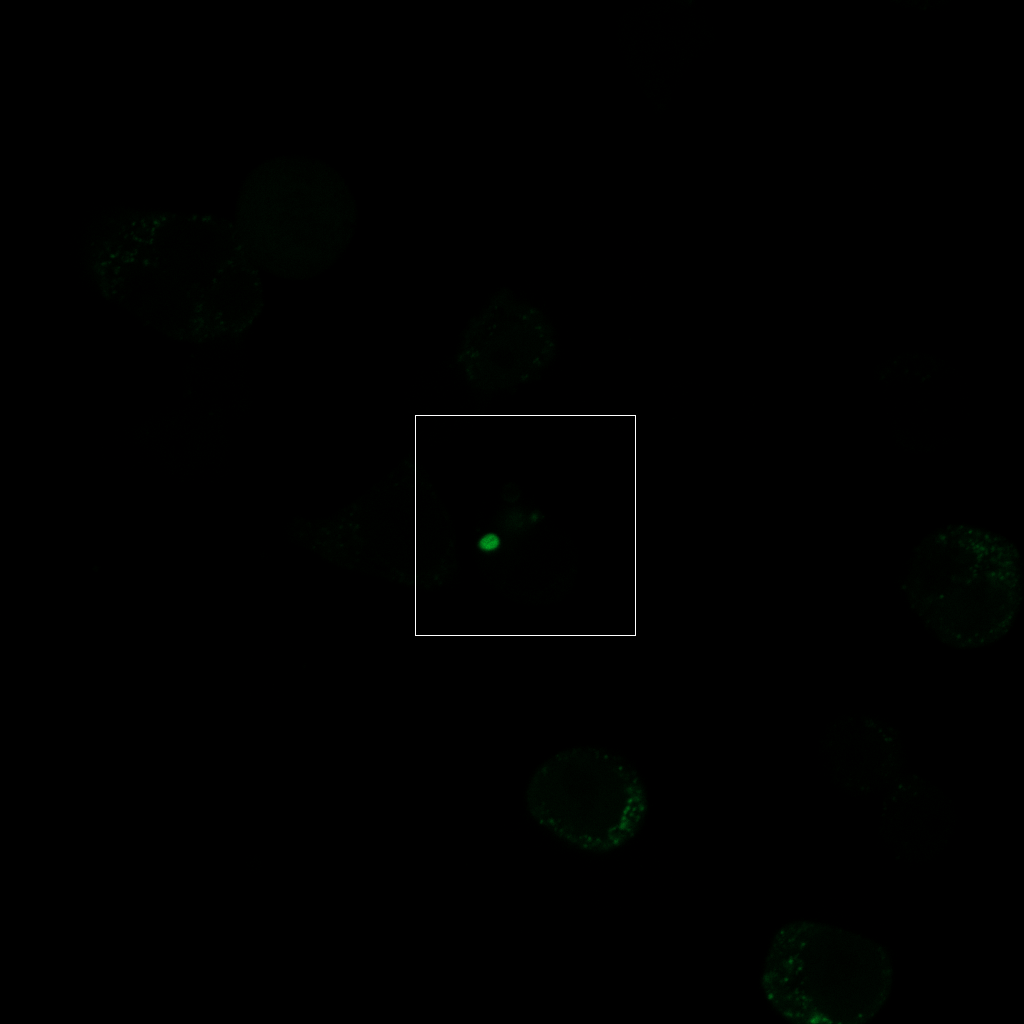

Supplement: Supplementary file 5 — Source data Fig. 2 [file 44318_2024_120_MOESM5_ESM.zip › Figure 2/2M/shCtrl/Bleaching.tif]

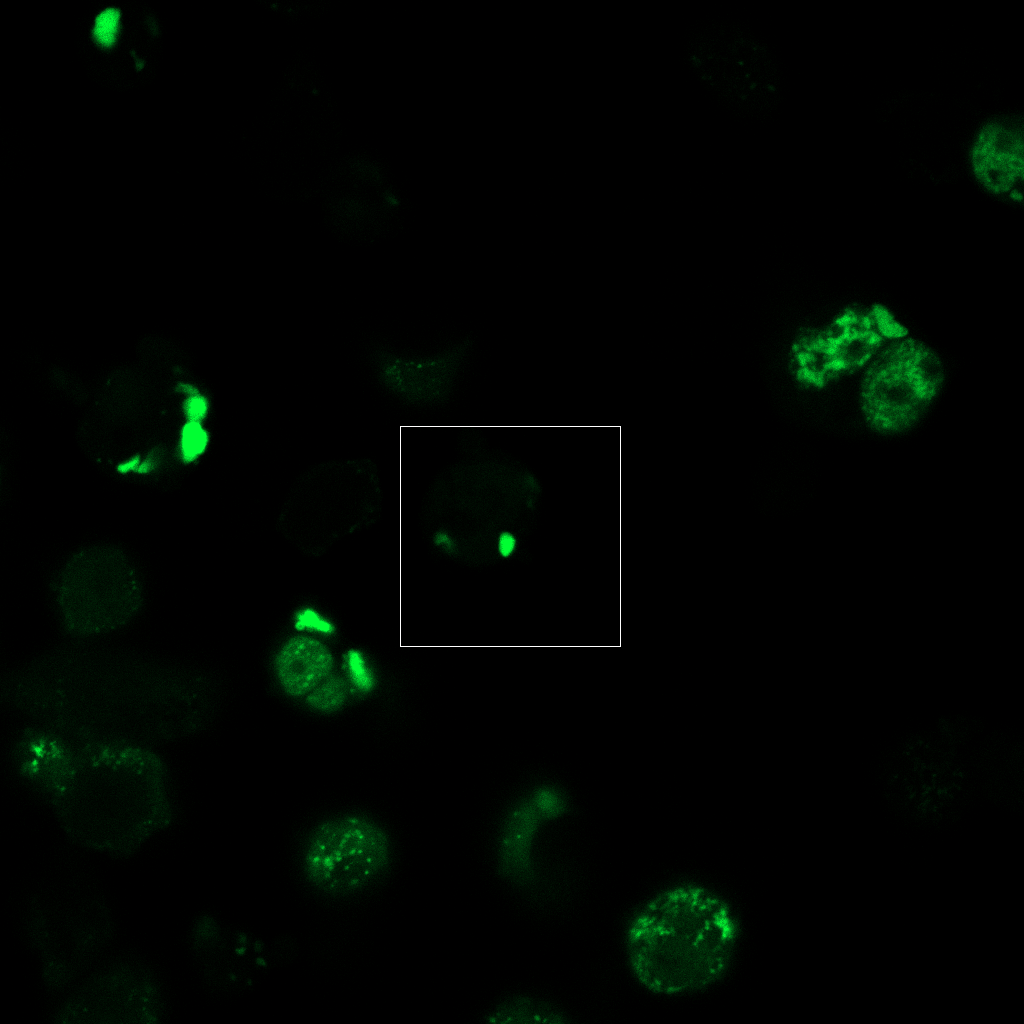

Supplement: Supplementary file 5 — Source data Fig. 2 [file 44318_2024_120_MOESM5_ESM.zip › Figure 2/2K/TAK-243/Bleachinng.tif]

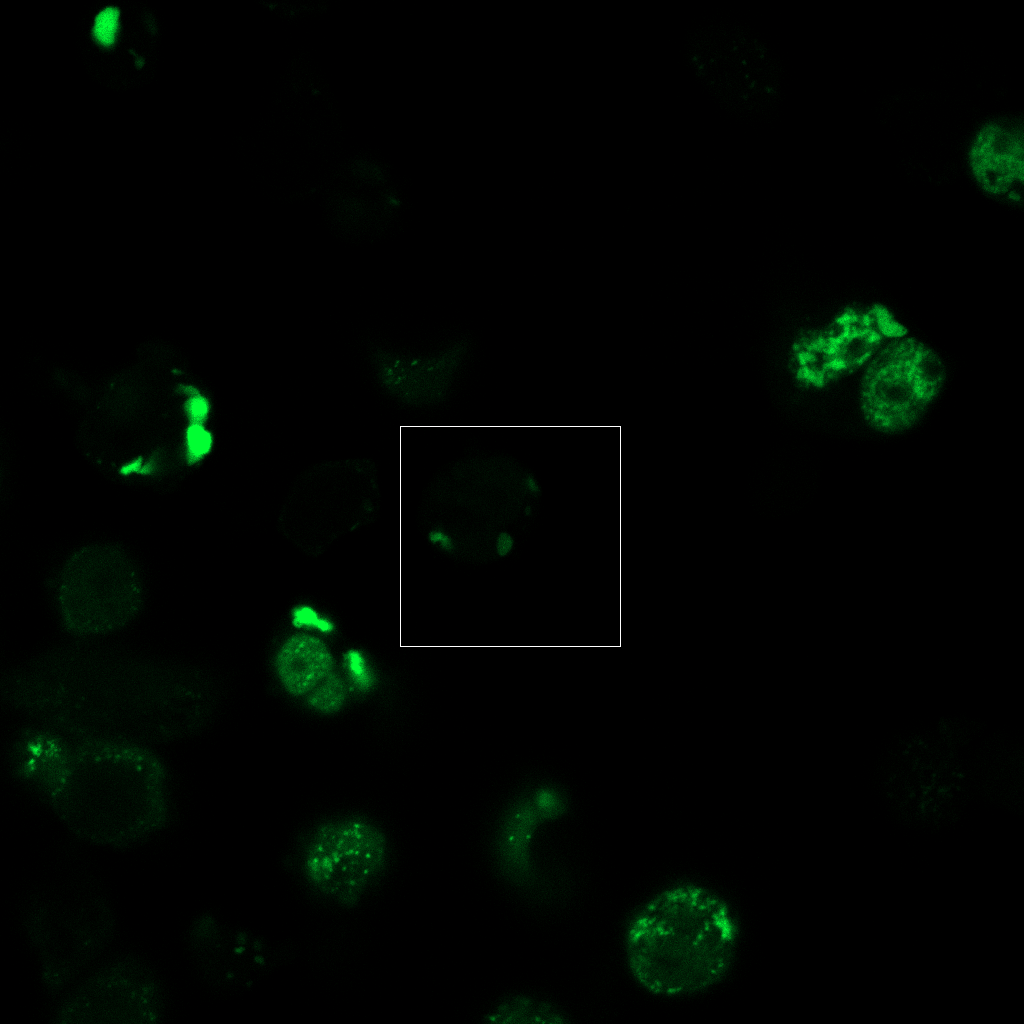

Supplement: Supplementary file 5 — Source data Fig. 2 [file 44318_2024_120_MOESM5_ESM.zip › Figure 2/2K/TAK-243/30s.tif]

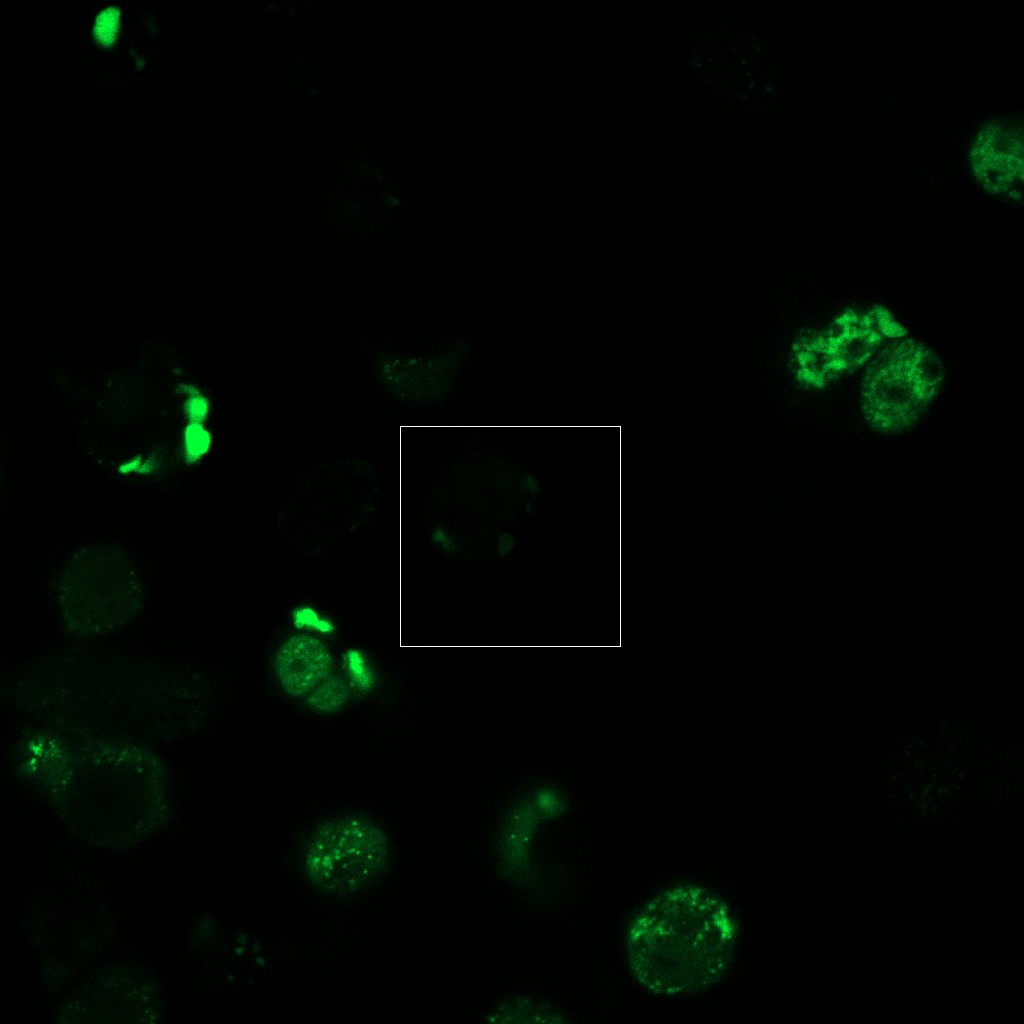

Supplement: Supplementary file 5 — Source data Fig. 2 [file 44318_2024_120_MOESM5_ESM.zip › Figure 2/2K/TAK-243/10s.tif]

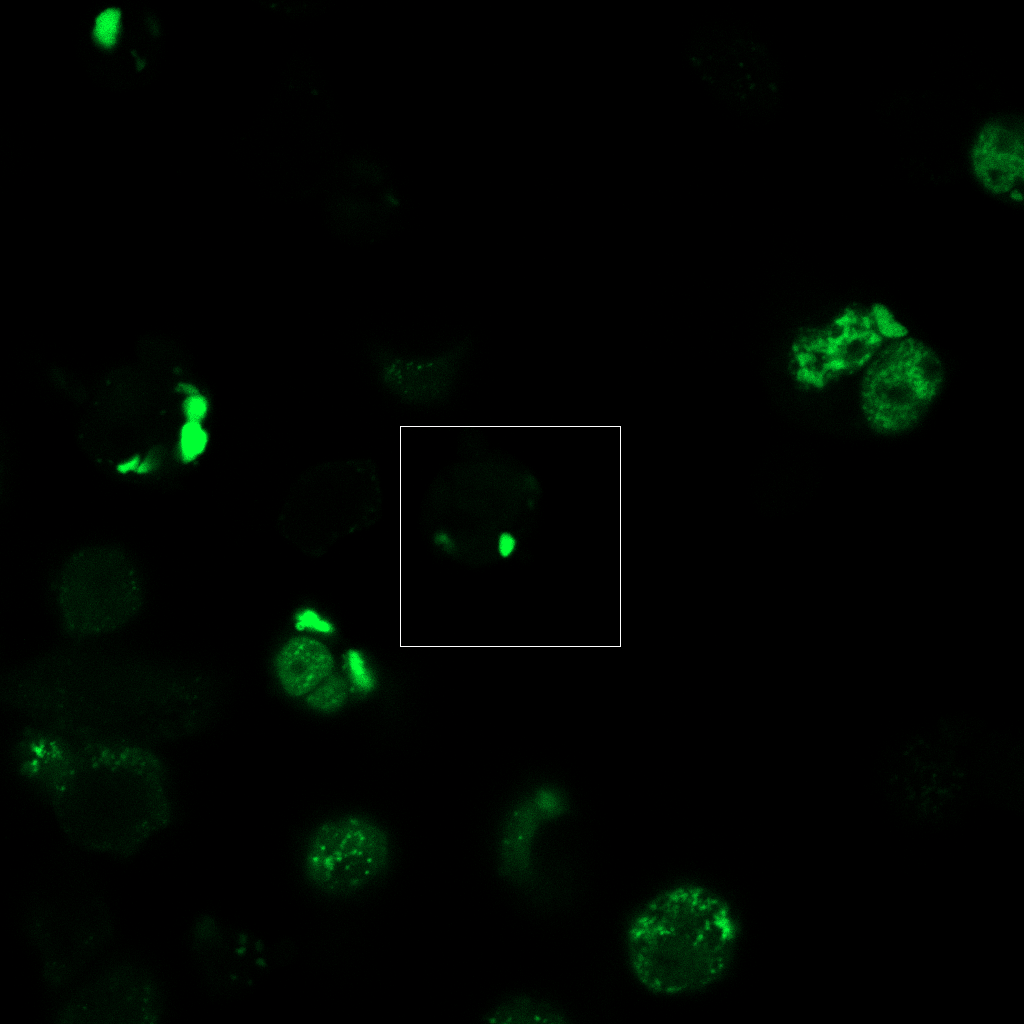

Supplement: Supplementary file 5 — Source data Fig. 2 [file 44318_2024_120_MOESM5_ESM.zip › Figure 2/2K/TAK-243/Pre.tif]

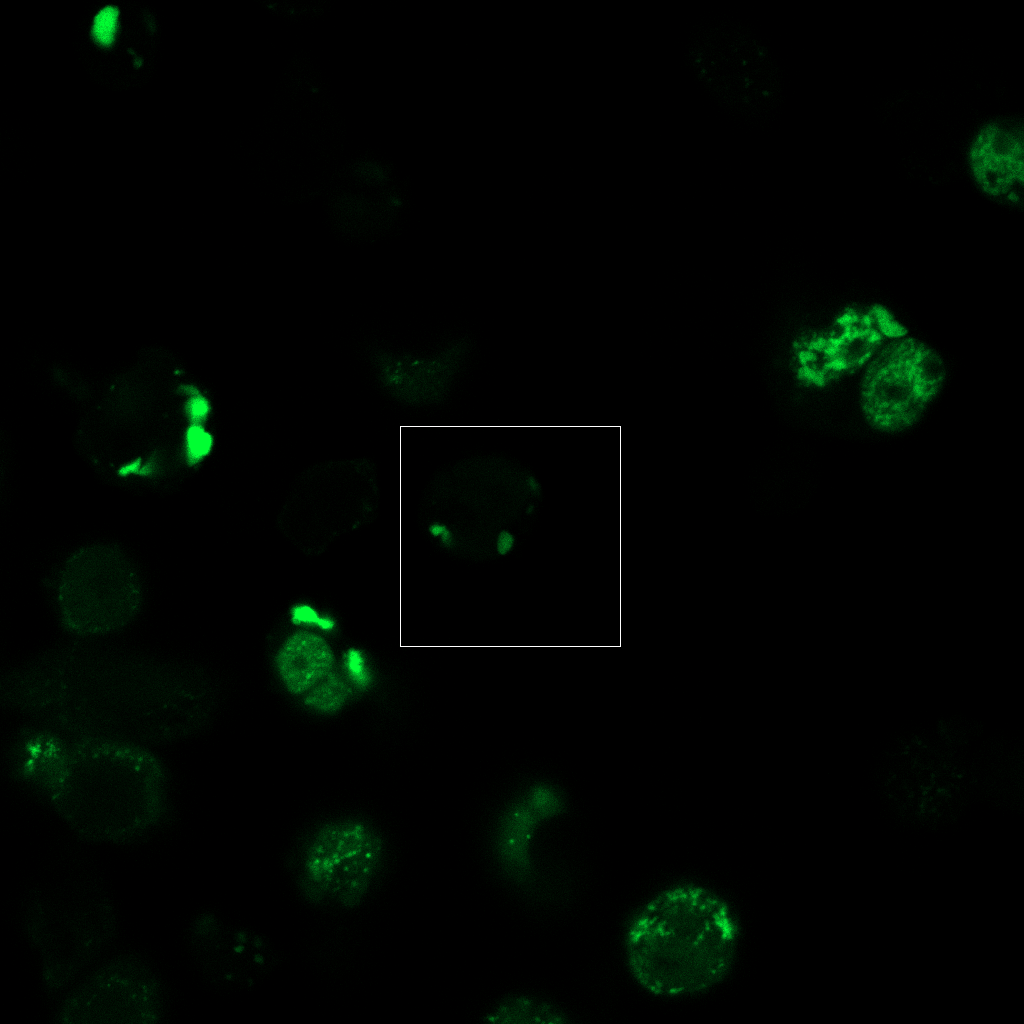

Supplement: Supplementary file 5 — Source data Fig. 2 [file 44318_2024_120_MOESM5_ESM.zip › Figure 2/2K/TAK-243/60s.tif]

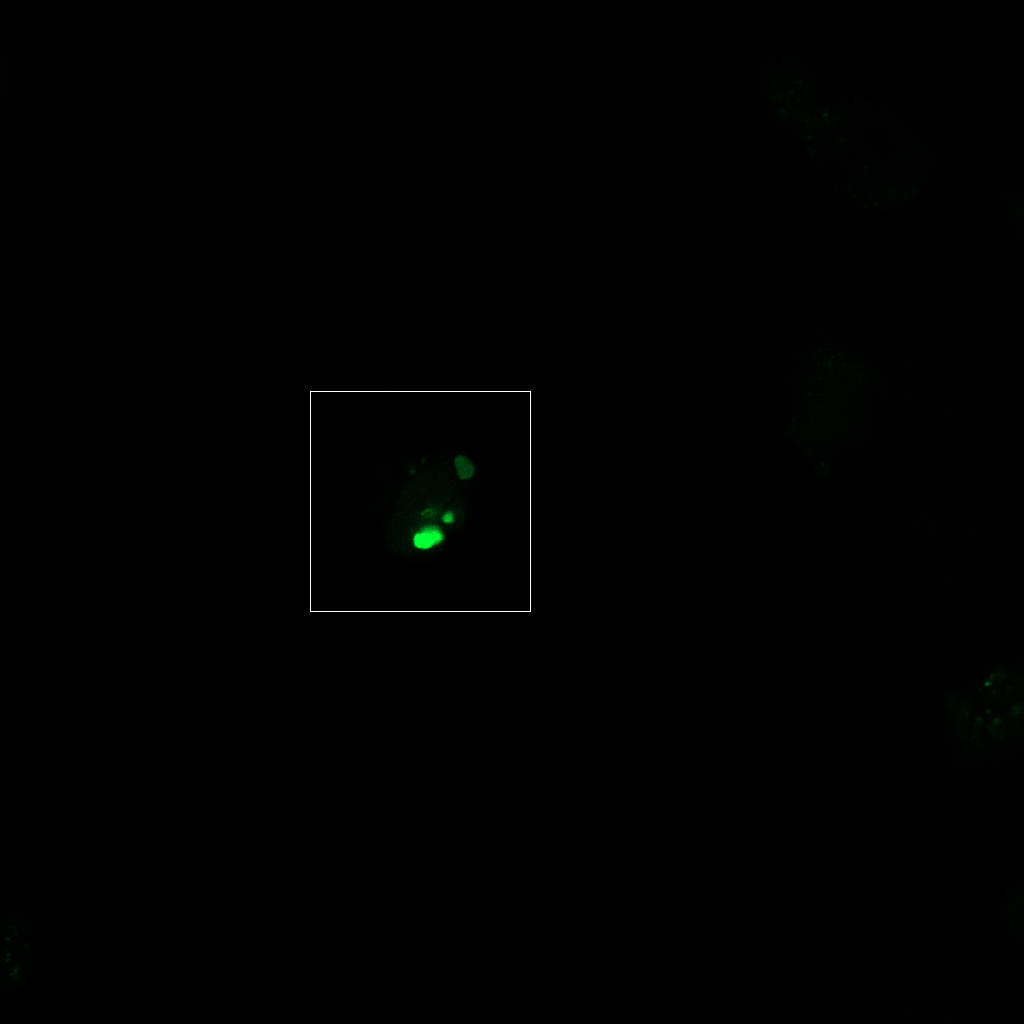

Supplement: Supplementary file 5 — Source data Fig. 2 [file 44318_2024_120_MOESM5_ESM.zip › Figure 2/2K/Mock/30s.tif]

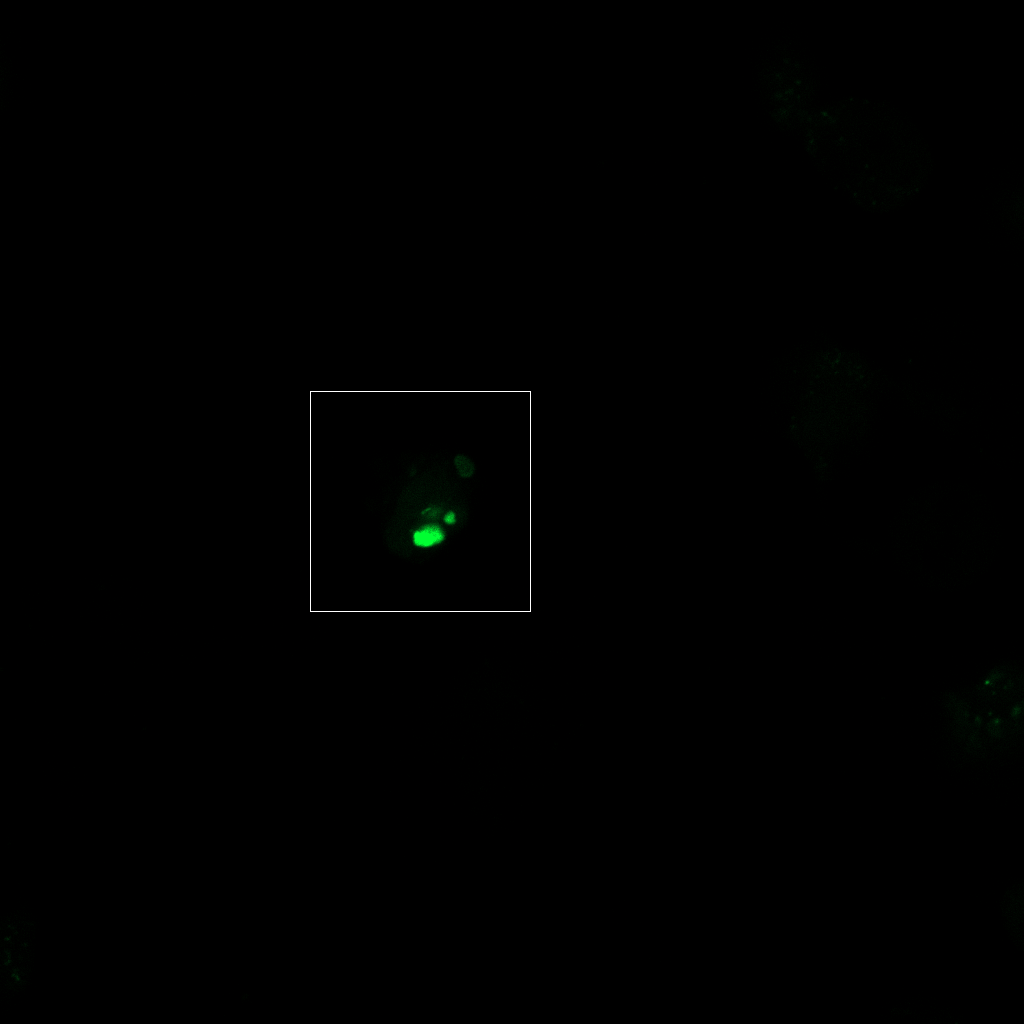

Supplement: Supplementary file 5 — Source data Fig. 2 [file 44318_2024_120_MOESM5_ESM.zip › Figure 2/2K/Mock/10s.tif]

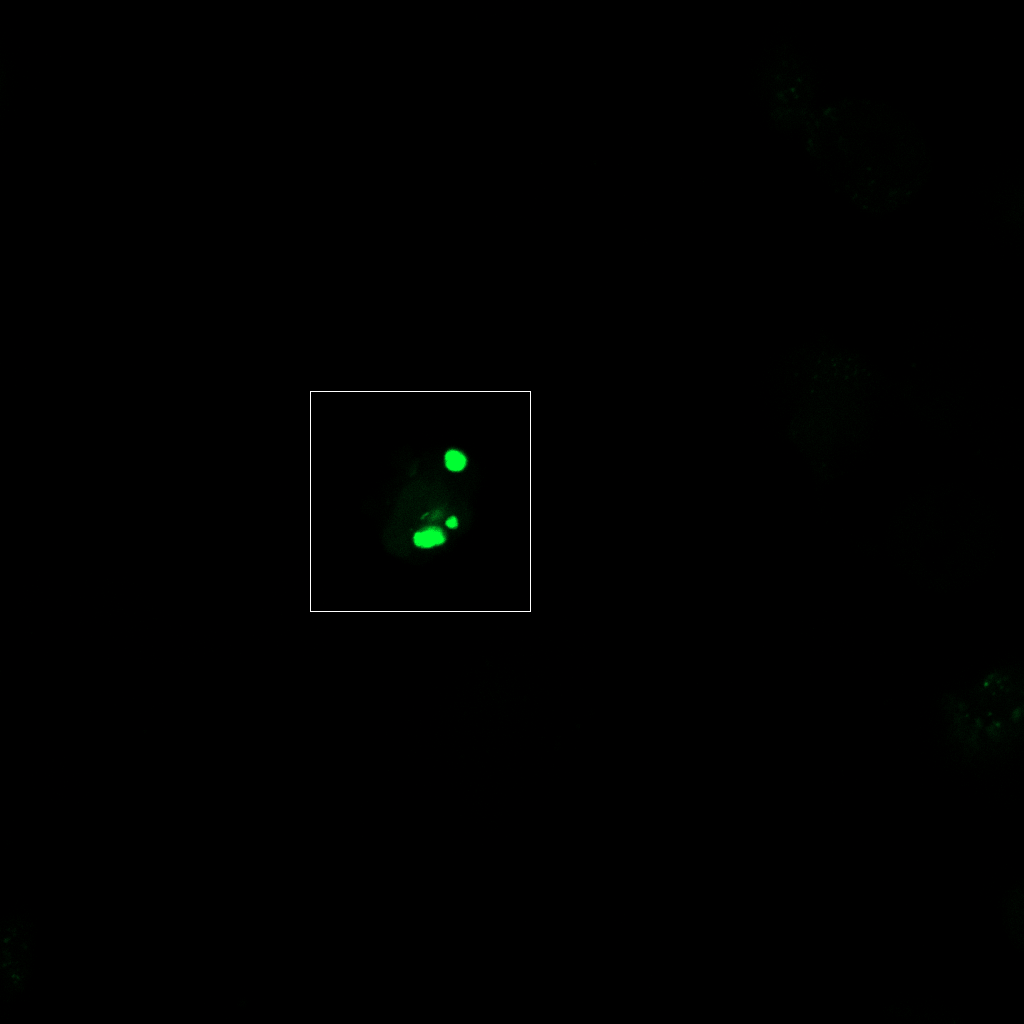

Supplement: Supplementary file 5 — Source data Fig. 2 [file 44318_2024_120_MOESM5_ESM.zip › Figure 2/2K/Mock/Pre.tif]

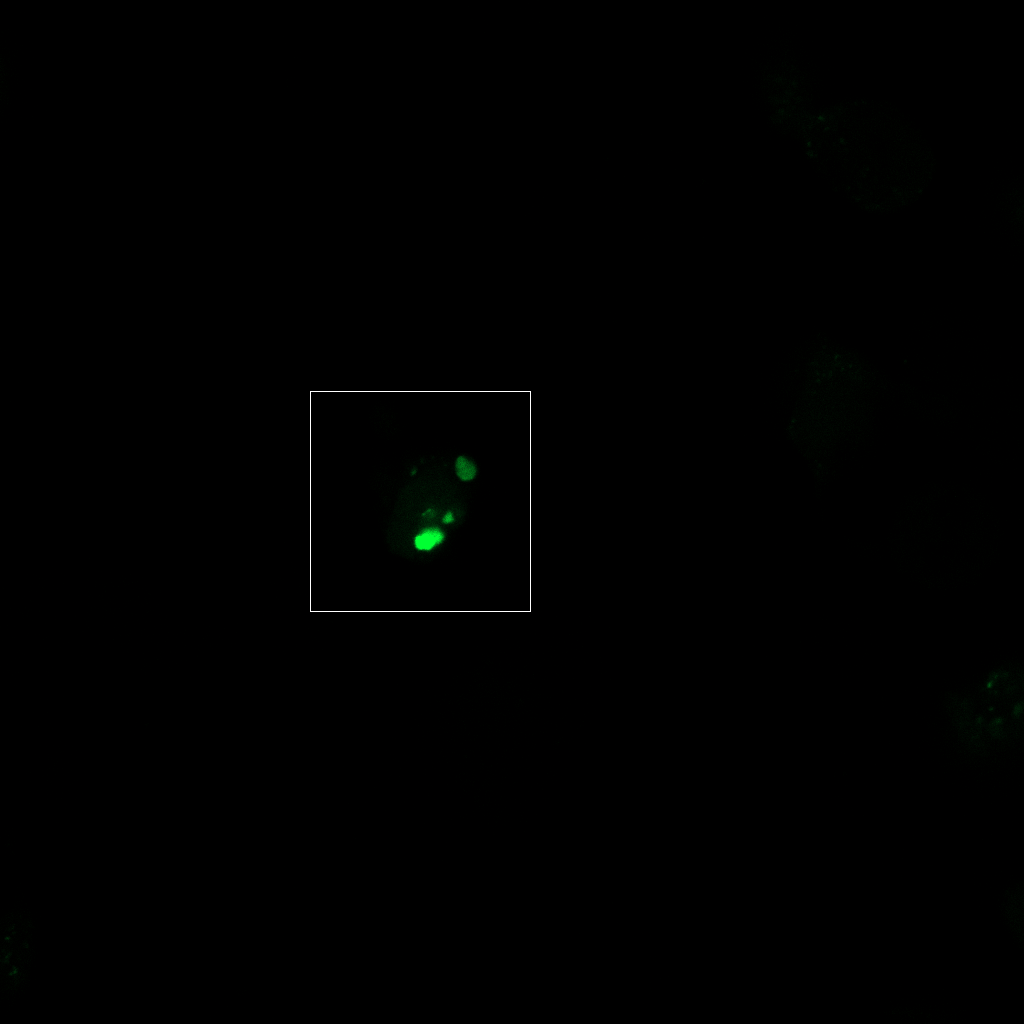

Supplement: Supplementary file 5 — Source data Fig. 2 [file 44318_2024_120_MOESM5_ESM.zip › Figure 2/2K/Mock/60s.tif]

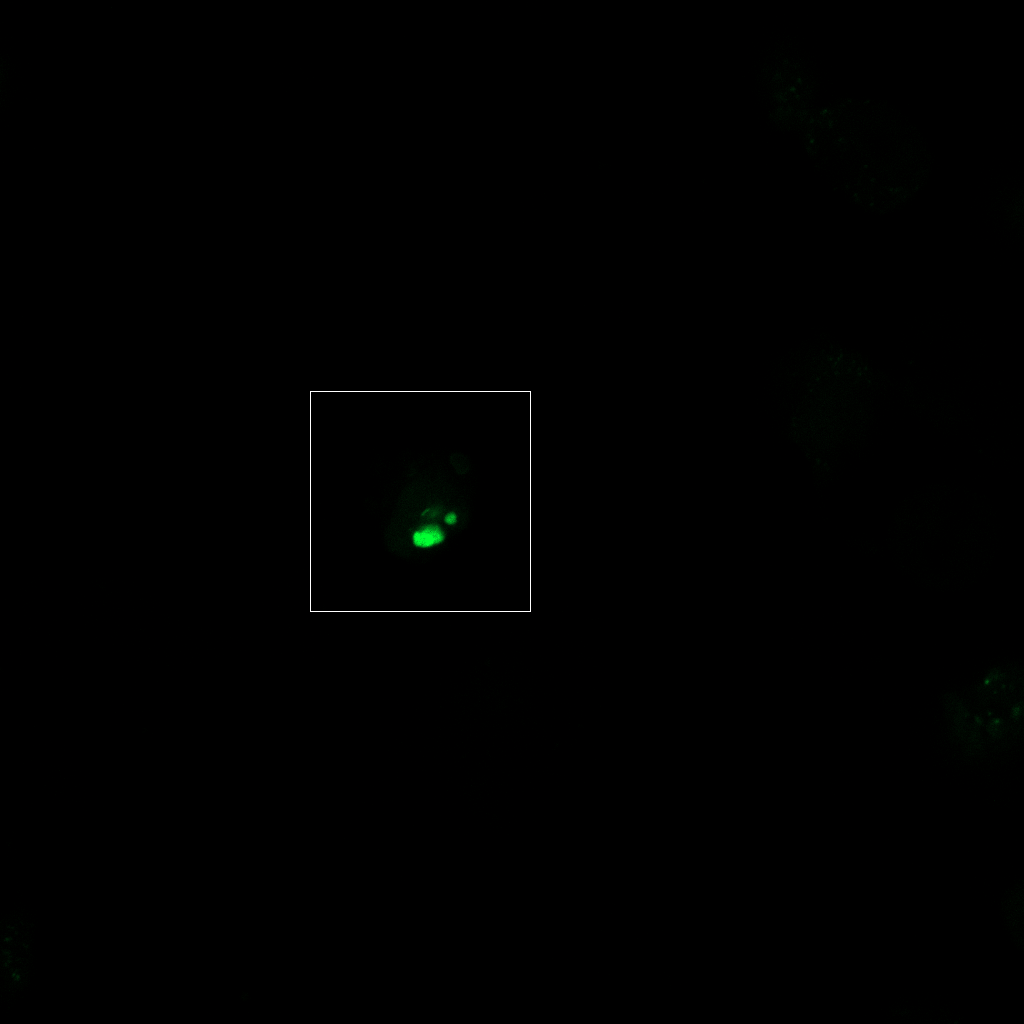

Supplement: Supplementary file 5 — Source data Fig. 2 [file 44318_2024_120_MOESM5_ESM.zip › Figure 2/2K/Mock/Bleaching.tif]

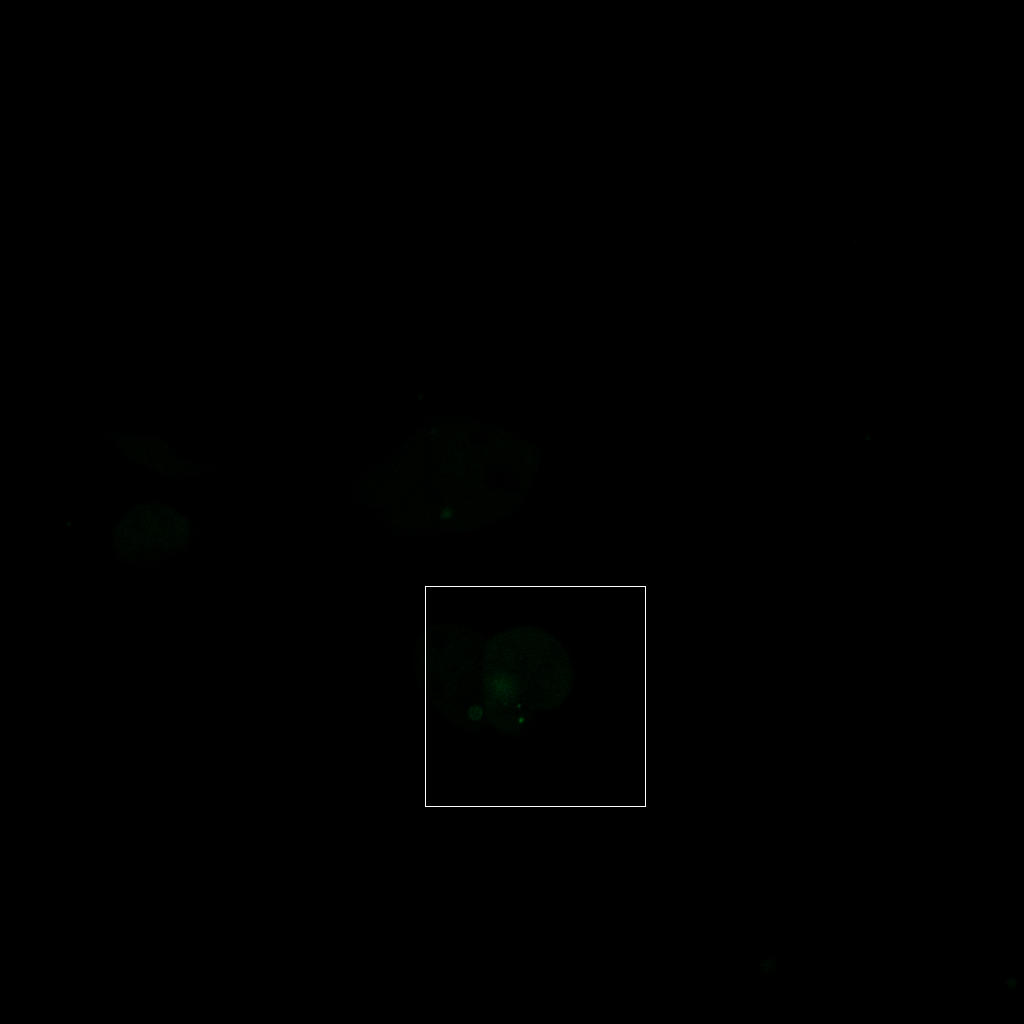

Supplement: Supplementary file 5 — Source data Fig. 2 [file 44318_2024_120_MOESM5_ESM.zip › Figure 2/2K/PYR-41/30s.tif]

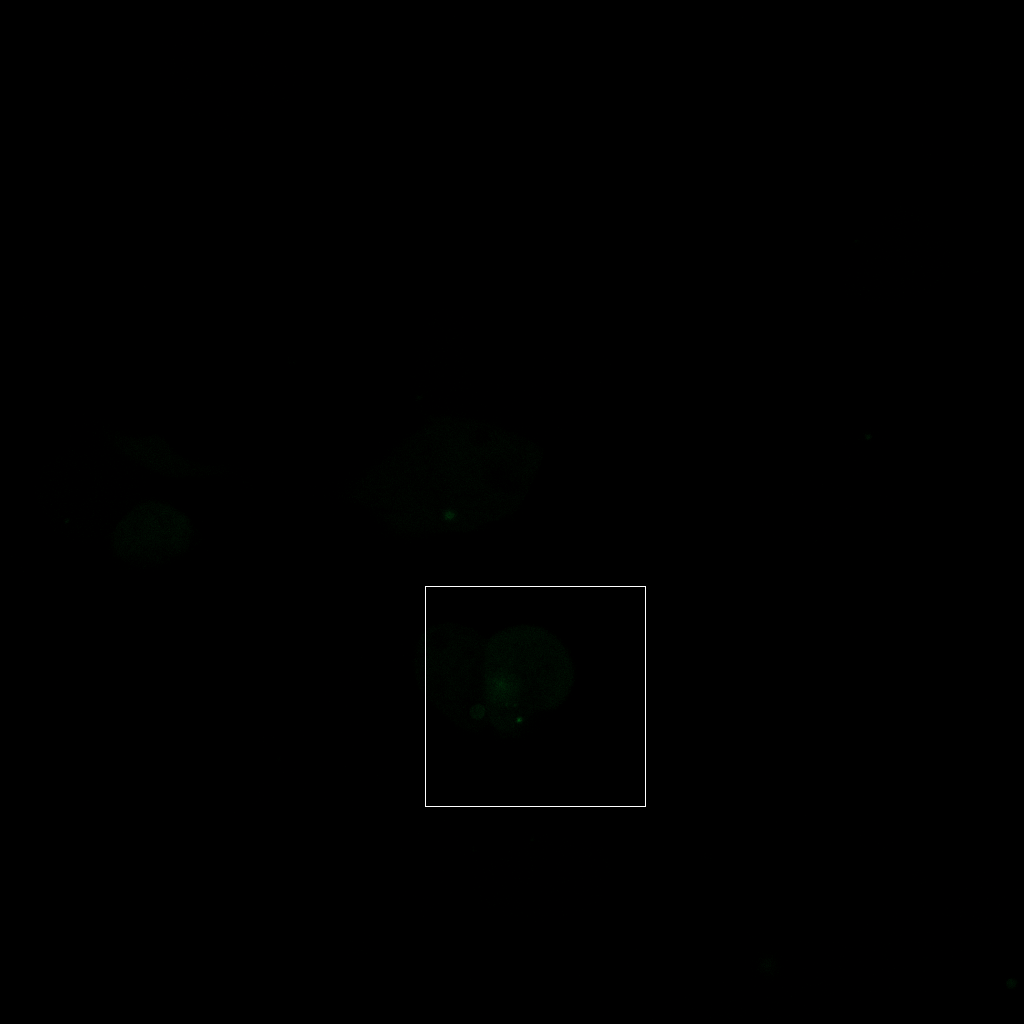

Supplement: Supplementary file 5 — Source data Fig. 2 [file 44318_2024_120_MOESM5_ESM.zip › Figure 2/2K/PYR-41/10s.tif]

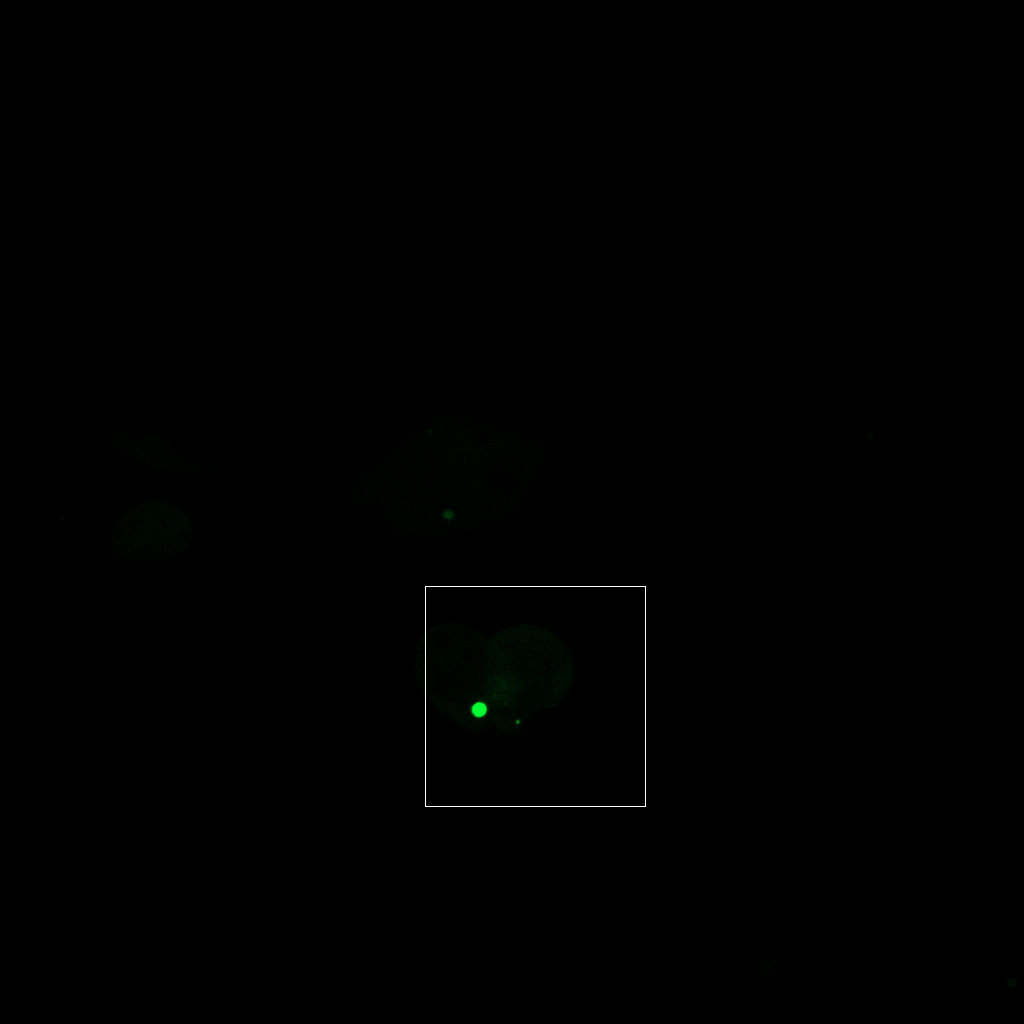

Supplement: Supplementary file 5 — Source data Fig. 2 [file 44318_2024_120_MOESM5_ESM.zip › Figure 2/2K/PYR-41/Pre.tif]

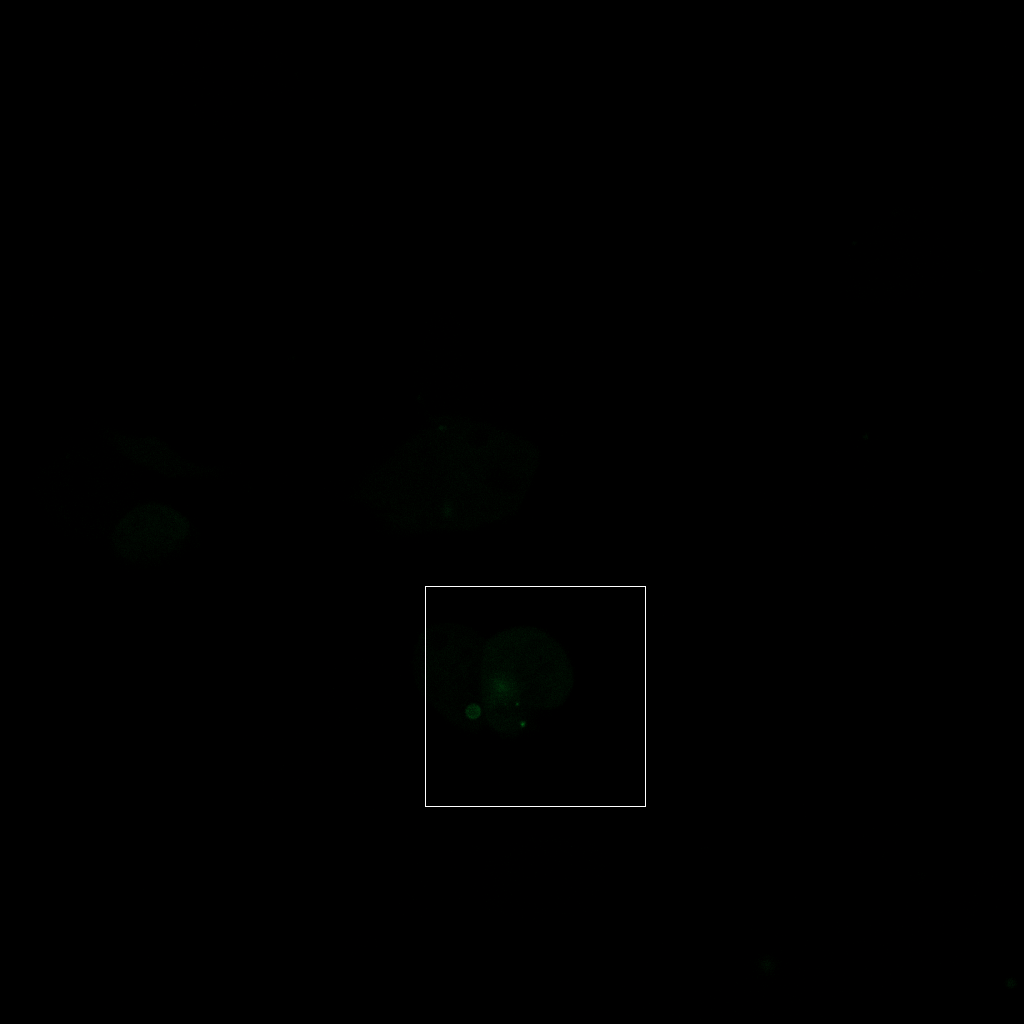

Supplement: Supplementary file 5 — Source data Fig. 2 [file 44318_2024_120_MOESM5_ESM.zip › Figure 2/2K/PYR-41/60s.tif]

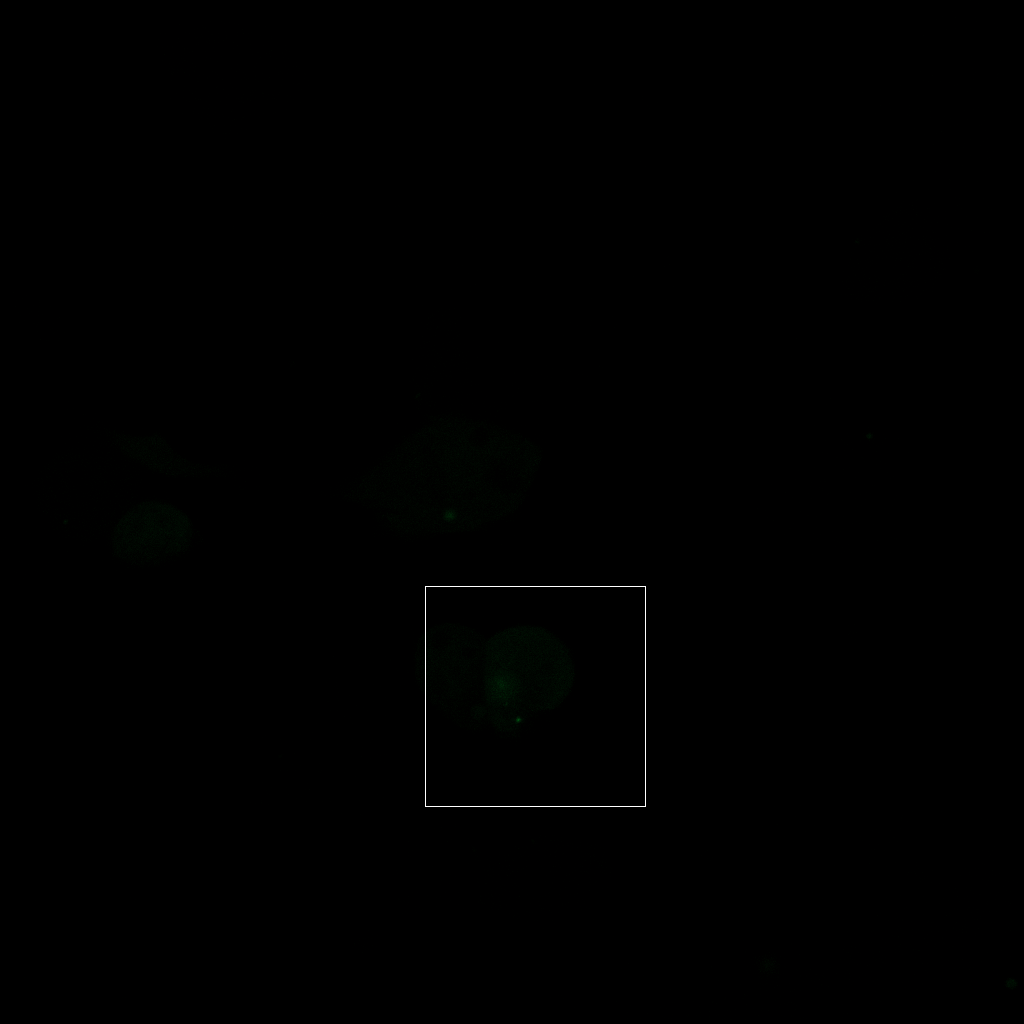

Supplement: Supplementary file 5 — Source data Fig. 2 [file 44318_2024_120_MOESM5_ESM.zip › Figure 2/2K/PYR-41/Bleaching.tif]
