# Supplementary figures and images for: Energy stress promotes P-bodies formation via lysine-63-linked polyubiquitination of HAX1 (part 2 of 4)
Source: EMBO J. 2024 May 20;43(13):11. doi: 10.1038/s44318-024-00120-6 (PMC11217408; doi:10.1038/s44318-024-00120-6)

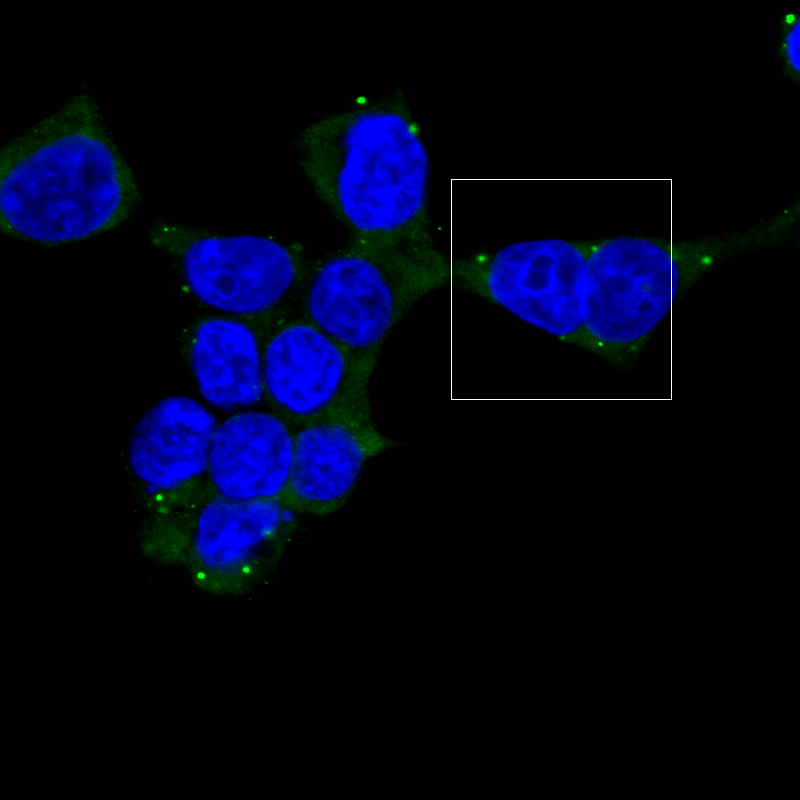

Supplement: Supplementary file 5 — Source data Fig. 2 [file 44318_2024_120_MOESM5_ESM.zip › Figure 2/2E/Mock/RA190.tif]

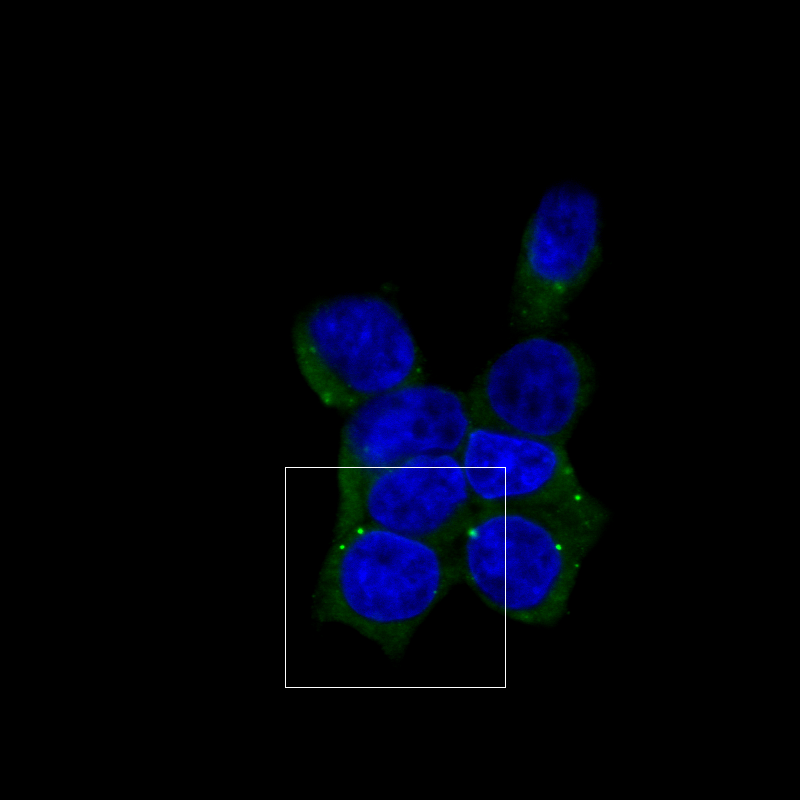

Supplement: Supplementary file 5 — Source data Fig. 2 [file 44318_2024_120_MOESM5_ESM.zip › Figure 2/2E/Mock/MG132.tif]

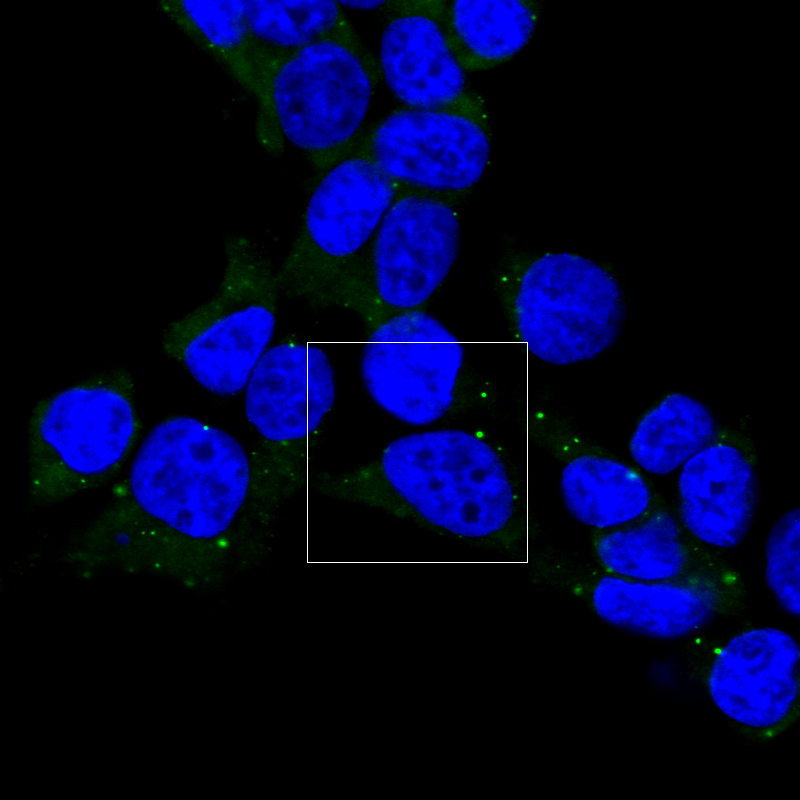

Supplement: Supplementary file 5 — Source data Fig. 2 [file 44318_2024_120_MOESM5_ESM.zip › Figure 2/2E/Mock/Mock.tif]

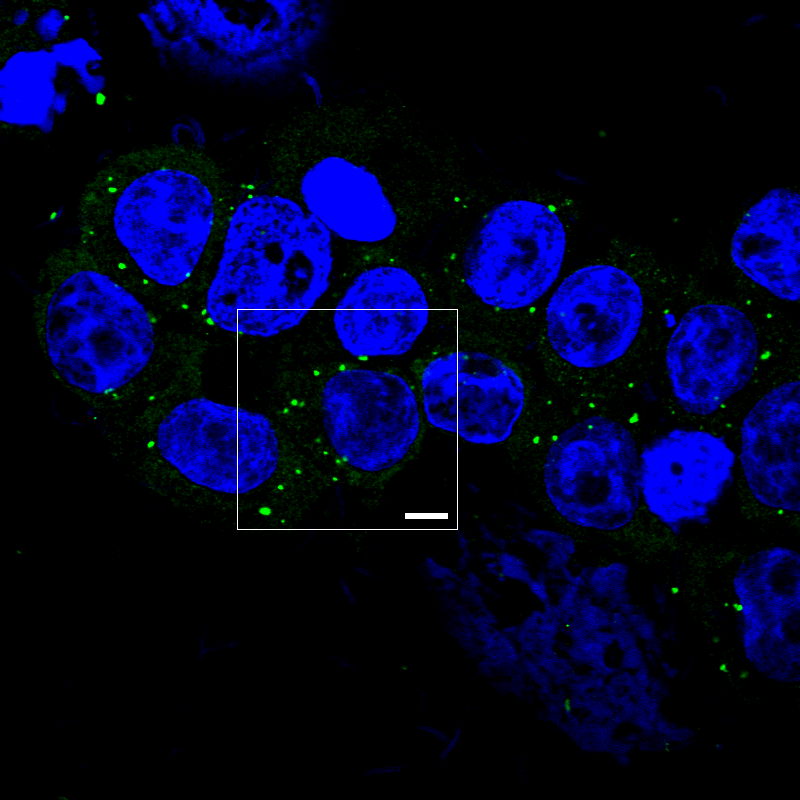

Supplement: Supplementary file 5 — Source data Fig. 2 [file 44318_2024_120_MOESM5_ESM.zip › Figure 2/2E/Oligomycin/RA190.tif]

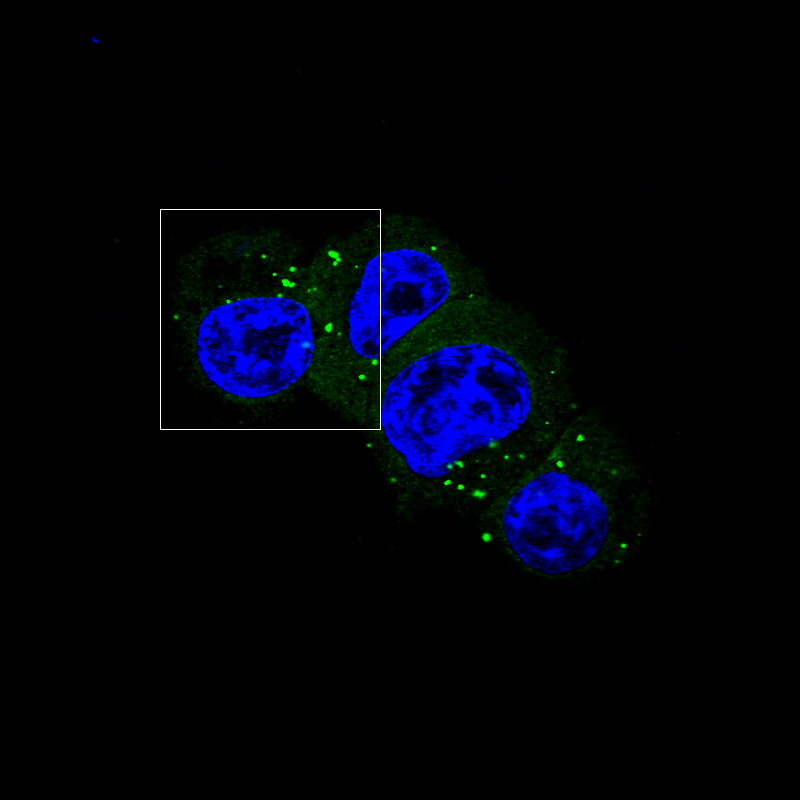

Supplement: Supplementary file 5 — Source data Fig. 2 [file 44318_2024_120_MOESM5_ESM.zip › Figure 2/2E/Oligomycin/MG132.tif]

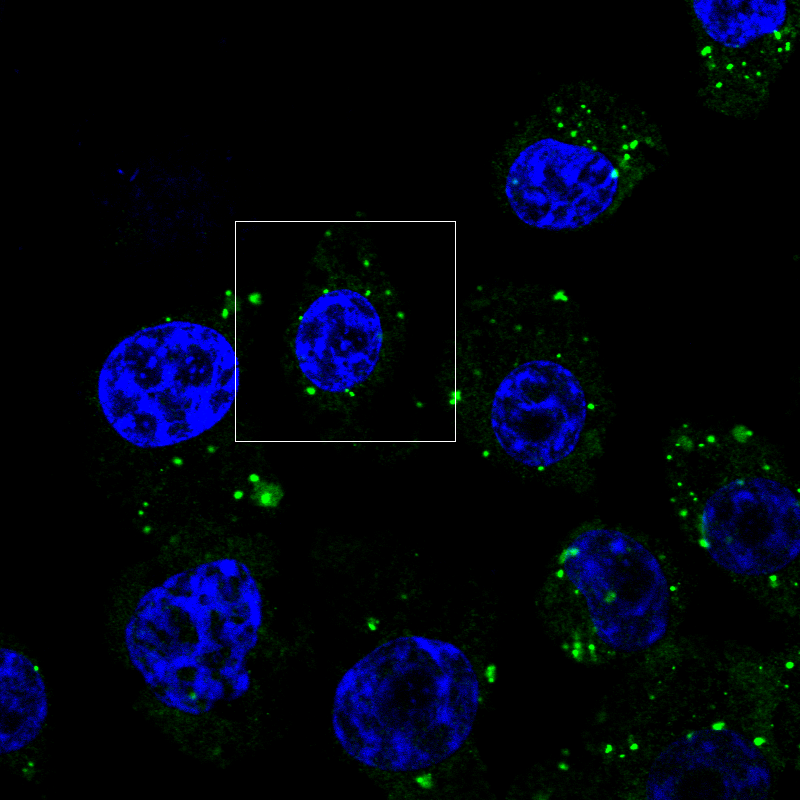

Supplement: Supplementary file 5 — Source data Fig. 2 [file 44318_2024_120_MOESM5_ESM.zip › Figure 2/2E/Oligomycin/Mock.tif]

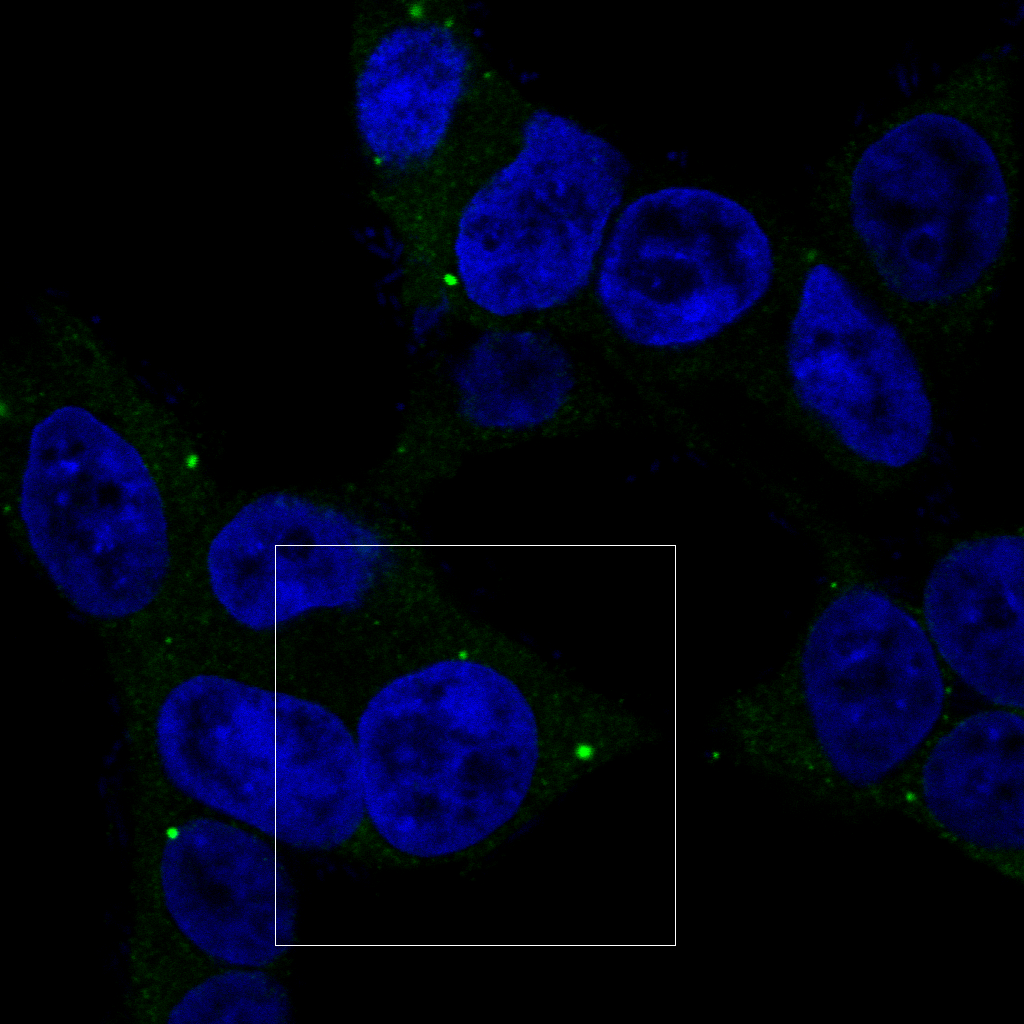

Supplement: Supplementary file 6 — Source data Fig. 3 [file 44318_2024_120_MOESM6_ESM.zip › Figure 3/3H/Vector.tif]

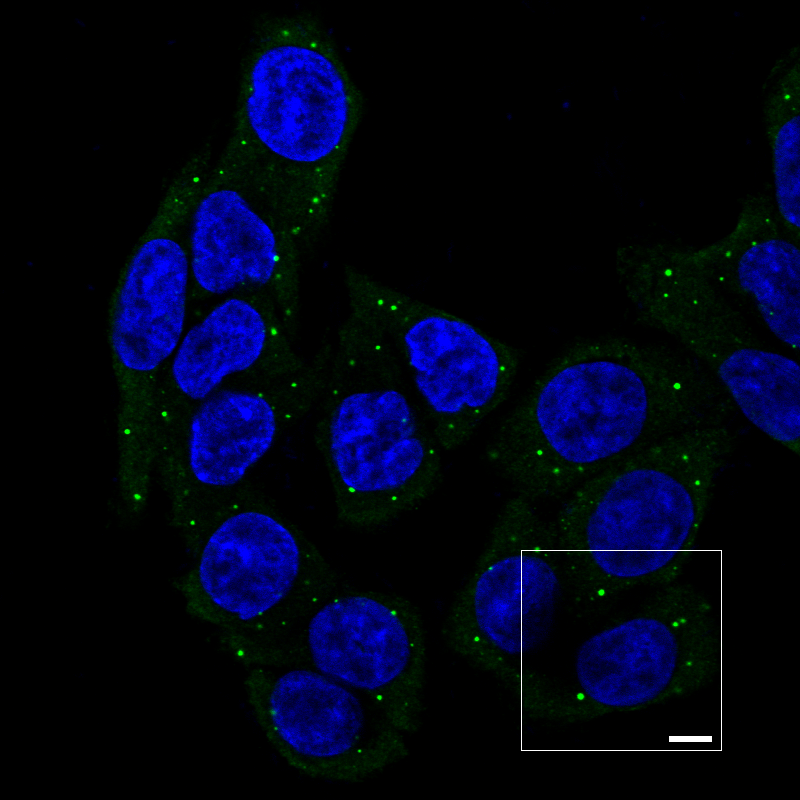

Supplement: Supplementary file 6 — Source data Fig. 3 [file 44318_2024_120_MOESM6_ESM.zip › Figure 3/3H/TRIM23.tif]

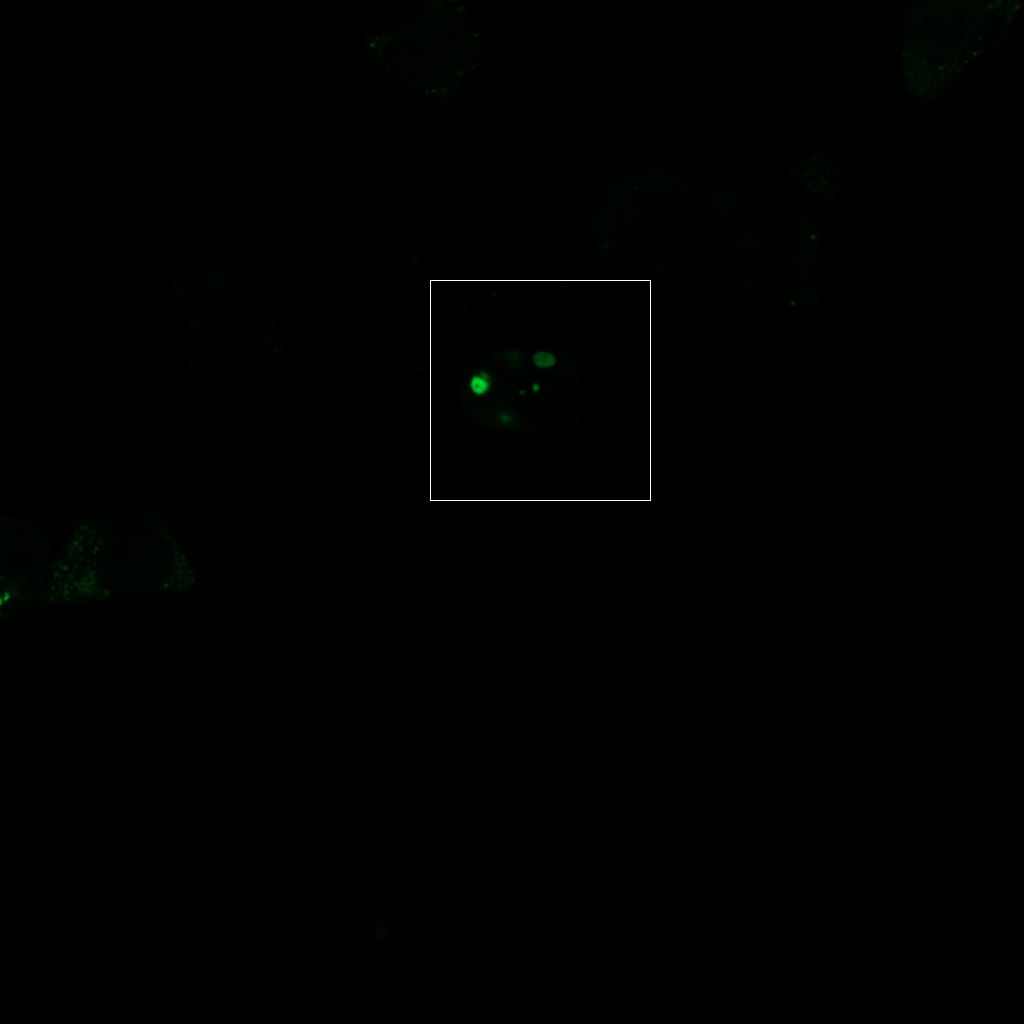

Supplement: Supplementary file 6 — Source data Fig. 3 [file 44318_2024_120_MOESM6_ESM.zip › Figure 3/3L/Vevtor/30s.tif]

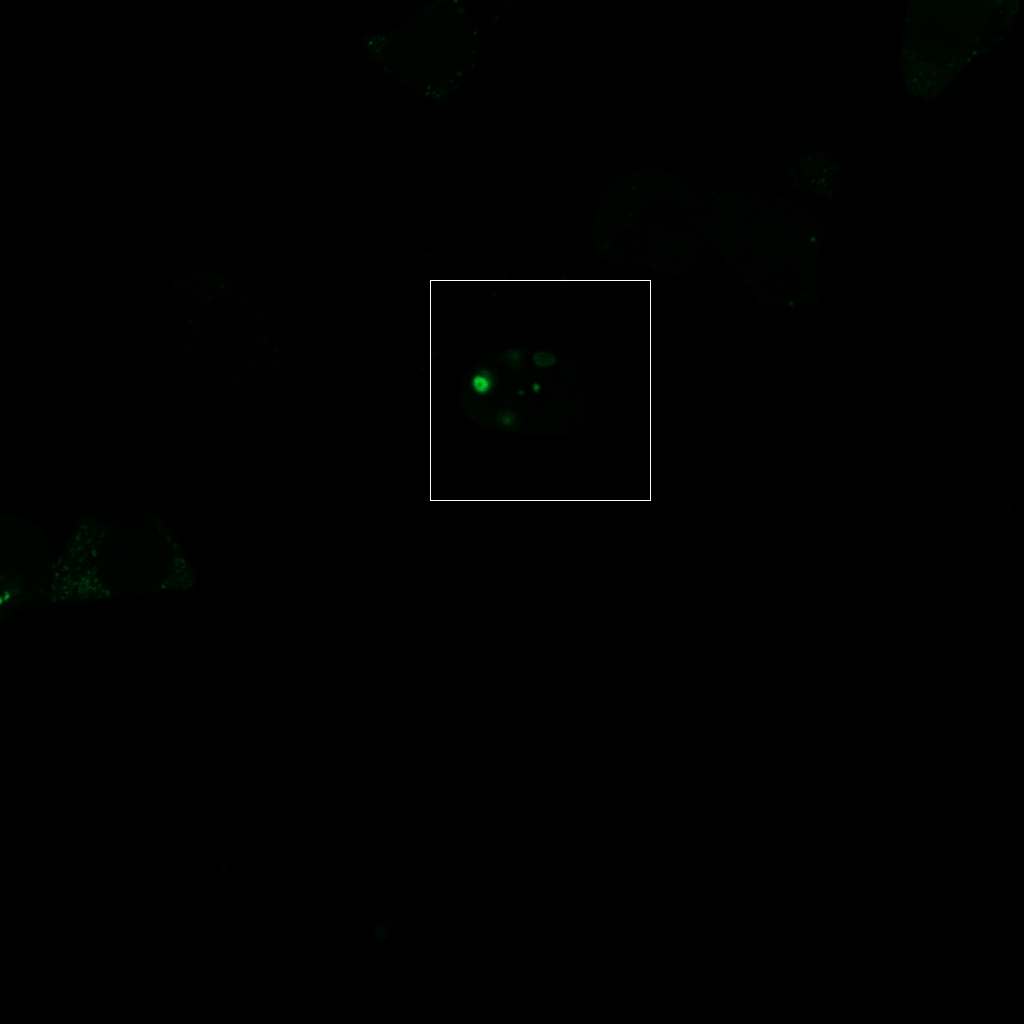

Supplement: Supplementary file 6 — Source data Fig. 3 [file 44318_2024_120_MOESM6_ESM.zip › Figure 3/3L/Vevtor/10s.tif]

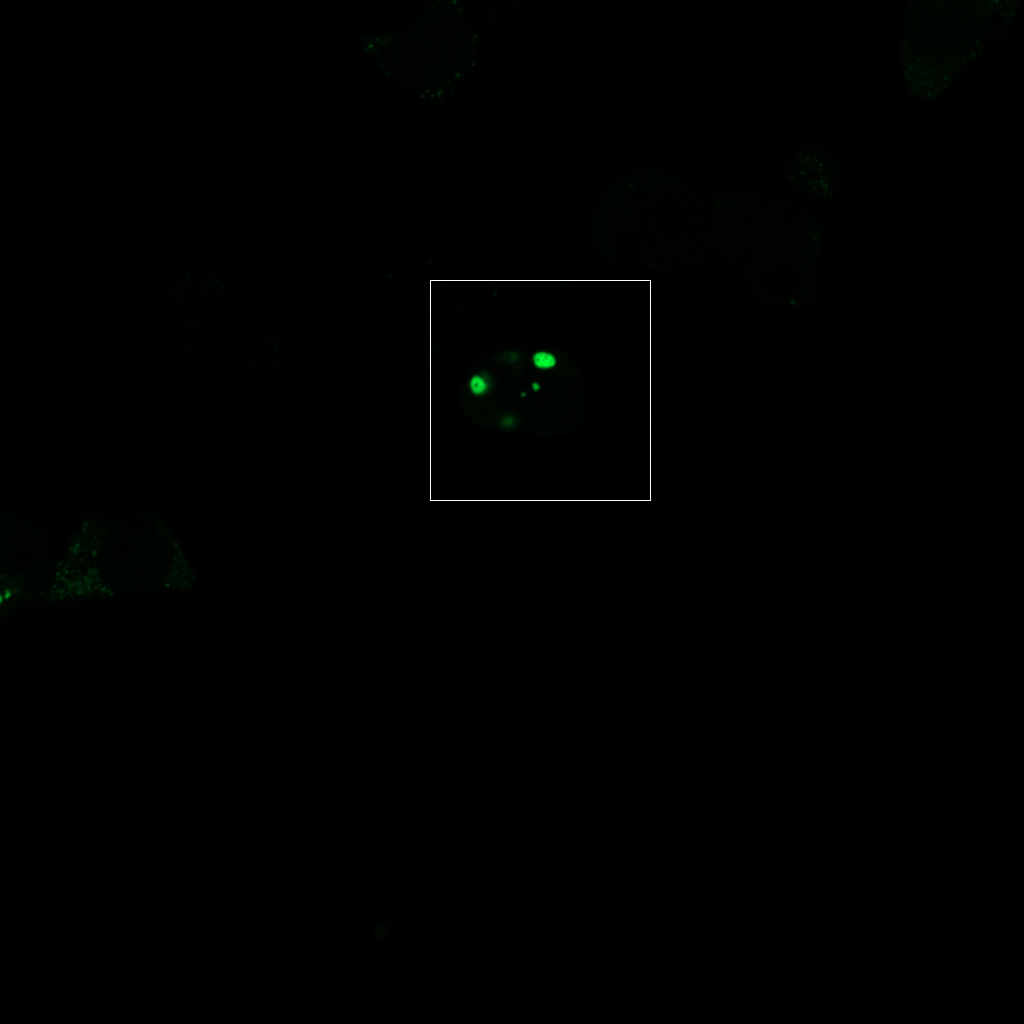

Supplement: Supplementary file 6 — Source data Fig. 3 [file 44318_2024_120_MOESM6_ESM.zip › Figure 3/3L/Vevtor/Pre.tif]

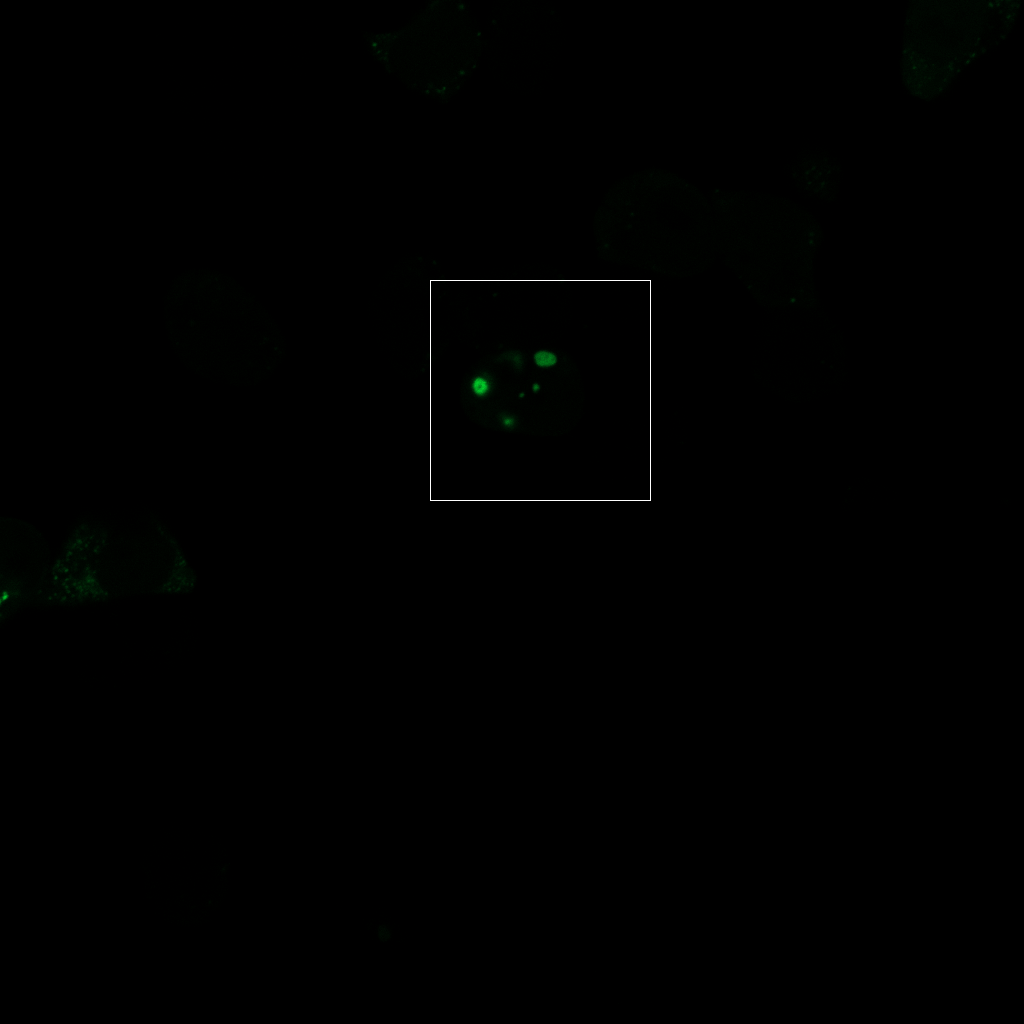

Supplement: Supplementary file 6 — Source data Fig. 3 [file 44318_2024_120_MOESM6_ESM.zip › Figure 3/3L/Vevtor/60s.tif]

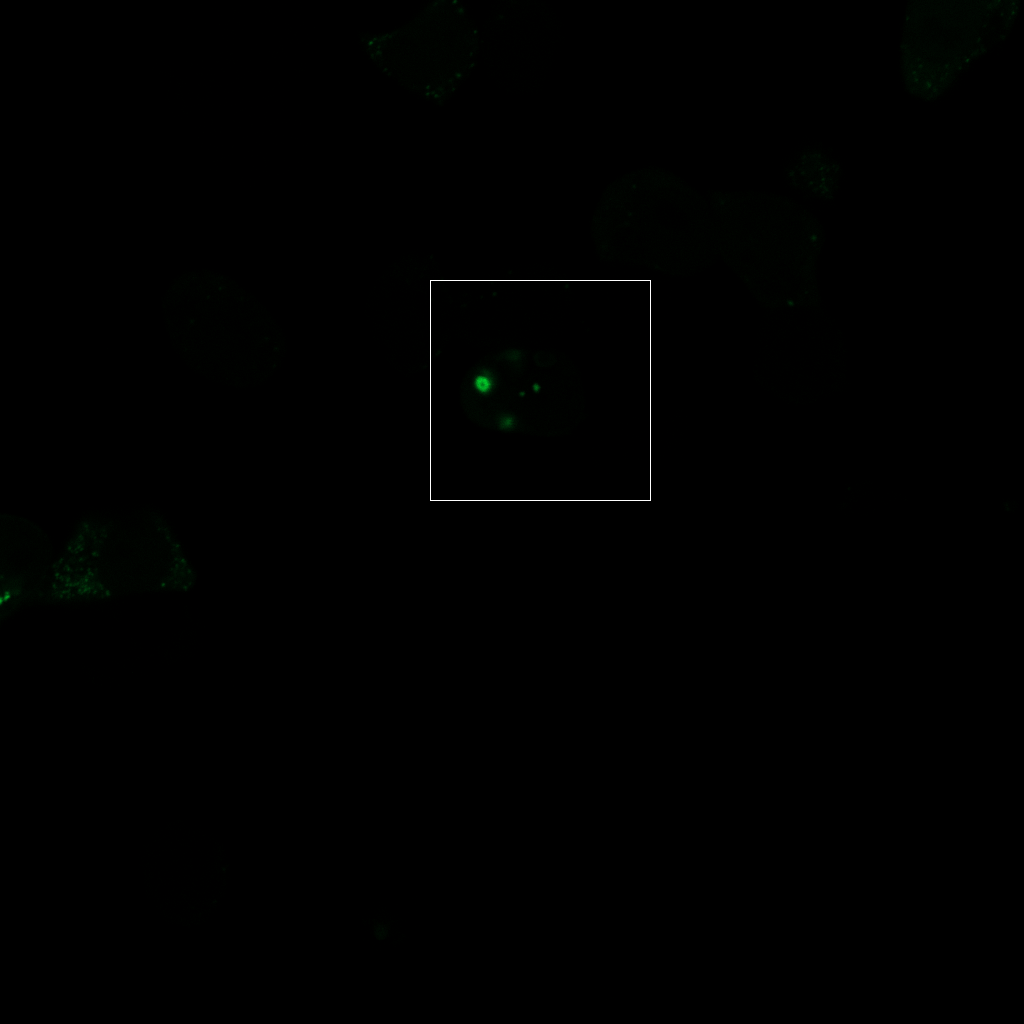

Supplement: Supplementary file 6 — Source data Fig. 3 [file 44318_2024_120_MOESM6_ESM.zip › Figure 3/3L/Vevtor/Bleaching.tif]

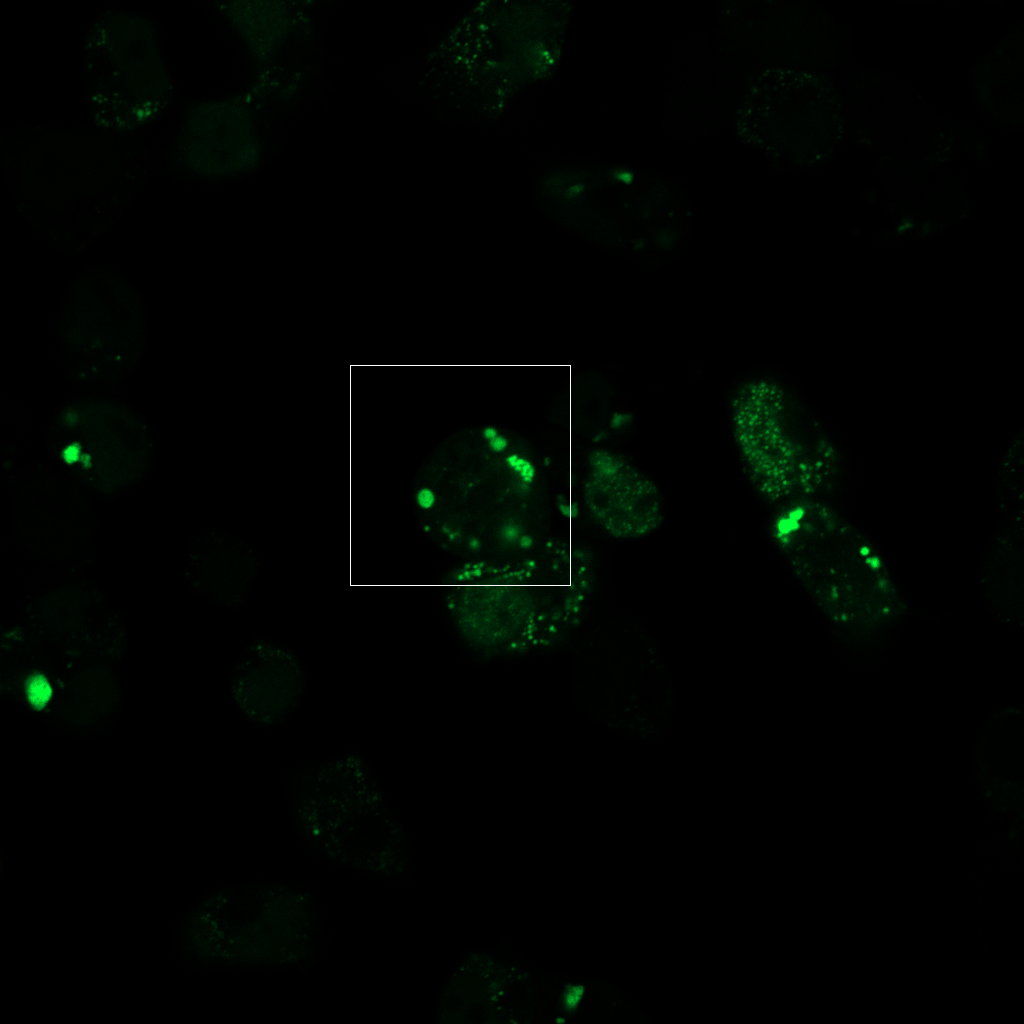

Supplement: Supplementary file 6 — Source data Fig. 3 [file 44318_2024_120_MOESM6_ESM.zip › Figure 3/3L/TRIM23/30s.tif]

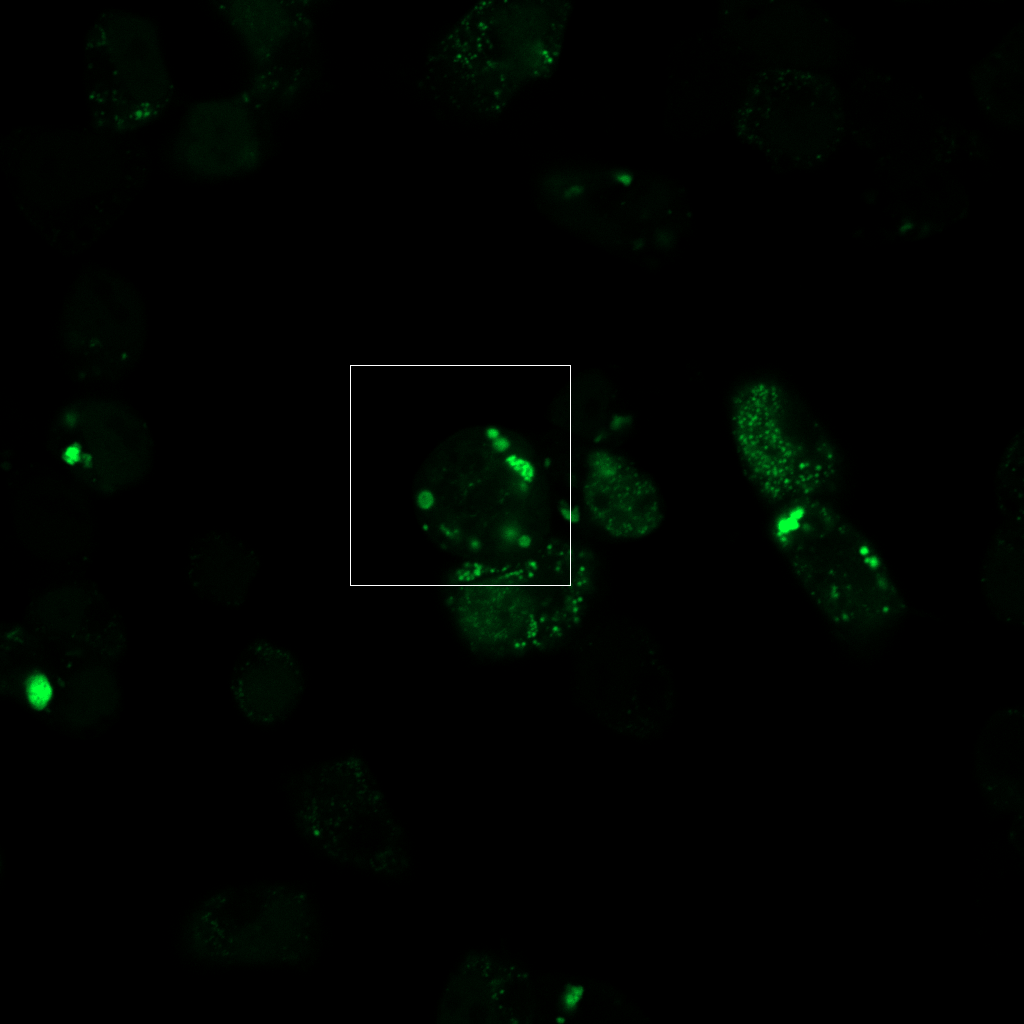

Supplement: Supplementary file 6 — Source data Fig. 3 [file 44318_2024_120_MOESM6_ESM.zip › Figure 3/3L/TRIM23/10s.tif]

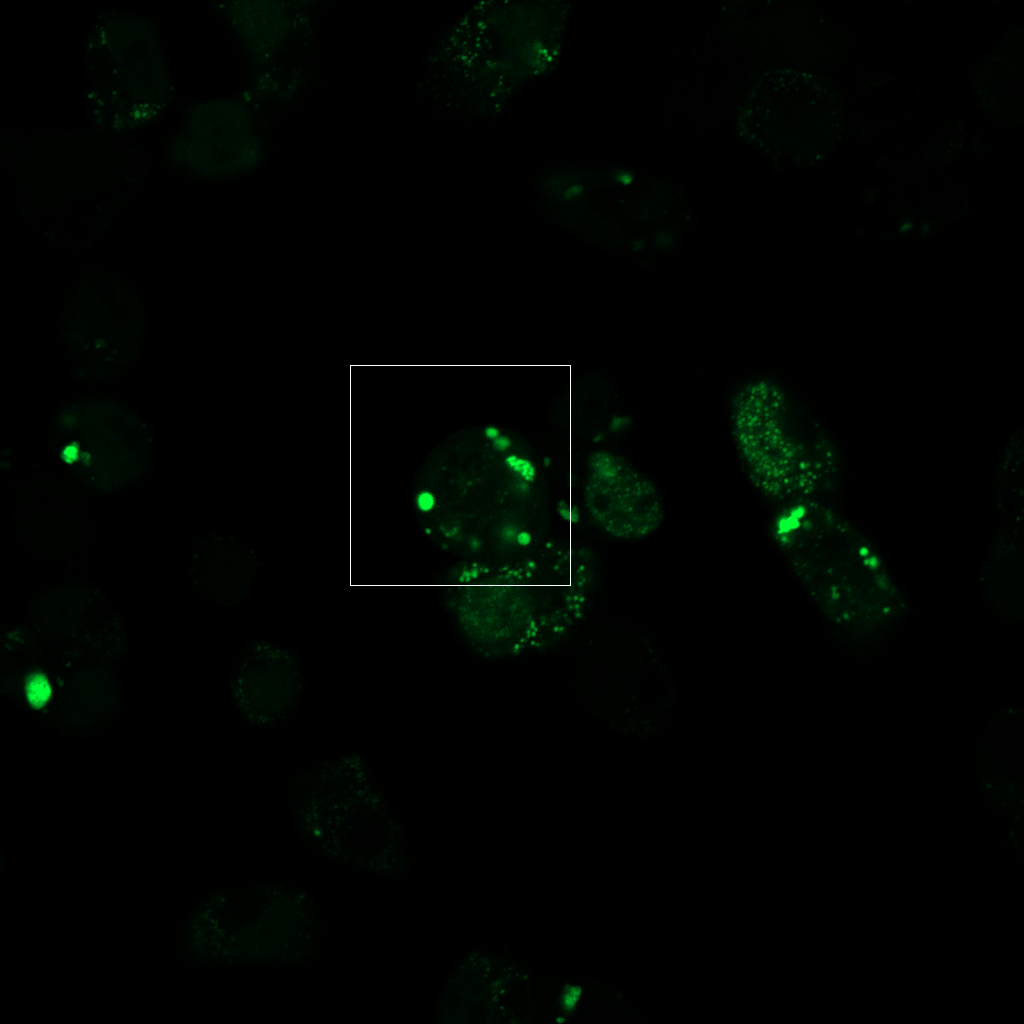

Supplement: Supplementary file 6 — Source data Fig. 3 [file 44318_2024_120_MOESM6_ESM.zip › Figure 3/3L/TRIM23/Pre.tif]

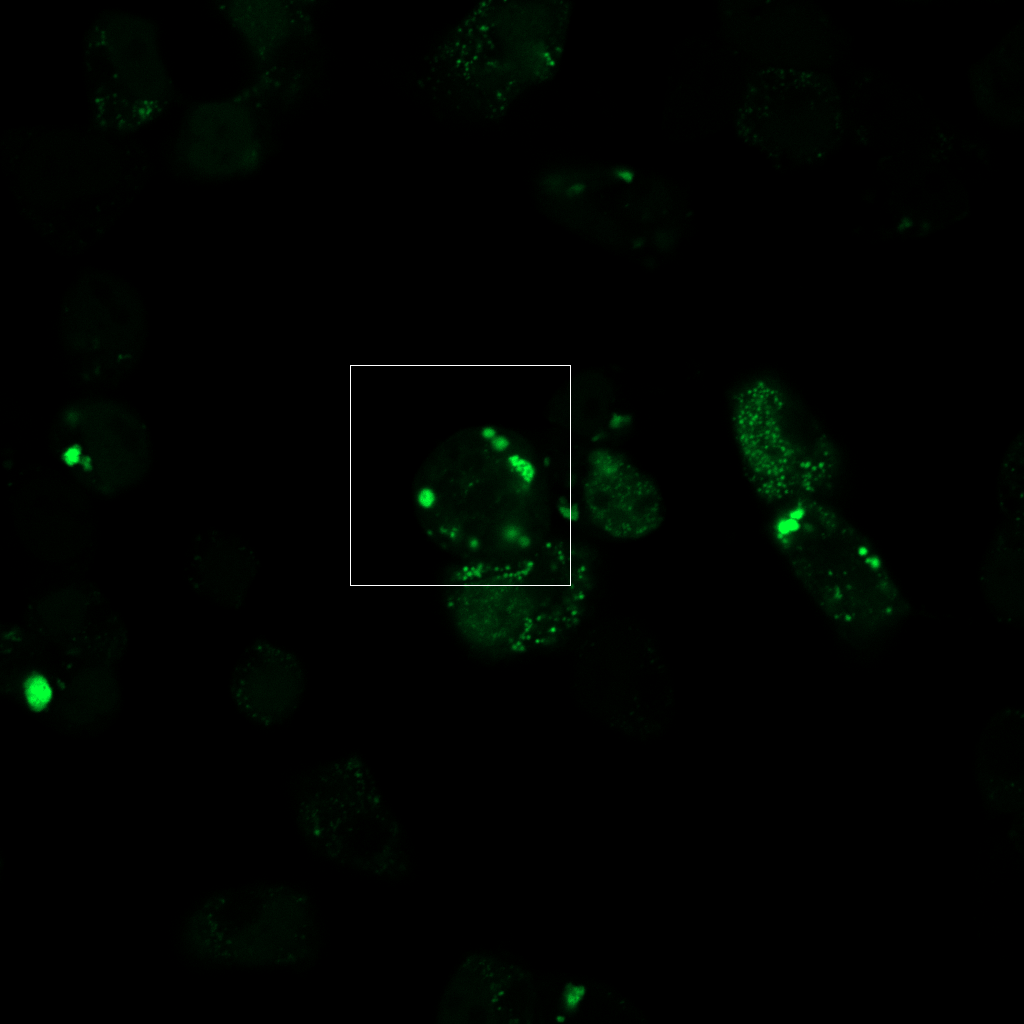

Supplement: Supplementary file 6 — Source data Fig. 3 [file 44318_2024_120_MOESM6_ESM.zip › Figure 3/3L/TRIM23/60s.tif]

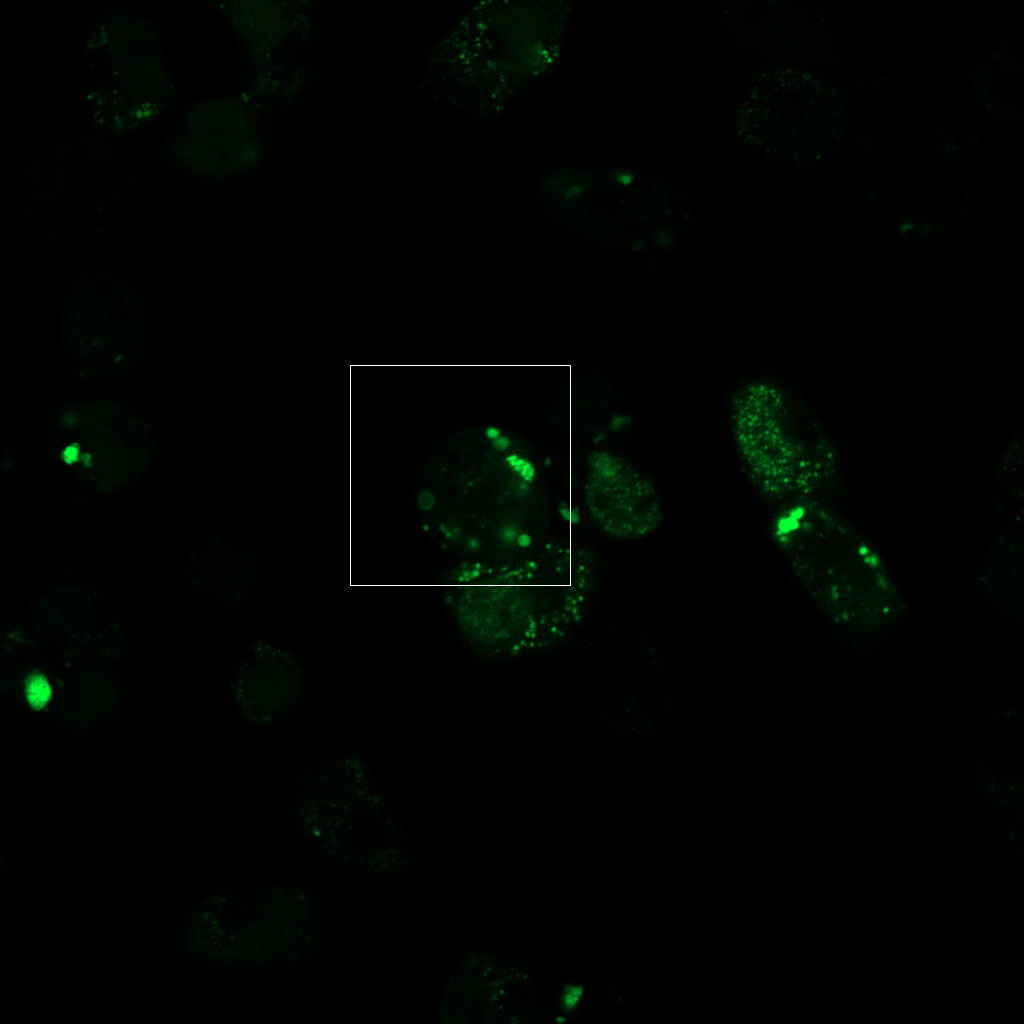

Supplement: Supplementary file 6 — Source data Fig. 3 [file 44318_2024_120_MOESM6_ESM.zip › Figure 3/3L/TRIM23/Bleaching.tif]

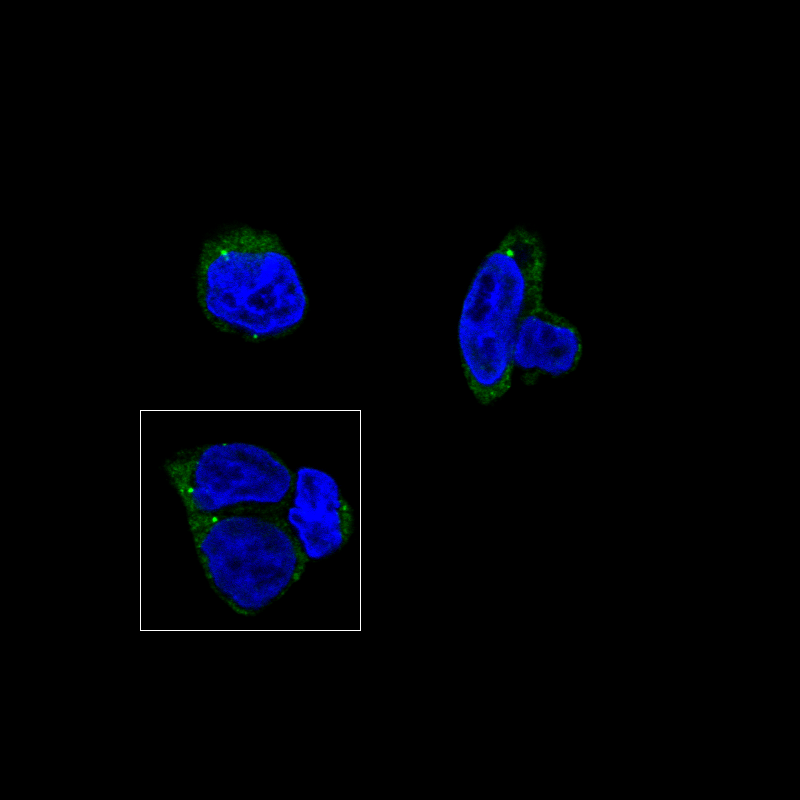

Supplement: Supplementary file 6 — Source data Fig. 3 [file 44318_2024_120_MOESM6_ESM.zip › Figure 3/3D/Mock/sgCtrl.tif]

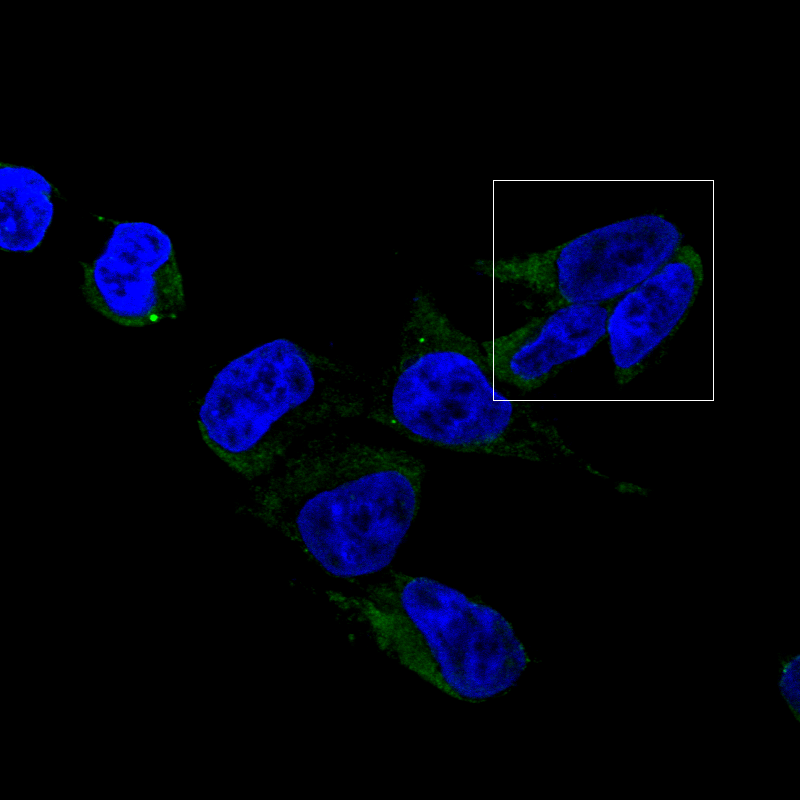

Supplement: Supplementary file 6 — Source data Fig. 3 [file 44318_2024_120_MOESM6_ESM.zip › Figure 3/3D/Mock/sgTRIM23.tif]

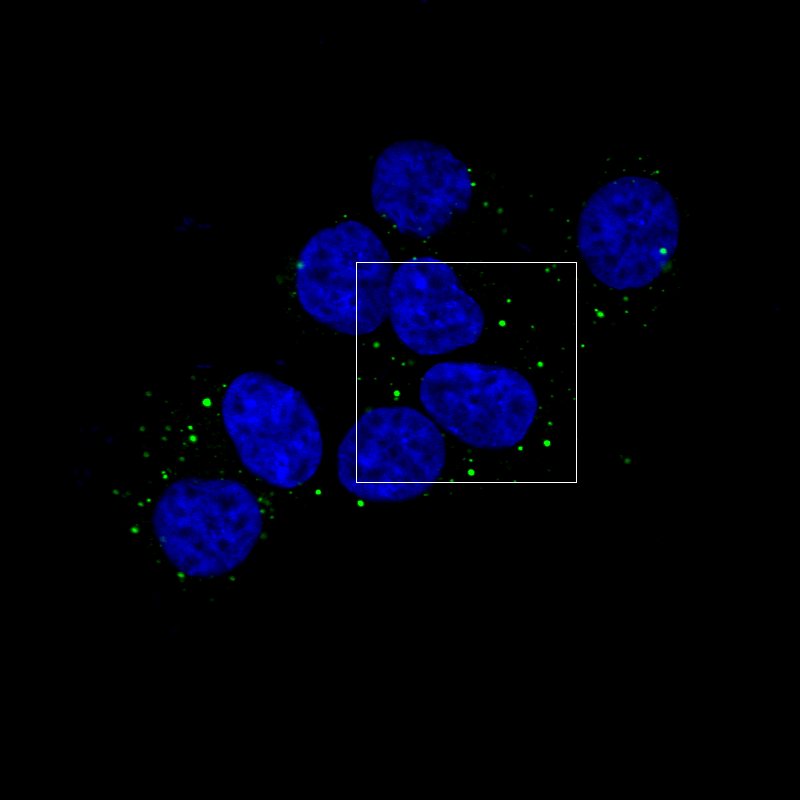

Supplement: Supplementary file 6 — Source data Fig. 3 [file 44318_2024_120_MOESM6_ESM.zip › Figure 3/3D/Oligomycin/sgCtrl.tif]

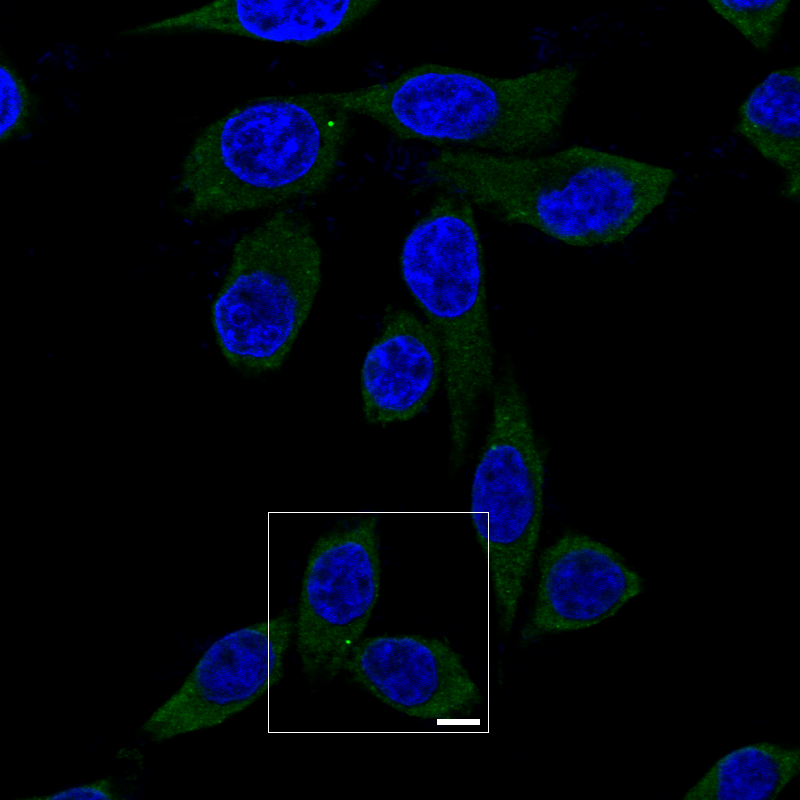

Supplement: Supplementary file 6 — Source data Fig. 3 [file 44318_2024_120_MOESM6_ESM.zip › Figure 3/3D/Oligomycin/sgTRIM23.tif]

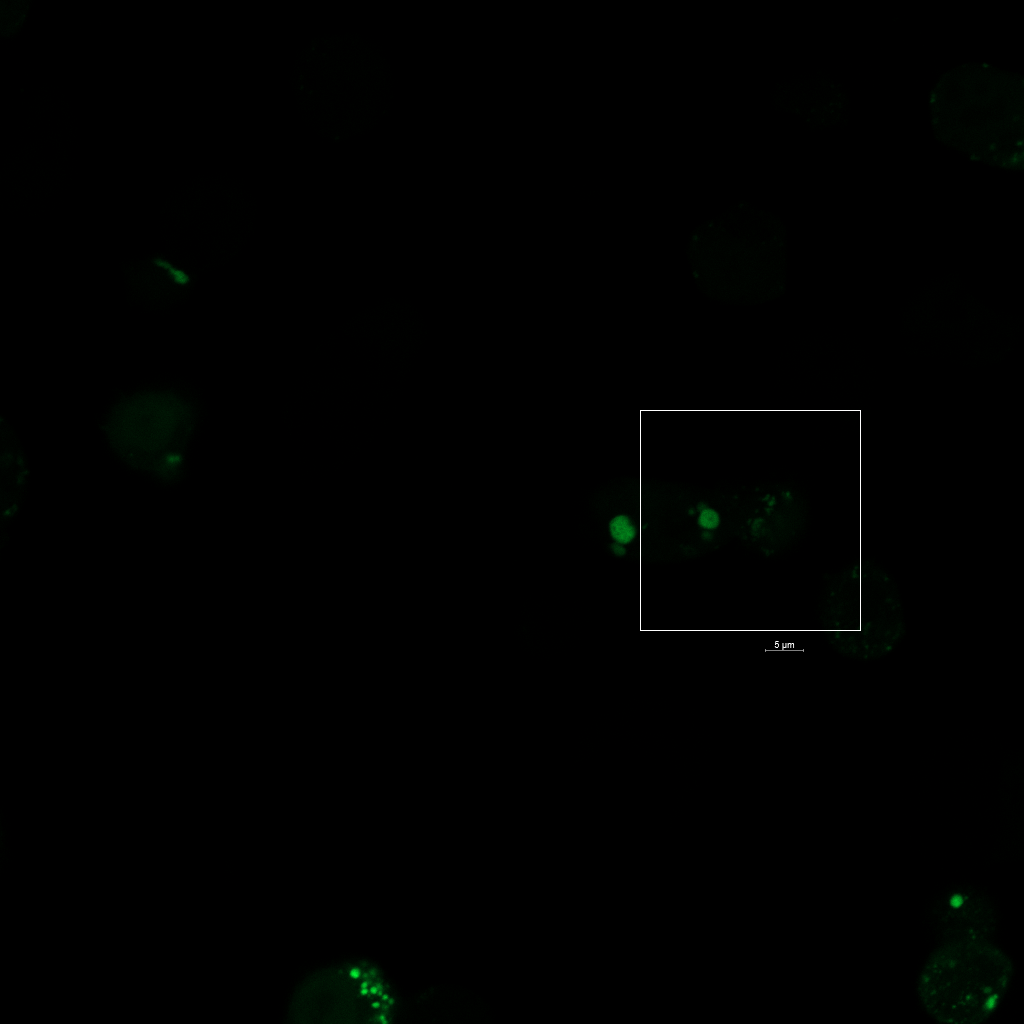

Supplement: Supplementary file 6 — Source data Fig. 3 [file 44318_2024_120_MOESM6_ESM.zip › Figure 3/3J/sgCtrl/30s.tif]

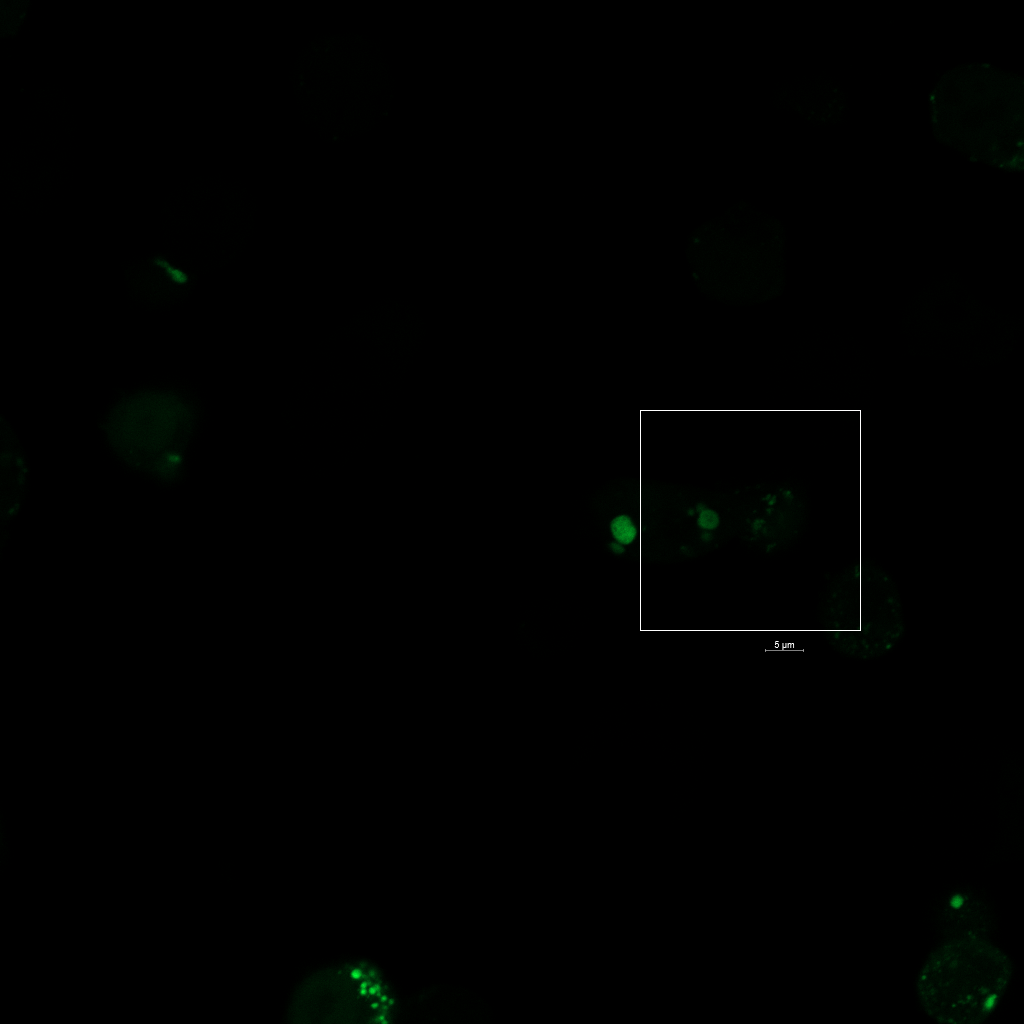

Supplement: Supplementary file 6 — Source data Fig. 3 [file 44318_2024_120_MOESM6_ESM.zip › Figure 3/3J/sgCtrl/10s.tif]

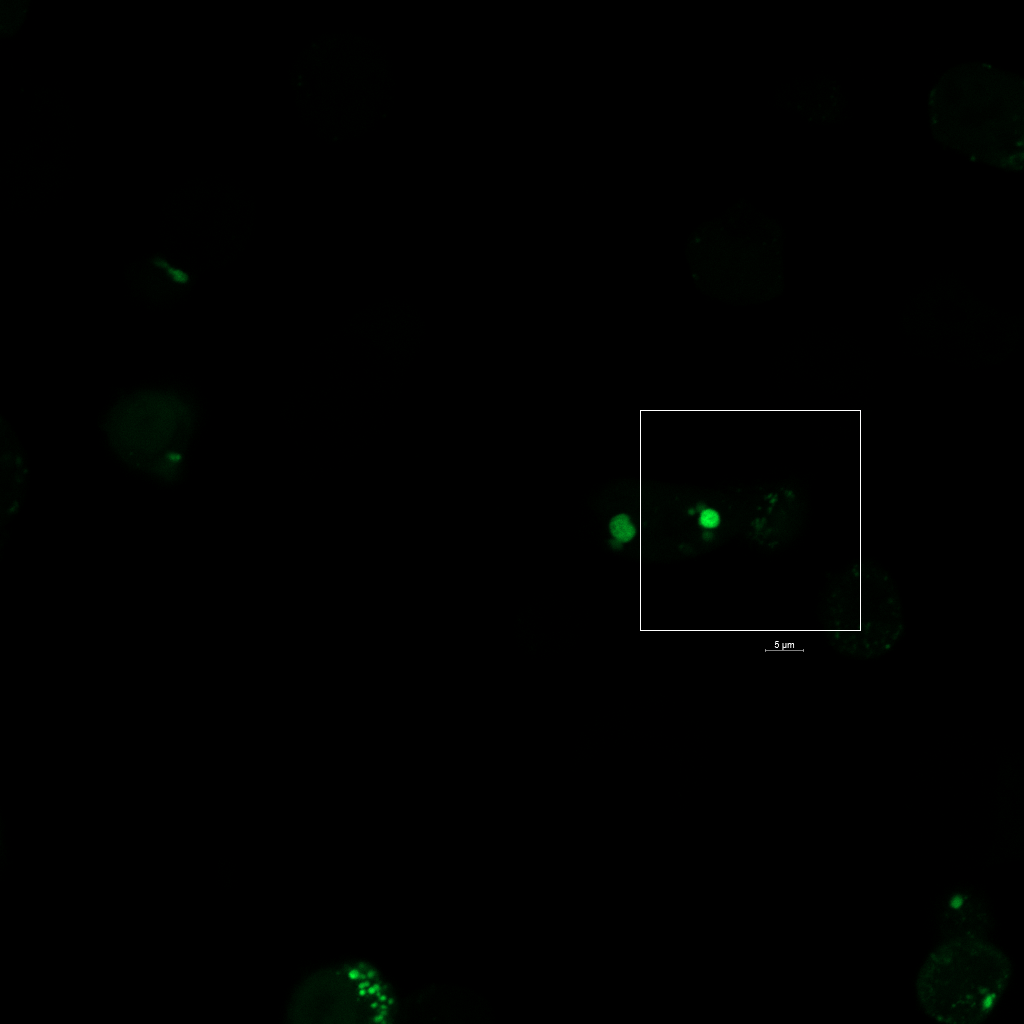

Supplement: Supplementary file 6 — Source data Fig. 3 [file 44318_2024_120_MOESM6_ESM.zip › Figure 3/3J/sgCtrl/Pre.tif]

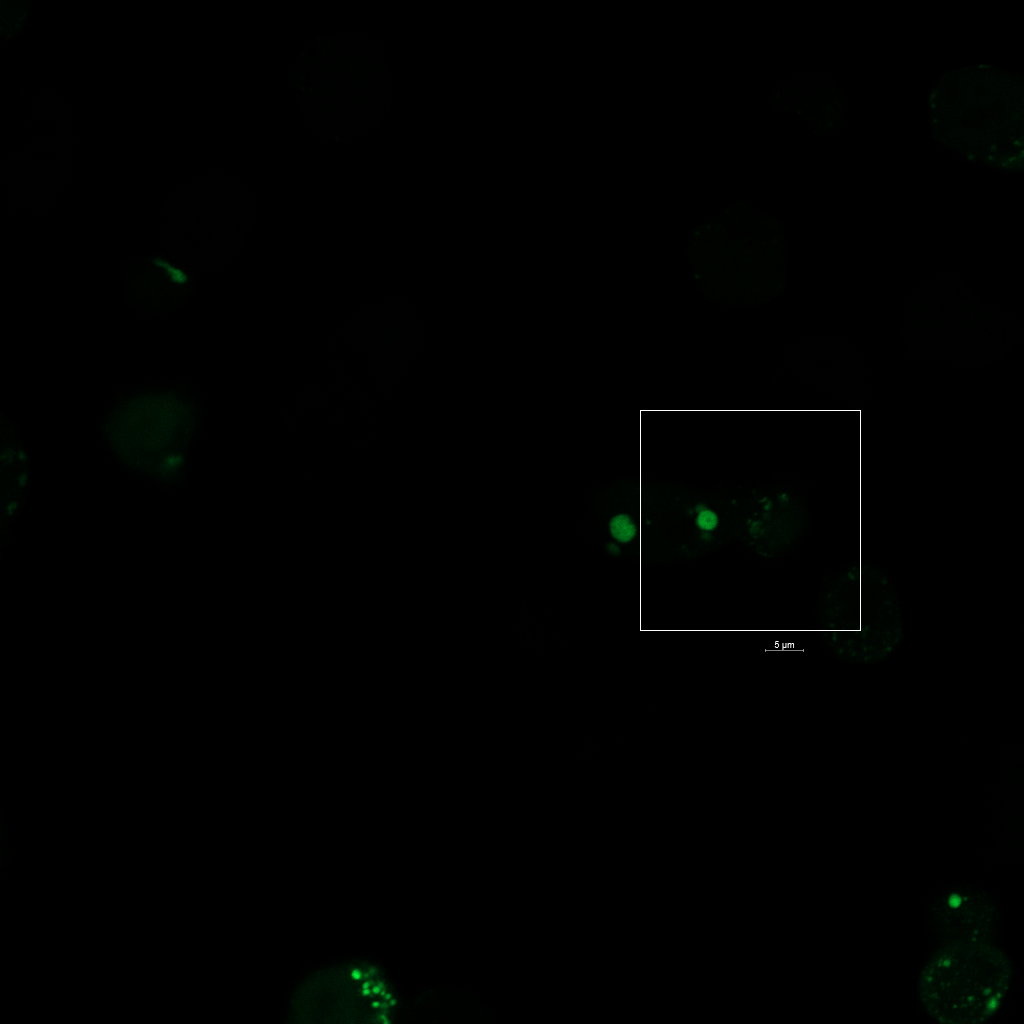

Supplement: Supplementary file 6 — Source data Fig. 3 [file 44318_2024_120_MOESM6_ESM.zip › Figure 3/3J/sgCtrl/60s.tif]

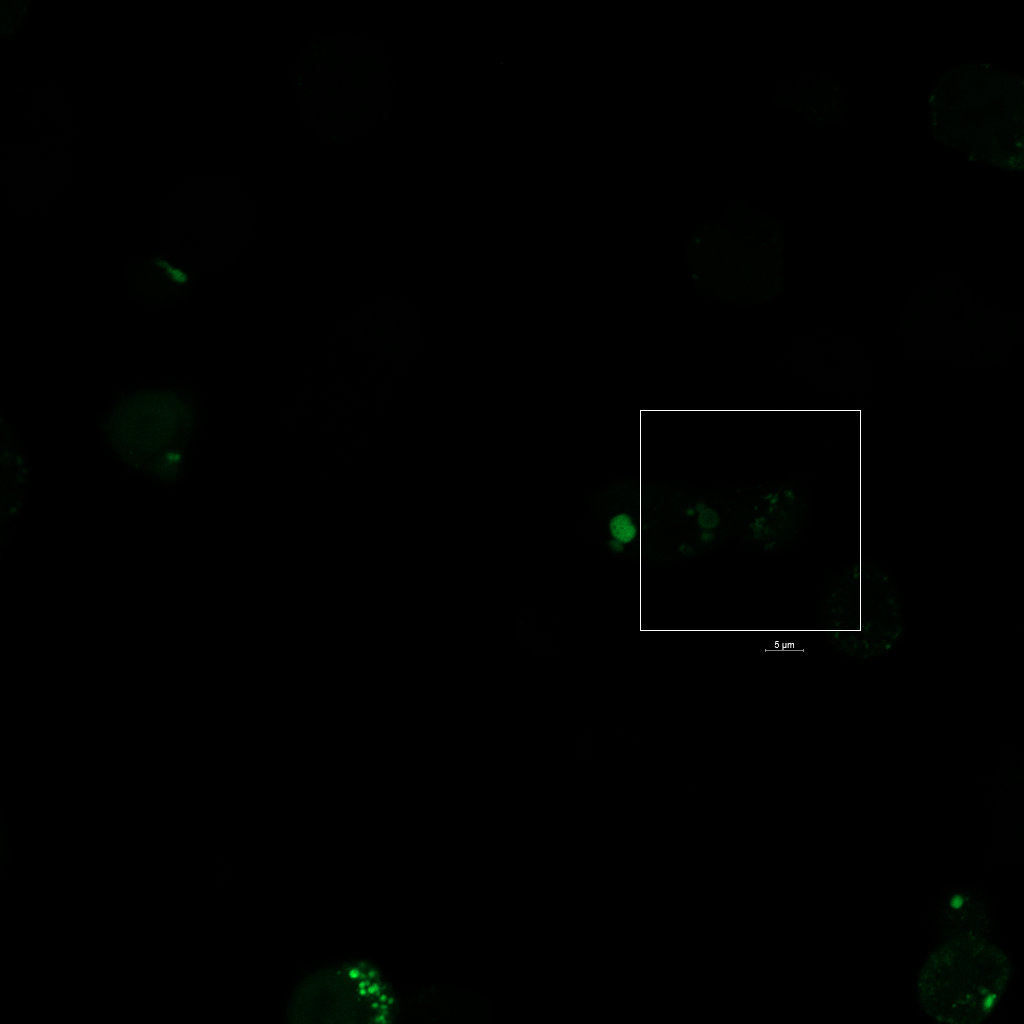

Supplement: Supplementary file 6 — Source data Fig. 3 [file 44318_2024_120_MOESM6_ESM.zip › Figure 3/3J/sgCtrl/Bleaching.tif]

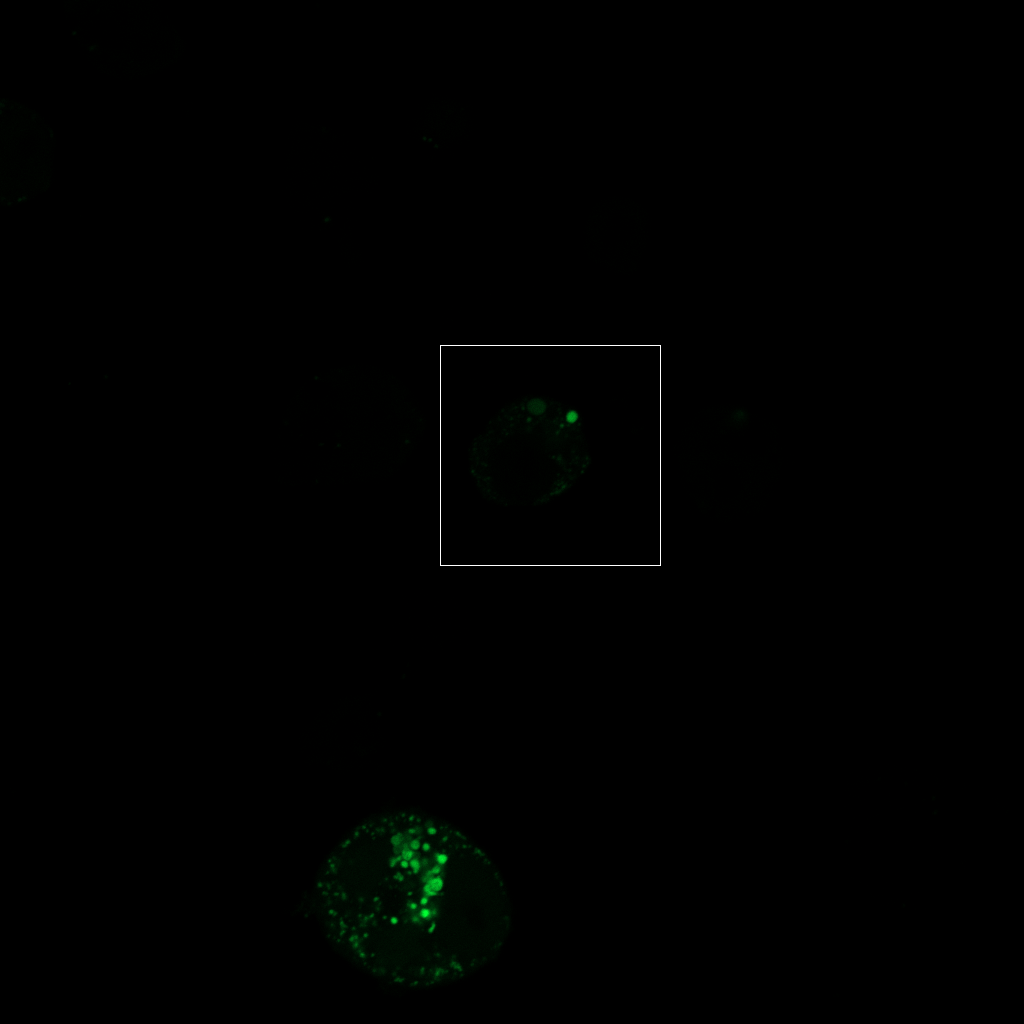

Supplement: Supplementary file 6 — Source data Fig. 3 [file 44318_2024_120_MOESM6_ESM.zip › Figure 3/3J/sgTRIM23/30s.tif]

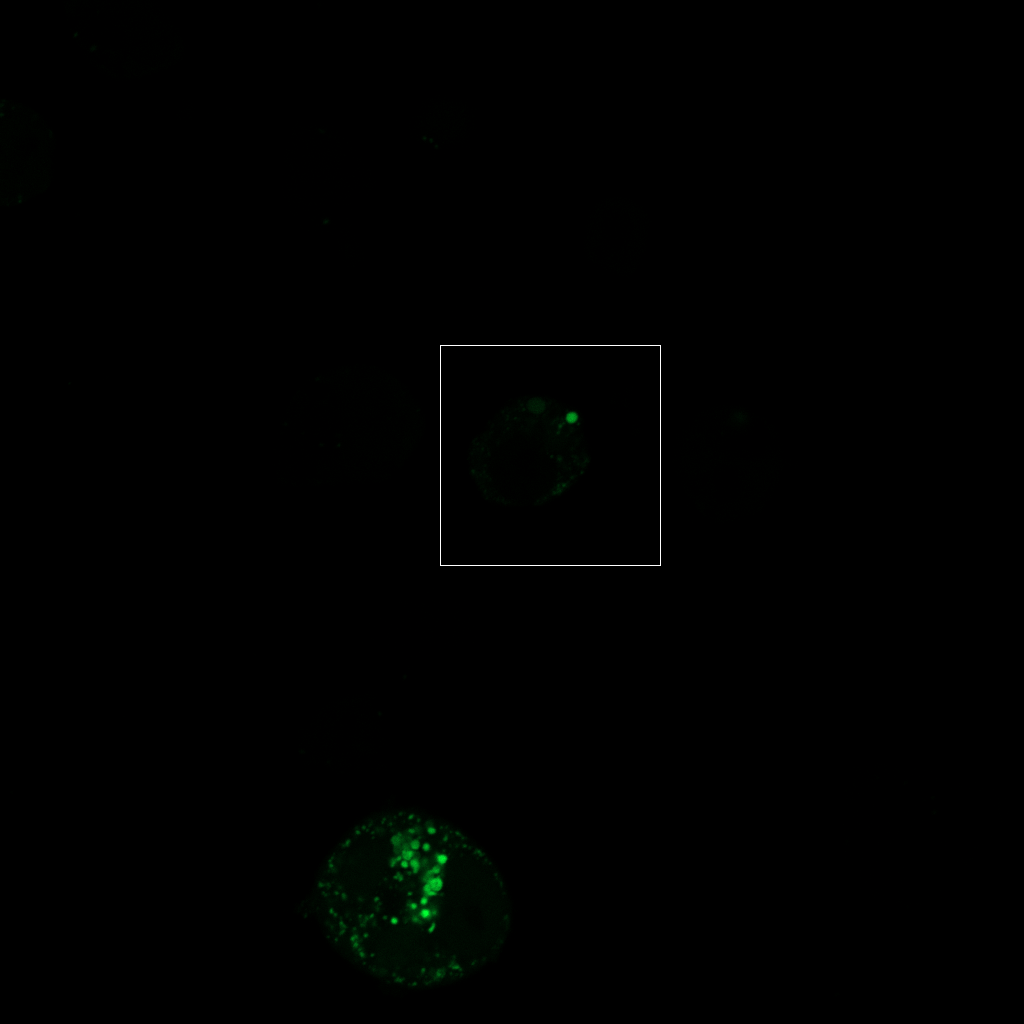

Supplement: Supplementary file 6 — Source data Fig. 3 [file 44318_2024_120_MOESM6_ESM.zip › Figure 3/3J/sgTRIM23/10s.tif]

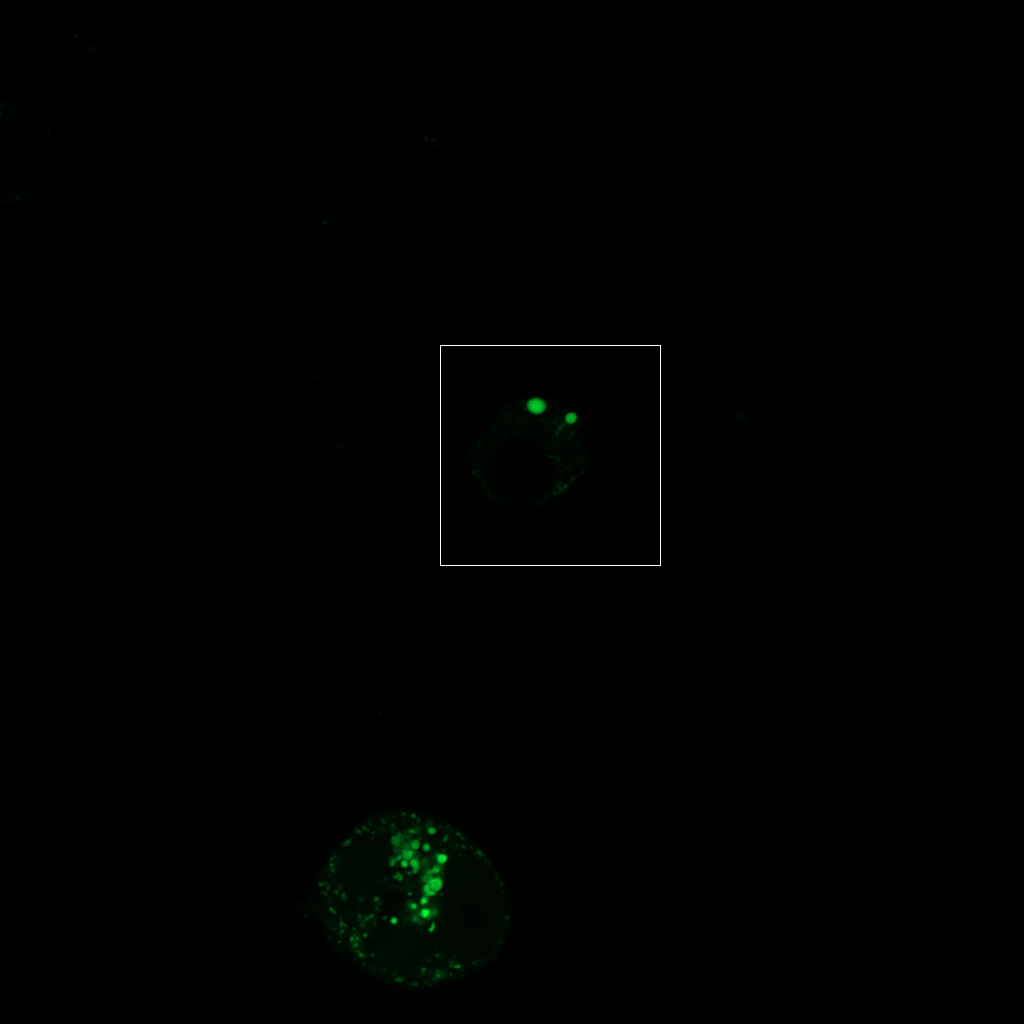

Supplement: Supplementary file 6 — Source data Fig. 3 [file 44318_2024_120_MOESM6_ESM.zip › Figure 3/3J/sgTRIM23/Pre.tif]

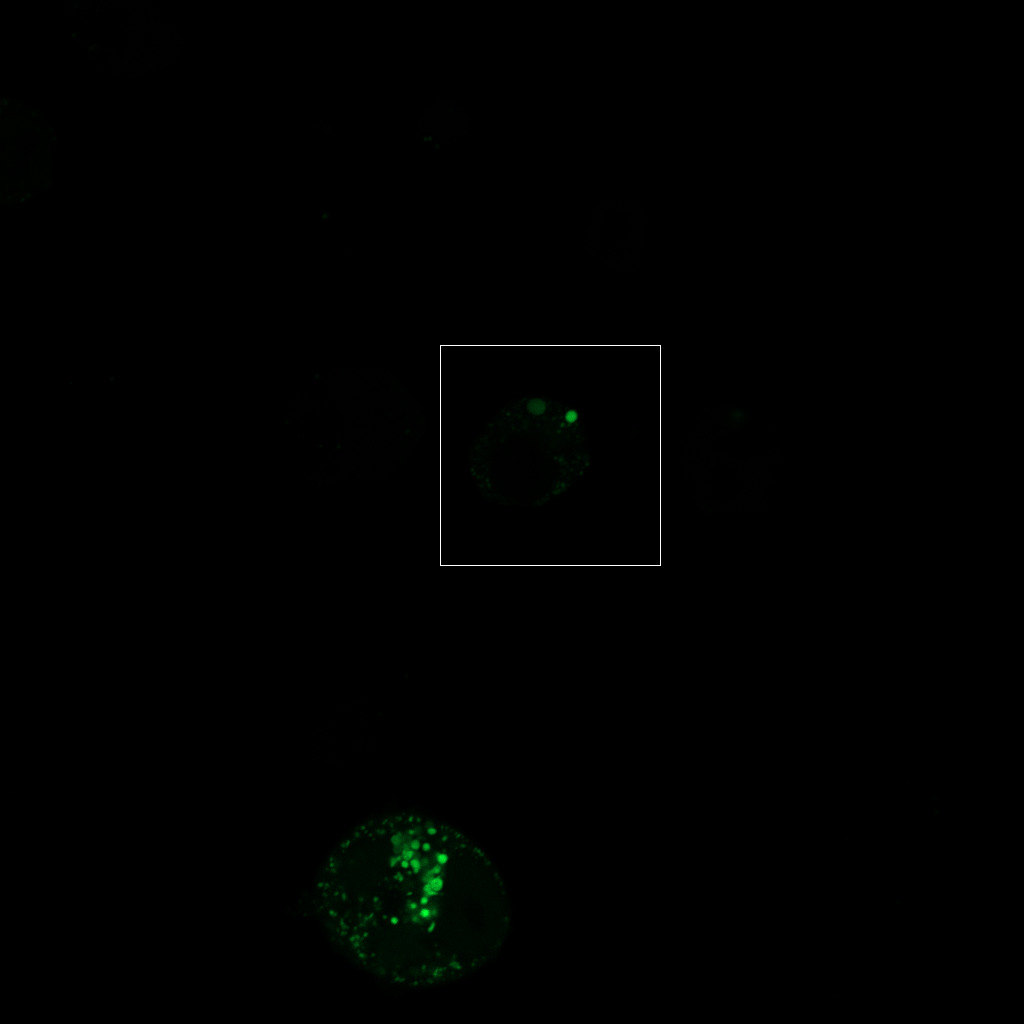

Supplement: Supplementary file 6 — Source data Fig. 3 [file 44318_2024_120_MOESM6_ESM.zip › Figure 3/3J/sgTRIM23/60s.tif]

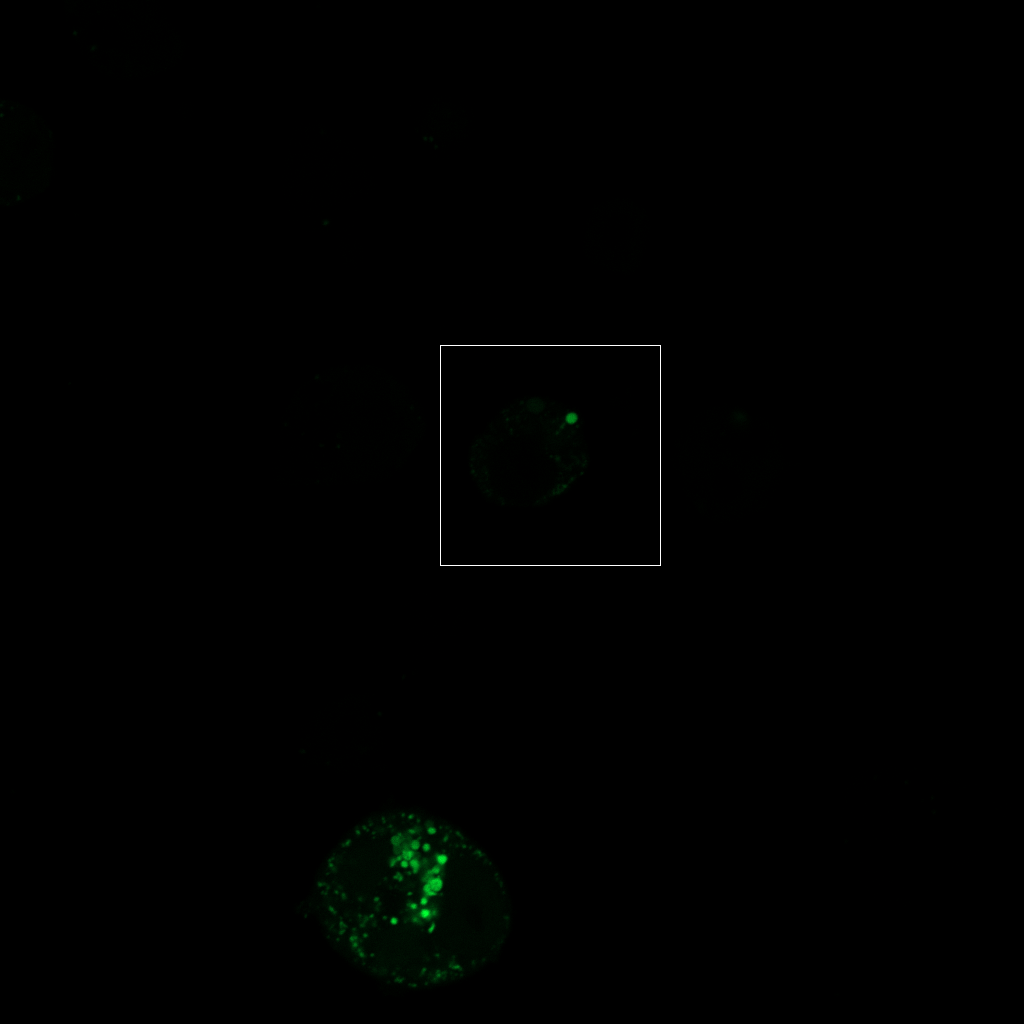

Supplement: Supplementary file 6 — Source data Fig. 3 [file 44318_2024_120_MOESM6_ESM.zip › Figure 3/3J/sgTRIM23/Bleaching.tif]

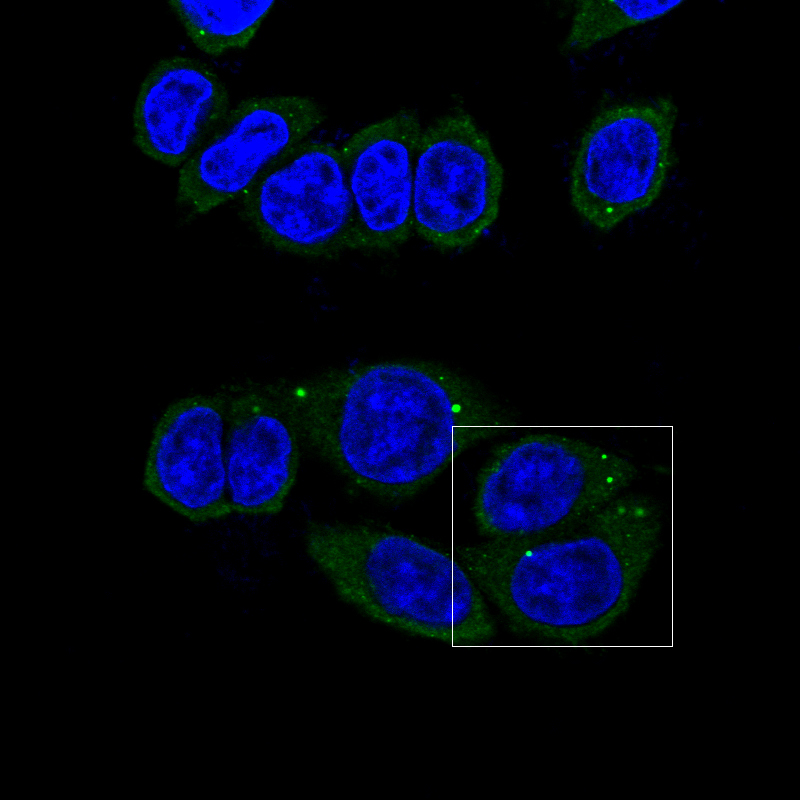

Supplement: Supplementary file 6 — Source data Fig. 3 [file 44318_2024_120_MOESM6_ESM.zip › Figure 3/3F/sgCtrl/Vector.tif]

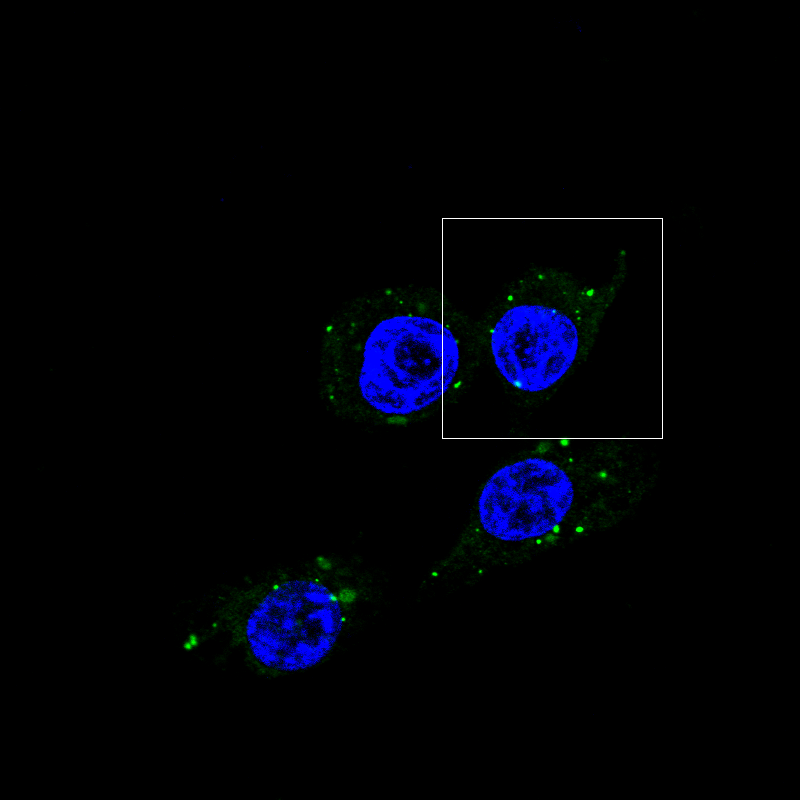

Supplement: Supplementary file 6 — Source data Fig. 3 [file 44318_2024_120_MOESM6_ESM.zip › Figure 3/3F/sgCtrl/Ub.tif]

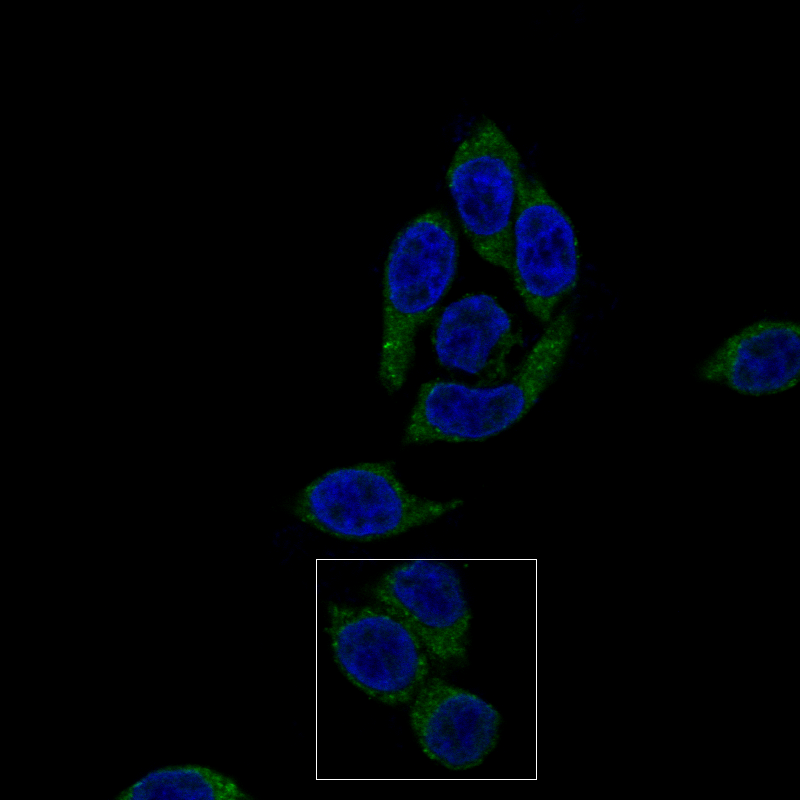

Supplement: Supplementary file 6 — Source data Fig. 3 [file 44318_2024_120_MOESM6_ESM.zip › Figure 3/3F/sgTRIM23/Vector.tif]

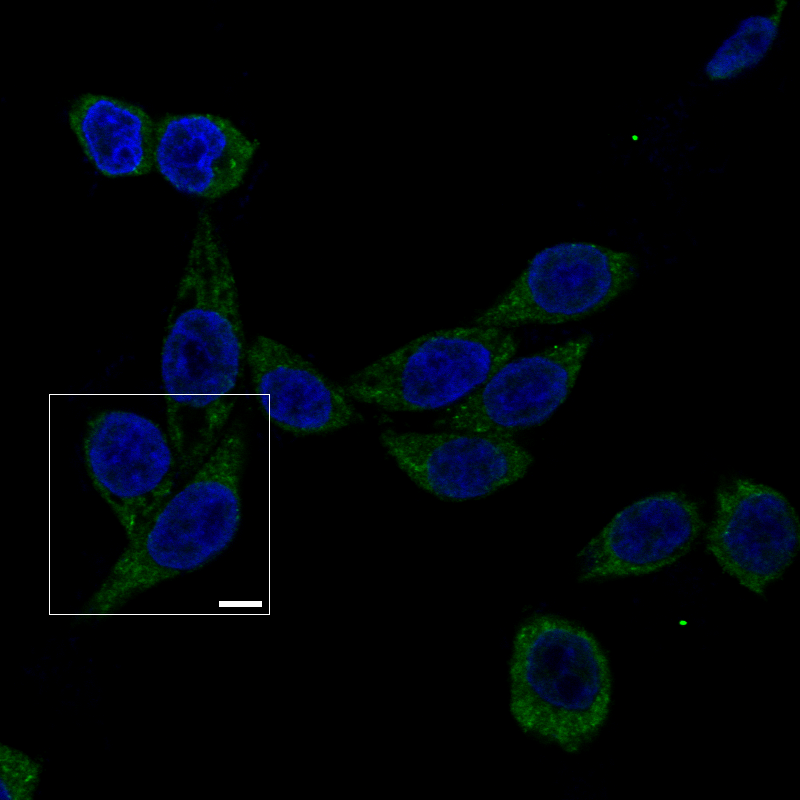

Supplement: Supplementary file 6 — Source data Fig. 3 [file 44318_2024_120_MOESM6_ESM.zip › Figure 3/3F/sgTRIM23/Ub.tif]

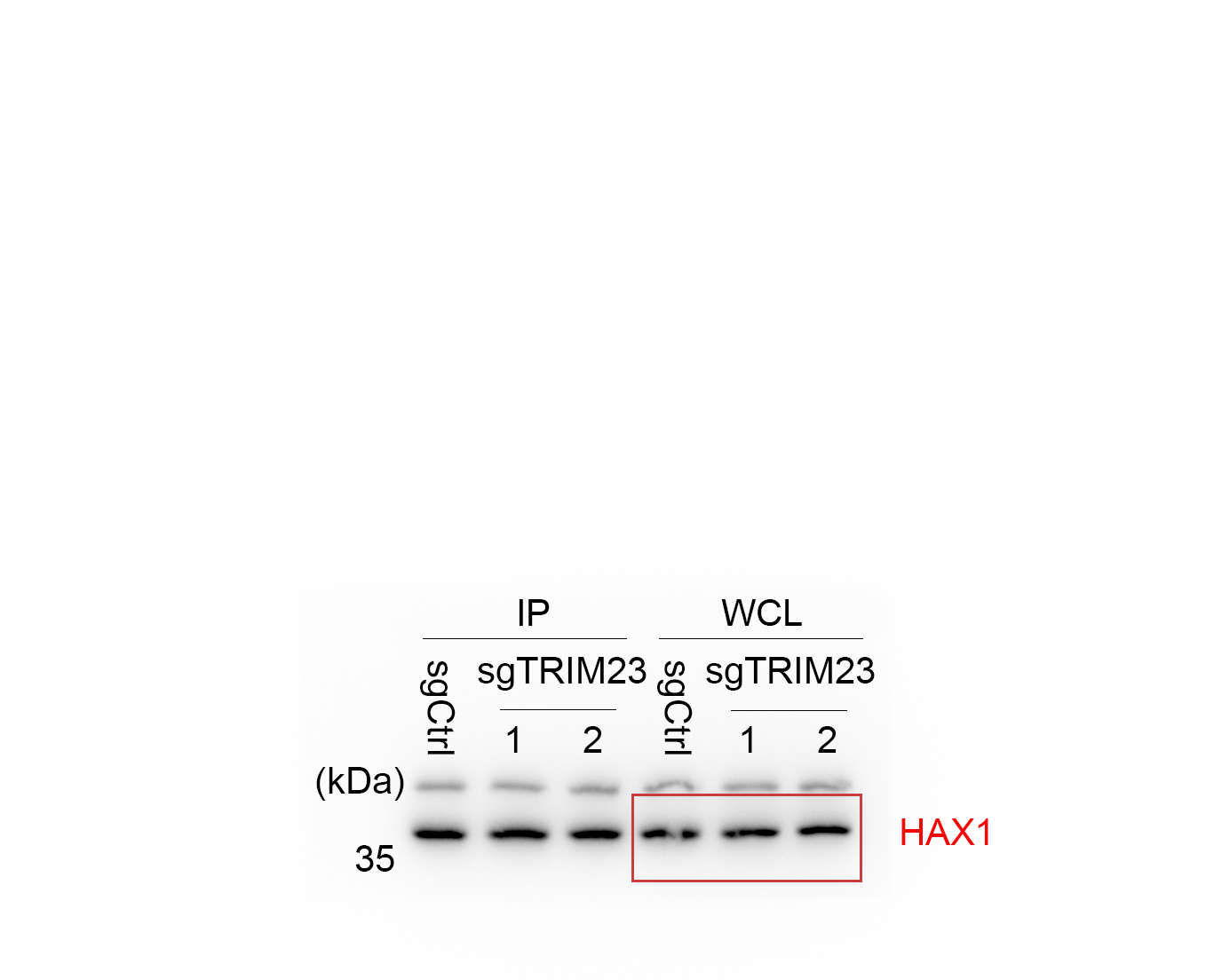

Supplement: Supplementary file 7 — Source data Fig. 4 [file 44318_2024_120_MOESM7_ESM.zip › Figure 4/4E/WCL/western-HAX1.Tif]

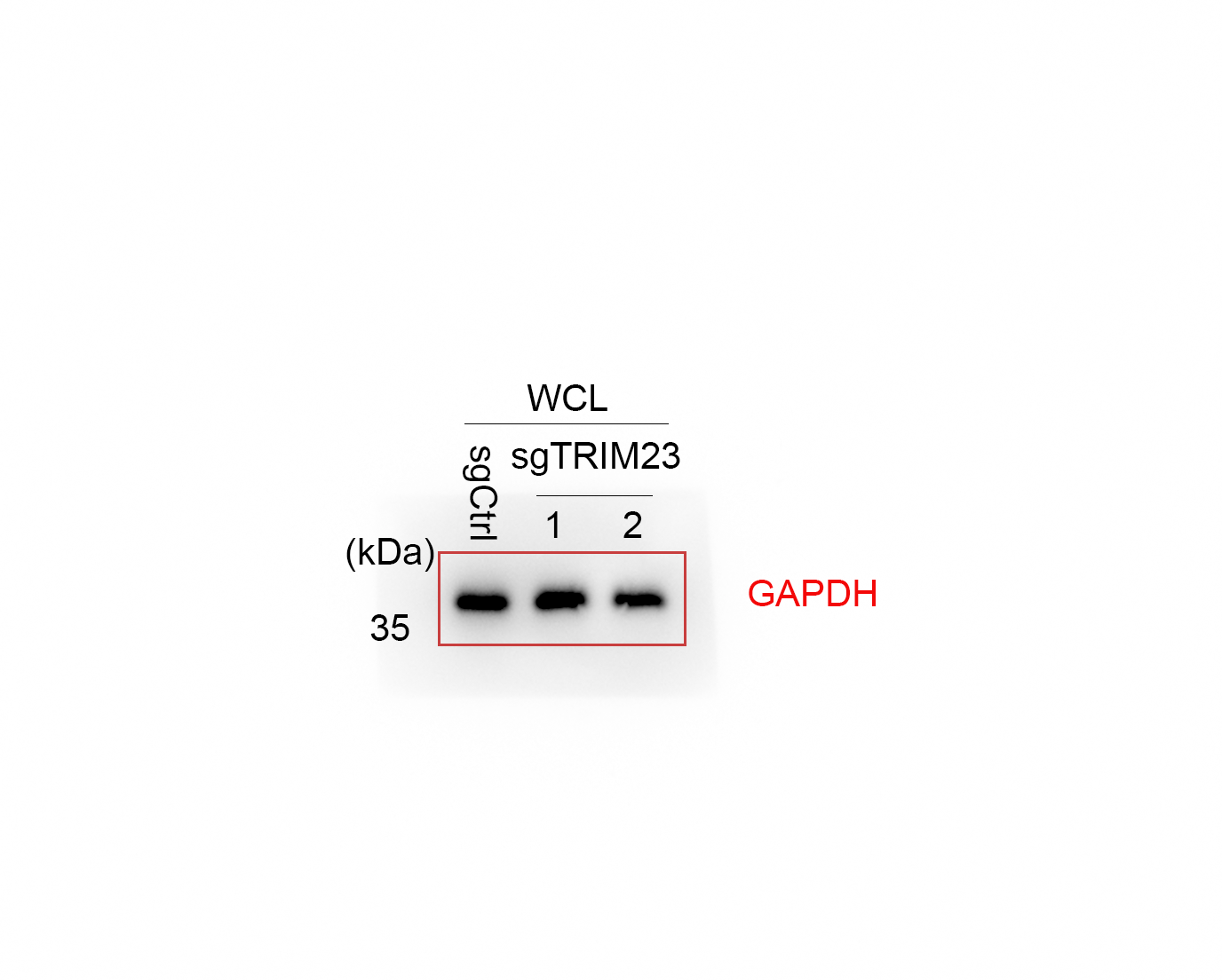

Supplement: Supplementary file 7 — Source data Fig. 4 [file 44318_2024_120_MOESM7_ESM.zip › Figure 4/4E/WCL/western-GAPDH.Tif]

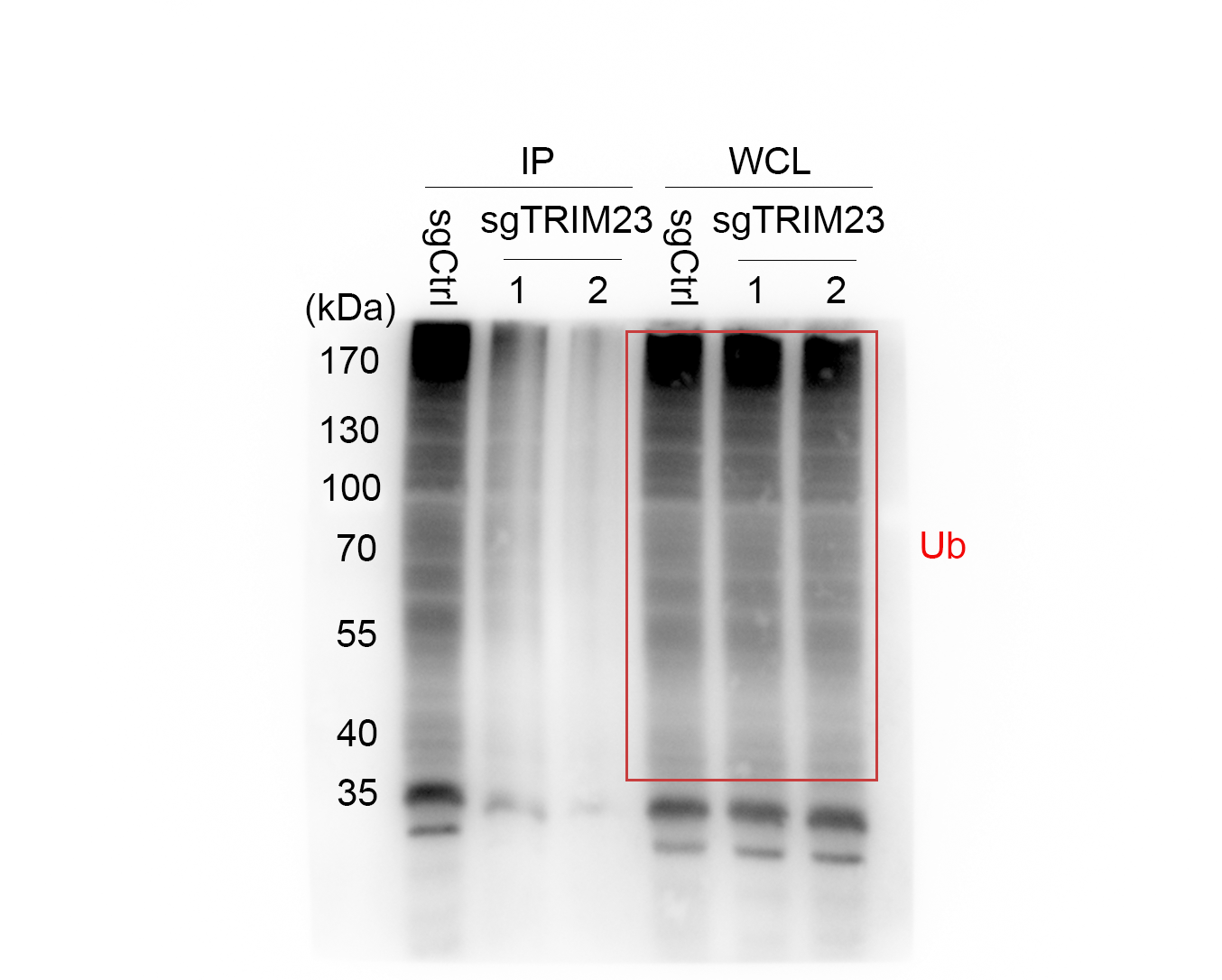

Supplement: Supplementary file 7 — Source data Fig. 4 [file 44318_2024_120_MOESM7_ESM.zip › Figure 4/4E/WCL/western-Ub-proteins.Tif]

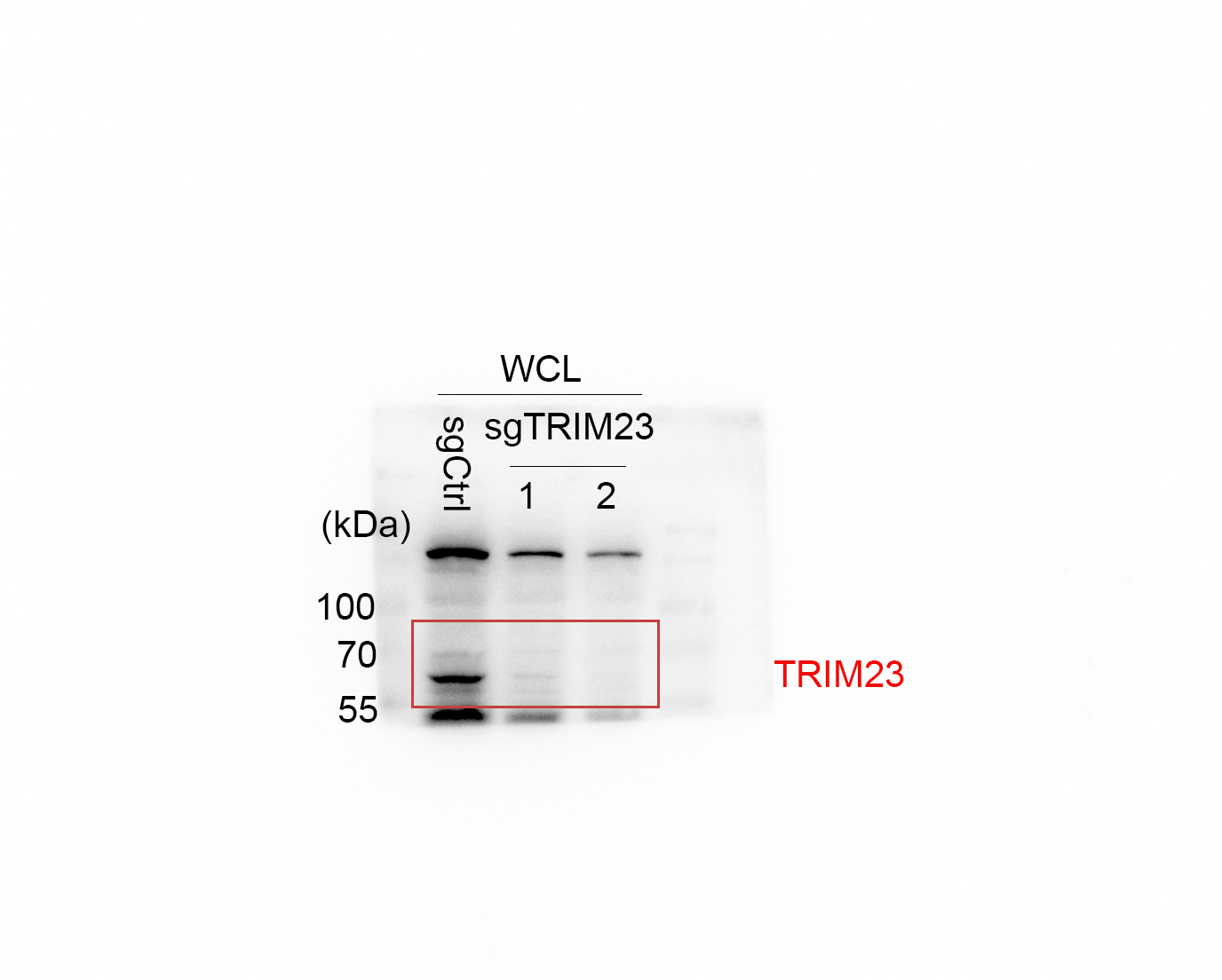

Supplement: Supplementary file 7 — Source data Fig. 4 [file 44318_2024_120_MOESM7_ESM.zip › Figure 4/4E/WCL/western-TRIM23.Tif]

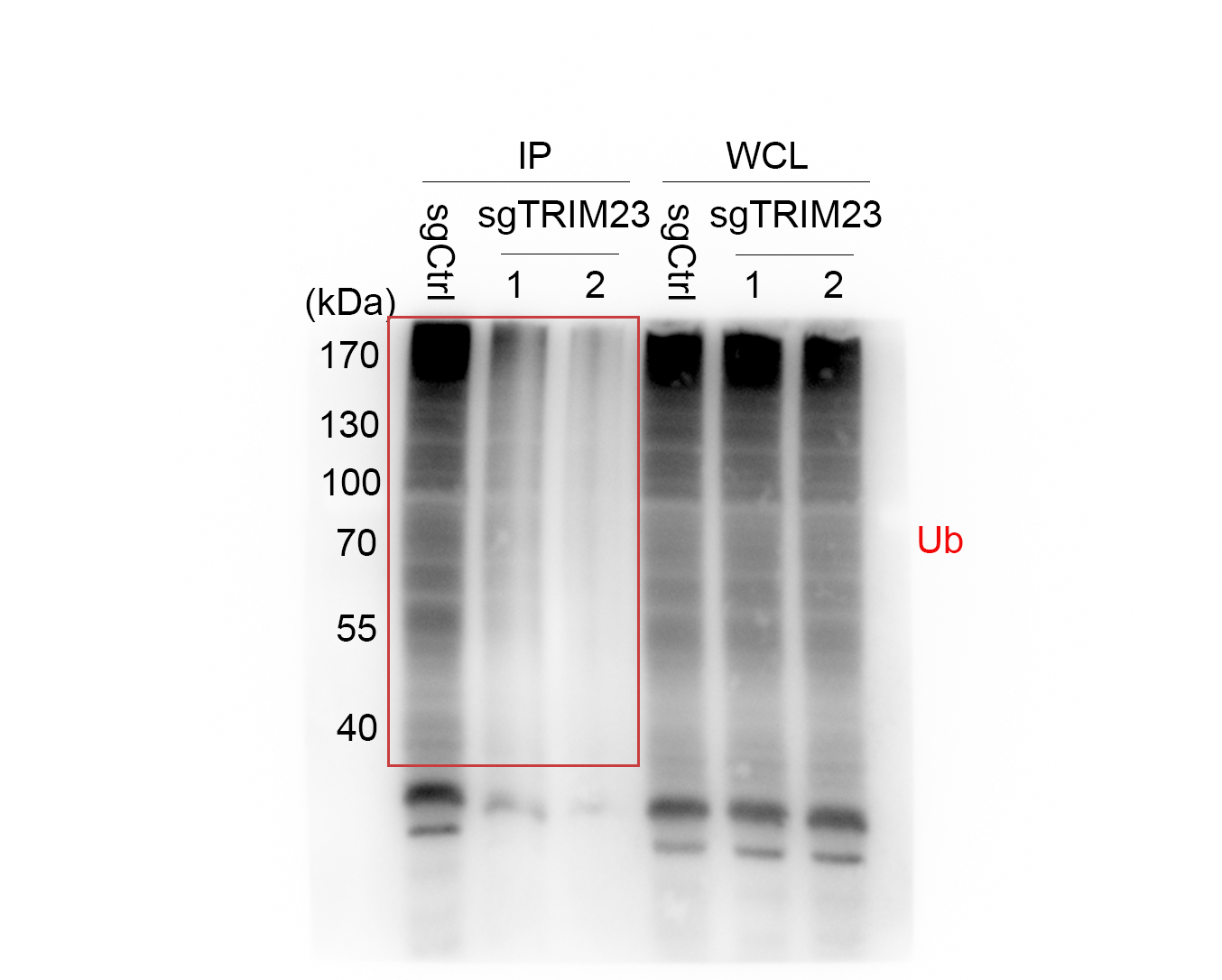

Supplement: Supplementary file 7 — Source data Fig. 4 [file 44318_2024_120_MOESM7_ESM.zip › Figure 4/4E/IP/western-HAX1-Ub.Tif]

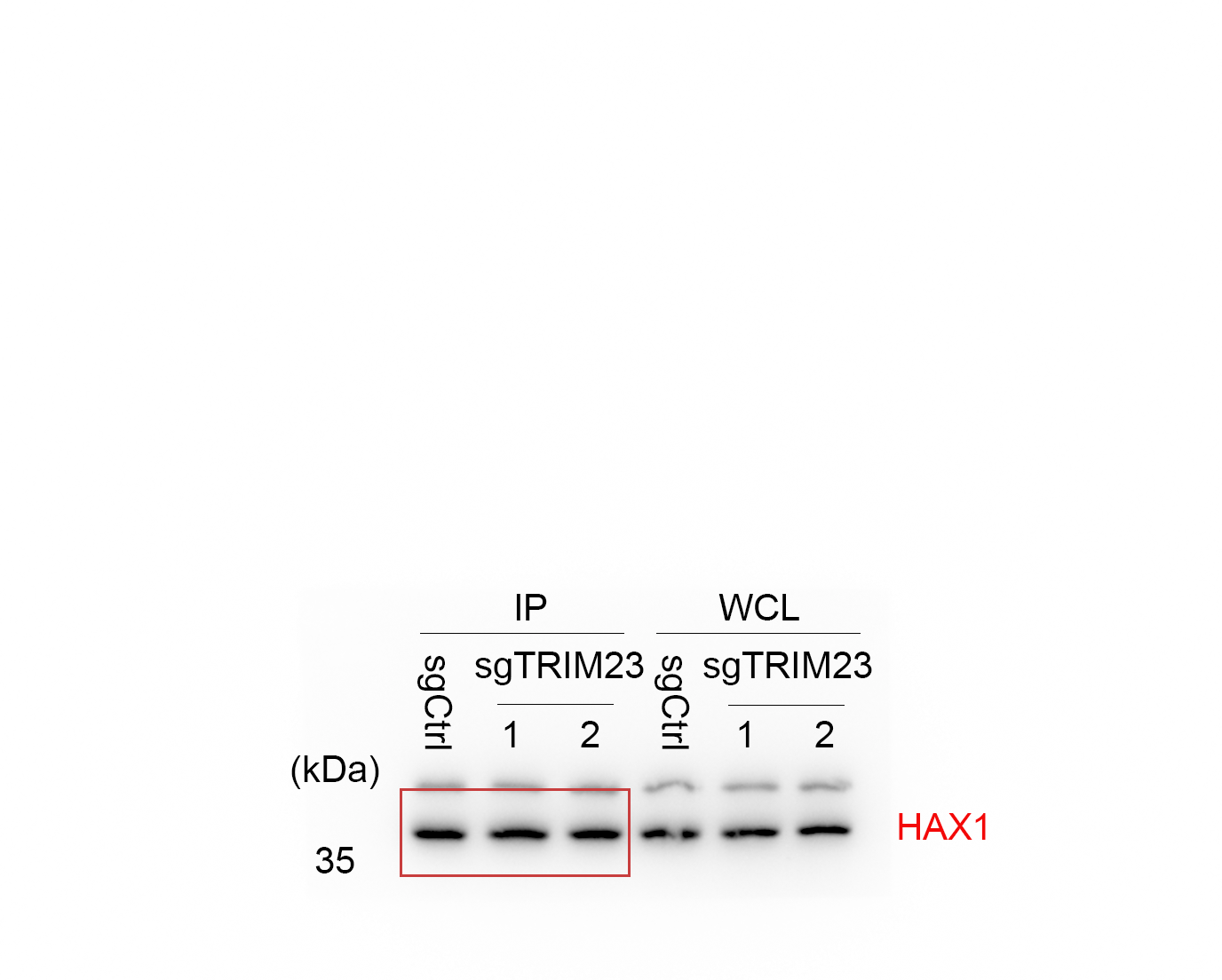

Supplement: Supplementary file 7 — Source data Fig. 4 [file 44318_2024_120_MOESM7_ESM.zip › Figure 4/4E/IP/western-HAX1.Tif]

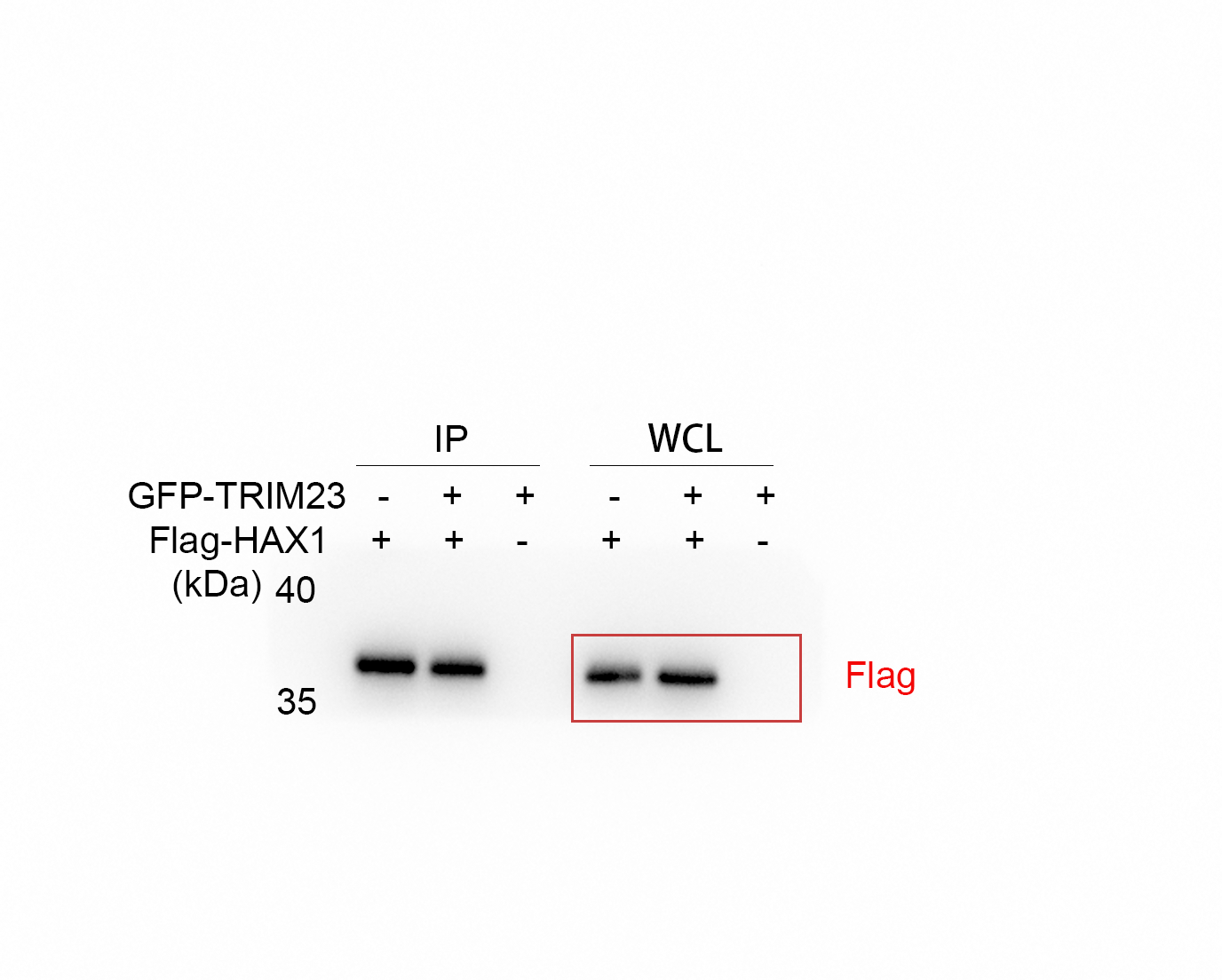

Supplement: Supplementary file 7 — Source data Fig. 4 [file 44318_2024_120_MOESM7_ESM.zip › Figure 4/4B/WCL/western-Flag-HAX1.Tif]

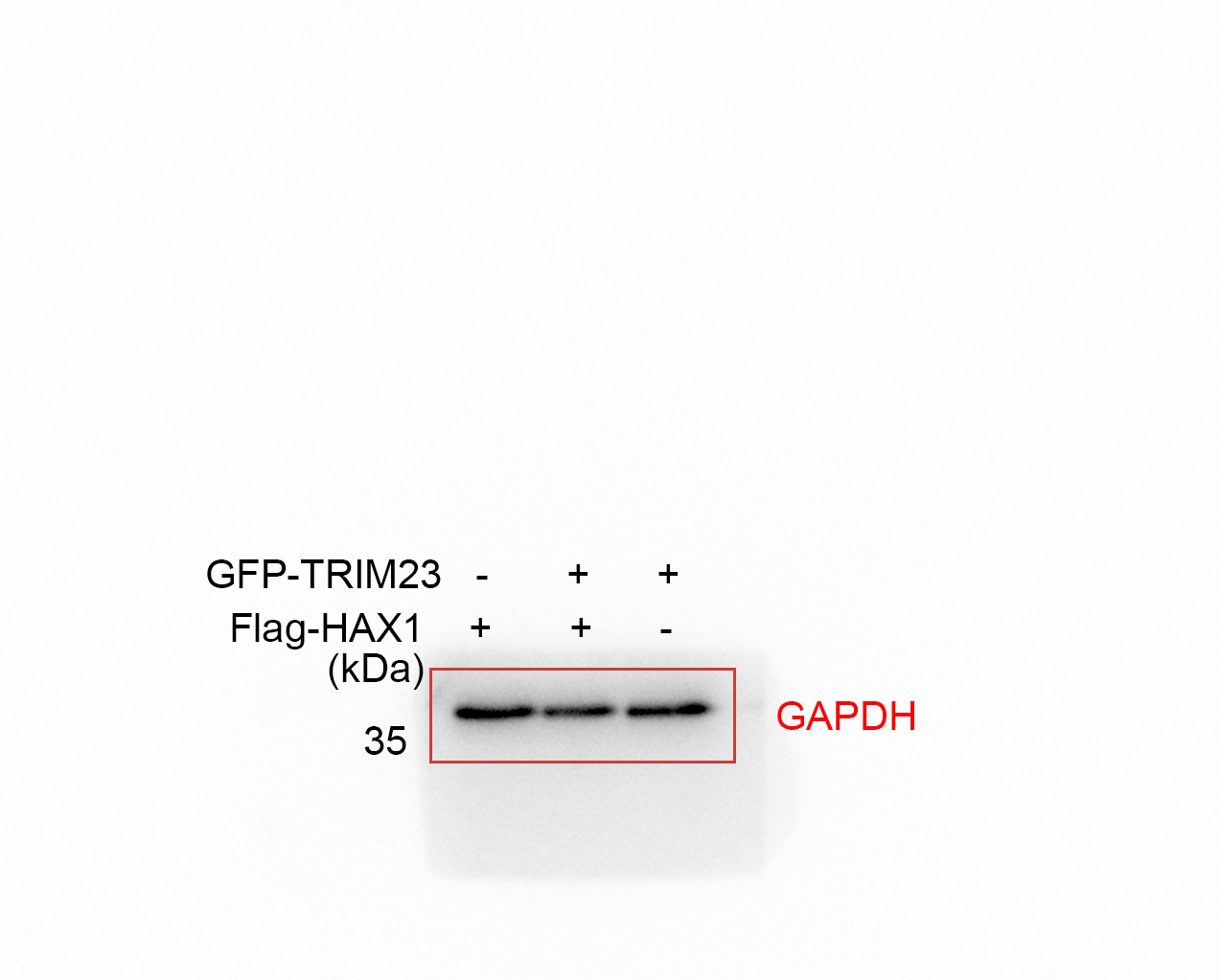

Supplement: Supplementary file 7 — Source data Fig. 4 [file 44318_2024_120_MOESM7_ESM.zip › Figure 4/4B/WCL/western-GAPDH.Tif]

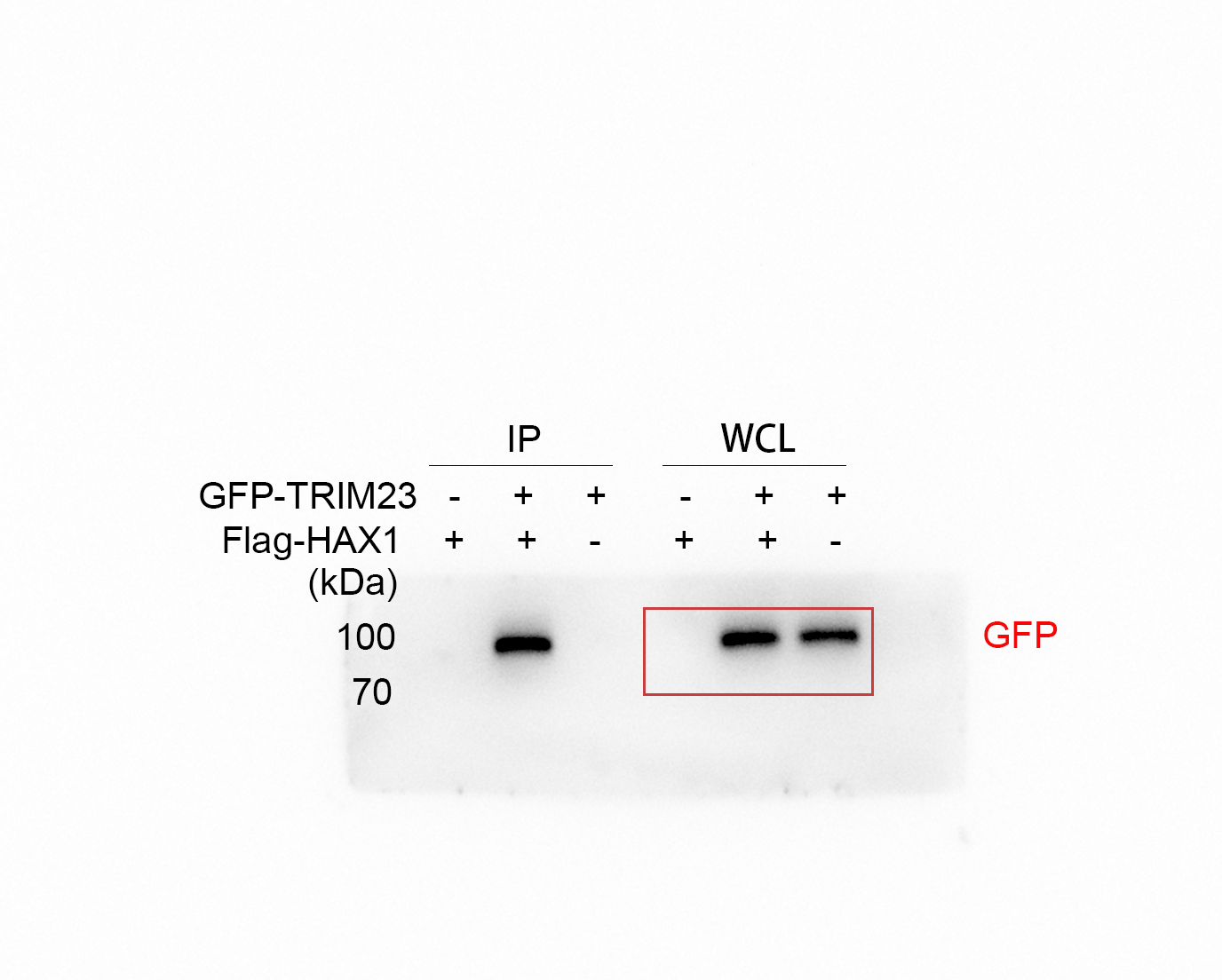

Supplement: Supplementary file 7 — Source data Fig. 4 [file 44318_2024_120_MOESM7_ESM.zip › Figure 4/4B/WCL/western-GFP-TRIM23.Tif]

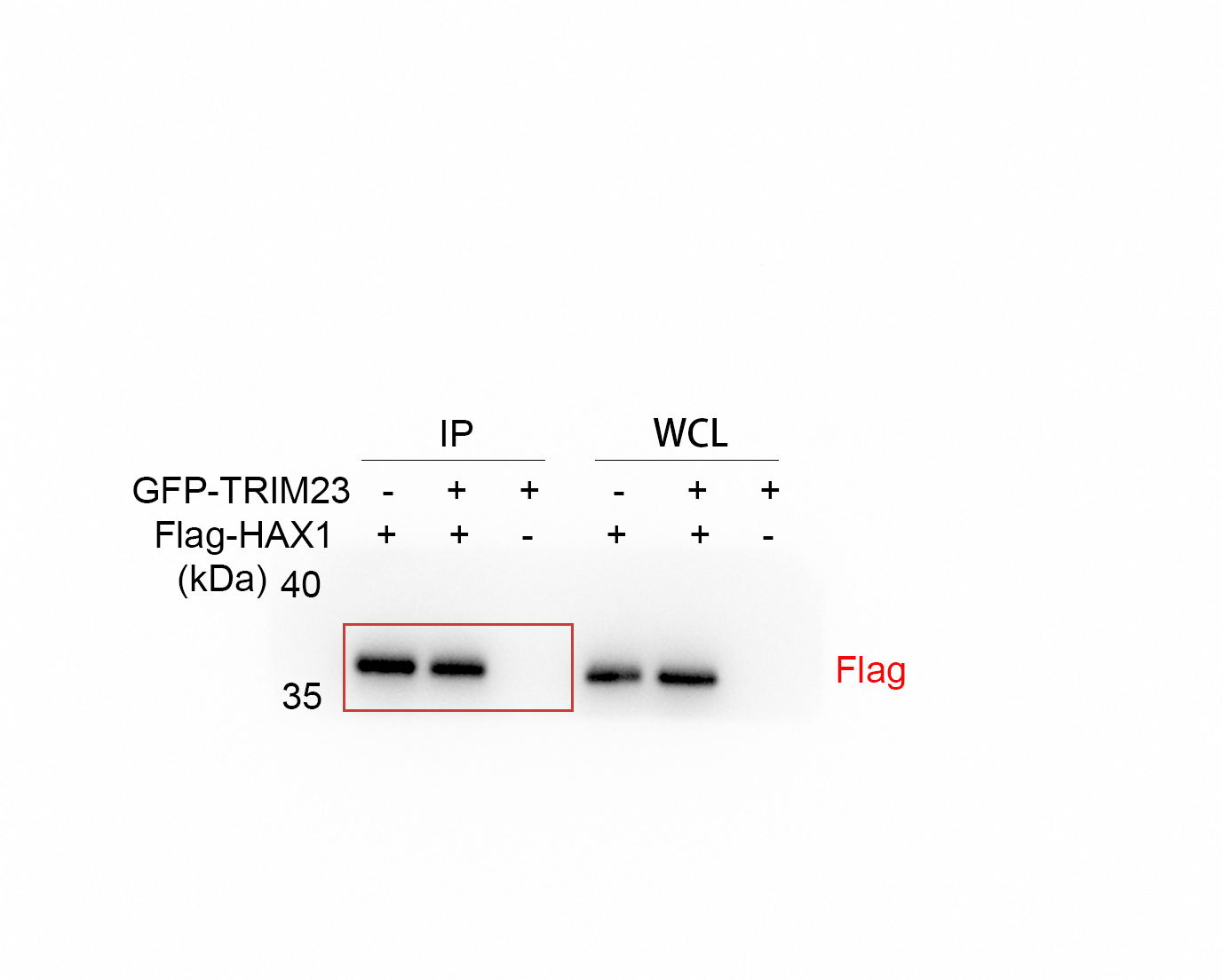

Supplement: Supplementary file 7 — Source data Fig. 4 [file 44318_2024_120_MOESM7_ESM.zip › Figure 4/4B/IP/western-Flag-HAX1.Tif]

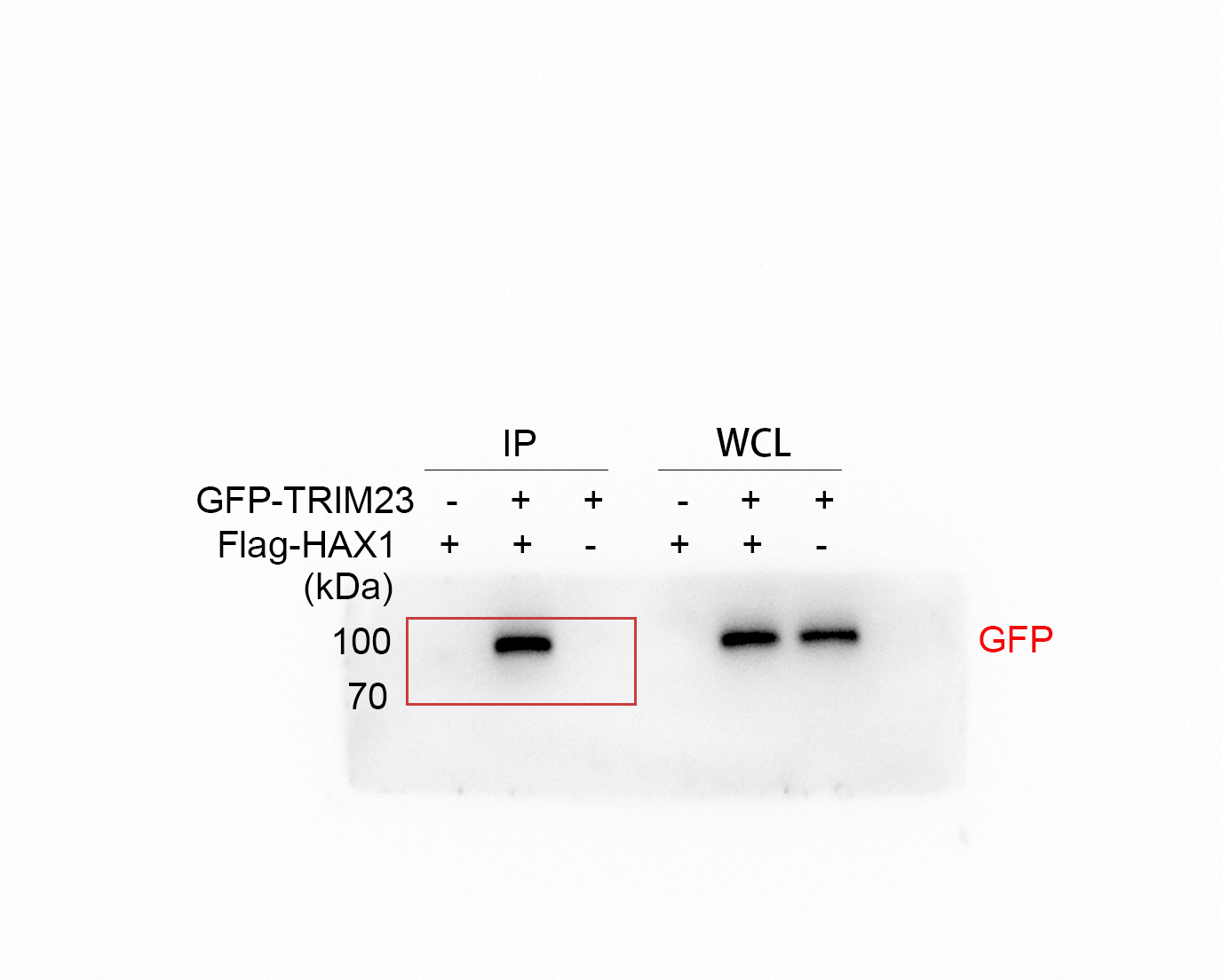

Supplement: Supplementary file 7 — Source data Fig. 4 [file 44318_2024_120_MOESM7_ESM.zip › Figure 4/4B/IP/western-GFP-TRIM23.Tif]

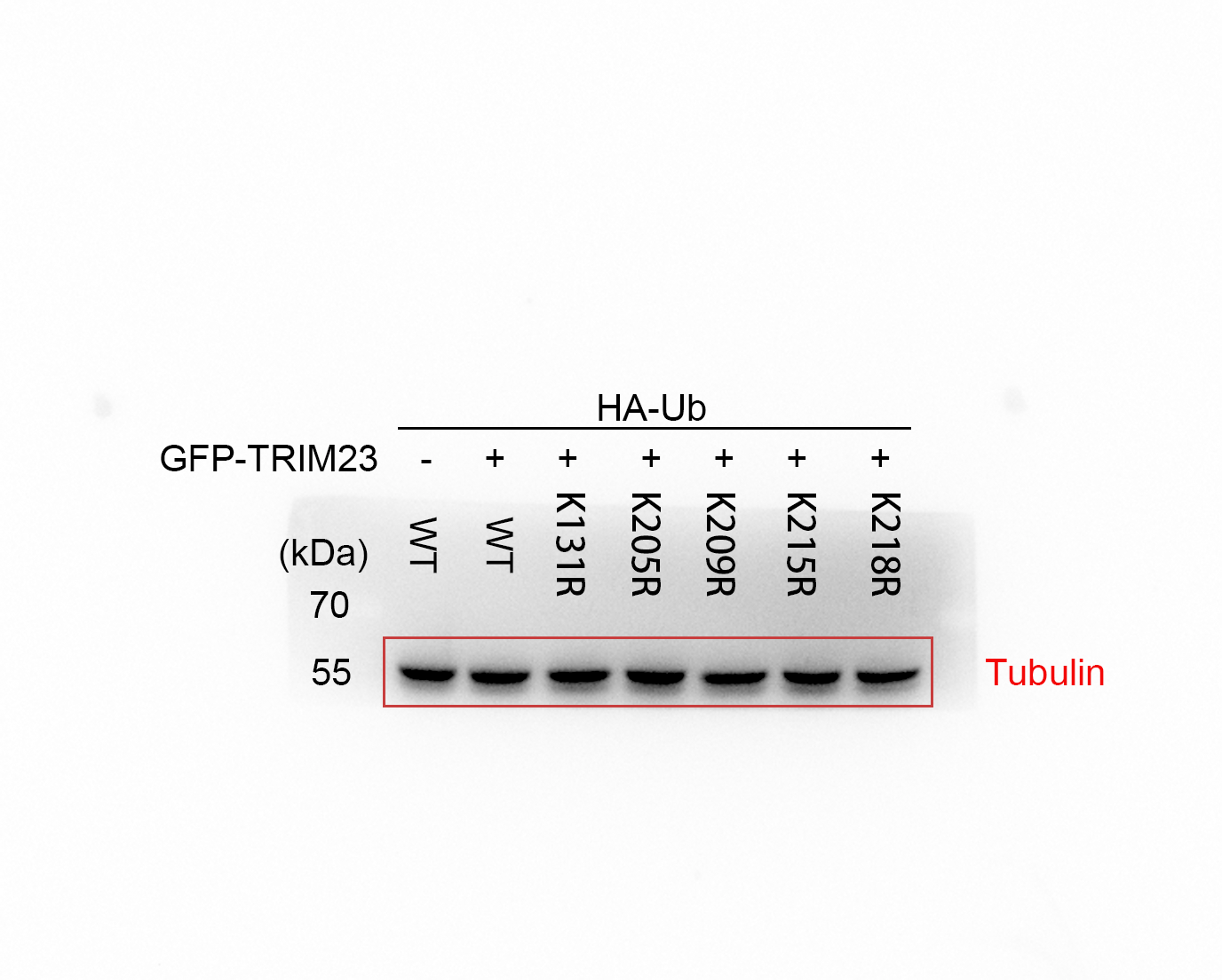

Supplement: Supplementary file 7 — Source data Fig. 4 [file 44318_2024_120_MOESM7_ESM.zip › Figure 4/4K/WCL/western-Tubulin.Tif]

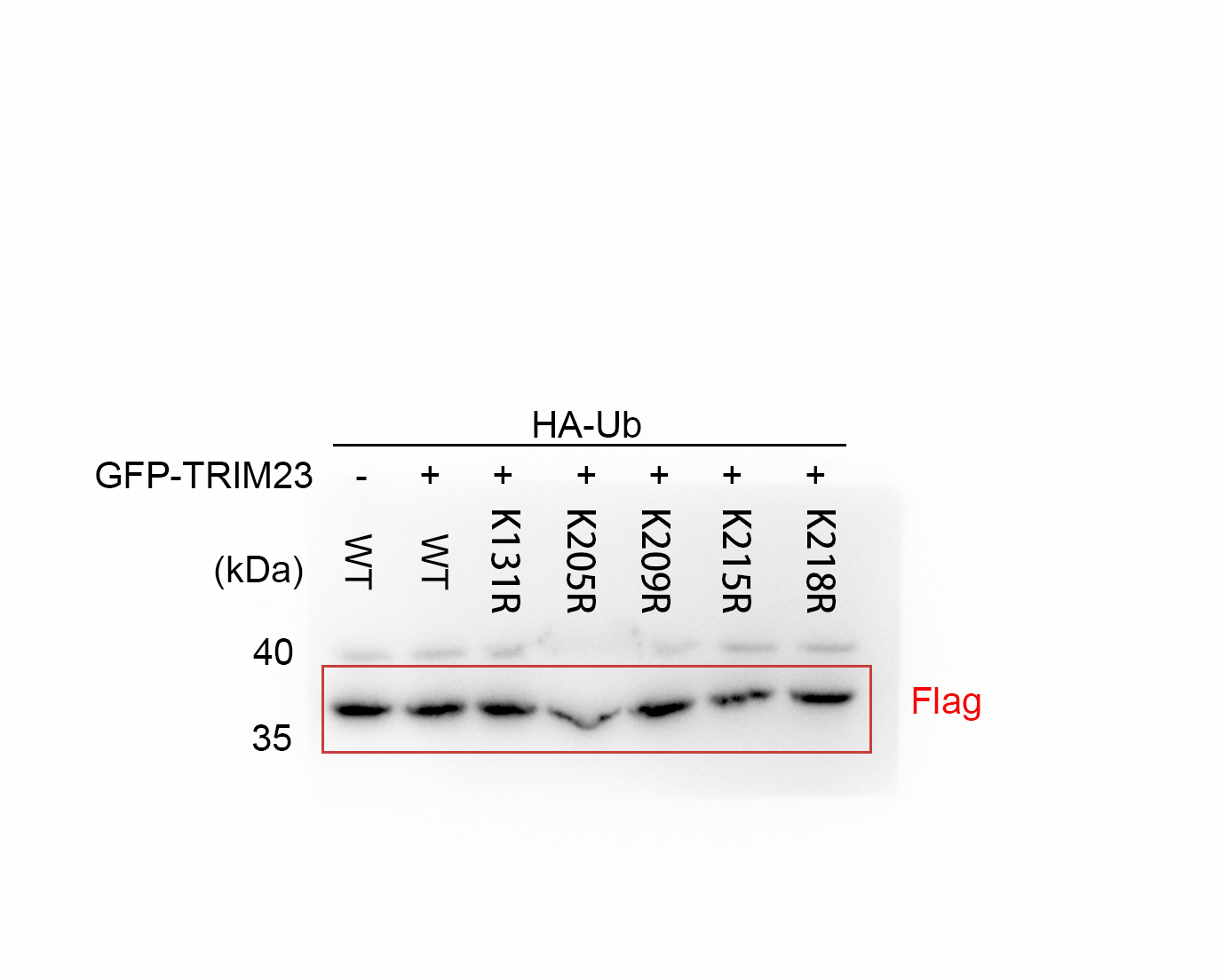

Supplement: Supplementary file 7 — Source data Fig. 4 [file 44318_2024_120_MOESM7_ESM.zip › Figure 4/4K/WCL/western-Flag-HAX1.Tif]

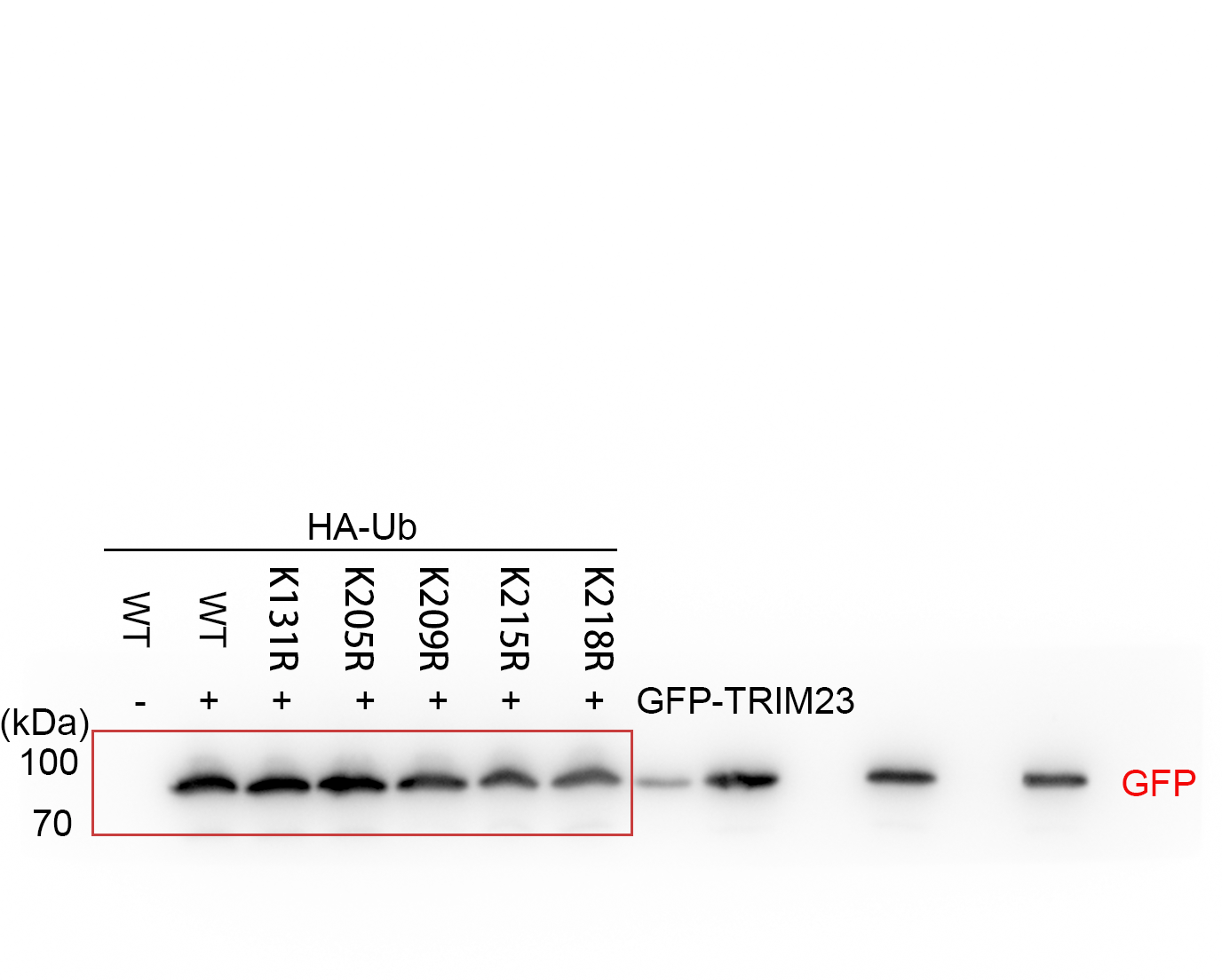

Supplement: Supplementary file 7 — Source data Fig. 4 [file 44318_2024_120_MOESM7_ESM.zip › Figure 4/4K/WCL/western-GFP-TRIM23.Tif]

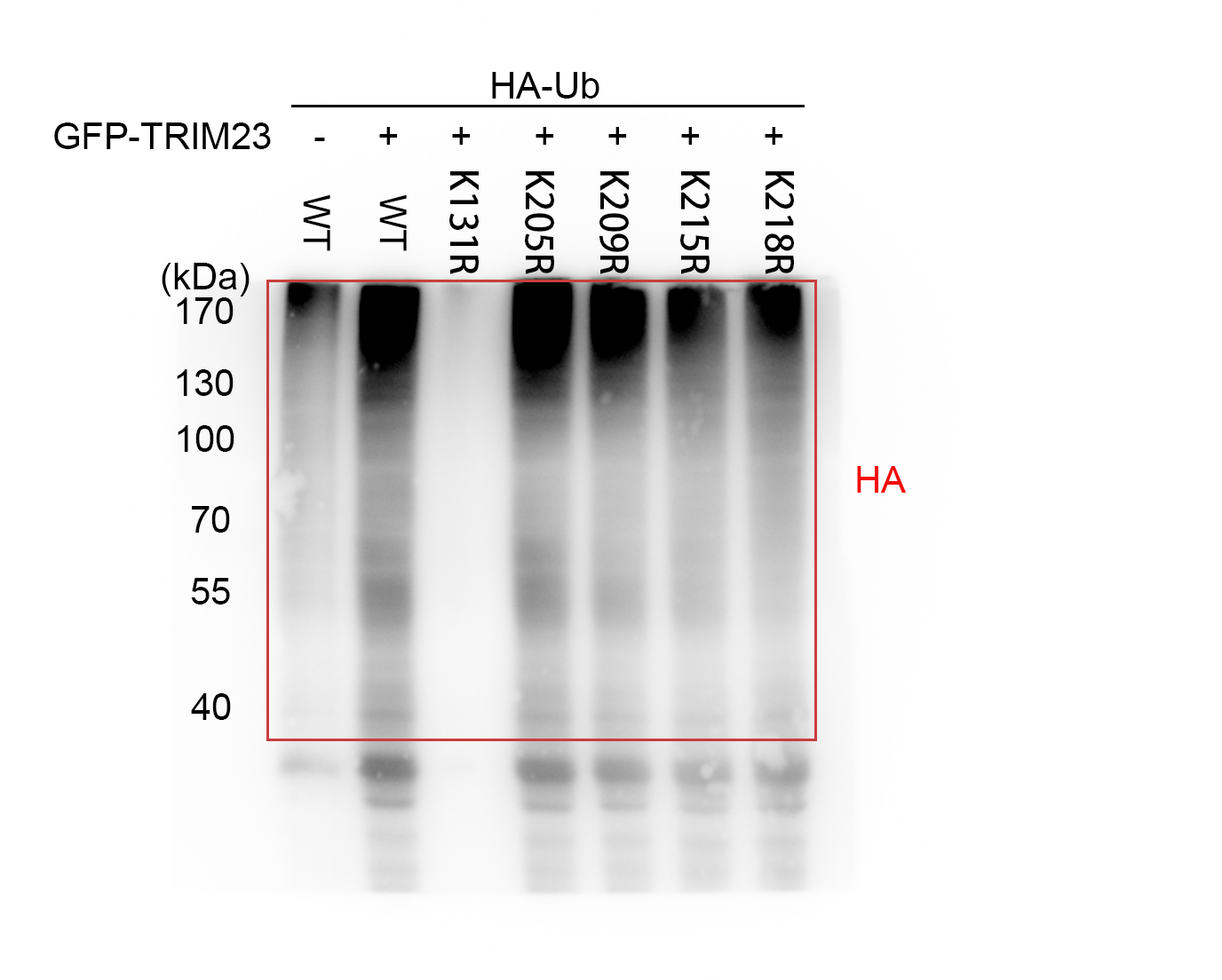

Supplement: Supplementary file 7 — Source data Fig. 4 [file 44318_2024_120_MOESM7_ESM.zip › Figure 4/4K/IP/western-Flag-HAX1-Ub.Tif]

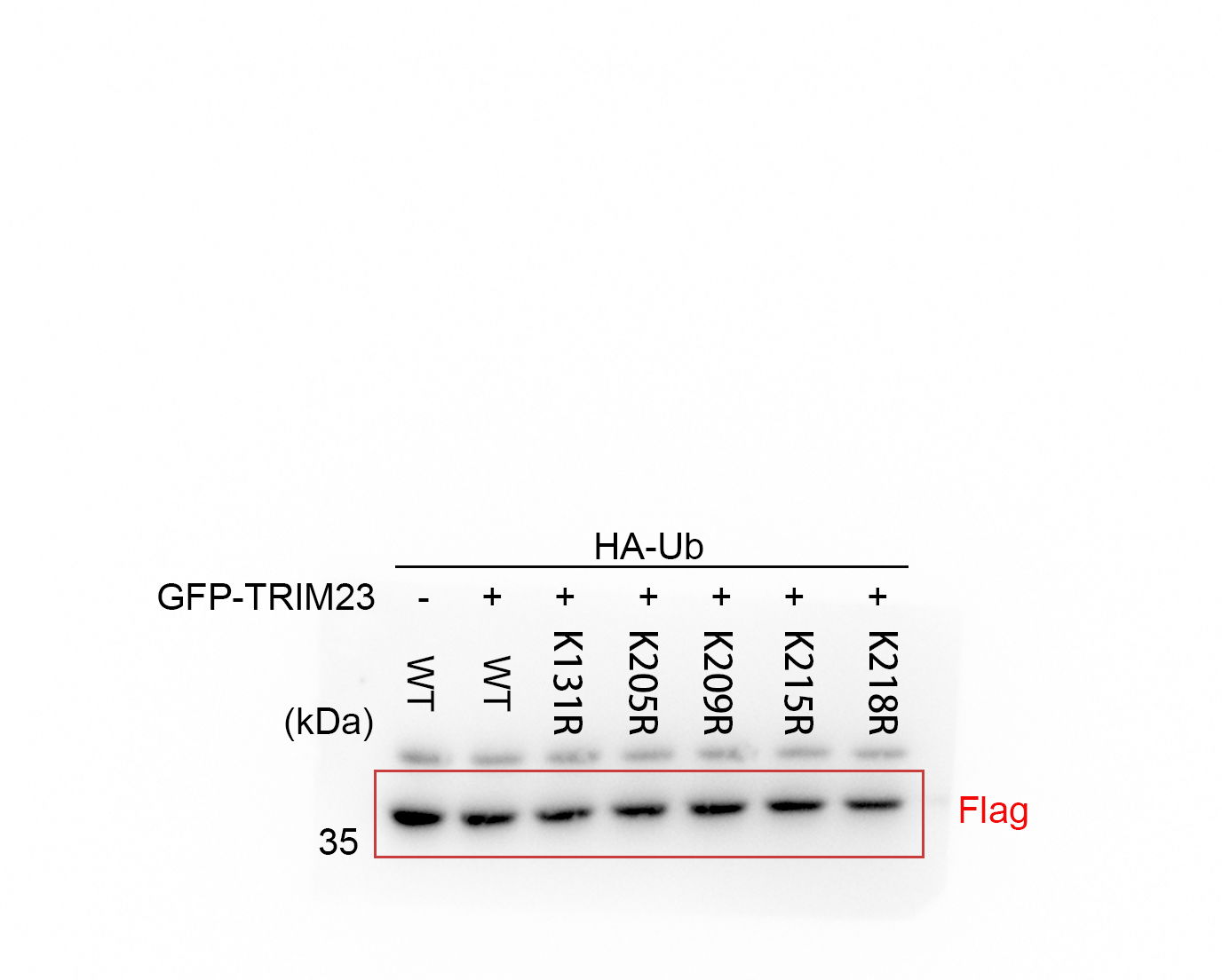

Supplement: Supplementary file 7 — Source data Fig. 4 [file 44318_2024_120_MOESM7_ESM.zip › Figure 4/4K/IP/western-Flag-HAX1.Tif]

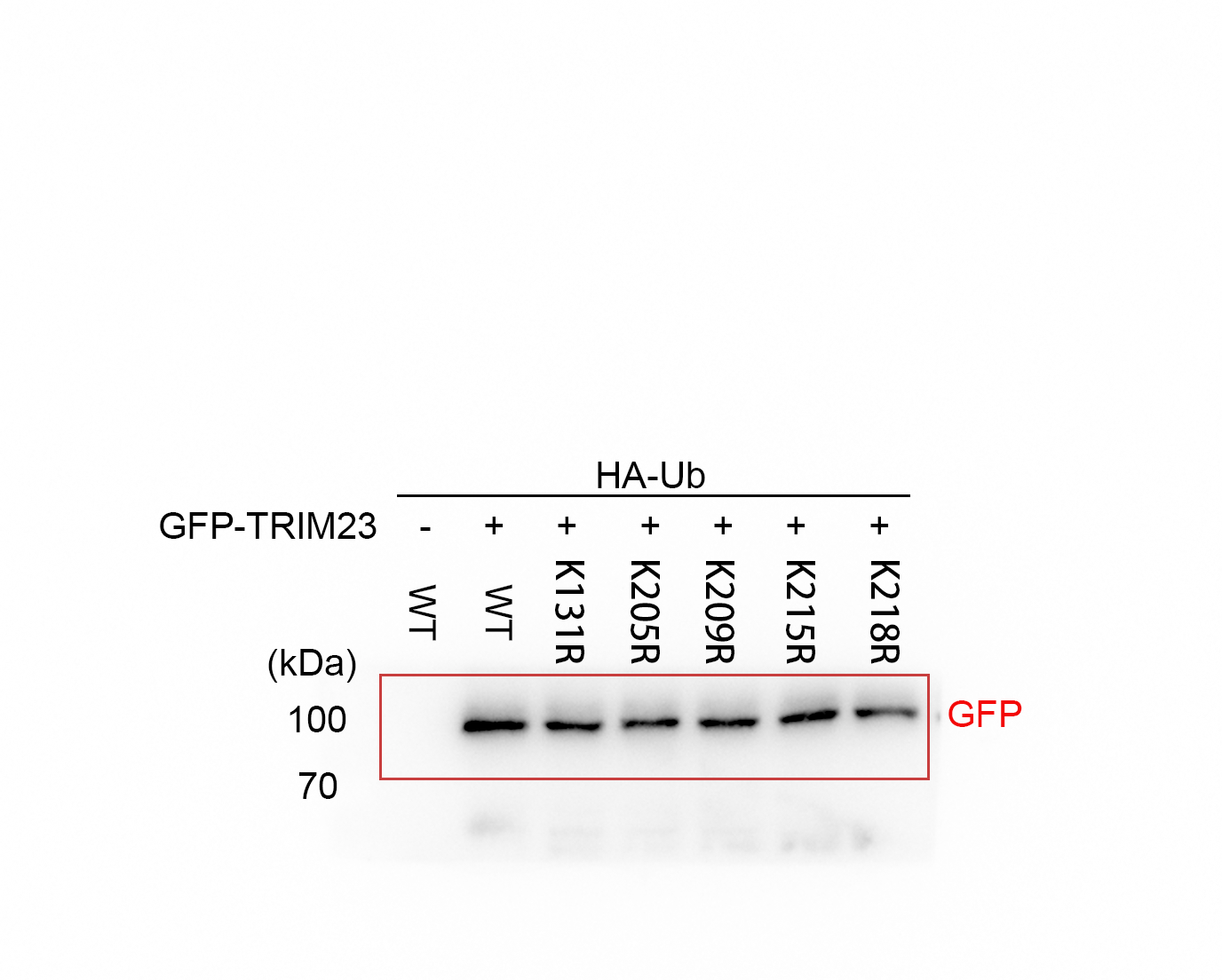

Supplement: Supplementary file 7 — Source data Fig. 4 [file 44318_2024_120_MOESM7_ESM.zip › Figure 4/4K/IP/western-GFP-TRIM23.Tif]

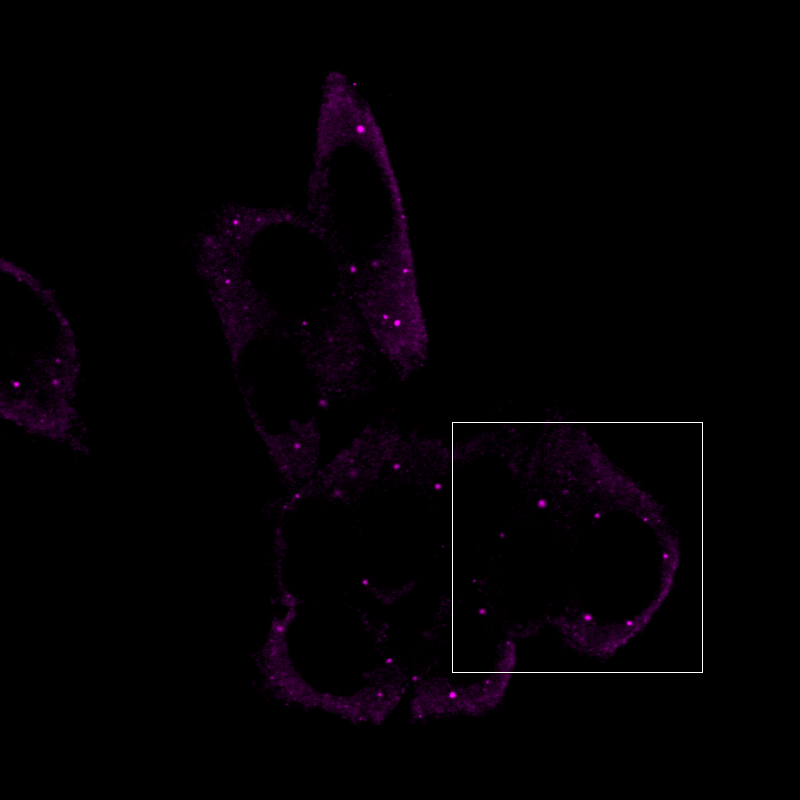

Supplement: Supplementary file 7 — Source data Fig. 4 [file 44318_2024_120_MOESM7_ESM.zip › Figure 4/4C/Below/mCherry-HAX1.tif]

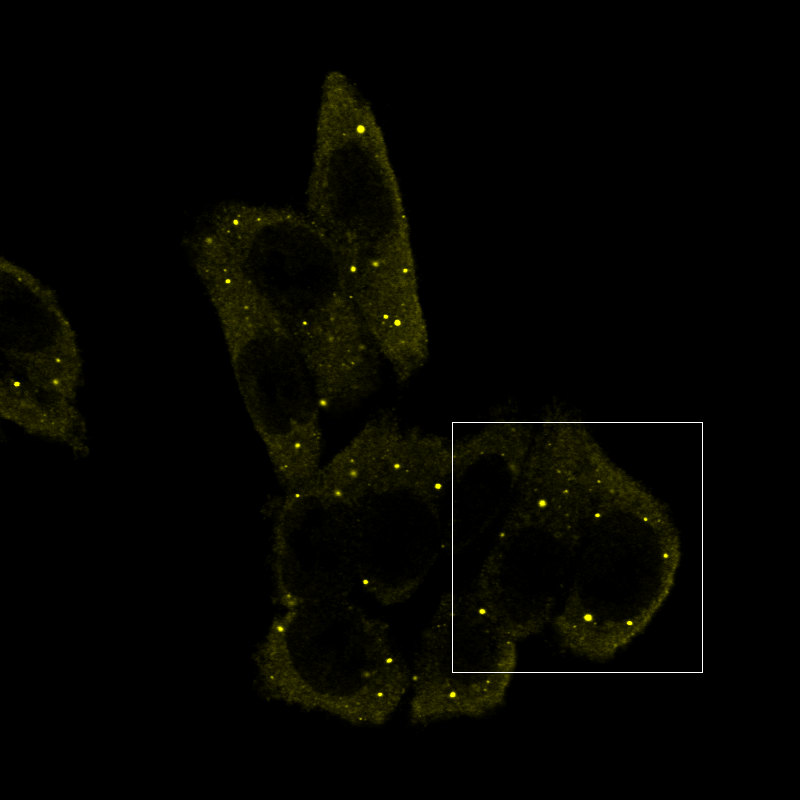

Supplement: Supplementary file 7 — Source data Fig. 4 [file 44318_2024_120_MOESM7_ESM.zip › Figure 4/4C/Below/GFP-LSM14A.tif]

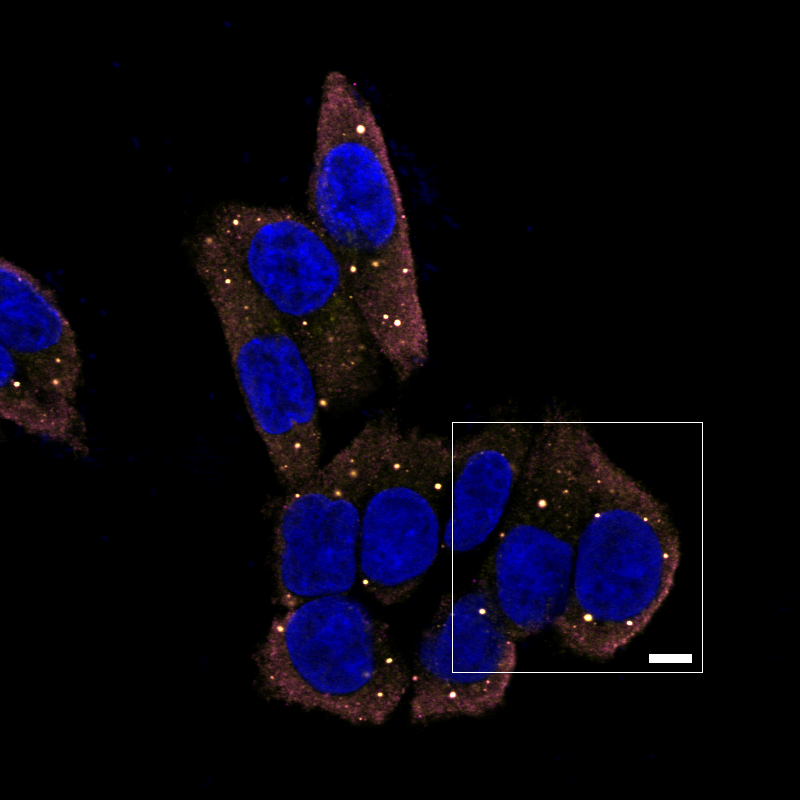

Supplement: Supplementary file 7 — Source data Fig. 4 [file 44318_2024_120_MOESM7_ESM.zip › Figure 4/4C/Below/Merge.tif]

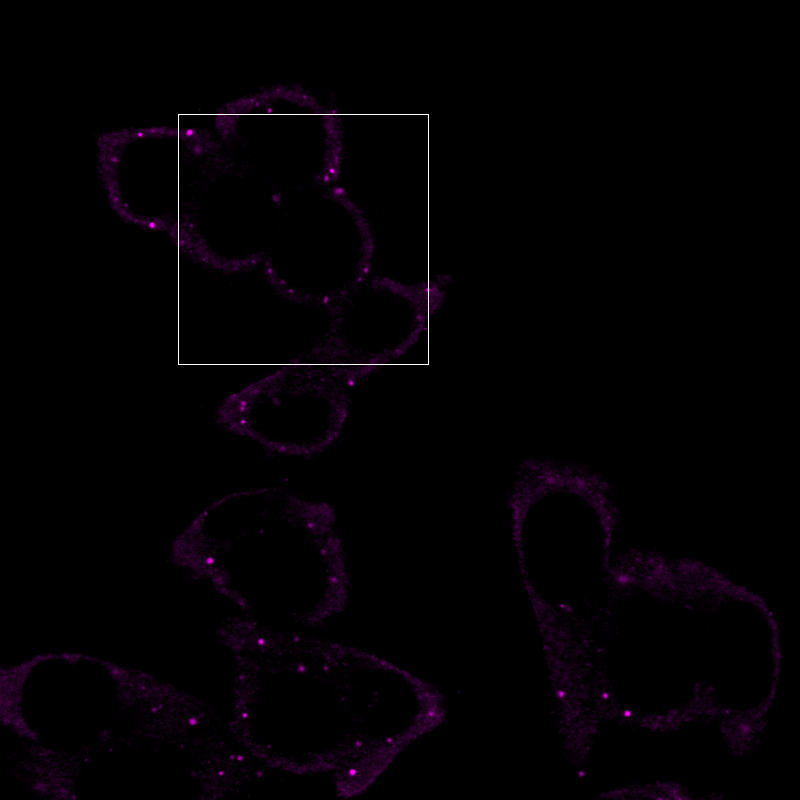

Supplement: Supplementary file 7 — Source data Fig. 4 [file 44318_2024_120_MOESM7_ESM.zip › Figure 4/4C/Above/mCherry-HAX1.tif]

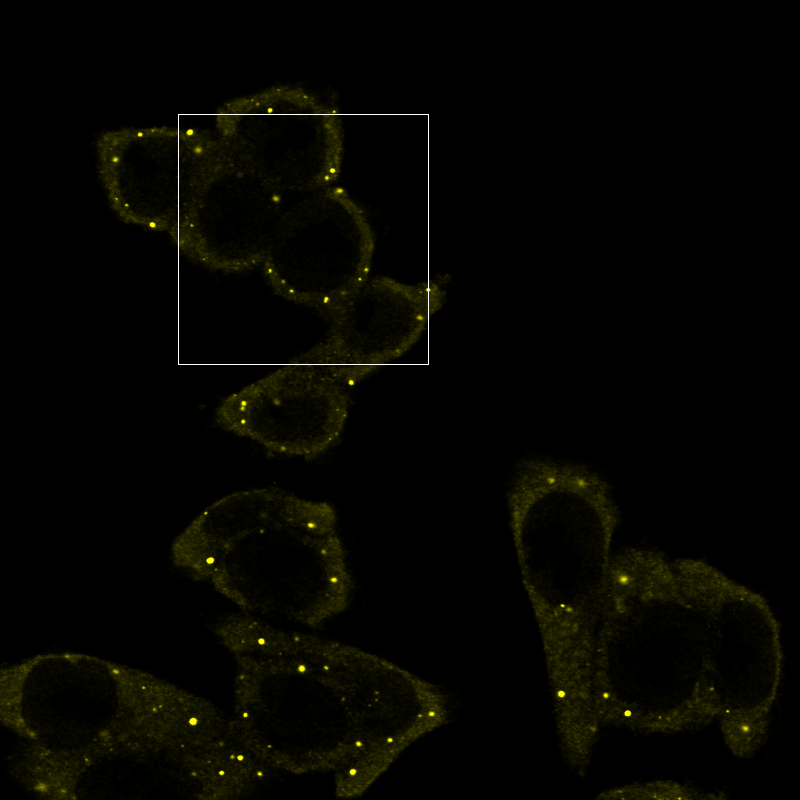

Supplement: Supplementary file 7 — Source data Fig. 4 [file 44318_2024_120_MOESM7_ESM.zip › Figure 4/4C/Above/GFP-TRIM23.tif]

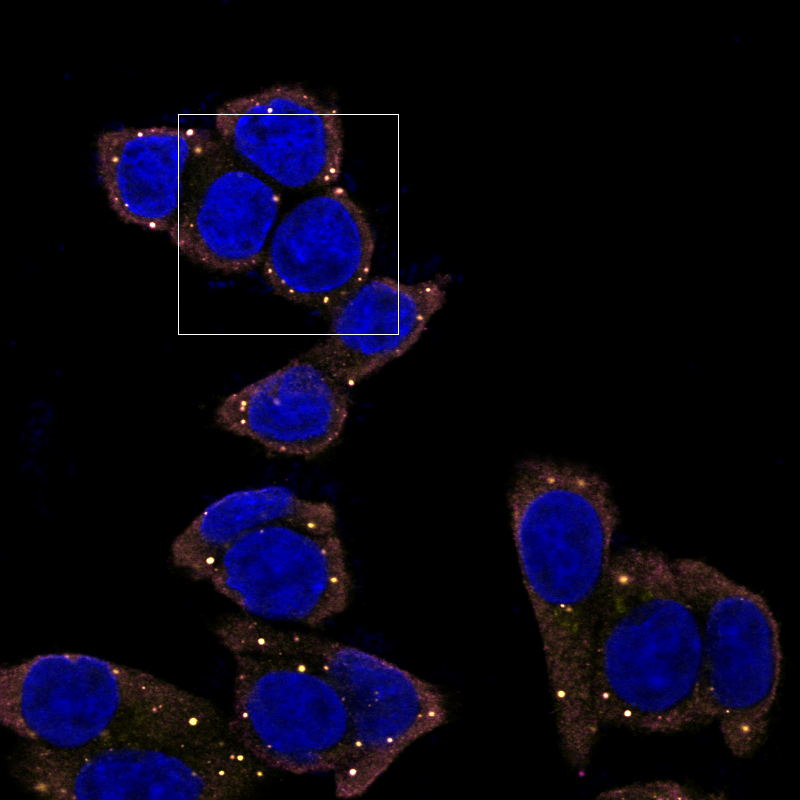

Supplement: Supplementary file 7 — Source data Fig. 4 [file 44318_2024_120_MOESM7_ESM.zip › Figure 4/4C/Above/Merge.tif]

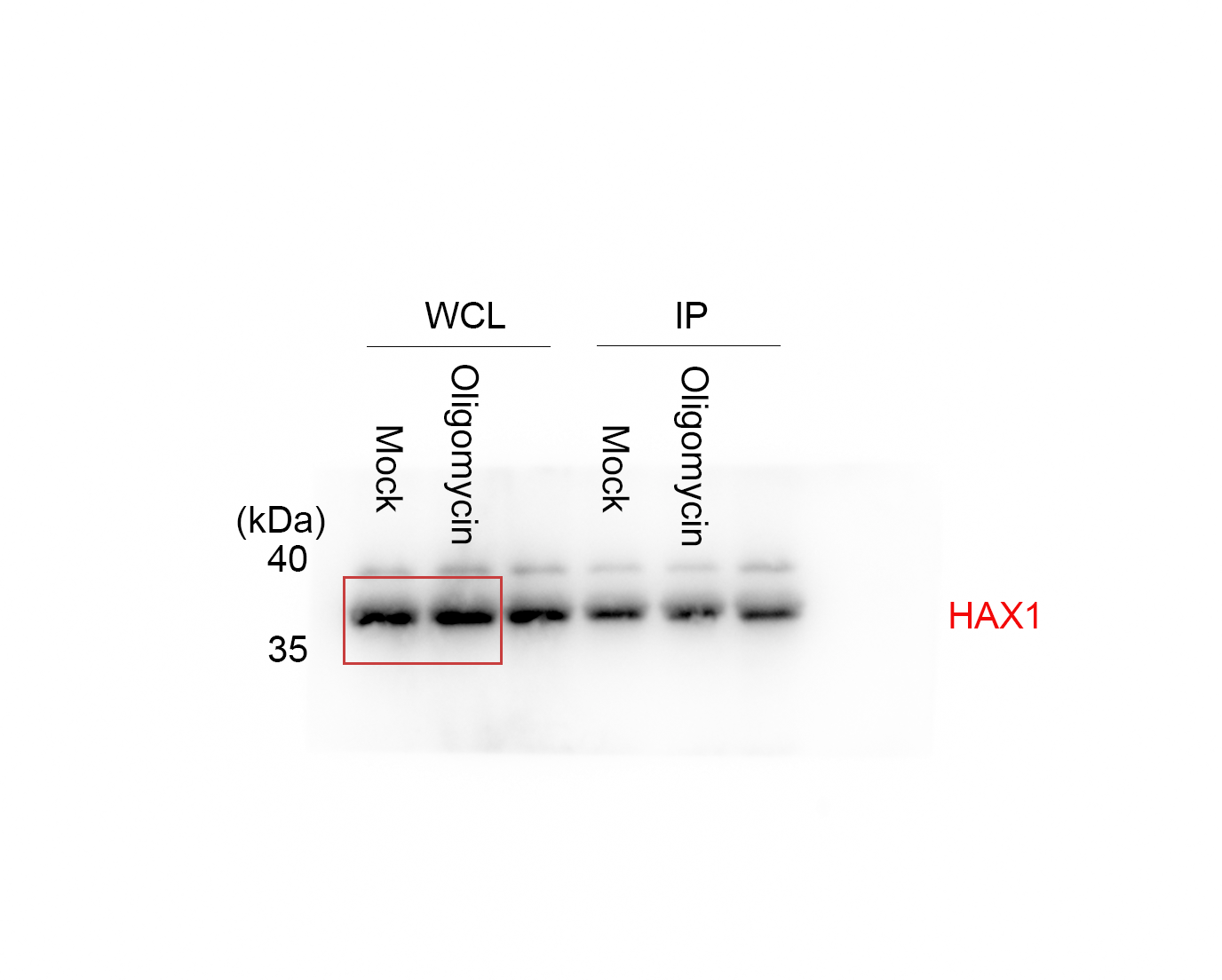

Supplement: Supplementary file 7 — Source data Fig. 4 [file 44318_2024_120_MOESM7_ESM.zip › Figure 4/4D/WCL/western-HAX1.Tif]

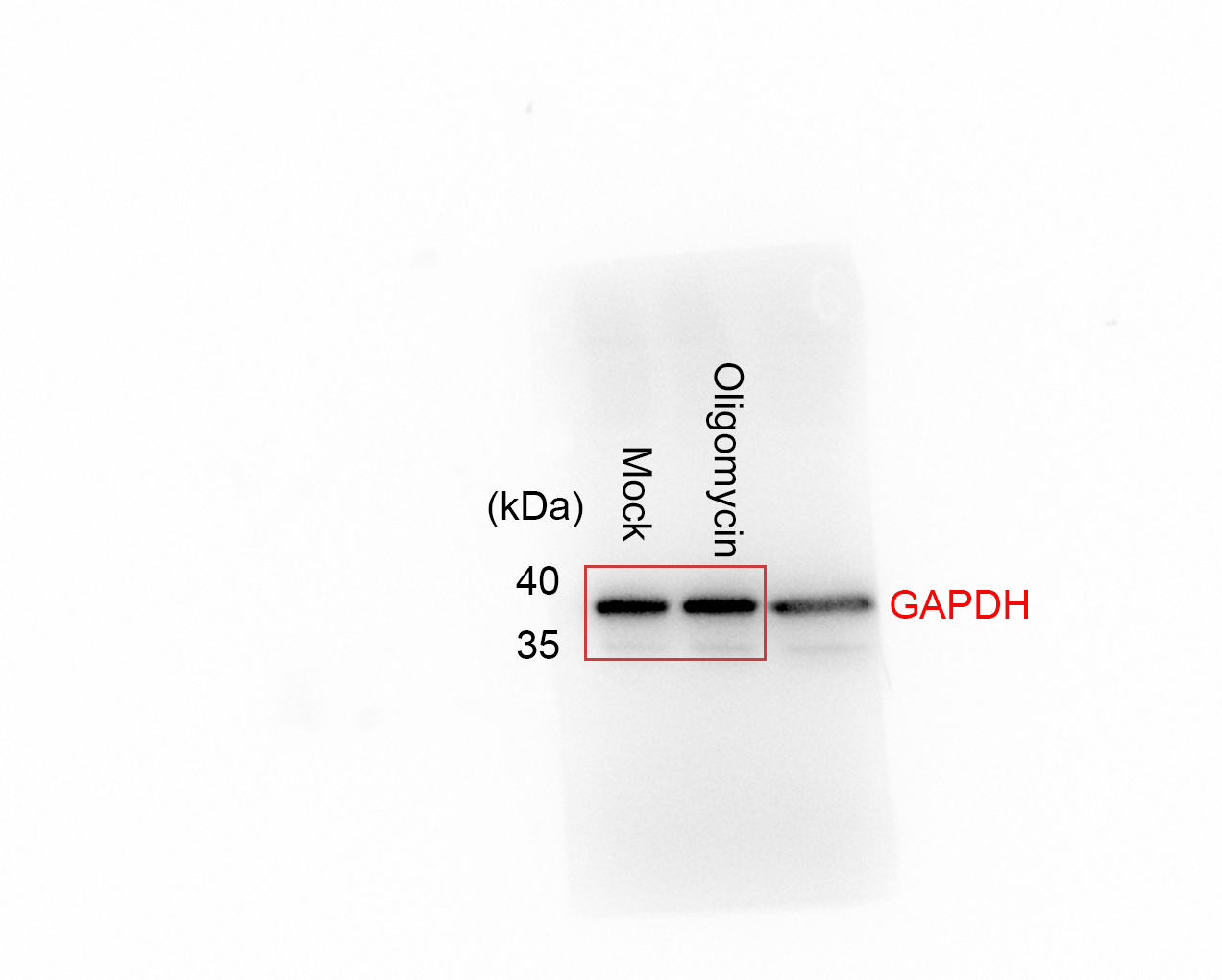

Supplement: Supplementary file 7 — Source data Fig. 4 [file 44318_2024_120_MOESM7_ESM.zip › Figure 4/4D/WCL/western-GAPDH.Tif]

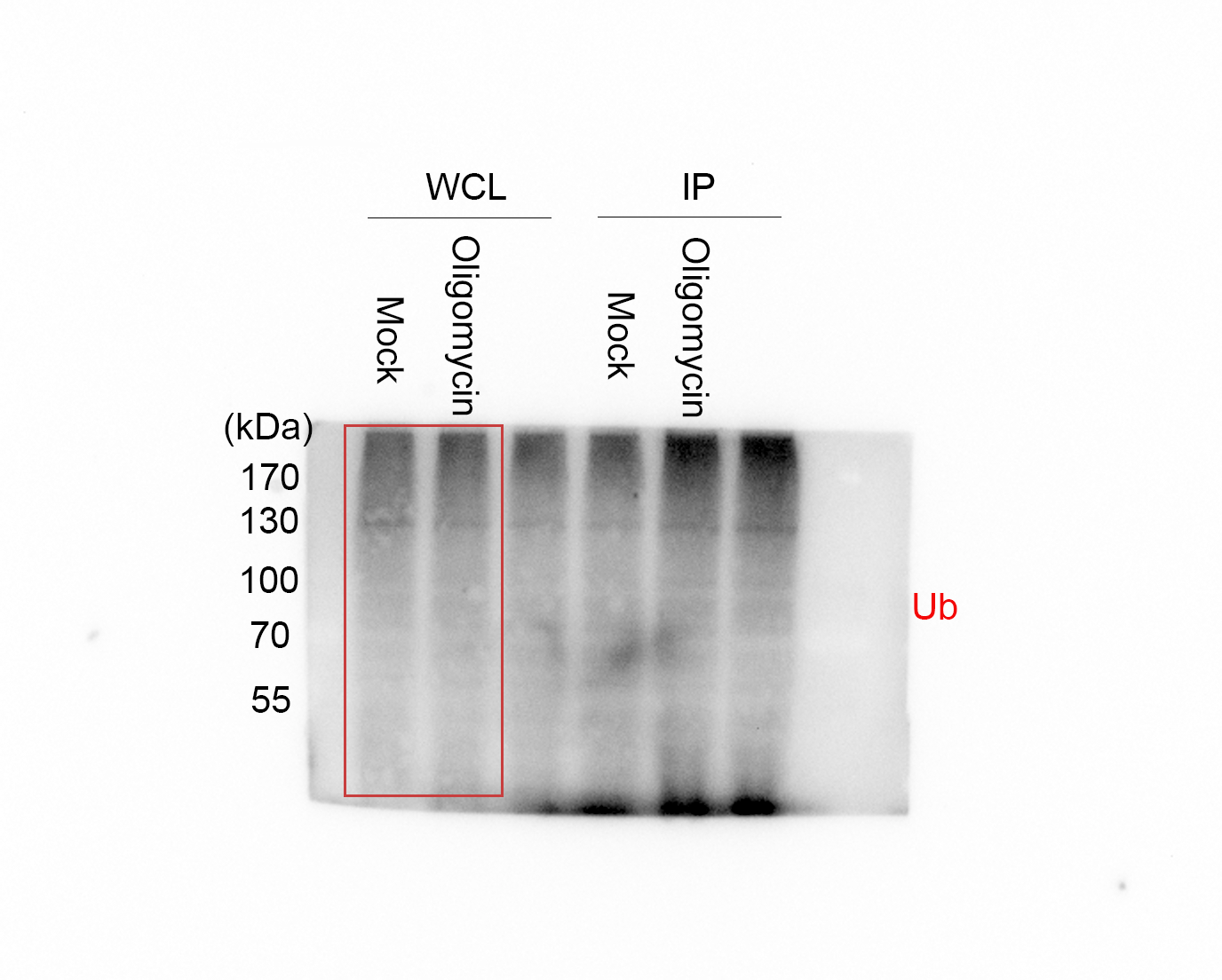

Supplement: Supplementary file 7 — Source data Fig. 4 [file 44318_2024_120_MOESM7_ESM.zip › Figure 4/4D/WCL/western-Ub-proteins.Tif]

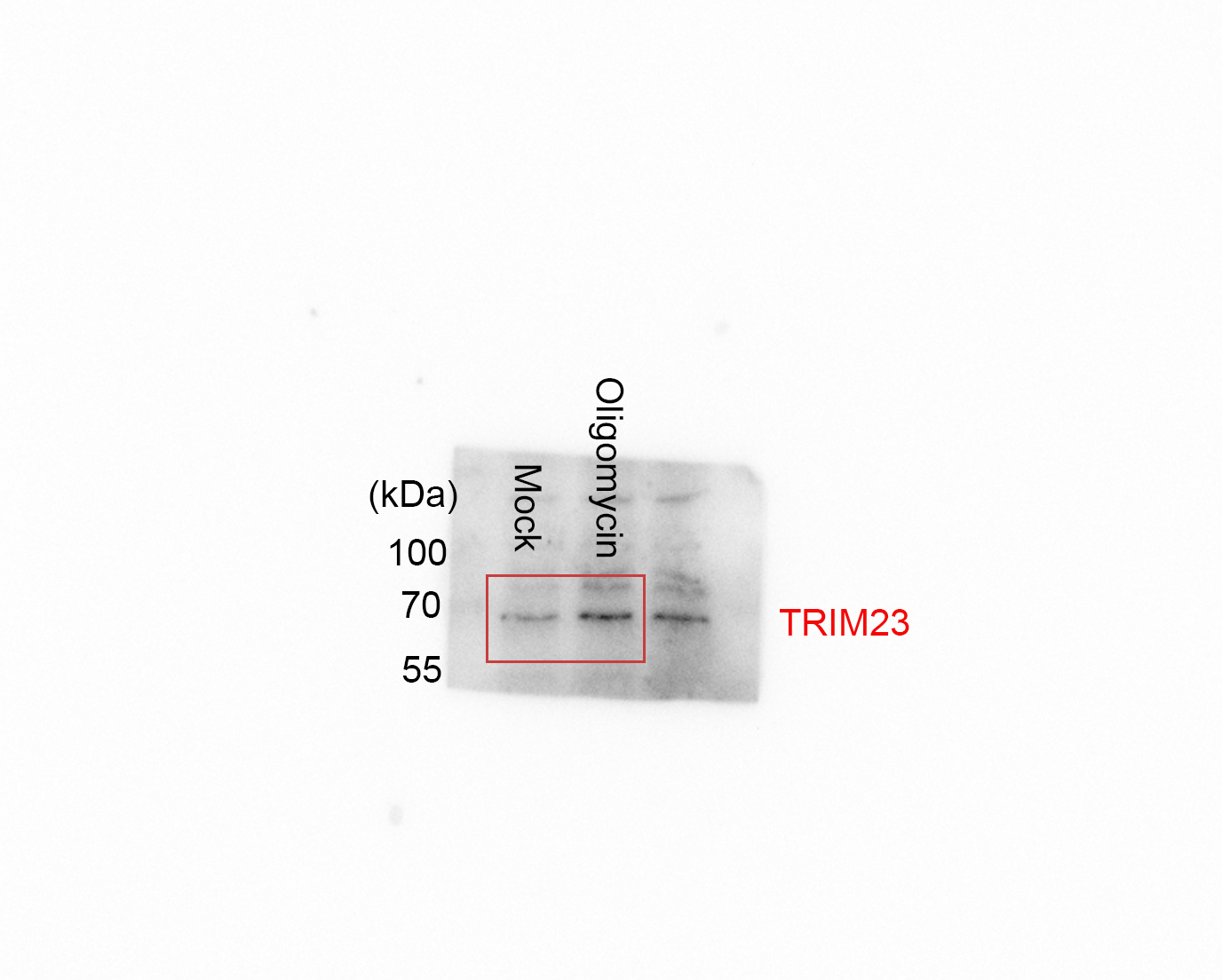

Supplement: Supplementary file 7 — Source data Fig. 4 [file 44318_2024_120_MOESM7_ESM.zip › Figure 4/4D/WCL/western-TRIM23.Tif]

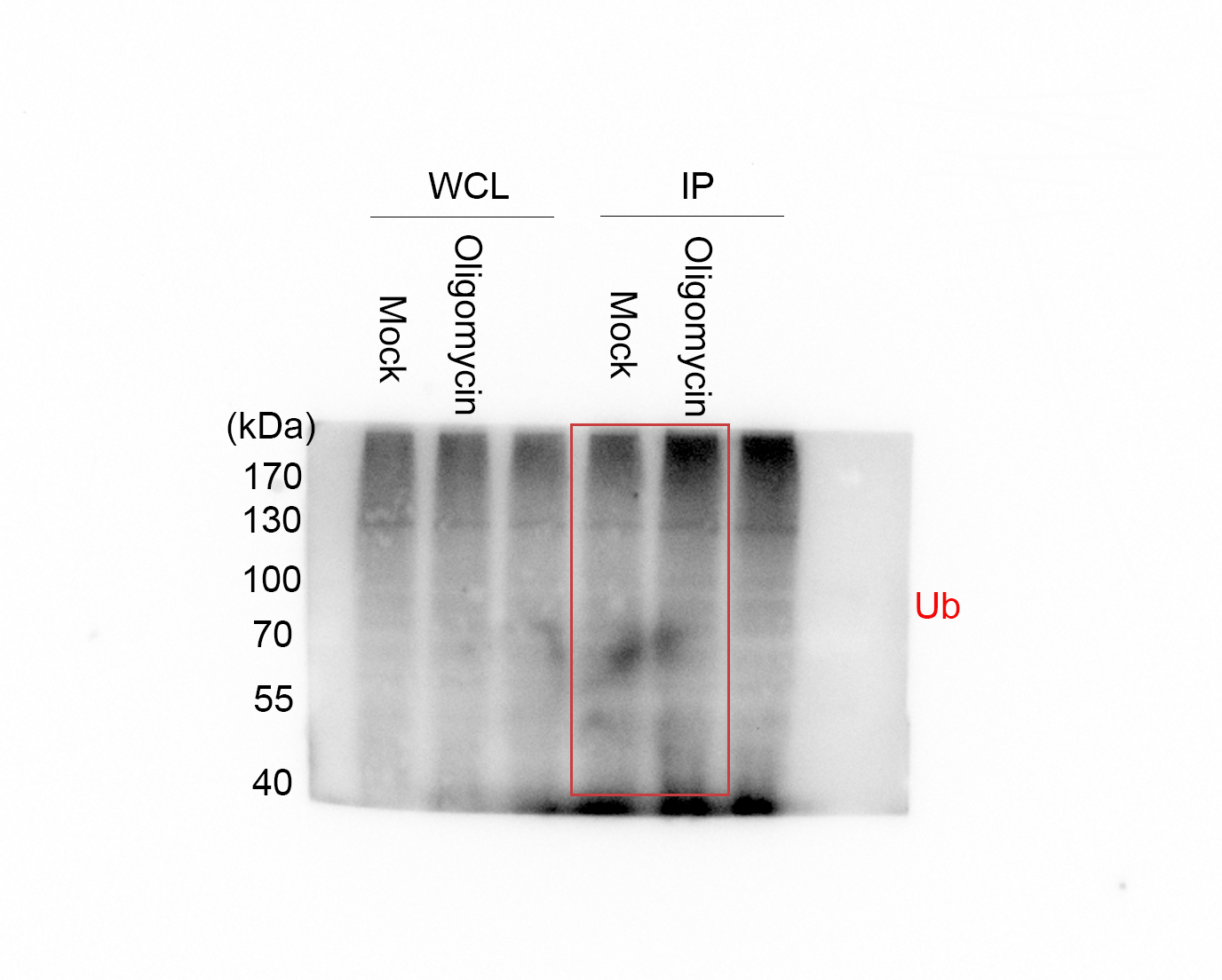

Supplement: Supplementary file 7 — Source data Fig. 4 [file 44318_2024_120_MOESM7_ESM.zip › Figure 4/4D/IP/western-HAX1-Ub.Tif]

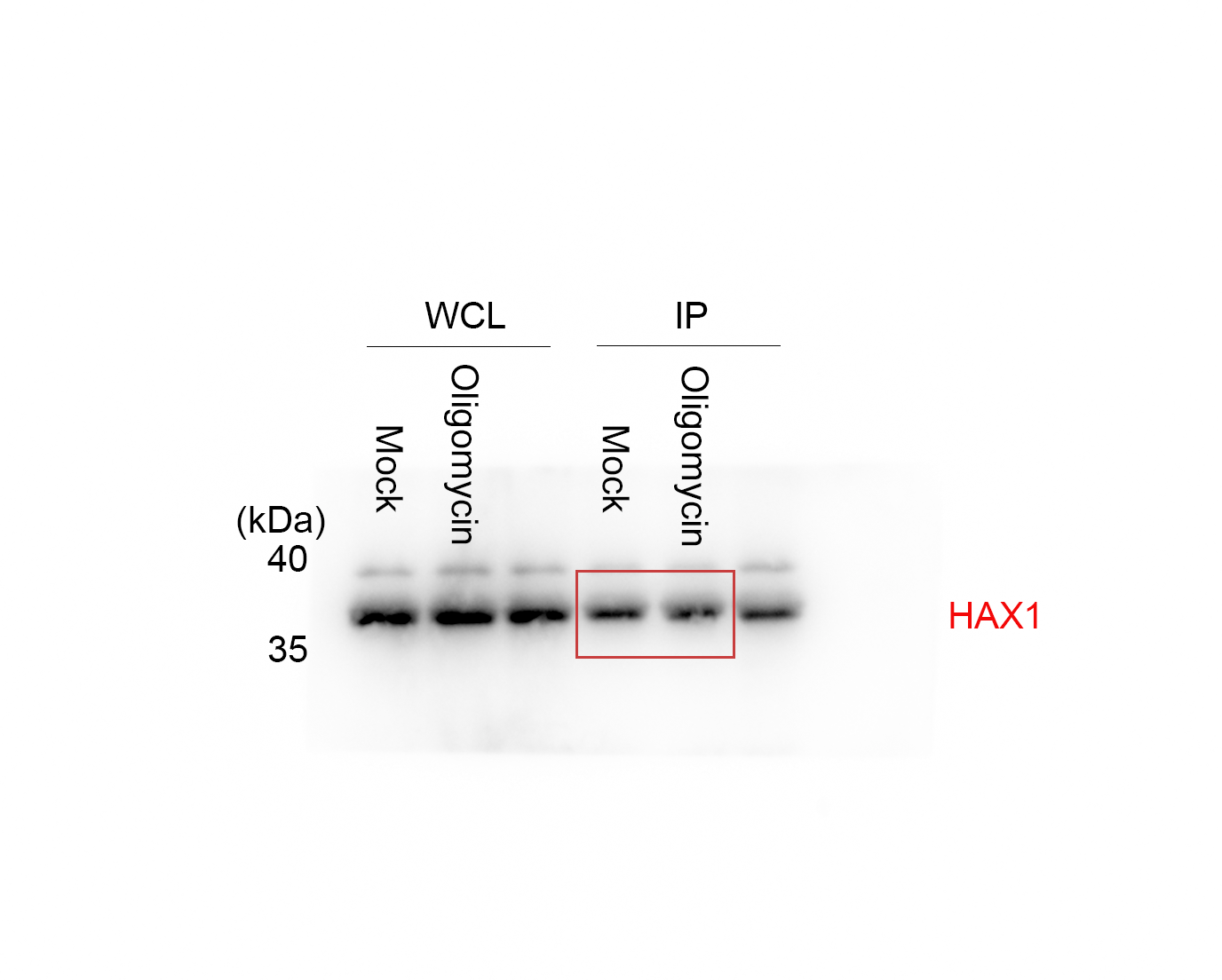

Supplement: Supplementary file 7 — Source data Fig. 4 [file 44318_2024_120_MOESM7_ESM.zip › Figure 4/4D/IP/western-HAX1.Tif]

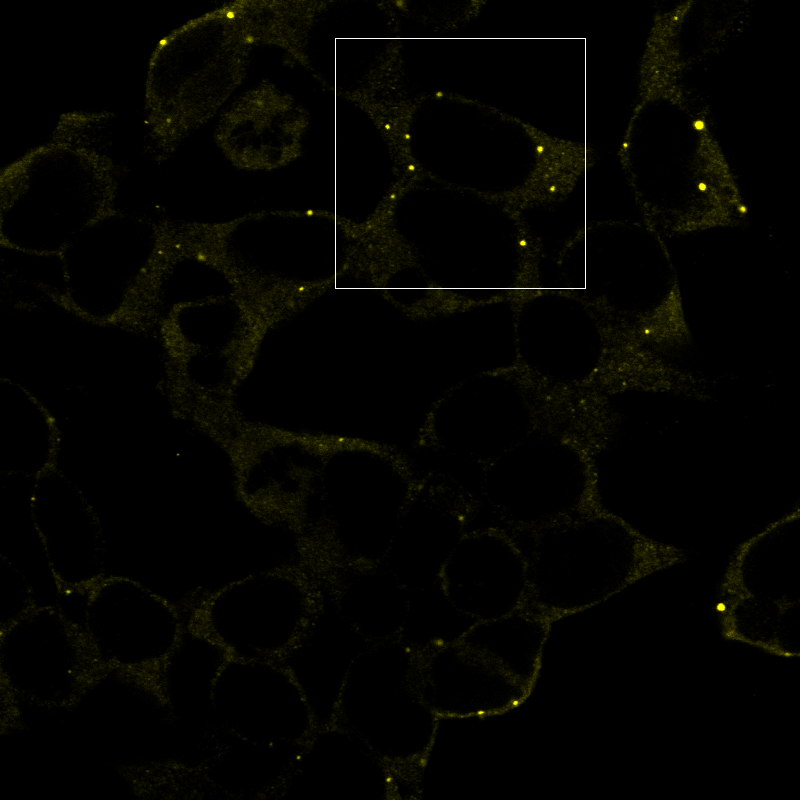

Supplement: Supplementary file 7 — Source data Fig. 4 [file 44318_2024_120_MOESM7_ESM.zip › Figure 4/4A/Below/LSM14A.tif]

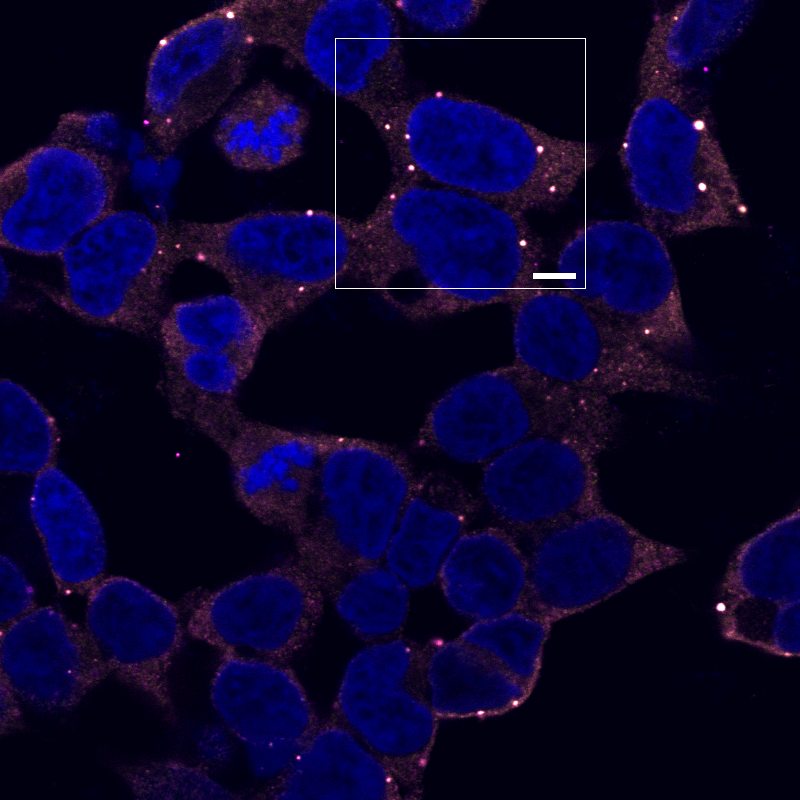

Supplement: Supplementary file 7 — Source data Fig. 4 [file 44318_2024_120_MOESM7_ESM.zip › Figure 4/4A/Below/Merge.tif]

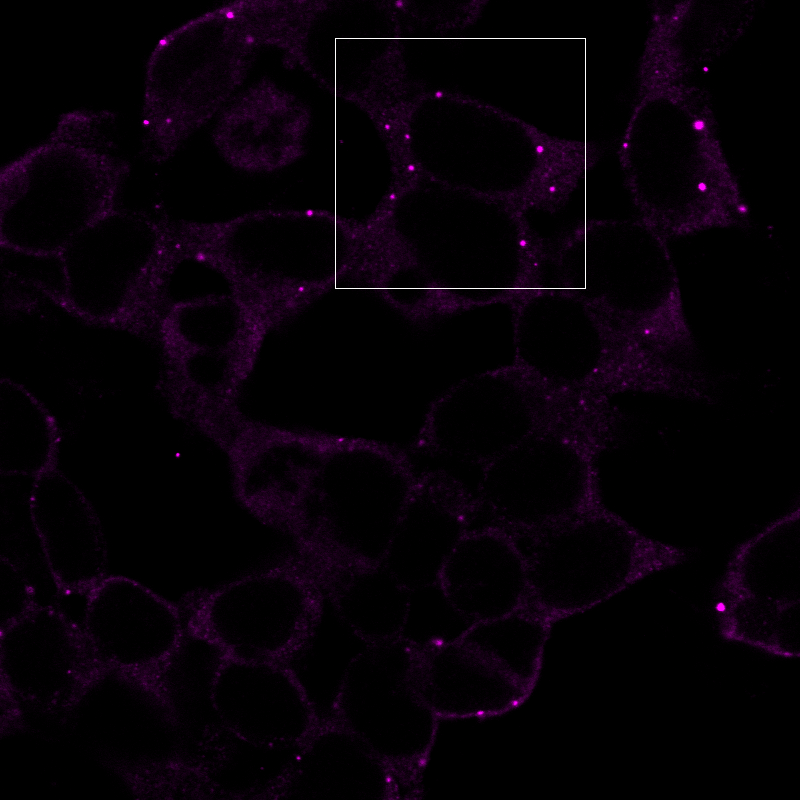

Supplement: Supplementary file 7 — Source data Fig. 4 [file 44318_2024_120_MOESM7_ESM.zip › Figure 4/4A/Below/TRIM23.tif]

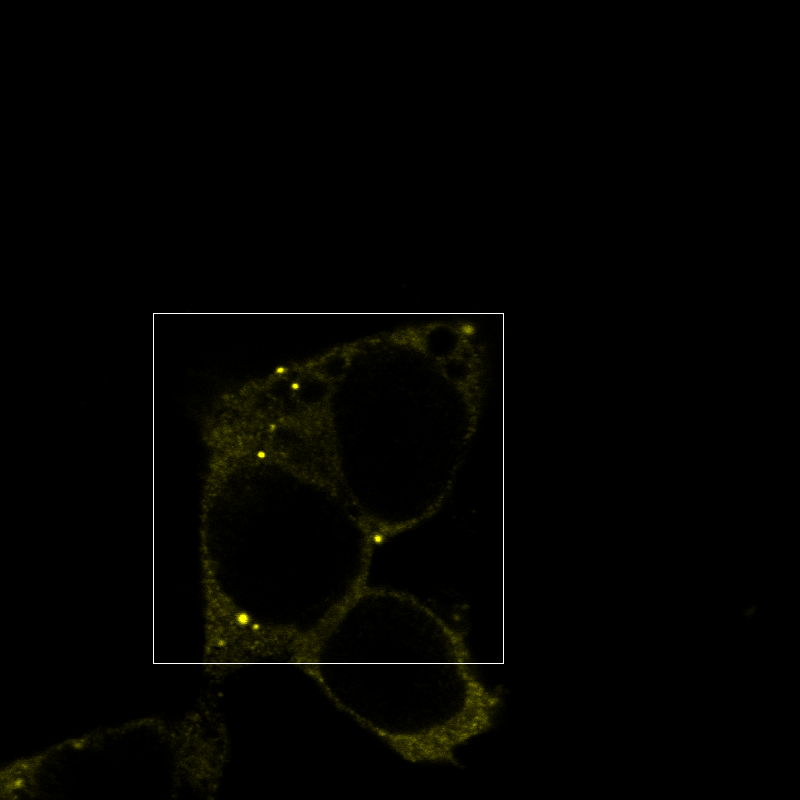

Supplement: Supplementary file 7 — Source data Fig. 4 [file 44318_2024_120_MOESM7_ESM.zip › Figure 4/4A/Above/DCP1A.tif]

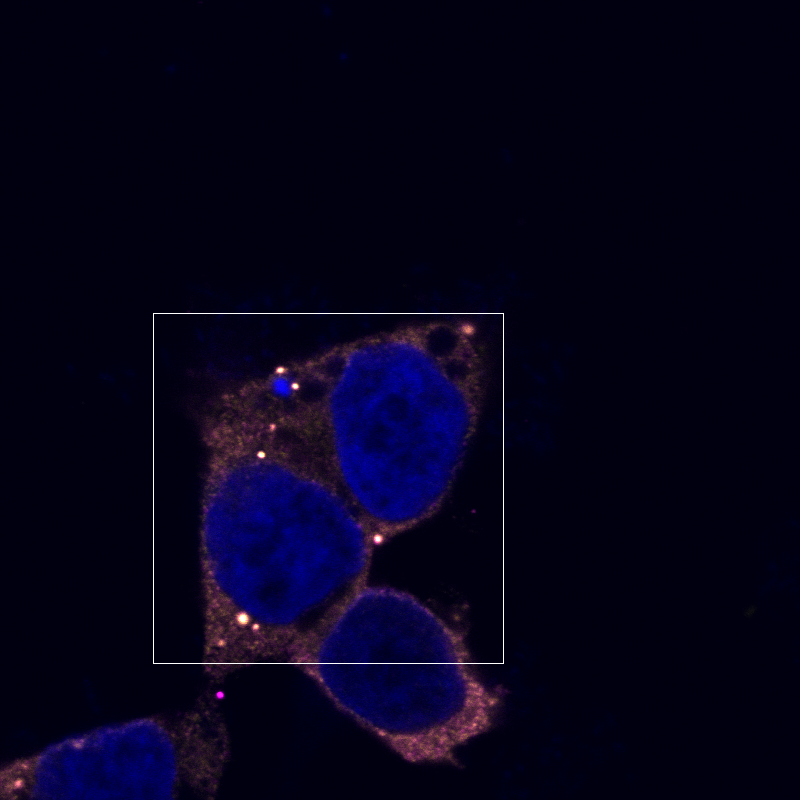

Supplement: Supplementary file 7 — Source data Fig. 4 [file 44318_2024_120_MOESM7_ESM.zip › Figure 4/4A/Above/Merge.tif]

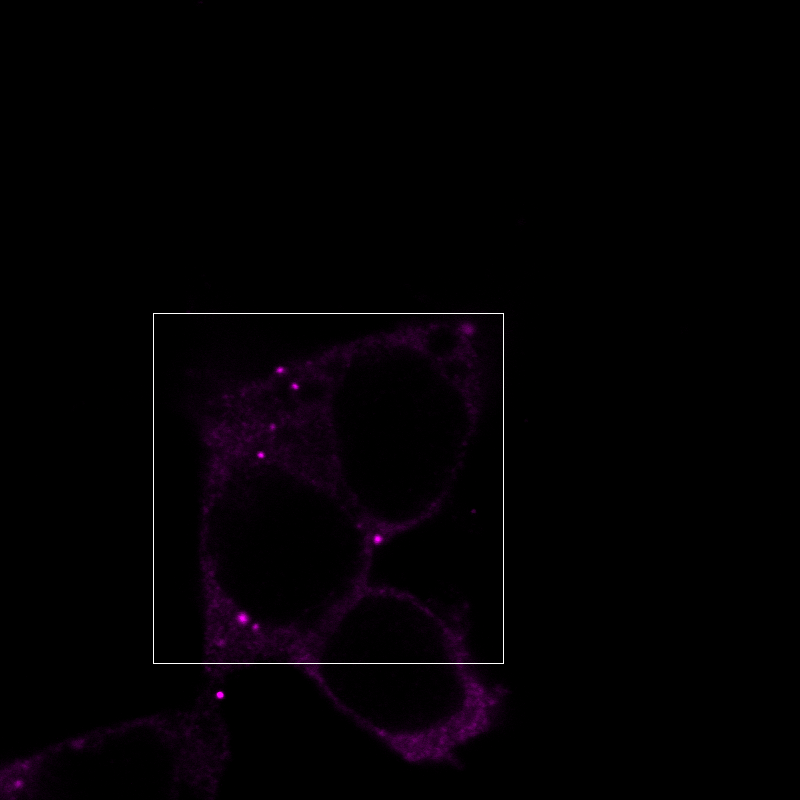

Supplement: Supplementary file 7 — Source data Fig. 4 [file 44318_2024_120_MOESM7_ESM.zip › Figure 4/4A/Above/TRIM23.tif]

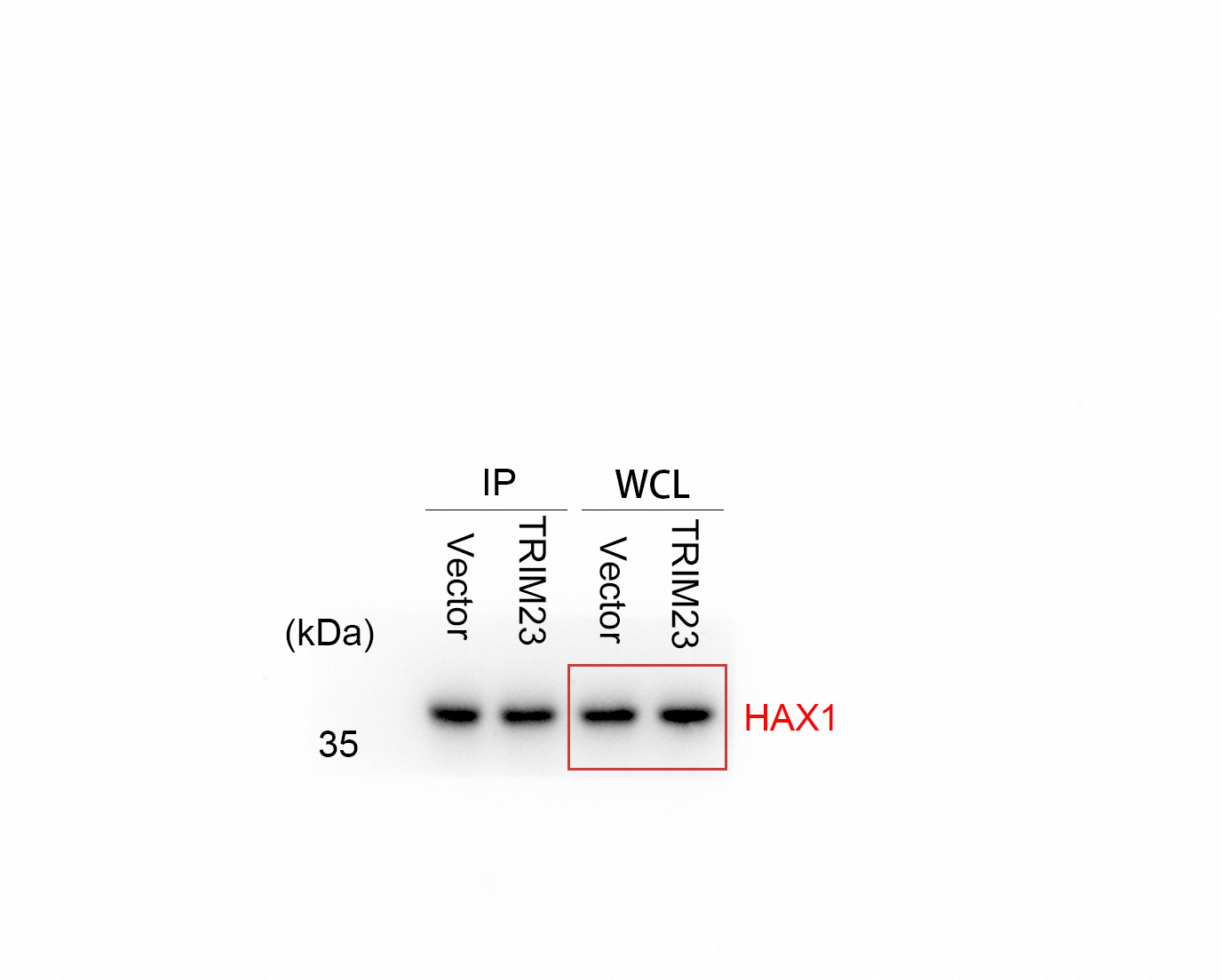

Supplement: Supplementary file 7 — Source data Fig. 4 [file 44318_2024_120_MOESM7_ESM.zip › Figure 4/4F/WCL/western-HAX1.Tif]

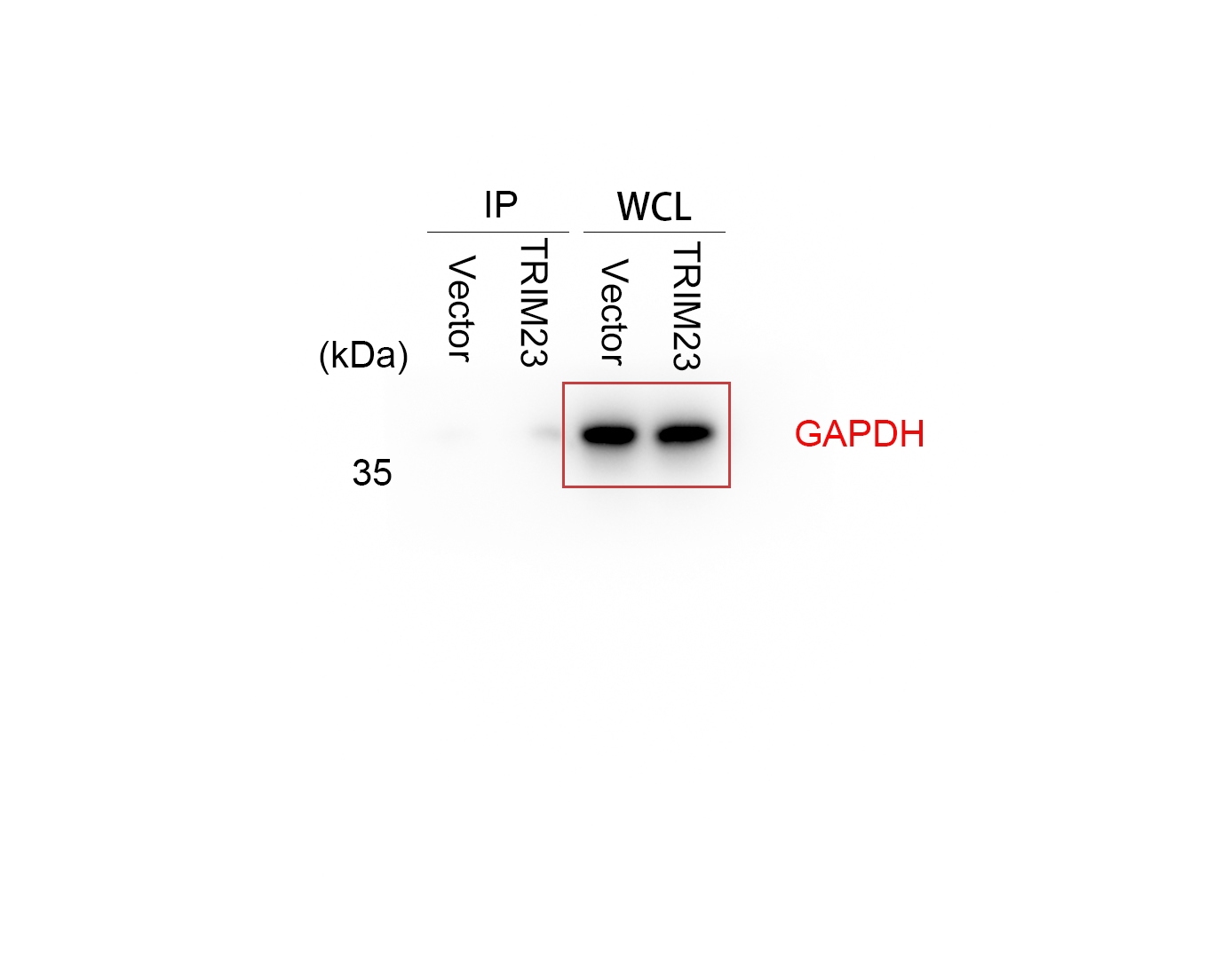

Supplement: Supplementary file 7 — Source data Fig. 4 [file 44318_2024_120_MOESM7_ESM.zip › Figure 4/4F/WCL/western-GAPDH.Tif]

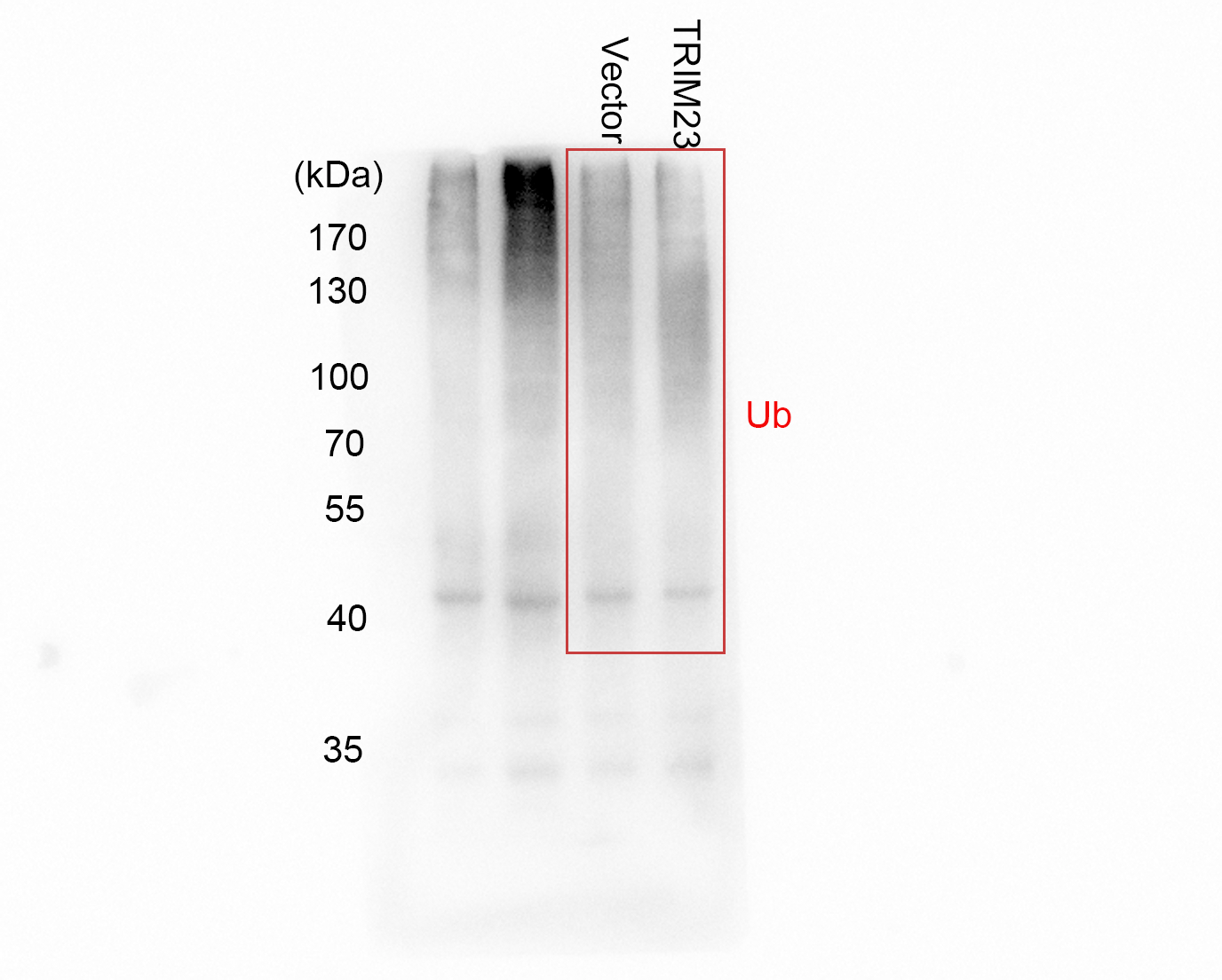

Supplement: Supplementary file 7 — Source data Fig. 4 [file 44318_2024_120_MOESM7_ESM.zip › Figure 4/4F/WCL/western-Ub-proteins.Tif]

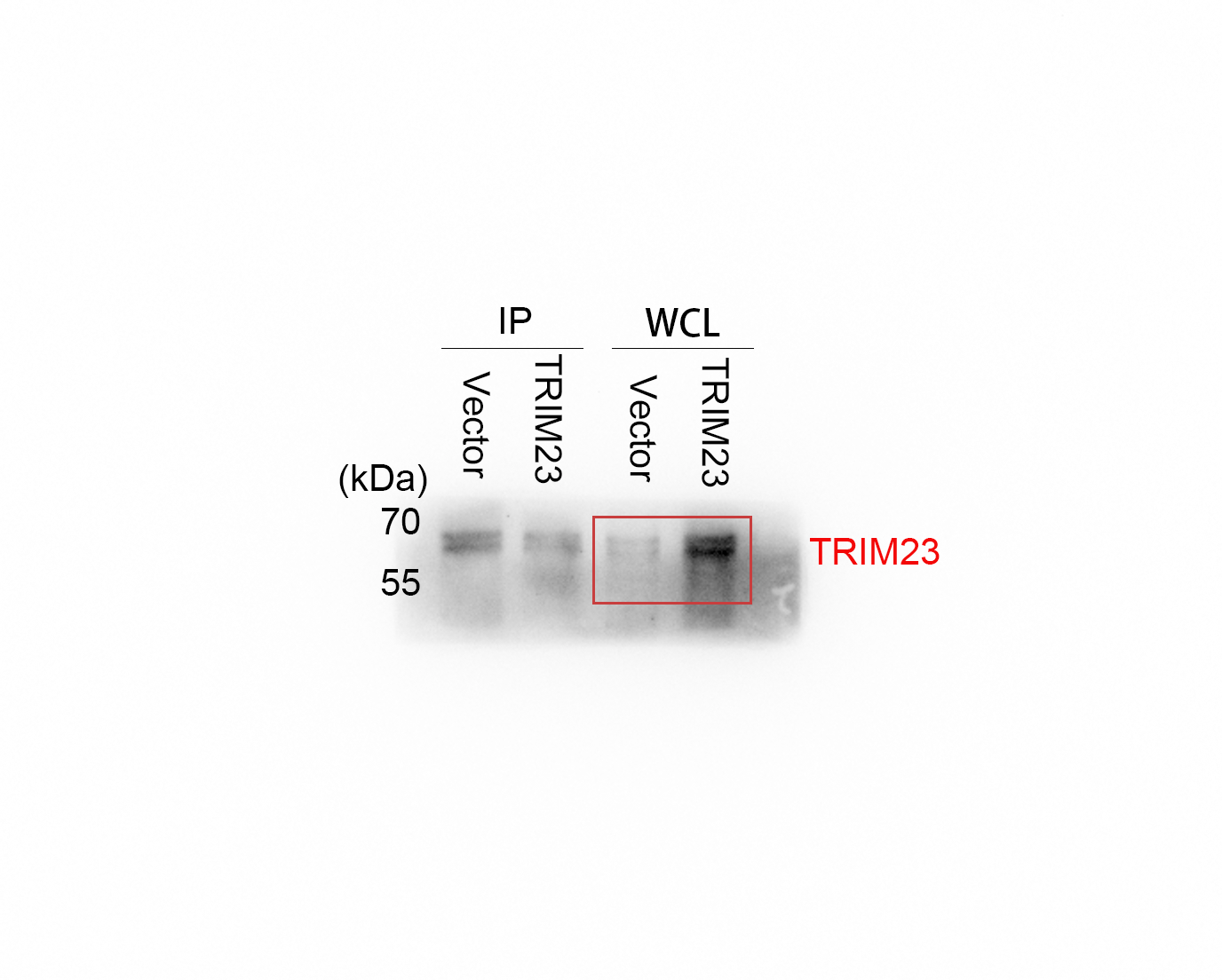

Supplement: Supplementary file 7 — Source data Fig. 4 [file 44318_2024_120_MOESM7_ESM.zip › Figure 4/4F/WCL/western-TRIM23.Tif]

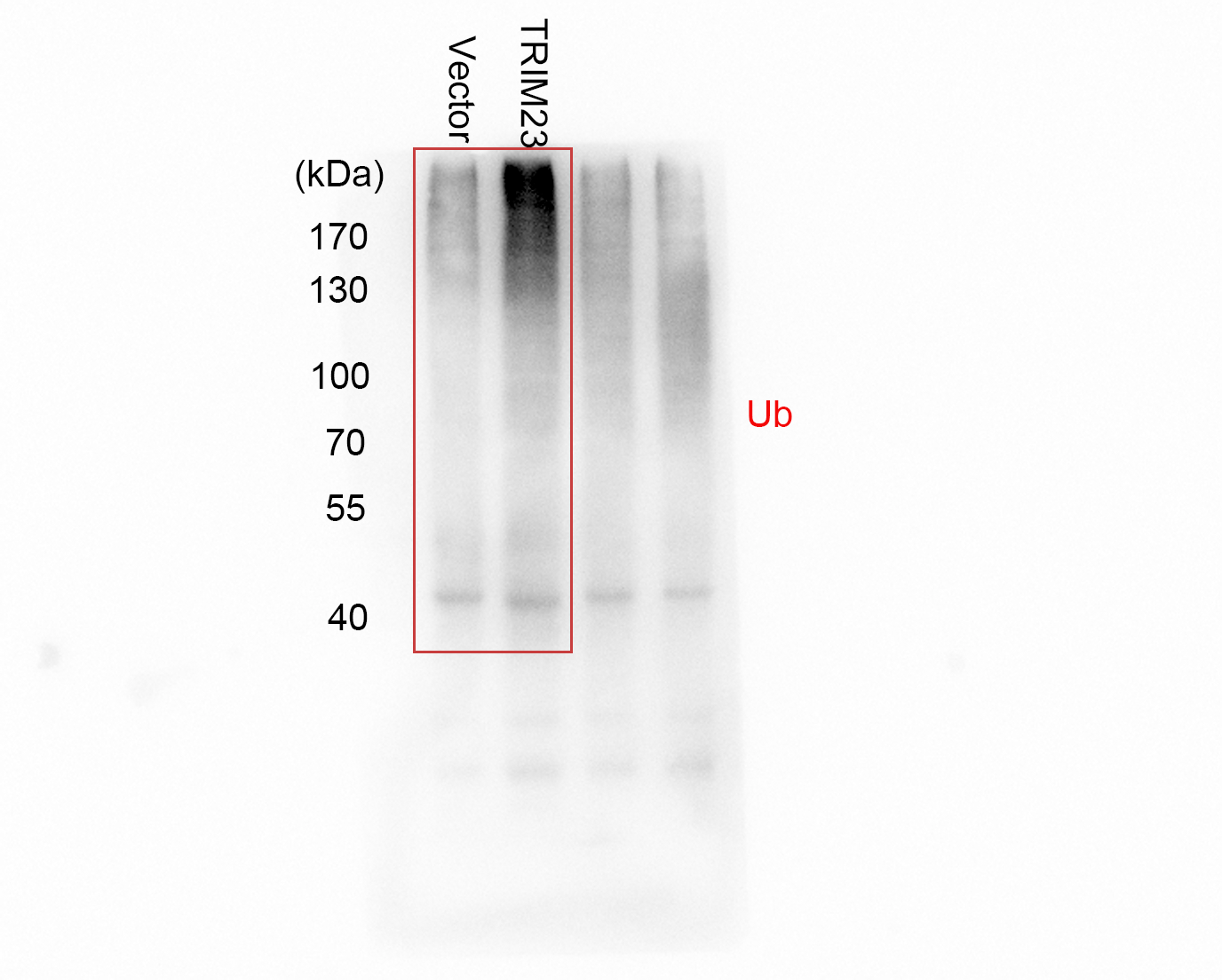

Supplement: Supplementary file 7 — Source data Fig. 4 [file 44318_2024_120_MOESM7_ESM.zip › Figure 4/4F/IP/western-HAX1-Ub.Tif]

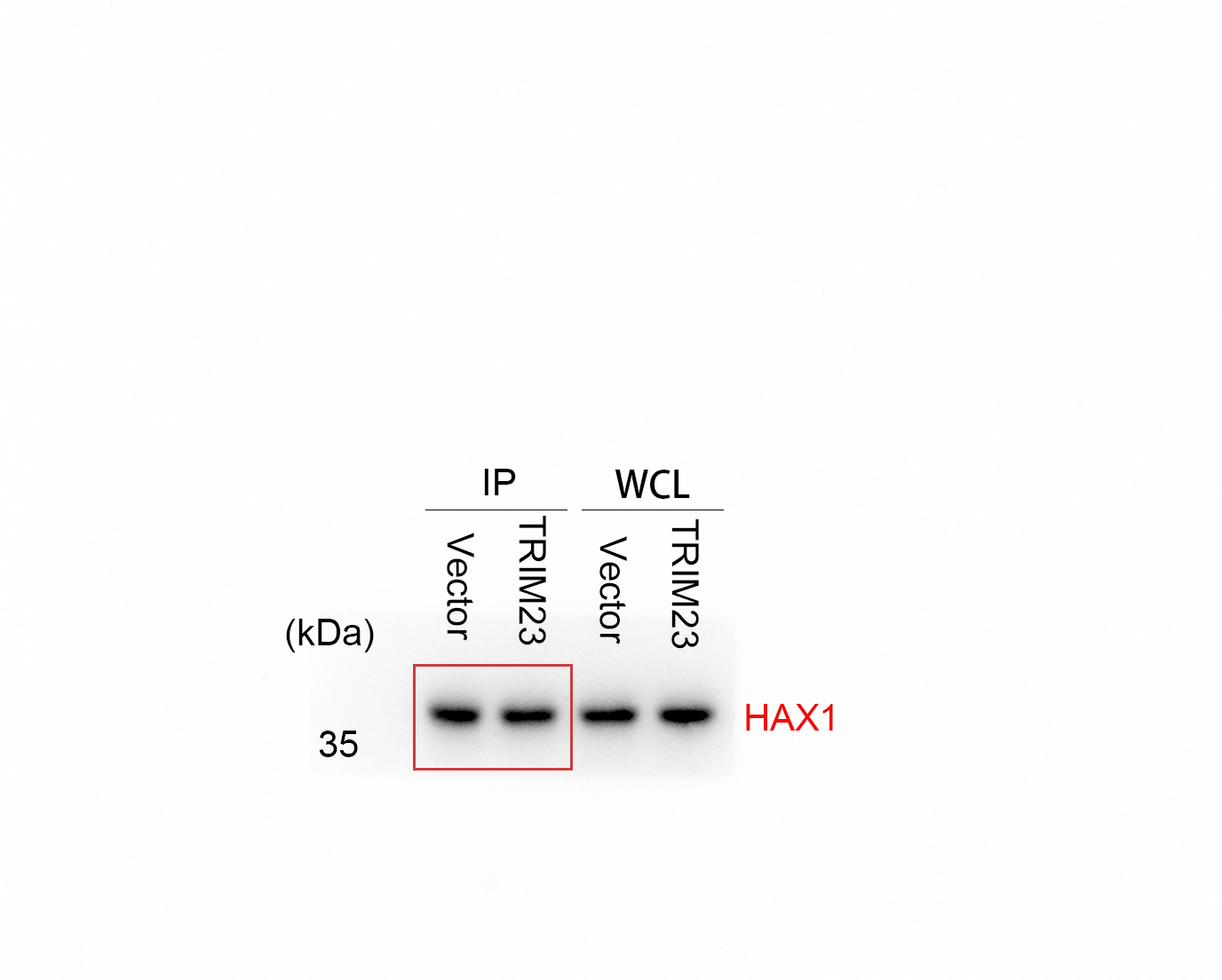

Supplement: Supplementary file 7 — Source data Fig. 4 [file 44318_2024_120_MOESM7_ESM.zip › Figure 4/4F/IP/western-HAX1.Tif]

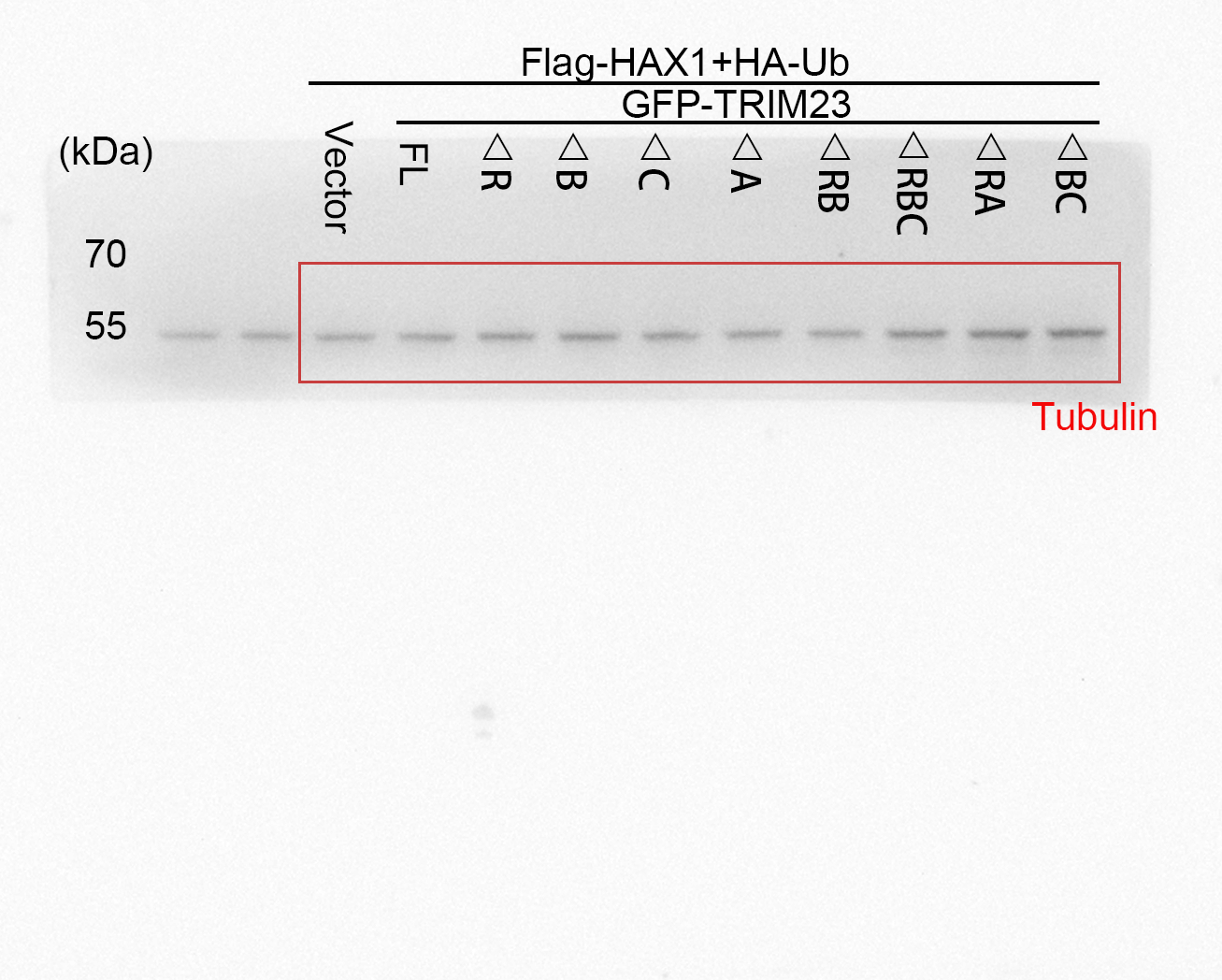

Supplement: Supplementary file 7 — Source data Fig. 4 [file 44318_2024_120_MOESM7_ESM.zip › Figure 4/4I/WCL/western-Tubulin.Tif]

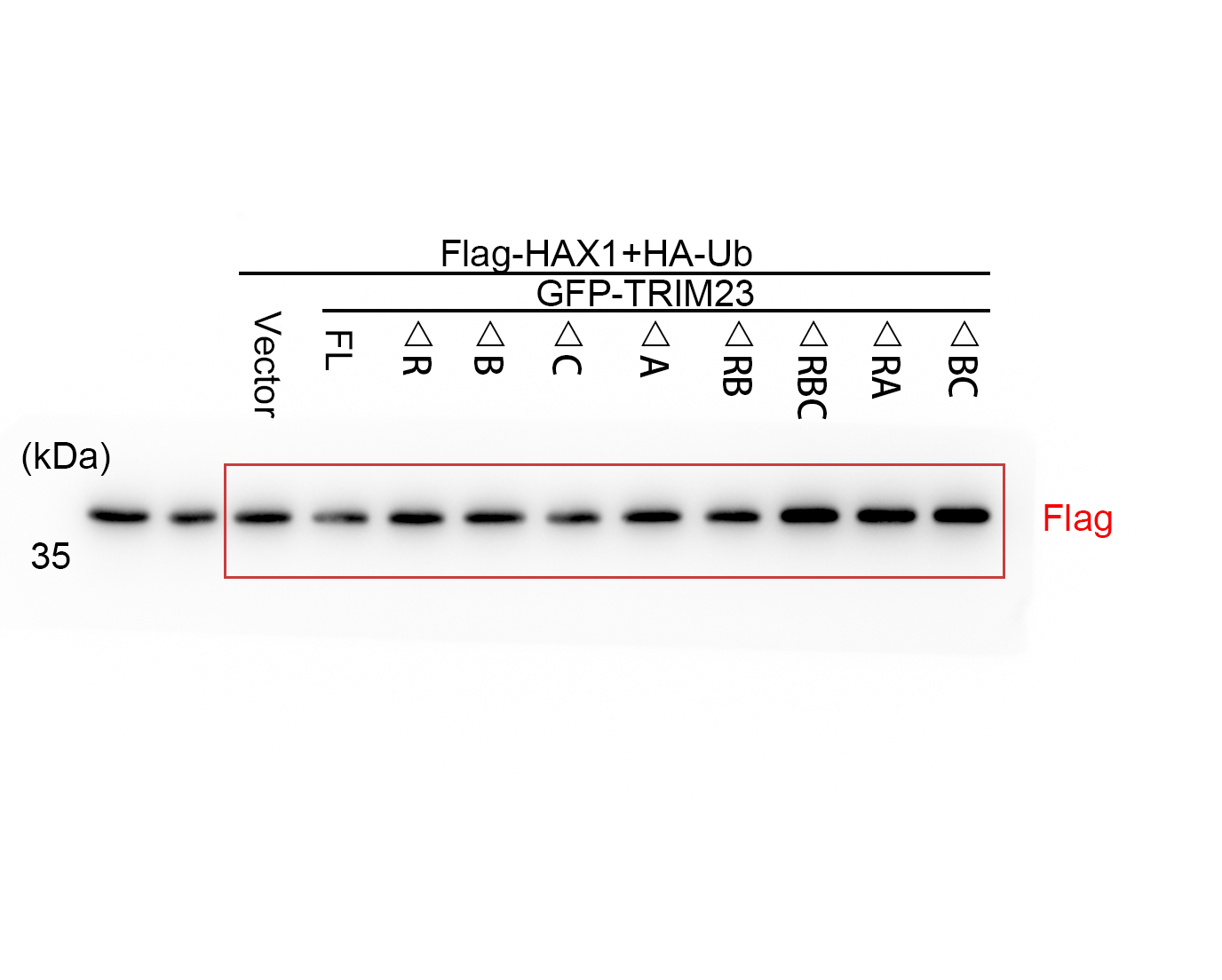

Supplement: Supplementary file 7 — Source data Fig. 4 [file 44318_2024_120_MOESM7_ESM.zip › Figure 4/4I/WCL/western-Flag-HAX1.Tif]

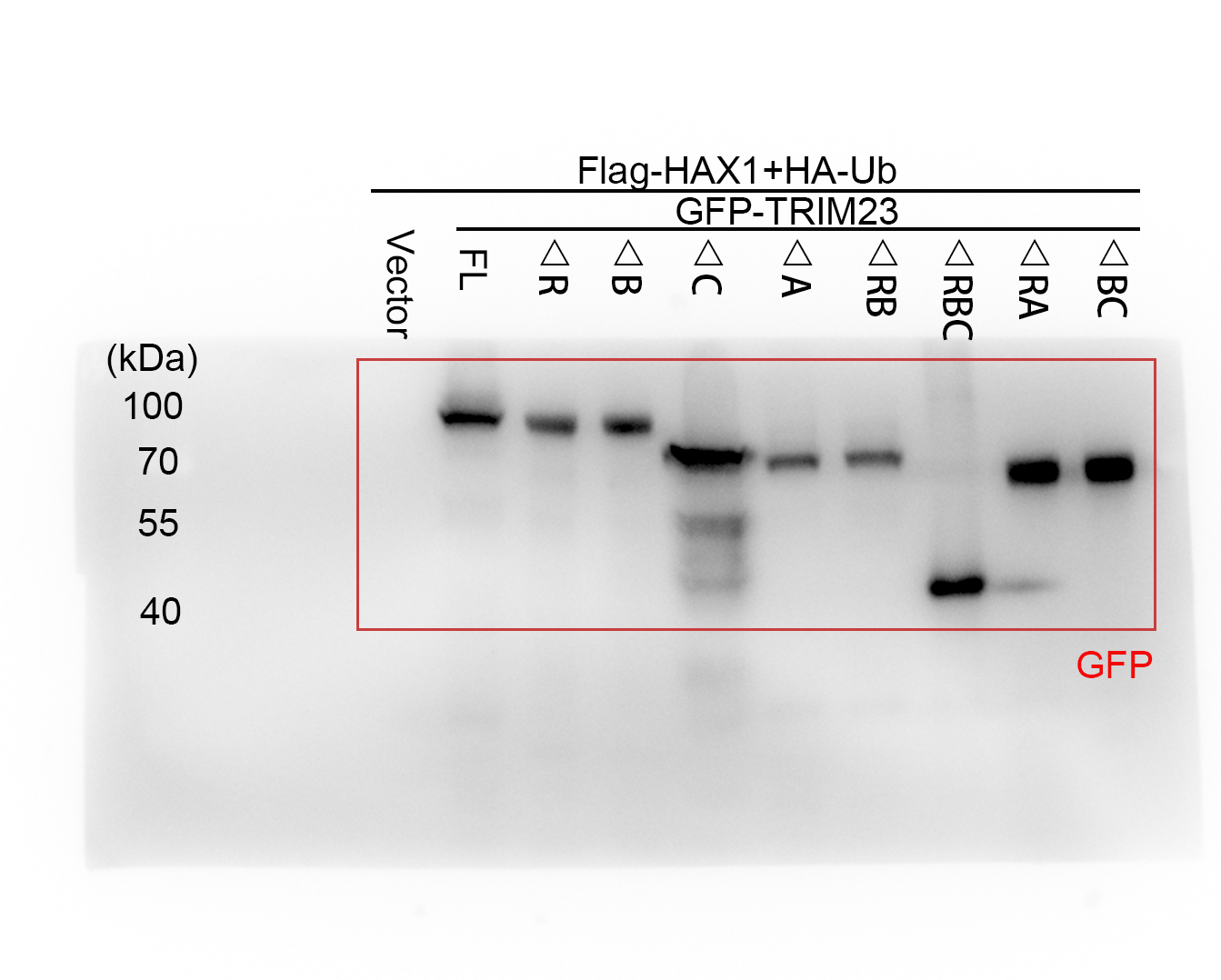

Supplement: Supplementary file 7 — Source data Fig. 4 [file 44318_2024_120_MOESM7_ESM.zip › Figure 4/4I/WCL/western-GFP-TRIM23.Tif]

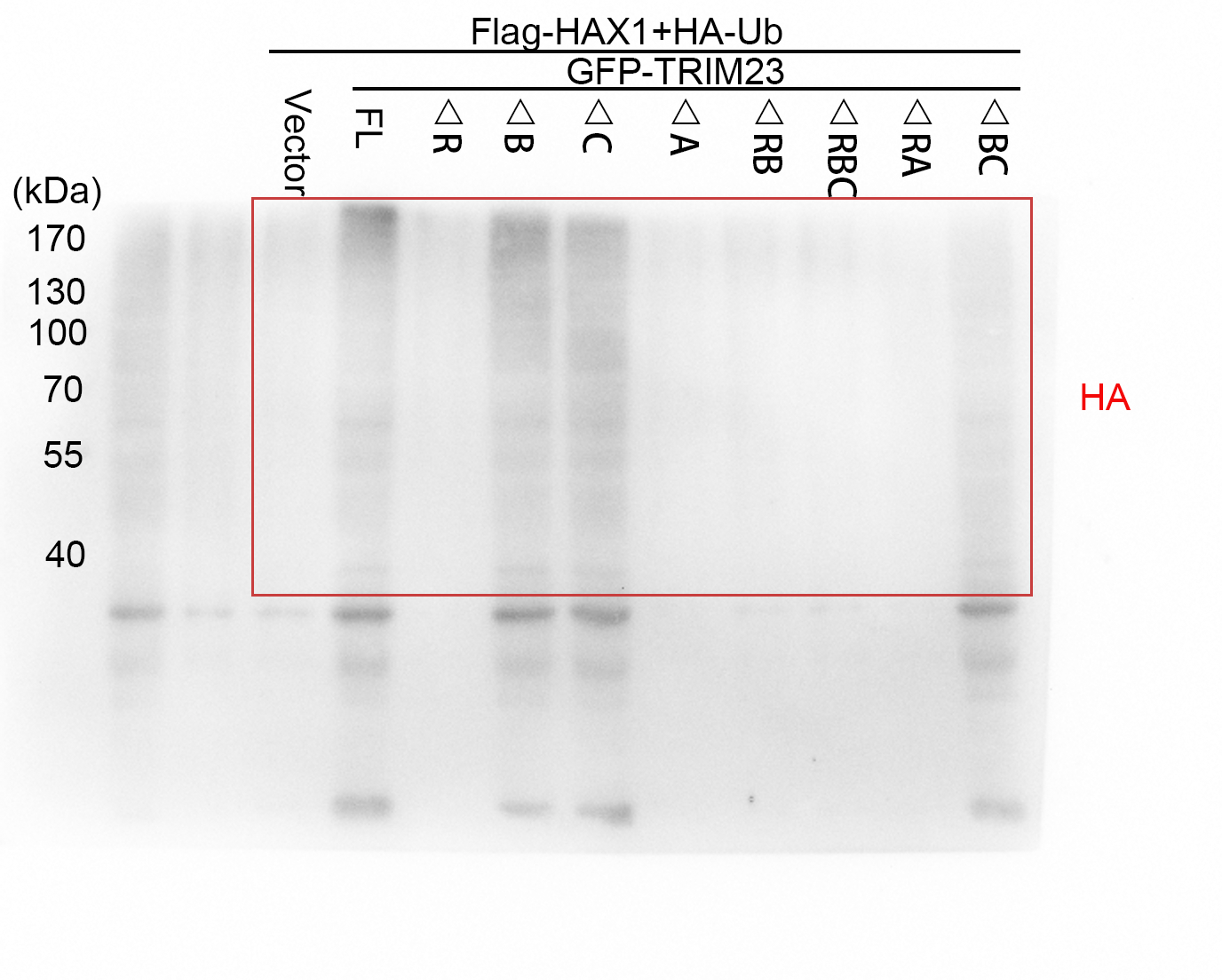

Supplement: Supplementary file 7 — Source data Fig. 4 [file 44318_2024_120_MOESM7_ESM.zip › Figure 4/4I/IP/western-Flag-HAX1-Ub.Tif]

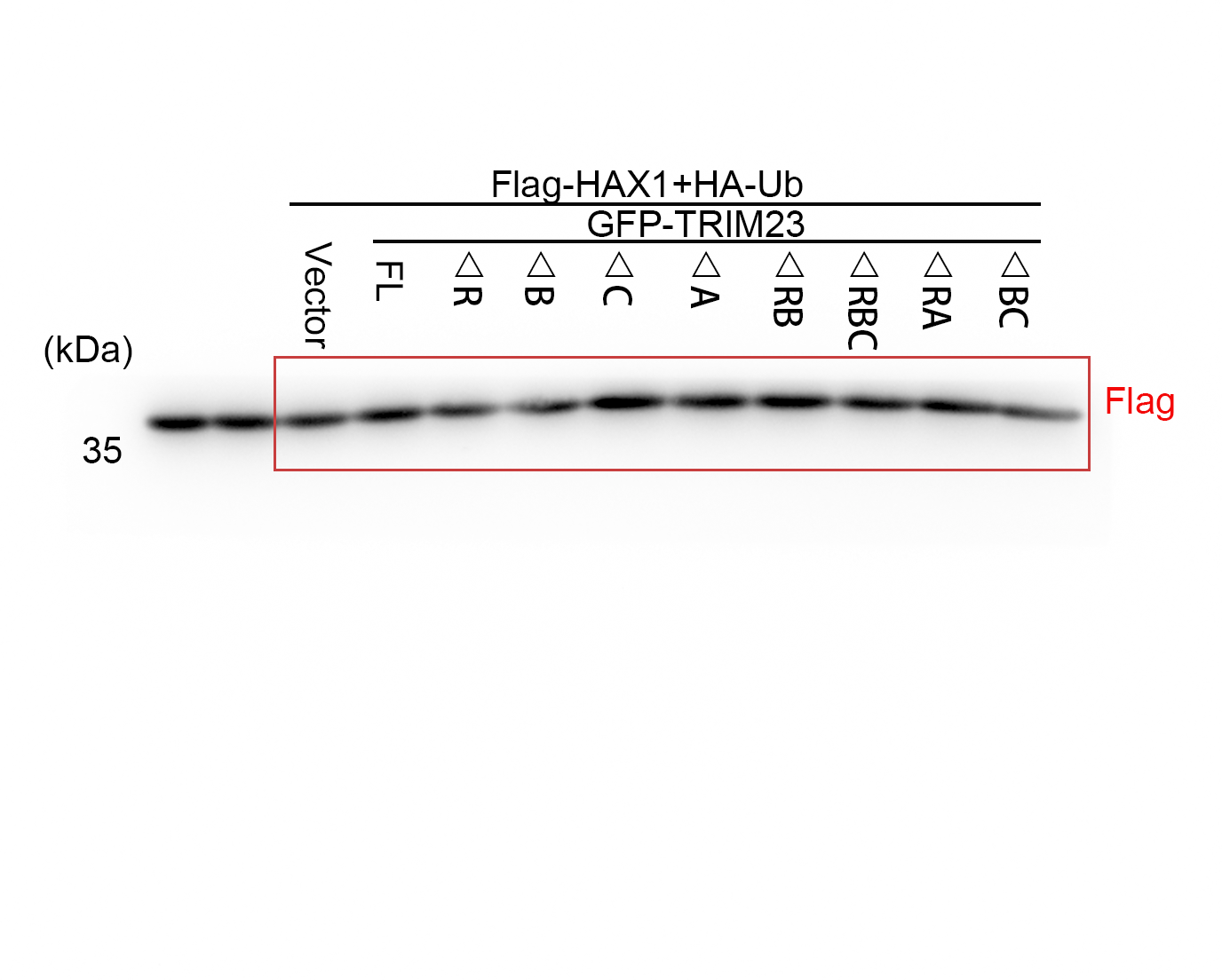

Supplement: Supplementary file 7 — Source data Fig. 4 [file 44318_2024_120_MOESM7_ESM.zip › Figure 4/4I/IP/western-Flag-HAX1.Tif]

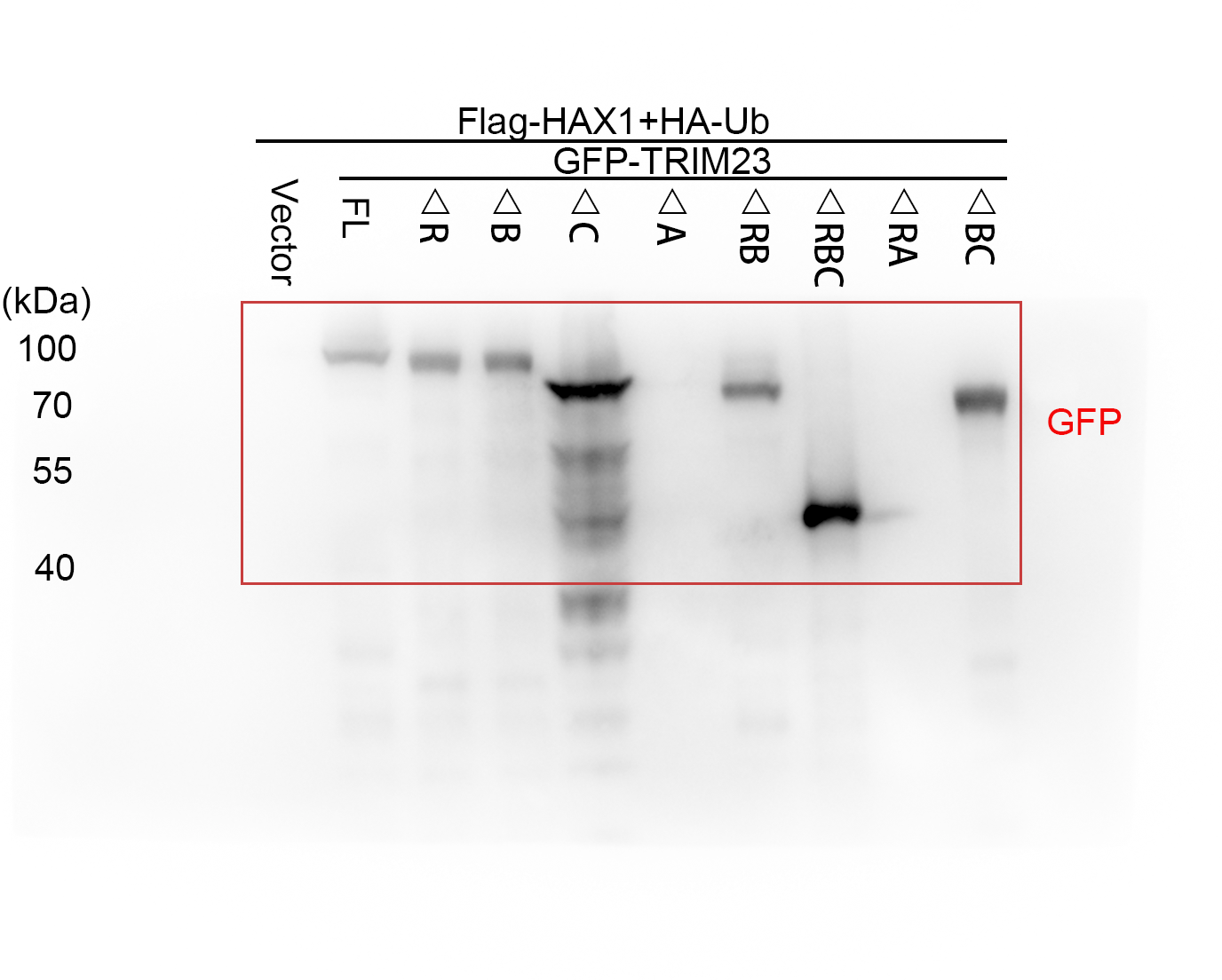

Supplement: Supplementary file 7 — Source data Fig. 4 [file 44318_2024_120_MOESM7_ESM.zip › Figure 4/4I/IP/western-GFP-TRIM23.Tif]

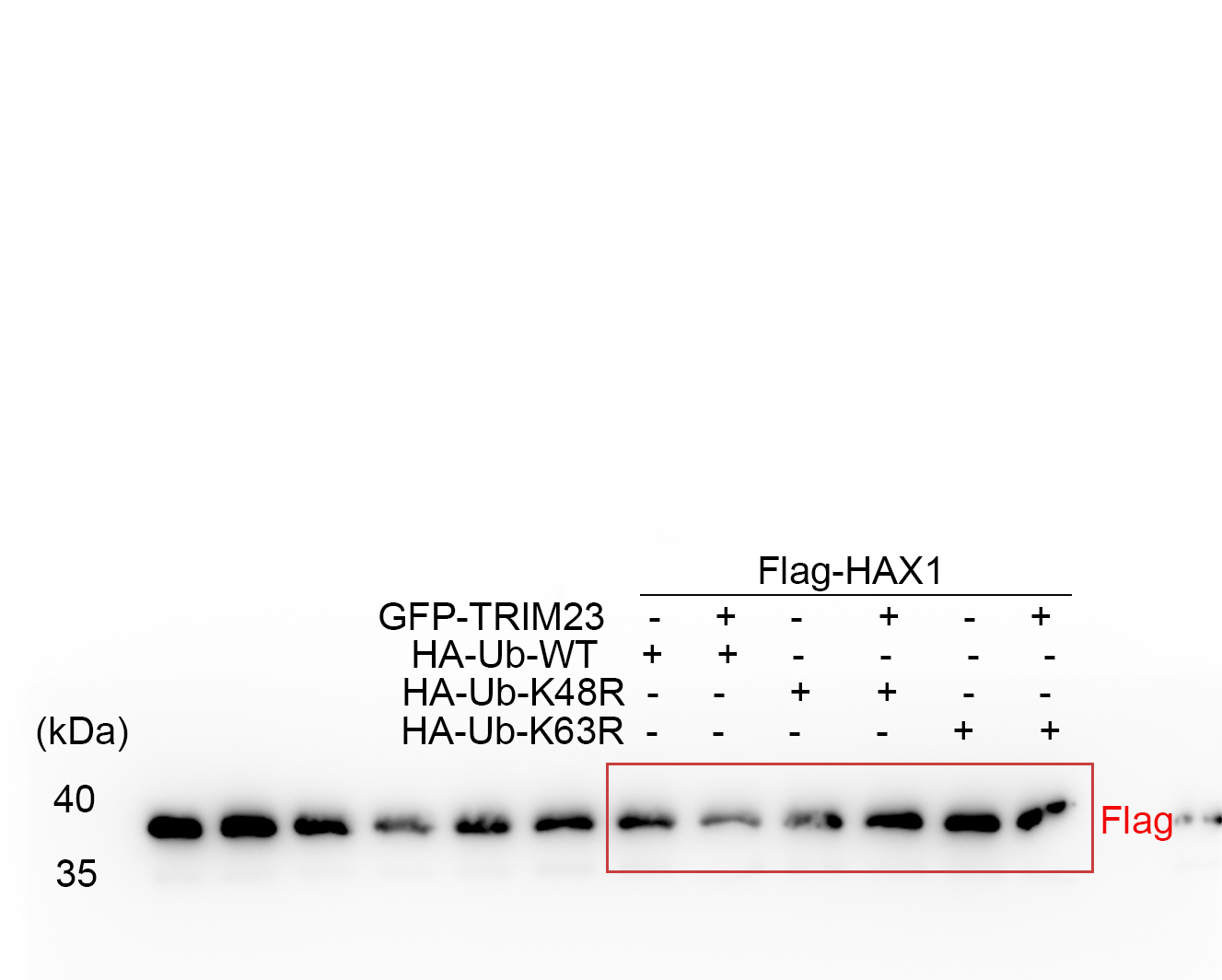

Supplement: Supplementary file 7 — Source data Fig. 4 [file 44318_2024_120_MOESM7_ESM.zip › Figure 4/4G/WCL/western-Flag-HAX1.Tif]

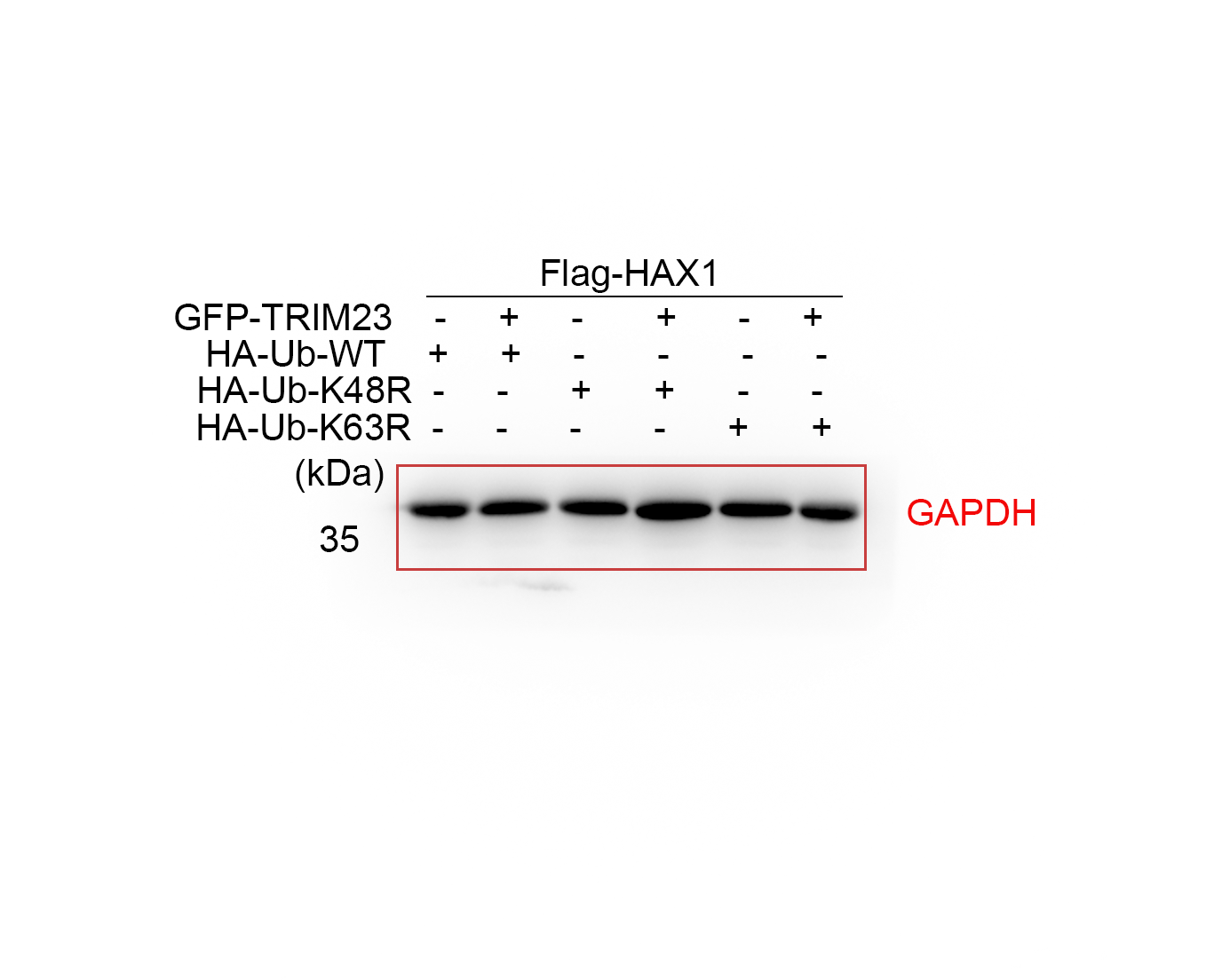

Supplement: Supplementary file 7 — Source data Fig. 4 [file 44318_2024_120_MOESM7_ESM.zip › Figure 4/4G/WCL/western-GAPDH.Tif]

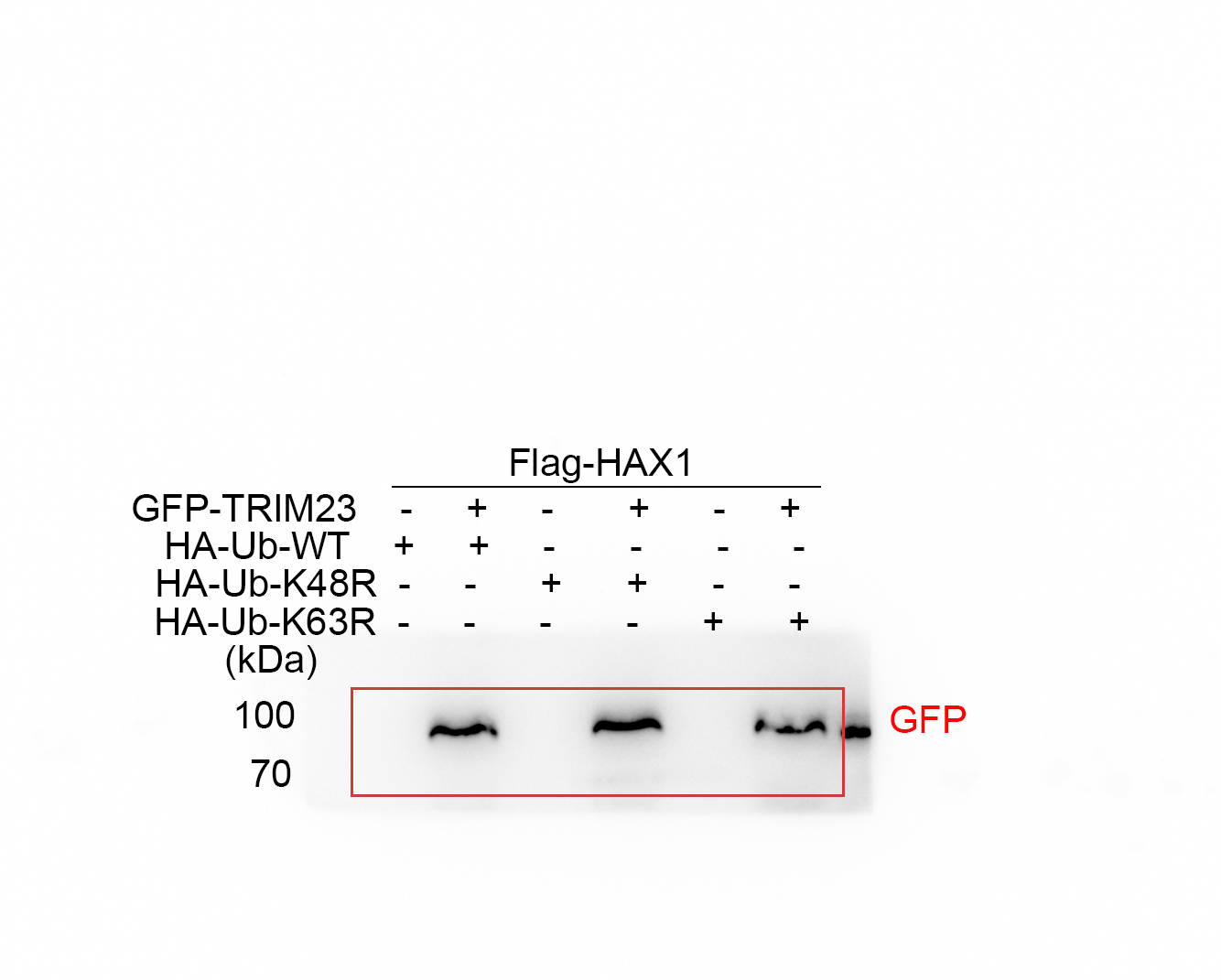

Supplement: Supplementary file 7 — Source data Fig. 4 [file 44318_2024_120_MOESM7_ESM.zip › Figure 4/4G/WCL/western-GFP-TRIM23.Tif]

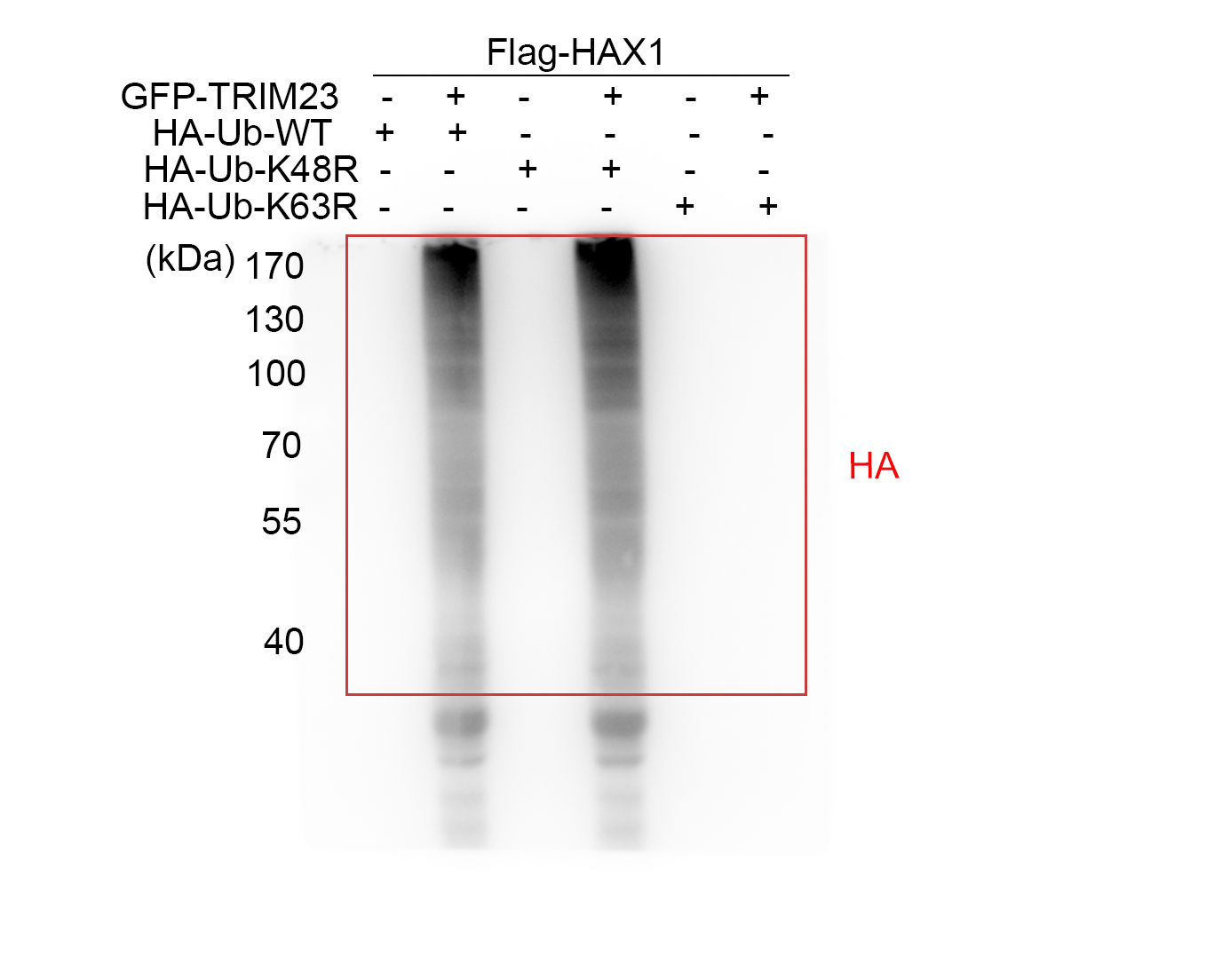

Supplement: Supplementary file 7 — Source data Fig. 4 [file 44318_2024_120_MOESM7_ESM.zip › Figure 4/4G/IP/western-Flag-HAX1-Ub.Tif]

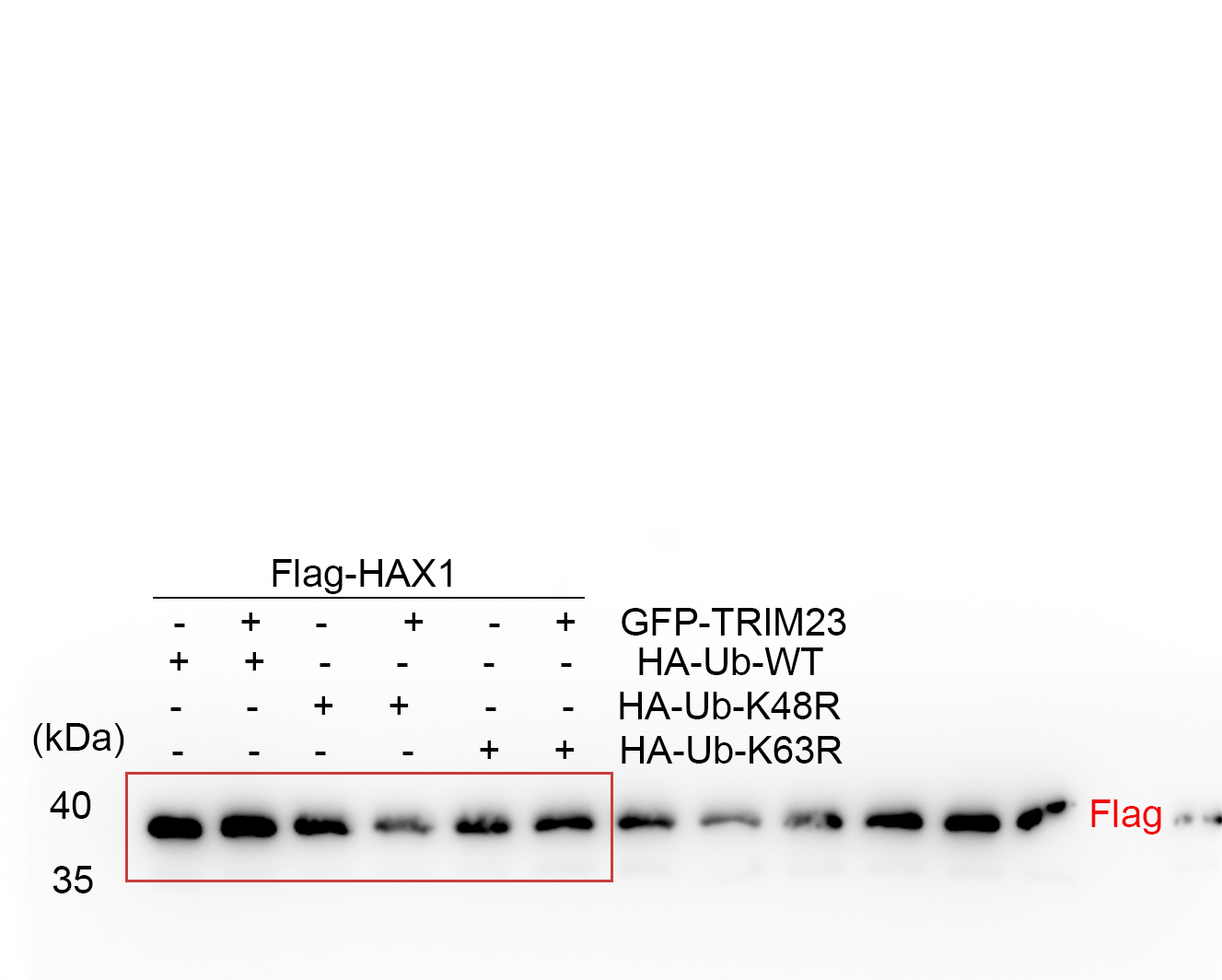

Supplement: Supplementary file 7 — Source data Fig. 4 [file 44318_2024_120_MOESM7_ESM.zip › Figure 4/4G/IP/western-Flag-HAX1.Tif]

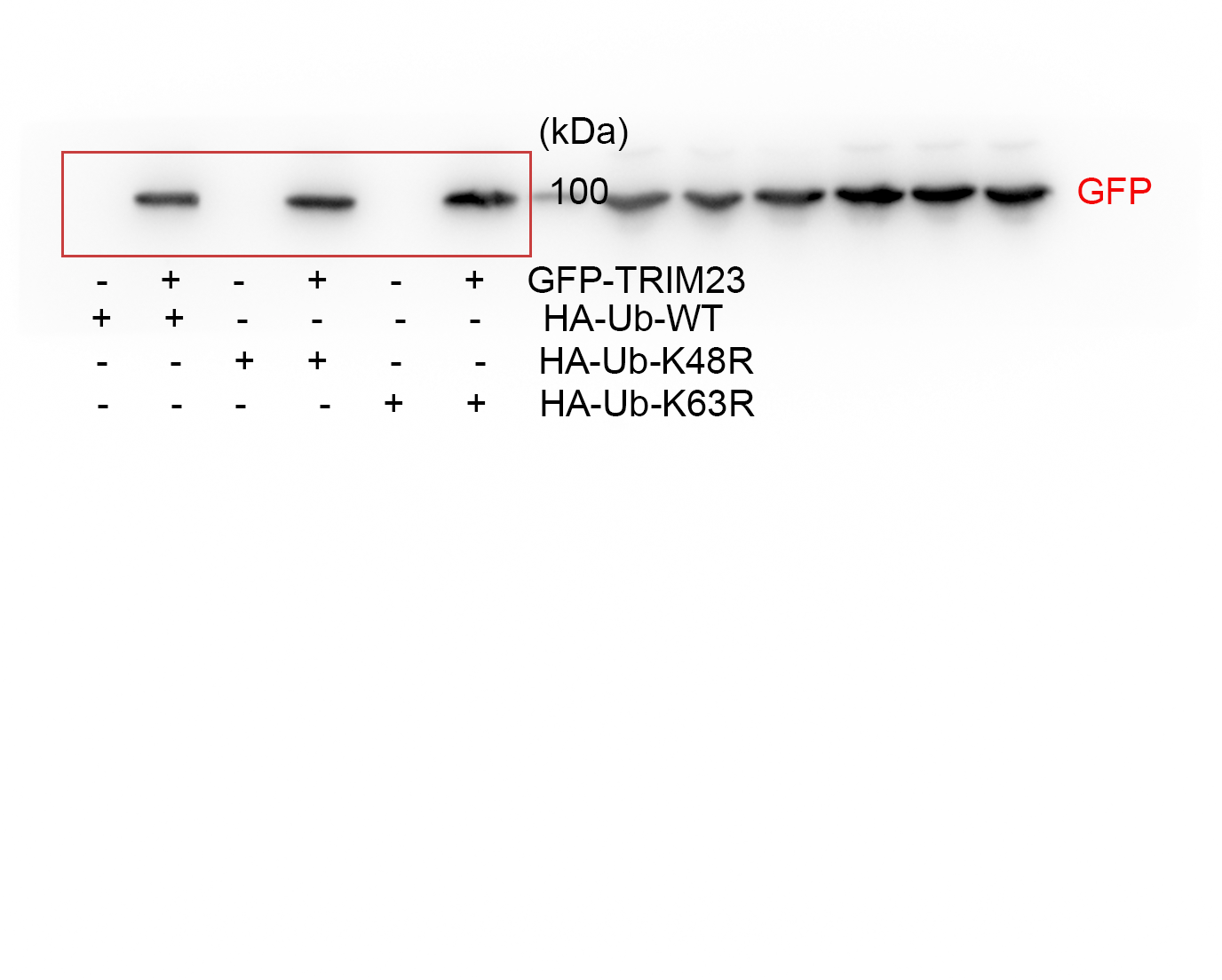

Supplement: Supplementary file 7 — Source data Fig. 4 [file 44318_2024_120_MOESM7_ESM.zip › Figure 4/4G/IP/western-GFP-TRIM23.Tif]

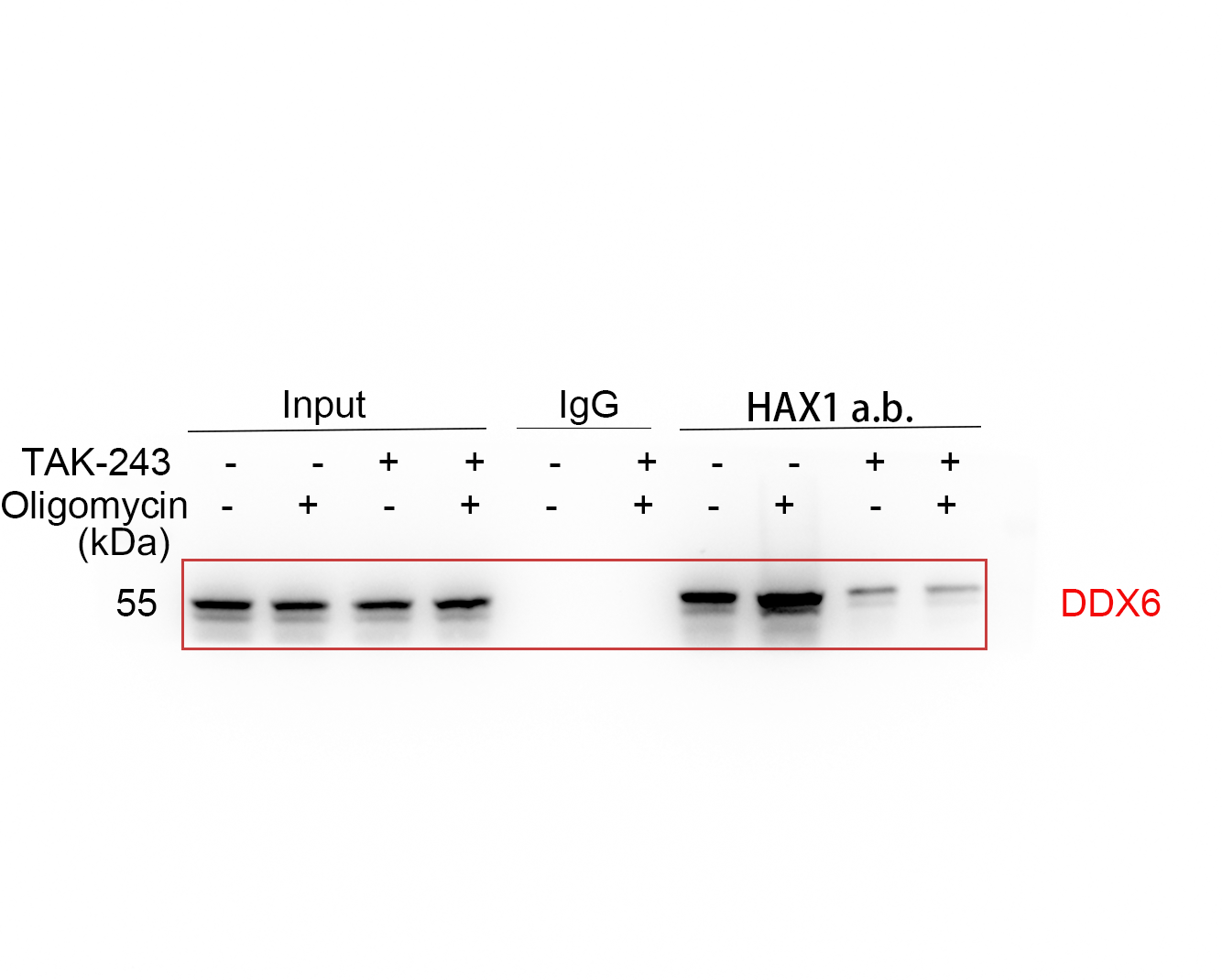

Supplement: Supplementary file 8 — Source data Fig. 5 [file 44318_2024_120_MOESM8_ESM.zip › Figure 5/5J/western-DDX6.Tif]

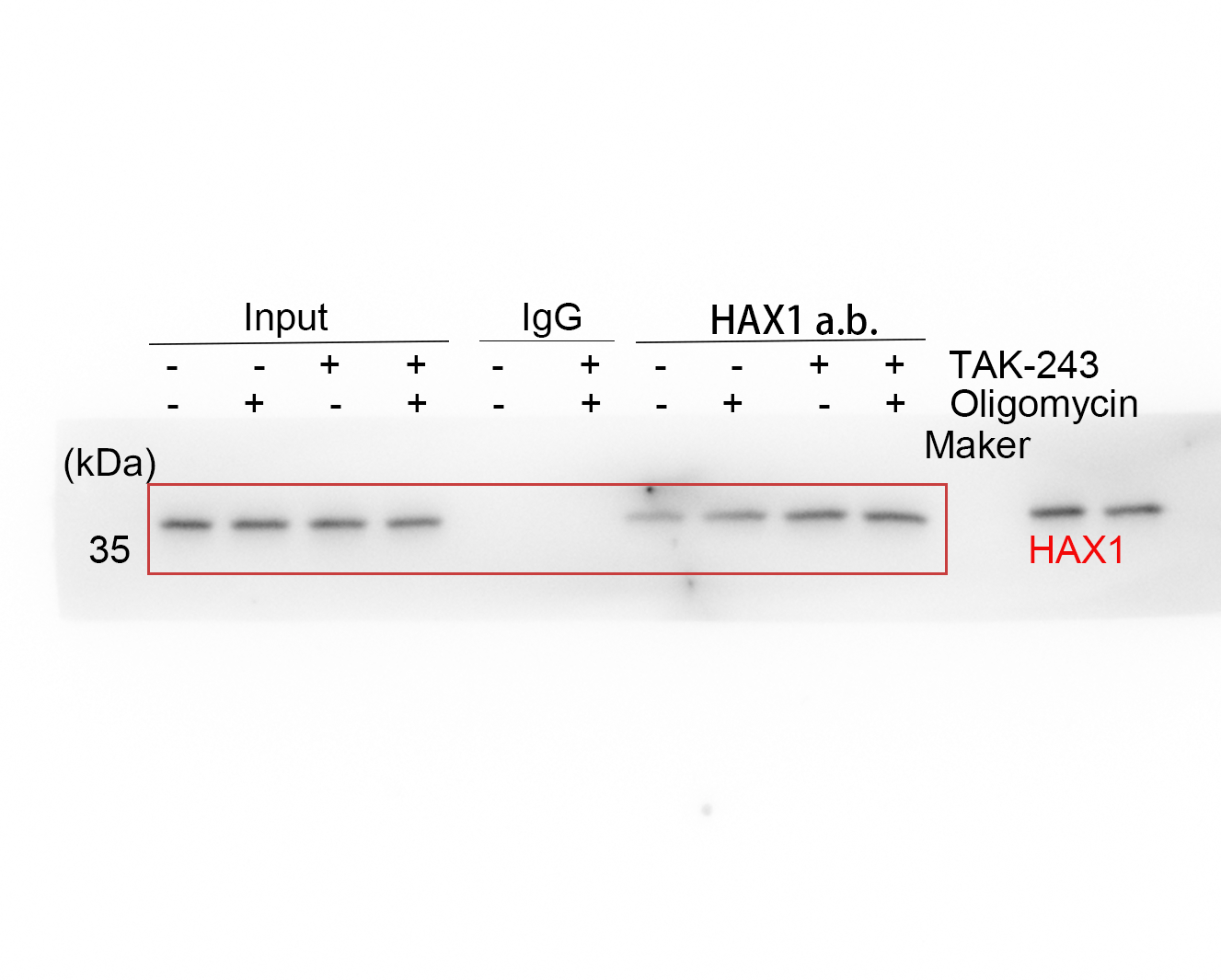

Supplement: Supplementary file 8 — Source data Fig. 5 [file 44318_2024_120_MOESM8_ESM.zip › Figure 5/5J/western-HAX1.Tif]

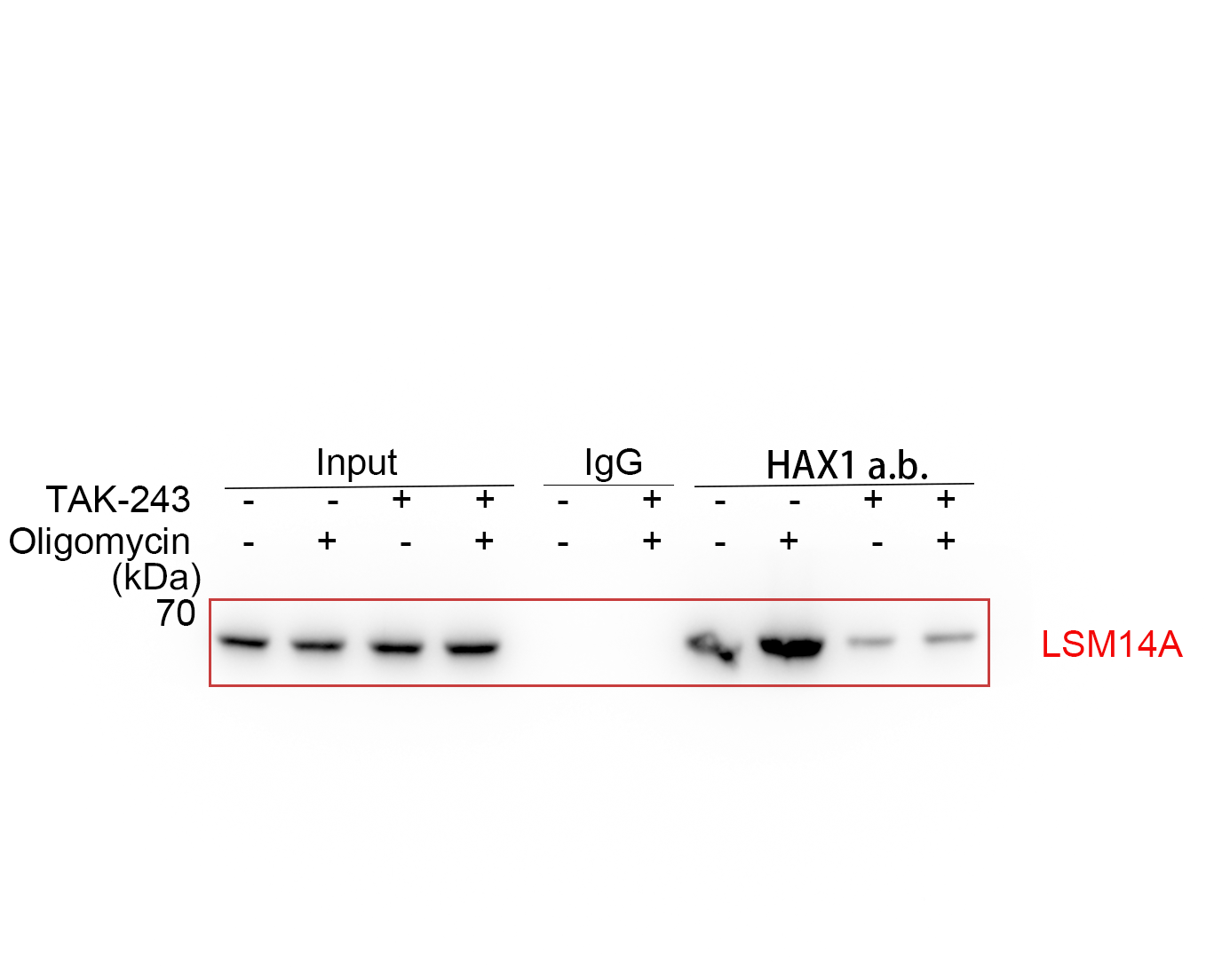

Supplement: Supplementary file 8 — Source data Fig. 5 [file 44318_2024_120_MOESM8_ESM.zip › Figure 5/5J/western-LSM14A.Tif]

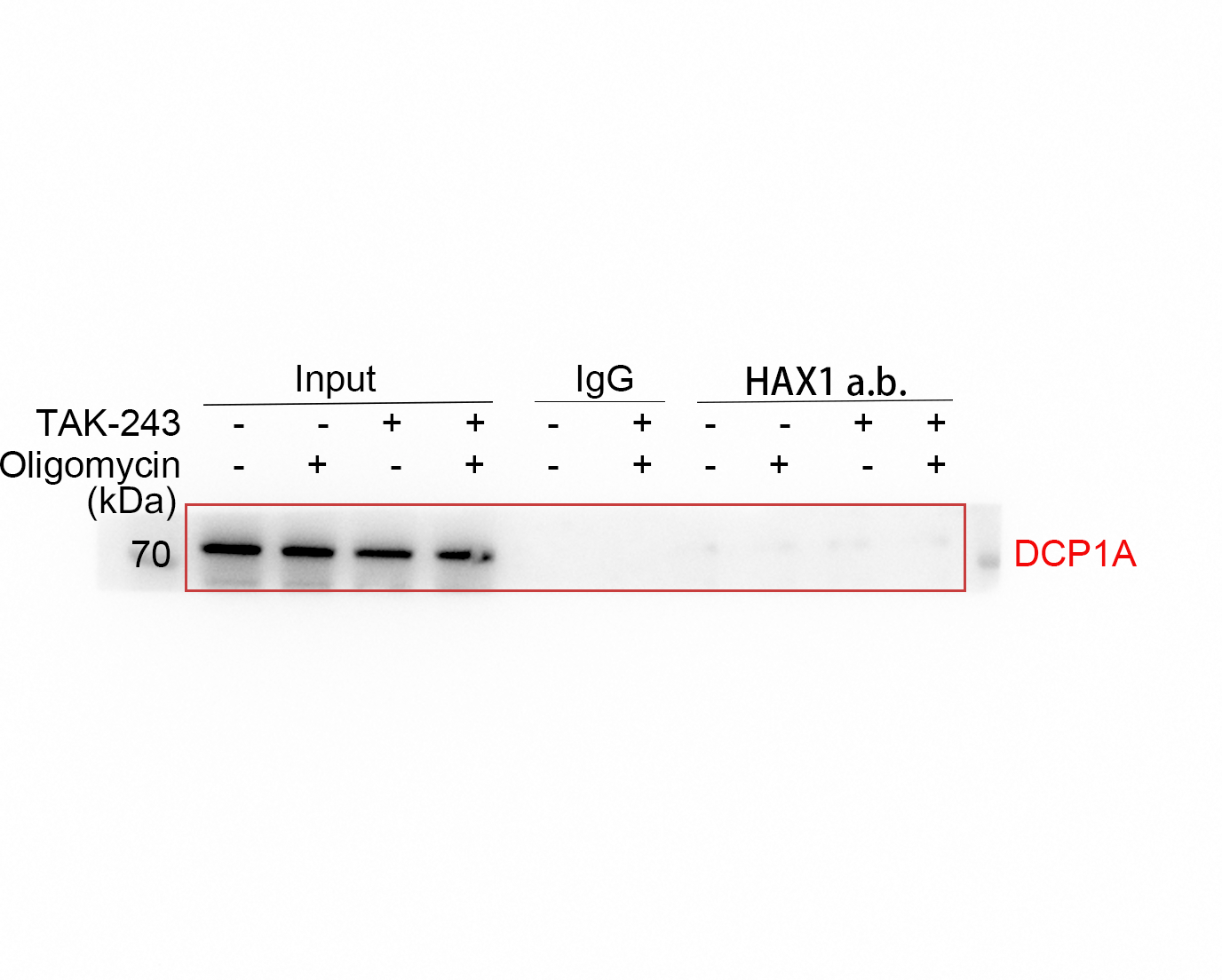

Supplement: Supplementary file 8 — Source data Fig. 5 [file 44318_2024_120_MOESM8_ESM.zip › Figure 5/5J/western-DCP1A.Tif]

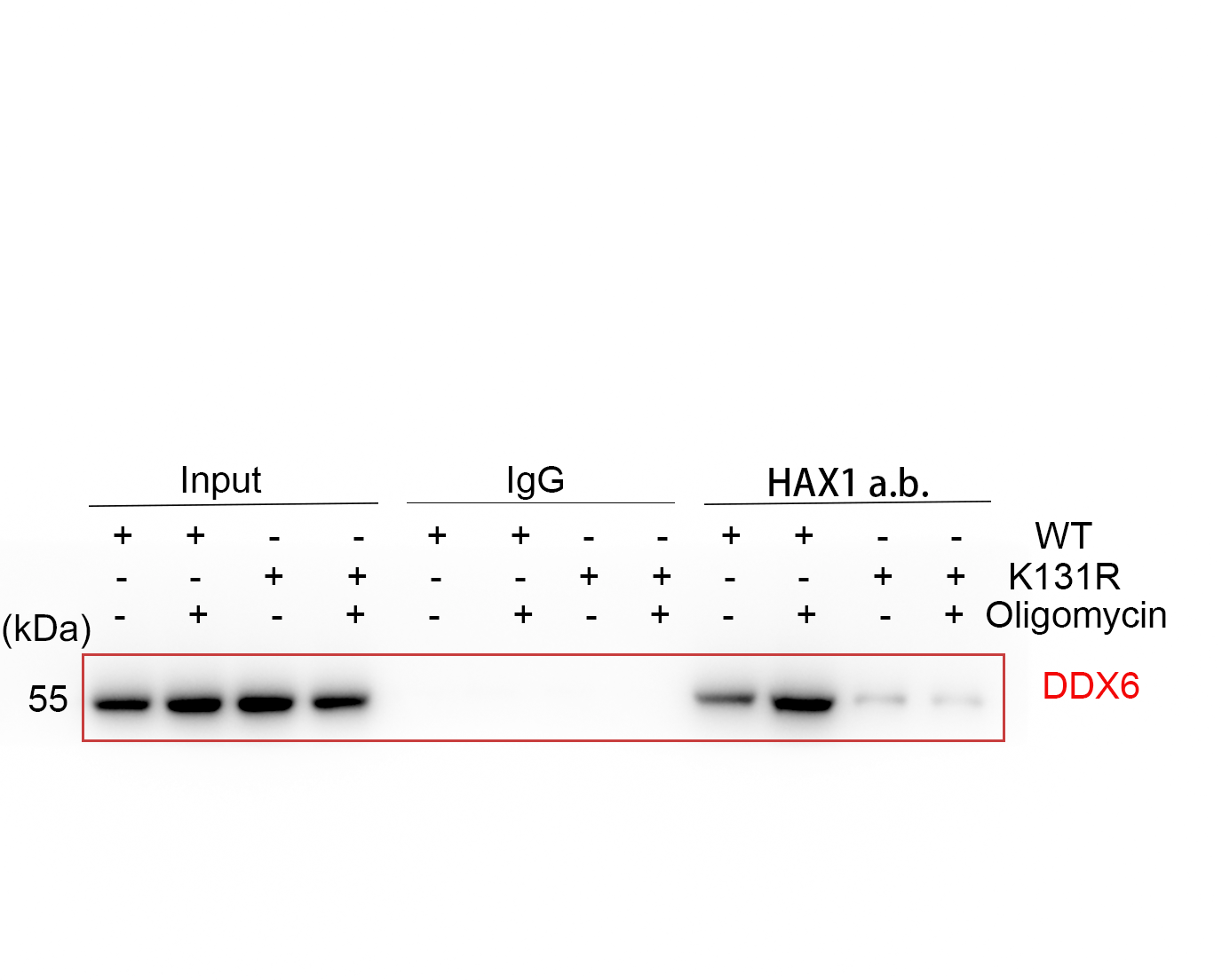

Supplement: Supplementary file 8 — Source data Fig. 5 [file 44318_2024_120_MOESM8_ESM.zip › Figure 5/5K/western-DDX6.Tif]

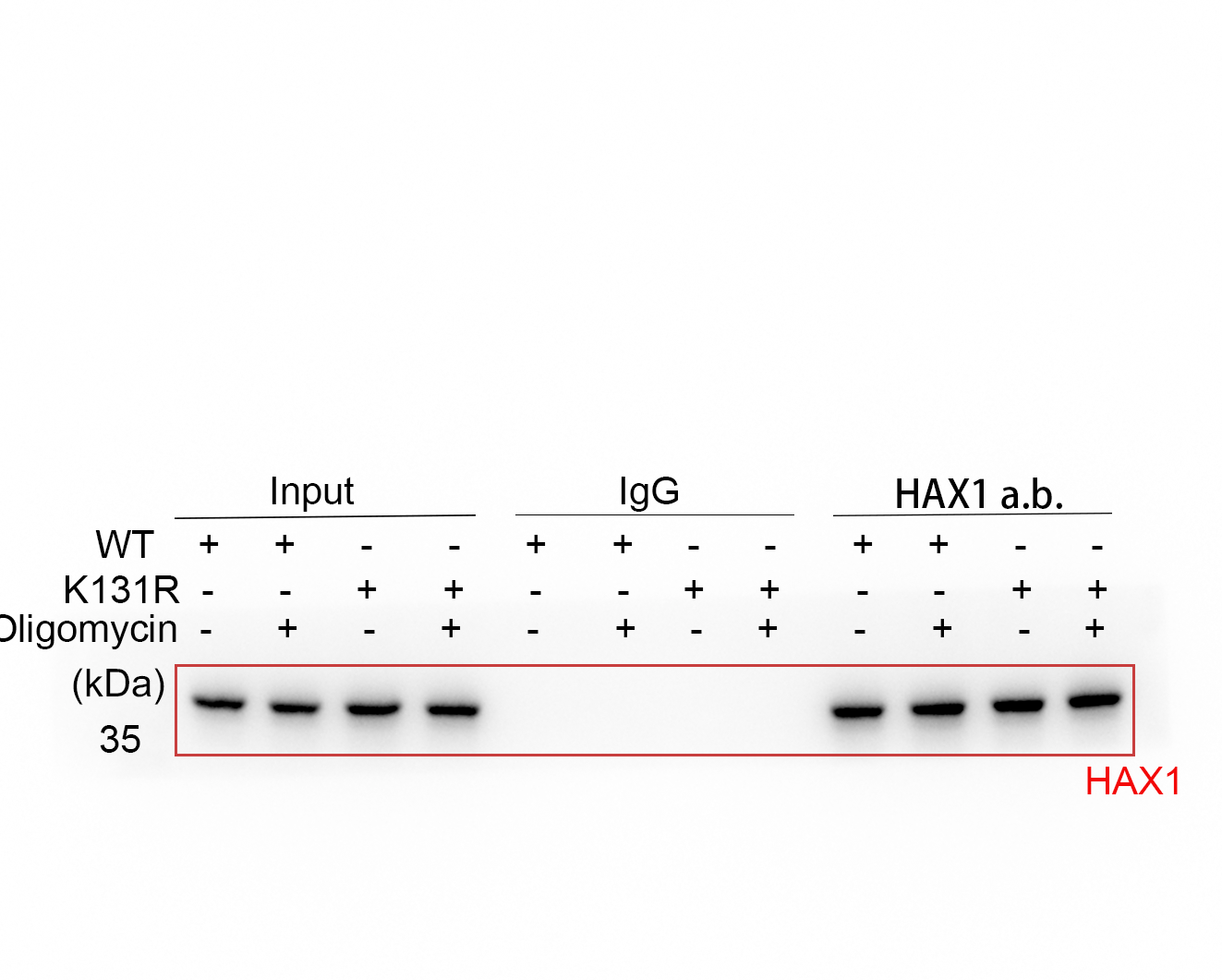

Supplement: Supplementary file 8 — Source data Fig. 5 [file 44318_2024_120_MOESM8_ESM.zip › Figure 5/5K/western-HAX1.Tif]

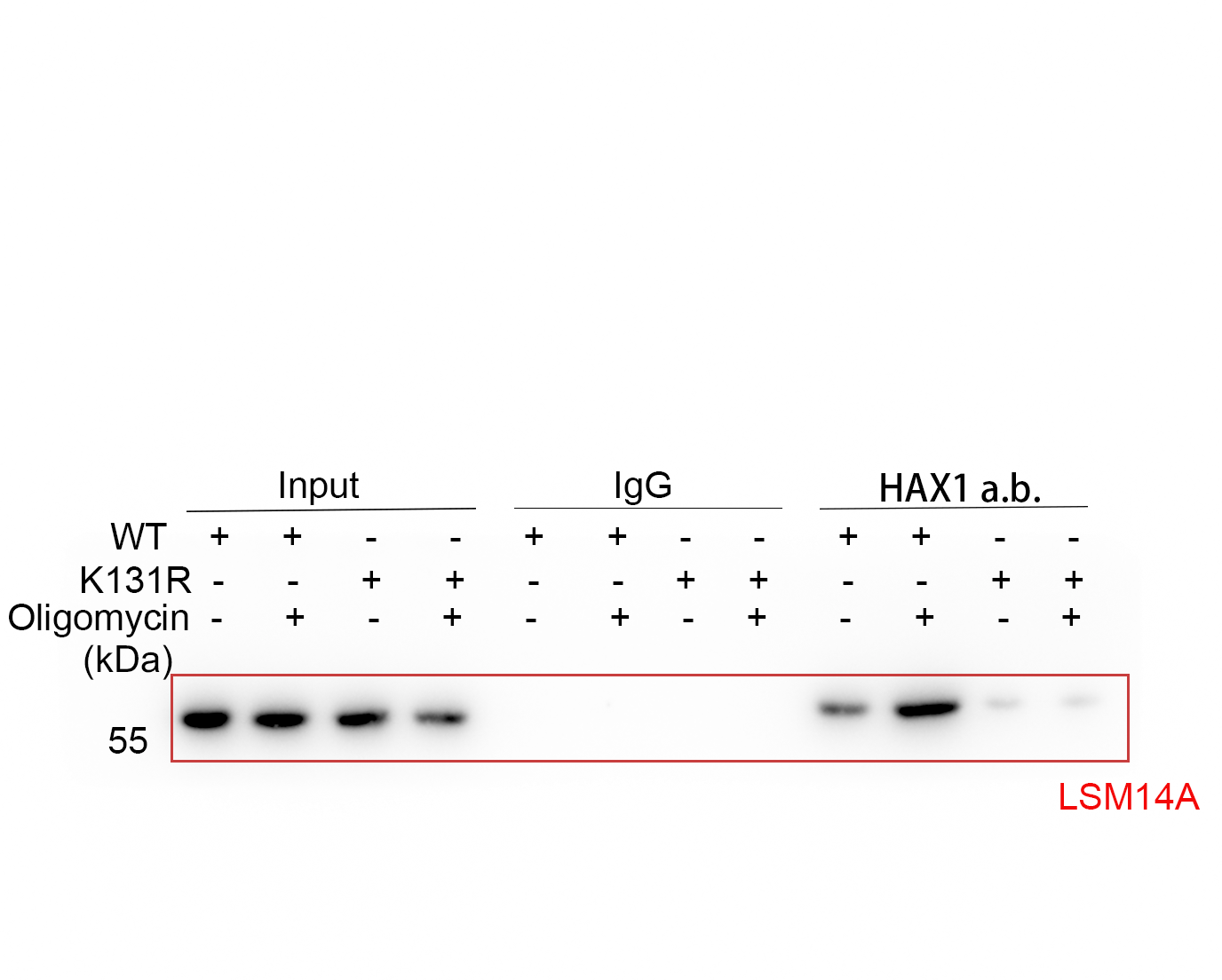

Supplement: Supplementary file 8 — Source data Fig. 5 [file 44318_2024_120_MOESM8_ESM.zip › Figure 5/5K/western-LSM14A.Tif]

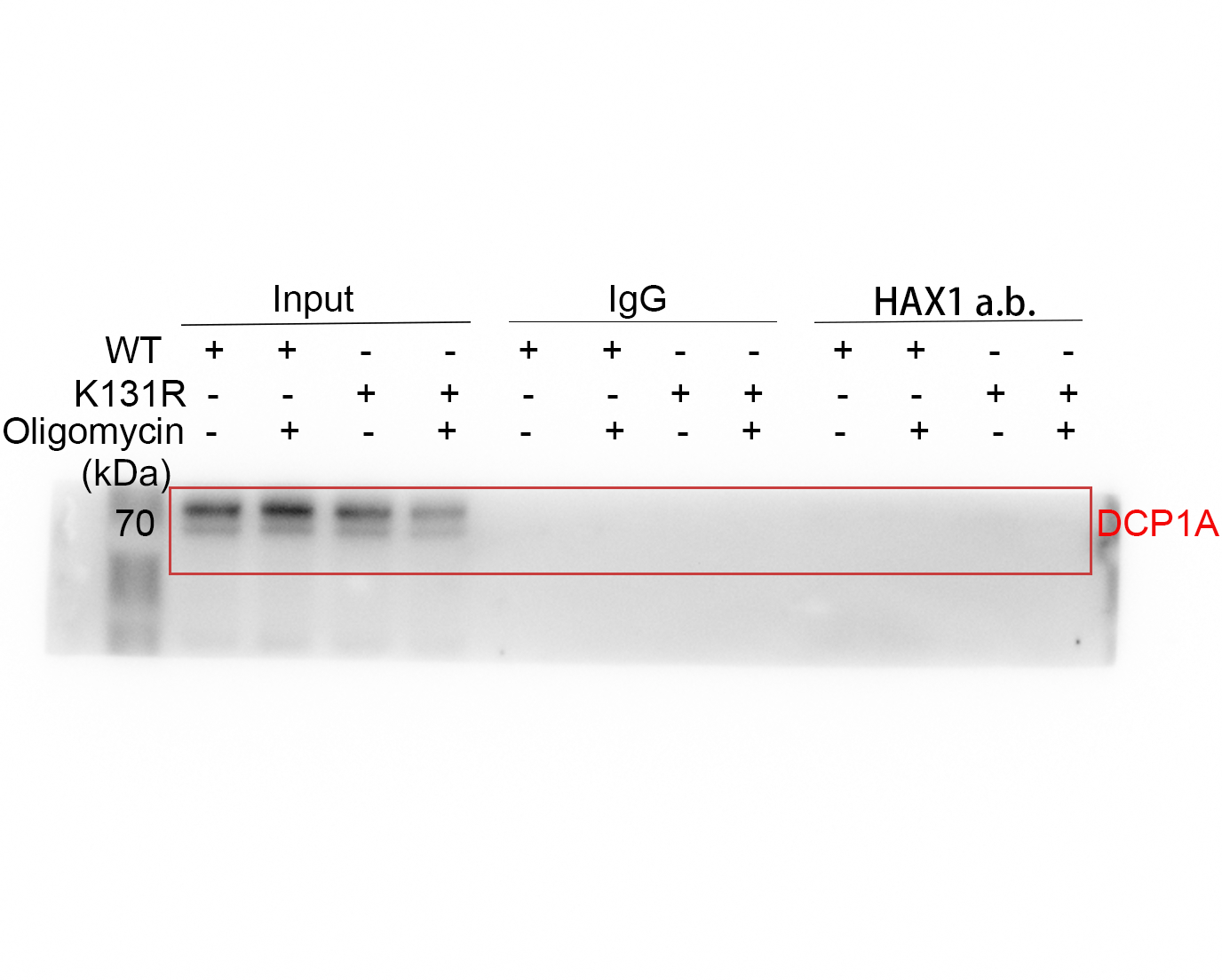

Supplement: Supplementary file 8 — Source data Fig. 5 [file 44318_2024_120_MOESM8_ESM.zip › Figure 5/5K/western-DCP1A.Tif]

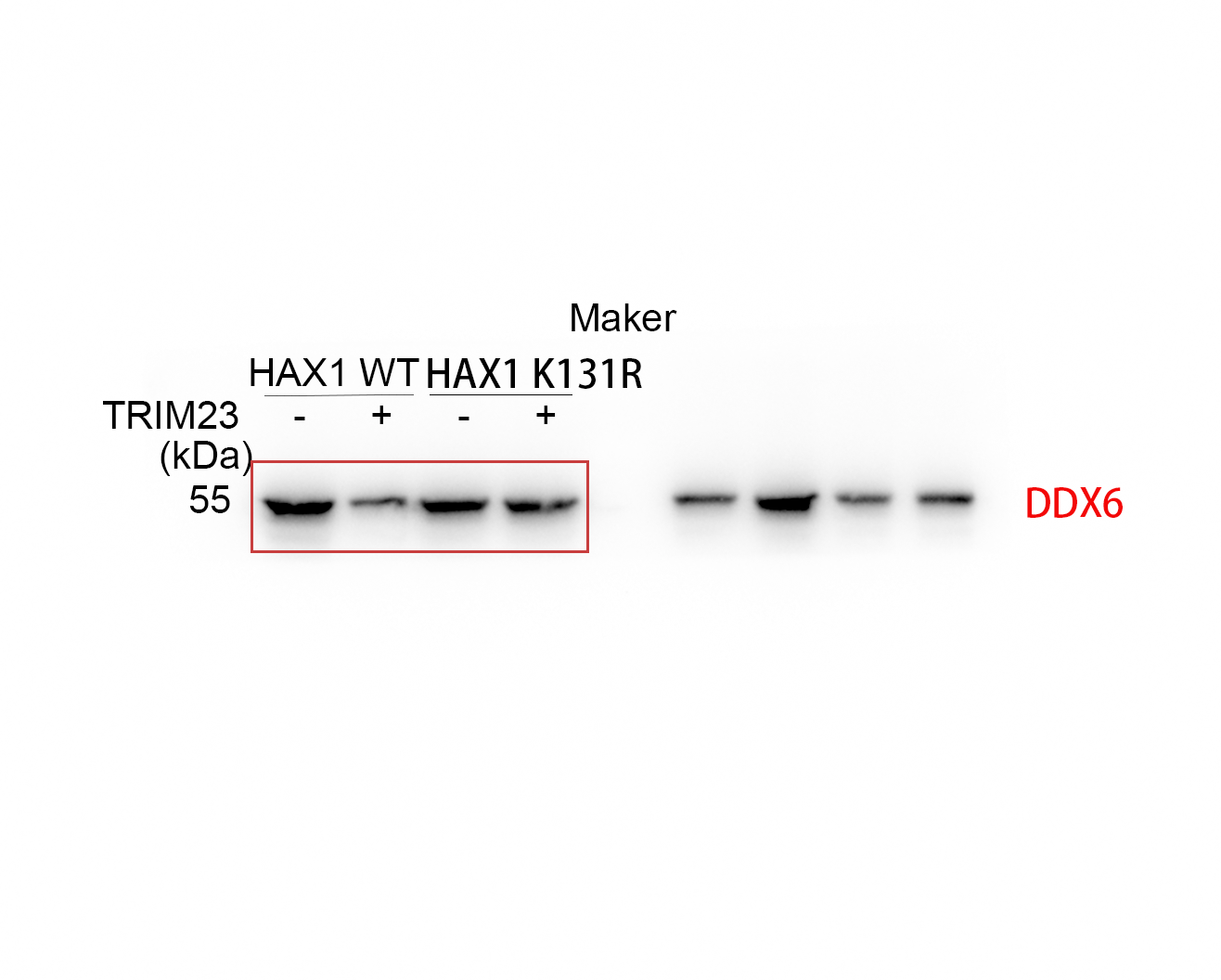

Supplement: Supplementary file 8 — Source data Fig. 5 [file 44318_2024_120_MOESM8_ESM.zip › Figure 5/5I/S2/western-DDX6.Tif]

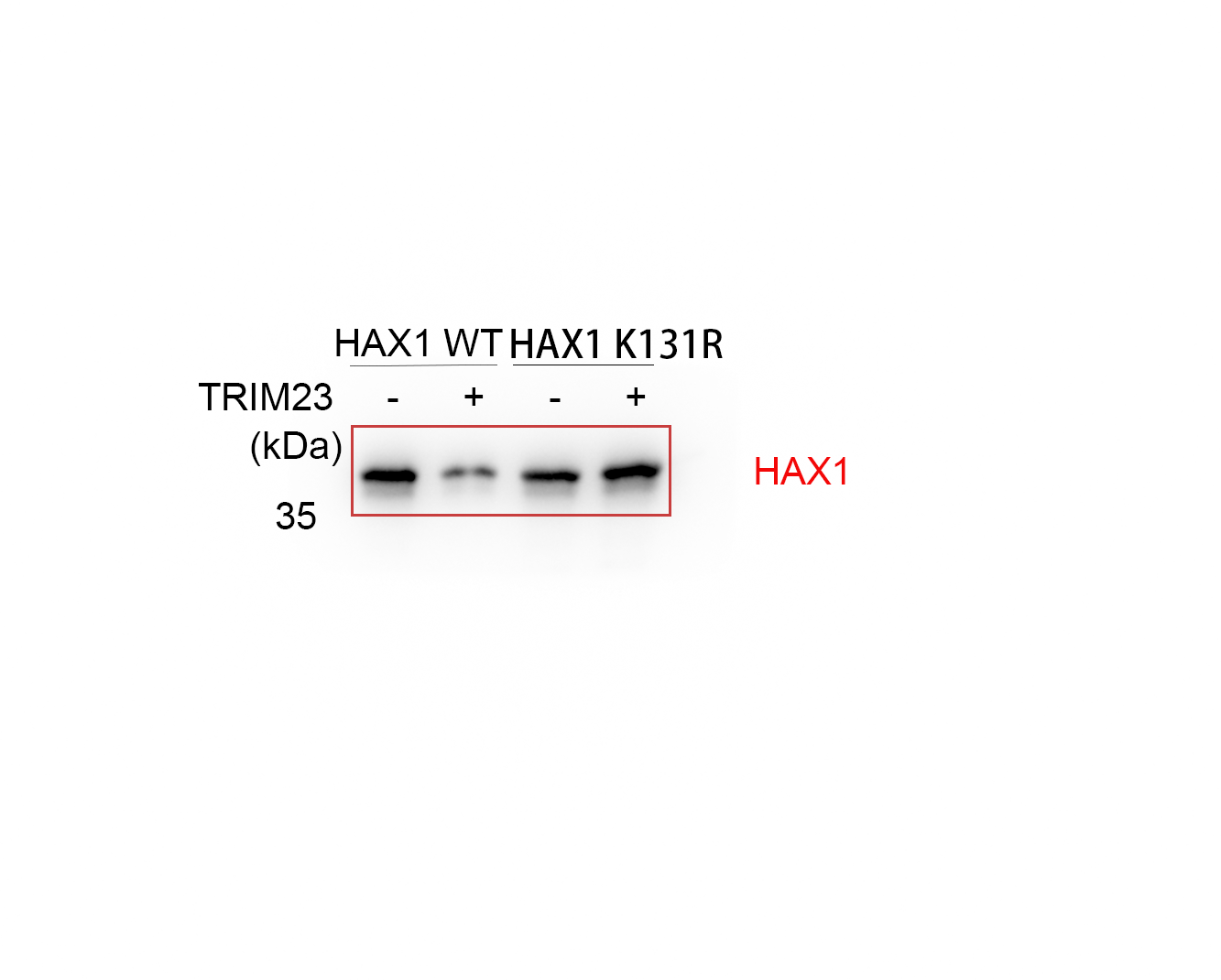

Supplement: Supplementary file 8 — Source data Fig. 5 [file 44318_2024_120_MOESM8_ESM.zip › Figure 5/5I/S2/western-HAX1.Tif]

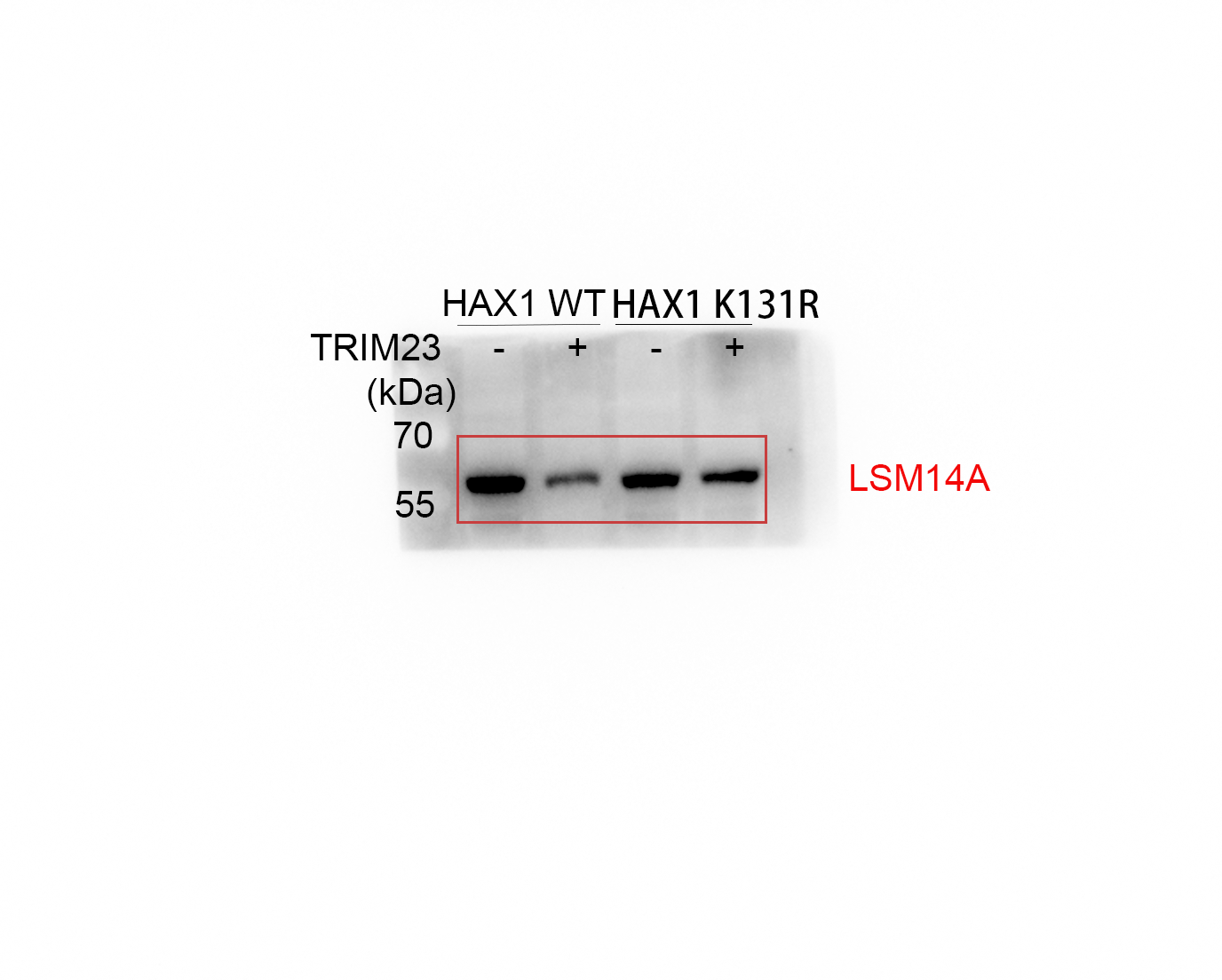

Supplement: Supplementary file 8 — Source data Fig. 5 [file 44318_2024_120_MOESM8_ESM.zip › Figure 5/5I/S2/western-LSM14A.Tif]
