# Supplementary figures and images for: Energy stress promotes P-bodies formation via lysine-63-linked polyubiquitination of HAX1 (part 3 of 4)
Source: EMBO J. 2024 May 20;43(13):11. doi: 10.1038/s44318-024-00120-6 (PMC11217408; doi:10.1038/s44318-024-00120-6)

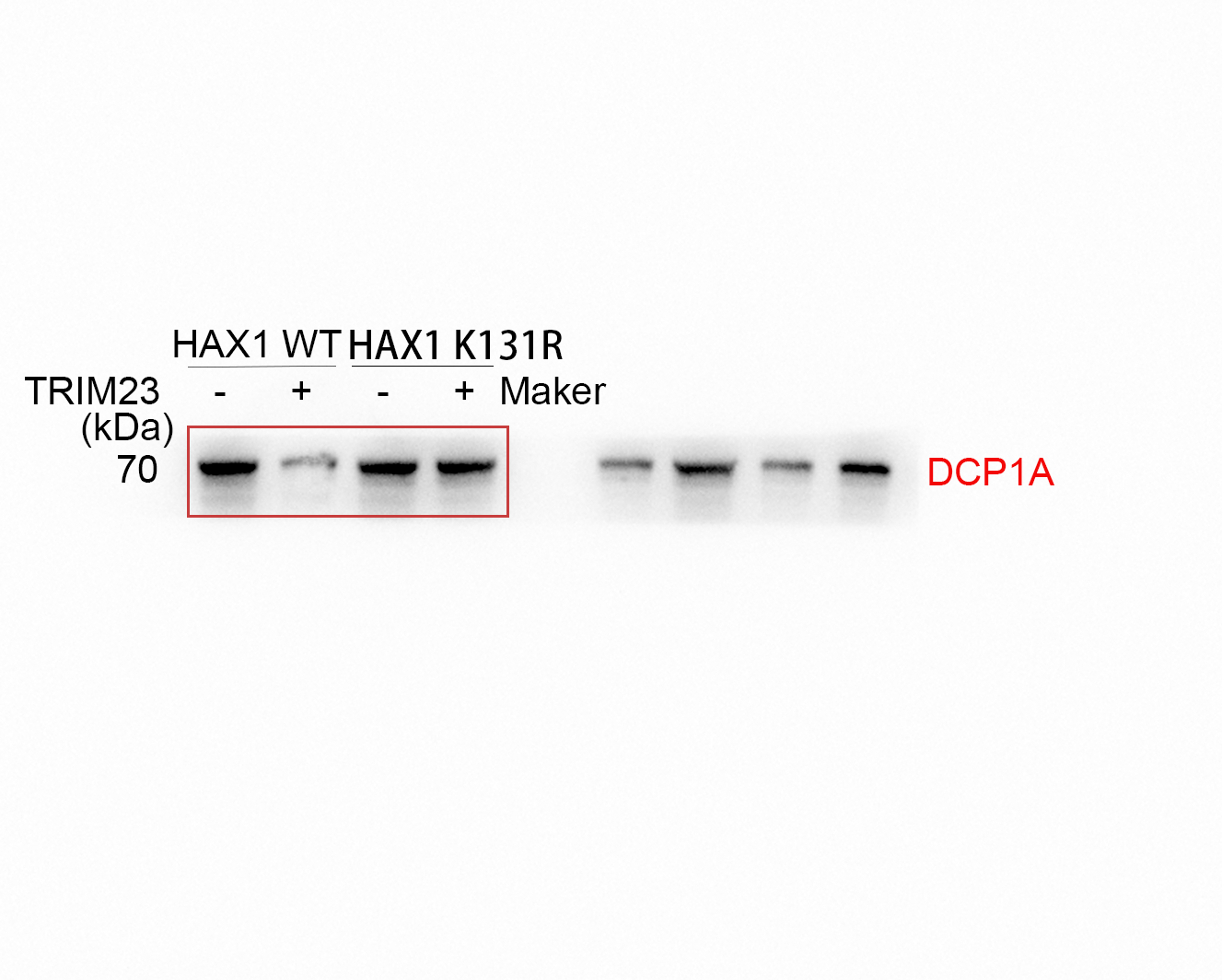

Supplement: Supplementary file 8 — Source data Fig. 5 [file 44318_2024_120_MOESM8_ESM.zip › Figure 5/5I/S2/western-DCP1A.Tif]

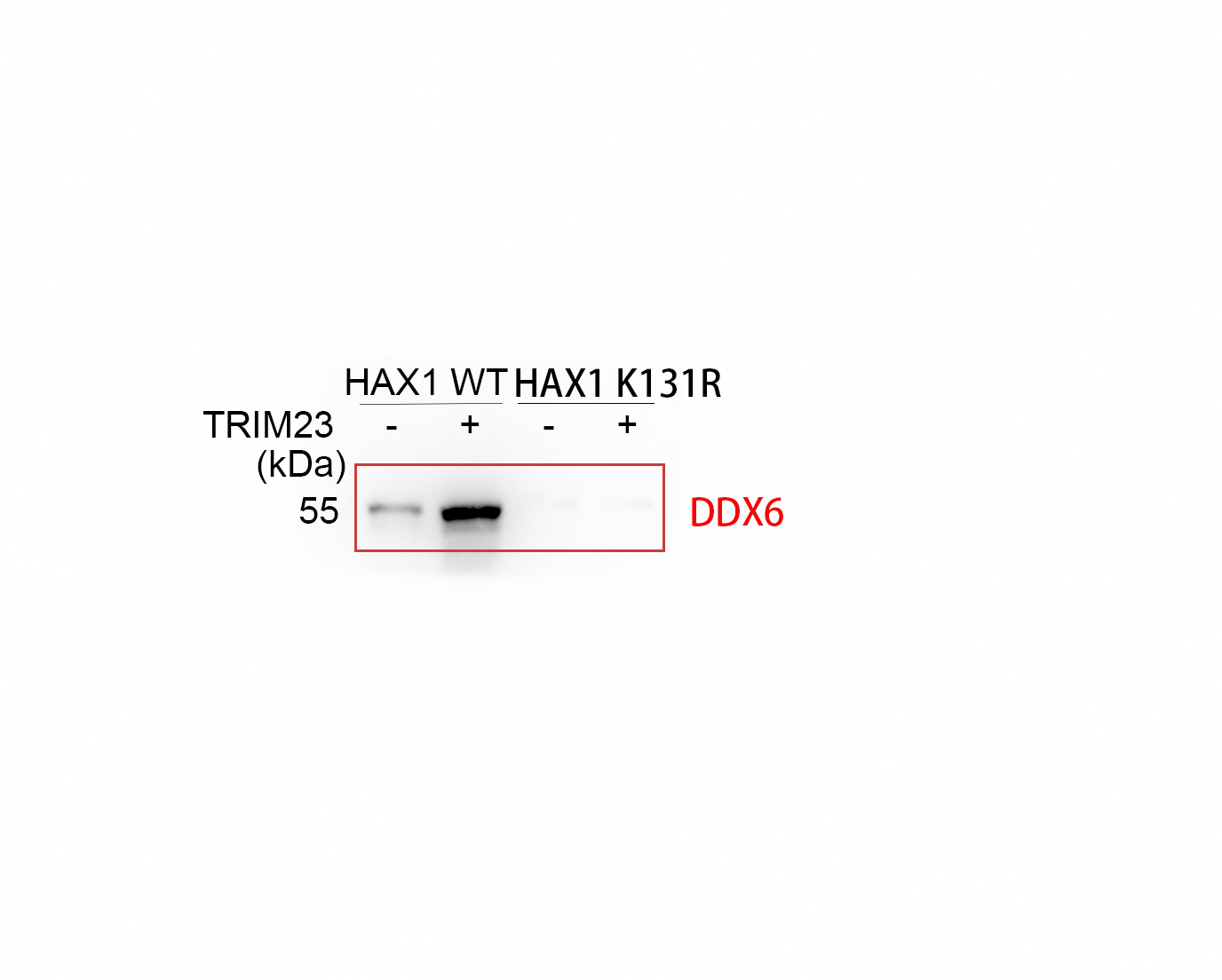

Supplement: Supplementary file 8 — Source data Fig. 5 [file 44318_2024_120_MOESM8_ESM.zip › Figure 5/5I/P2/western-DDX6.Tif]

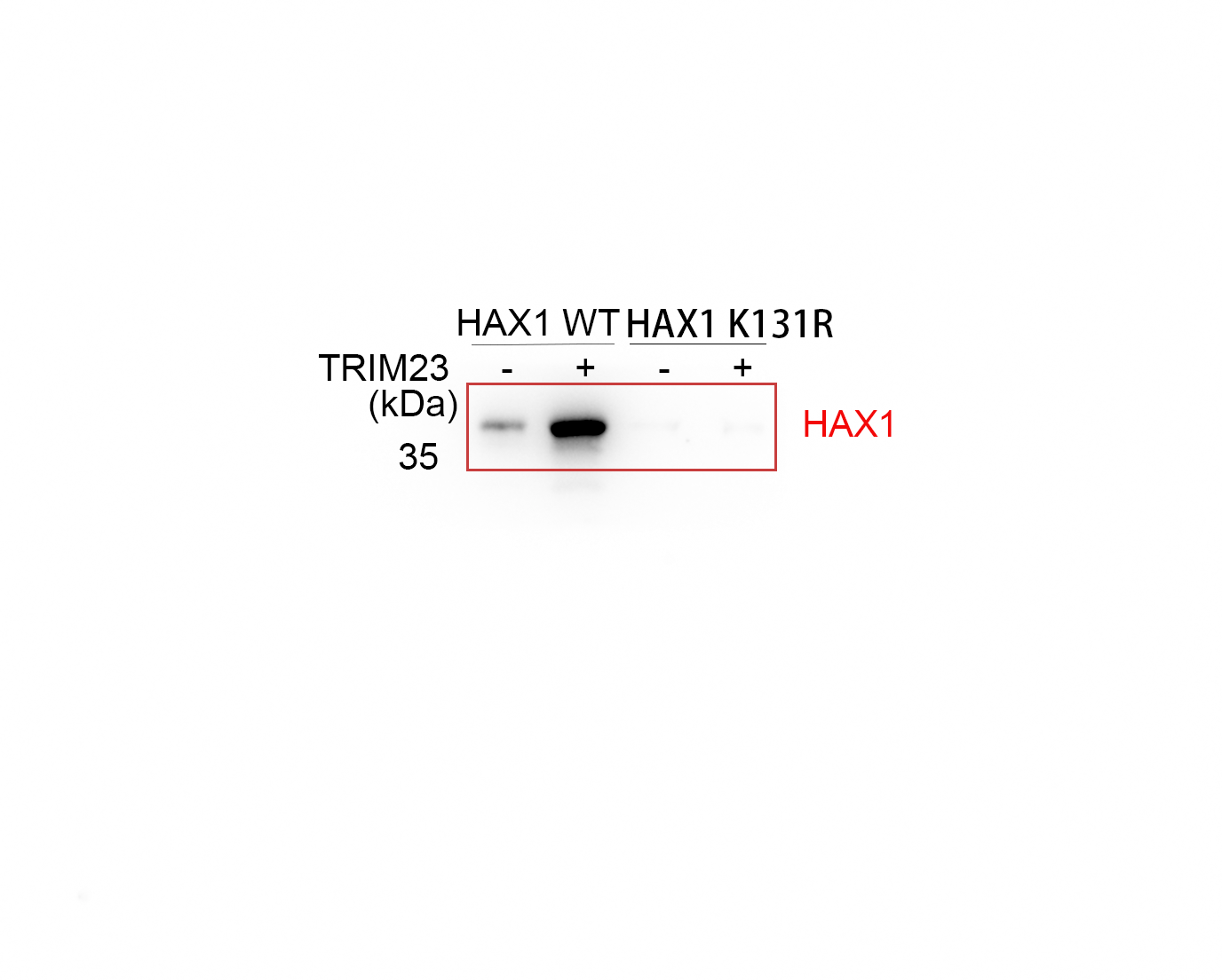

Supplement: Supplementary file 8 — Source data Fig. 5 [file 44318_2024_120_MOESM8_ESM.zip › Figure 5/5I/P2/western-HAX1.Tif]

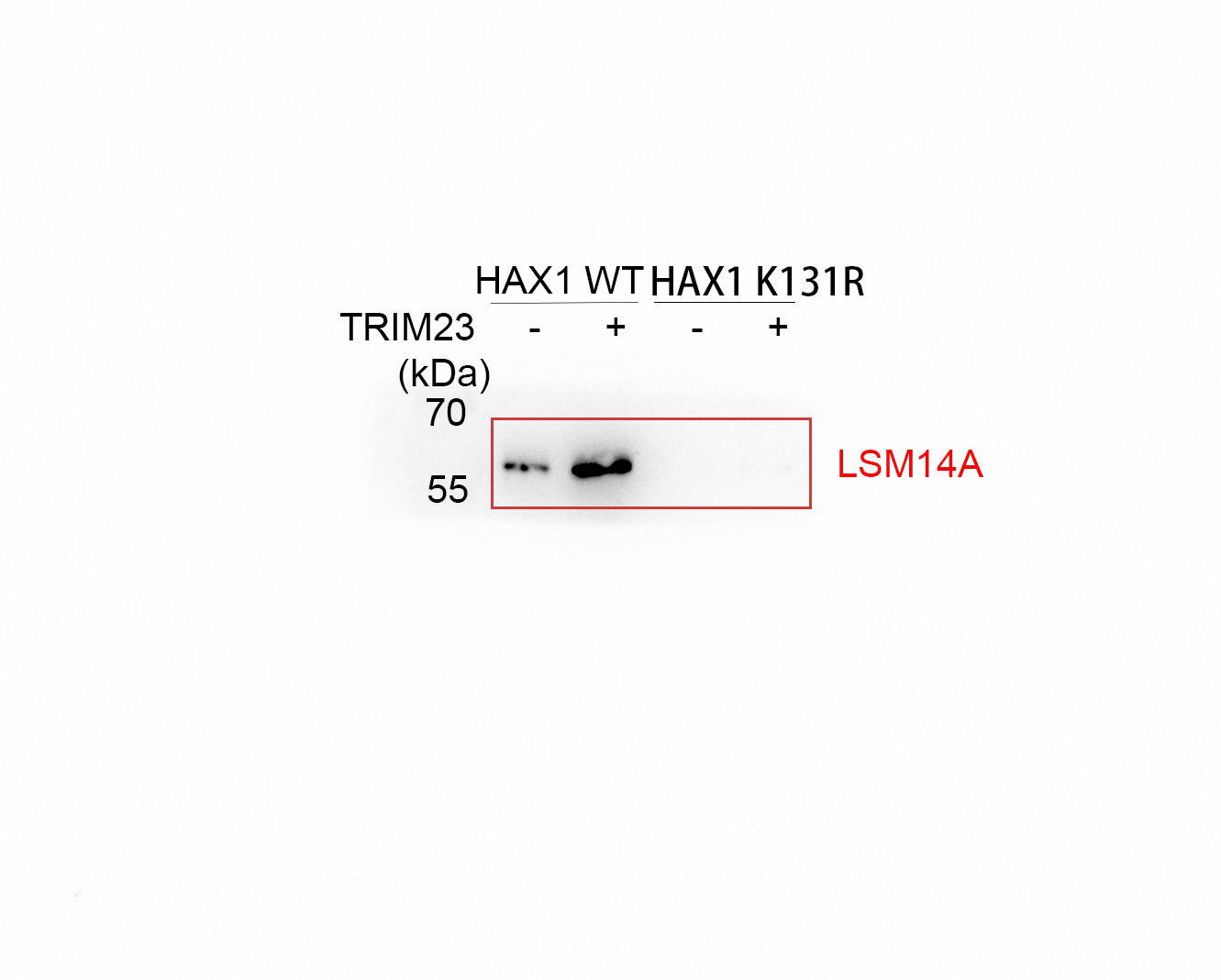

Supplement: Supplementary file 8 — Source data Fig. 5 [file 44318_2024_120_MOESM8_ESM.zip › Figure 5/5I/P2/western-LSM14A.Tif]

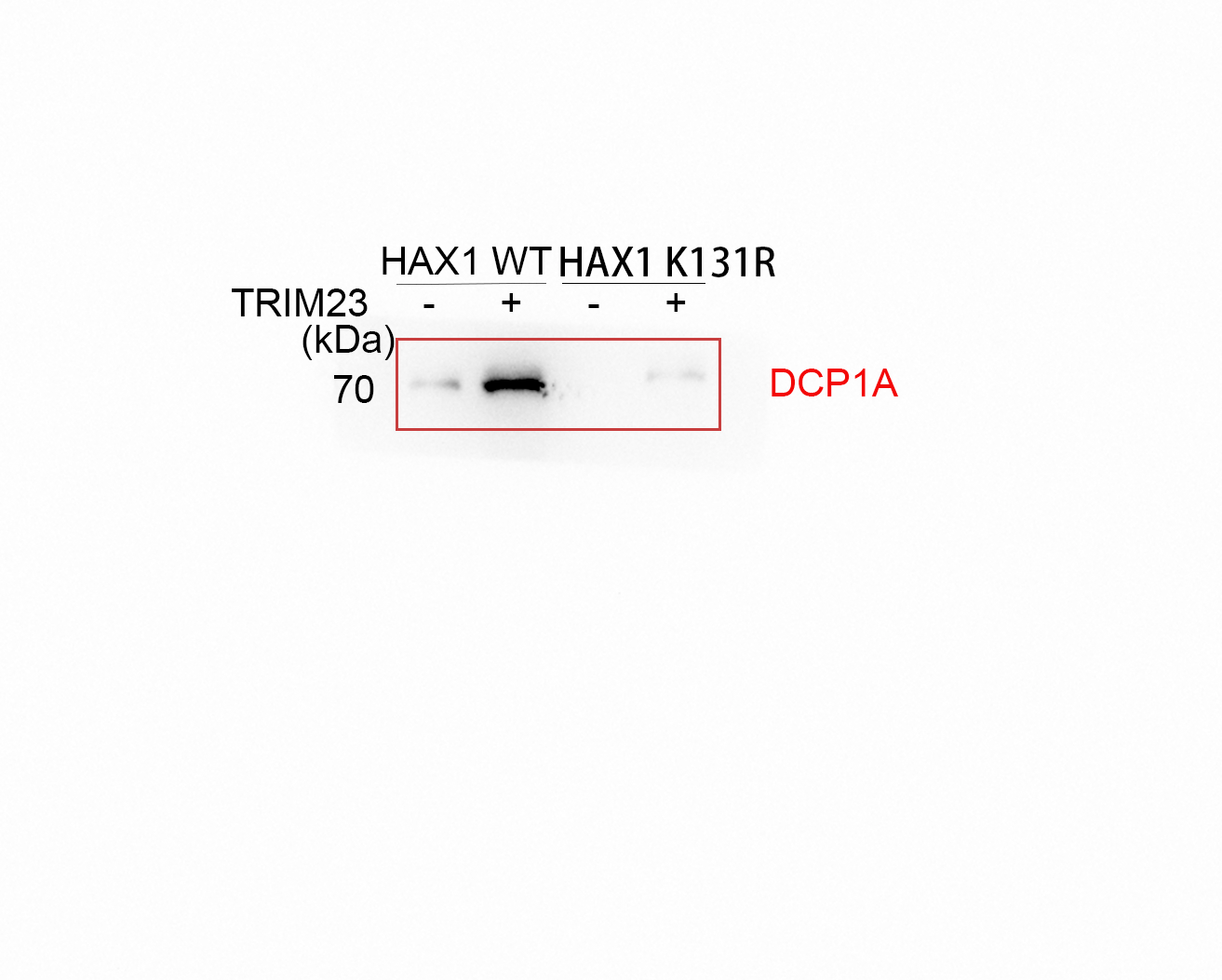

Supplement: Supplementary file 8 — Source data Fig. 5 [file 44318_2024_120_MOESM8_ESM.zip › Figure 5/5I/P2/western-DCP1A.Tif]

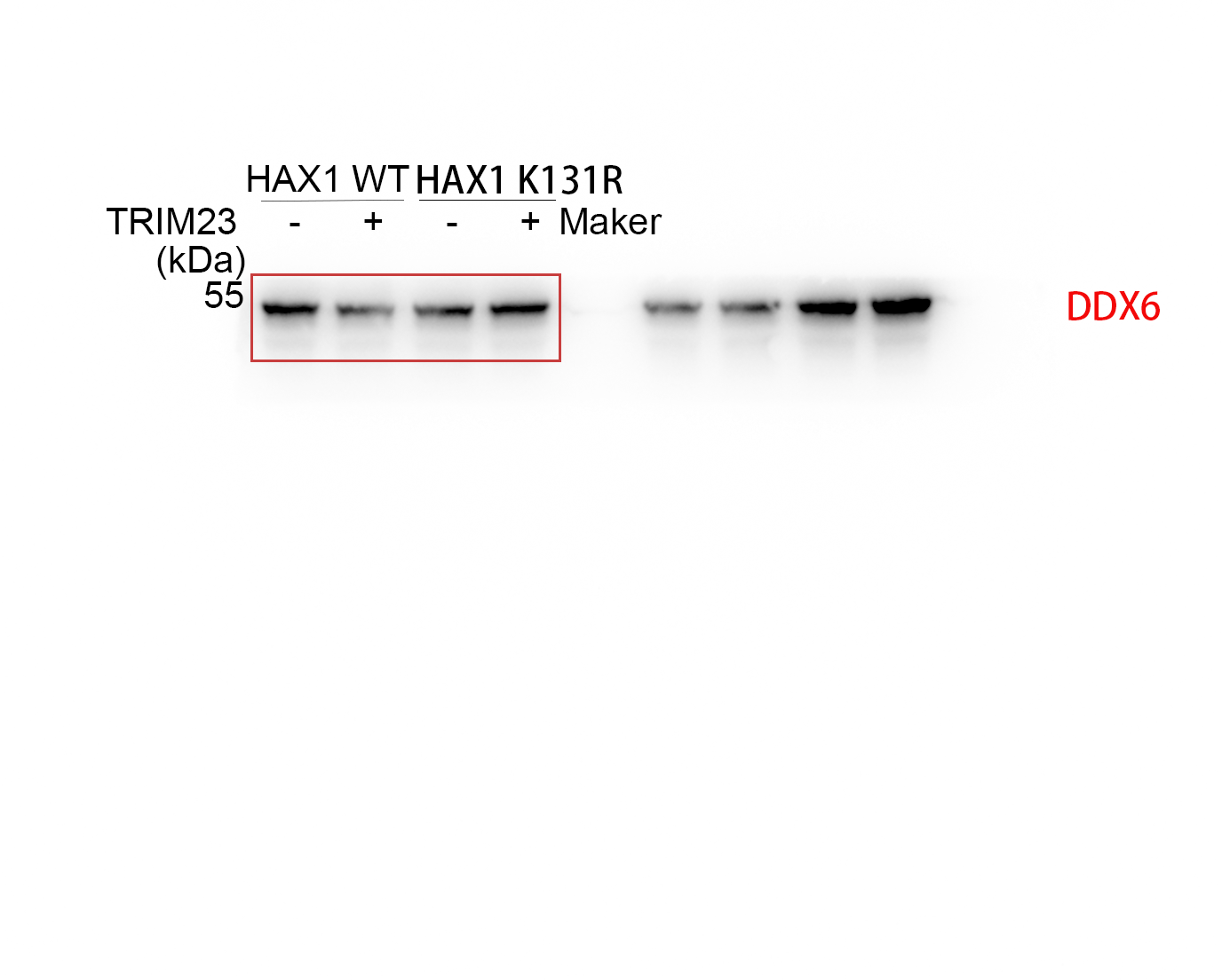

Supplement: Supplementary file 8 — Source data Fig. 5 [file 44318_2024_120_MOESM8_ESM.zip › Figure 5/5I/WCL/western-DDX6.Tif]

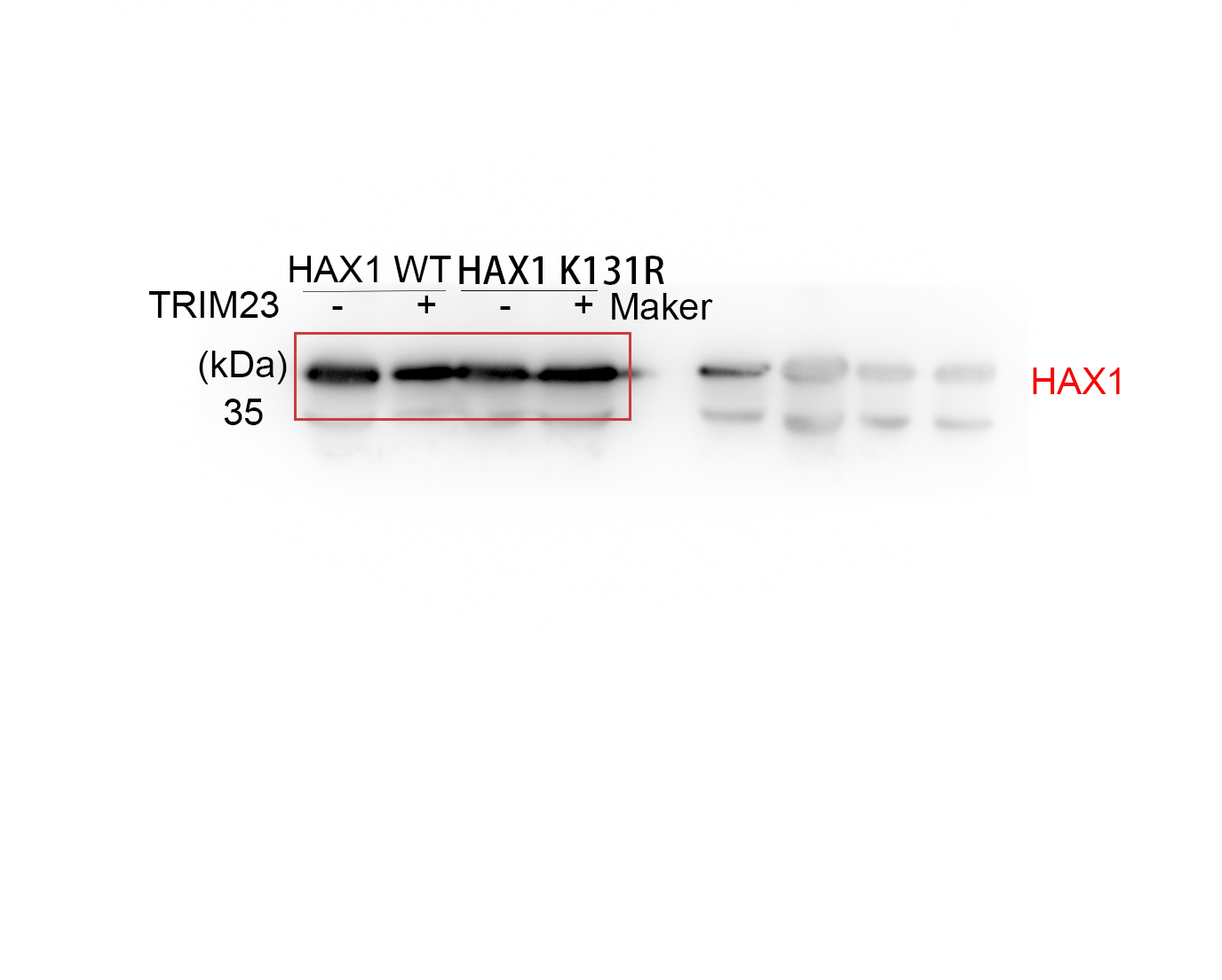

Supplement: Supplementary file 8 — Source data Fig. 5 [file 44318_2024_120_MOESM8_ESM.zip › Figure 5/5I/WCL/western-HAX1.Tif]

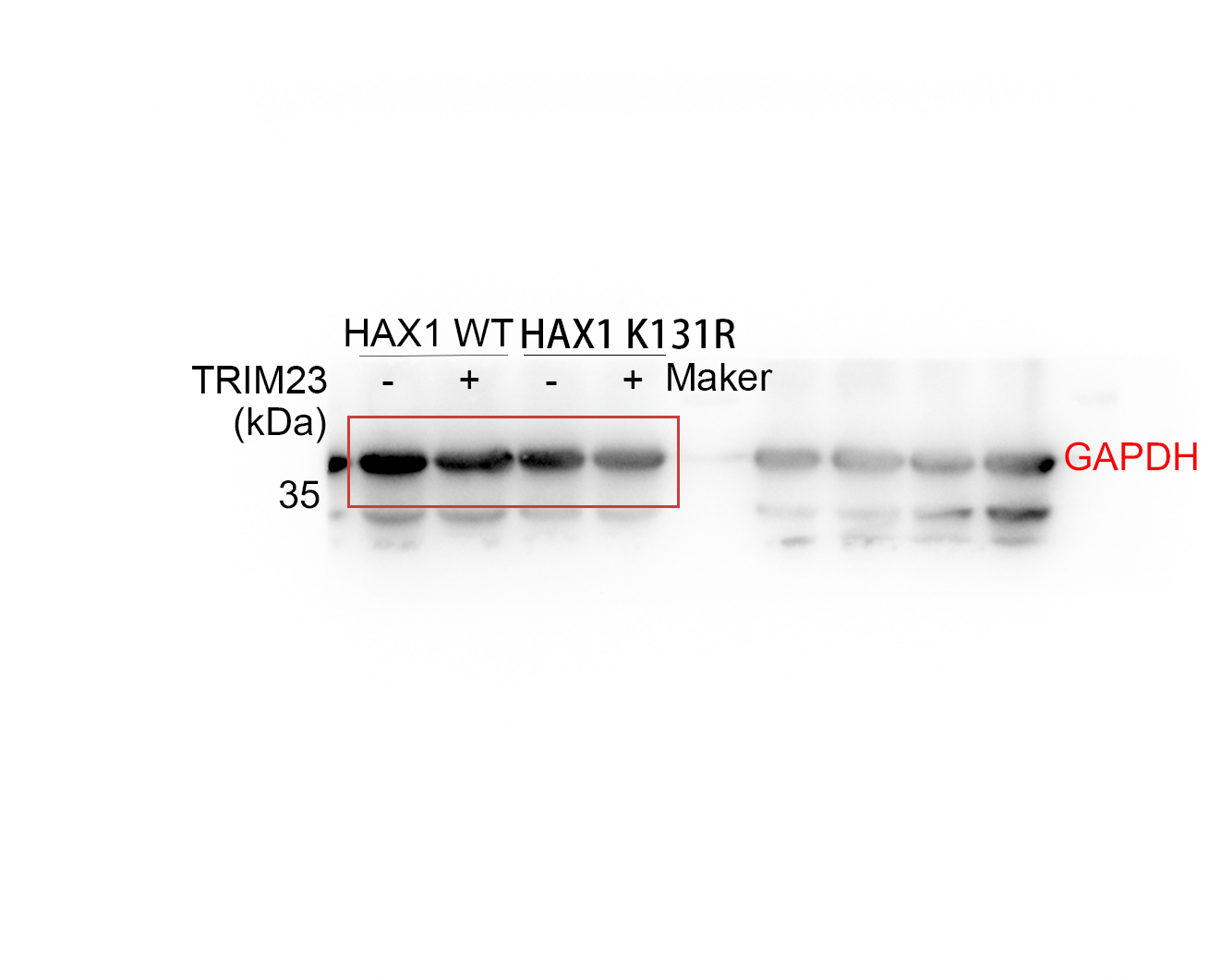

Supplement: Supplementary file 8 — Source data Fig. 5 [file 44318_2024_120_MOESM8_ESM.zip › Figure 5/5I/WCL/western-GAPDH.Tif]

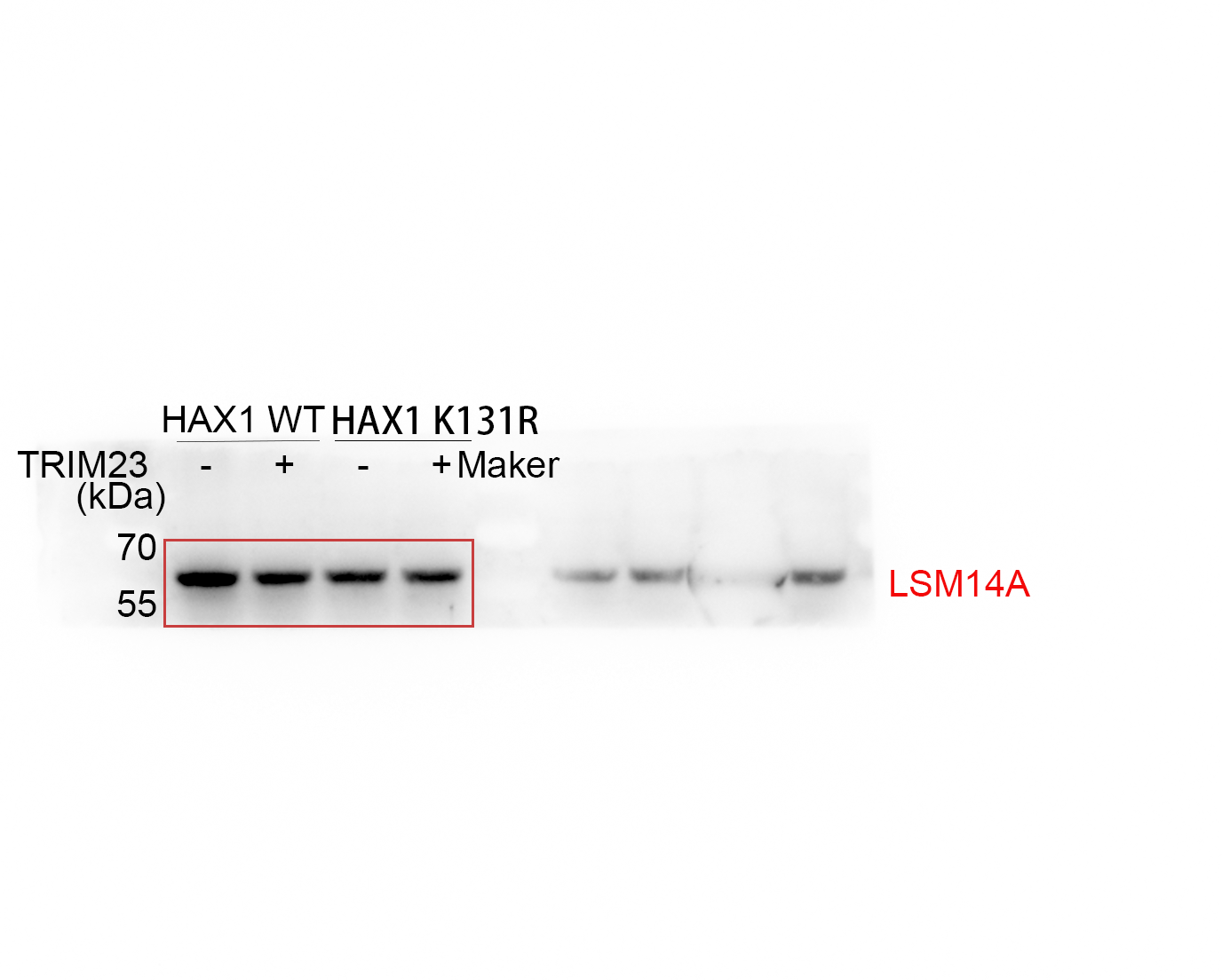

Supplement: Supplementary file 8 — Source data Fig. 5 [file 44318_2024_120_MOESM8_ESM.zip › Figure 5/5I/WCL/western-LSM14A.Tif]

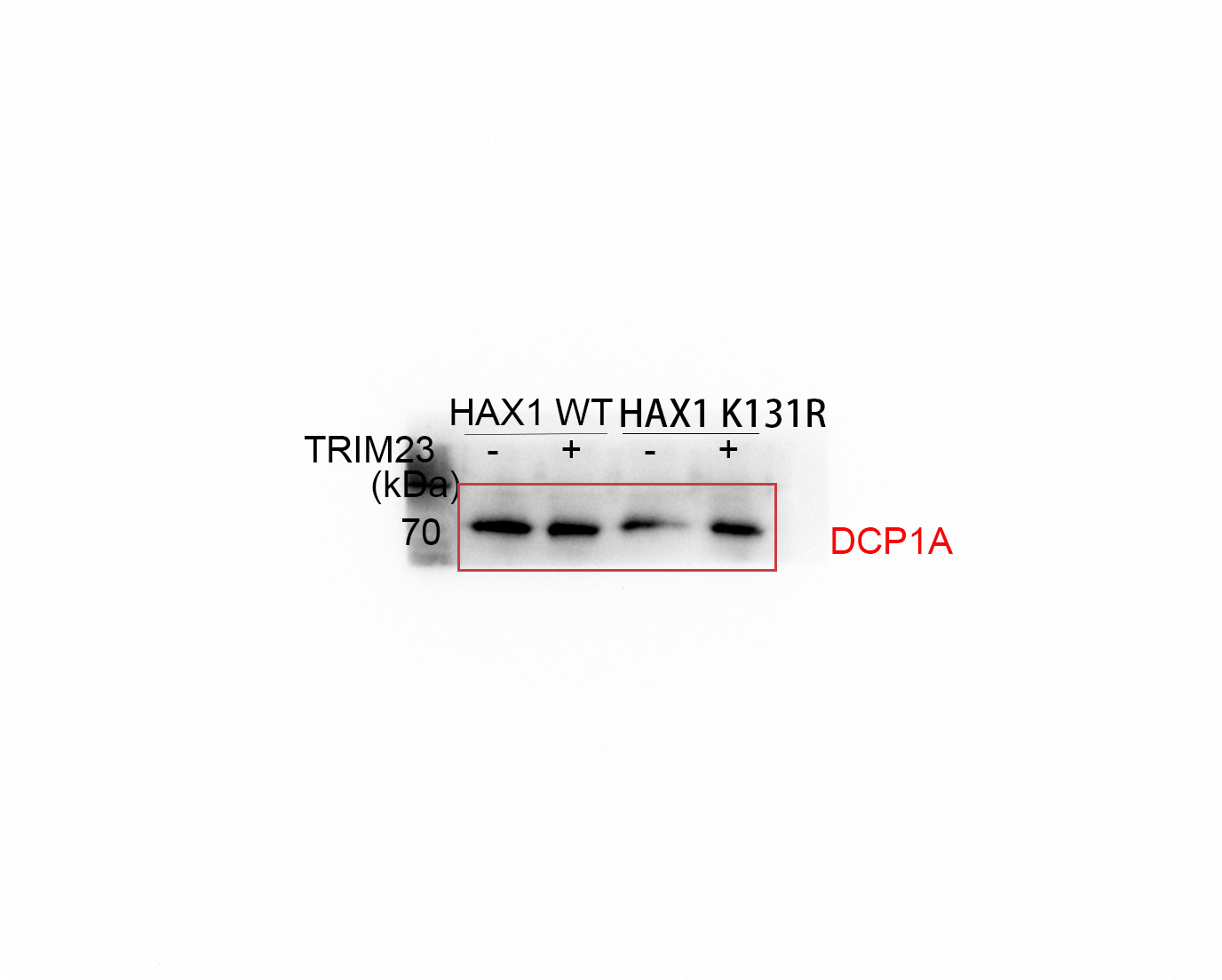

Supplement: Supplementary file 8 — Source data Fig. 5 [file 44318_2024_120_MOESM8_ESM.zip › Figure 5/5I/WCL/western-DCP1A.Tif]

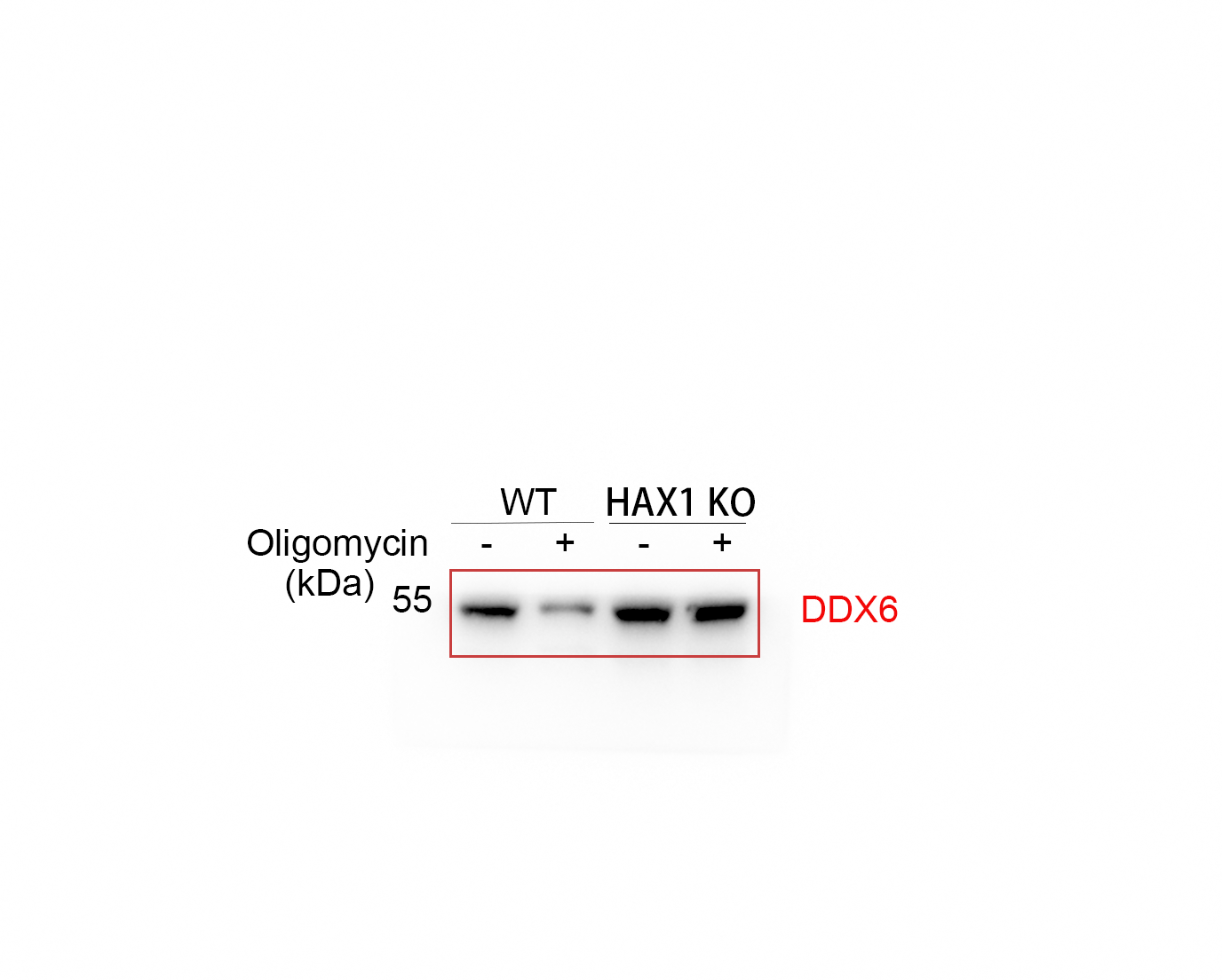

Supplement: Supplementary file 8 — Source data Fig. 5 [file 44318_2024_120_MOESM8_ESM.zip › Figure 5/5G/S2/western-DDX6.Tif]

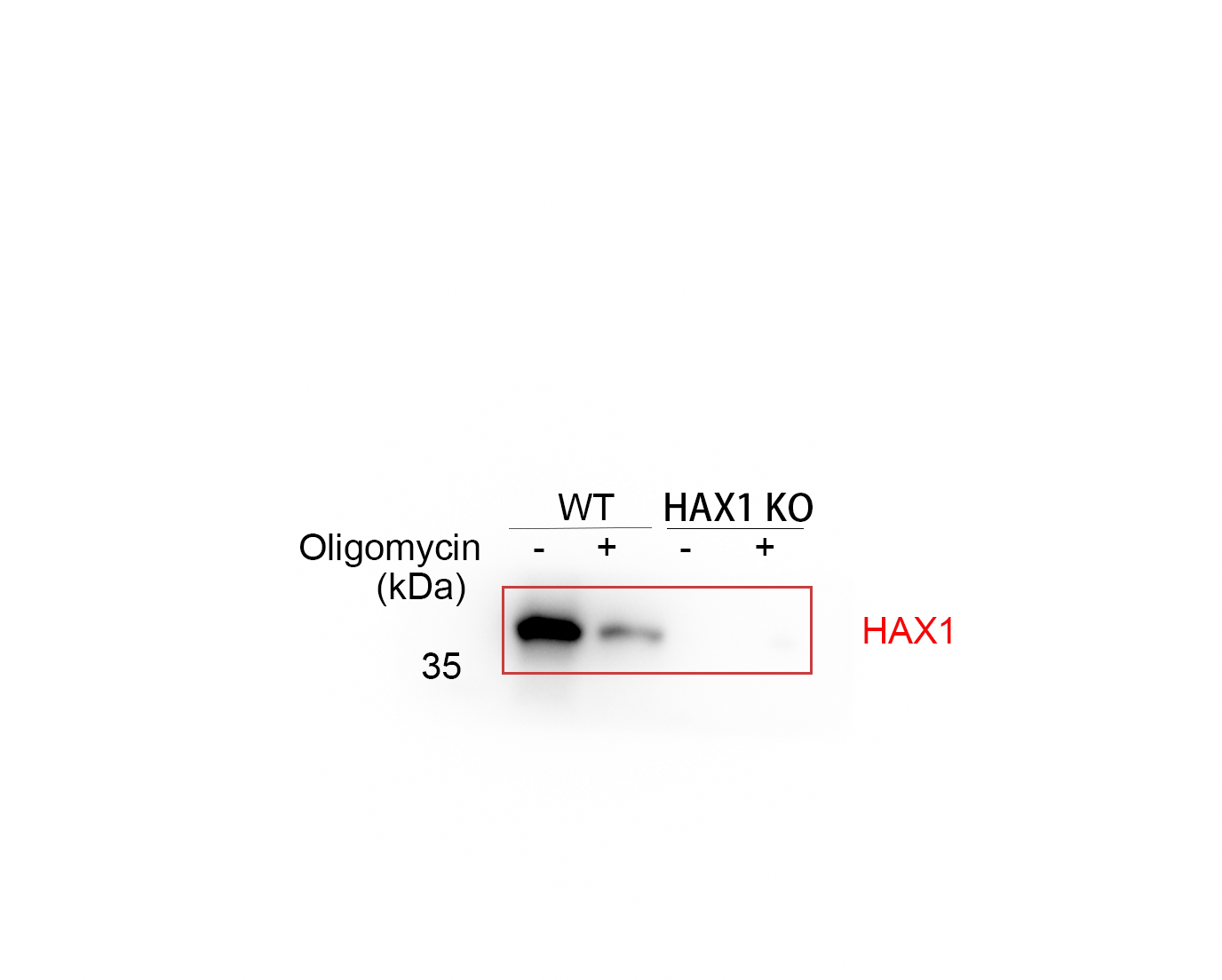

Supplement: Supplementary file 8 — Source data Fig. 5 [file 44318_2024_120_MOESM8_ESM.zip › Figure 5/5G/S2/western-HAX1.Tif]

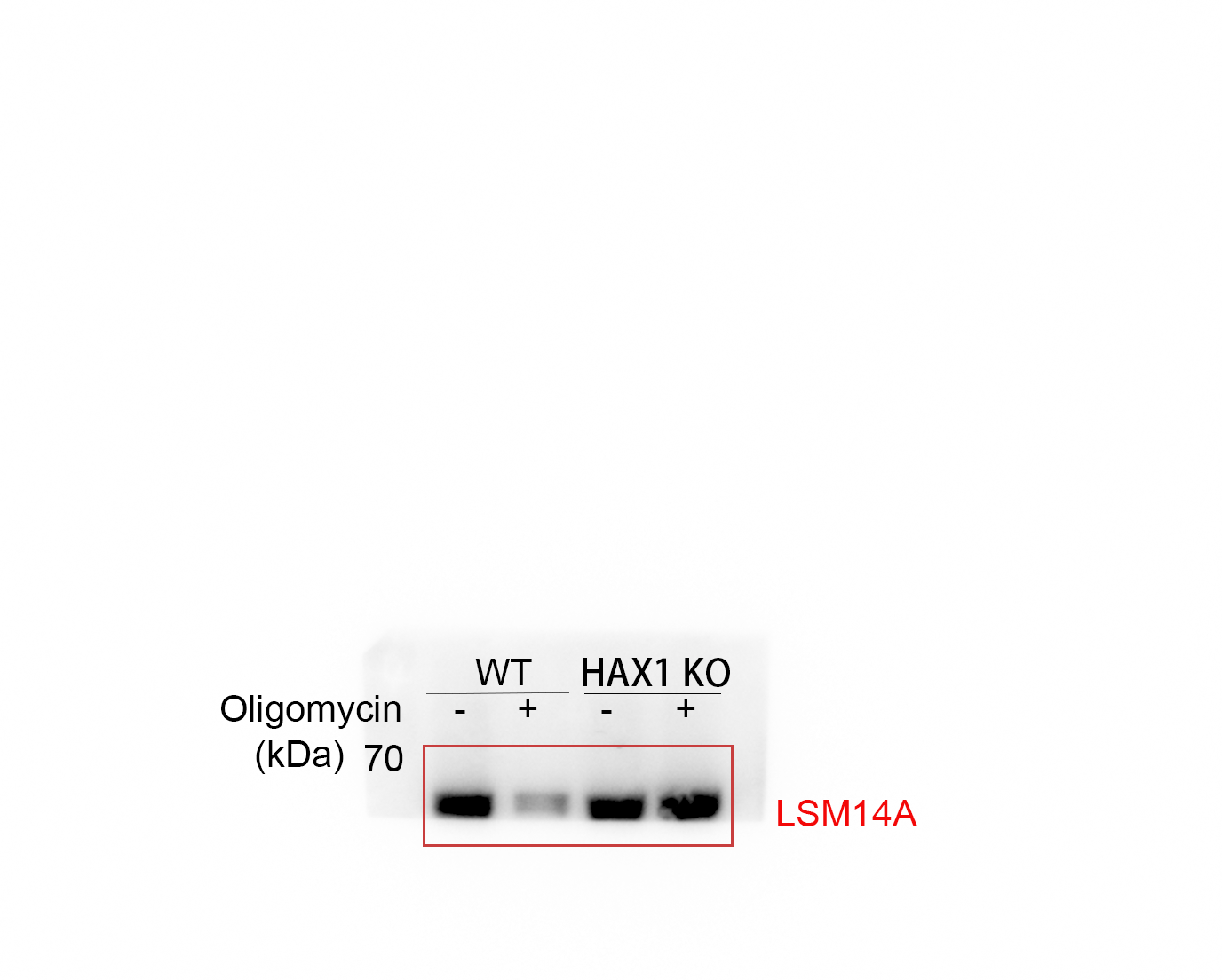

Supplement: Supplementary file 8 — Source data Fig. 5 [file 44318_2024_120_MOESM8_ESM.zip › Figure 5/5G/S2/western-LSM14A.Tif]

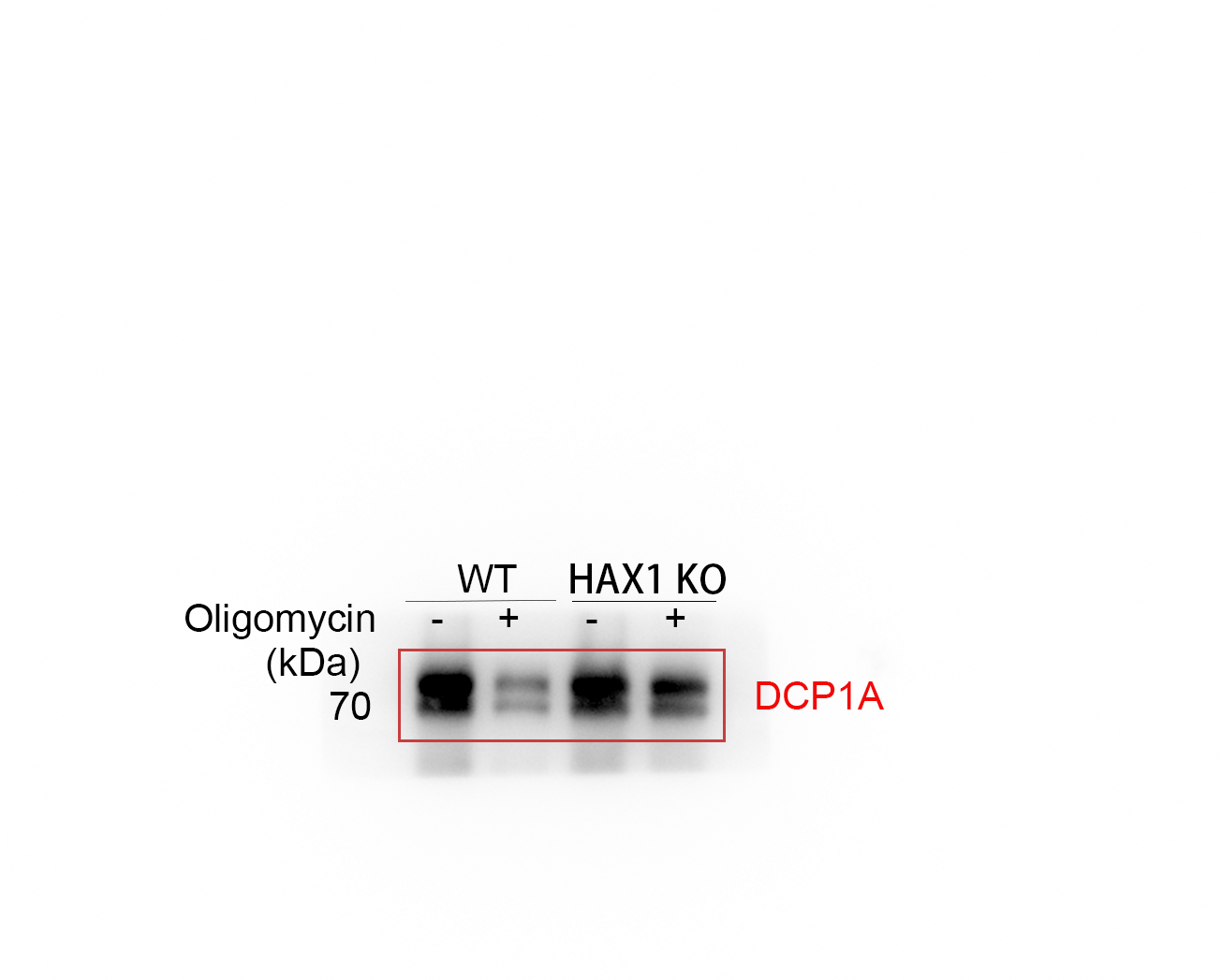

Supplement: Supplementary file 8 — Source data Fig. 5 [file 44318_2024_120_MOESM8_ESM.zip › Figure 5/5G/S2/western-DCP1A.Tif]

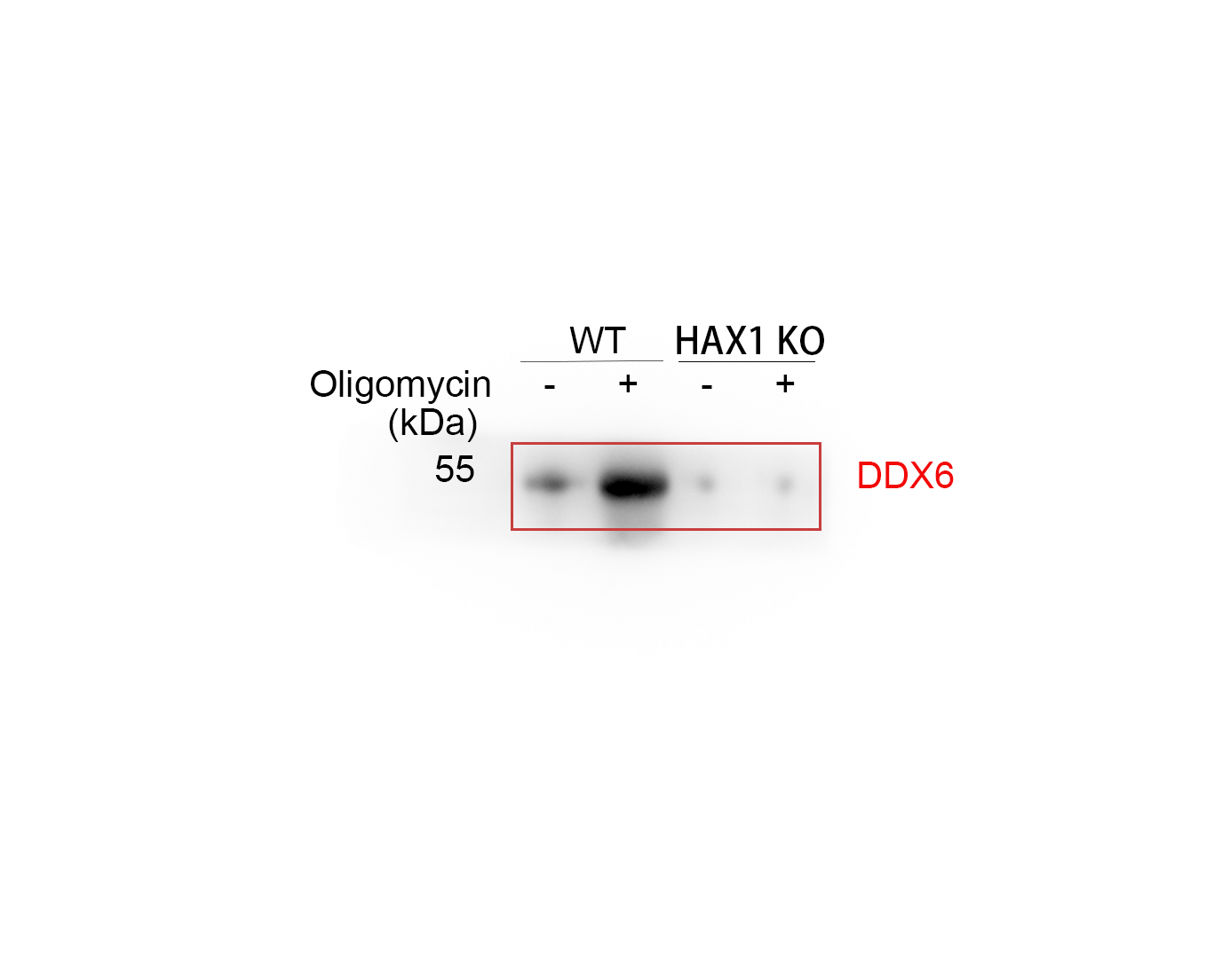

Supplement: Supplementary file 8 — Source data Fig. 5 [file 44318_2024_120_MOESM8_ESM.zip › Figure 5/5G/P2/western-DDX6.Tif]

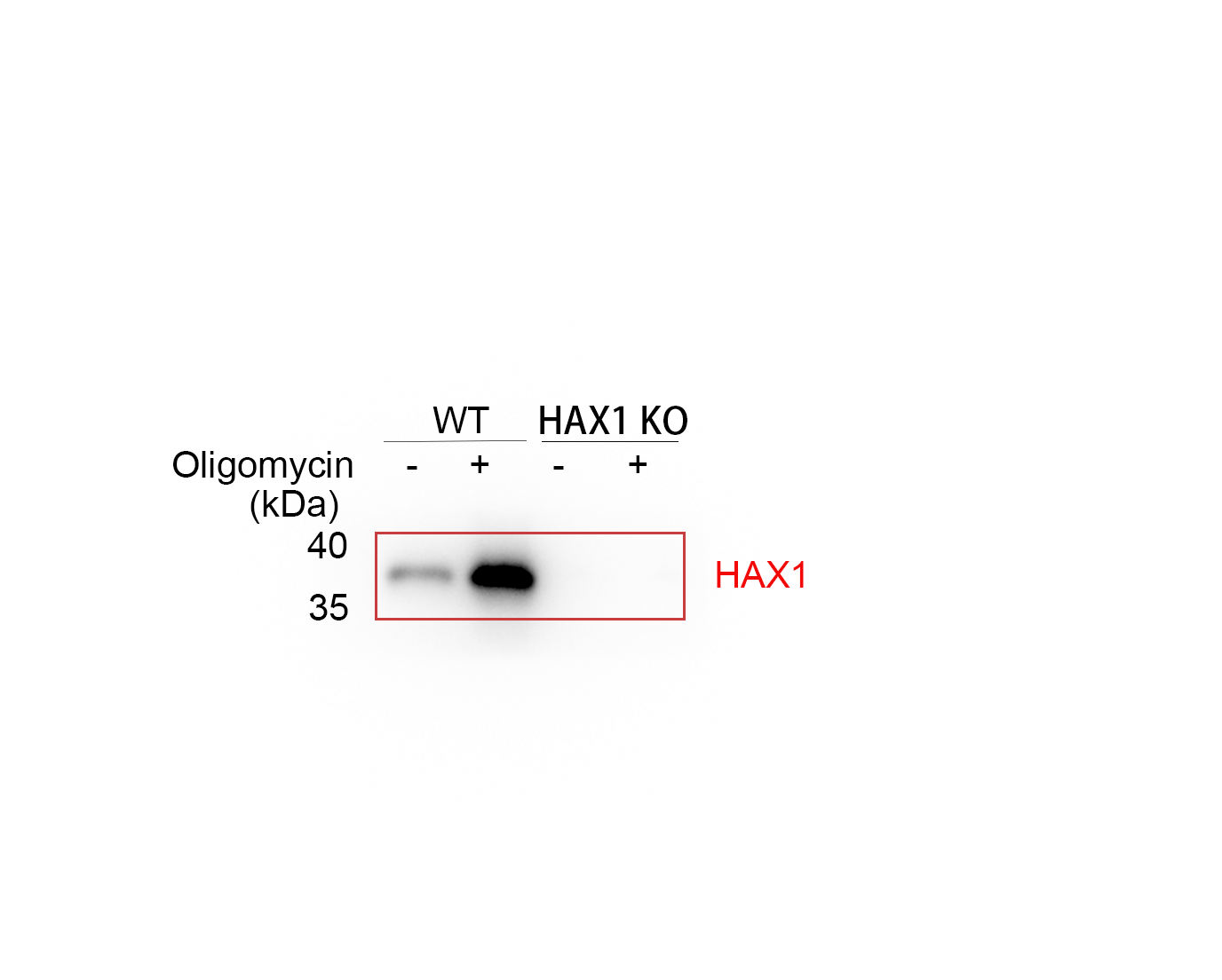

Supplement: Supplementary file 8 — Source data Fig. 5 [file 44318_2024_120_MOESM8_ESM.zip › Figure 5/5G/P2/western-HAX1.Tif]

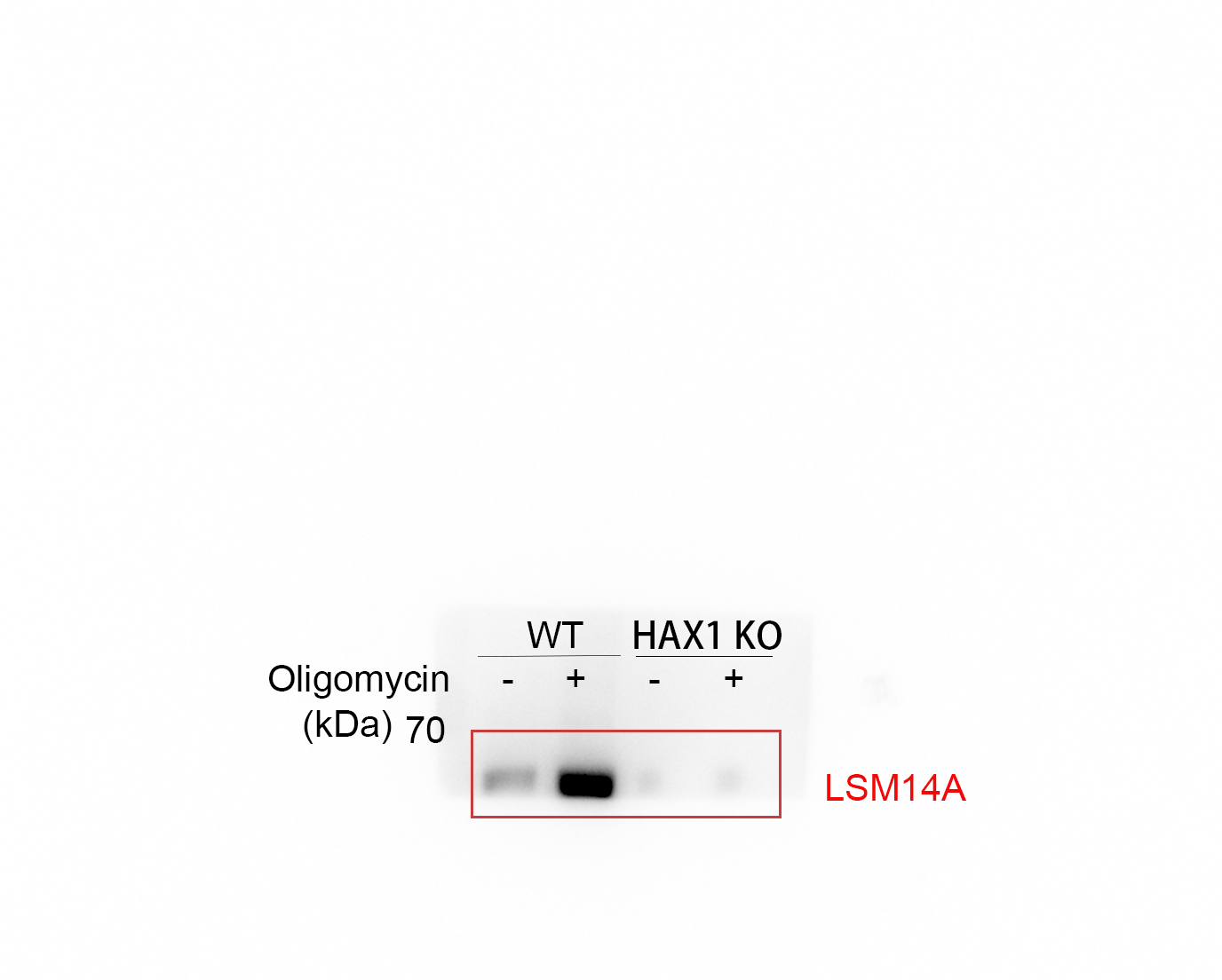

Supplement: Supplementary file 8 — Source data Fig. 5 [file 44318_2024_120_MOESM8_ESM.zip › Figure 5/5G/P2/western-LSM14A.Tif]

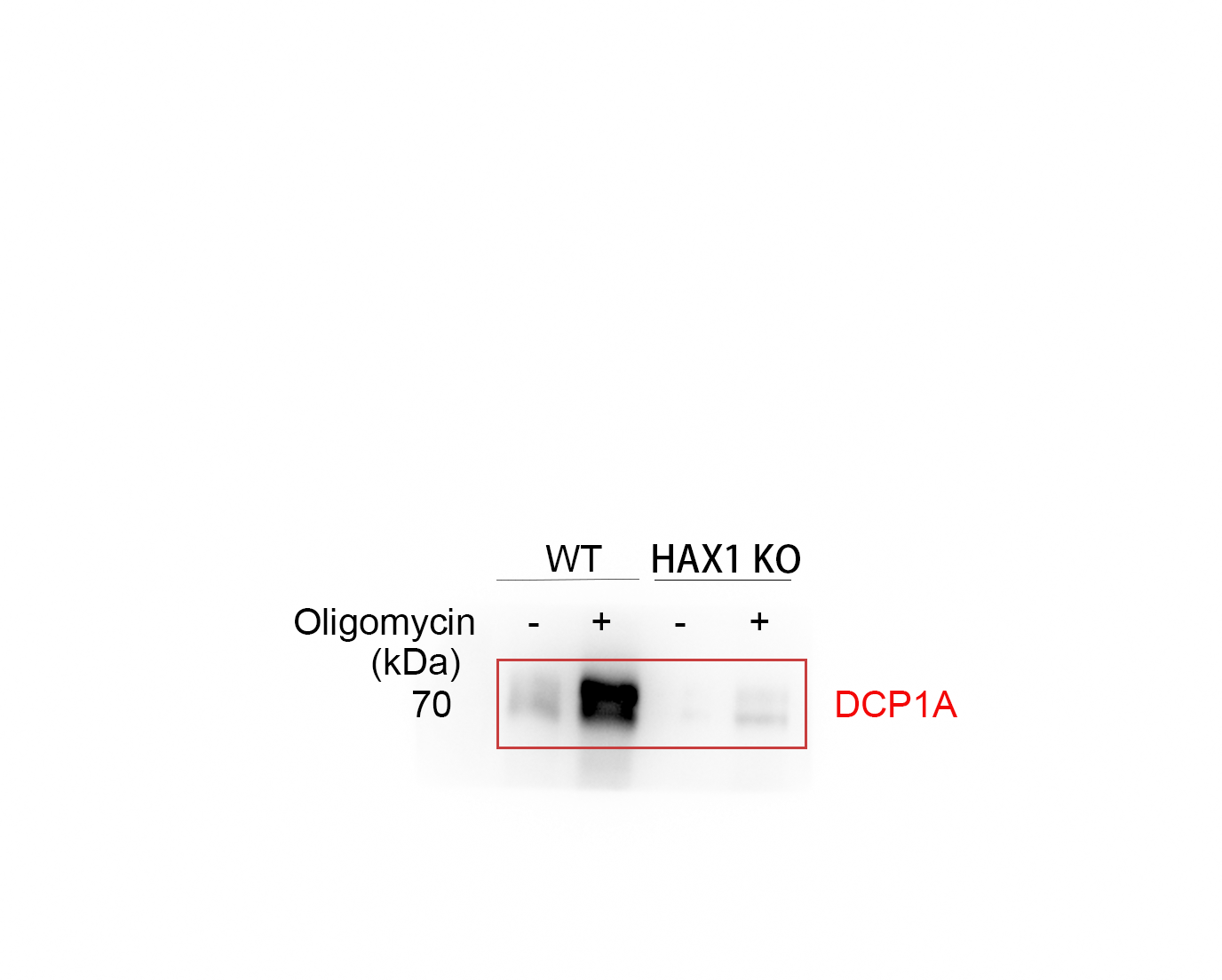

Supplement: Supplementary file 8 — Source data Fig. 5 [file 44318_2024_120_MOESM8_ESM.zip › Figure 5/5G/P2/western-DCP1A.Tif]

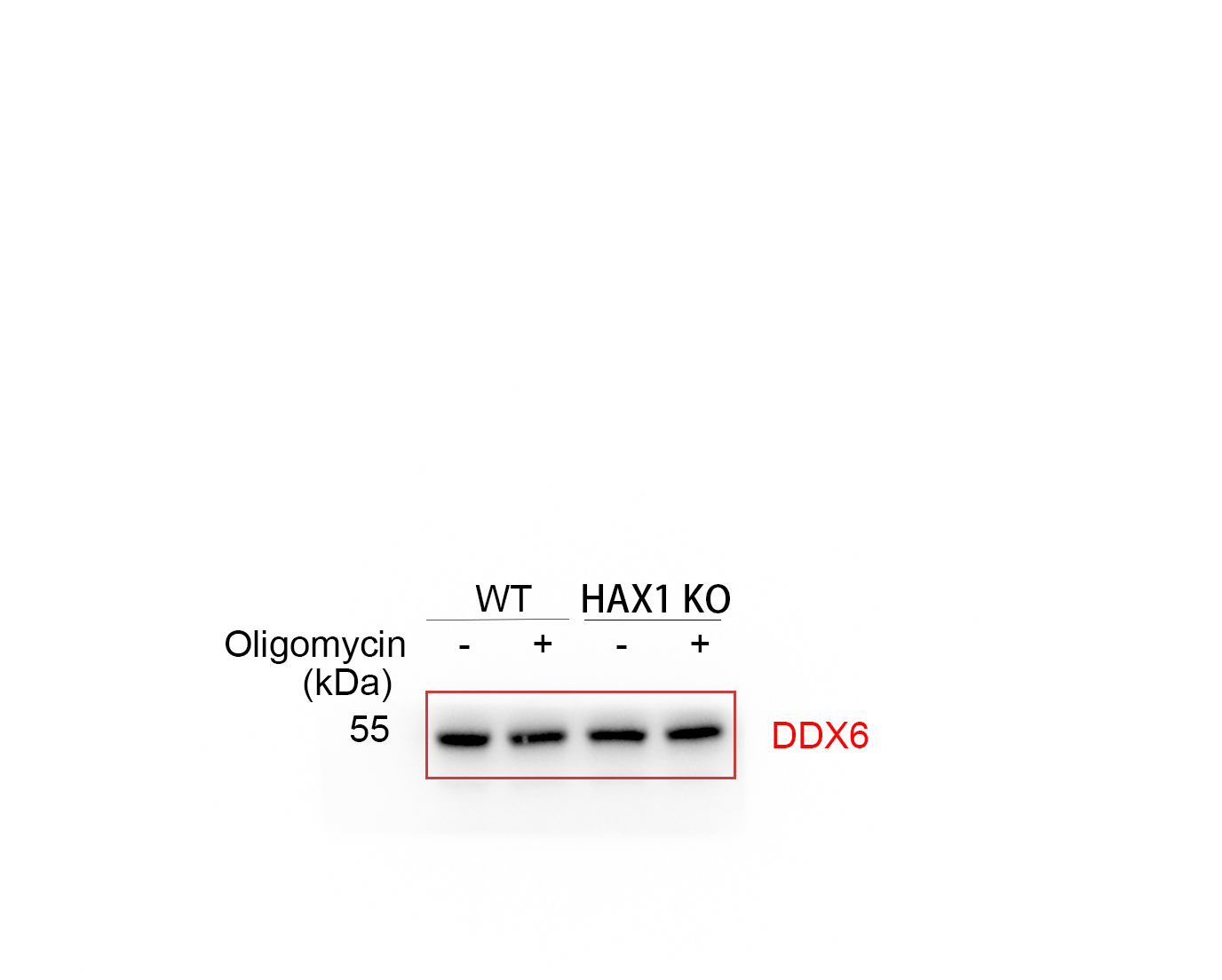

Supplement: Supplementary file 8 — Source data Fig. 5 [file 44318_2024_120_MOESM8_ESM.zip › Figure 5/5G/WCL/western-DDX6.Tif]

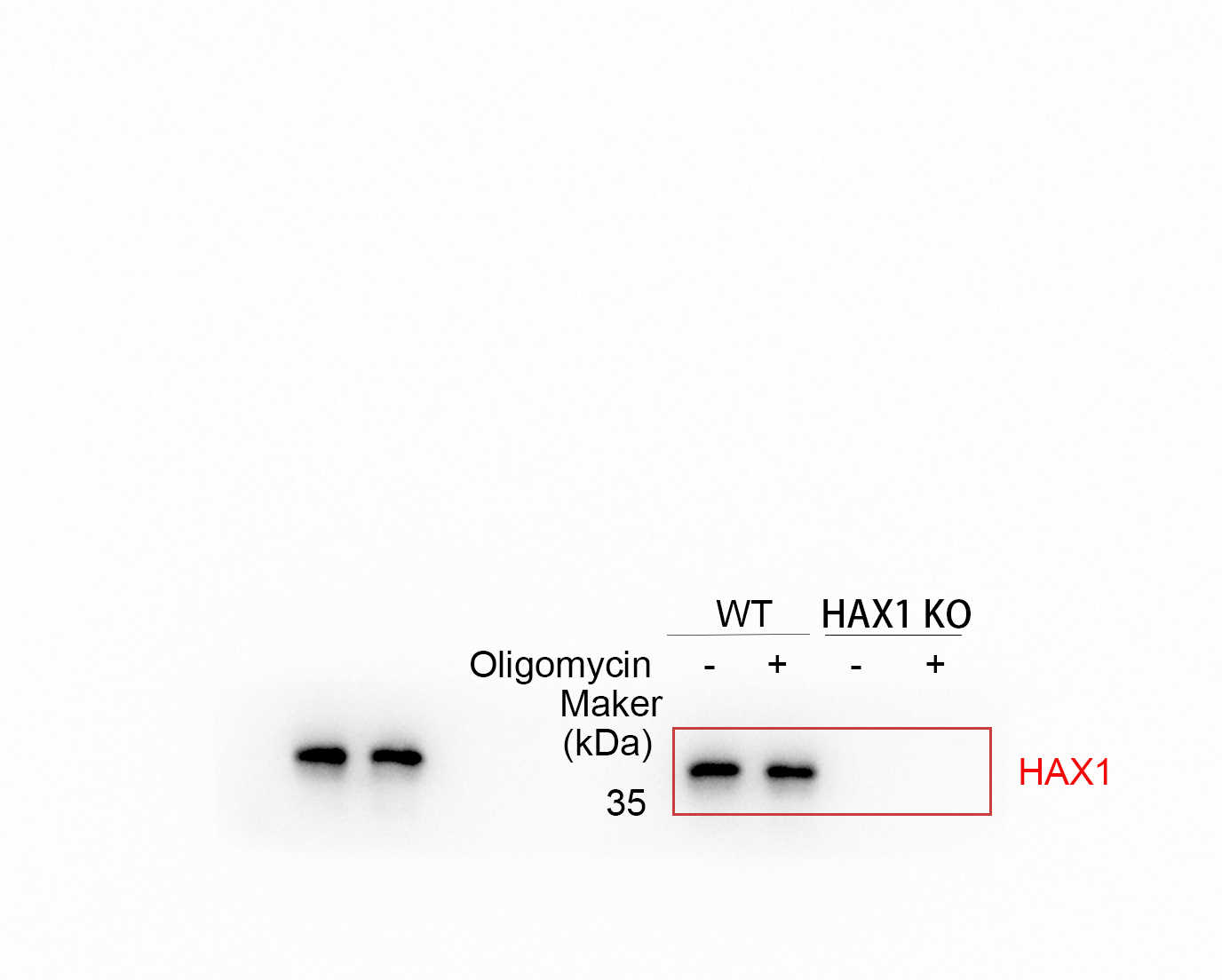

Supplement: Supplementary file 8 — Source data Fig. 5 [file 44318_2024_120_MOESM8_ESM.zip › Figure 5/5G/WCL/western-HAX1.Tif]

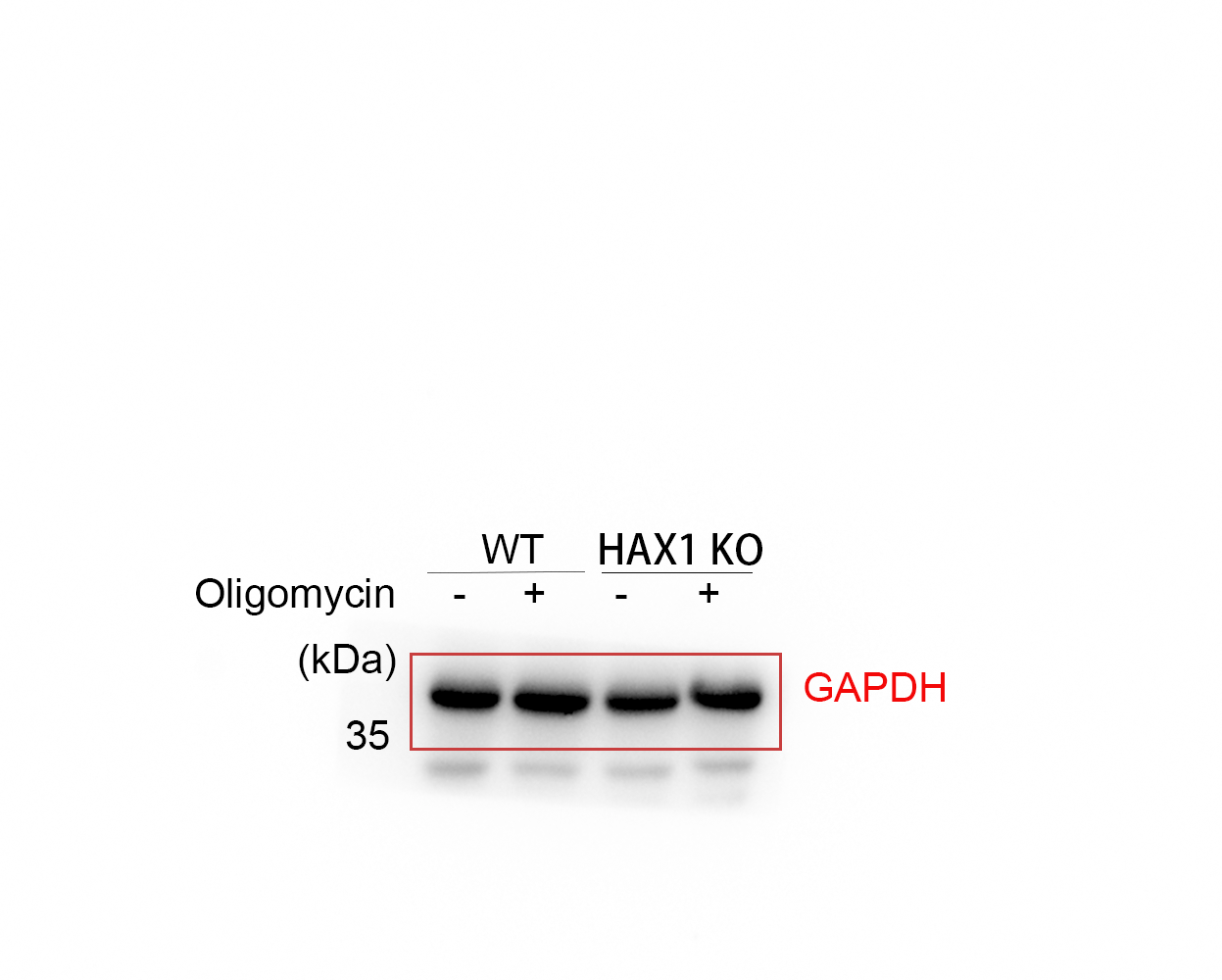

Supplement: Supplementary file 8 — Source data Fig. 5 [file 44318_2024_120_MOESM8_ESM.zip › Figure 5/5G/WCL/western-GAPDH.Tif]

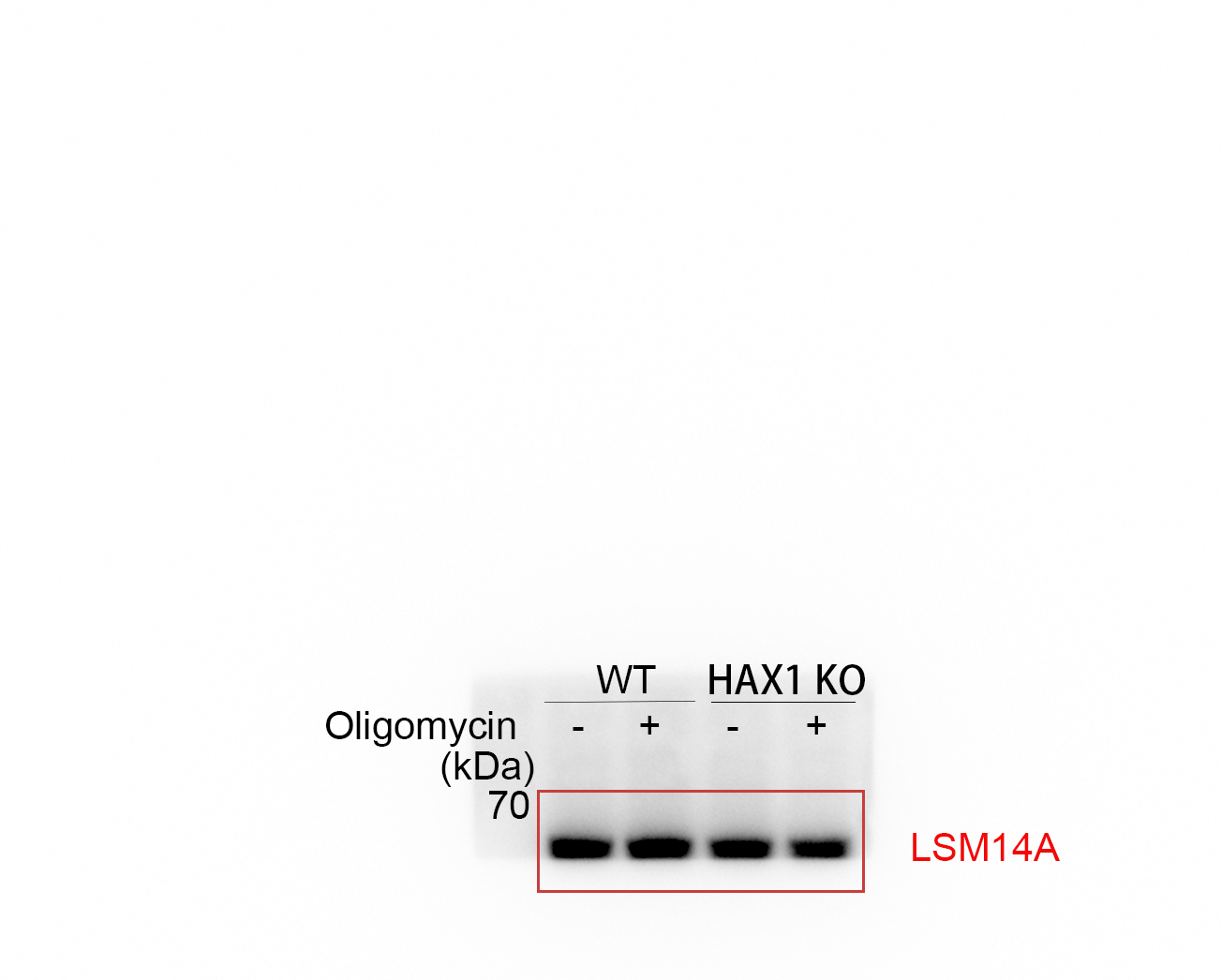

Supplement: Supplementary file 8 — Source data Fig. 5 [file 44318_2024_120_MOESM8_ESM.zip › Figure 5/5G/WCL/western-LSM14A.Tif]

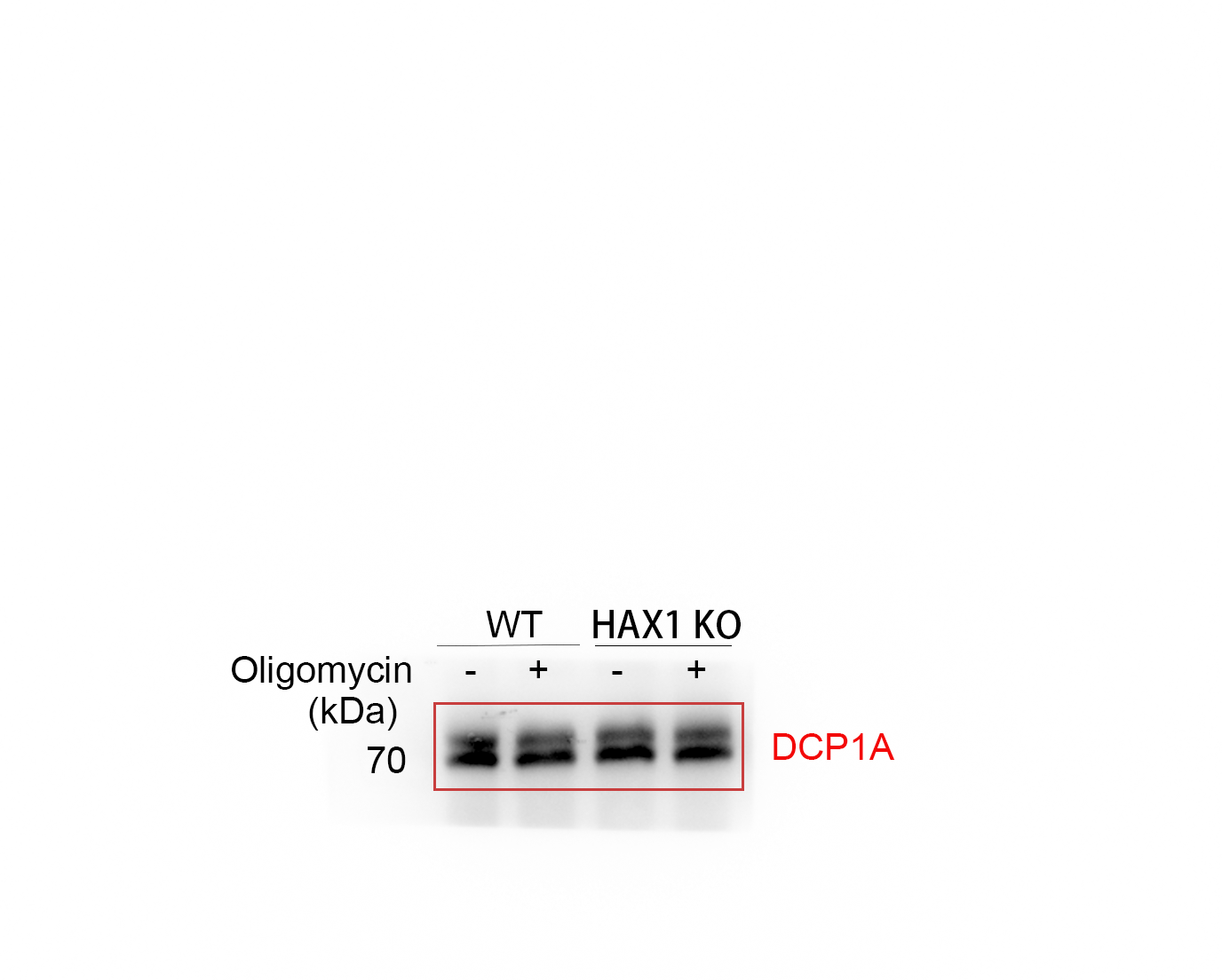

Supplement: Supplementary file 8 — Source data Fig. 5 [file 44318_2024_120_MOESM8_ESM.zip › Figure 5/5G/WCL/western-DCP1A.Tif]

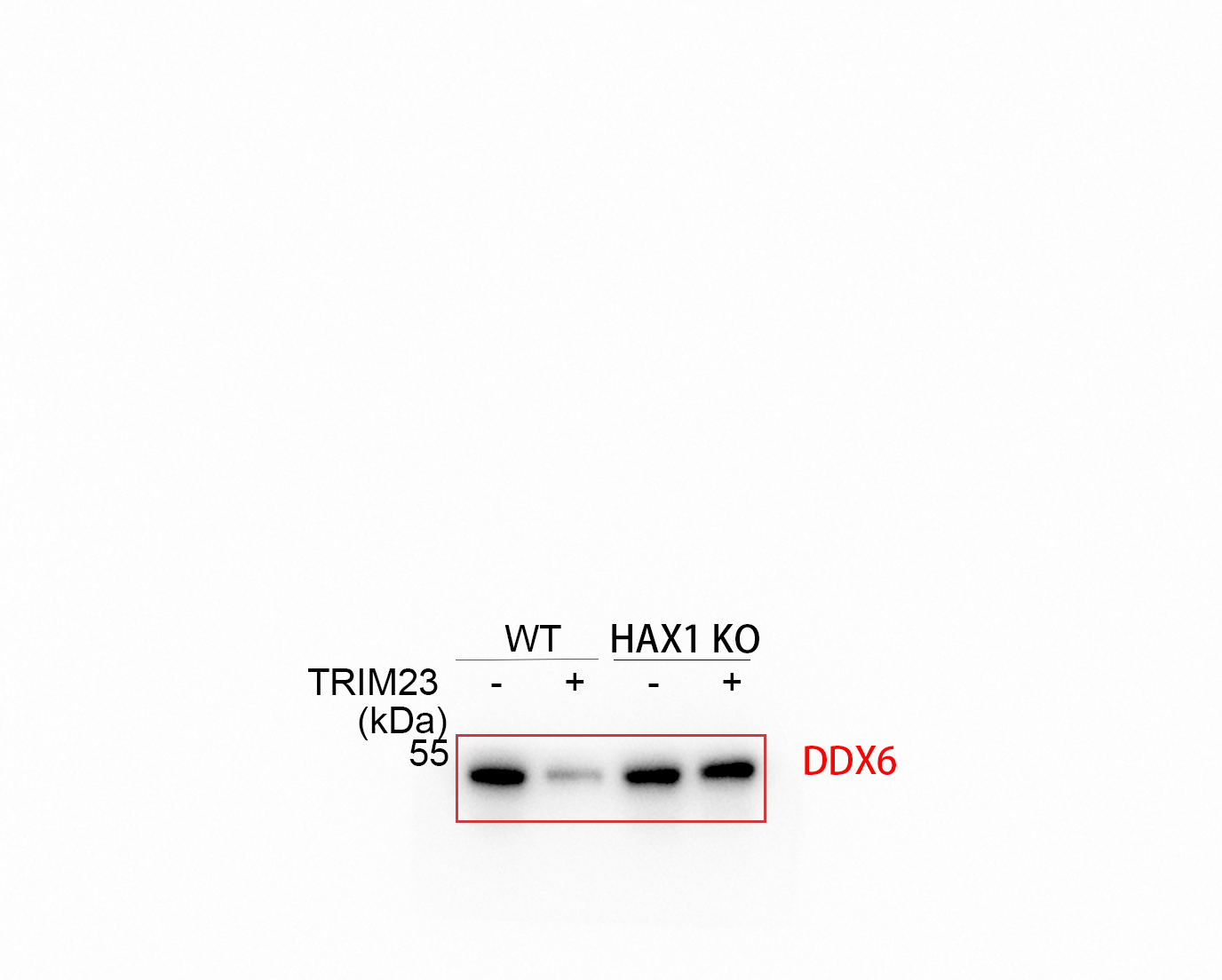

Supplement: Supplementary file 8 — Source data Fig. 5 [file 44318_2024_120_MOESM8_ESM.zip › Figure 5/5F/S2/western-DDX6.Tif]

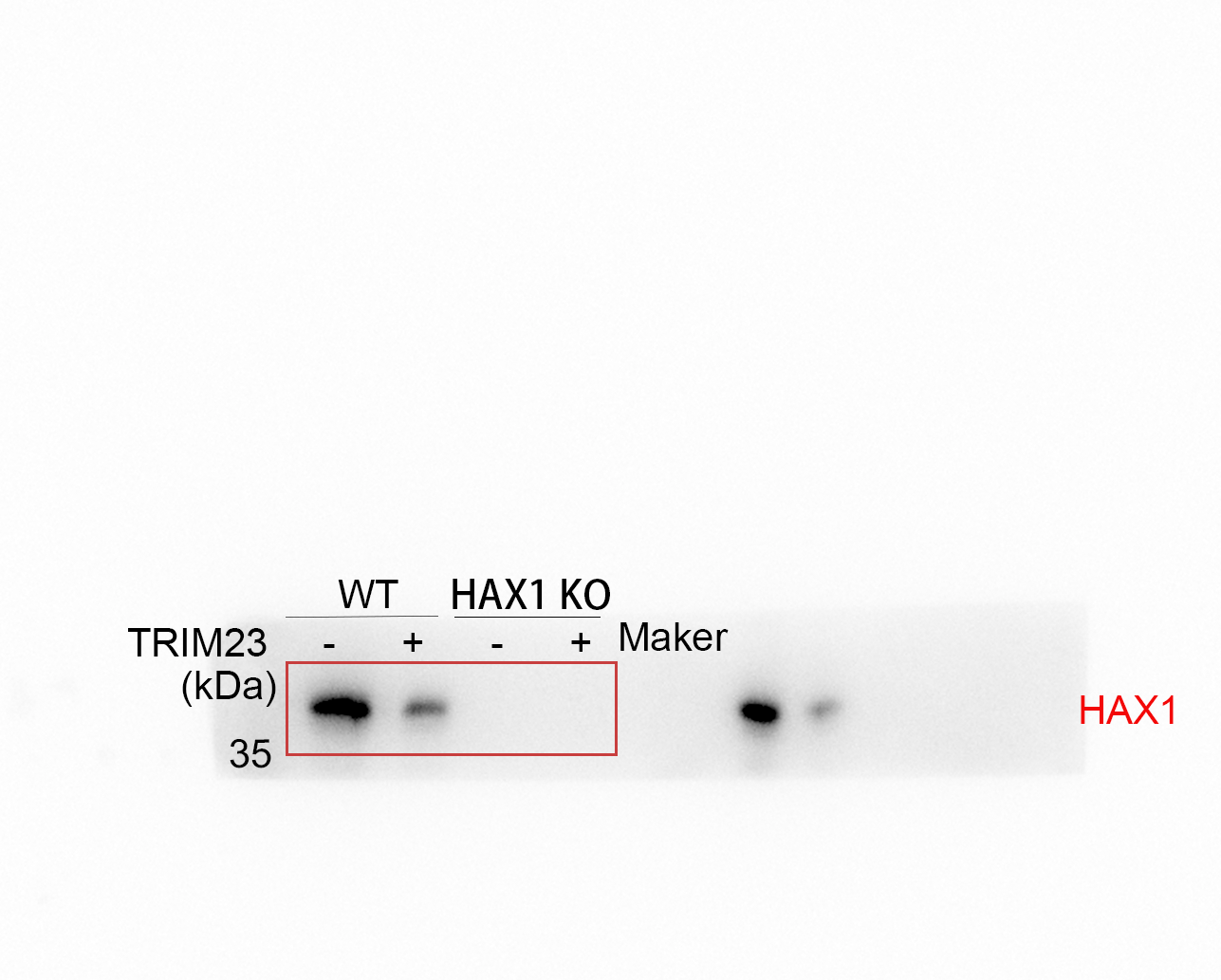

Supplement: Supplementary file 8 — Source data Fig. 5 [file 44318_2024_120_MOESM8_ESM.zip › Figure 5/5F/S2/western-HAX1.Tif]

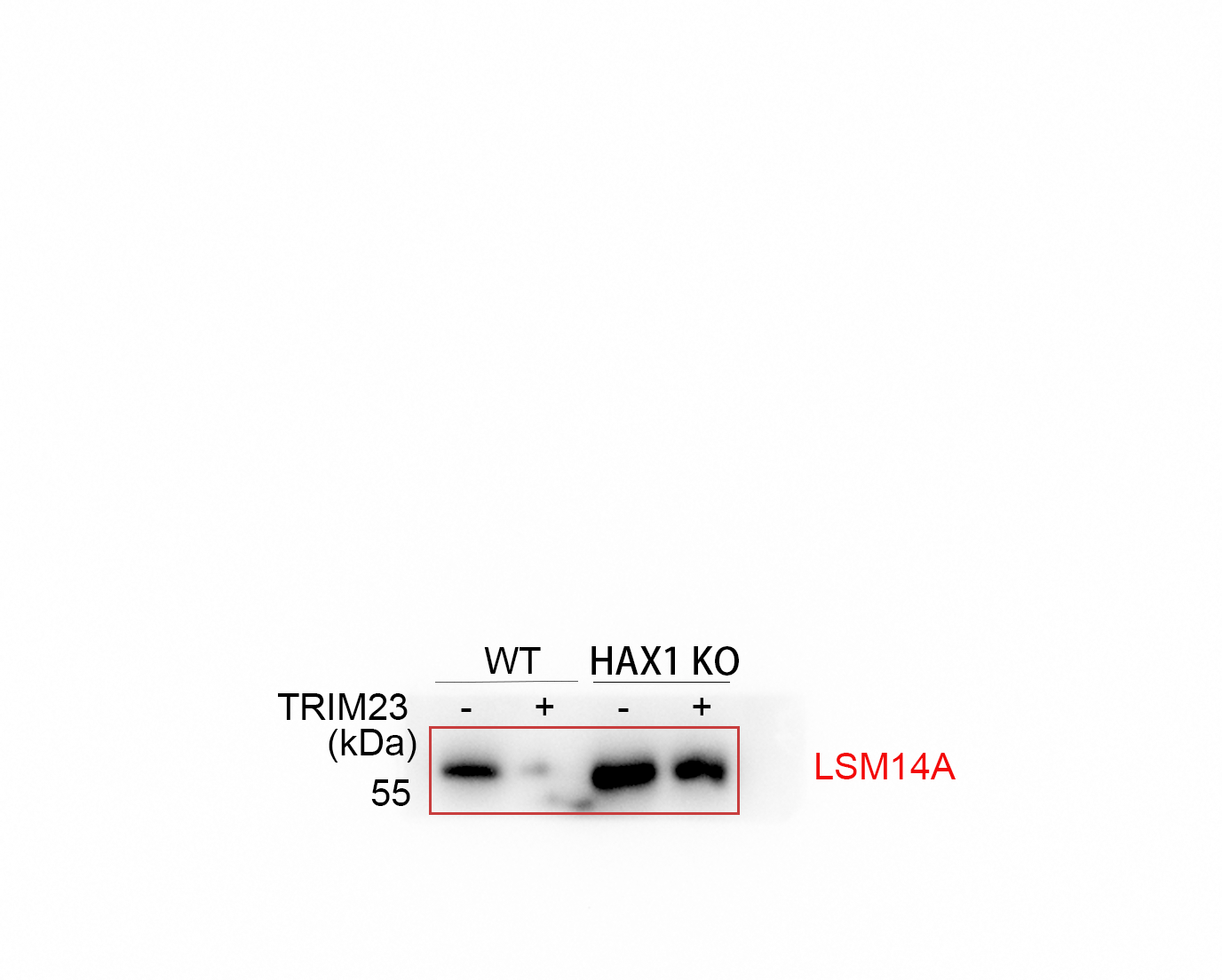

Supplement: Supplementary file 8 — Source data Fig. 5 [file 44318_2024_120_MOESM8_ESM.zip › Figure 5/5F/S2/western-LSM14A.Tif]

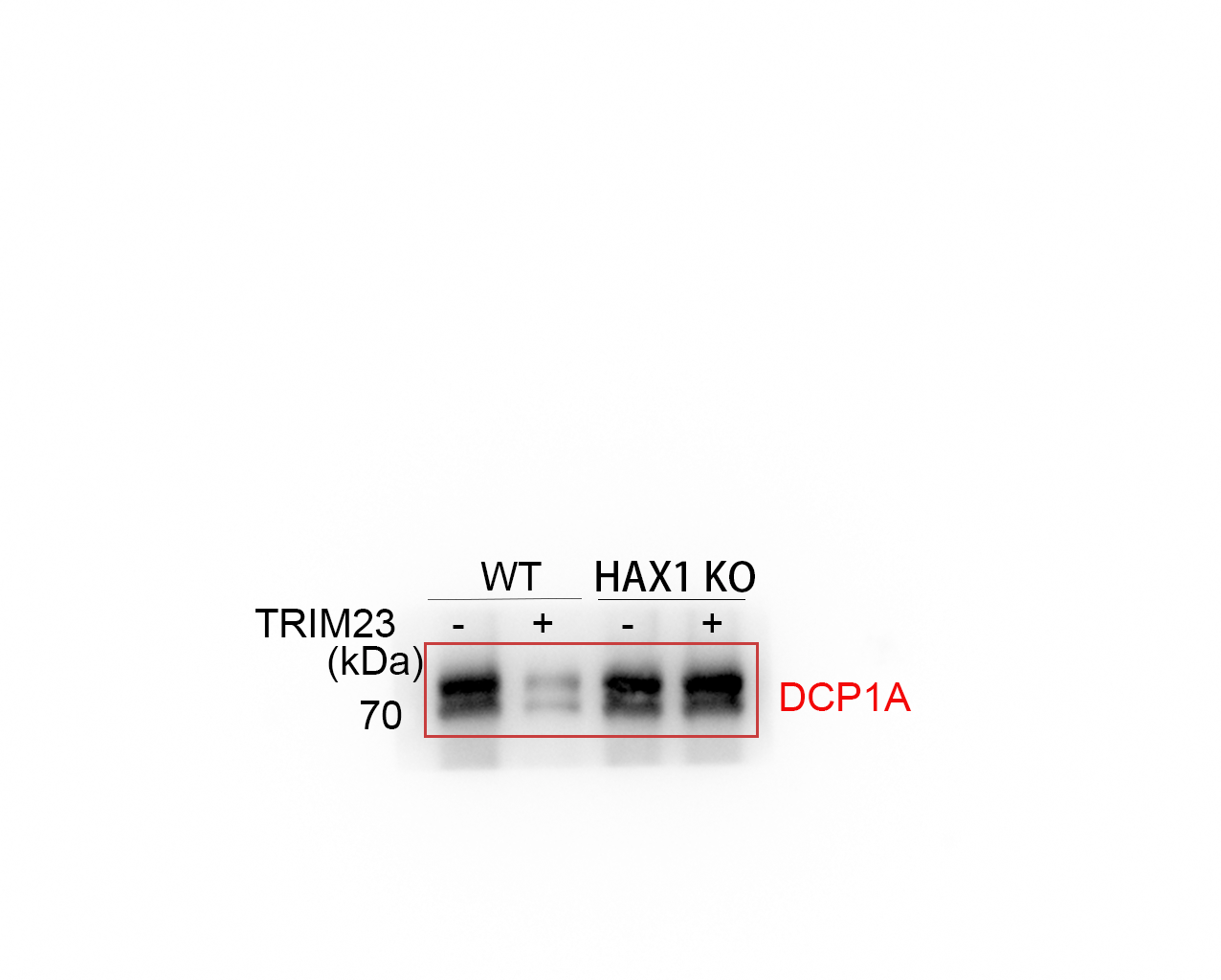

Supplement: Supplementary file 8 — Source data Fig. 5 [file 44318_2024_120_MOESM8_ESM.zip › Figure 5/5F/S2/western-DCP1A.Tif]

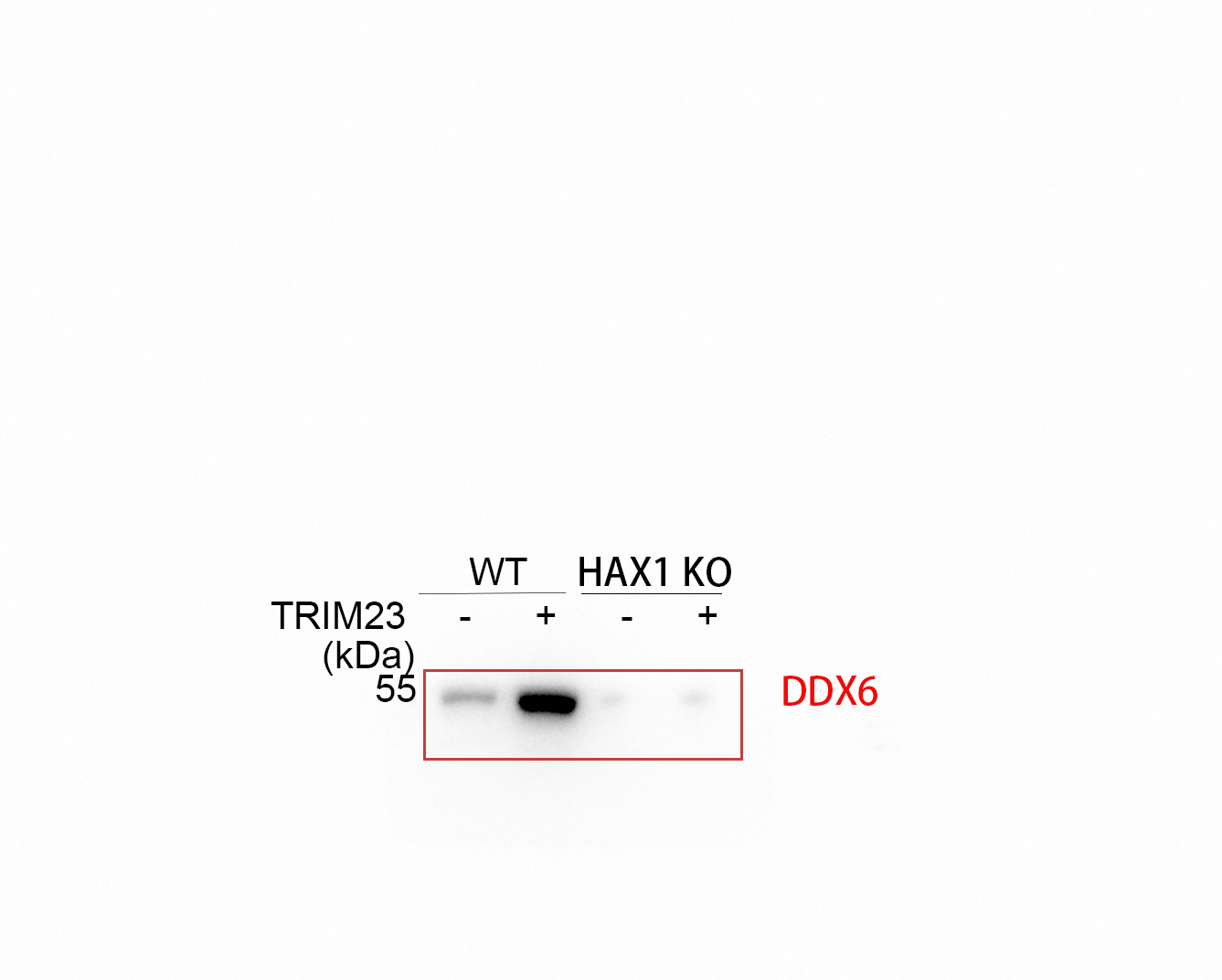

Supplement: Supplementary file 8 — Source data Fig. 5 [file 44318_2024_120_MOESM8_ESM.zip › Figure 5/5F/P2/western-DDX6.Tif]

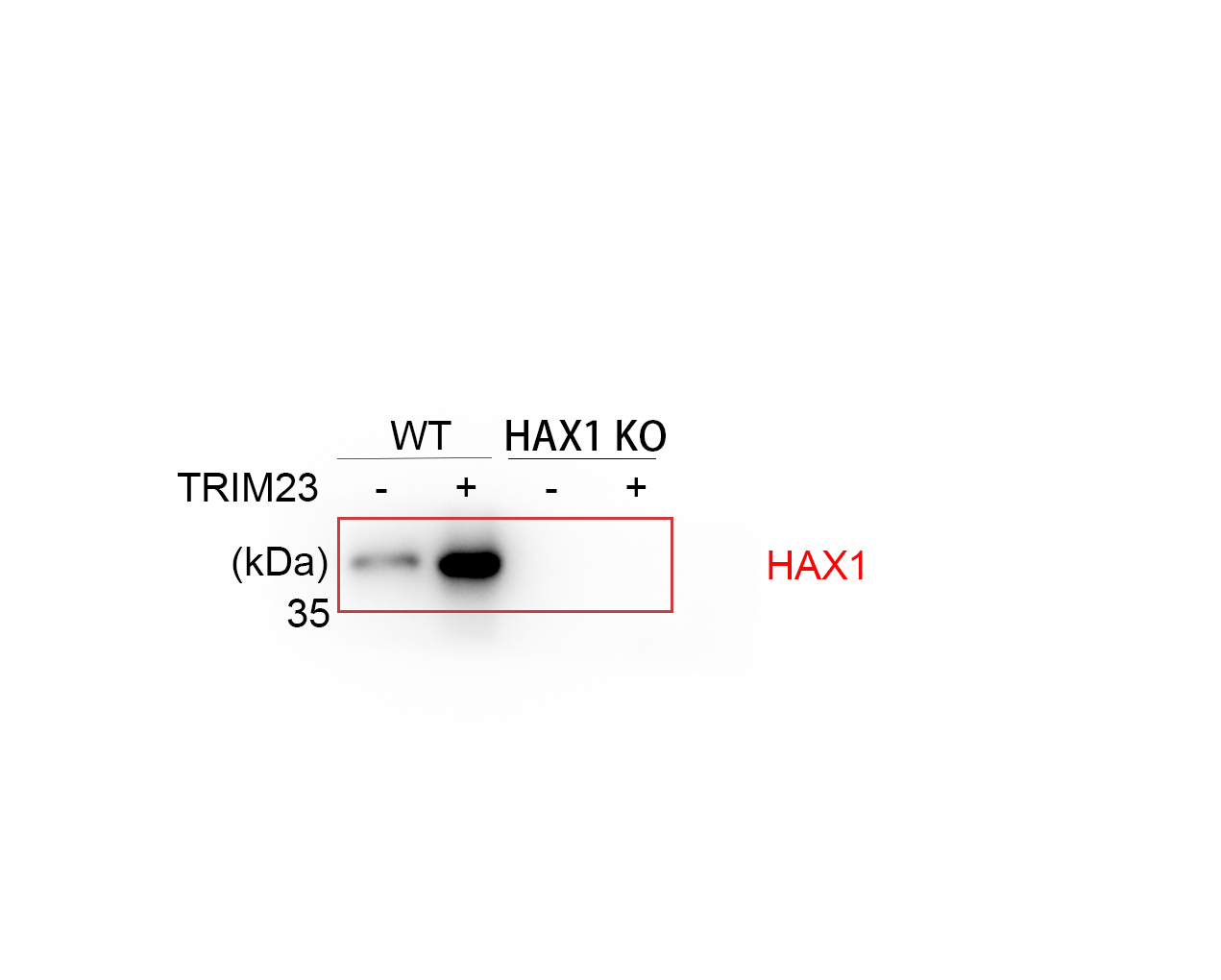

Supplement: Supplementary file 8 — Source data Fig. 5 [file 44318_2024_120_MOESM8_ESM.zip › Figure 5/5F/P2/western-HAX1.Tif]

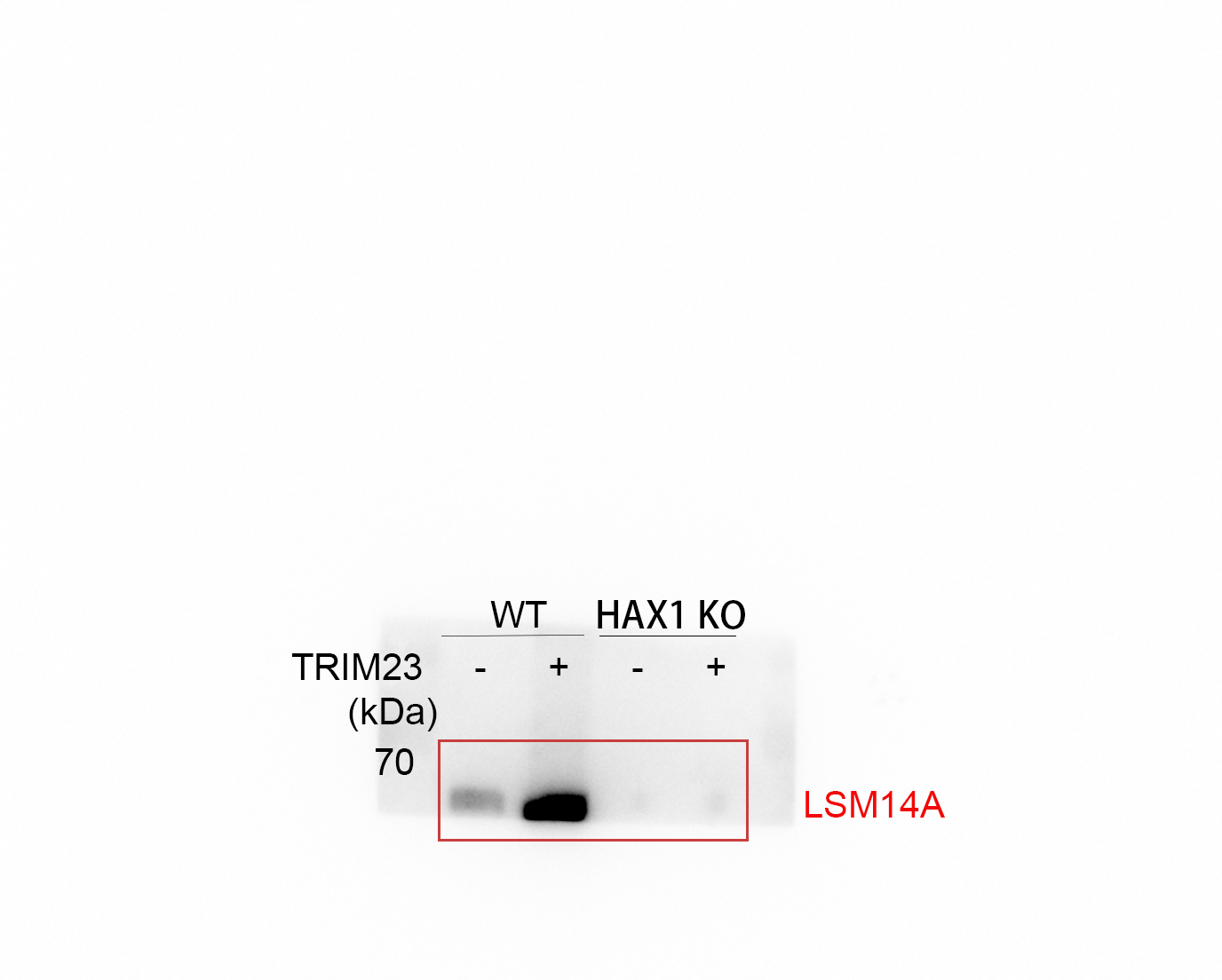

Supplement: Supplementary file 8 — Source data Fig. 5 [file 44318_2024_120_MOESM8_ESM.zip › Figure 5/5F/P2/western-LSM14A.Tif]

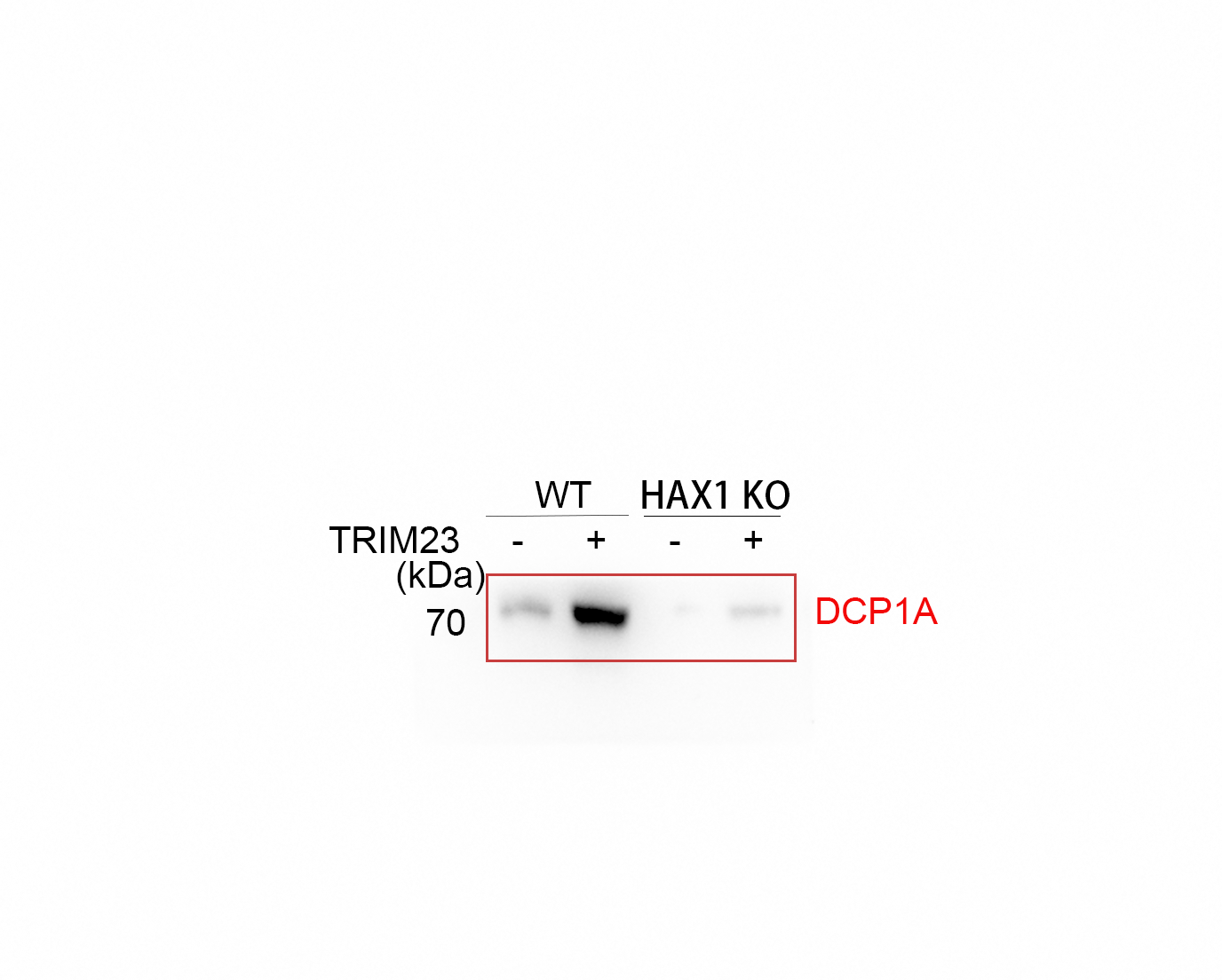

Supplement: Supplementary file 8 — Source data Fig. 5 [file 44318_2024_120_MOESM8_ESM.zip › Figure 5/5F/P2/western-DCP1A.Tif]

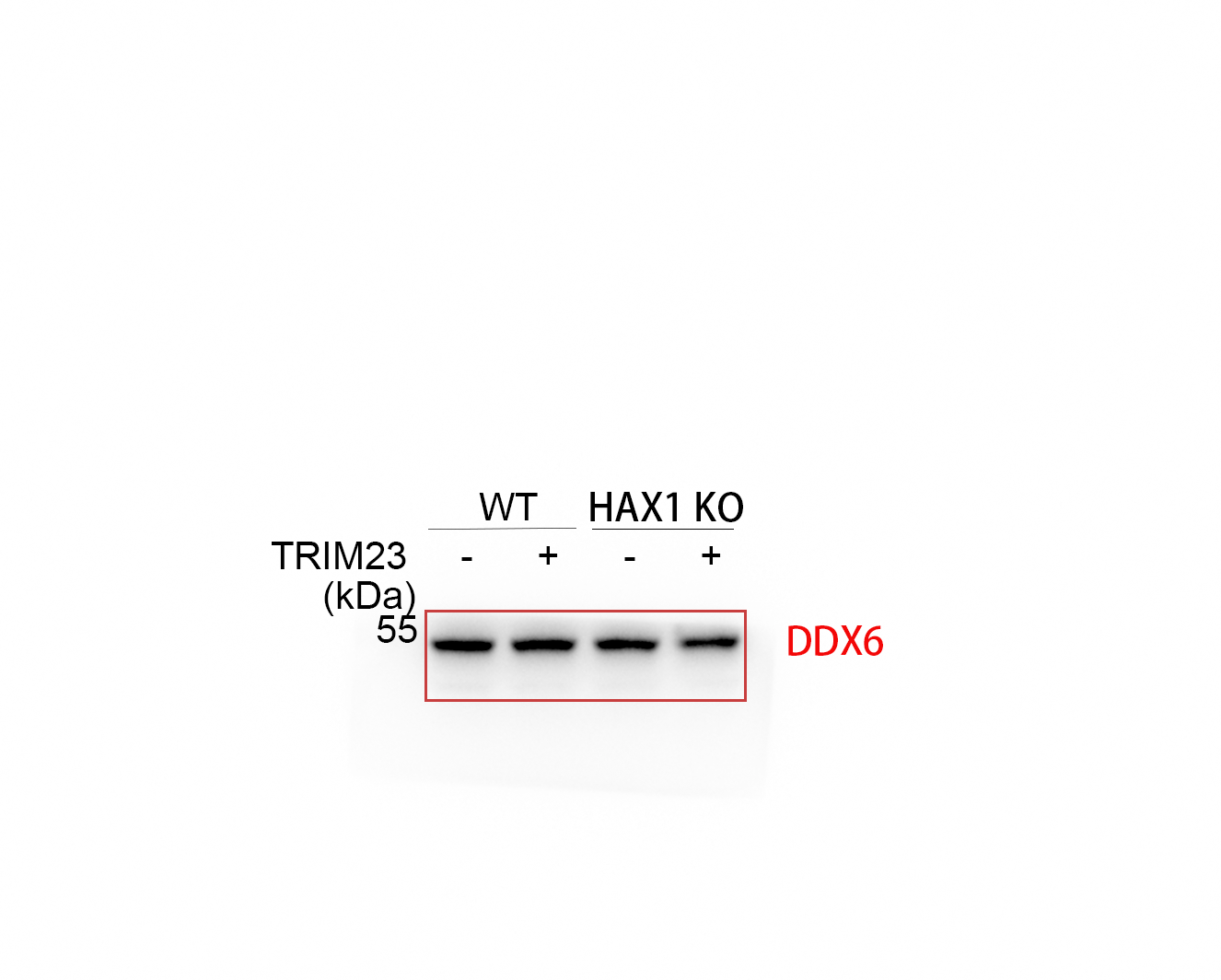

Supplement: Supplementary file 8 — Source data Fig. 5 [file 44318_2024_120_MOESM8_ESM.zip › Figure 5/5F/WCL/western-DDX6.Tif]

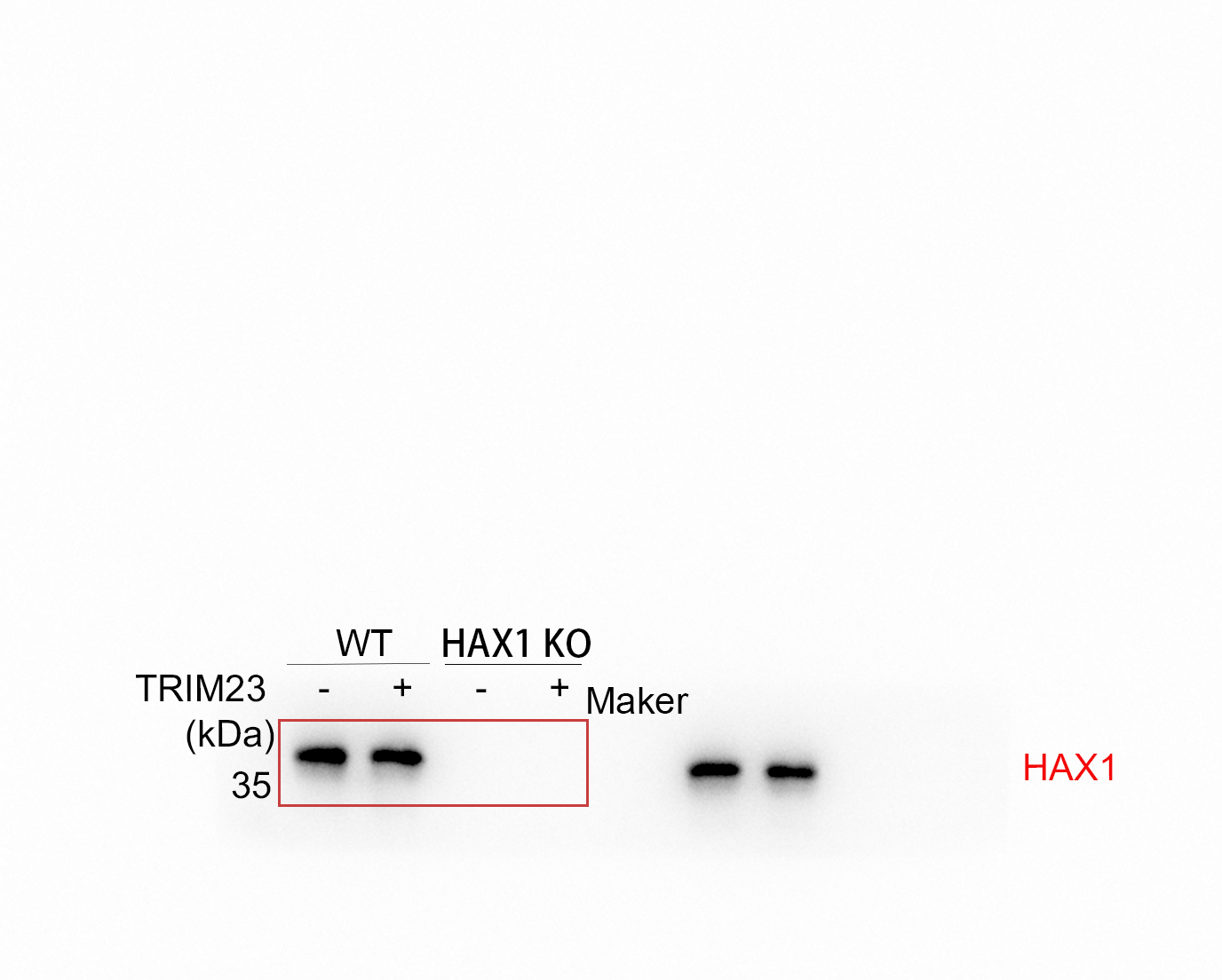

Supplement: Supplementary file 8 — Source data Fig. 5 [file 44318_2024_120_MOESM8_ESM.zip › Figure 5/5F/WCL/western-HAX1.Tif]

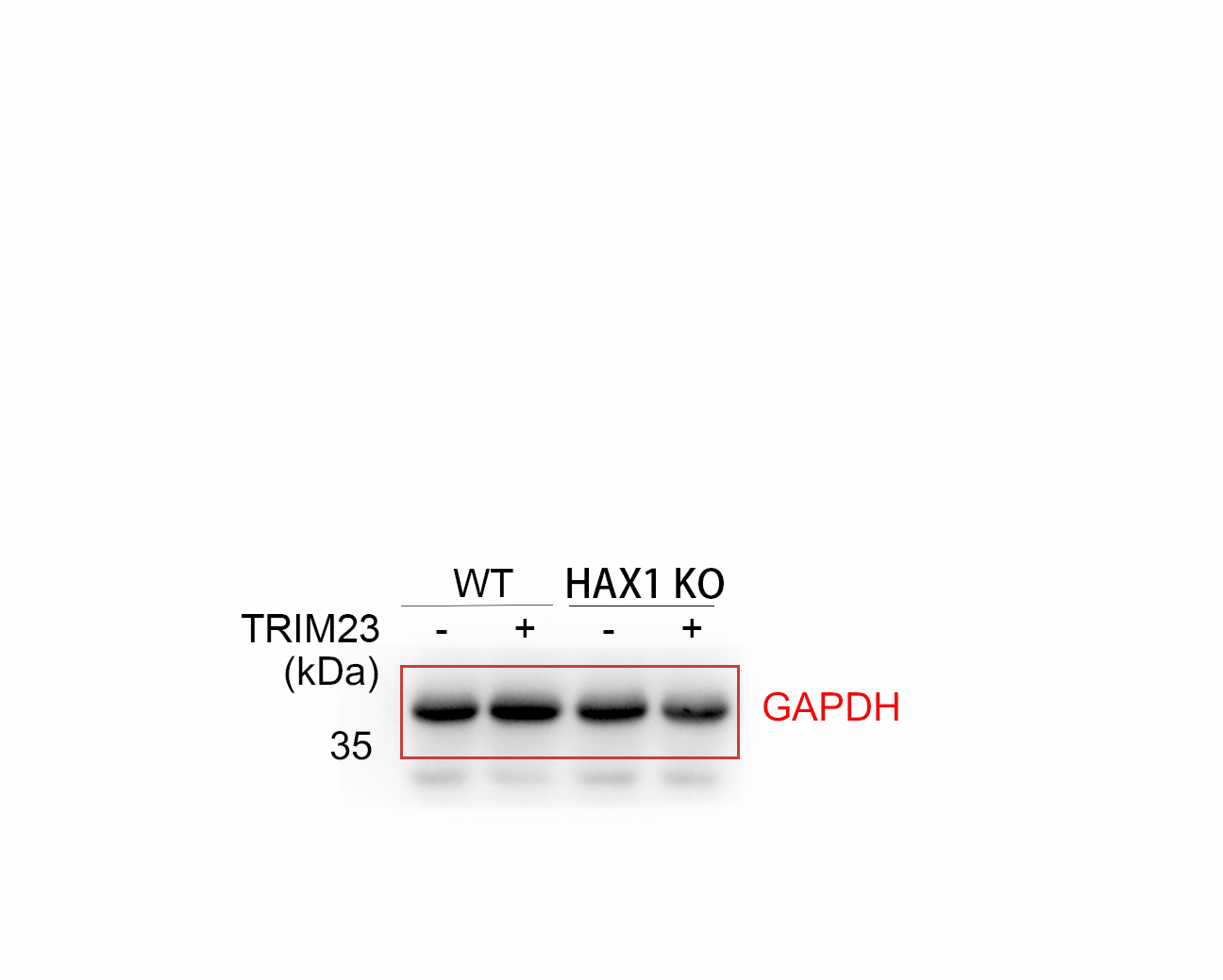

Supplement: Supplementary file 8 — Source data Fig. 5 [file 44318_2024_120_MOESM8_ESM.zip › Figure 5/5F/WCL/western-GAPDH.Tif]

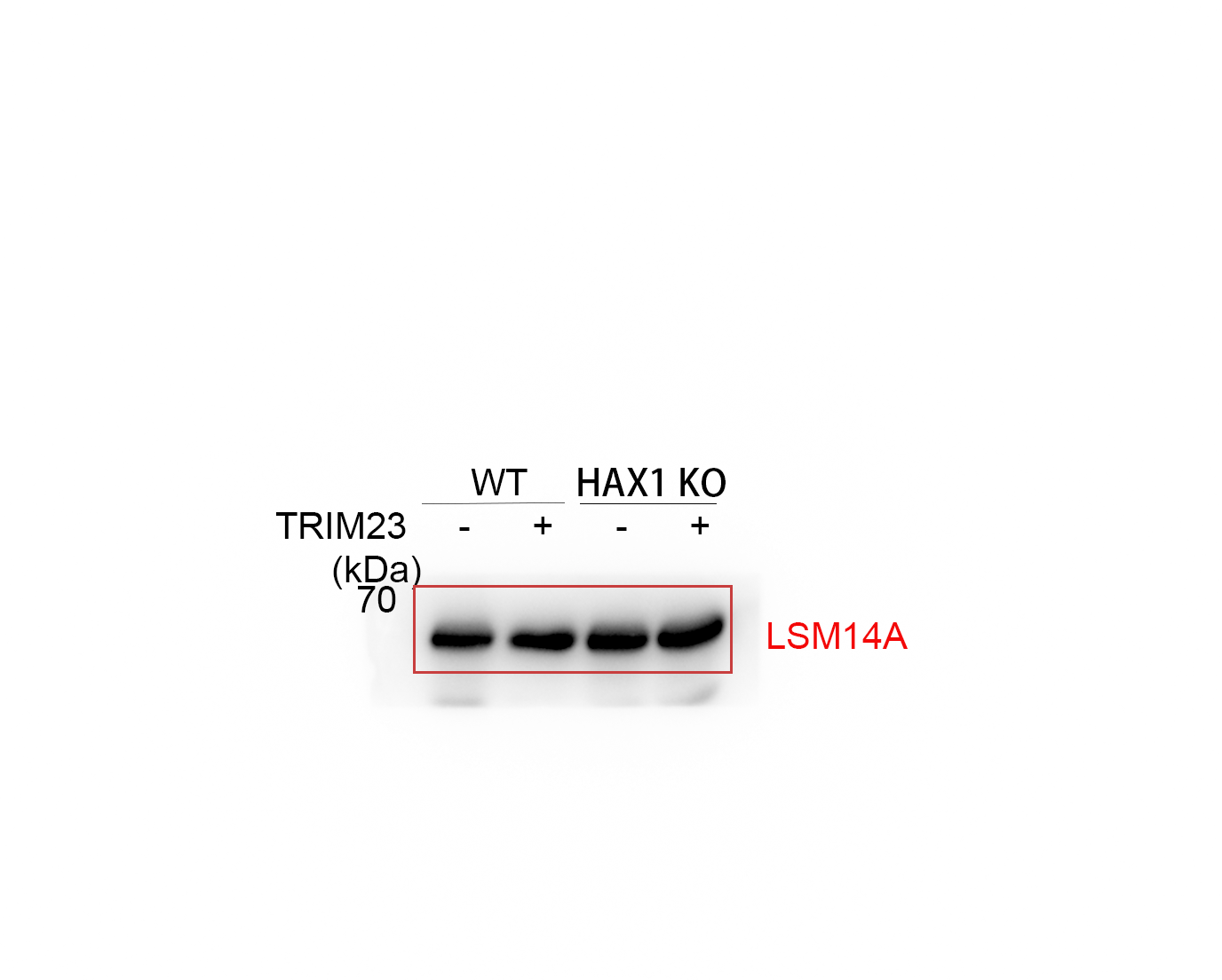

Supplement: Supplementary file 8 — Source data Fig. 5 [file 44318_2024_120_MOESM8_ESM.zip › Figure 5/5F/WCL/western-LSM14A.Tif]

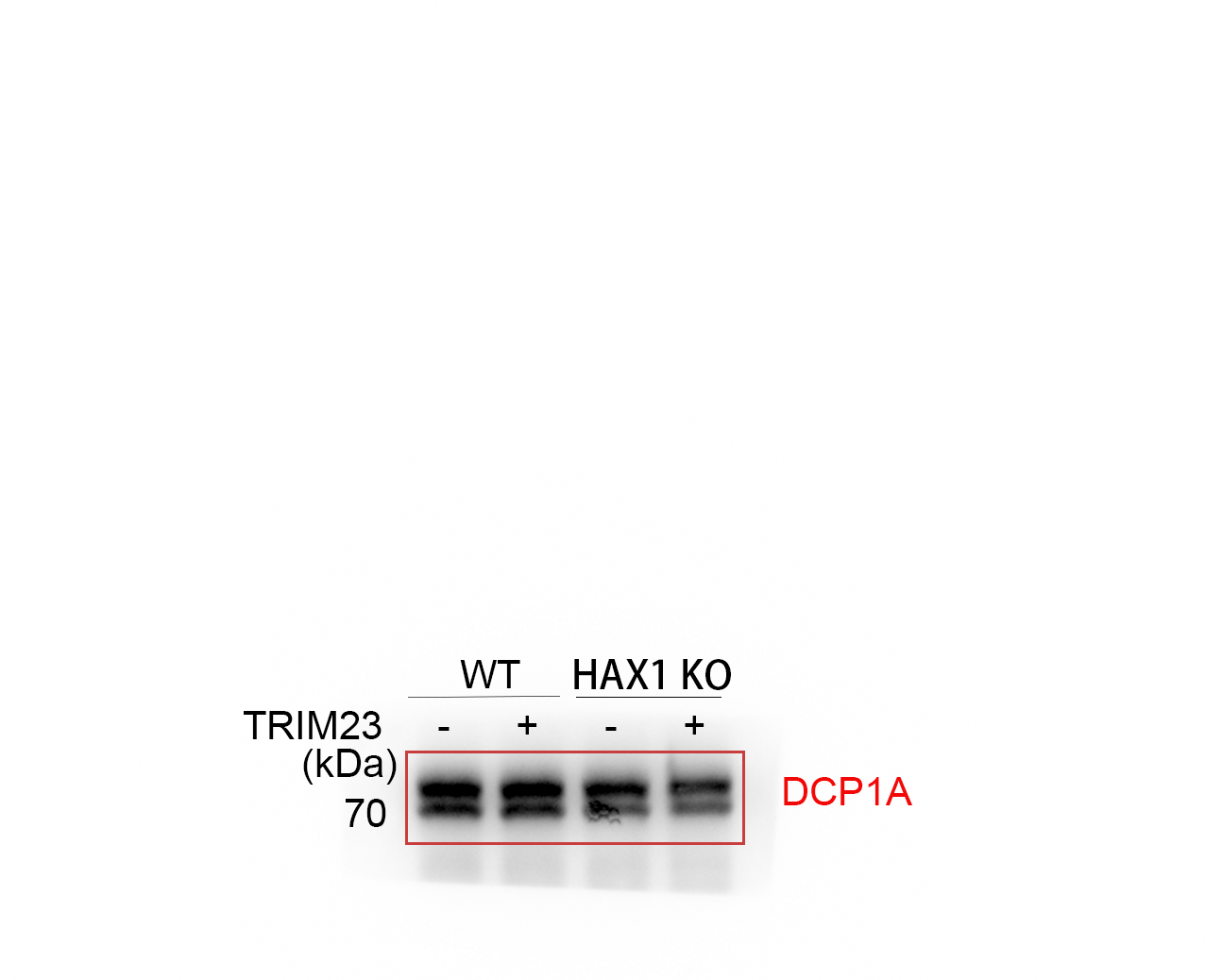

Supplement: Supplementary file 8 — Source data Fig. 5 [file 44318_2024_120_MOESM8_ESM.zip › Figure 5/5F/WCL/western-DCP1A.Tif]

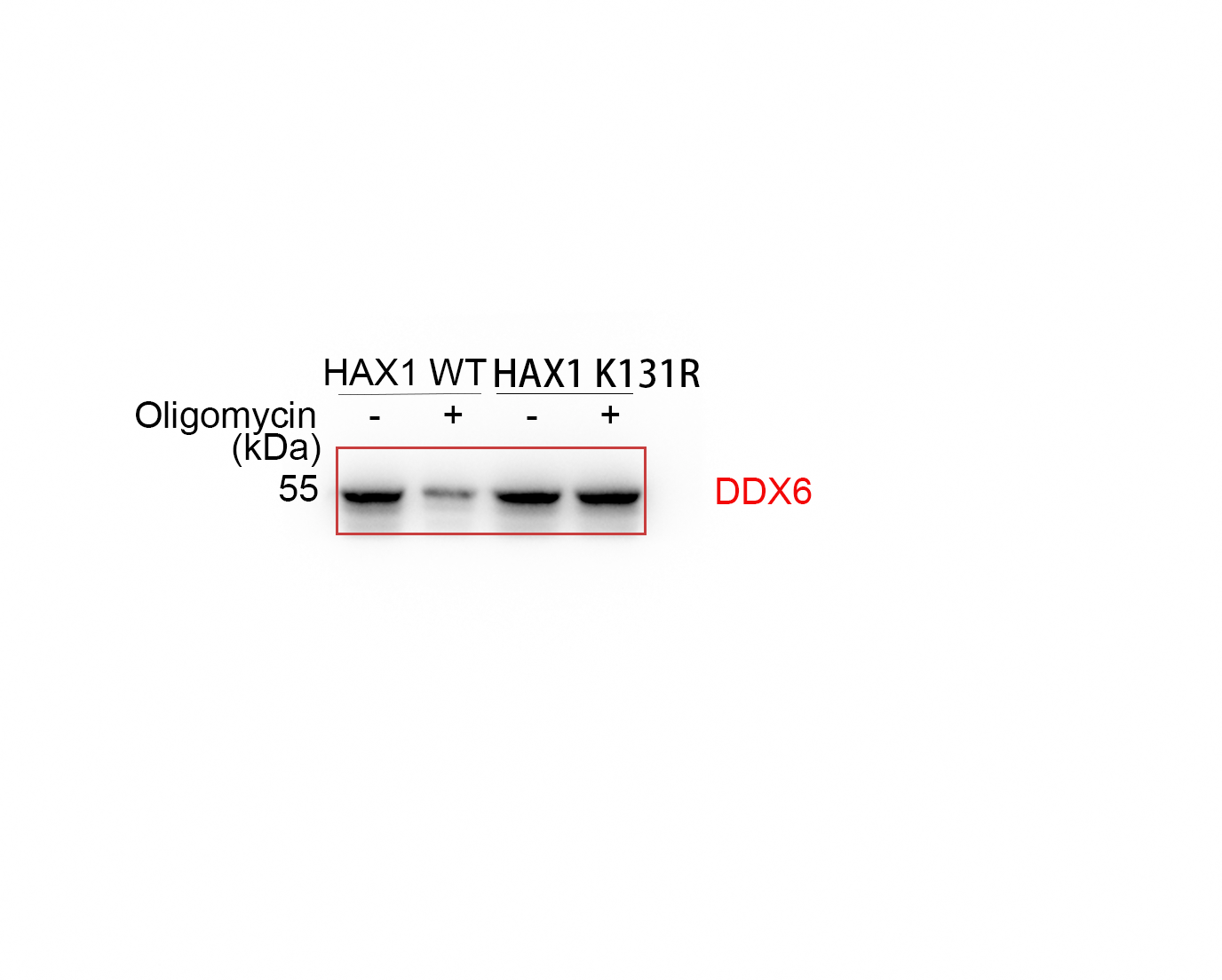

Supplement: Supplementary file 8 — Source data Fig. 5 [file 44318_2024_120_MOESM8_ESM.zip › Figure 5/5H/S2/western-DDX6.Tif]

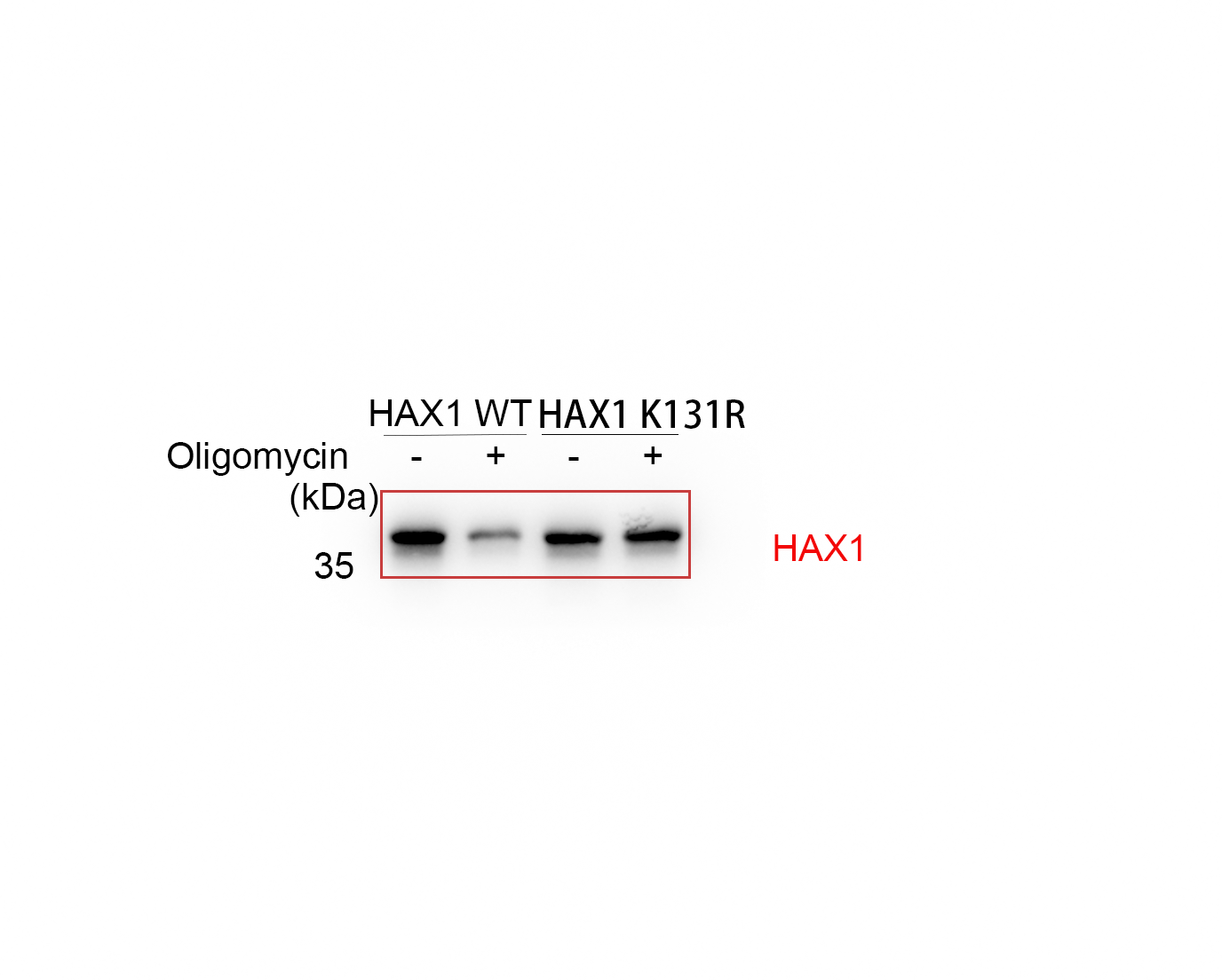

Supplement: Supplementary file 8 — Source data Fig. 5 [file 44318_2024_120_MOESM8_ESM.zip › Figure 5/5H/S2/western-HAX1.Tif]

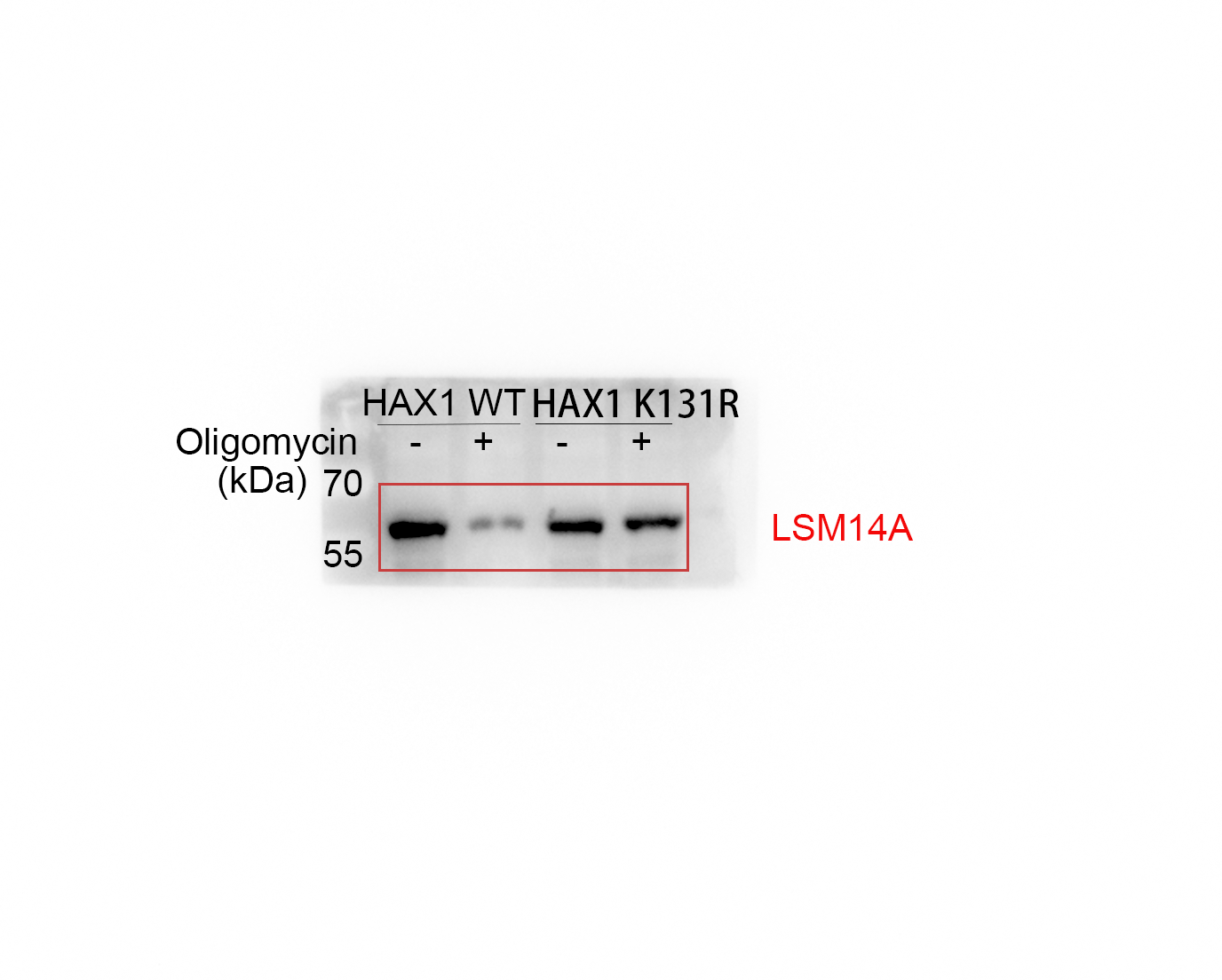

Supplement: Supplementary file 8 — Source data Fig. 5 [file 44318_2024_120_MOESM8_ESM.zip › Figure 5/5H/S2/western-LSM14A.Tif]

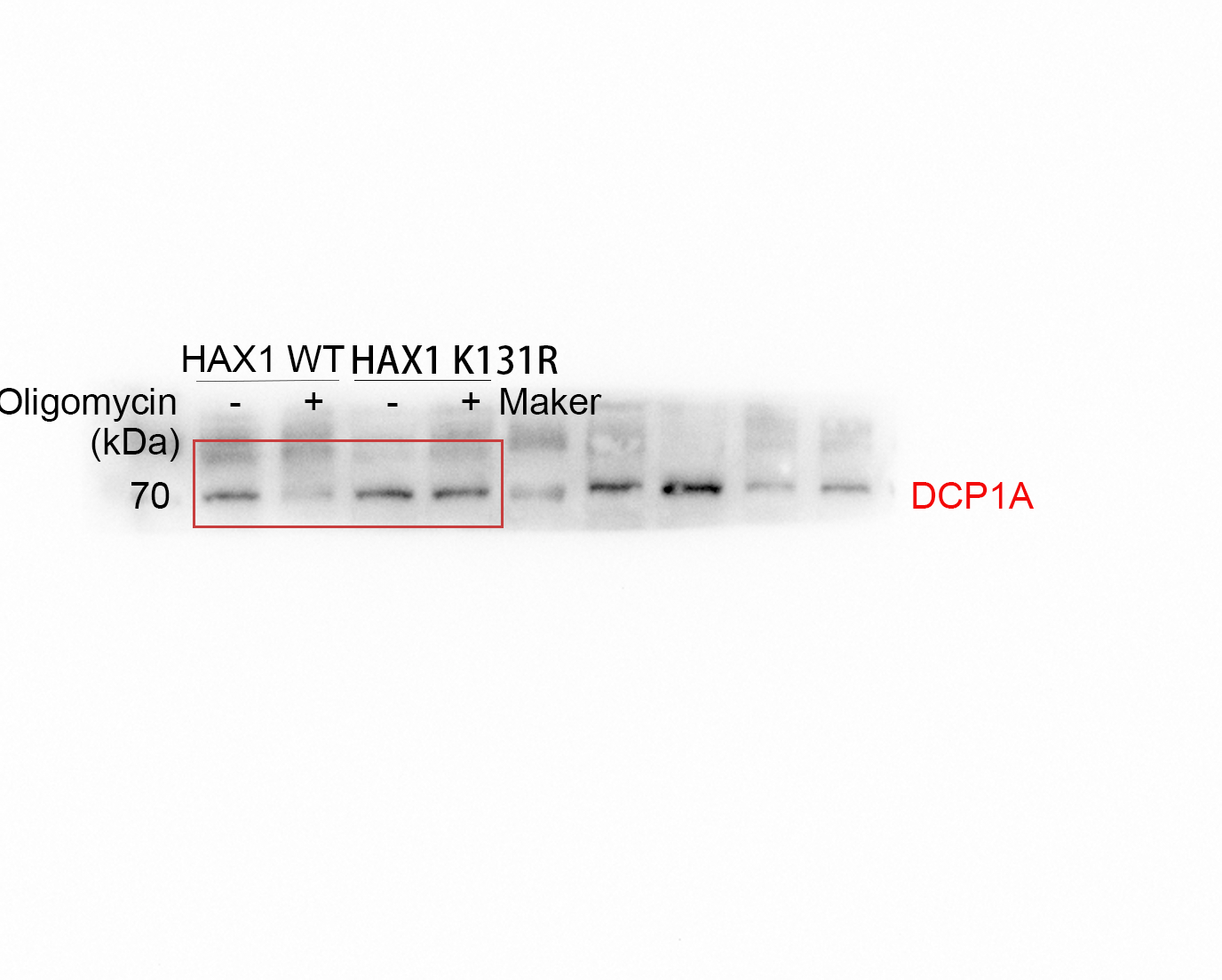

Supplement: Supplementary file 8 — Source data Fig. 5 [file 44318_2024_120_MOESM8_ESM.zip › Figure 5/5H/S2/western-DCP1A.Tif]

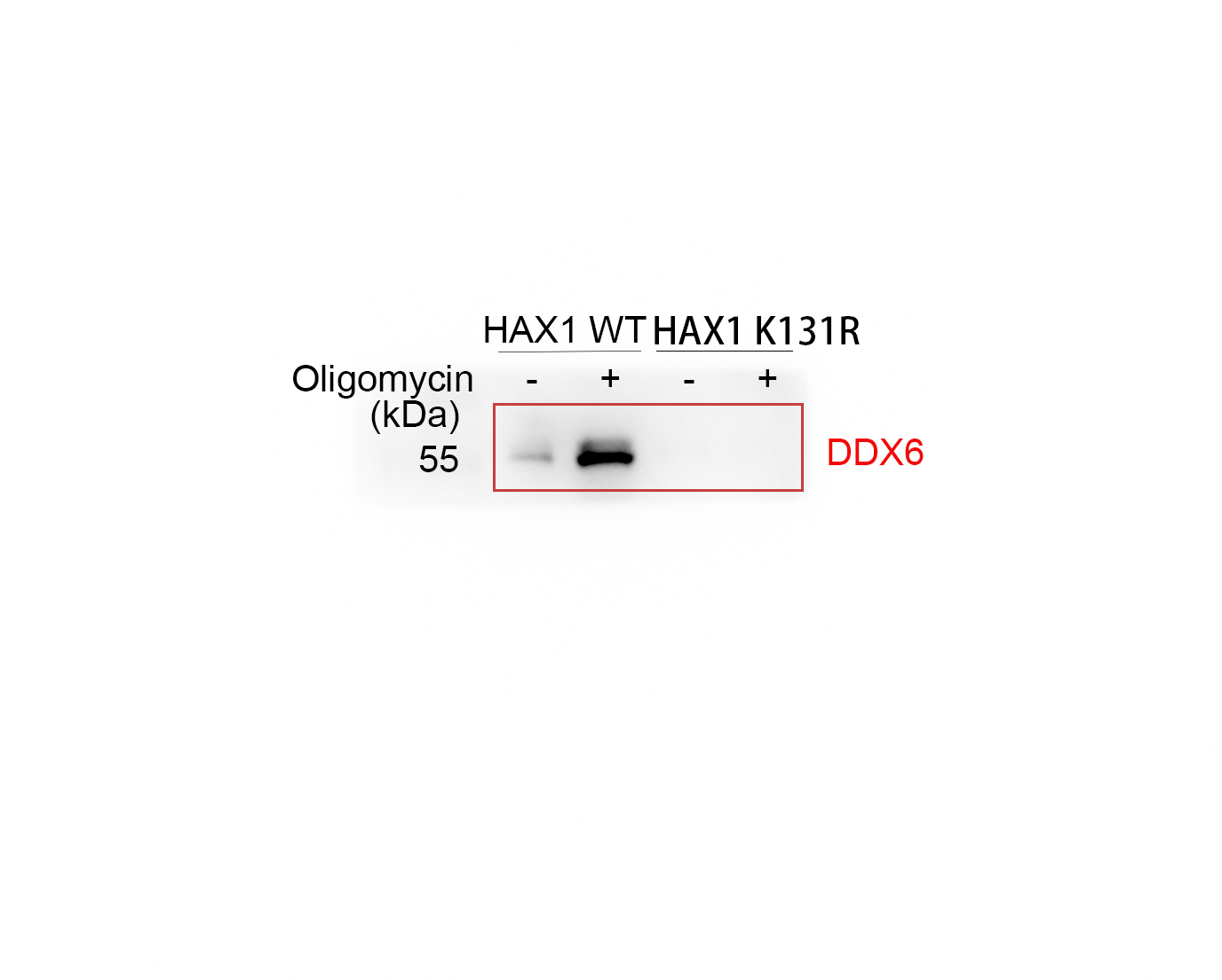

Supplement: Supplementary file 8 — Source data Fig. 5 [file 44318_2024_120_MOESM8_ESM.zip › Figure 5/5H/P2/western-DDX6.Tif]

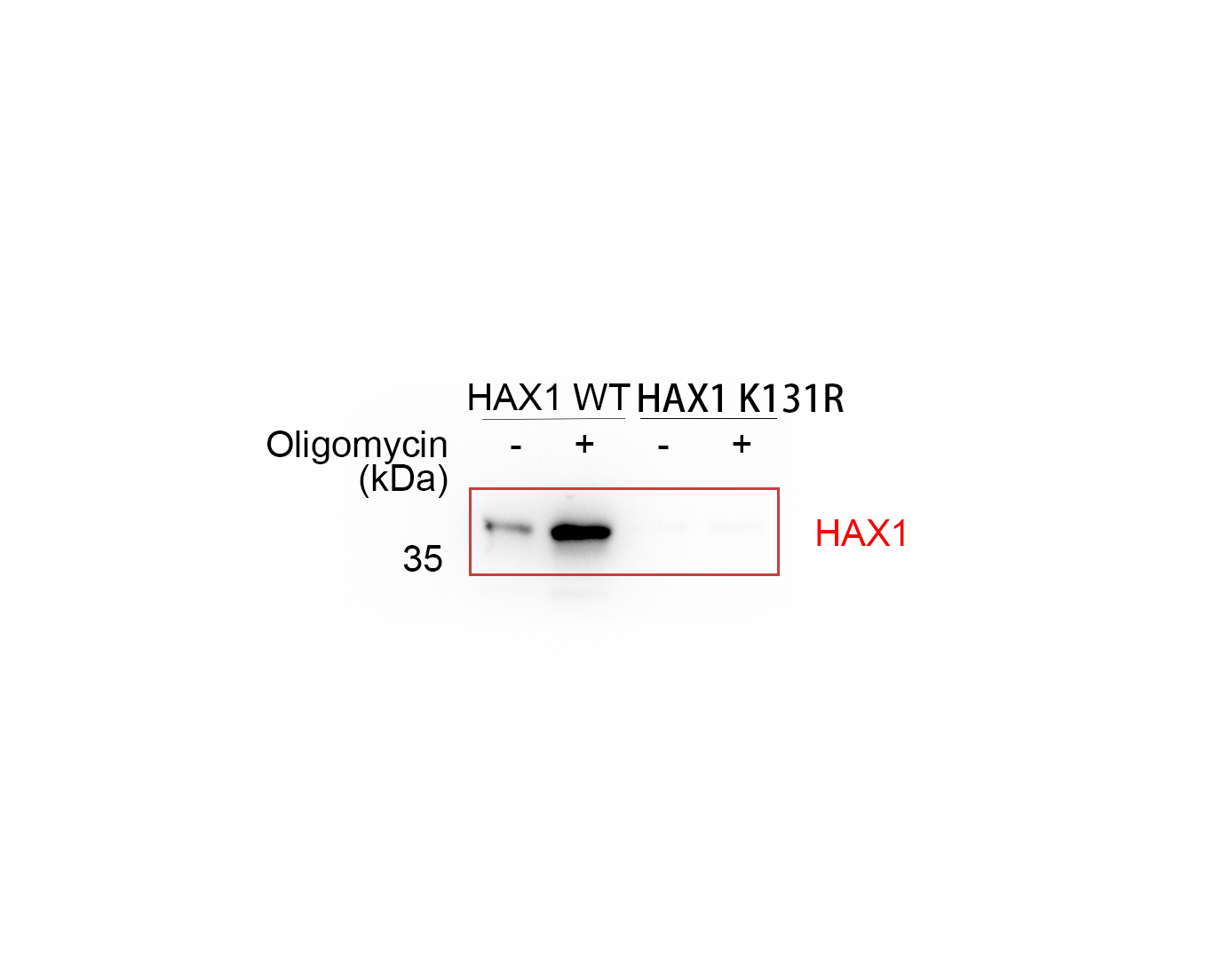

Supplement: Supplementary file 8 — Source data Fig. 5 [file 44318_2024_120_MOESM8_ESM.zip › Figure 5/5H/P2/western-HAX1.Tif]

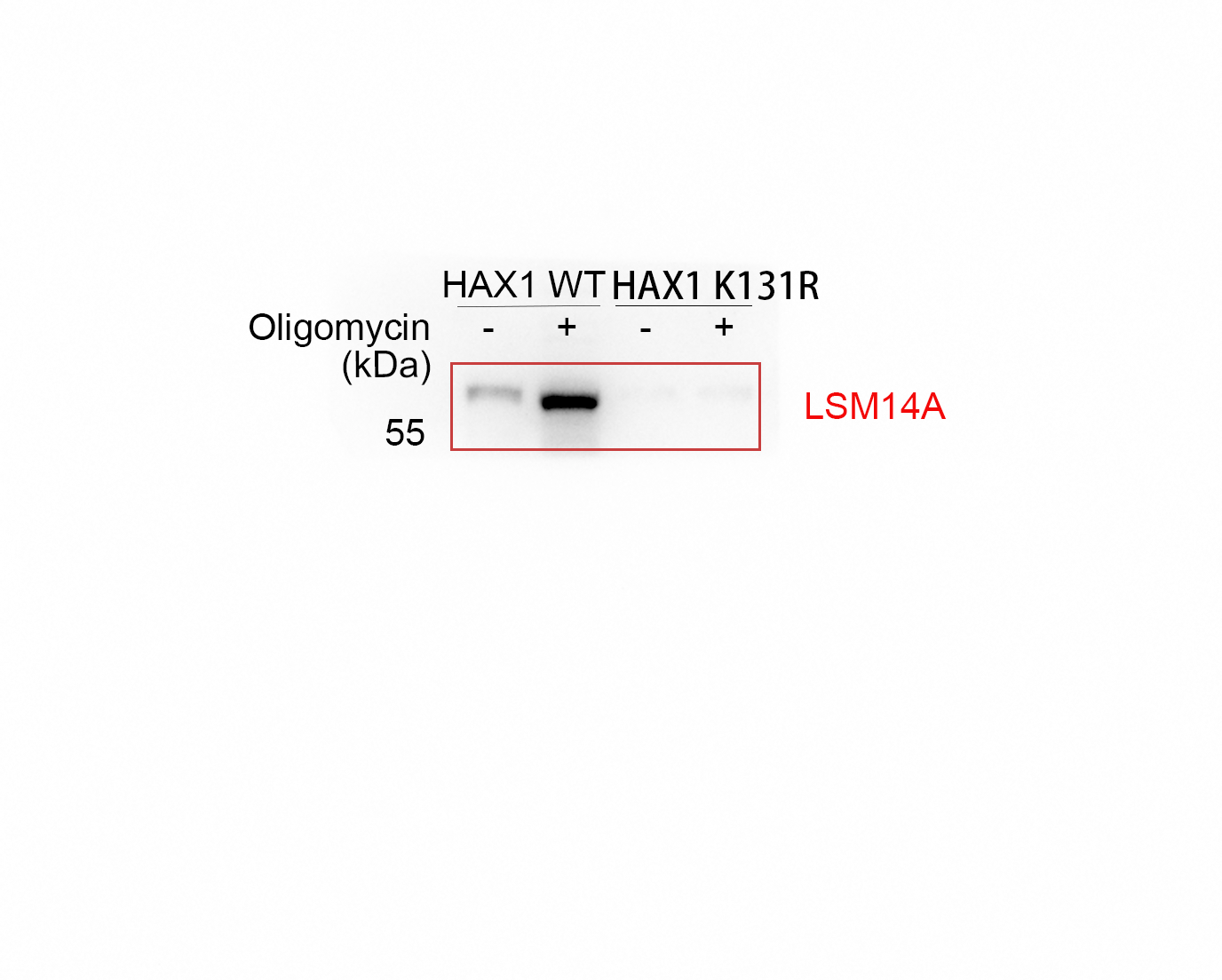

Supplement: Supplementary file 8 — Source data Fig. 5 [file 44318_2024_120_MOESM8_ESM.zip › Figure 5/5H/P2/western-LSM14A.Tif]

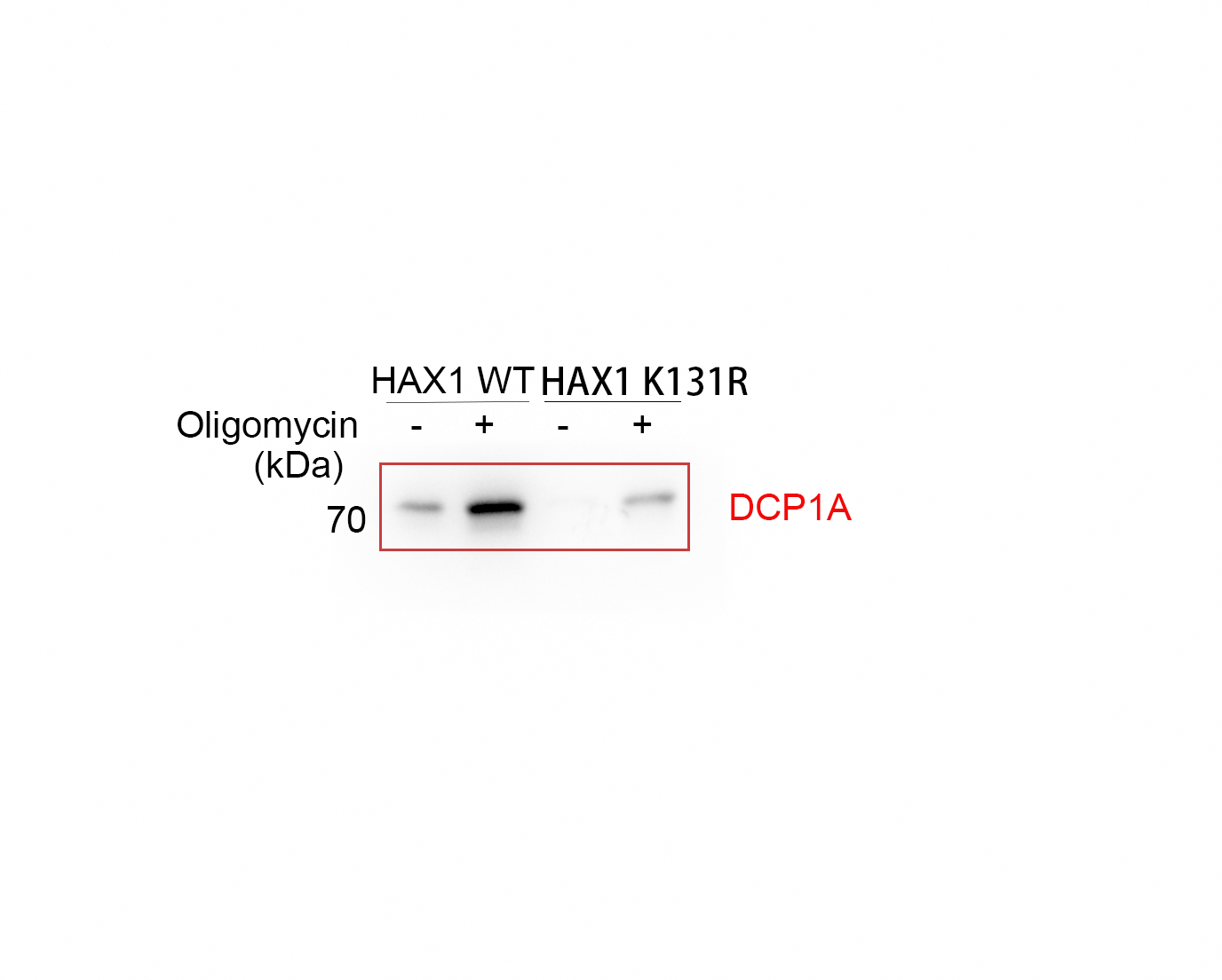

Supplement: Supplementary file 8 — Source data Fig. 5 [file 44318_2024_120_MOESM8_ESM.zip › Figure 5/5H/P2/western-DCP1A.Tif]

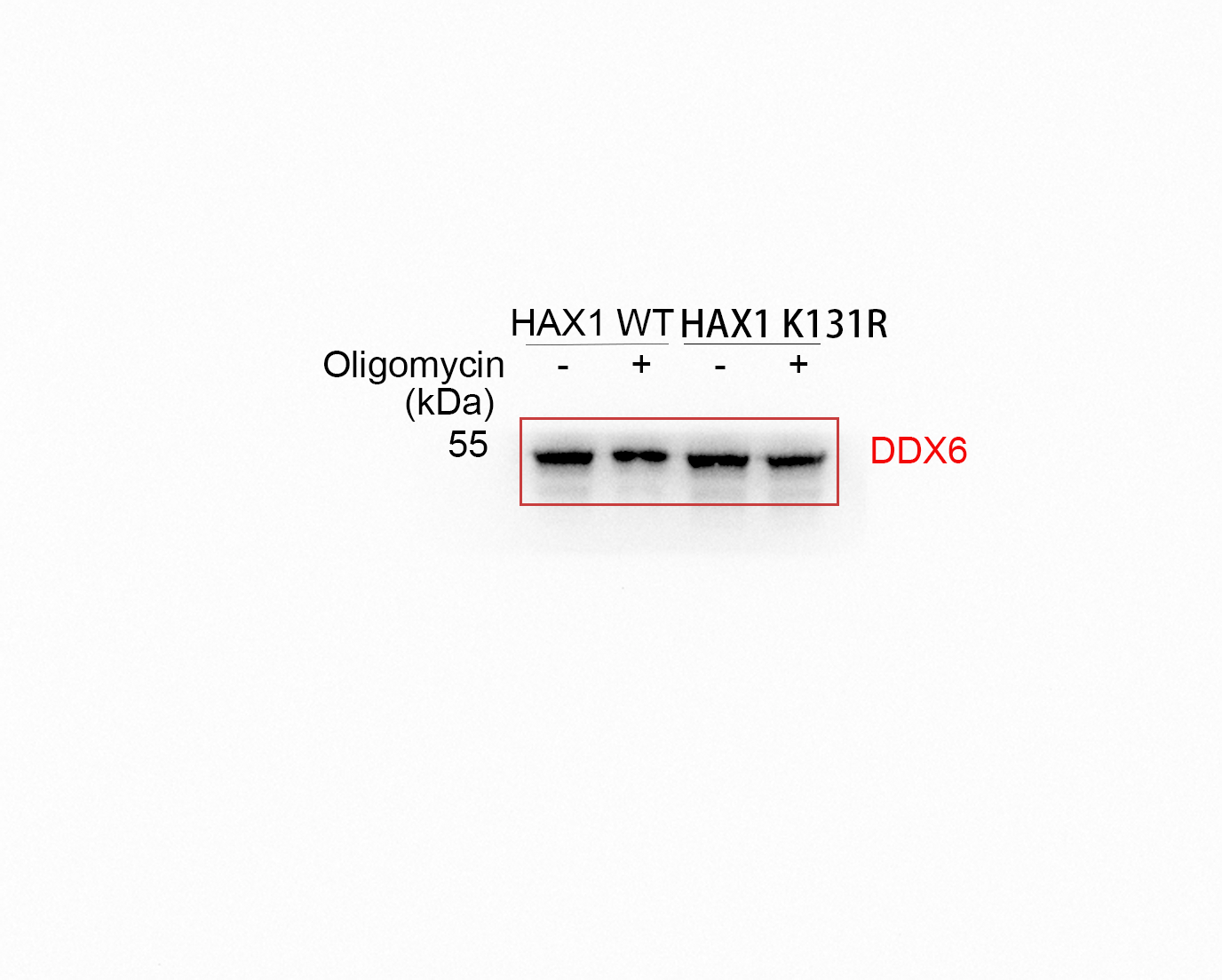

Supplement: Supplementary file 8 — Source data Fig. 5 [file 44318_2024_120_MOESM8_ESM.zip › Figure 5/5H/WCL/western-DDX6.Tif]

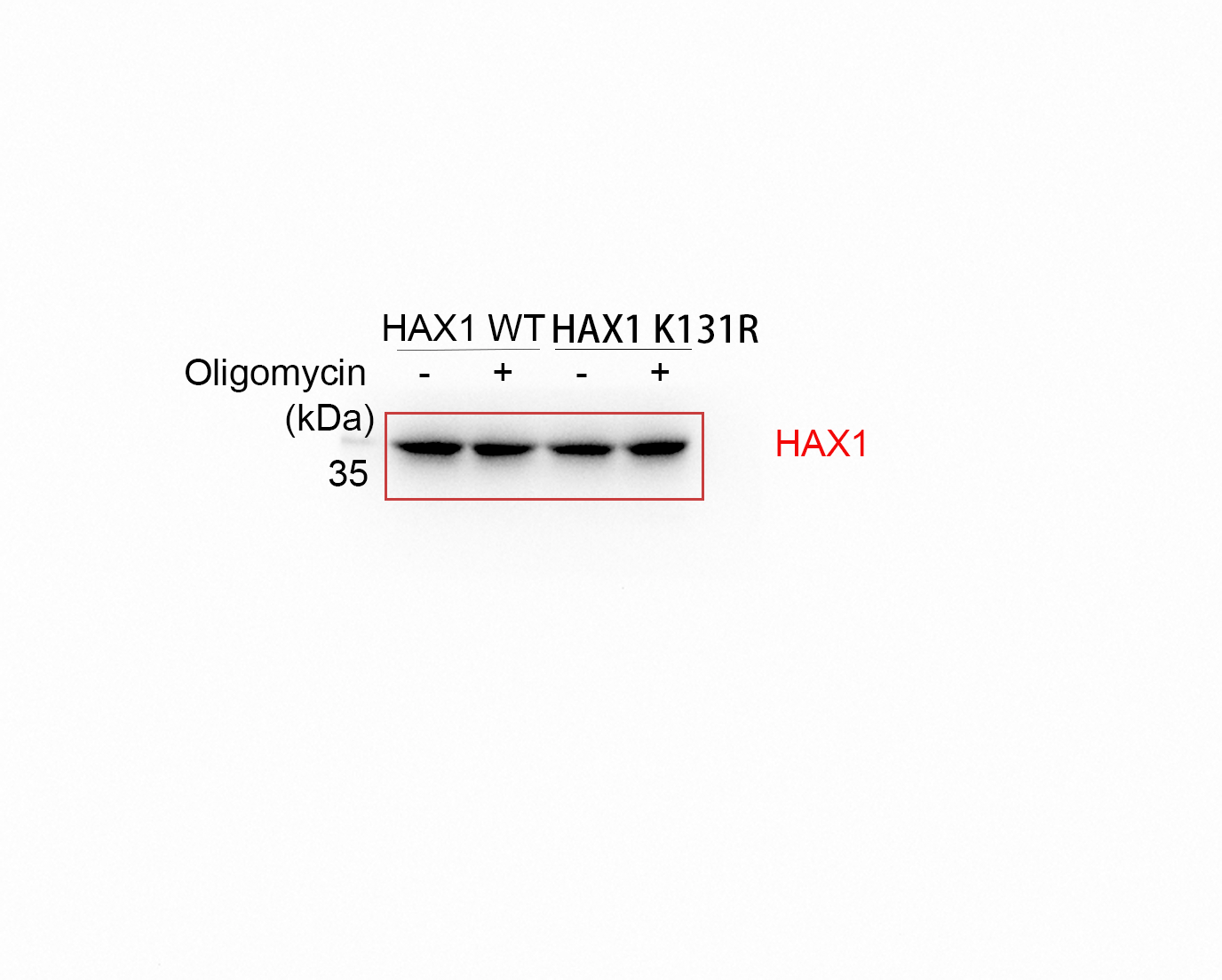

Supplement: Supplementary file 8 — Source data Fig. 5 [file 44318_2024_120_MOESM8_ESM.zip › Figure 5/5H/WCL/western-HAX1.Tif]

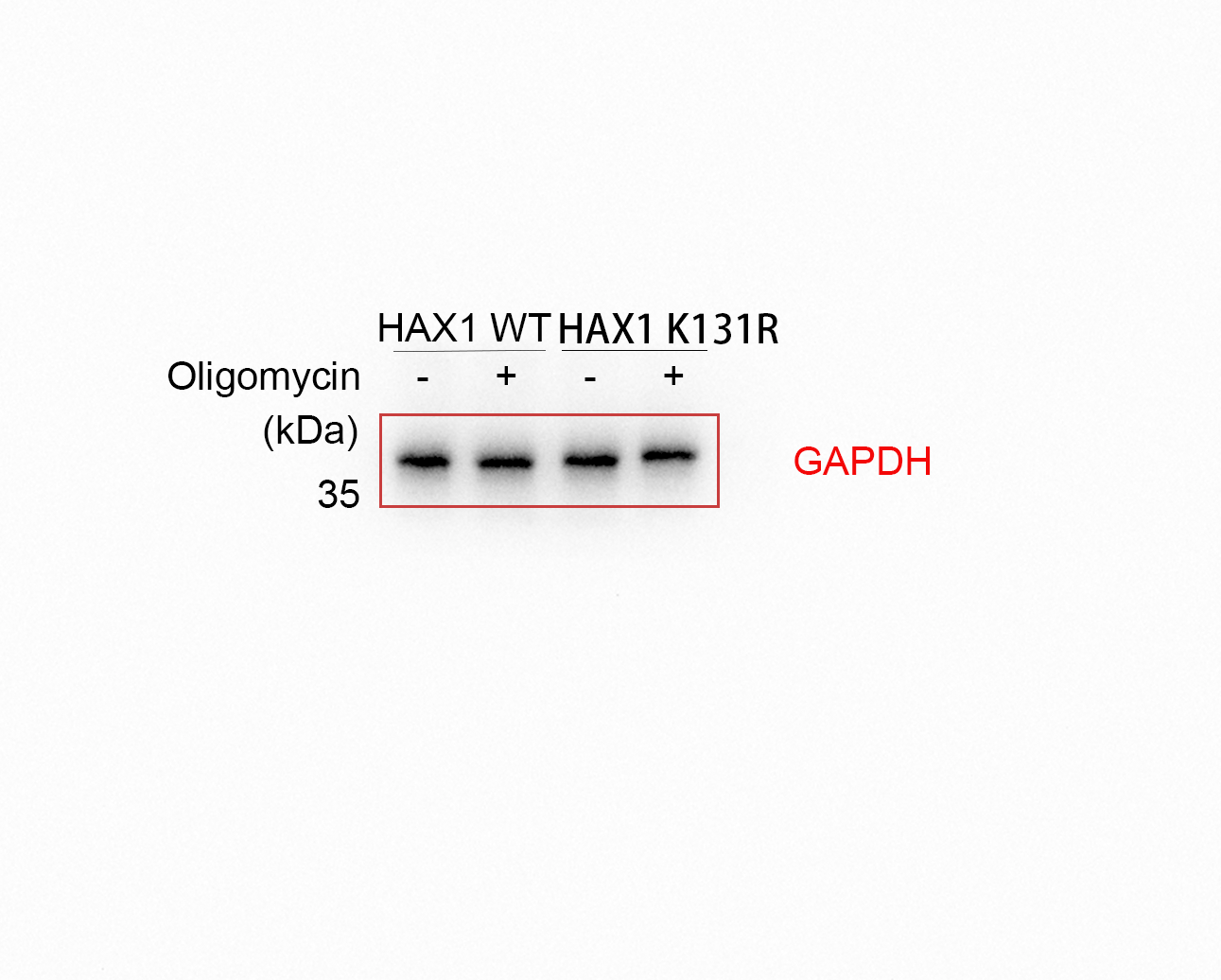

Supplement: Supplementary file 8 — Source data Fig. 5 [file 44318_2024_120_MOESM8_ESM.zip › Figure 5/5H/WCL/western-GAPDH.Tif]

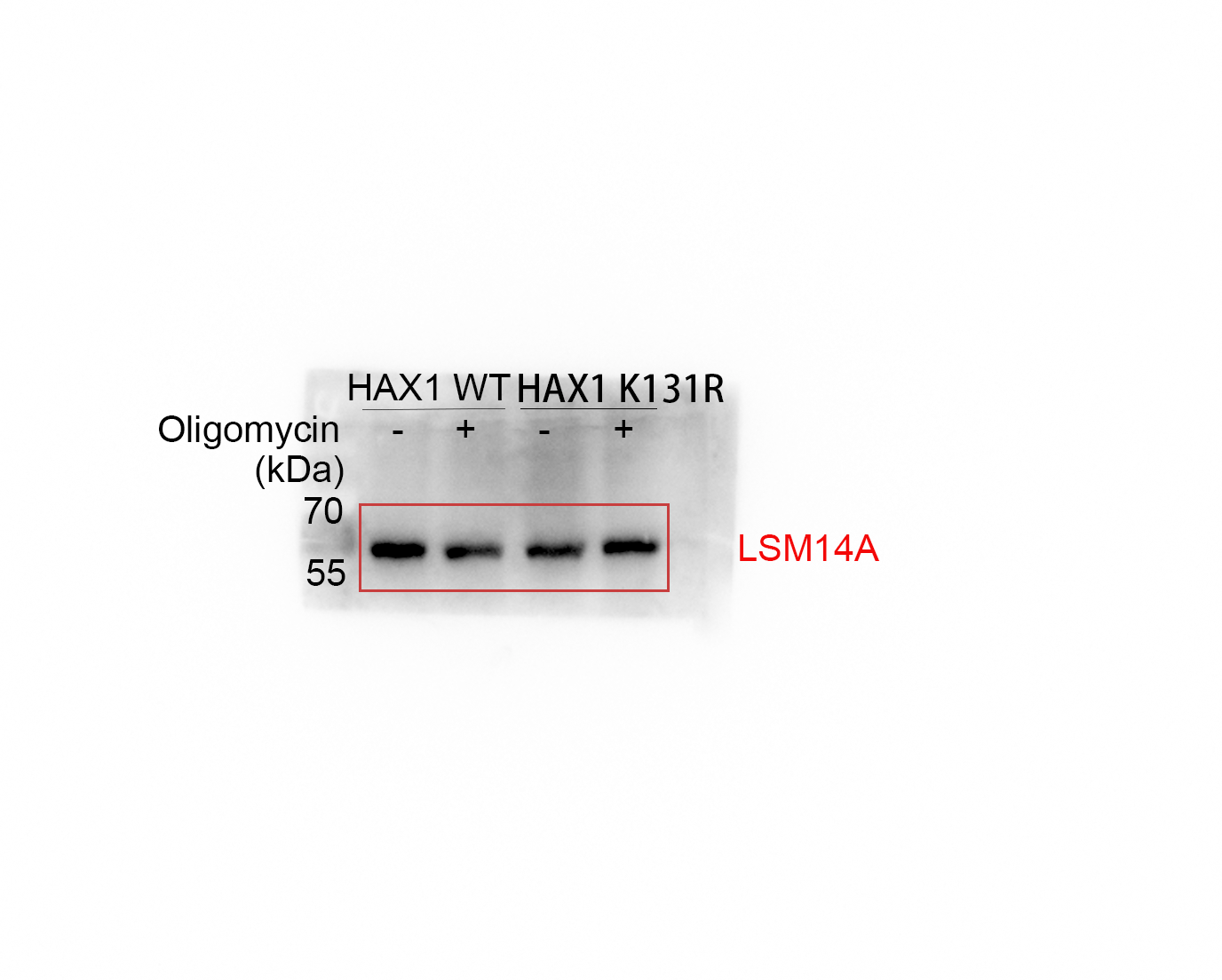

Supplement: Supplementary file 8 — Source data Fig. 5 [file 44318_2024_120_MOESM8_ESM.zip › Figure 5/5H/WCL/western-LSM14A.Tif]

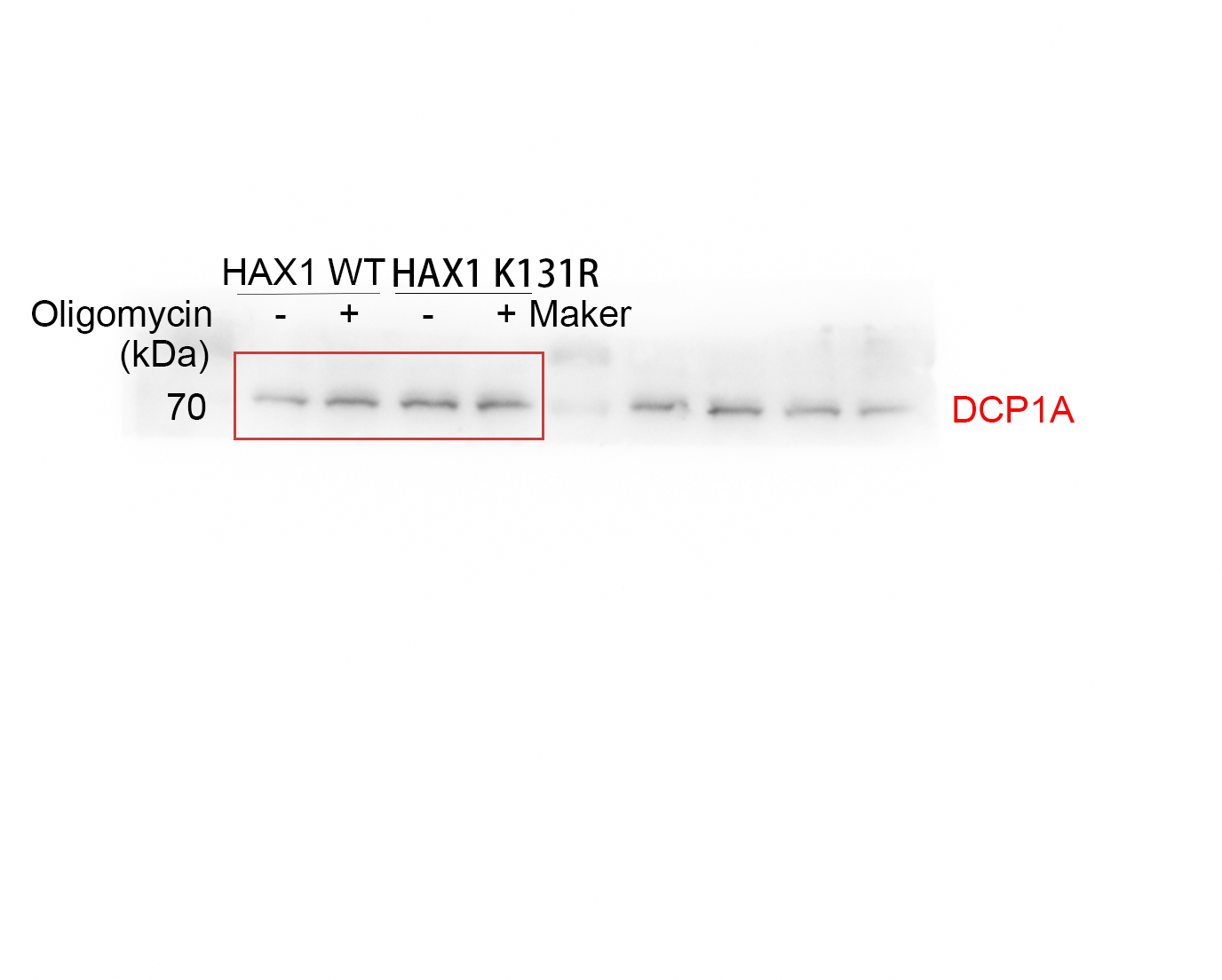

Supplement: Supplementary file 8 — Source data Fig. 5 [file 44318_2024_120_MOESM8_ESM.zip › Figure 5/5H/WCL/western-DCP1A.Tif]

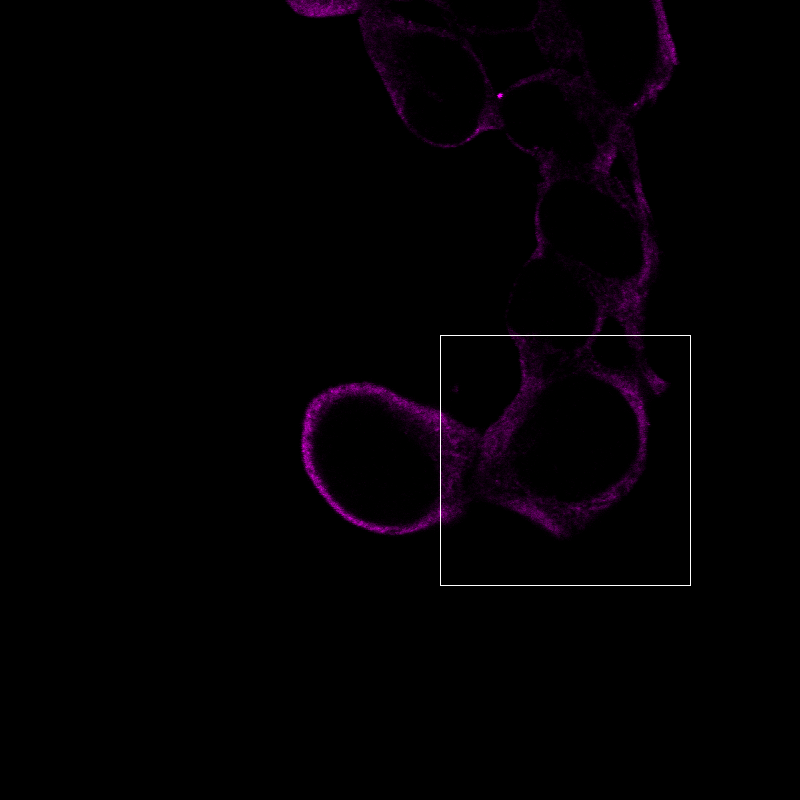

Supplement: Supplementary file 8 — Source data Fig. 5 [file 44318_2024_120_MOESM8_ESM.zip › Figure 5/5A/Mock/HAX1 KO/DCP1A.tif]

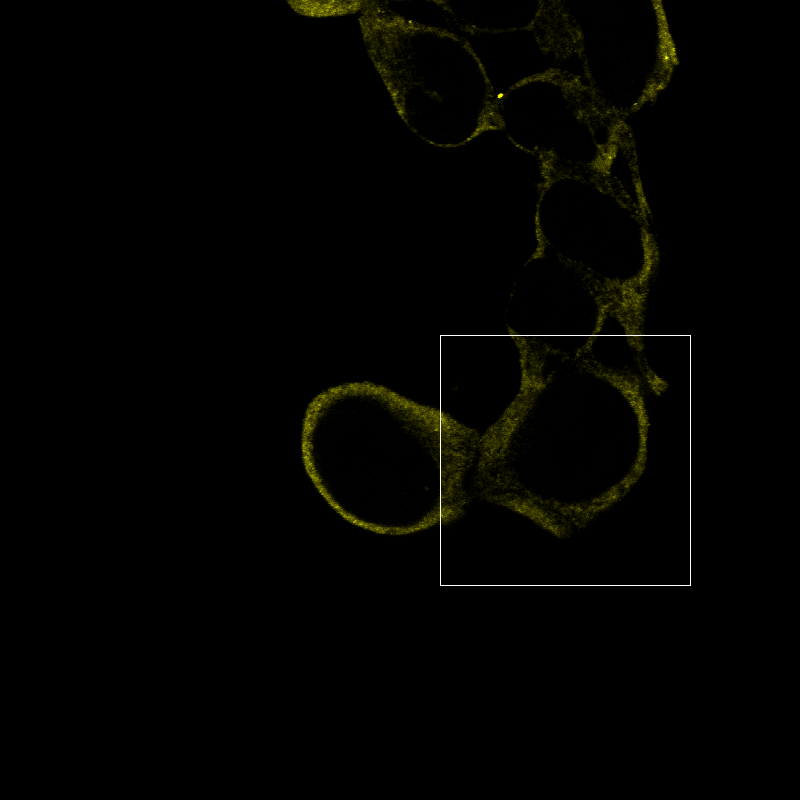

Supplement: Supplementary file 8 — Source data Fig. 5 [file 44318_2024_120_MOESM8_ESM.zip › Figure 5/5A/Mock/HAX1 KO/LSM14A.tif]

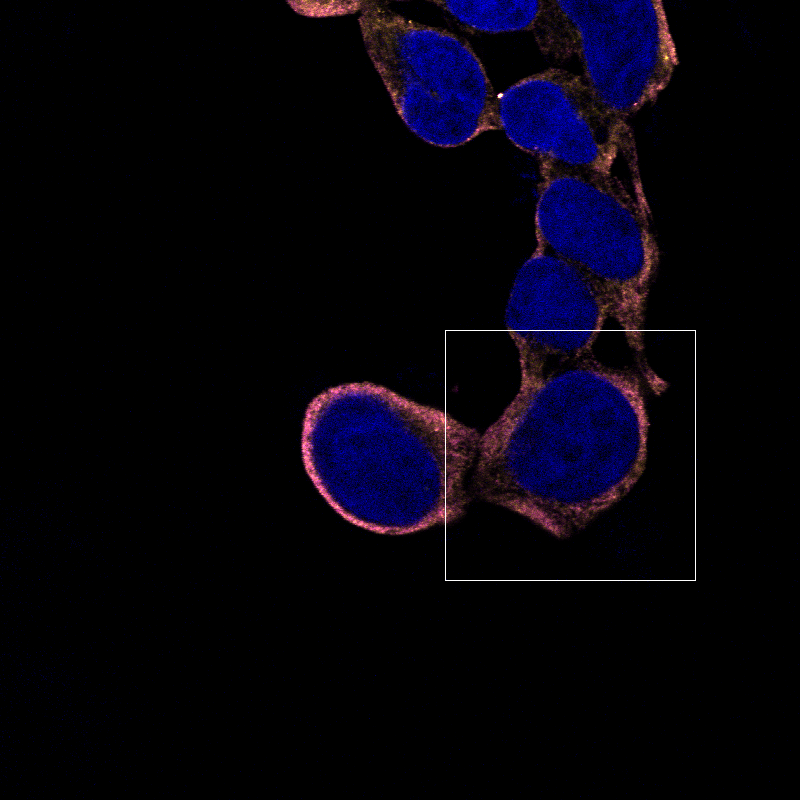

Supplement: Supplementary file 8 — Source data Fig. 5 [file 44318_2024_120_MOESM8_ESM.zip › Figure 5/5A/Mock/HAX1 KO/Merge.tif]

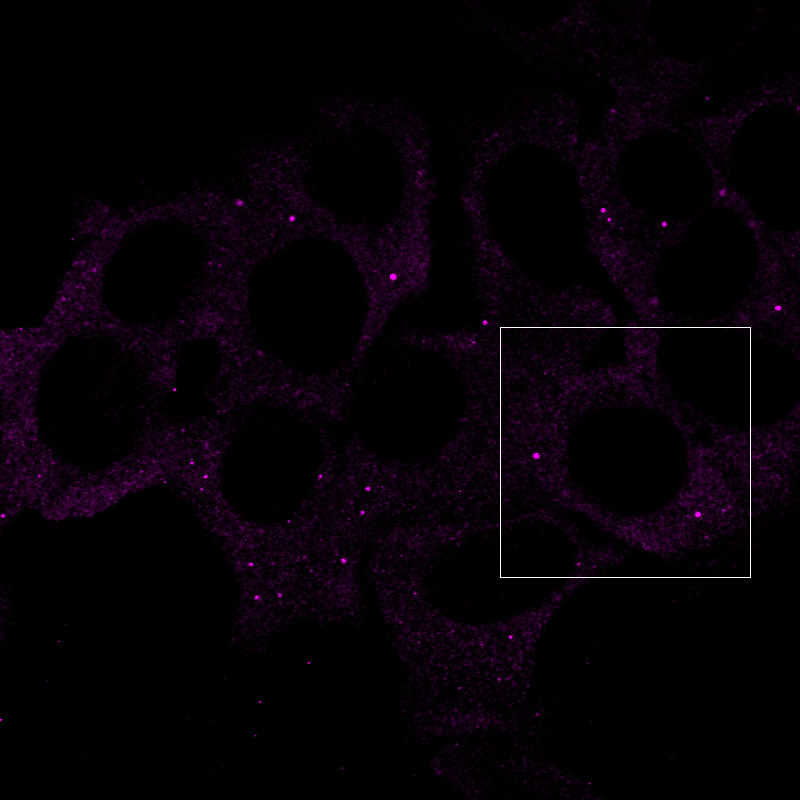

Supplement: Supplementary file 8 — Source data Fig. 5 [file 44318_2024_120_MOESM8_ESM.zip › Figure 5/5A/Mock/WT/DCP1A.tif]

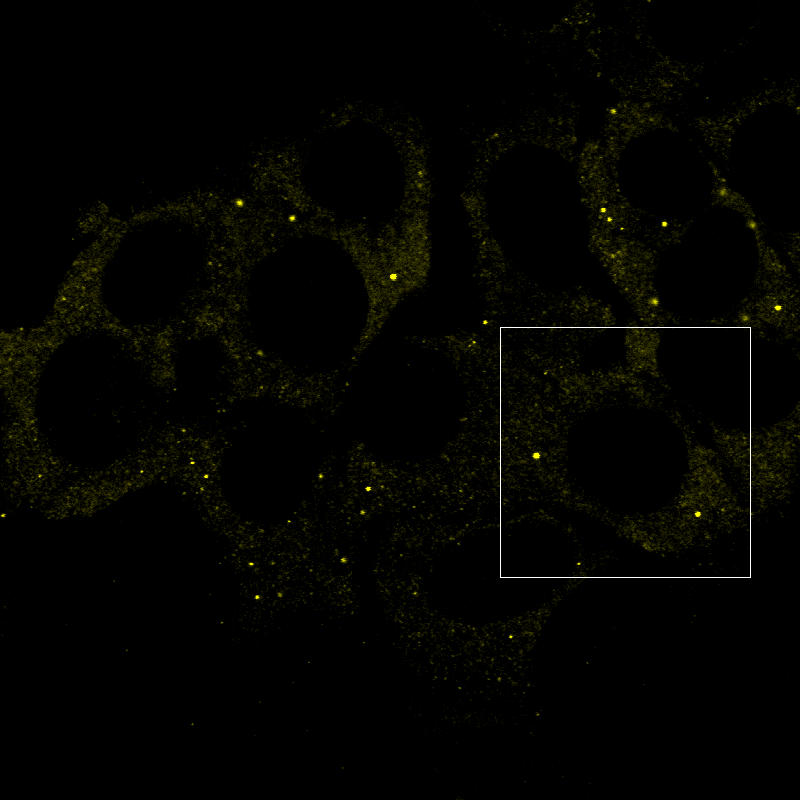

Supplement: Supplementary file 8 — Source data Fig. 5 [file 44318_2024_120_MOESM8_ESM.zip › Figure 5/5A/Mock/WT/LSM14A.tif]

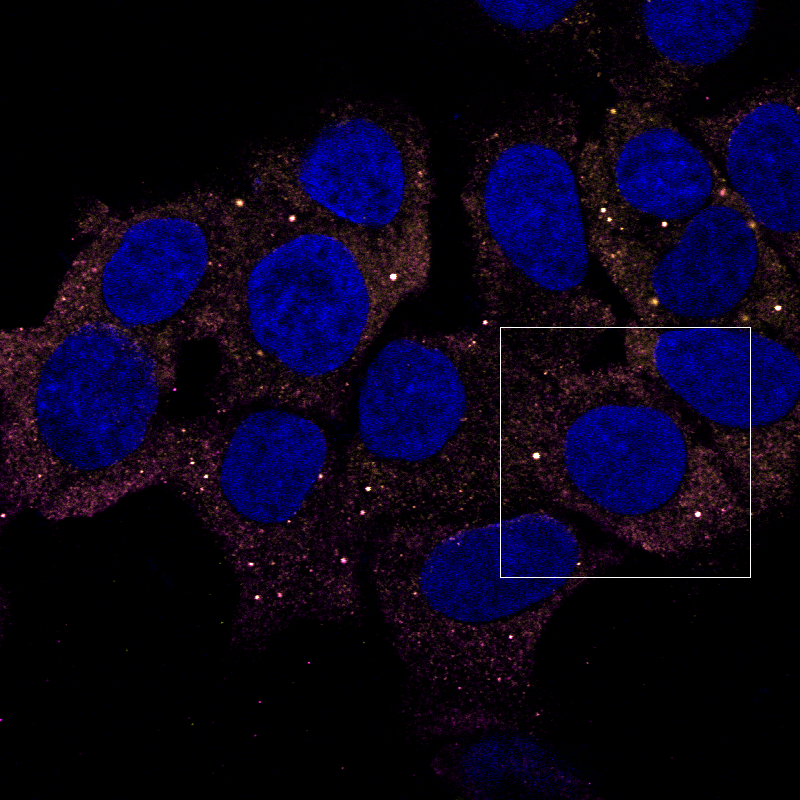

Supplement: Supplementary file 8 — Source data Fig. 5 [file 44318_2024_120_MOESM8_ESM.zip › Figure 5/5A/Mock/WT/Merge.tif]

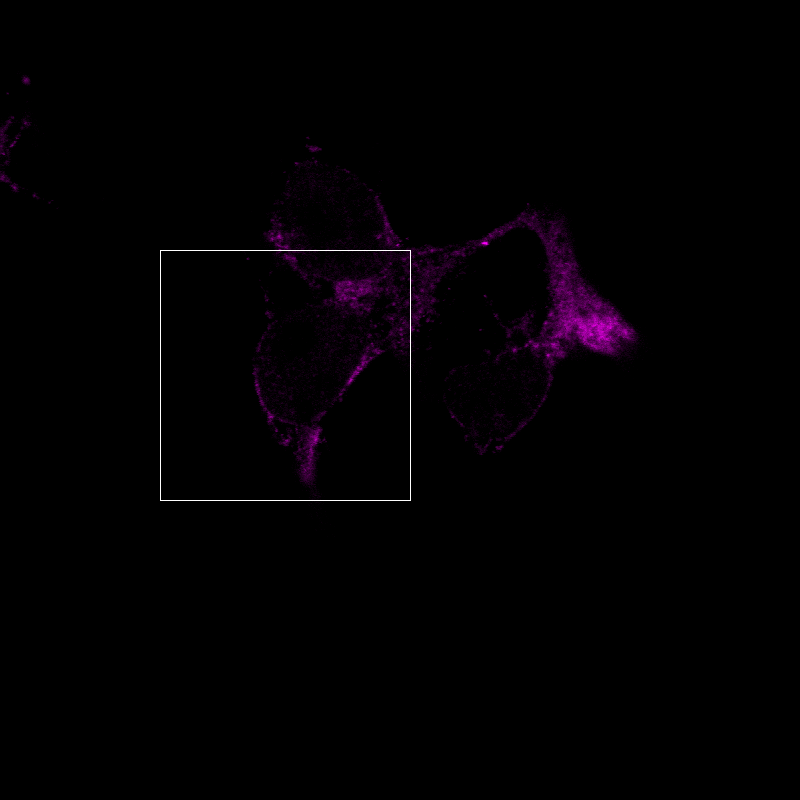

Supplement: Supplementary file 8 — Source data Fig. 5 [file 44318_2024_120_MOESM8_ESM.zip › Figure 5/5A/Oligomycin/HAX1 KO/DCP1A.tif]

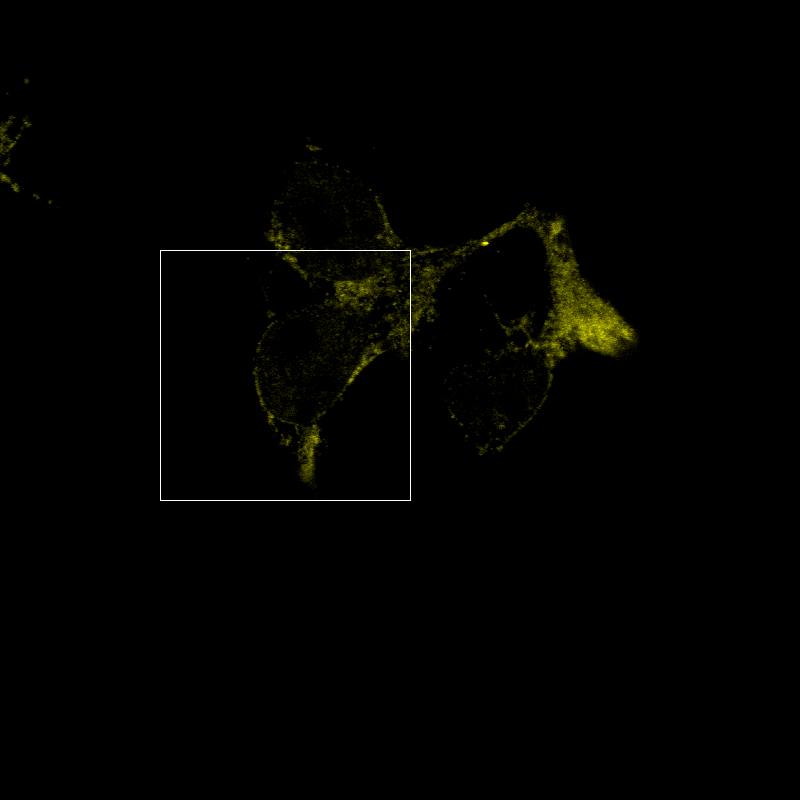

Supplement: Supplementary file 8 — Source data Fig. 5 [file 44318_2024_120_MOESM8_ESM.zip › Figure 5/5A/Oligomycin/HAX1 KO/LSM14A.tif]

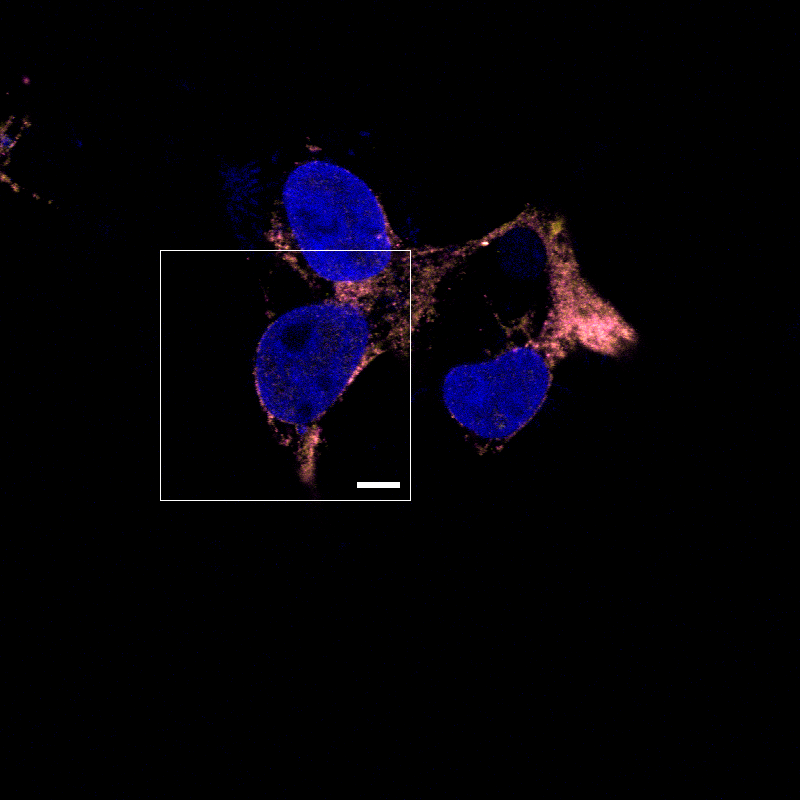

Supplement: Supplementary file 8 — Source data Fig. 5 [file 44318_2024_120_MOESM8_ESM.zip › Figure 5/5A/Oligomycin/HAX1 KO/Merge.tif]

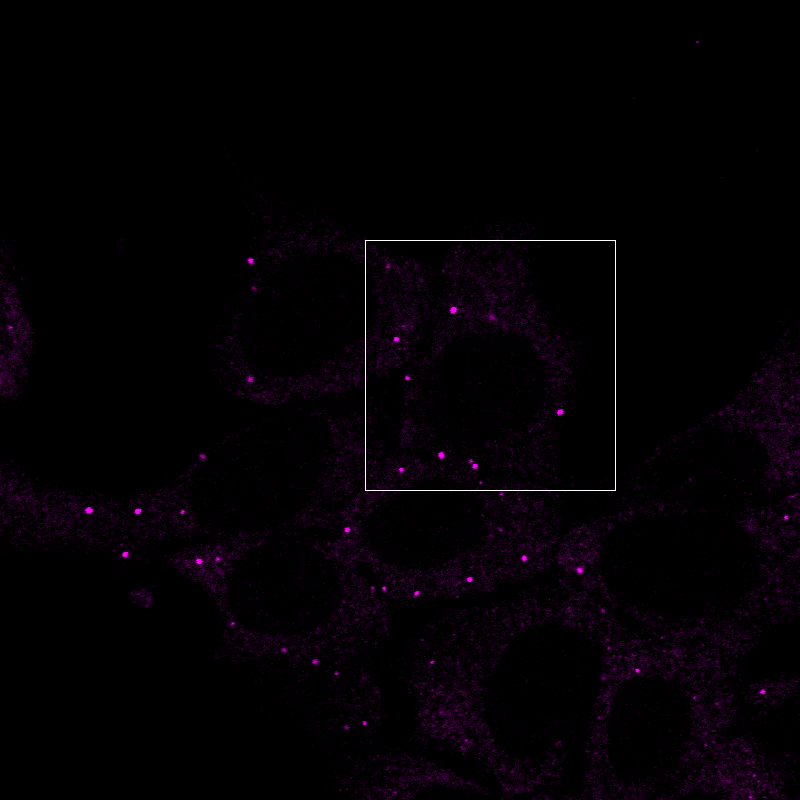

Supplement: Supplementary file 8 — Source data Fig. 5 [file 44318_2024_120_MOESM8_ESM.zip › Figure 5/5A/Oligomycin/WT/DCP1A.tif]

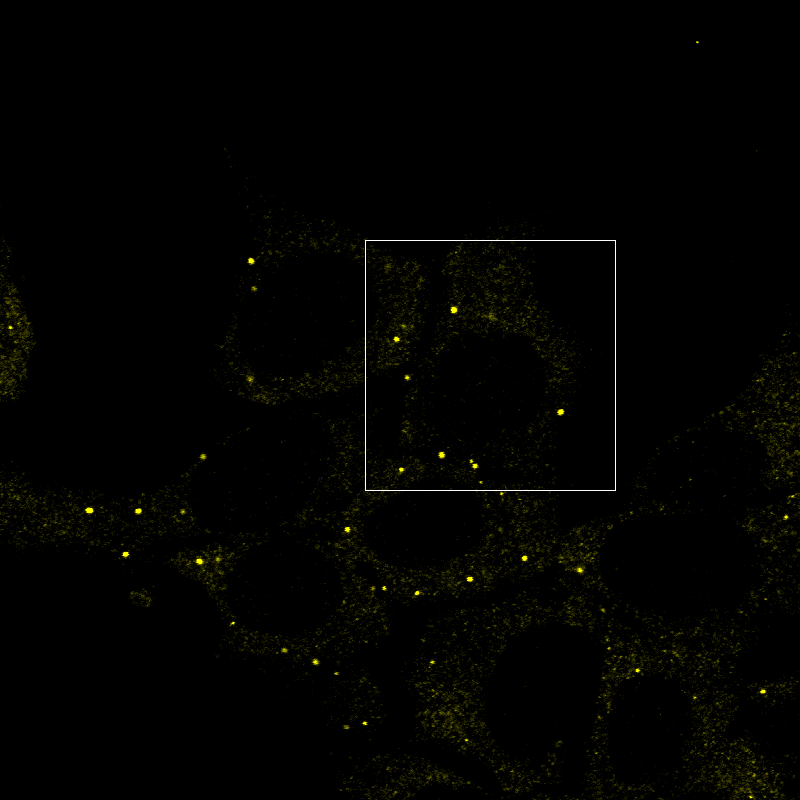

Supplement: Supplementary file 8 — Source data Fig. 5 [file 44318_2024_120_MOESM8_ESM.zip › Figure 5/5A/Oligomycin/WT/LSM14A.tif]

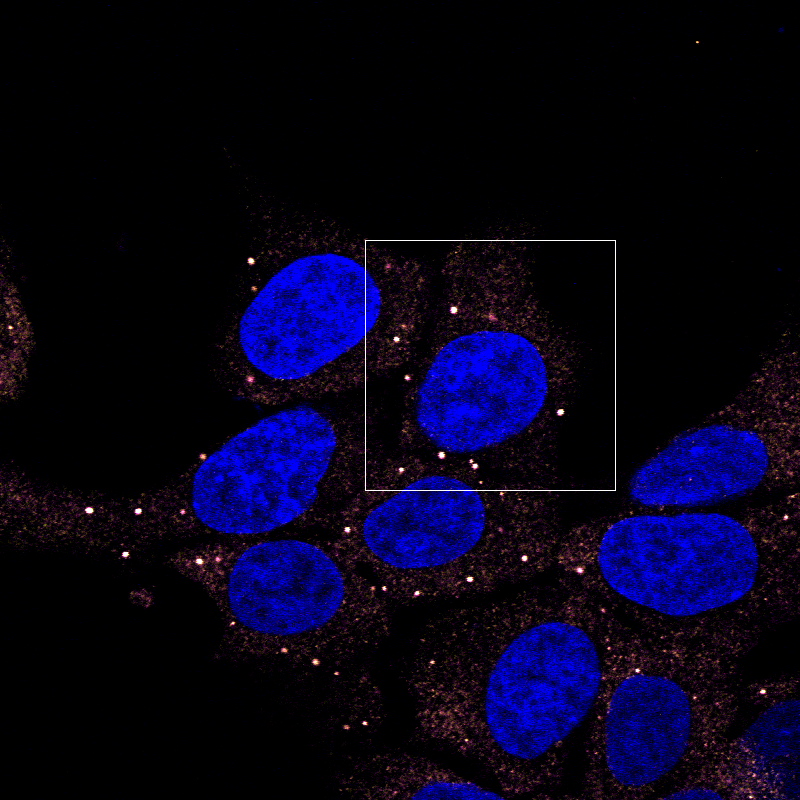

Supplement: Supplementary file 8 — Source data Fig. 5 [file 44318_2024_120_MOESM8_ESM.zip › Figure 5/5A/Oligomycin/WT/Merge.tif]

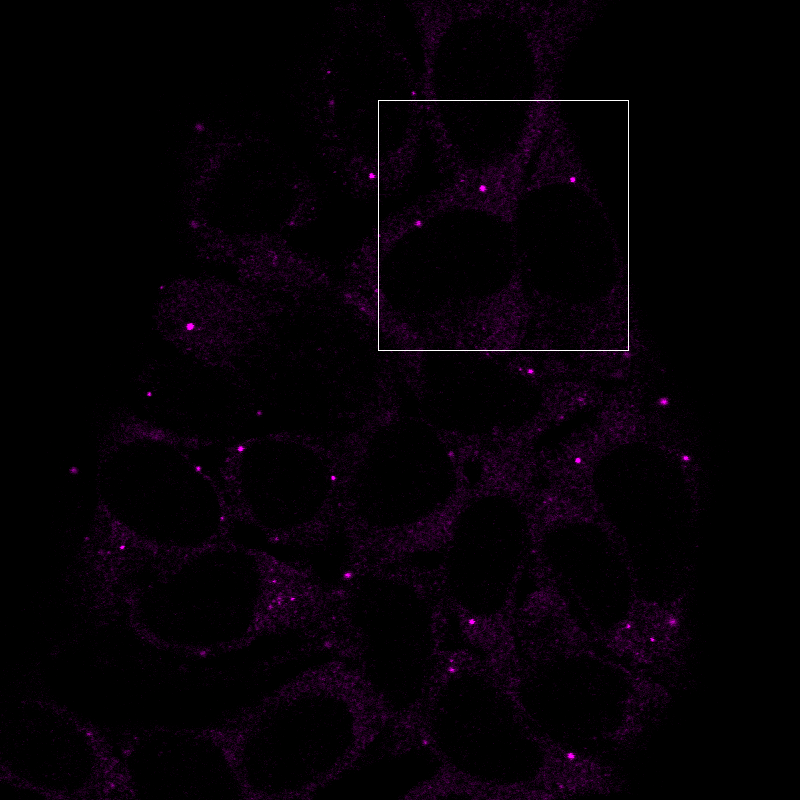

Supplement: Supplementary file 8 — Source data Fig. 5 [file 44318_2024_120_MOESM8_ESM.zip › Figure 5/5C/HAX1-WT/Mock/DCP1A.tif]

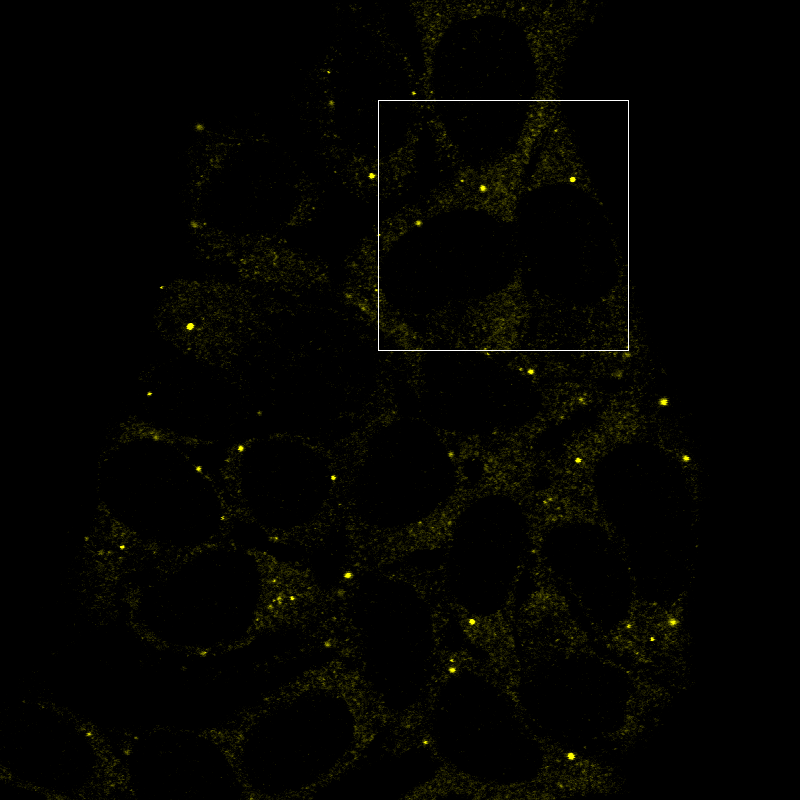

Supplement: Supplementary file 8 — Source data Fig. 5 [file 44318_2024_120_MOESM8_ESM.zip › Figure 5/5C/HAX1-WT/Mock/LSM14A.tif]

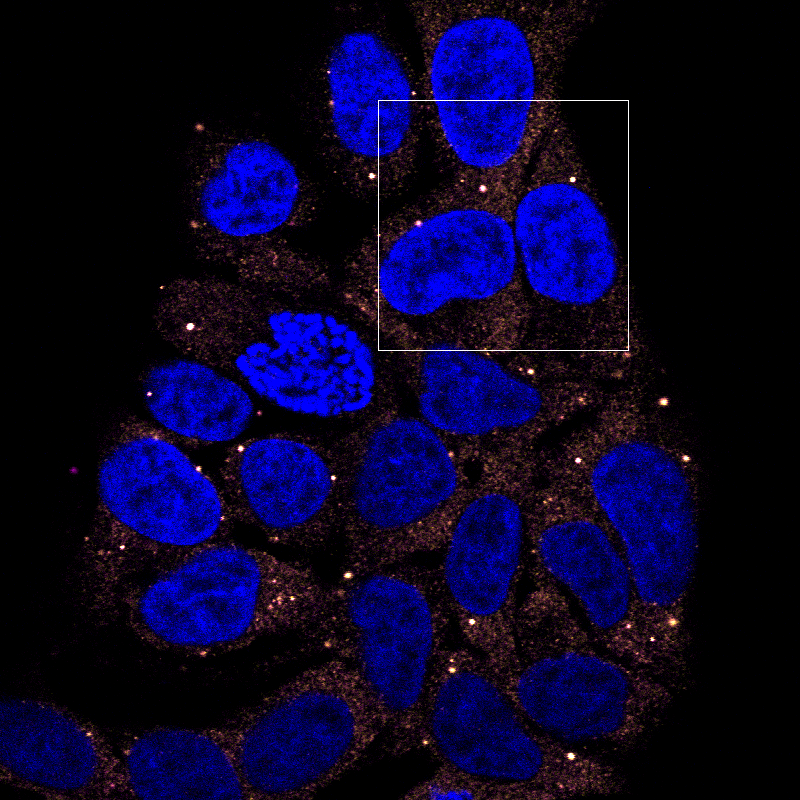

Supplement: Supplementary file 8 — Source data Fig. 5 [file 44318_2024_120_MOESM8_ESM.zip › Figure 5/5C/HAX1-WT/Mock/Merge.tif]

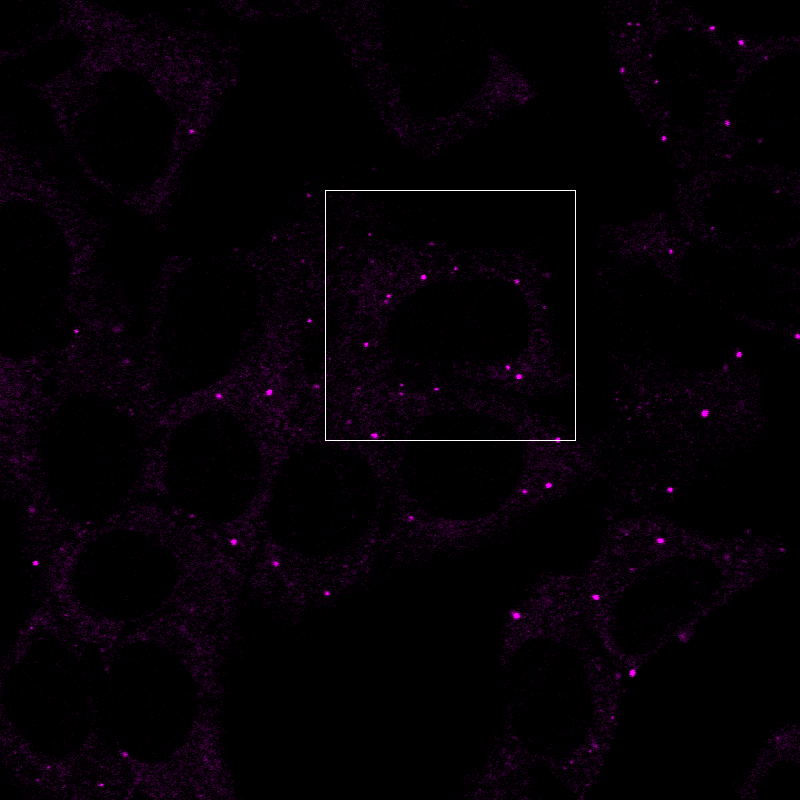

Supplement: Supplementary file 8 — Source data Fig. 5 [file 44318_2024_120_MOESM8_ESM.zip › Figure 5/5C/HAX1-WT/Oligomycin/DCP1A.tif]

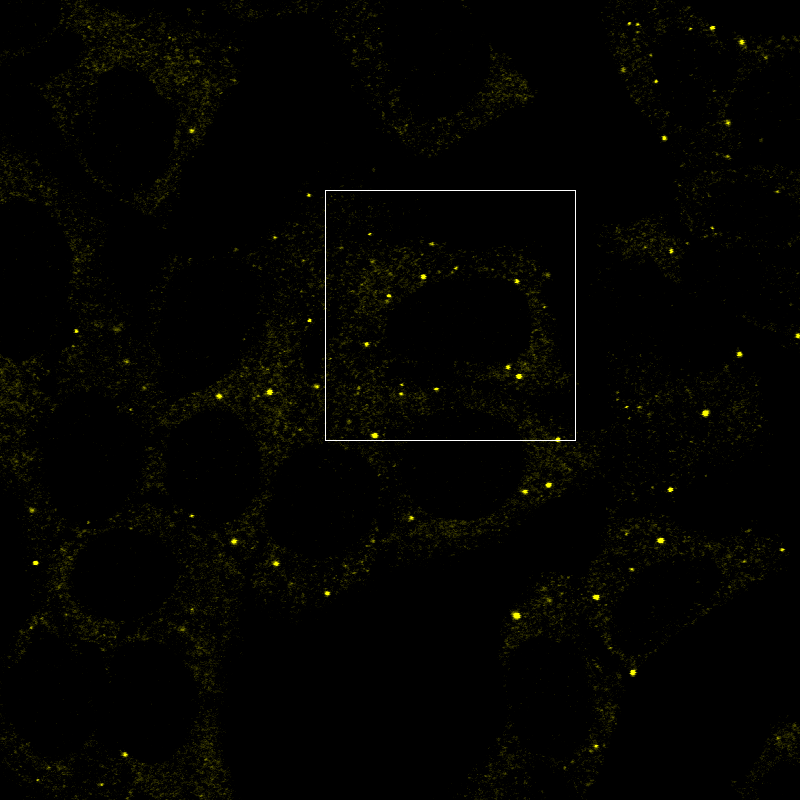

Supplement: Supplementary file 8 — Source data Fig. 5 [file 44318_2024_120_MOESM8_ESM.zip › Figure 5/5C/HAX1-WT/Oligomycin/LSM14A.tif]

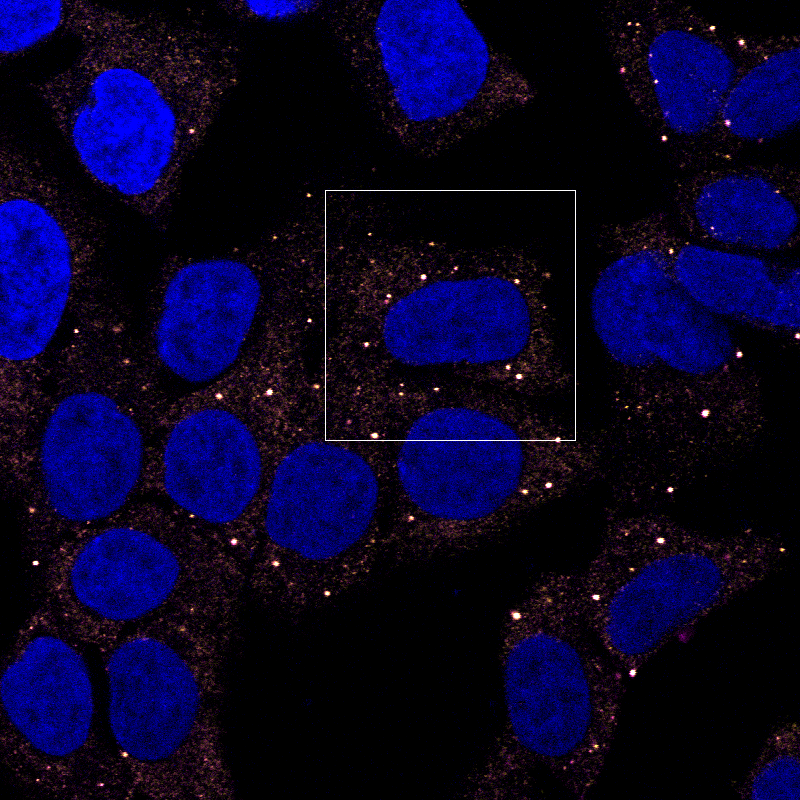

Supplement: Supplementary file 8 — Source data Fig. 5 [file 44318_2024_120_MOESM8_ESM.zip › Figure 5/5C/HAX1-WT/Oligomycin/Merge.tif]

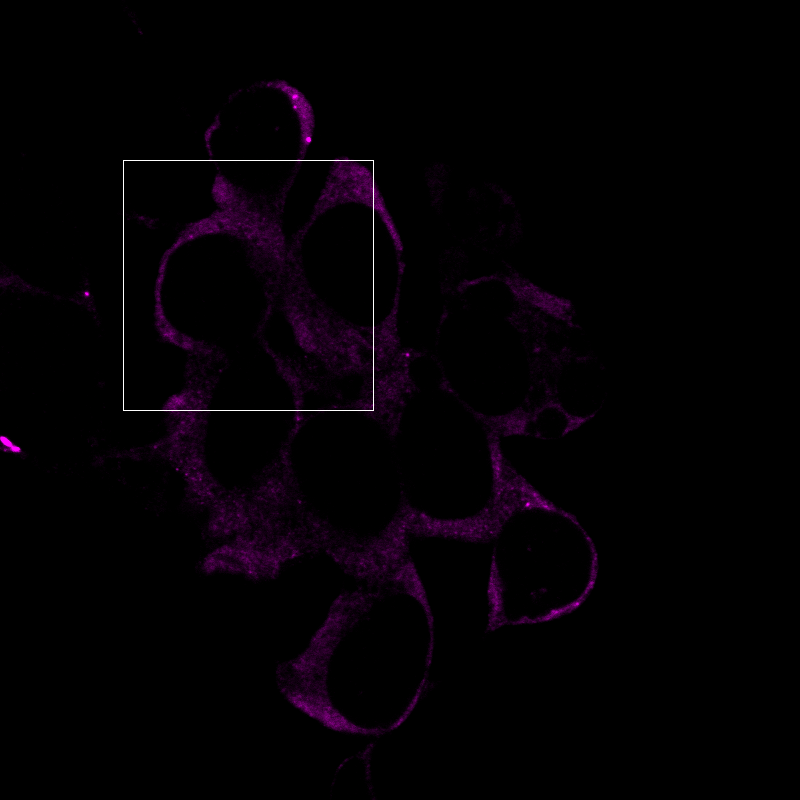

Supplement: Supplementary file 8 — Source data Fig. 5 [file 44318_2024_120_MOESM8_ESM.zip › Figure 5/5C/HAX1-K131R/Mock/DCP1A.tif]

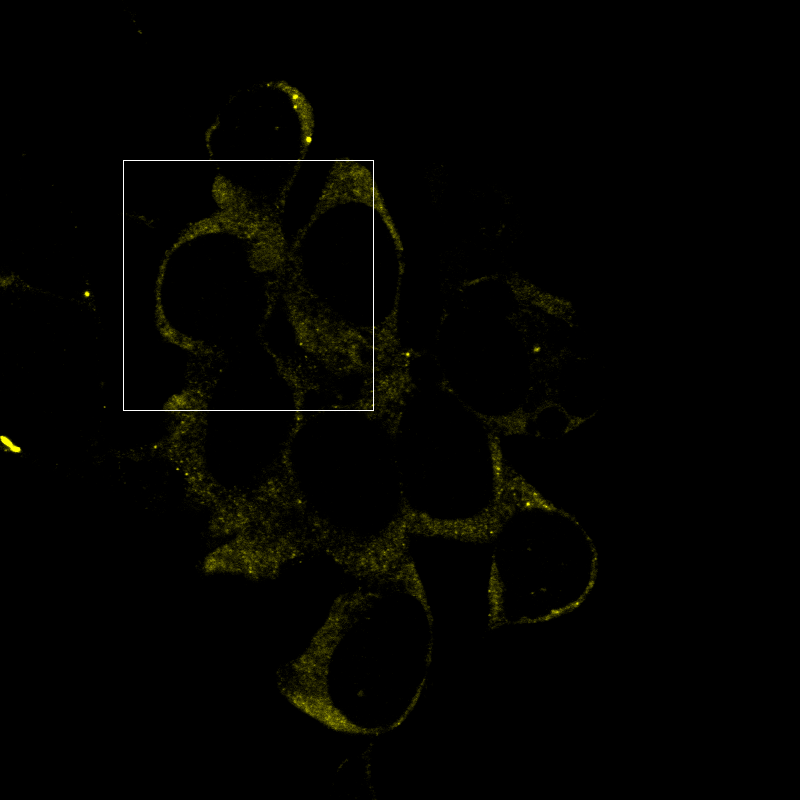

Supplement: Supplementary file 8 — Source data Fig. 5 [file 44318_2024_120_MOESM8_ESM.zip › Figure 5/5C/HAX1-K131R/Mock/LSM14A.tif]

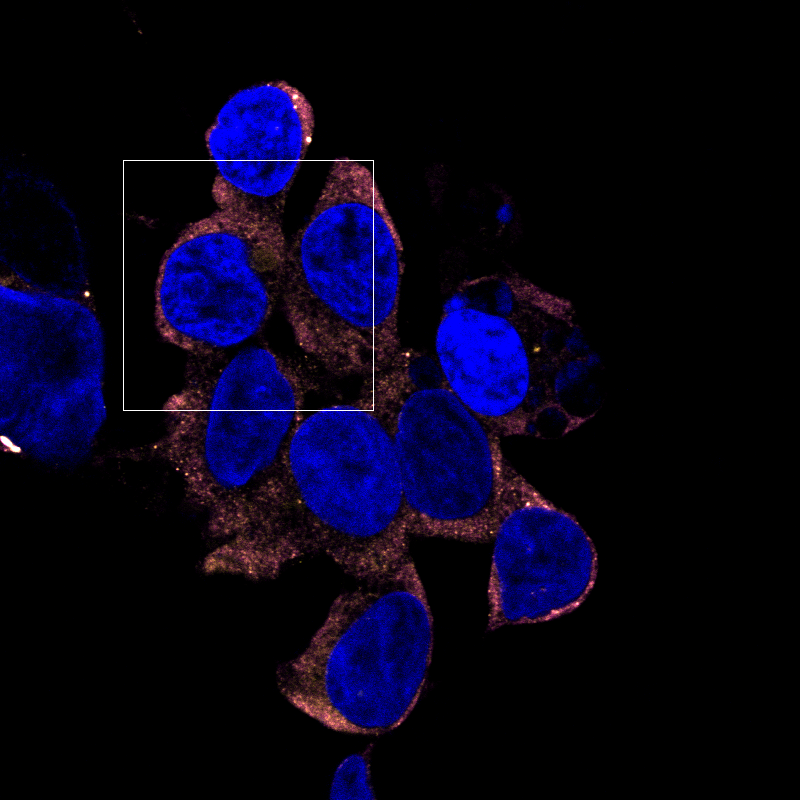

Supplement: Supplementary file 8 — Source data Fig. 5 [file 44318_2024_120_MOESM8_ESM.zip › Figure 5/5C/HAX1-K131R/Mock/Merge.tif]

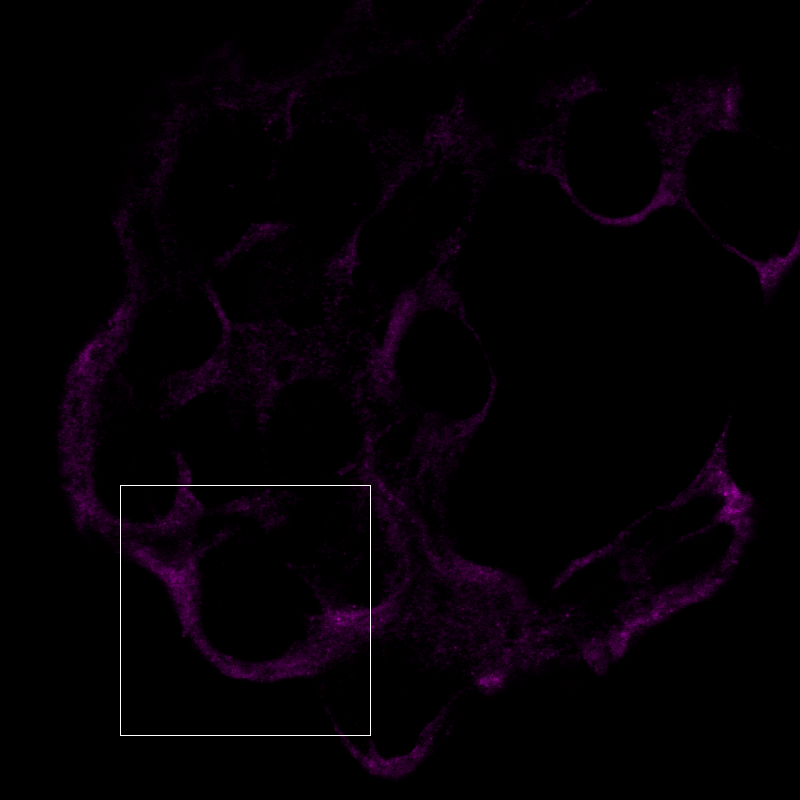

Supplement: Supplementary file 8 — Source data Fig. 5 [file 44318_2024_120_MOESM8_ESM.zip › Figure 5/5C/HAX1-K131R/Oligomycin/DCP1A.tif]

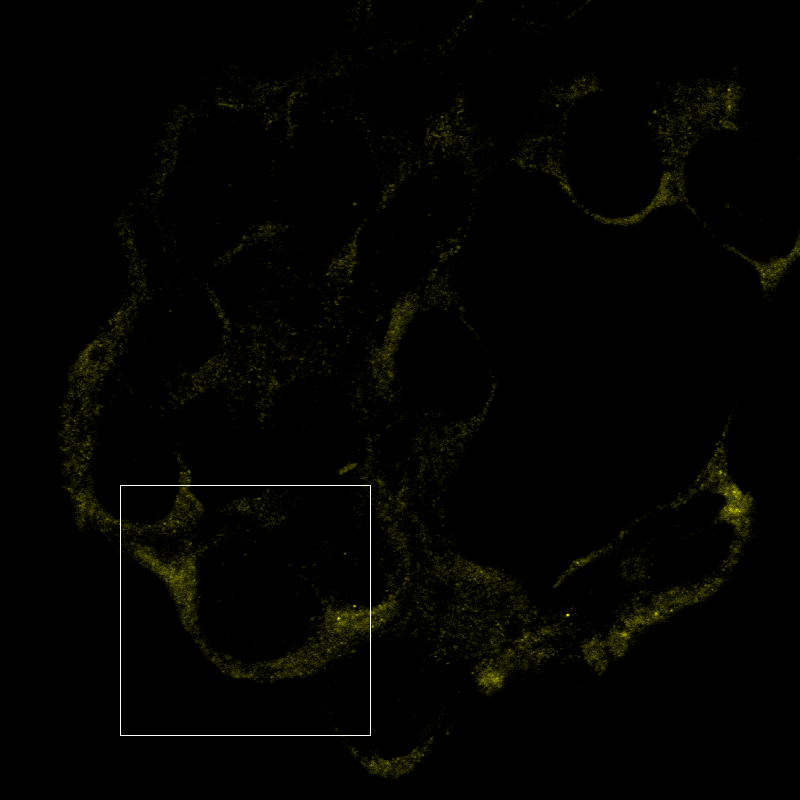

Supplement: Supplementary file 8 — Source data Fig. 5 [file 44318_2024_120_MOESM8_ESM.zip › Figure 5/5C/HAX1-K131R/Oligomycin/LSM14A.tif]

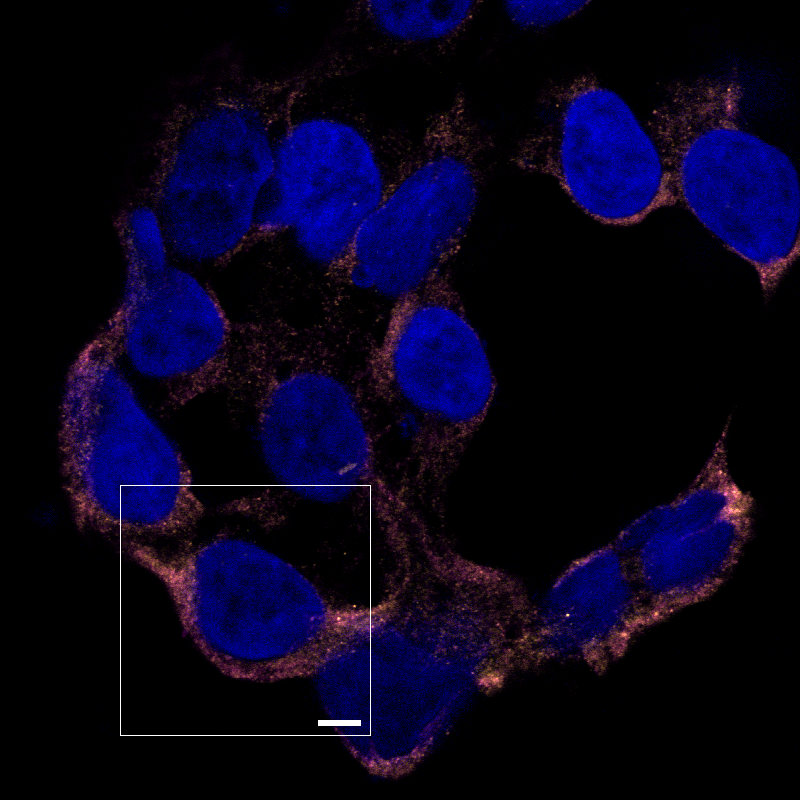

Supplement: Supplementary file 8 — Source data Fig. 5 [file 44318_2024_120_MOESM8_ESM.zip › Figure 5/5C/HAX1-K131R/Oligomycin/Merge.tif]

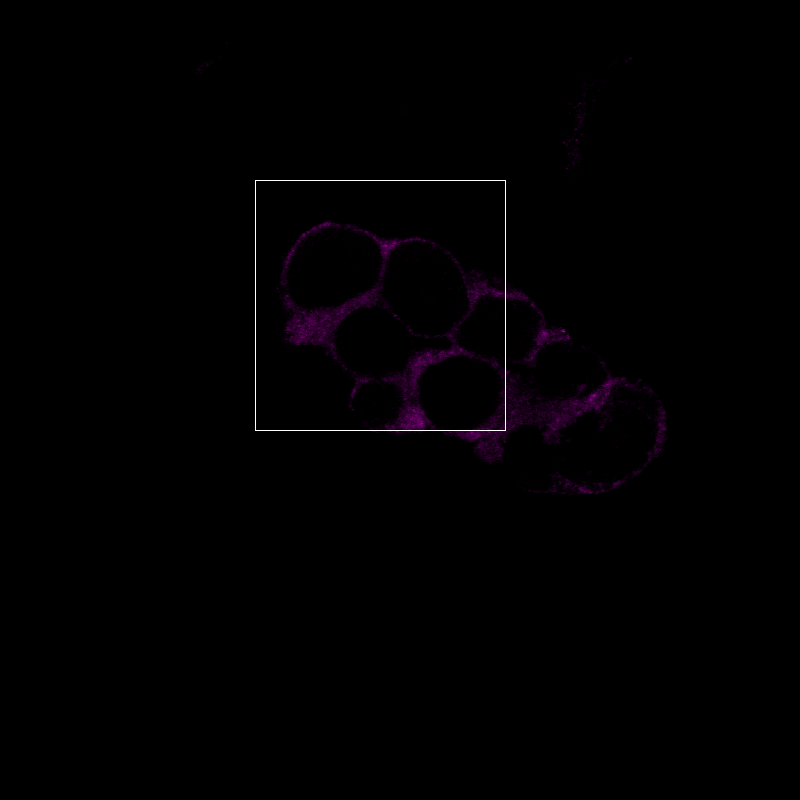

Supplement: Supplementary file 8 — Source data Fig. 5 [file 44318_2024_120_MOESM8_ESM.zip › Figure 5/5C/Vector/Mock/DCP1A.tif]

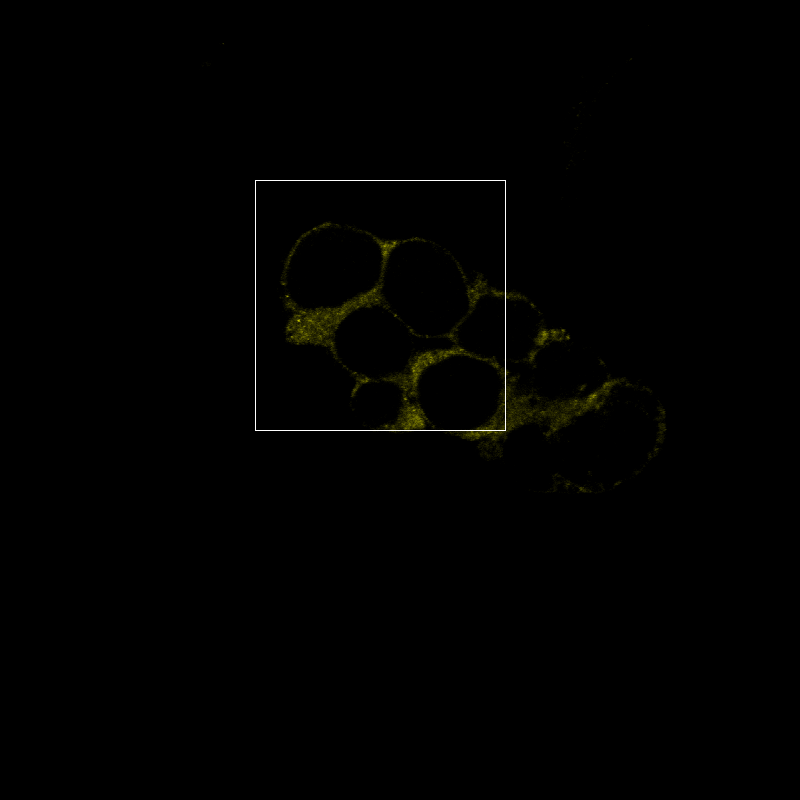

Supplement: Supplementary file 8 — Source data Fig. 5 [file 44318_2024_120_MOESM8_ESM.zip › Figure 5/5C/Vector/Mock/LSM14A.tif]

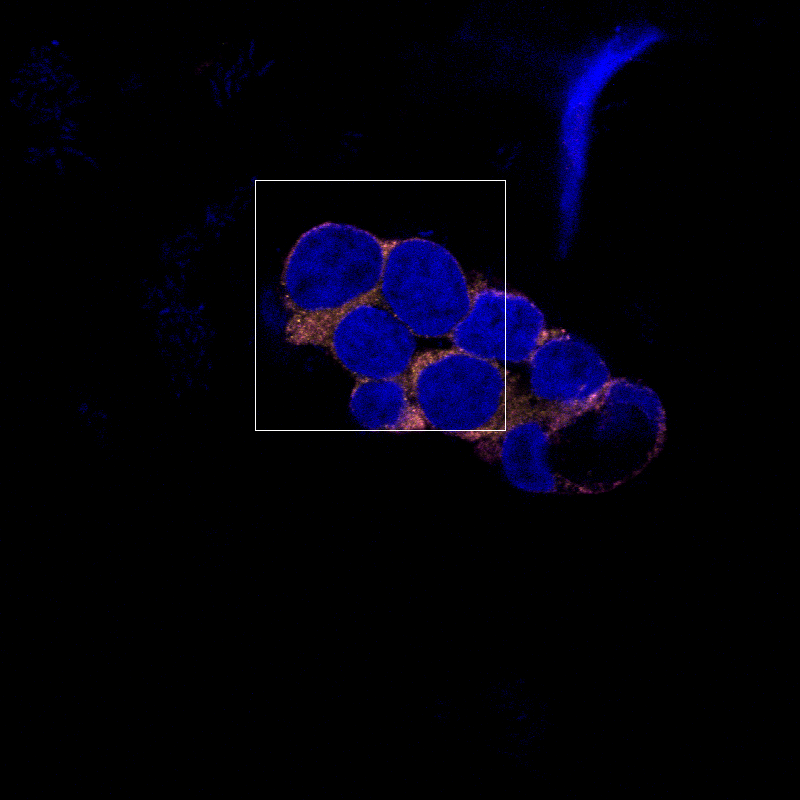

Supplement: Supplementary file 8 — Source data Fig. 5 [file 44318_2024_120_MOESM8_ESM.zip › Figure 5/5C/Vector/Mock/Merge.tif]

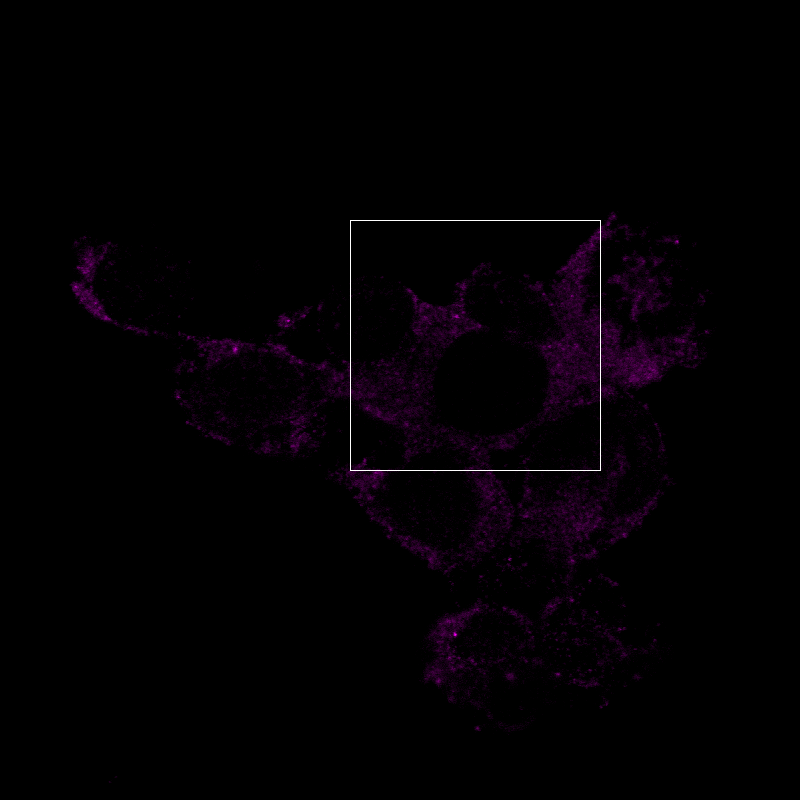

Supplement: Supplementary file 8 — Source data Fig. 5 [file 44318_2024_120_MOESM8_ESM.zip › Figure 5/5C/Vector/Oligomycin/DCP1A.tif]

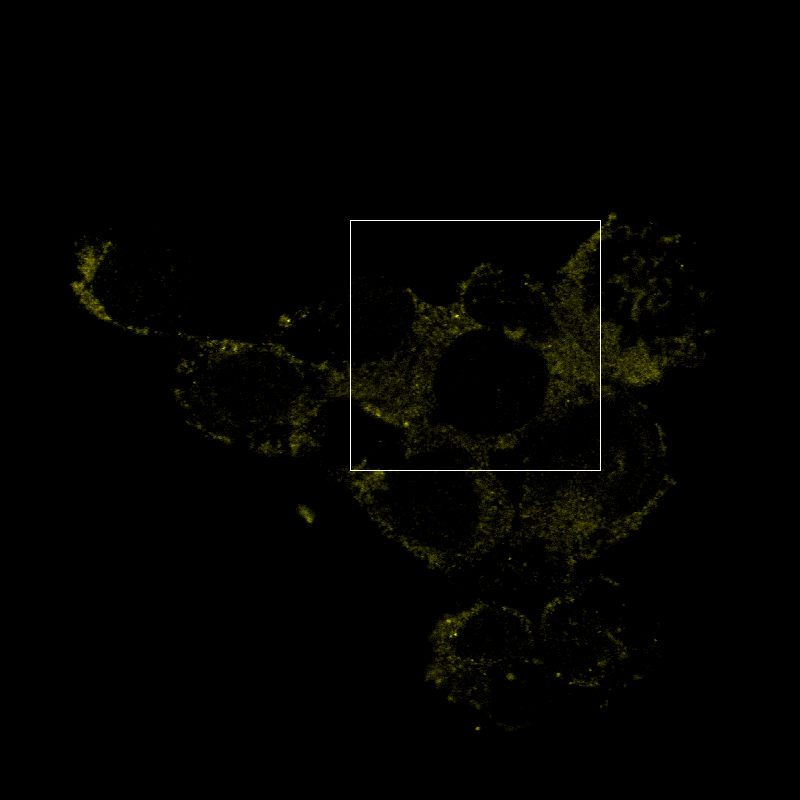

Supplement: Supplementary file 8 — Source data Fig. 5 [file 44318_2024_120_MOESM8_ESM.zip › Figure 5/5C/Vector/Oligomycin/LSM14A.tif]

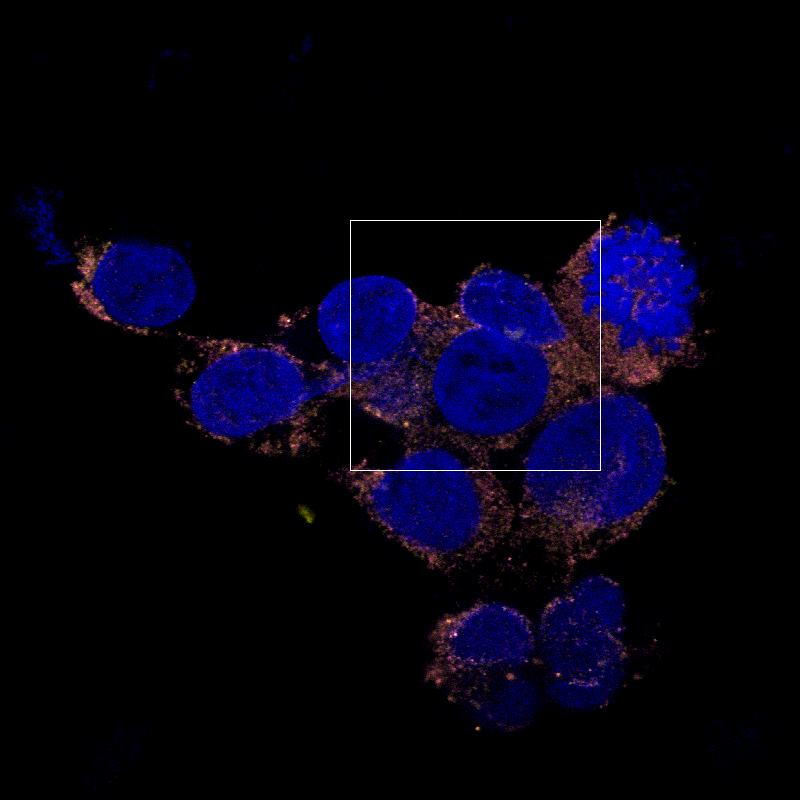

Supplement: Supplementary file 8 — Source data Fig. 5 [file 44318_2024_120_MOESM8_ESM.zip › Figure 5/5C/Vector/Oligomycin/Merge.tif]

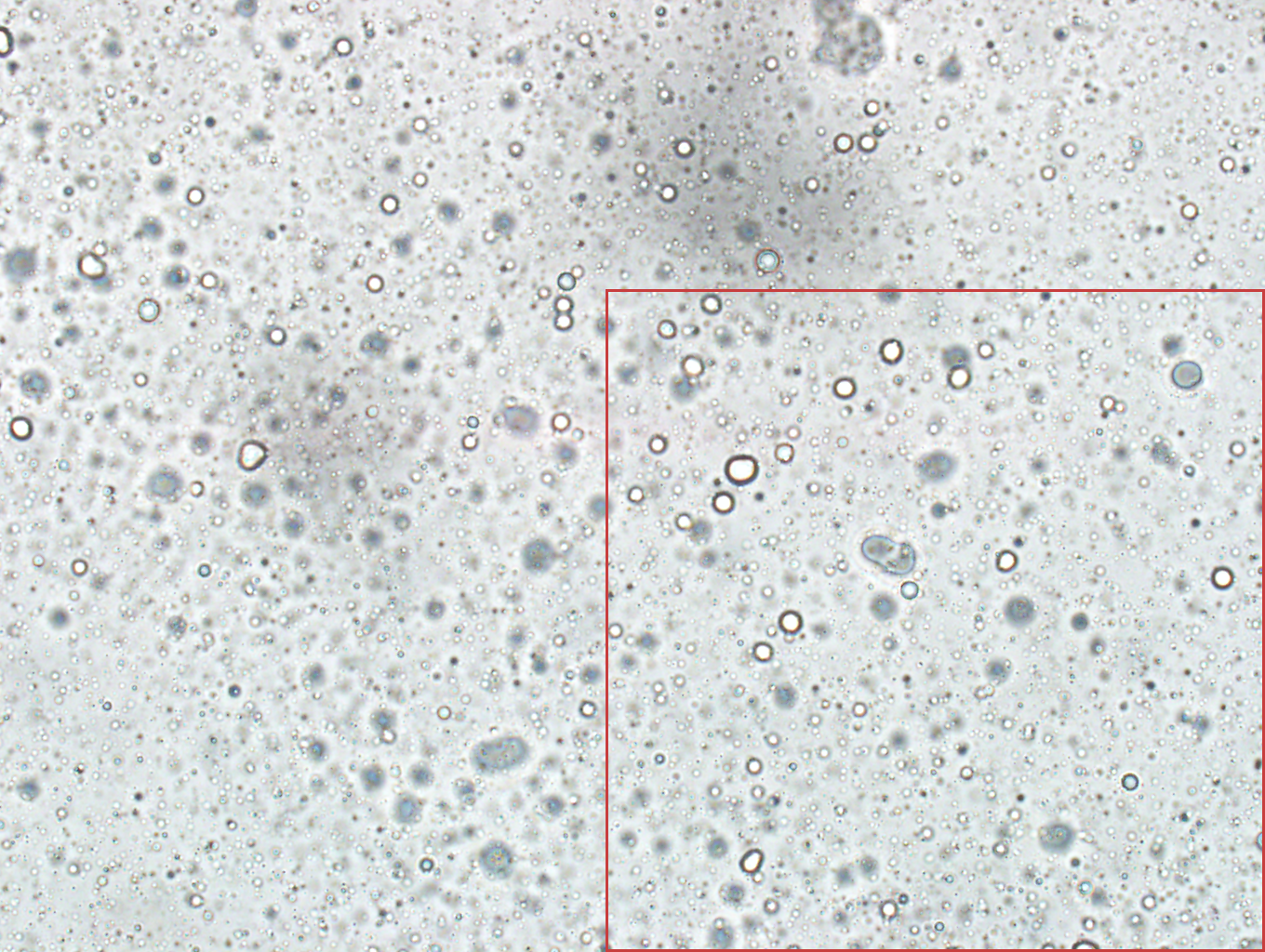

Supplement: Supplementary file 9 — Source data Fig. 6 [file 44318_2024_120_MOESM9_ESM.zip › Figure 6/6B/10% PEG 20uM.tif]

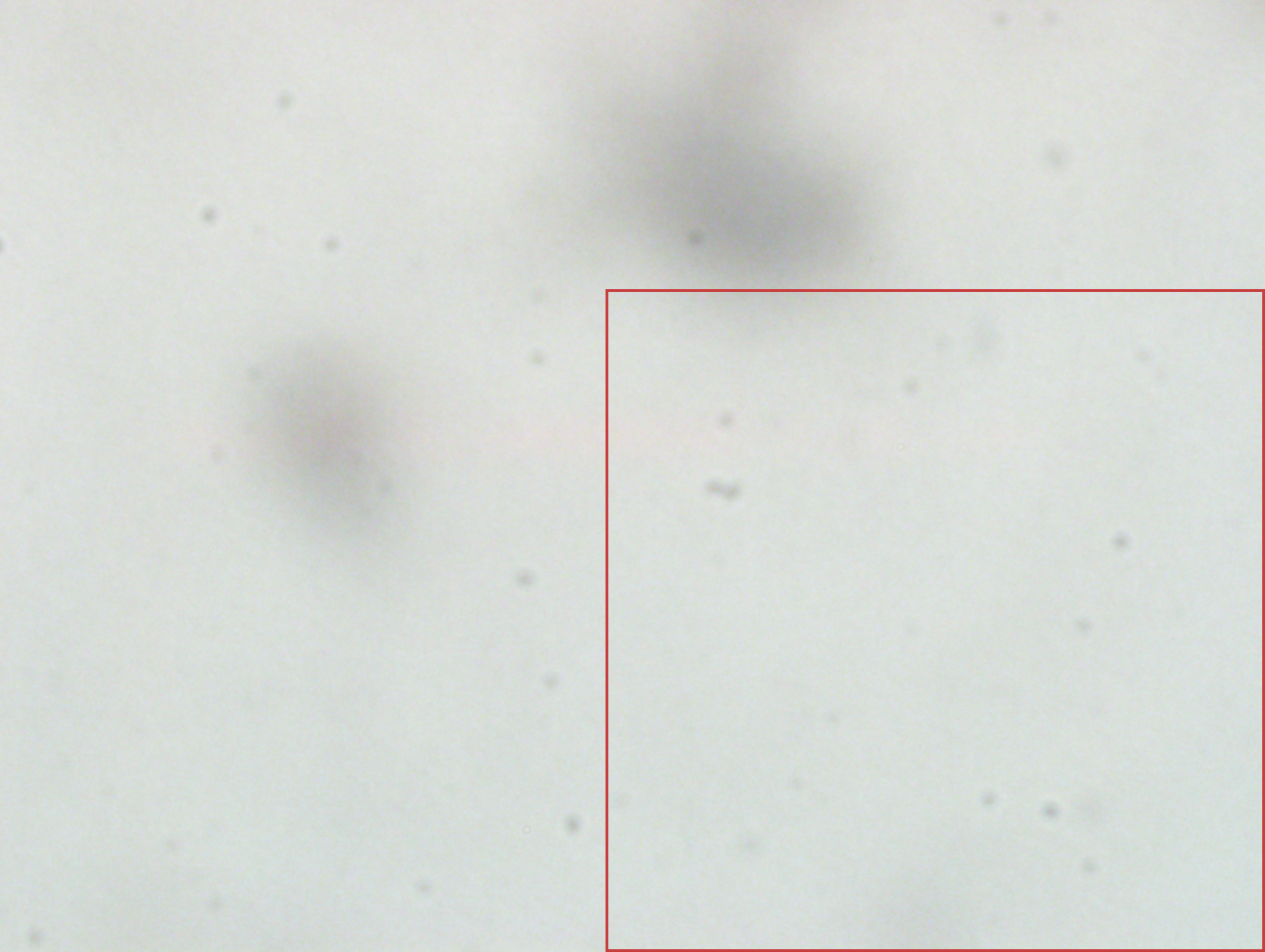

Supplement: Supplementary file 9 — Source data Fig. 6 [file 44318_2024_120_MOESM9_ESM.zip › Figure 6/6B/5% PEG 20uM.tif]

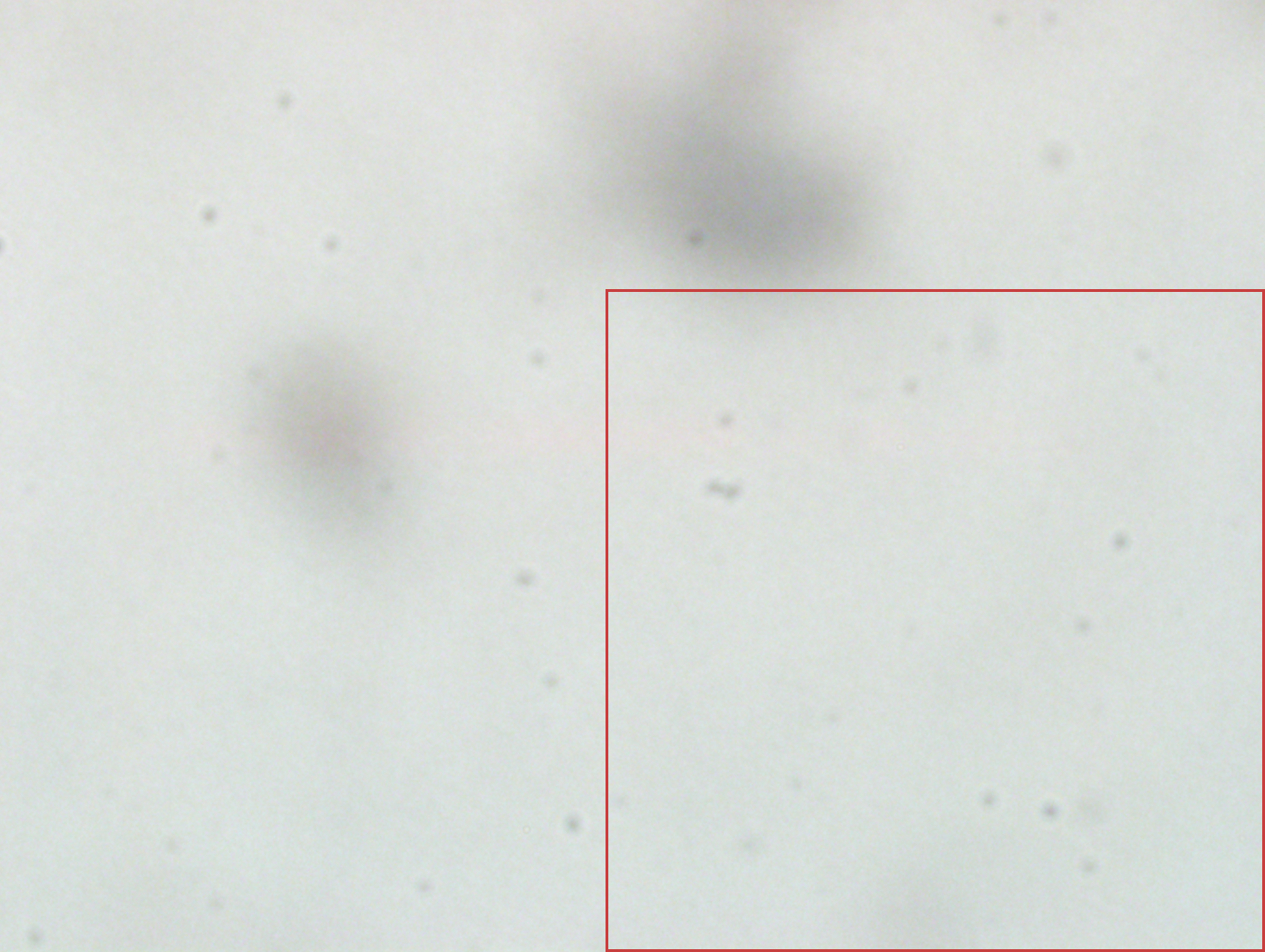

Supplement: Supplementary file 9 — Source data Fig. 6 [file 44318_2024_120_MOESM9_ESM.zip › Figure 6/6B/10% PEG 2.5uM.tif]

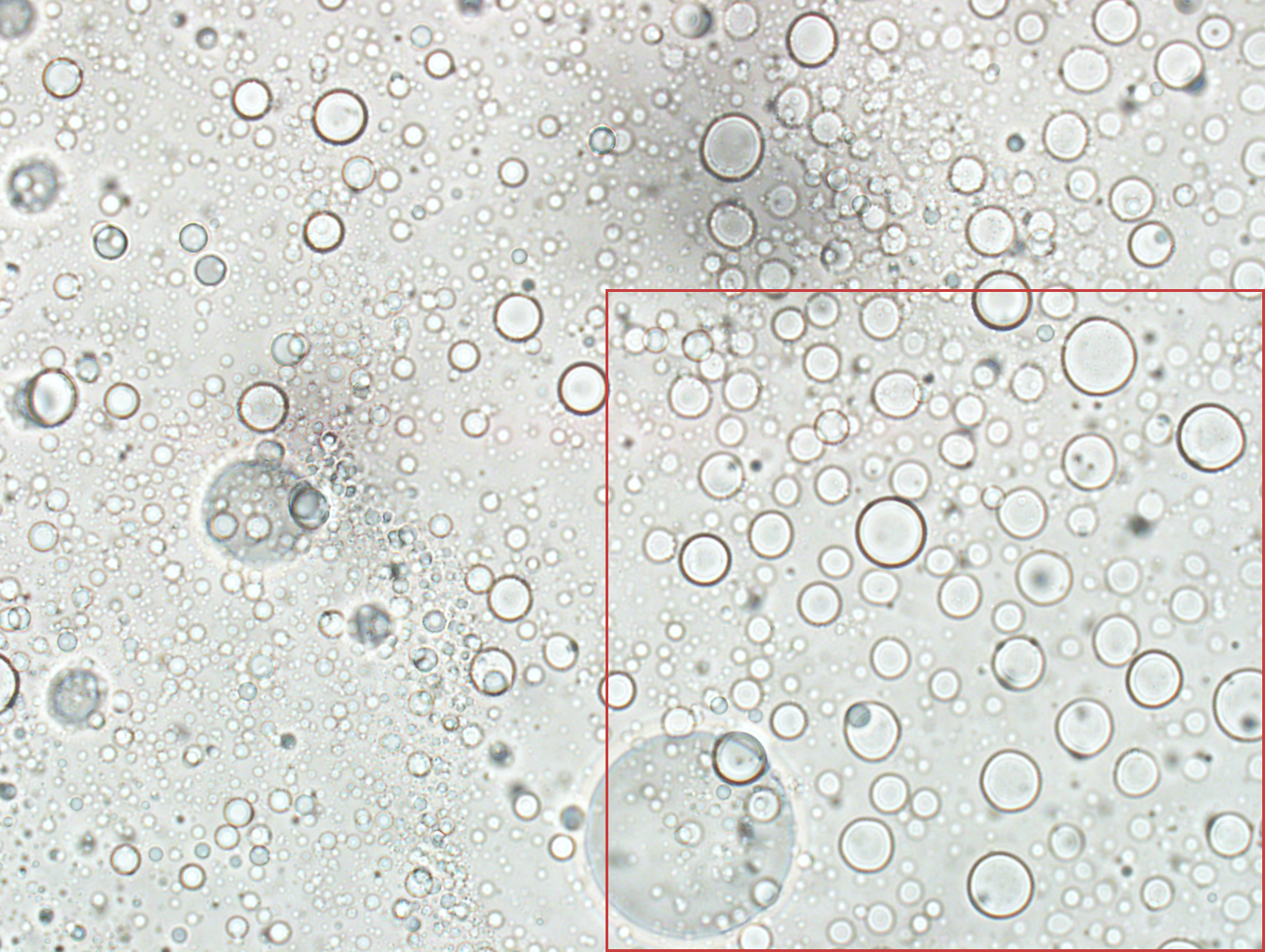

Supplement: Supplementary file 9 — Source data Fig. 6 [file 44318_2024_120_MOESM9_ESM.zip › Figure 6/6B/20% PEG 20uM.tif]

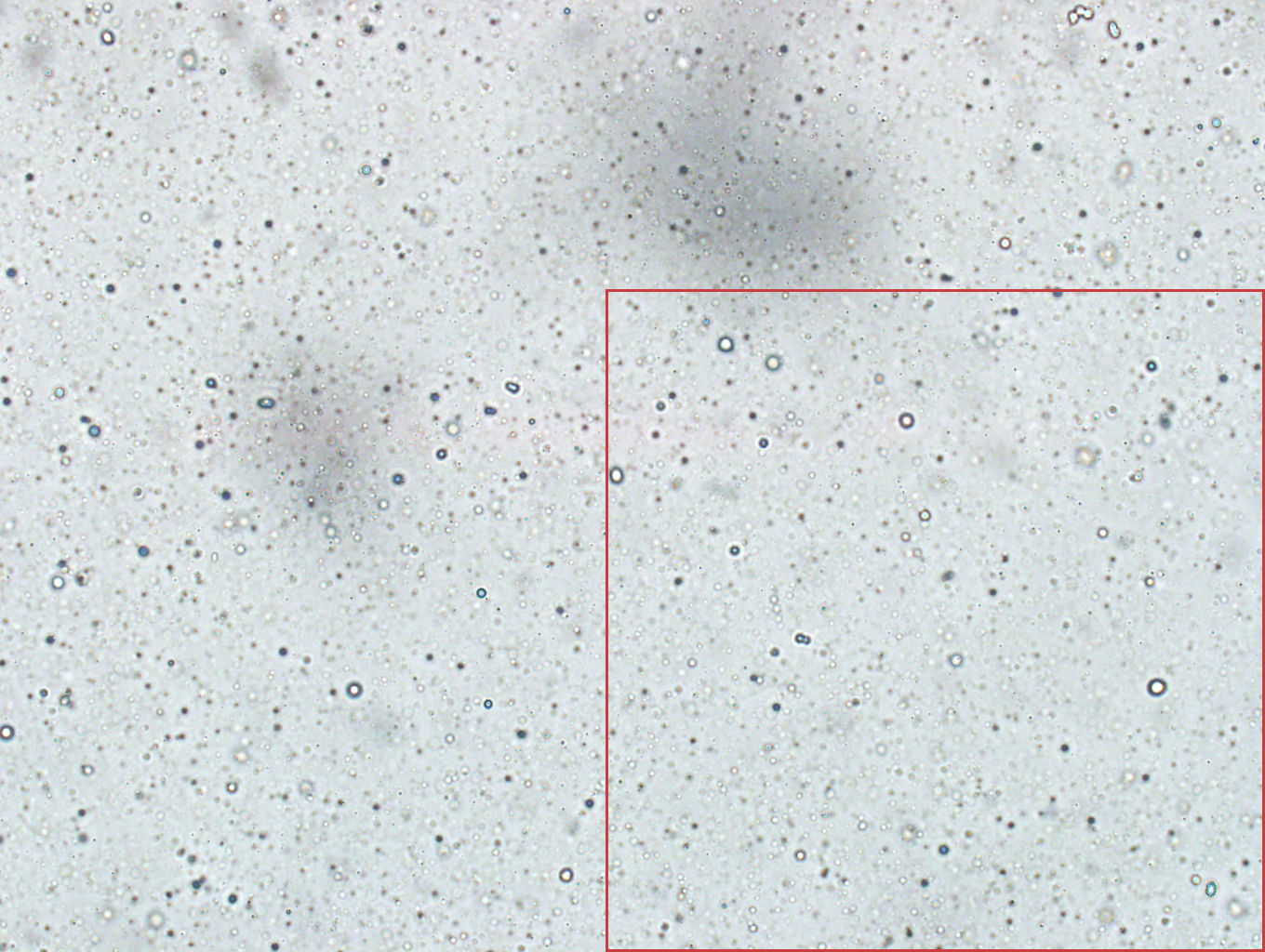

Supplement: Supplementary file 9 — Source data Fig. 6 [file 44318_2024_120_MOESM9_ESM.zip › Figure 6/6B/20% PEG 5uM.tif]

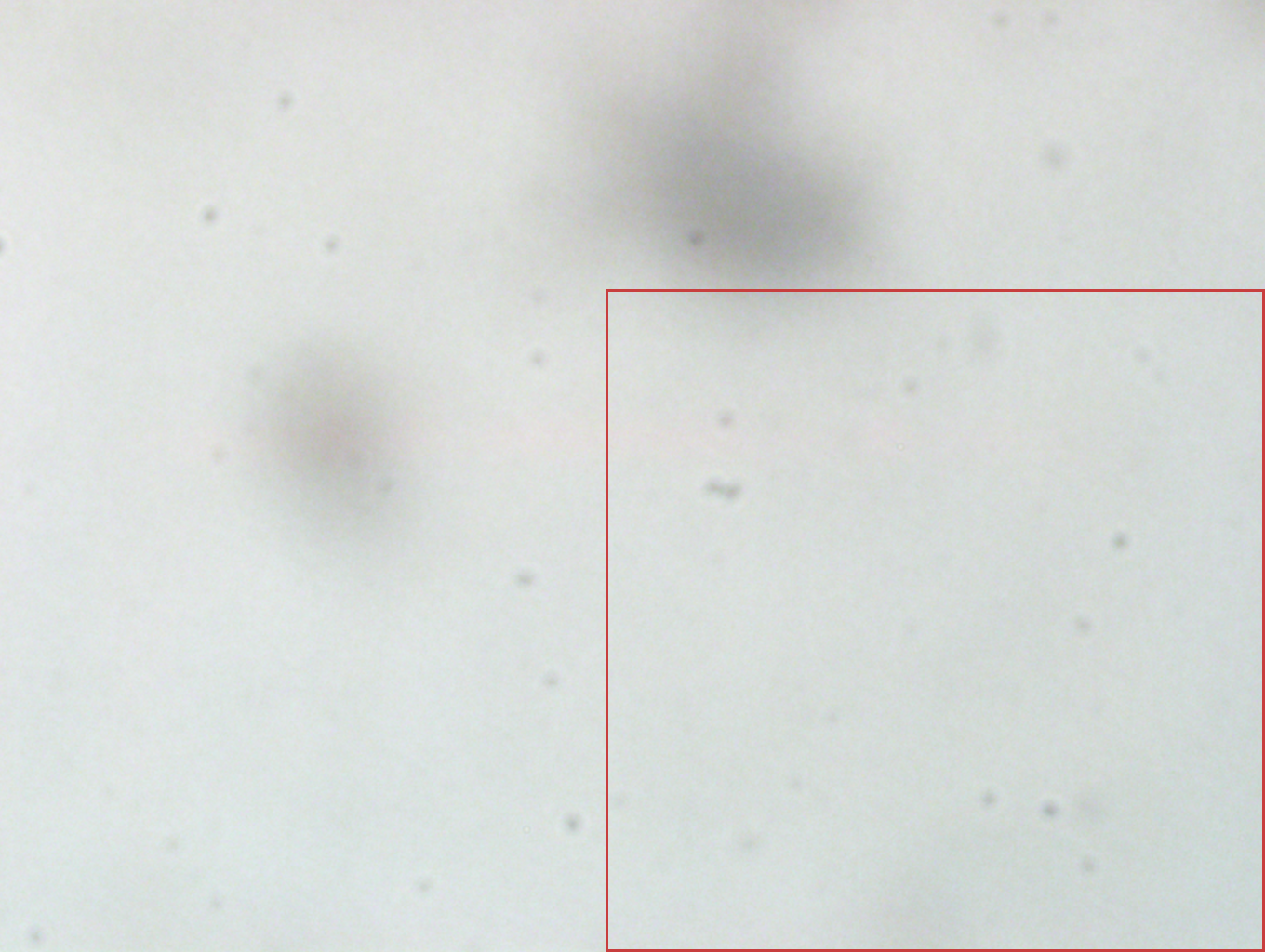

Supplement: Supplementary file 9 — Source data Fig. 6 [file 44318_2024_120_MOESM9_ESM.zip › Figure 6/6B/5% PEG 5uM.tif]

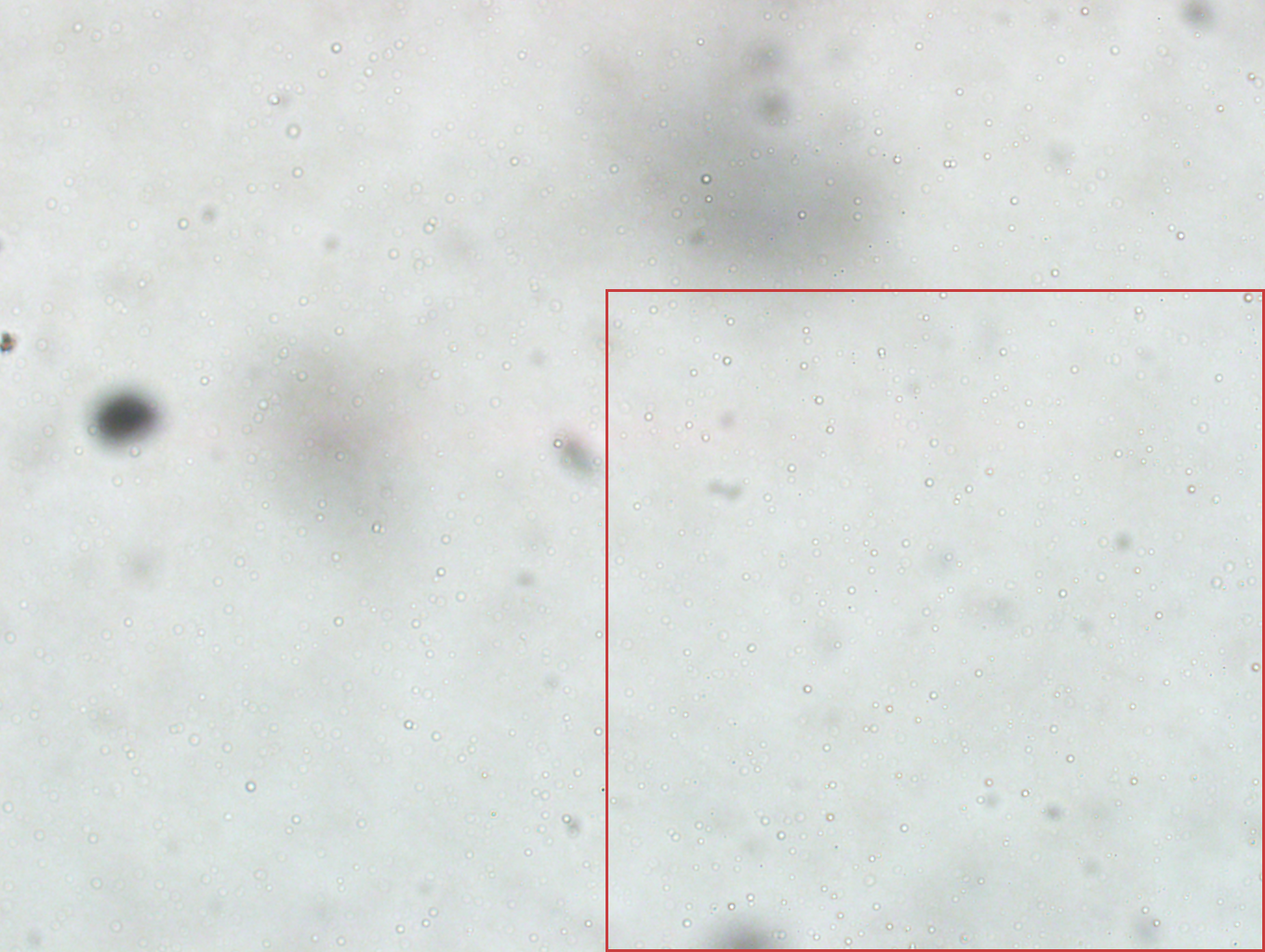

Supplement: Supplementary file 9 — Source data Fig. 6 [file 44318_2024_120_MOESM9_ESM.zip › Figure 6/6B/10% PEG 5uM.tif]

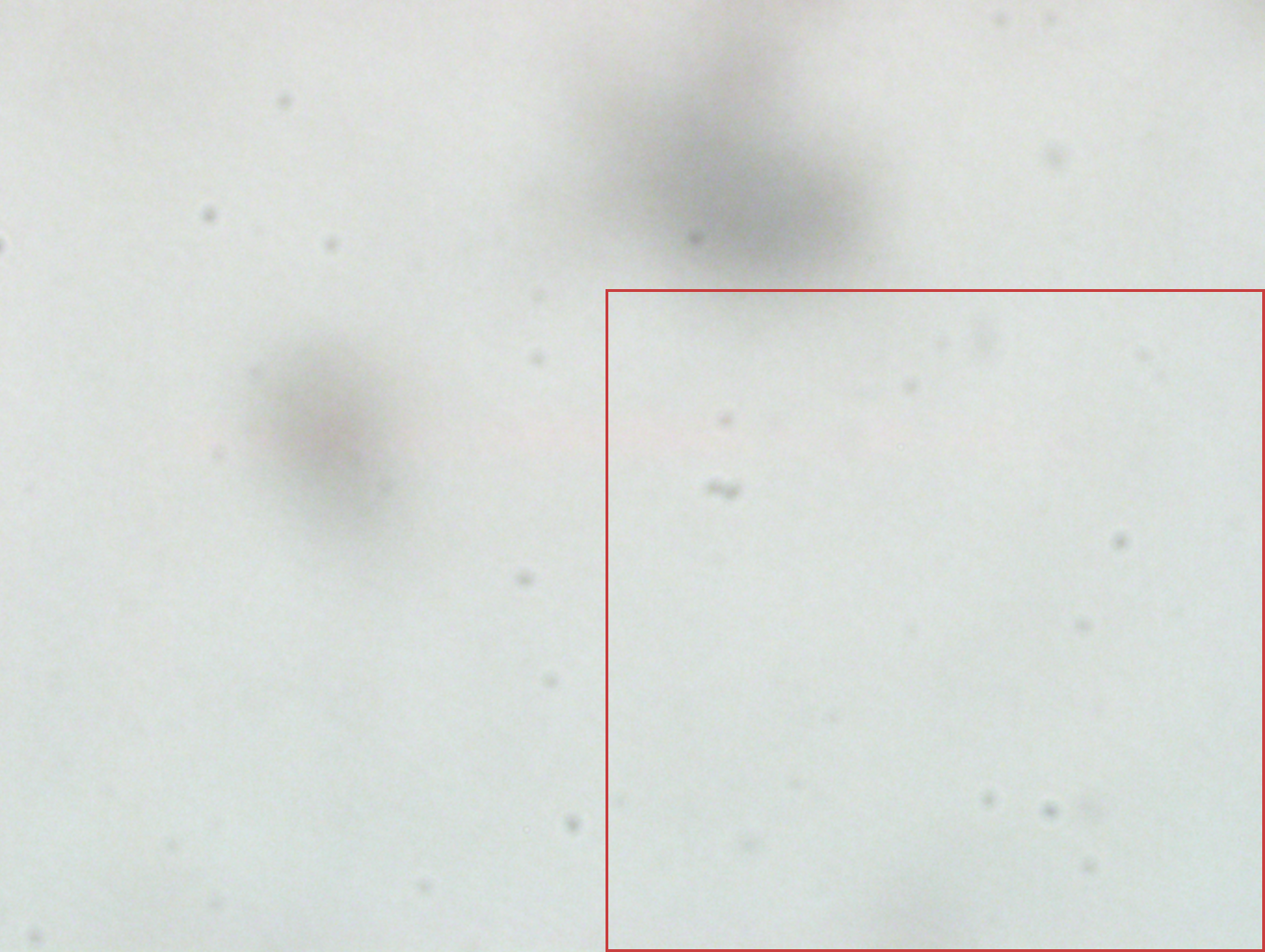

Supplement: Supplementary file 9 — Source data Fig. 6 [file 44318_2024_120_MOESM9_ESM.zip › Figure 6/6B/5% PEG 10uM.tif]

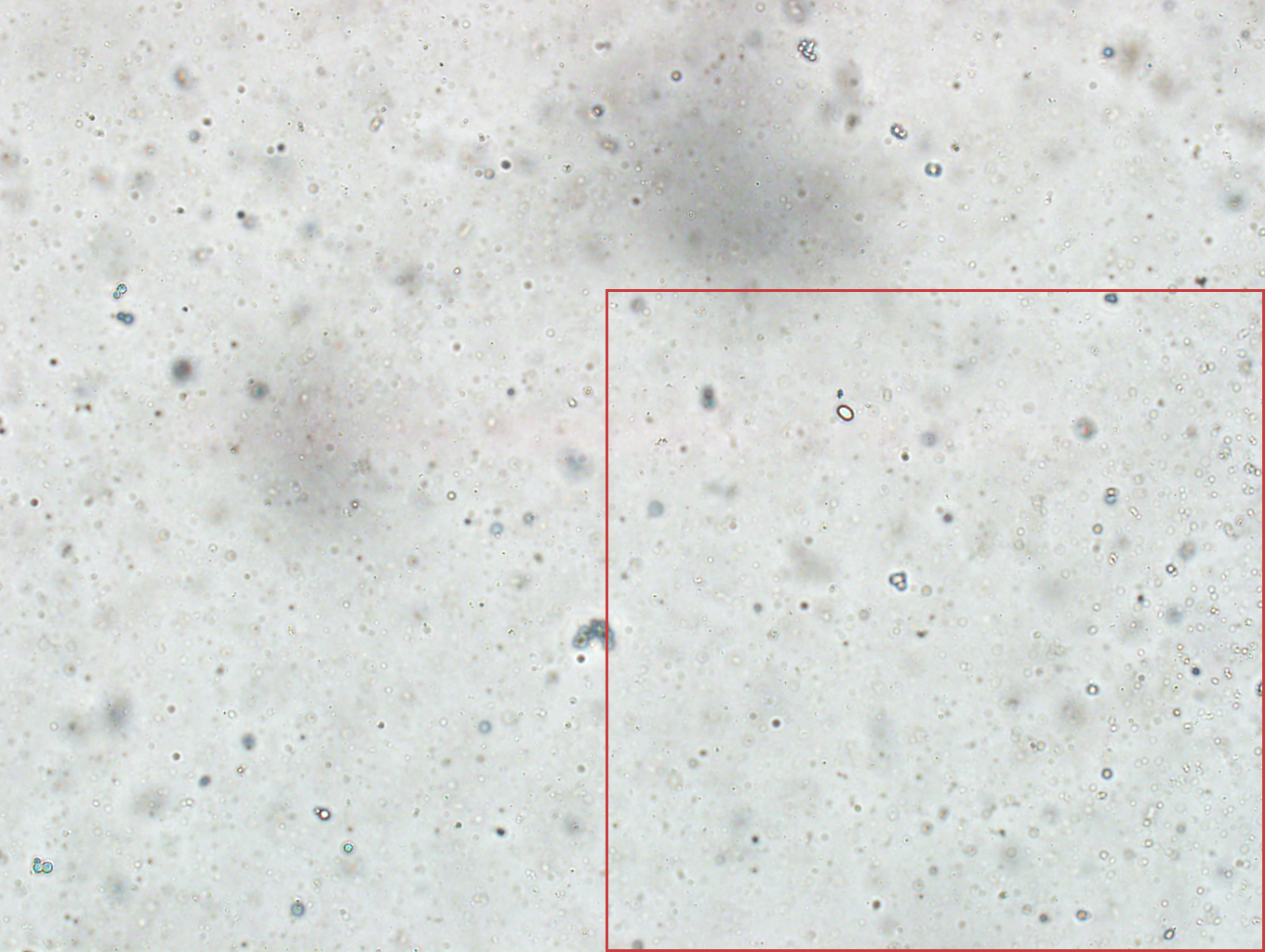

Supplement: Supplementary file 9 — Source data Fig. 6 [file 44318_2024_120_MOESM9_ESM.zip › Figure 6/6B/20% PEG 2.5uM.tif]

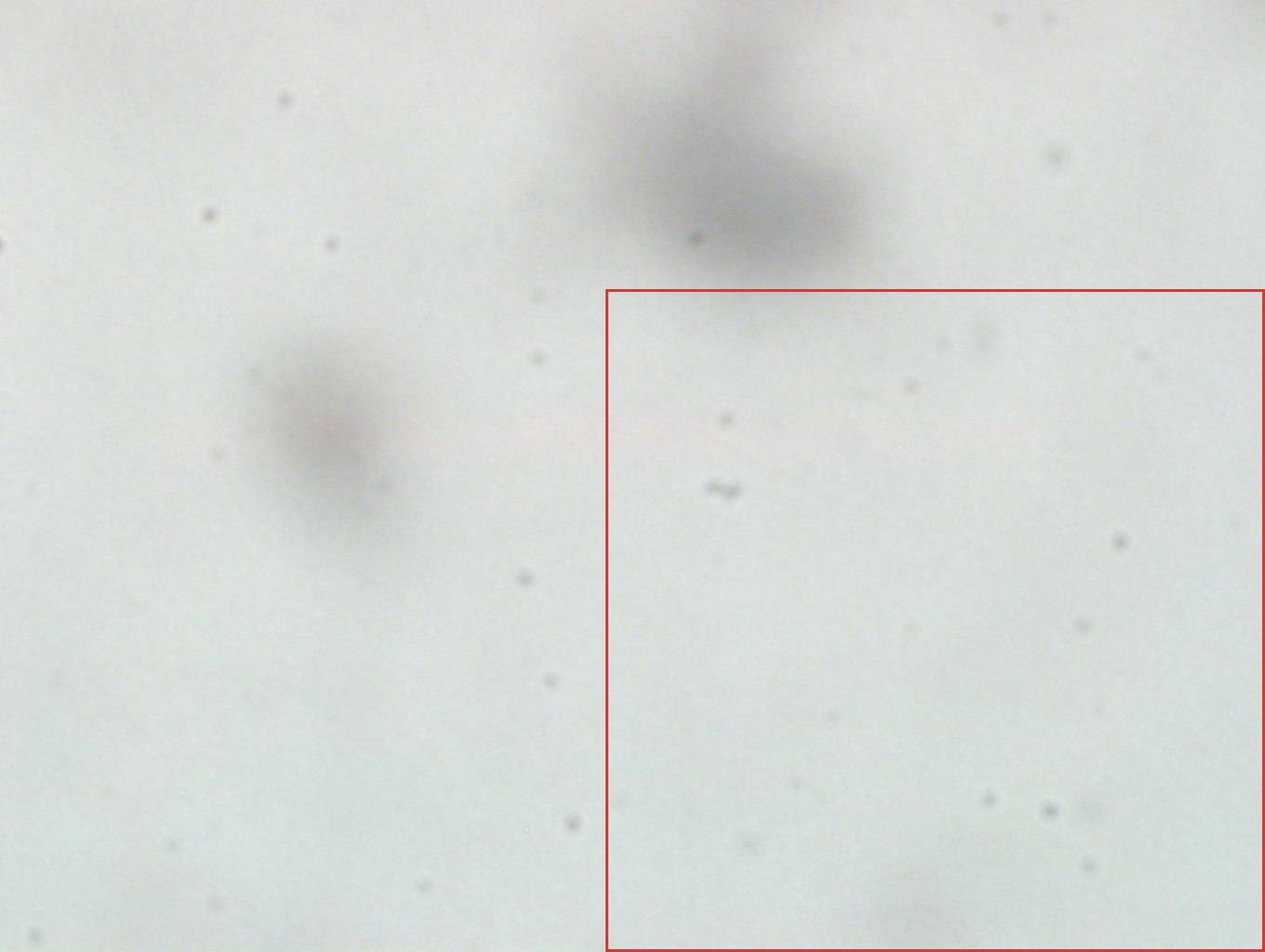

Supplement: Supplementary file 9 — Source data Fig. 6 [file 44318_2024_120_MOESM9_ESM.zip › Figure 6/6B/5% PEG 2.5uM.tif]

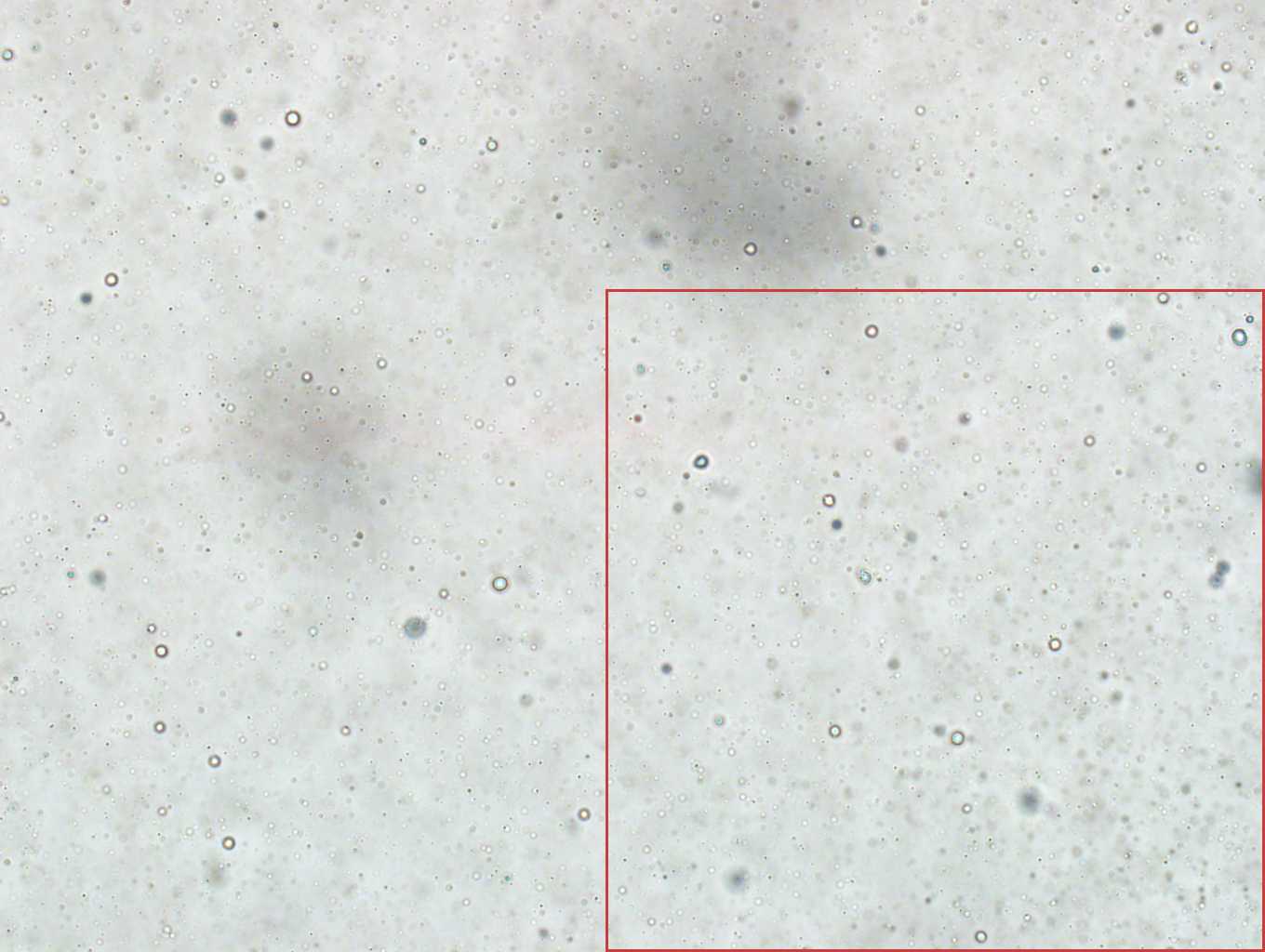

Supplement: Supplementary file 9 — Source data Fig. 6 [file 44318_2024_120_MOESM9_ESM.zip › Figure 6/6B/10% PEG 10uM.tif]

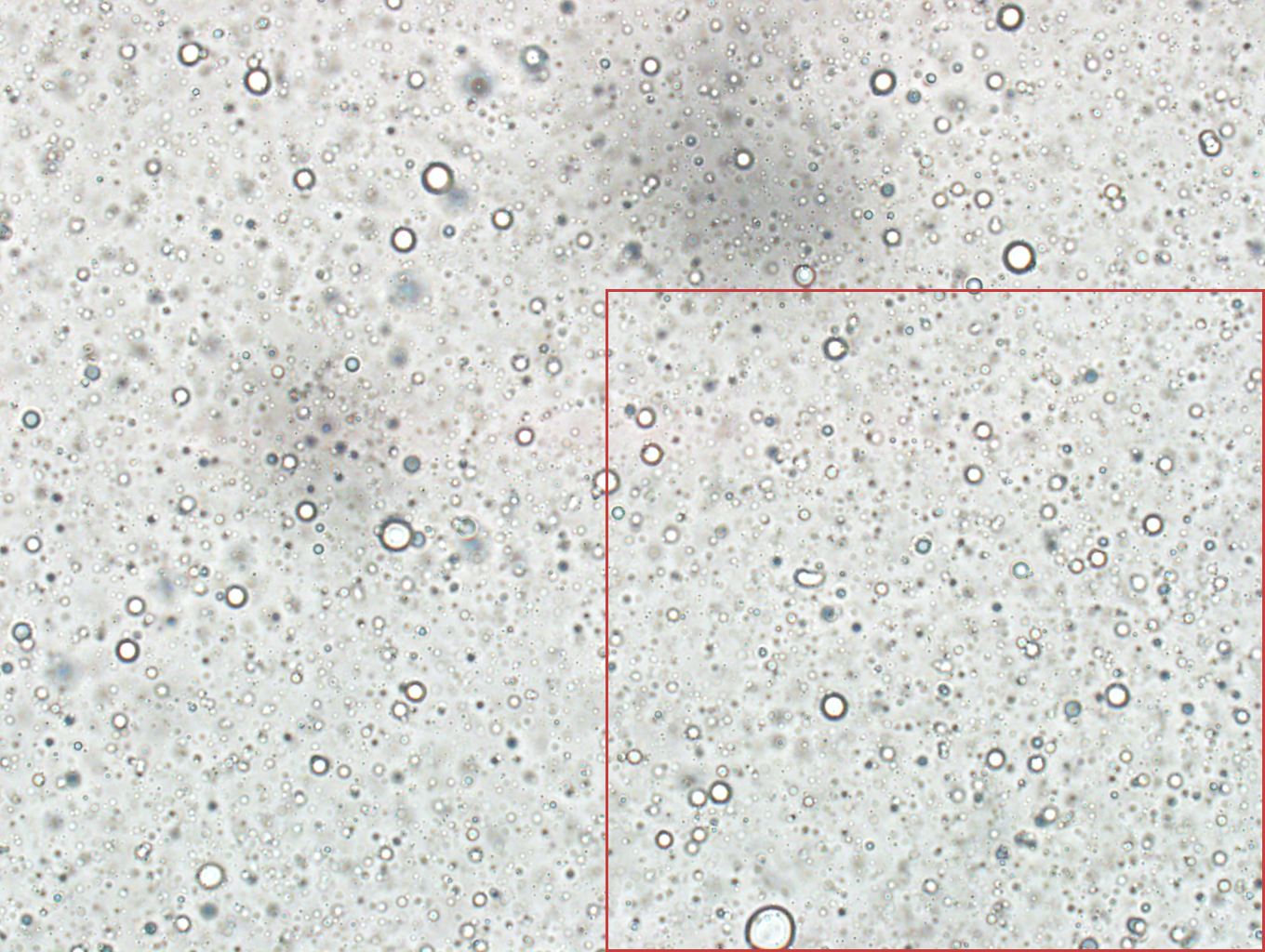

Supplement: Supplementary file 9 — Source data Fig. 6 [file 44318_2024_120_MOESM9_ESM.zip › Figure 6/6B/20% PEG 10uM.tif]

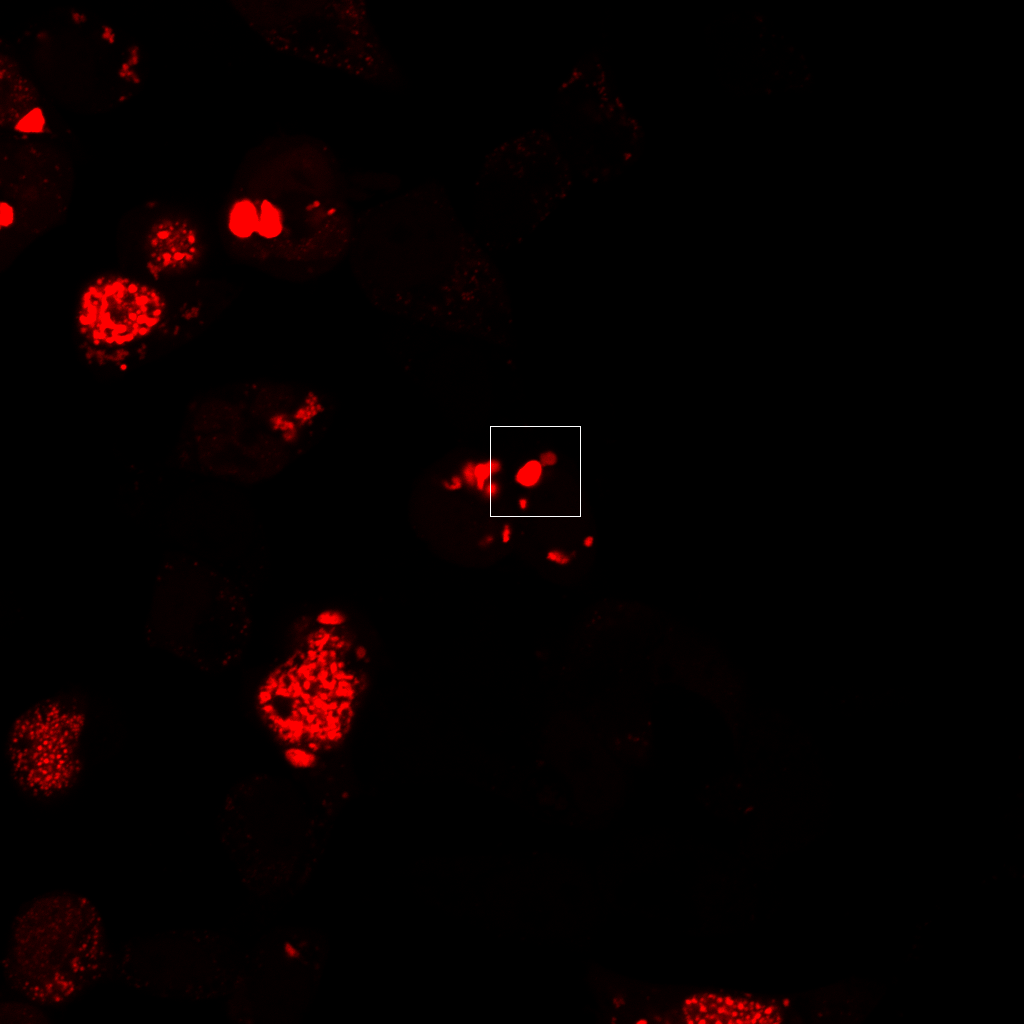

Supplement: Supplementary file 9 — Source data Fig. 6 [file 44318_2024_120_MOESM9_ESM.zip › Figure 6/6D/30s.tif]

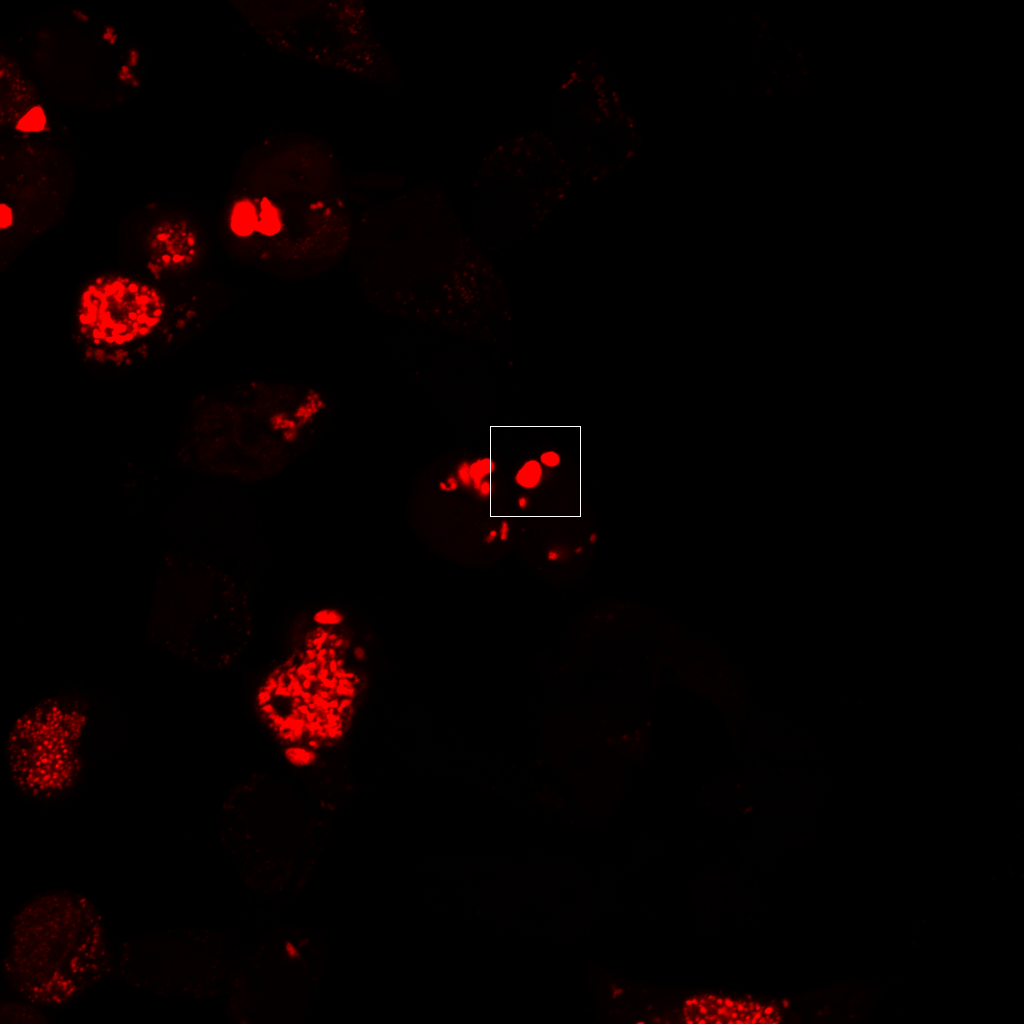

Supplement: Supplementary file 9 — Source data Fig. 6 [file 44318_2024_120_MOESM9_ESM.zip › Figure 6/6D/Pre.tif]

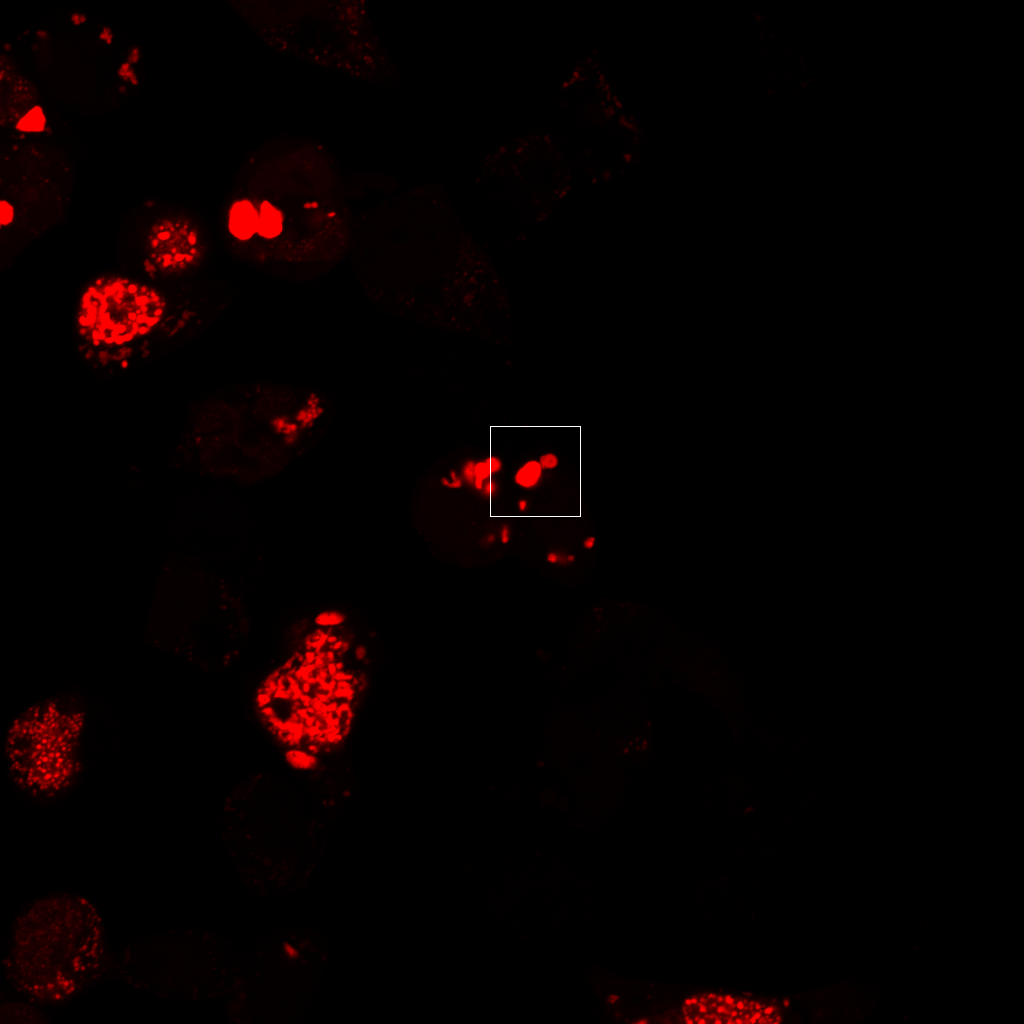

Supplement: Supplementary file 9 — Source data Fig. 6 [file 44318_2024_120_MOESM9_ESM.zip › Figure 6/6D/60s.tif]

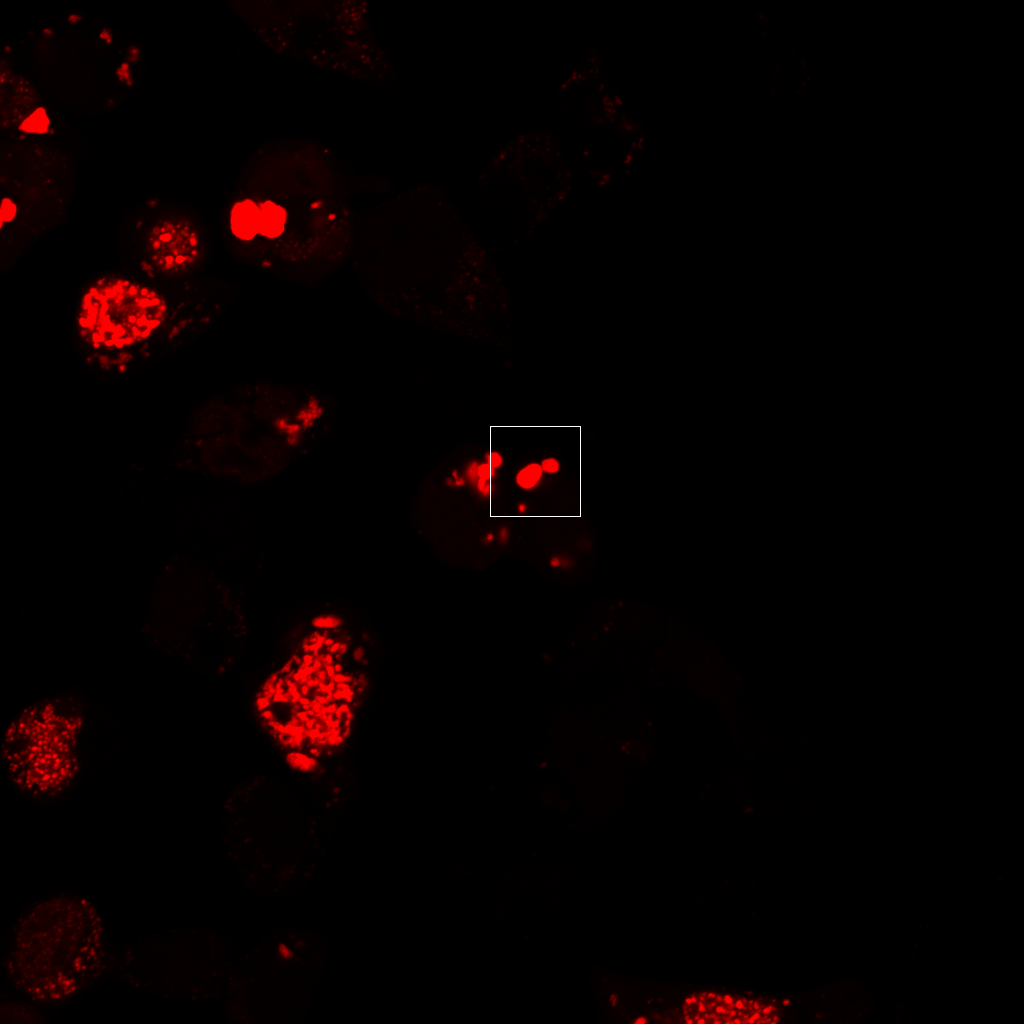

Supplement: Supplementary file 9 — Source data Fig. 6 [file 44318_2024_120_MOESM9_ESM.zip › Figure 6/6D/120s.tif]

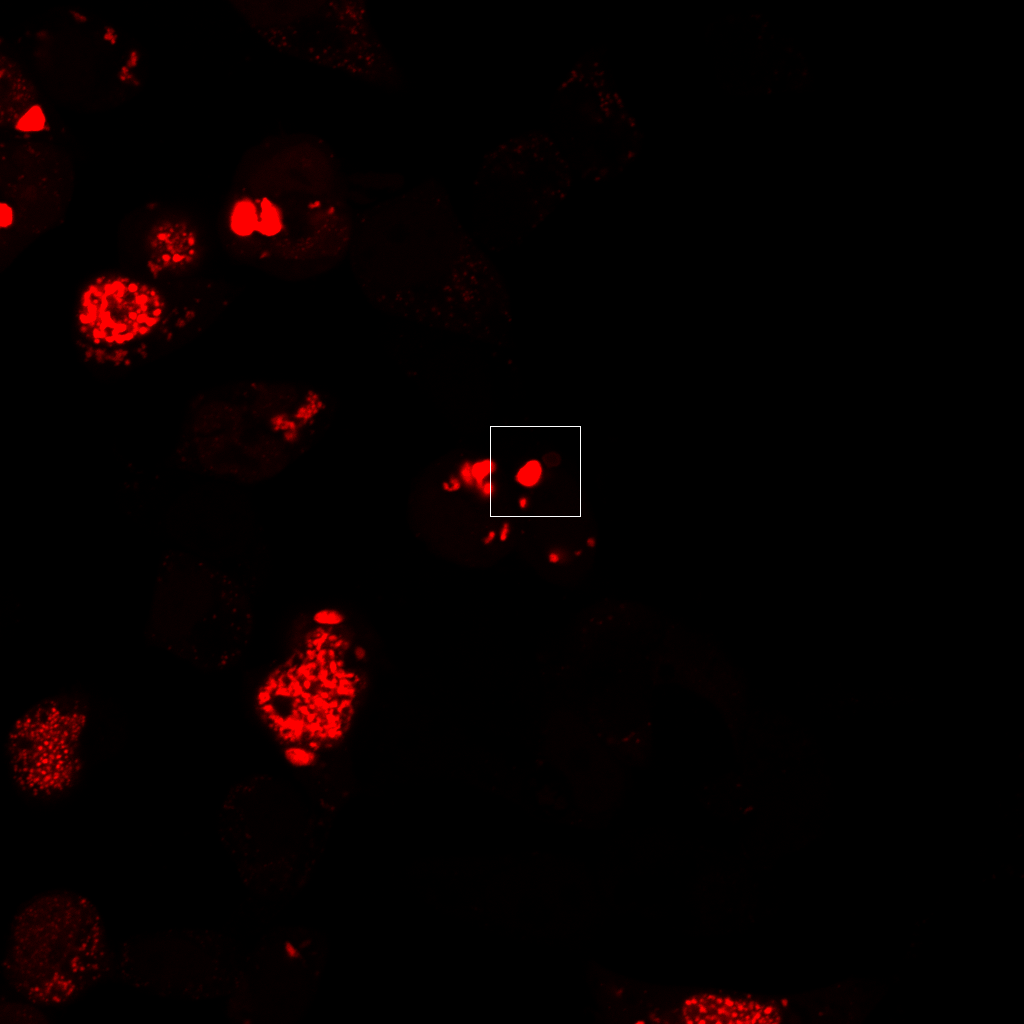

Supplement: Supplementary file 9 — Source data Fig. 6 [file 44318_2024_120_MOESM9_ESM.zip › Figure 6/6D/Bleaching.tif]

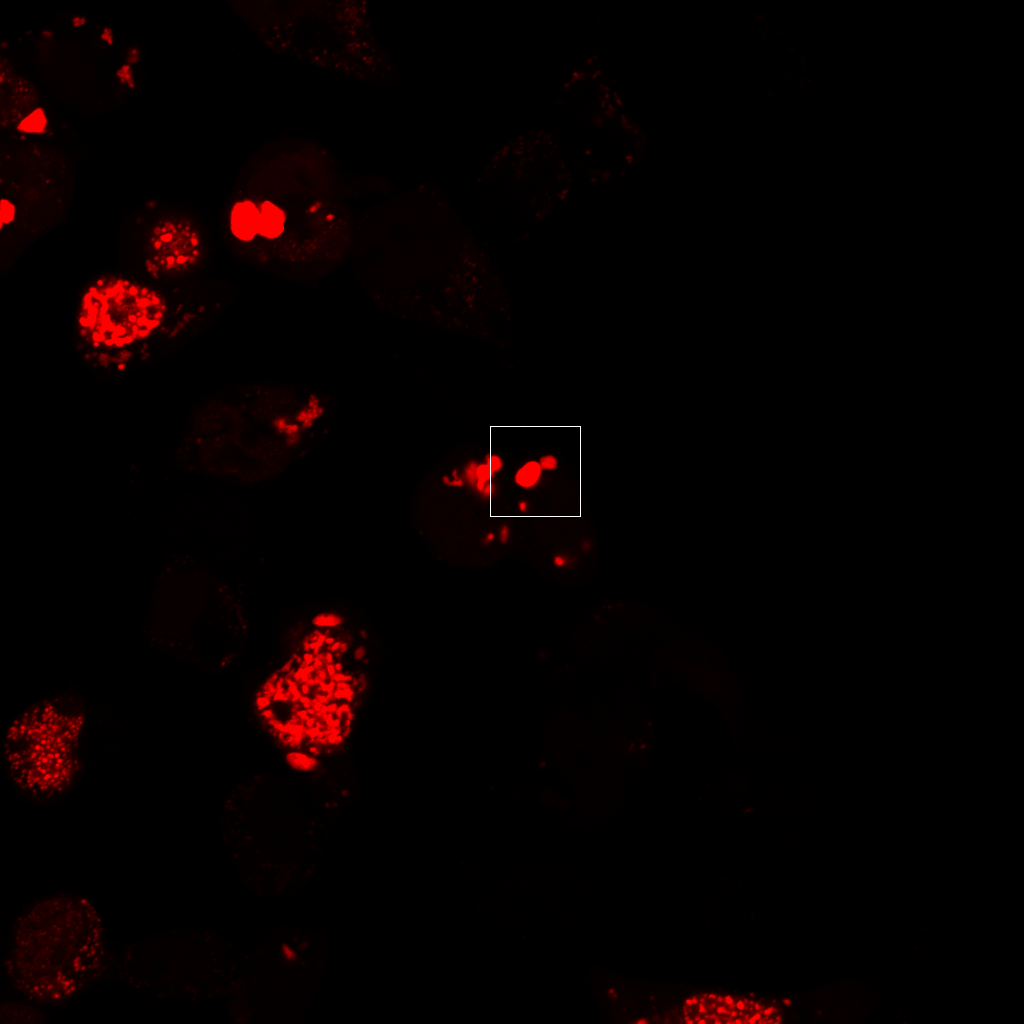

Supplement: Supplementary file 9 — Source data Fig. 6 [file 44318_2024_120_MOESM9_ESM.zip › Figure 6/6D/90s.tif]

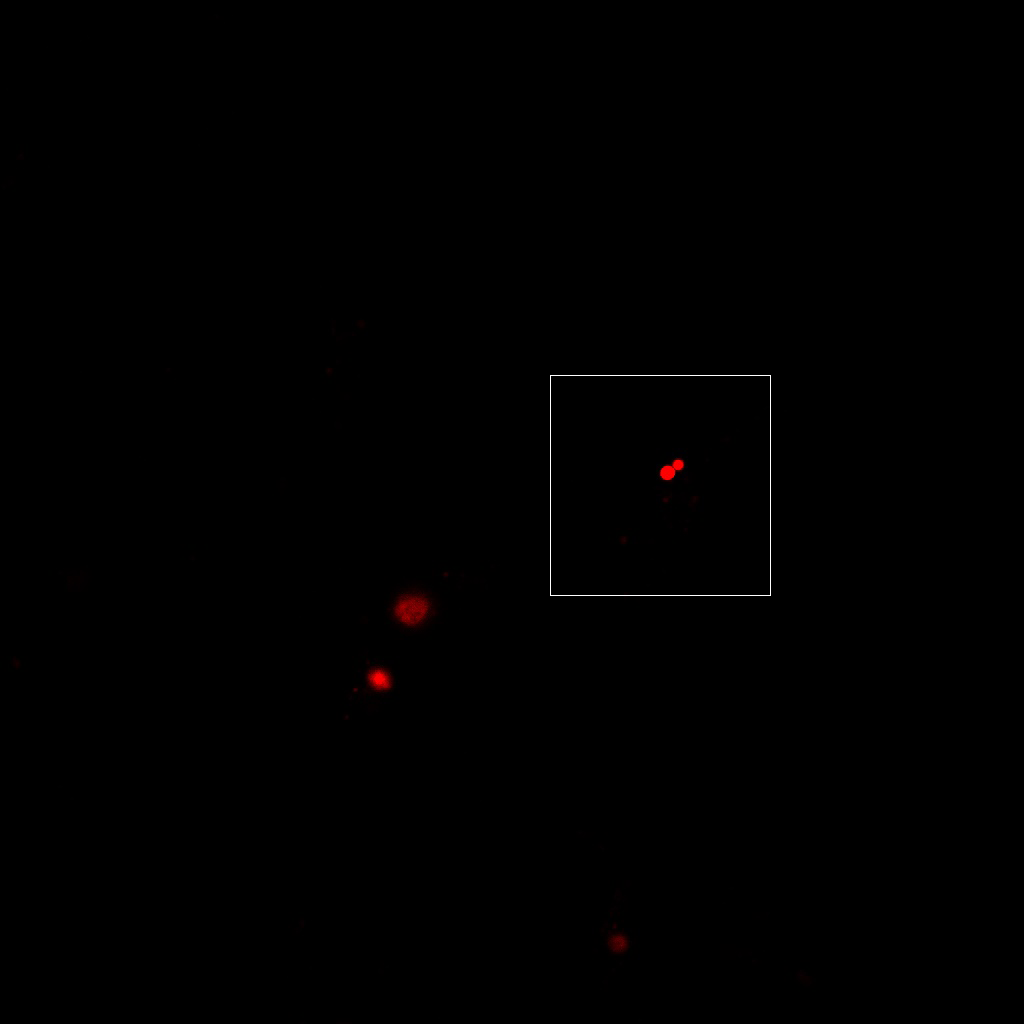

Supplement: Supplementary file 9 — Source data Fig. 6 [file 44318_2024_120_MOESM9_ESM.zip › Figure 6/6C/0min.tif]

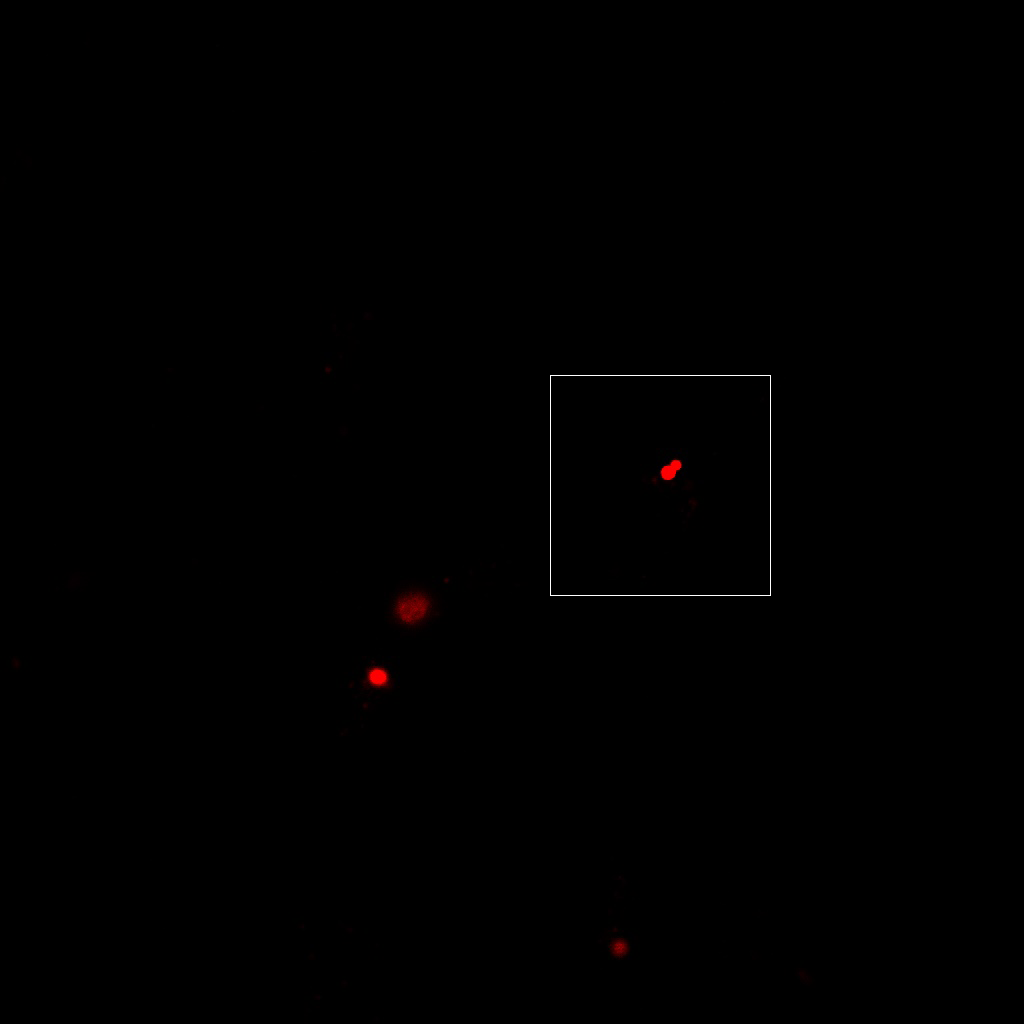

Supplement: Supplementary file 9 — Source data Fig. 6 [file 44318_2024_120_MOESM9_ESM.zip › Figure 6/6C/6min.tif]

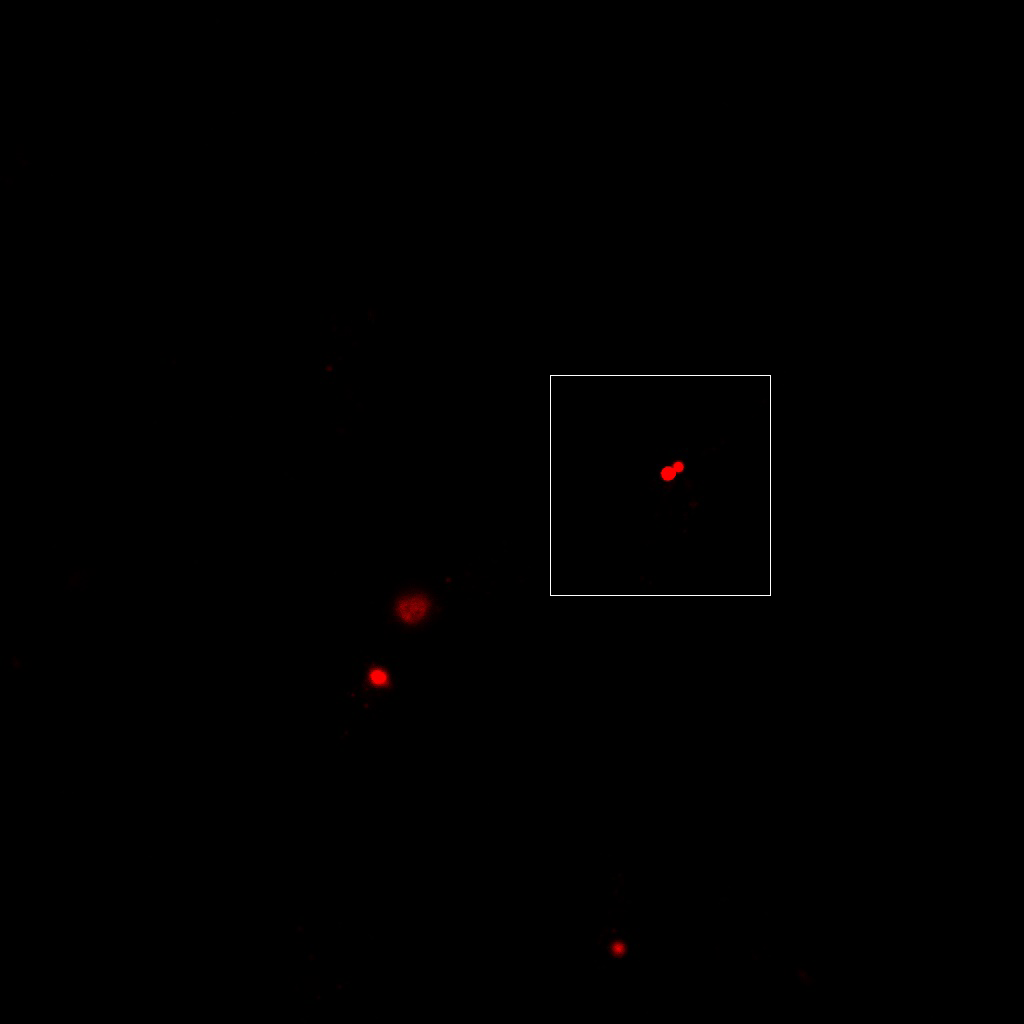

Supplement: Supplementary file 9 — Source data Fig. 6 [file 44318_2024_120_MOESM9_ESM.zip › Figure 6/6C/3min.tif]
