# Supplementary figures and images for: Energy stress promotes P-bodies formation via lysine-63-linked polyubiquitination of HAX1 (part 4 of 4)
Source: EMBO J. 2024 May 20;43(13):11. doi: 10.1038/s44318-024-00120-6 (PMC11217408; doi:10.1038/s44318-024-00120-6)

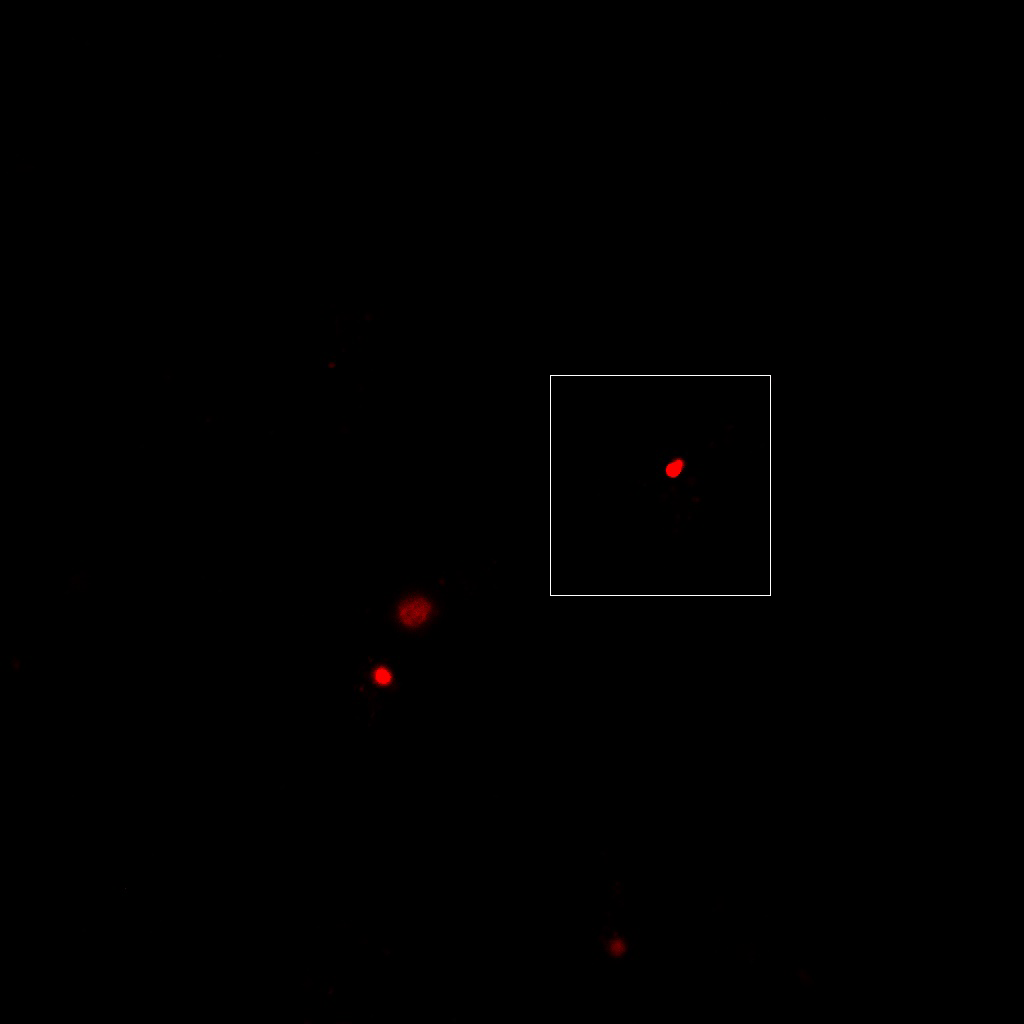

Supplement: Supplementary file 9 — Source data Fig. 6 [file 44318_2024_120_MOESM9_ESM.zip › Figure 6/6C/9min.tif]

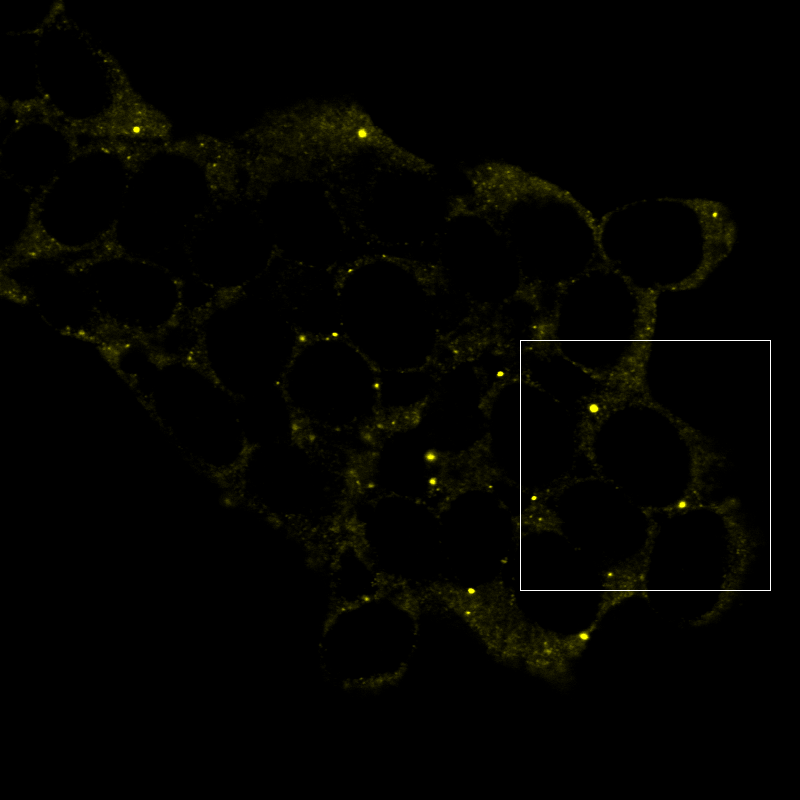

Supplement: Supplementary file 9 — Source data Fig. 6 [file 44318_2024_120_MOESM9_ESM.zip › Figure 6/6F/IDR1/LSM14A.tif]

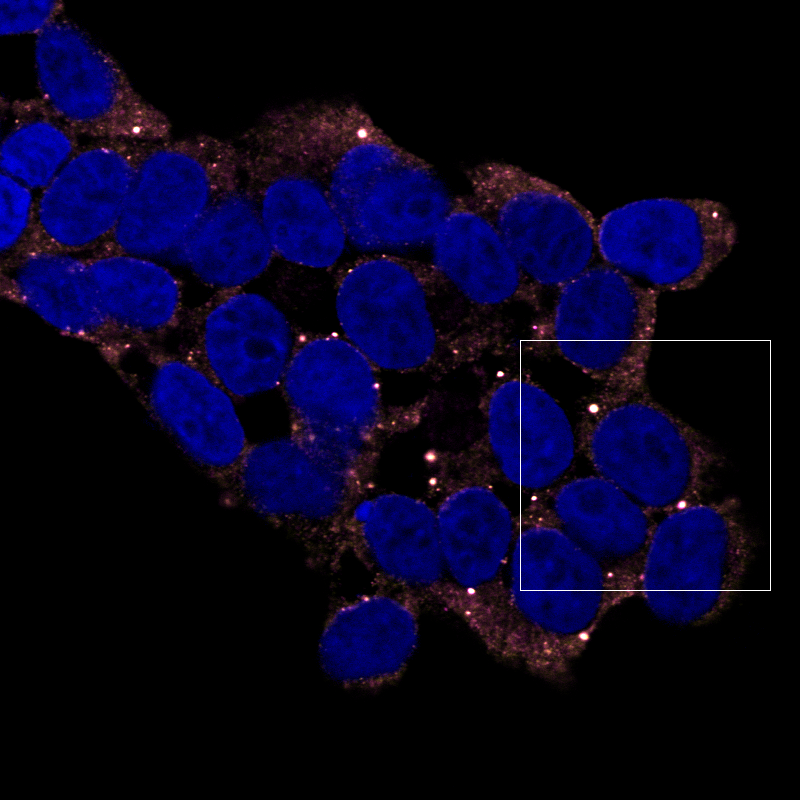

Supplement: Supplementary file 9 — Source data Fig. 6 [file 44318_2024_120_MOESM9_ESM.zip › Figure 6/6F/IDR1/Merge.tif]

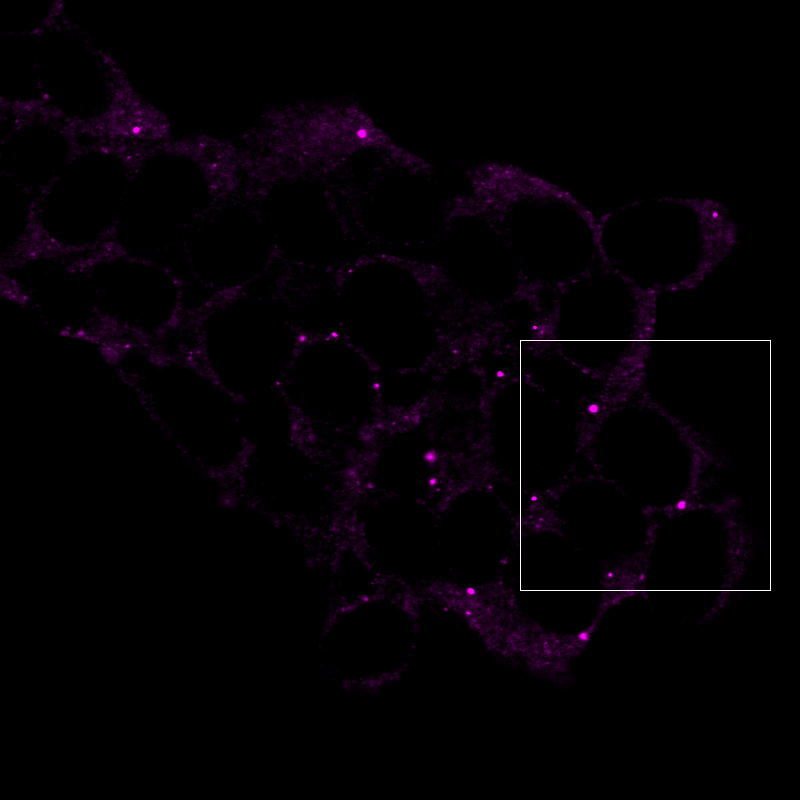

Supplement: Supplementary file 9 — Source data Fig. 6 [file 44318_2024_120_MOESM9_ESM.zip › Figure 6/6F/IDR1/HAX1.tif]

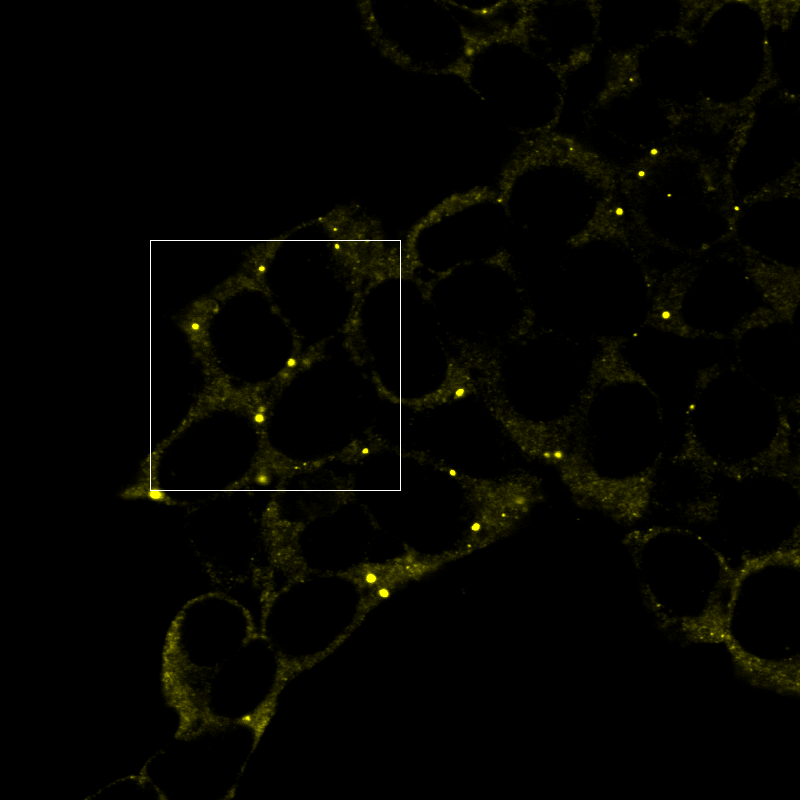

Supplement: Supplementary file 9 — Source data Fig. 6 [file 44318_2024_120_MOESM9_ESM.zip › Figure 6/6F/FL/LSM14A.tif]

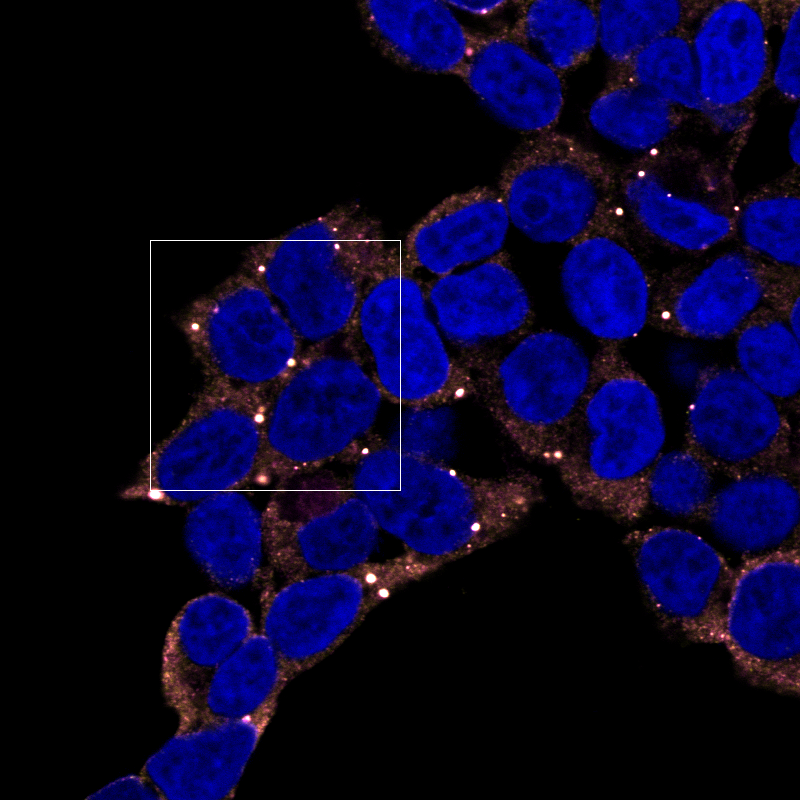

Supplement: Supplementary file 9 — Source data Fig. 6 [file 44318_2024_120_MOESM9_ESM.zip › Figure 6/6F/FL/Merge.tif]

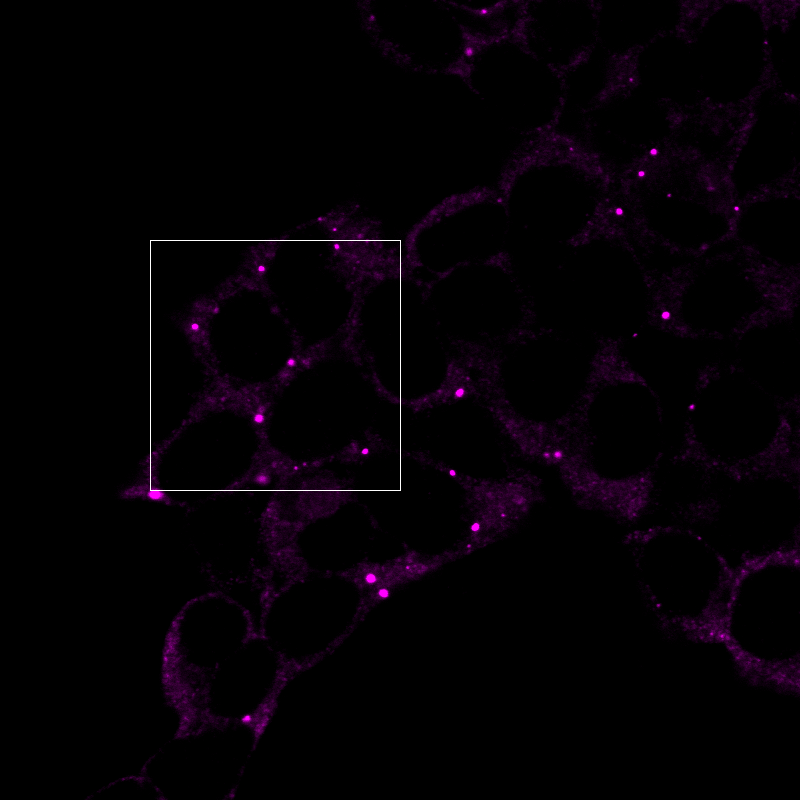

Supplement: Supplementary file 9 — Source data Fig. 6 [file 44318_2024_120_MOESM9_ESM.zip › Figure 6/6F/FL/HAX1.tif]

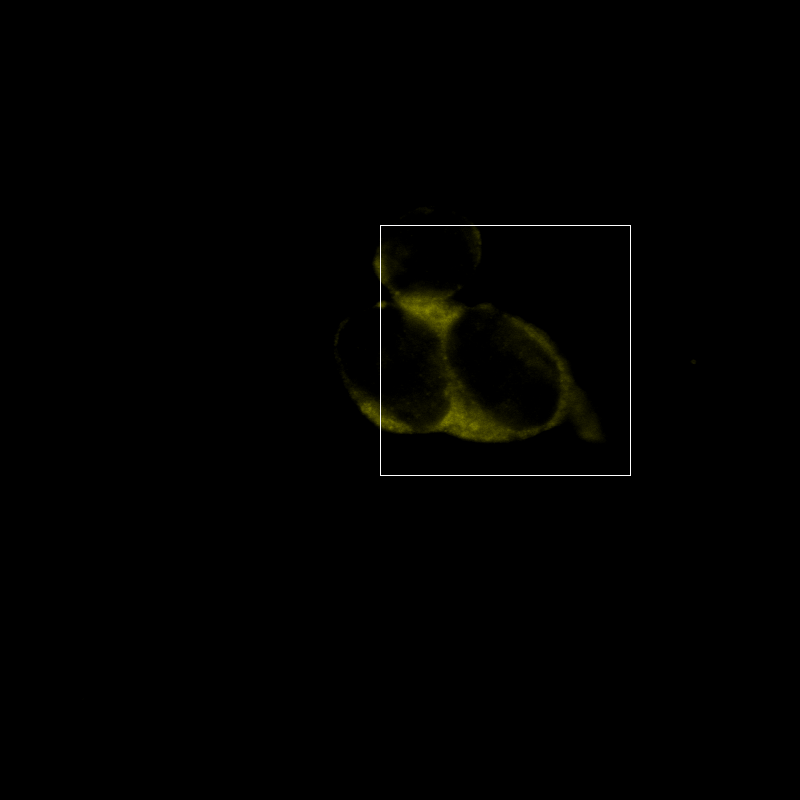

Supplement: Supplementary file 9 — Source data Fig. 6 [file 44318_2024_120_MOESM9_ESM.zip › Figure 6/6F/IDR2/LSM14A.tif]

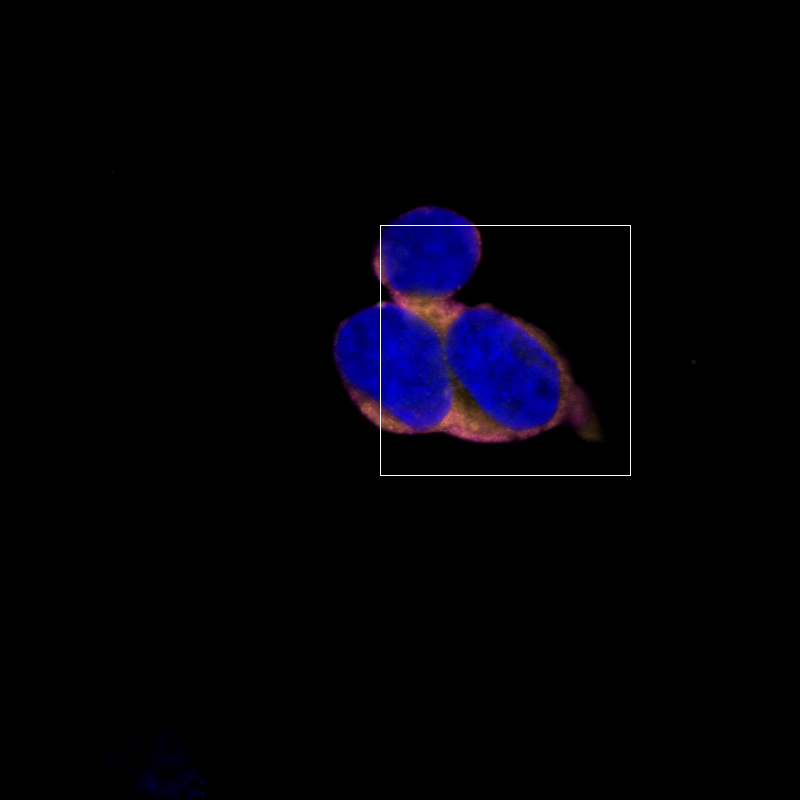

Supplement: Supplementary file 9 — Source data Fig. 6 [file 44318_2024_120_MOESM9_ESM.zip › Figure 6/6F/IDR2/Merge.tif]

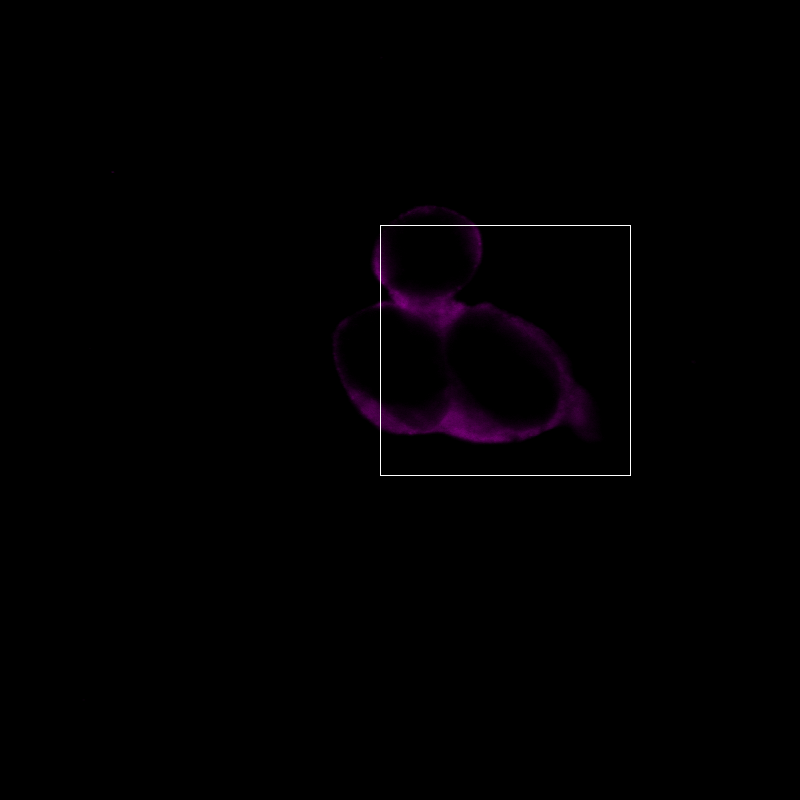

Supplement: Supplementary file 9 — Source data Fig. 6 [file 44318_2024_120_MOESM9_ESM.zip › Figure 6/6F/IDR2/HAX1.tif]

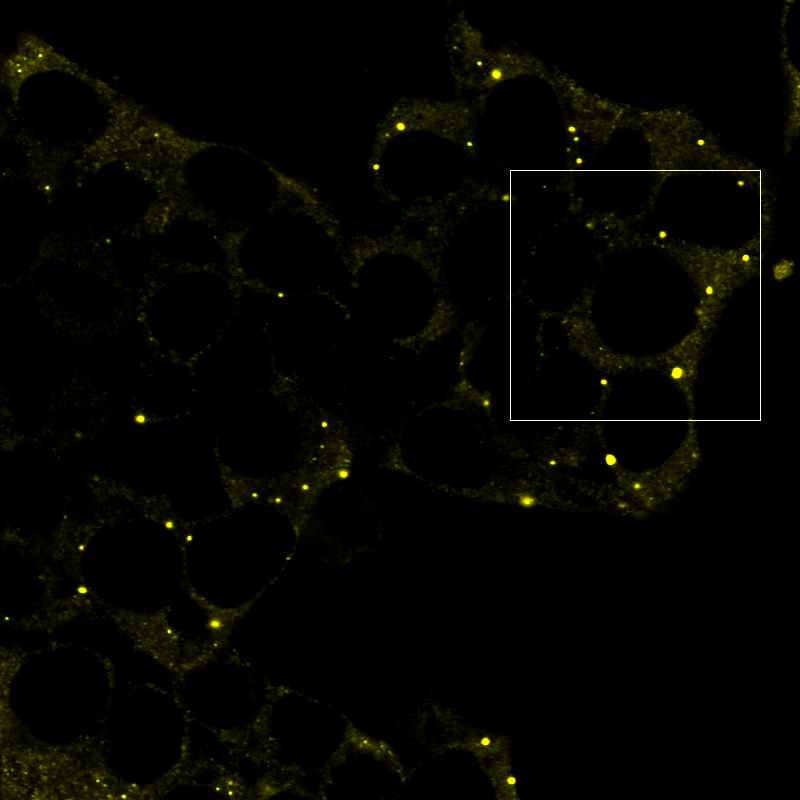

Supplement: Supplementary file 9 — Source data Fig. 6 [file 44318_2024_120_MOESM9_ESM.zip › Figure 6/6F/IDR3/LSM14A.tif]

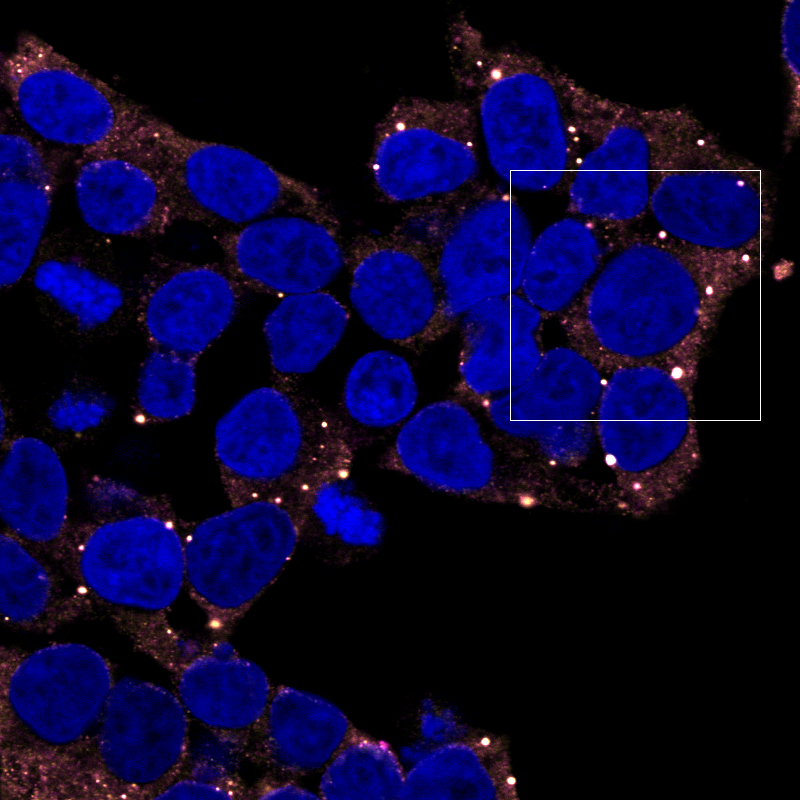

Supplement: Supplementary file 9 — Source data Fig. 6 [file 44318_2024_120_MOESM9_ESM.zip › Figure 6/6F/IDR3/Merge.tif]

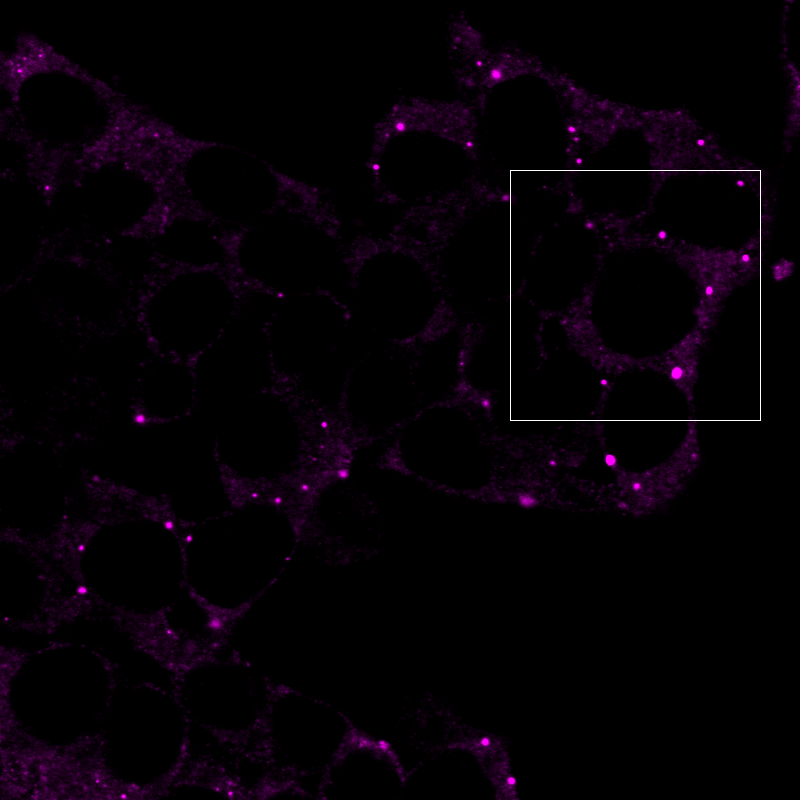

Supplement: Supplementary file 9 — Source data Fig. 6 [file 44318_2024_120_MOESM9_ESM.zip › Figure 6/6F/IDR3/HAX1.tif]

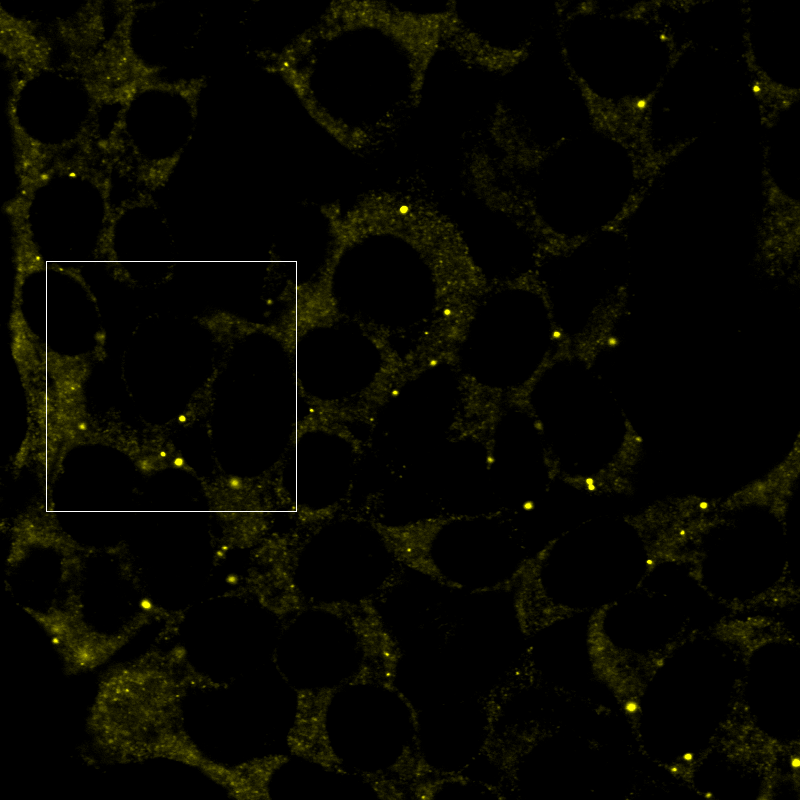

Supplement: Supplementary file 9 — Source data Fig. 6 [file 44318_2024_120_MOESM9_ESM.zip › Figure 6/6F/IDR4/LSM14A.tif]

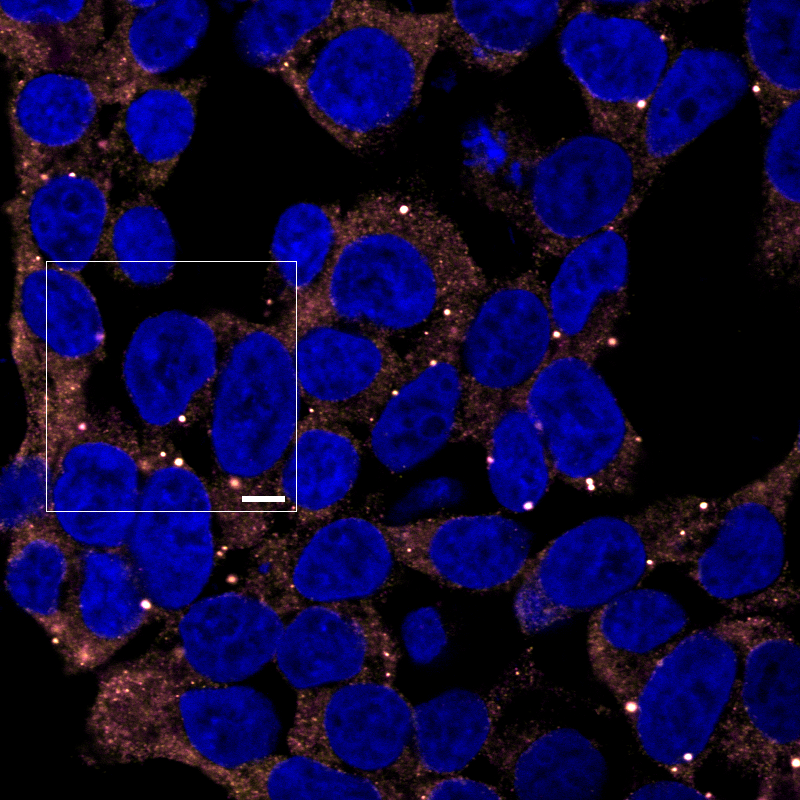

Supplement: Supplementary file 9 — Source data Fig. 6 [file 44318_2024_120_MOESM9_ESM.zip › Figure 6/6F/IDR4/Merge.tif]

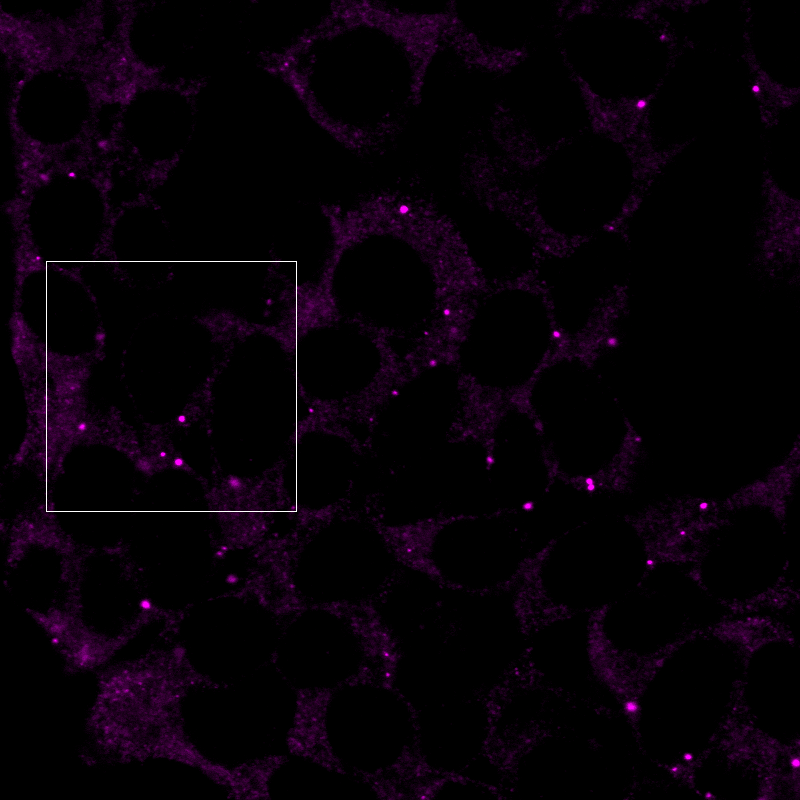

Supplement: Supplementary file 9 — Source data Fig. 6 [file 44318_2024_120_MOESM9_ESM.zip › Figure 6/6F/IDR4/HAX1.tif]

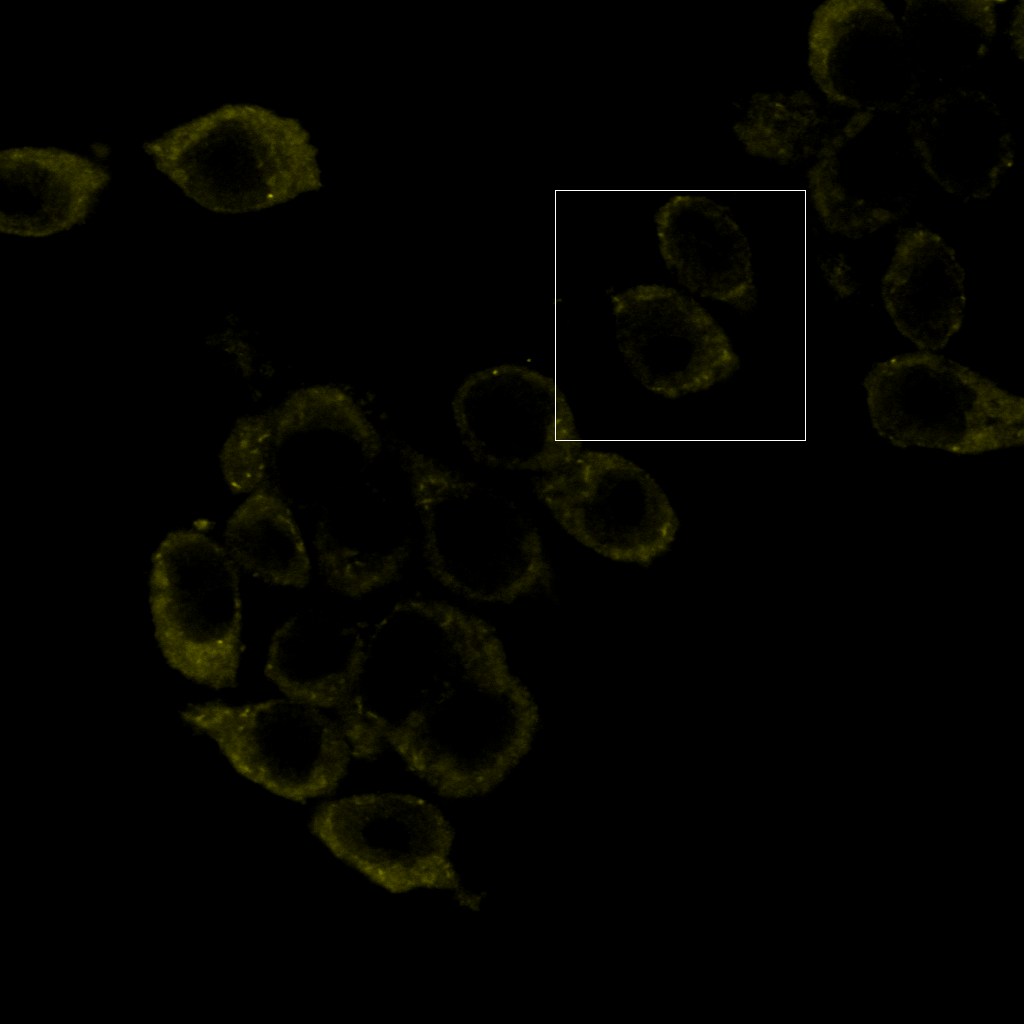

Supplement: Supplementary file 9 — Source data Fig. 6 [file 44318_2024_120_MOESM9_ESM.zip › Figure 6/6F/Vector/LSM14A.tif]

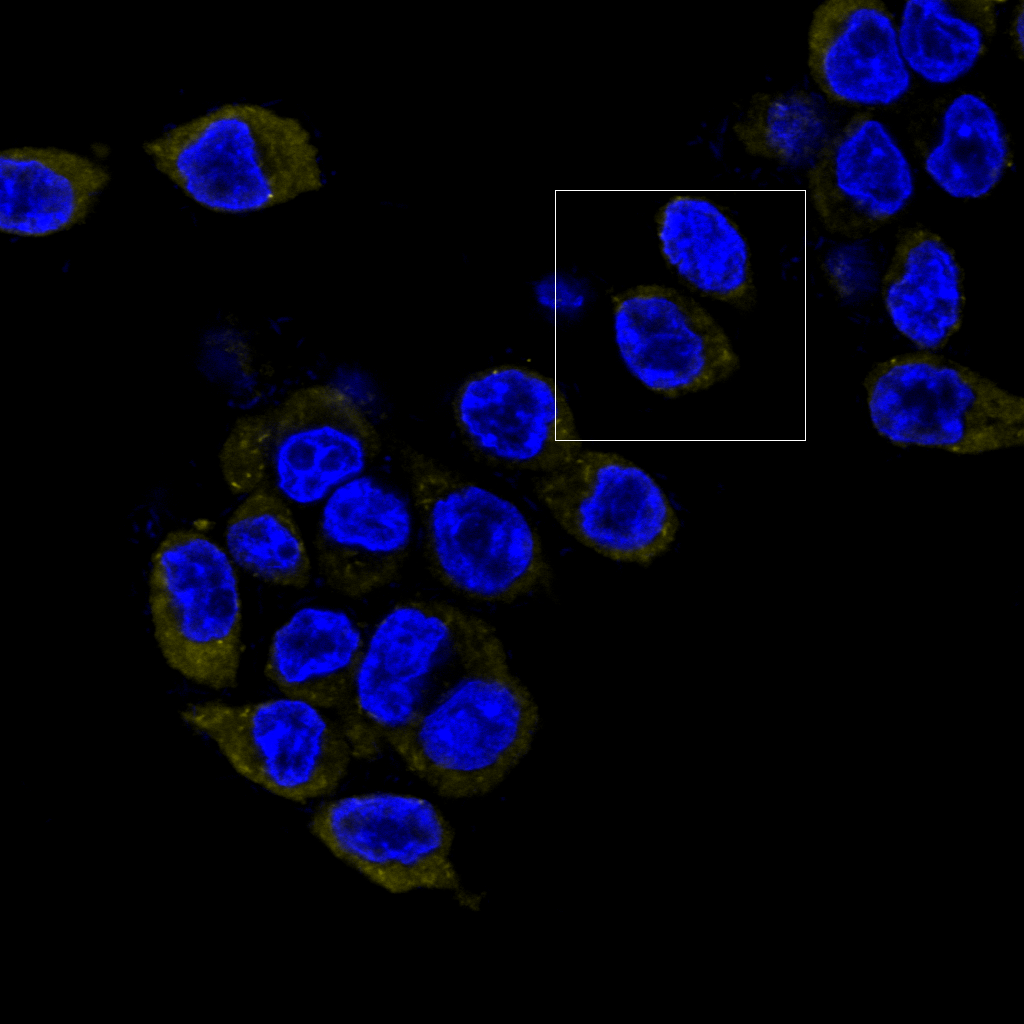

Supplement: Supplementary file 9 — Source data Fig. 6 [file 44318_2024_120_MOESM9_ESM.zip › Figure 6/6F/Vector/Merge.tif]

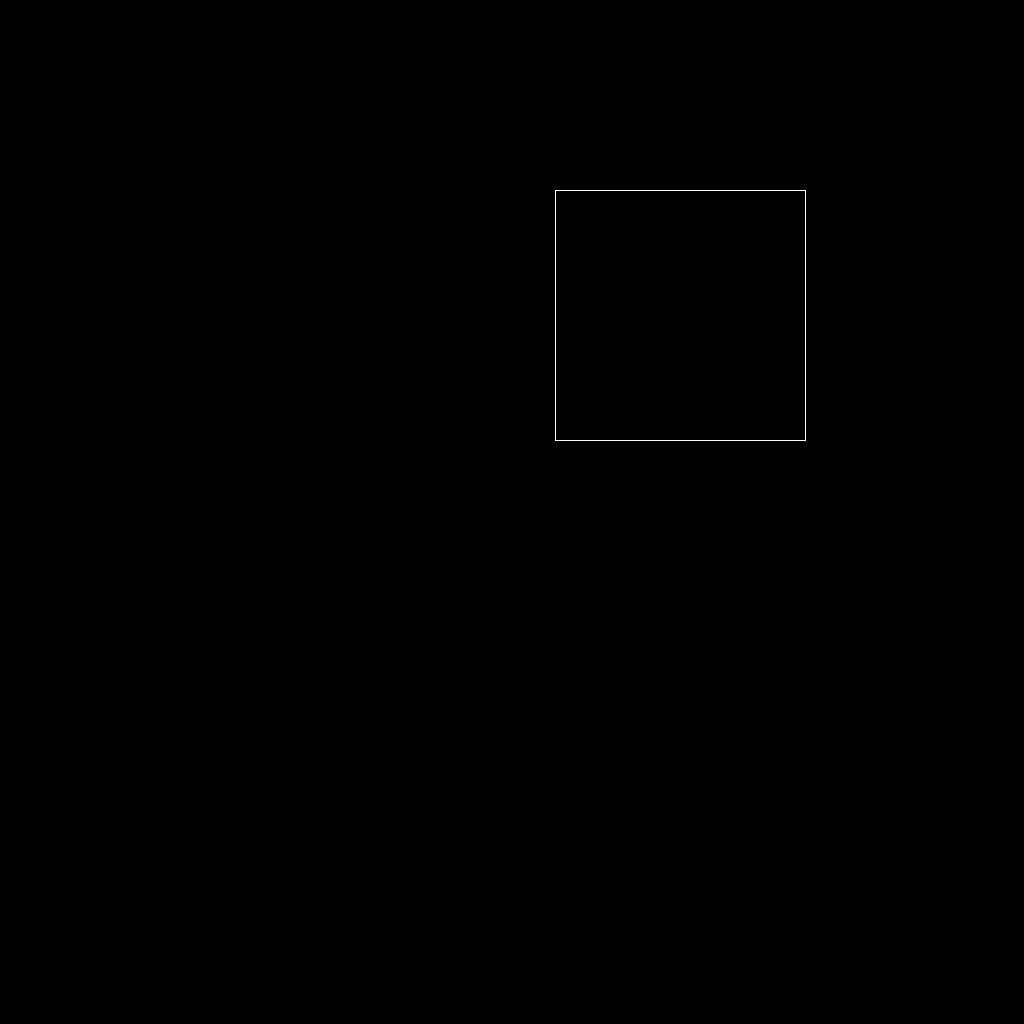

Supplement: Supplementary file 9 — Source data Fig. 6 [file 44318_2024_120_MOESM9_ESM.zip › Figure 6/6F/Vector/HAX1.tif]

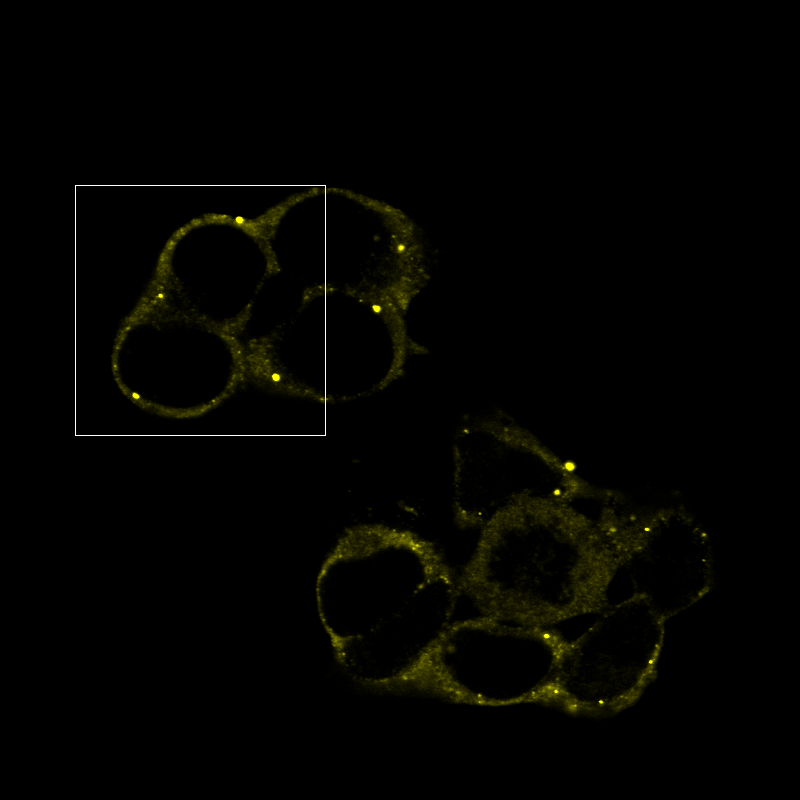

Supplement: Supplementary file 9 — Source data Fig. 6 [file 44318_2024_120_MOESM9_ESM.zip › Figure 6/6H/HAX1-WT-mCherry/Mock/LSM14A.tif]

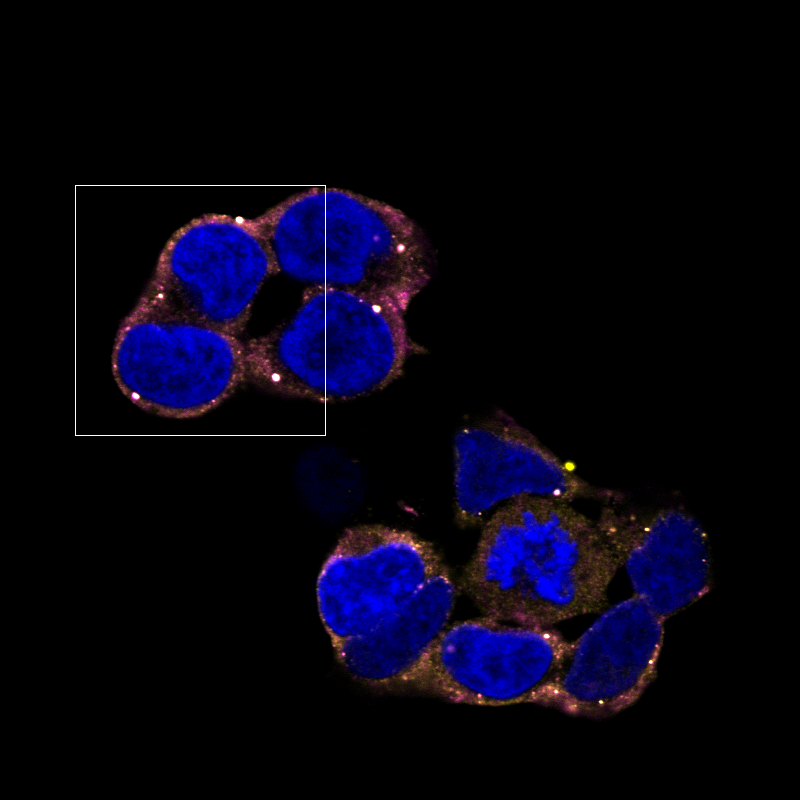

Supplement: Supplementary file 9 — Source data Fig. 6 [file 44318_2024_120_MOESM9_ESM.zip › Figure 6/6H/HAX1-WT-mCherry/Mock/Merge.tif]

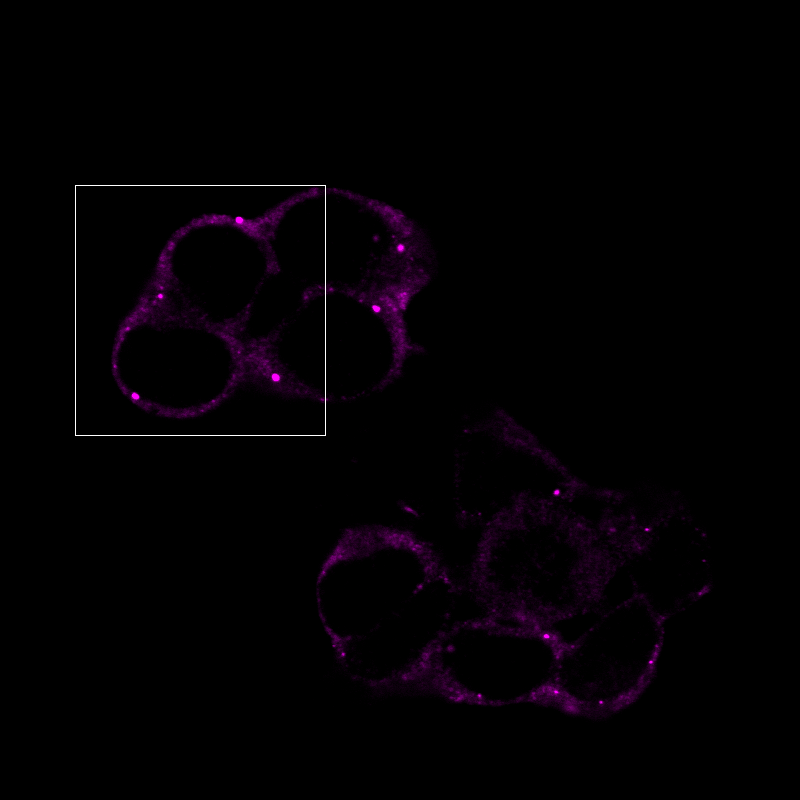

Supplement: Supplementary file 9 — Source data Fig. 6 [file 44318_2024_120_MOESM9_ESM.zip › Figure 6/6H/HAX1-WT-mCherry/Mock/HAX1.tif]

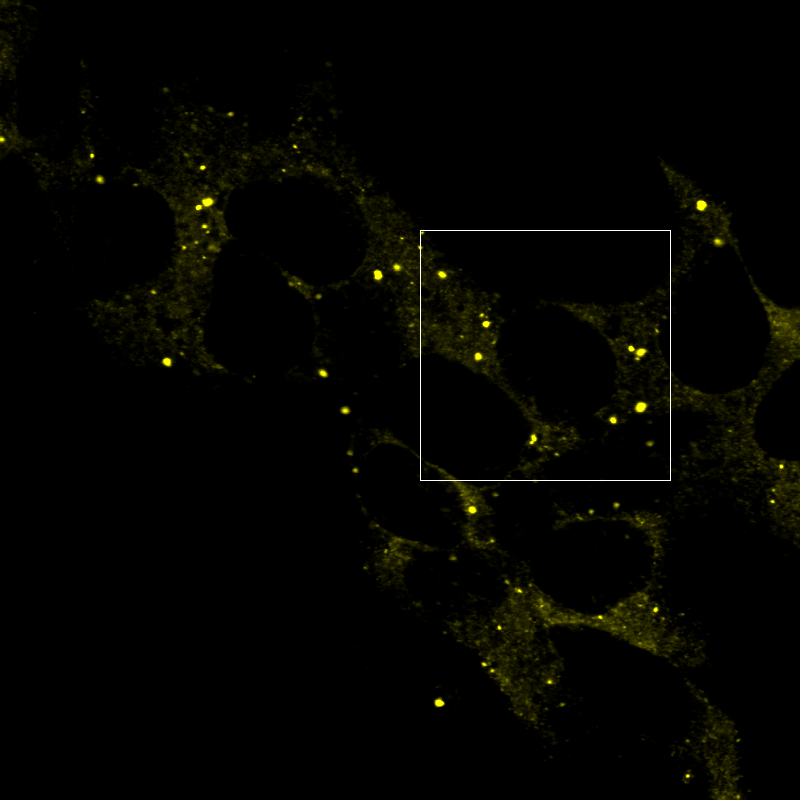

Supplement: Supplementary file 9 — Source data Fig. 6 [file 44318_2024_120_MOESM9_ESM.zip › Figure 6/6H/HAX1-WT-mCherry/Oligomycin/LSM14A.tif]

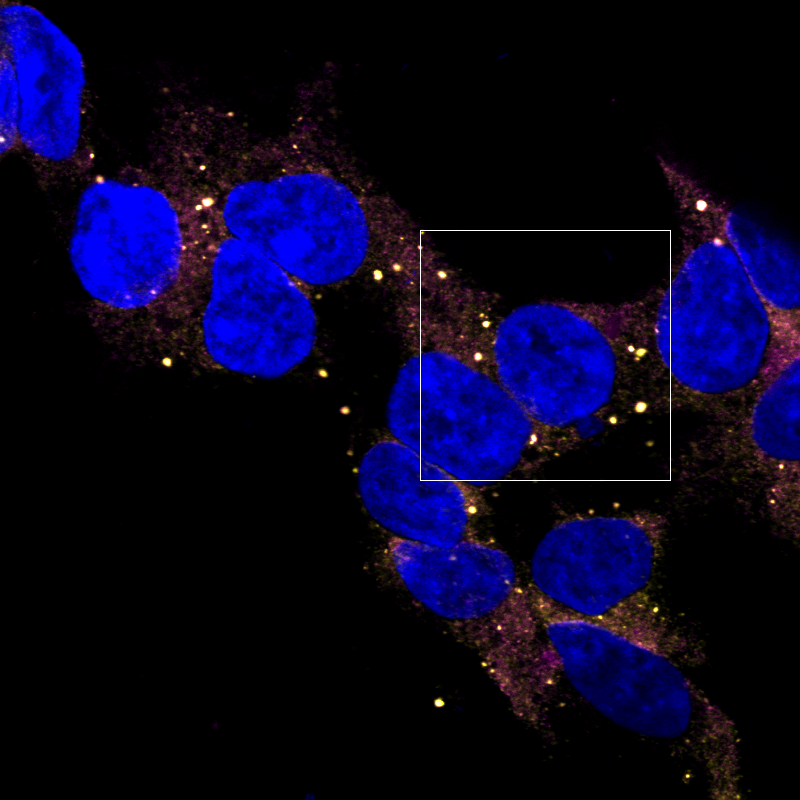

Supplement: Supplementary file 9 — Source data Fig. 6 [file 44318_2024_120_MOESM9_ESM.zip › Figure 6/6H/HAX1-WT-mCherry/Oligomycin/Merge.tif]

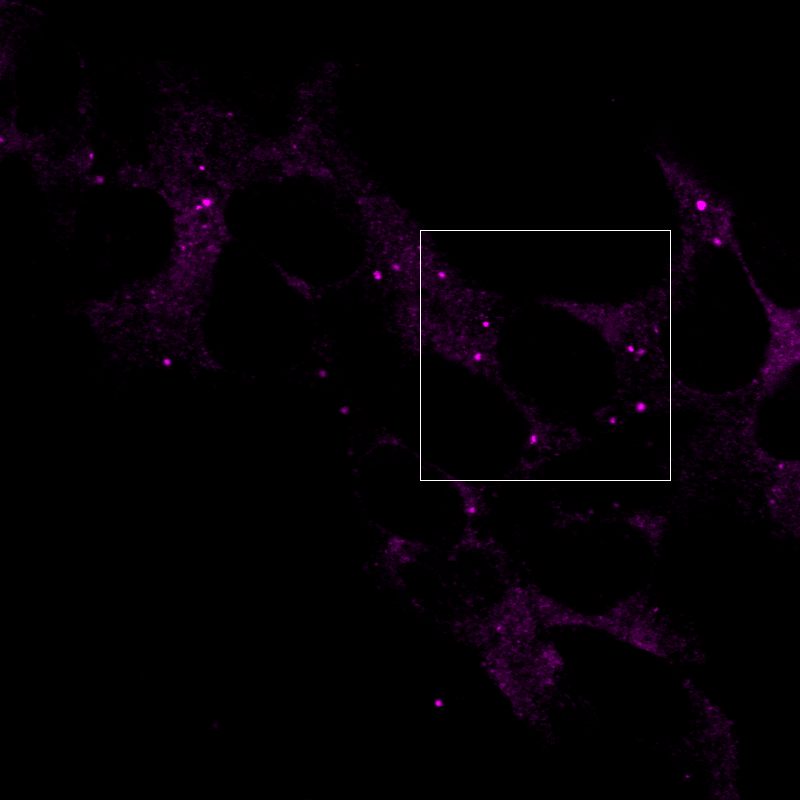

Supplement: Supplementary file 9 — Source data Fig. 6 [file 44318_2024_120_MOESM9_ESM.zip › Figure 6/6H/HAX1-WT-mCherry/Oligomycin/HAX1.tif]

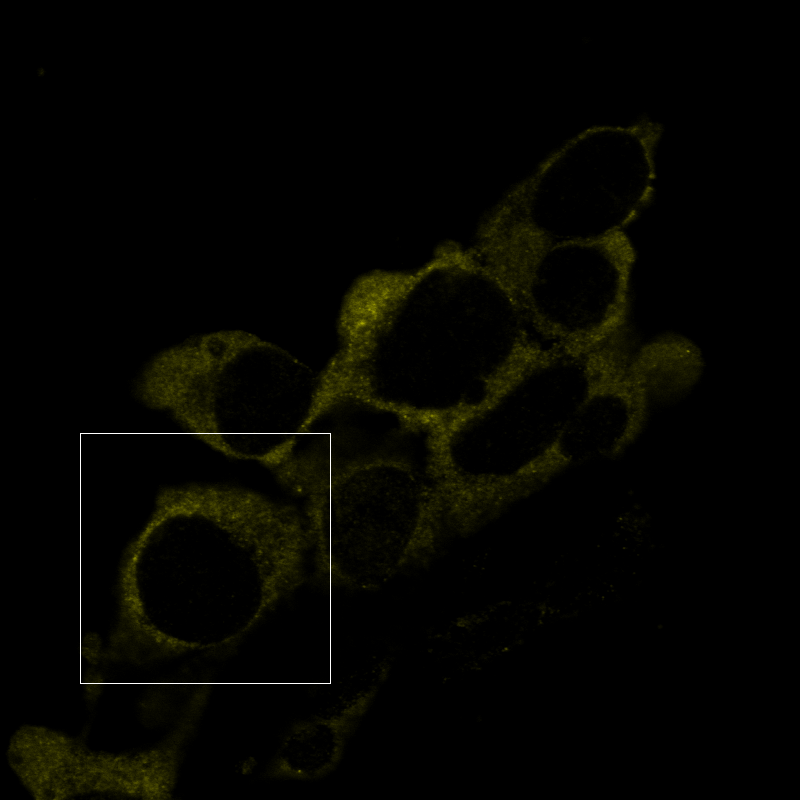

Supplement: Supplementary file 9 — Source data Fig. 6 [file 44318_2024_120_MOESM9_ESM.zip › Figure 6/6H/HAX1-K131R-mCherry/Mock/LSM14A.tif]

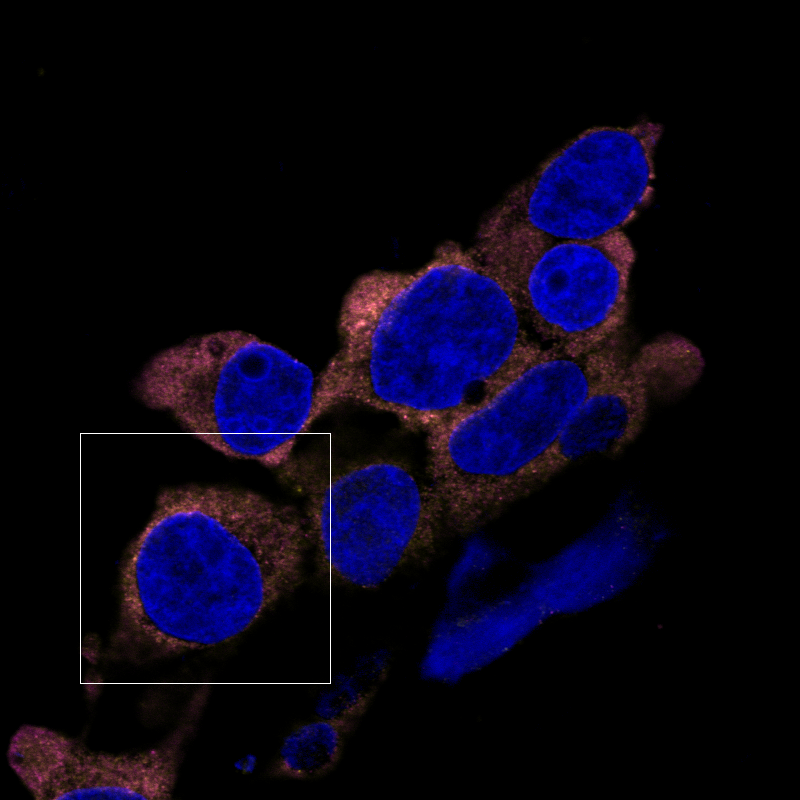

Supplement: Supplementary file 9 — Source data Fig. 6 [file 44318_2024_120_MOESM9_ESM.zip › Figure 6/6H/HAX1-K131R-mCherry/Mock/Merge.tif]

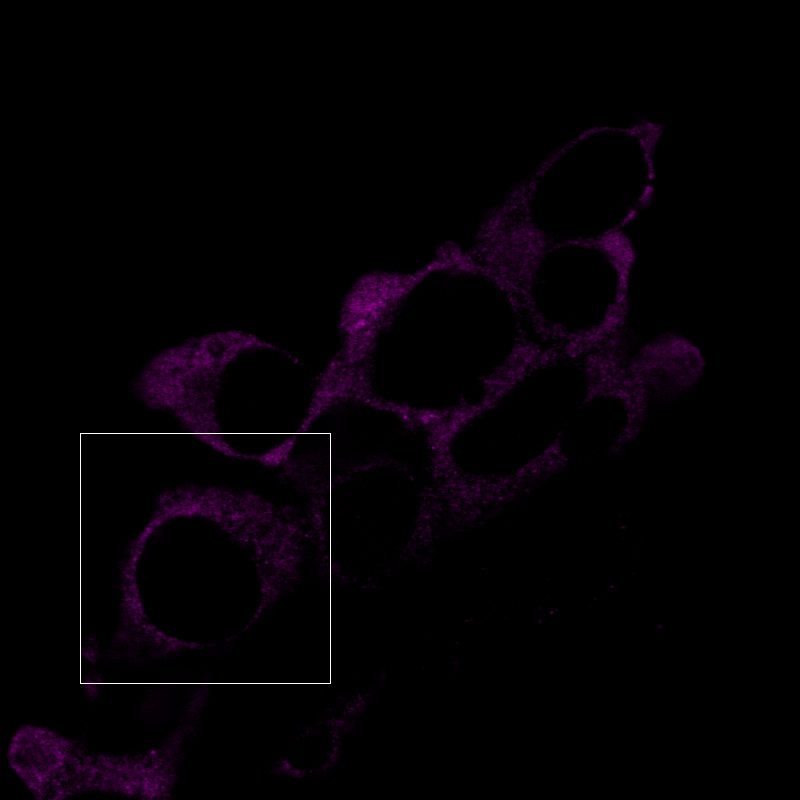

Supplement: Supplementary file 9 — Source data Fig. 6 [file 44318_2024_120_MOESM9_ESM.zip › Figure 6/6H/HAX1-K131R-mCherry/Mock/HAX1.tif]

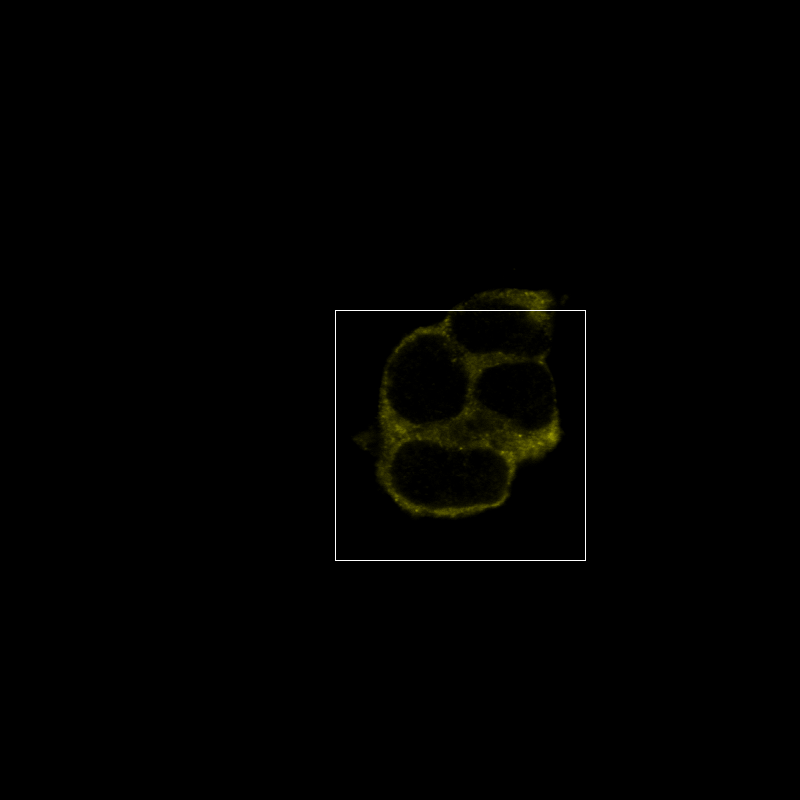

Supplement: Supplementary file 9 — Source data Fig. 6 [file 44318_2024_120_MOESM9_ESM.zip › Figure 6/6H/HAX1-K131R-mCherry/Oligomycin/LSM14A.tif]

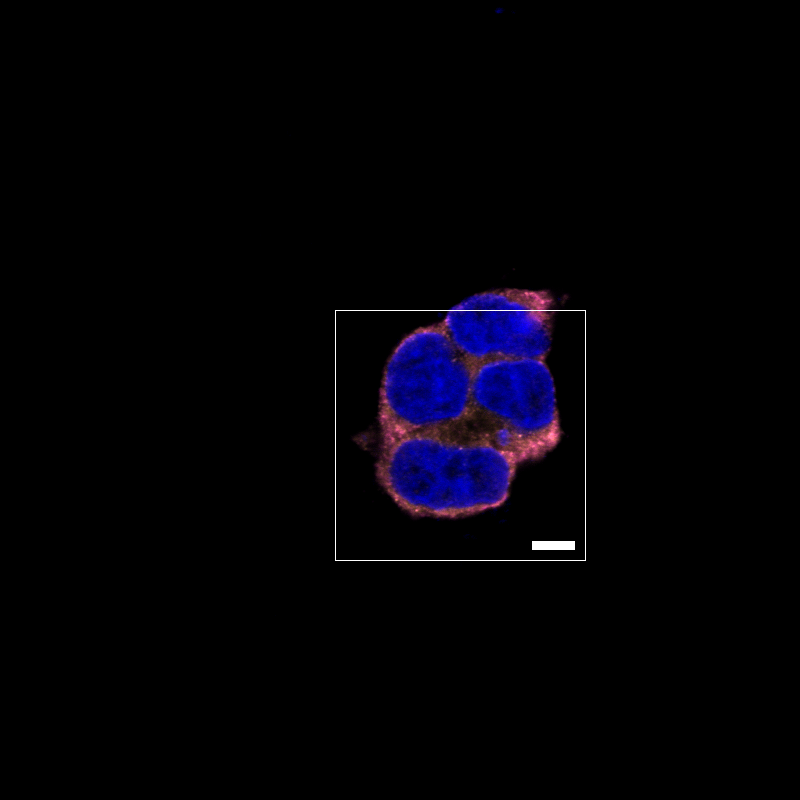

Supplement: Supplementary file 9 — Source data Fig. 6 [file 44318_2024_120_MOESM9_ESM.zip › Figure 6/6H/HAX1-K131R-mCherry/Oligomycin/Merge.tif]

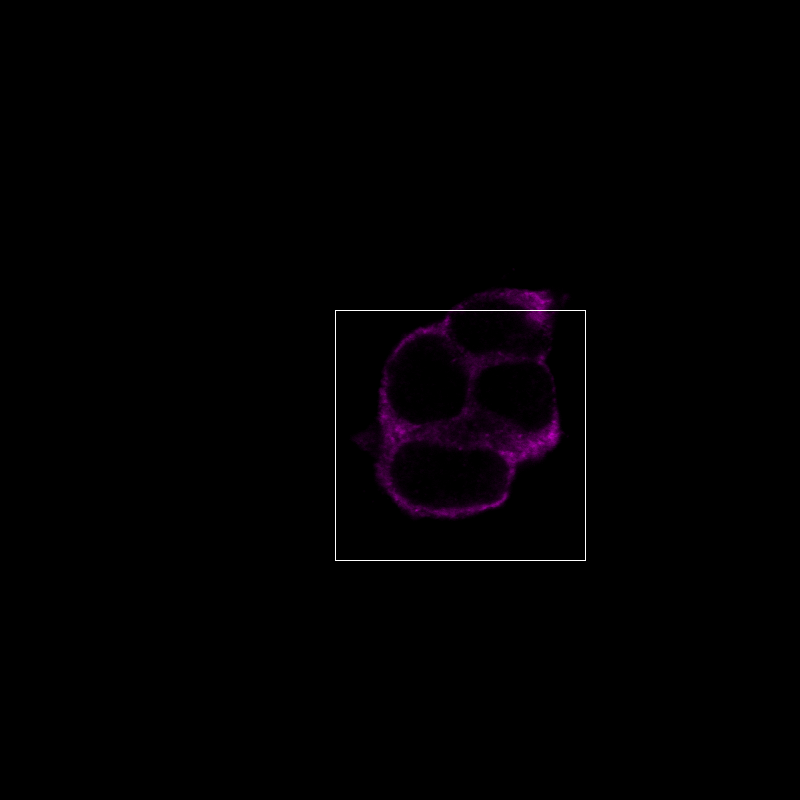

Supplement: Supplementary file 9 — Source data Fig. 6 [file 44318_2024_120_MOESM9_ESM.zip › Figure 6/6H/HAX1-K131R-mCherry/Oligomycin/HAX1.tif]

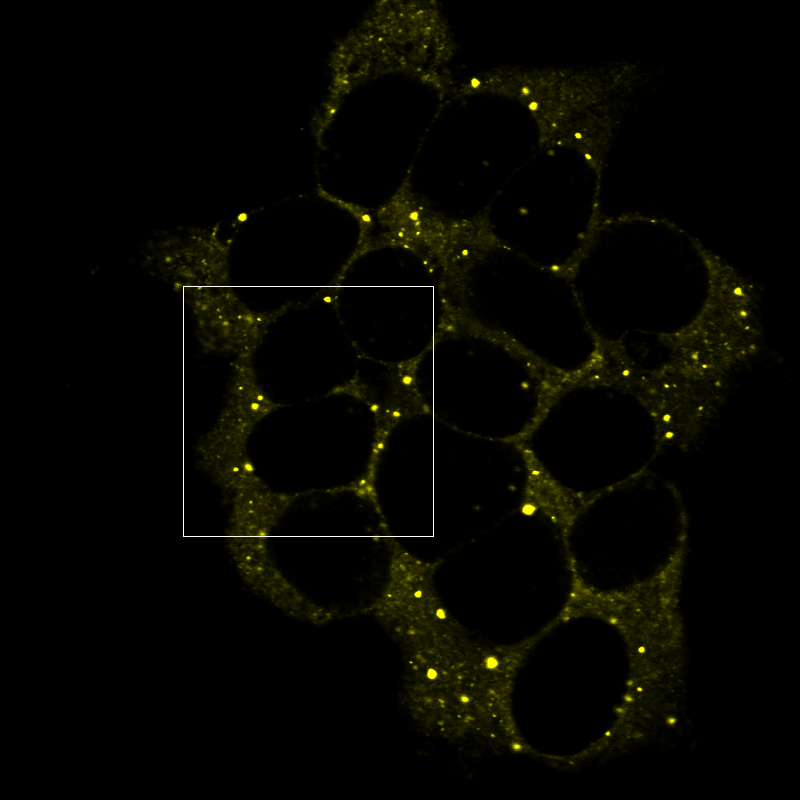

Supplement: Supplementary file 9 — Source data Fig. 6 [file 44318_2024_120_MOESM9_ESM.zip › Figure 6/6J/HAX1-WT-mCherry/TRIM23/LSM14A.tif]

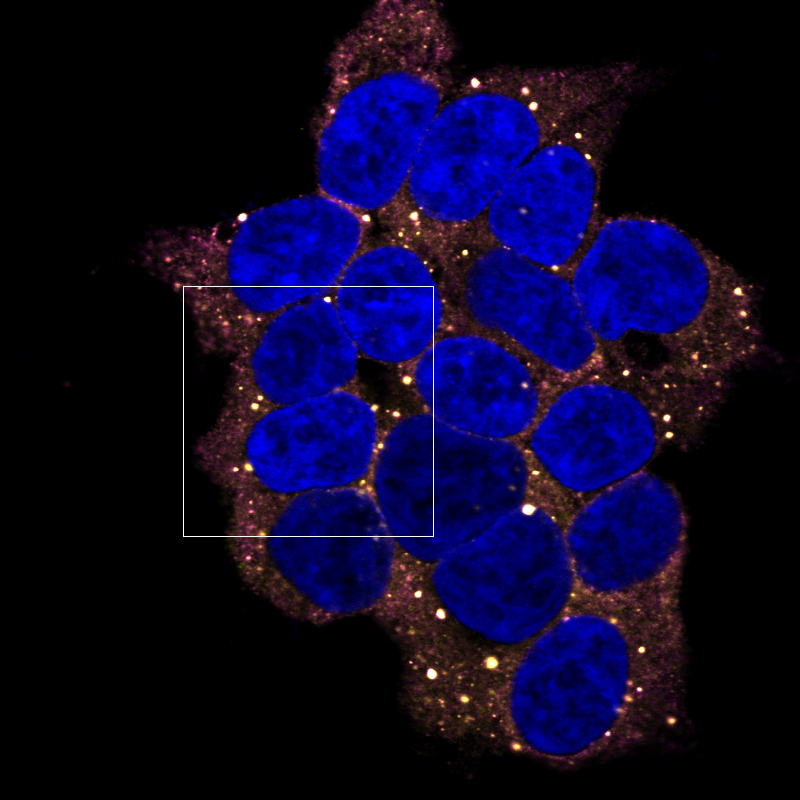

Supplement: Supplementary file 9 — Source data Fig. 6 [file 44318_2024_120_MOESM9_ESM.zip › Figure 6/6J/HAX1-WT-mCherry/TRIM23/Merge.tif]

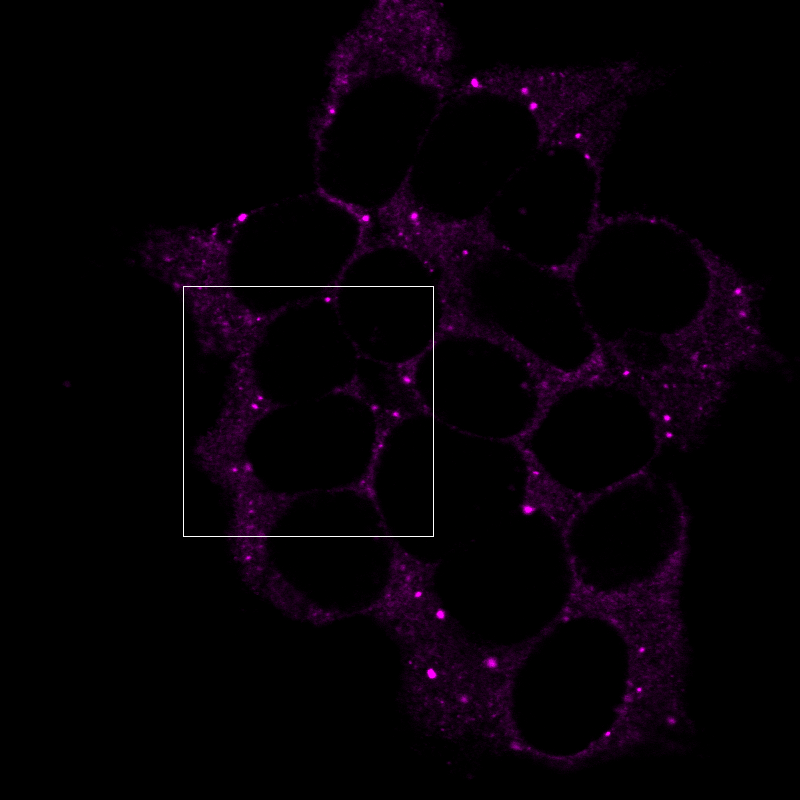

Supplement: Supplementary file 9 — Source data Fig. 6 [file 44318_2024_120_MOESM9_ESM.zip › Figure 6/6J/HAX1-WT-mCherry/TRIM23/HAX1.tif]

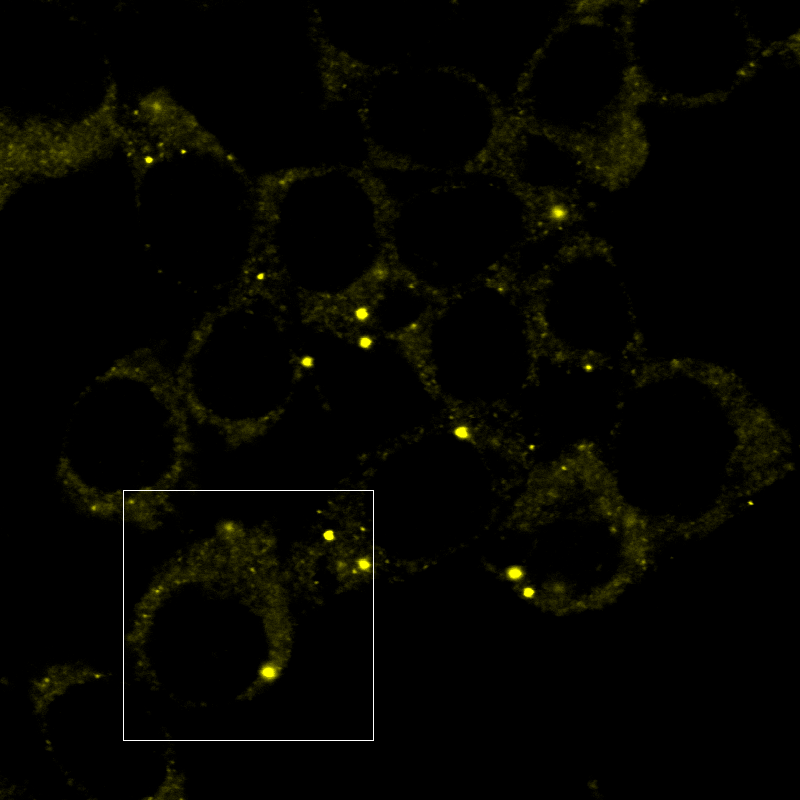

Supplement: Supplementary file 9 — Source data Fig. 6 [file 44318_2024_120_MOESM9_ESM.zip › Figure 6/6J/HAX1-WT-mCherry/Vector/LSM14A.tif]

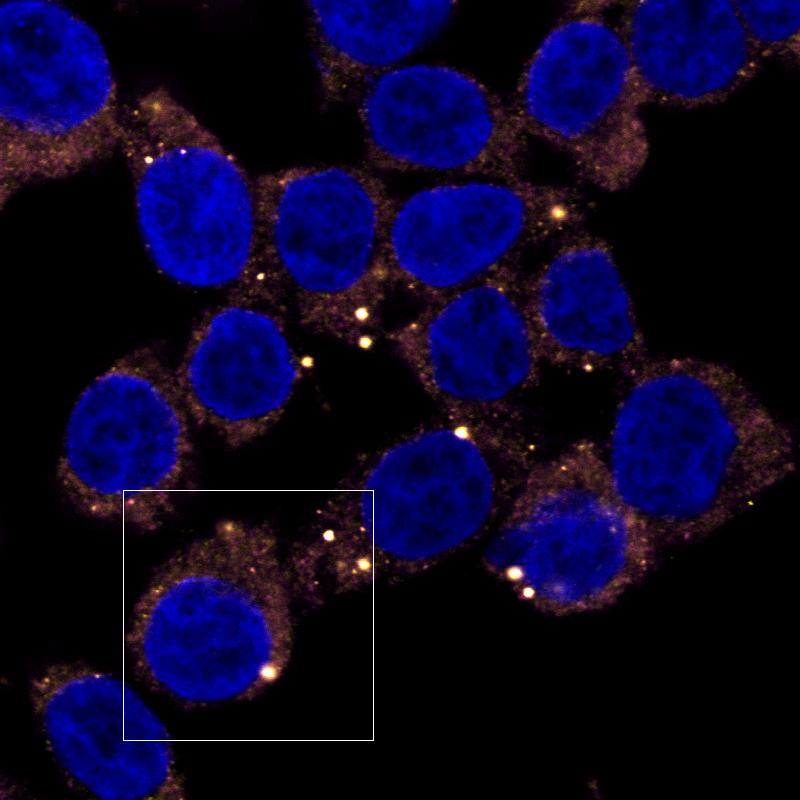

Supplement: Supplementary file 9 — Source data Fig. 6 [file 44318_2024_120_MOESM9_ESM.zip › Figure 6/6J/HAX1-WT-mCherry/Vector/Merge.tif]

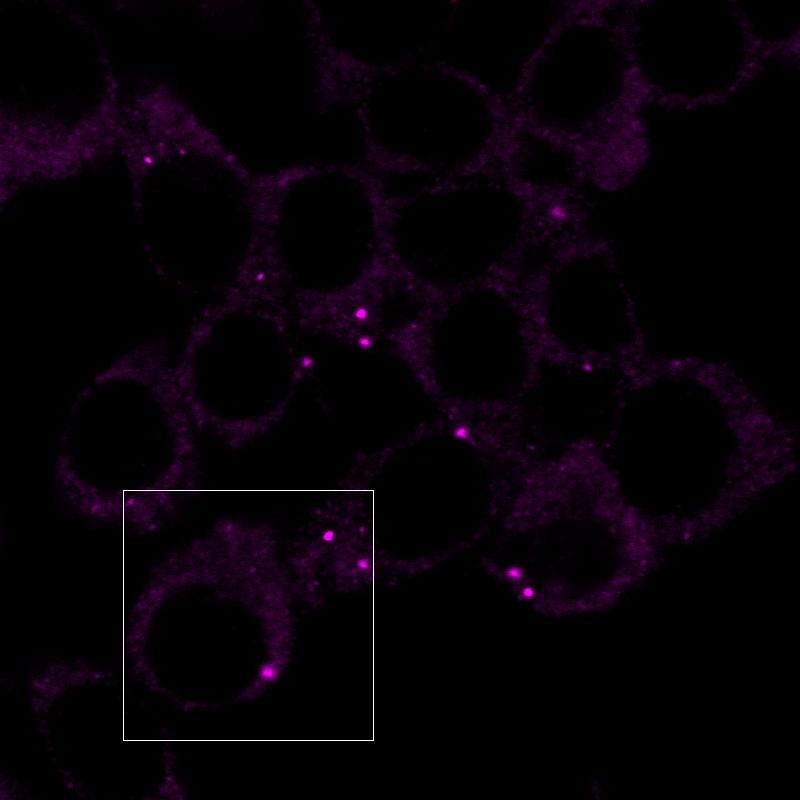

Supplement: Supplementary file 9 — Source data Fig. 6 [file 44318_2024_120_MOESM9_ESM.zip › Figure 6/6J/HAX1-WT-mCherry/Vector/HAX1.tif]

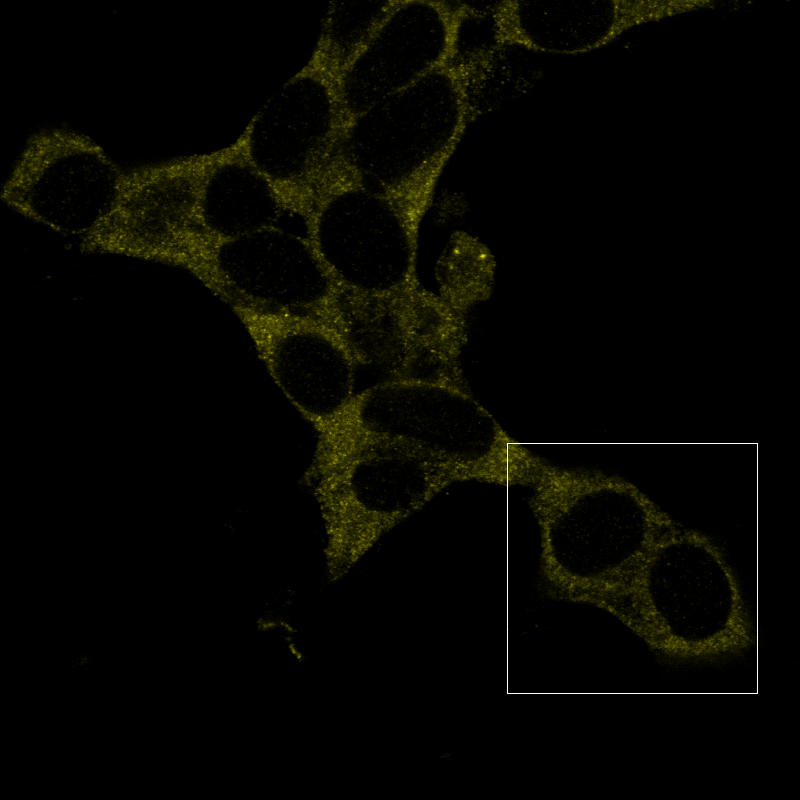

Supplement: Supplementary file 9 — Source data Fig. 6 [file 44318_2024_120_MOESM9_ESM.zip › Figure 6/6J/HAX1-K131R-mCherry/TRIM23/LSM14A.tif]

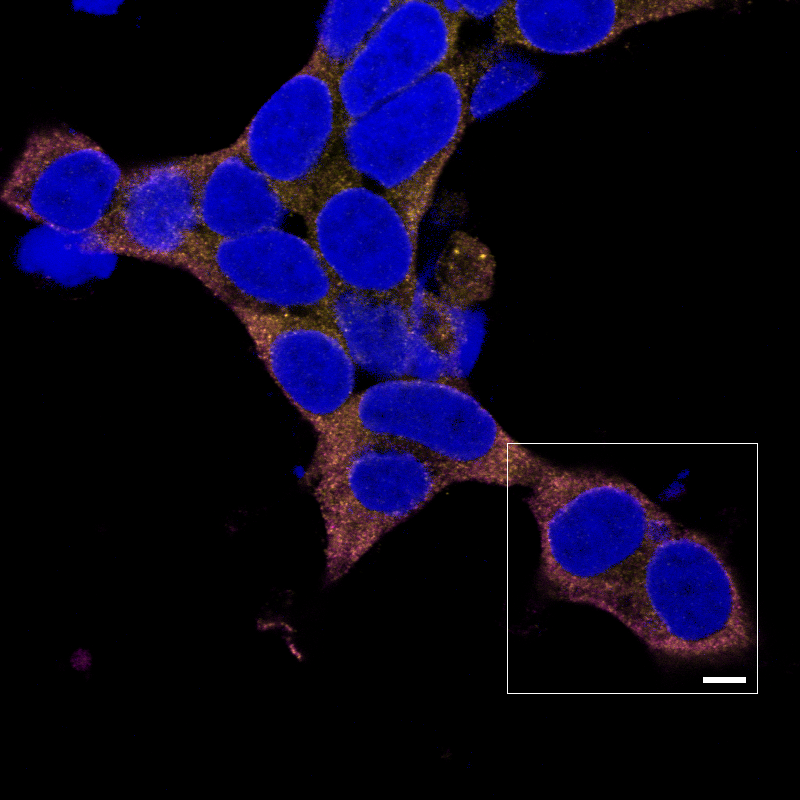

Supplement: Supplementary file 9 — Source data Fig. 6 [file 44318_2024_120_MOESM9_ESM.zip › Figure 6/6J/HAX1-K131R-mCherry/TRIM23/Merge.tif]

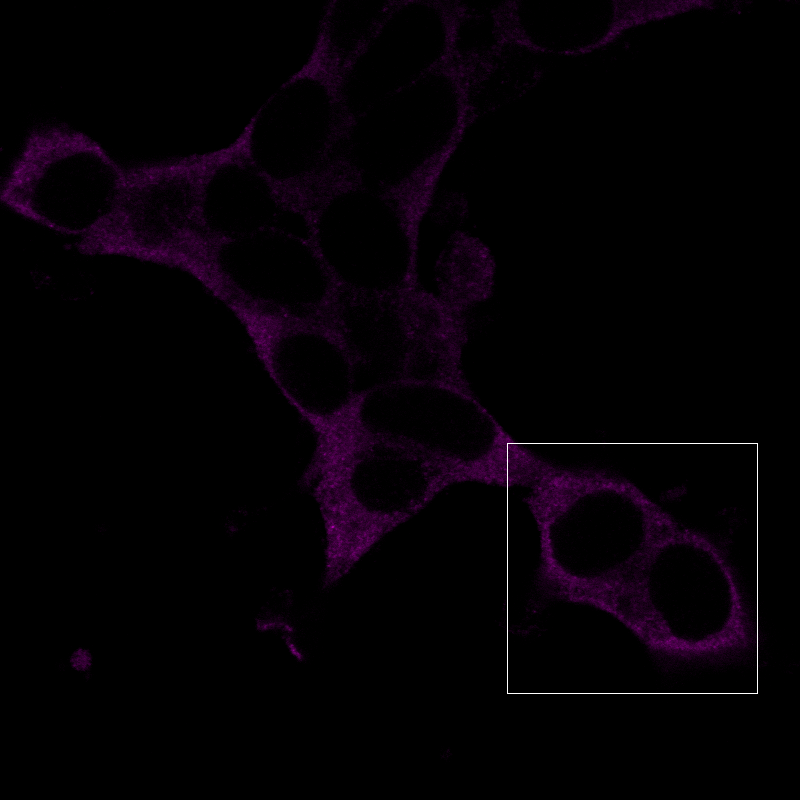

Supplement: Supplementary file 9 — Source data Fig. 6 [file 44318_2024_120_MOESM9_ESM.zip › Figure 6/6J/HAX1-K131R-mCherry/TRIM23/HAX1.tif]

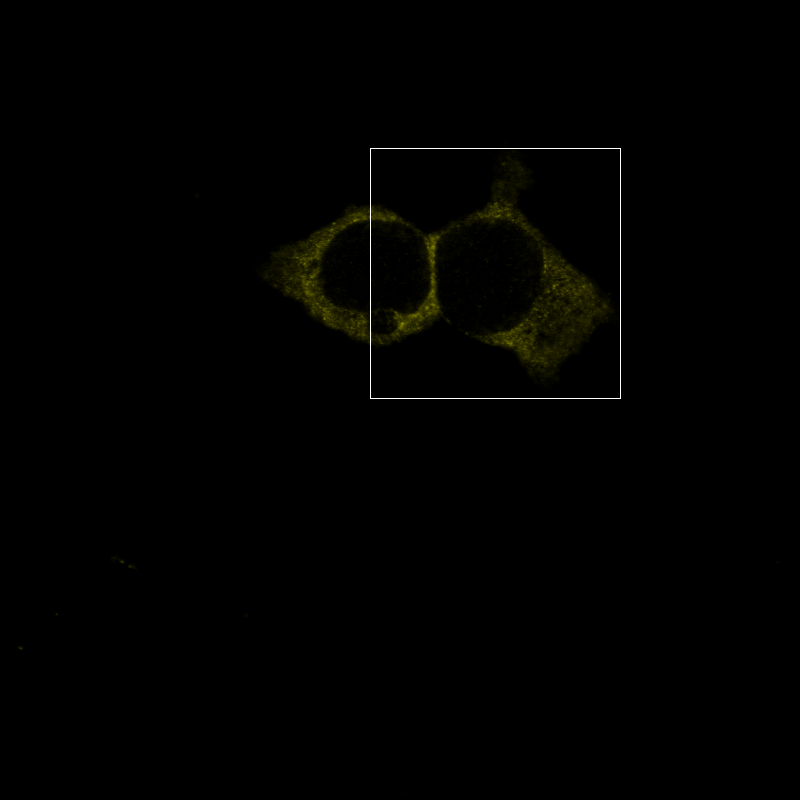

Supplement: Supplementary file 9 — Source data Fig. 6 [file 44318_2024_120_MOESM9_ESM.zip › Figure 6/6J/HAX1-K131R-mCherry/Vector/LSM14A.tif]

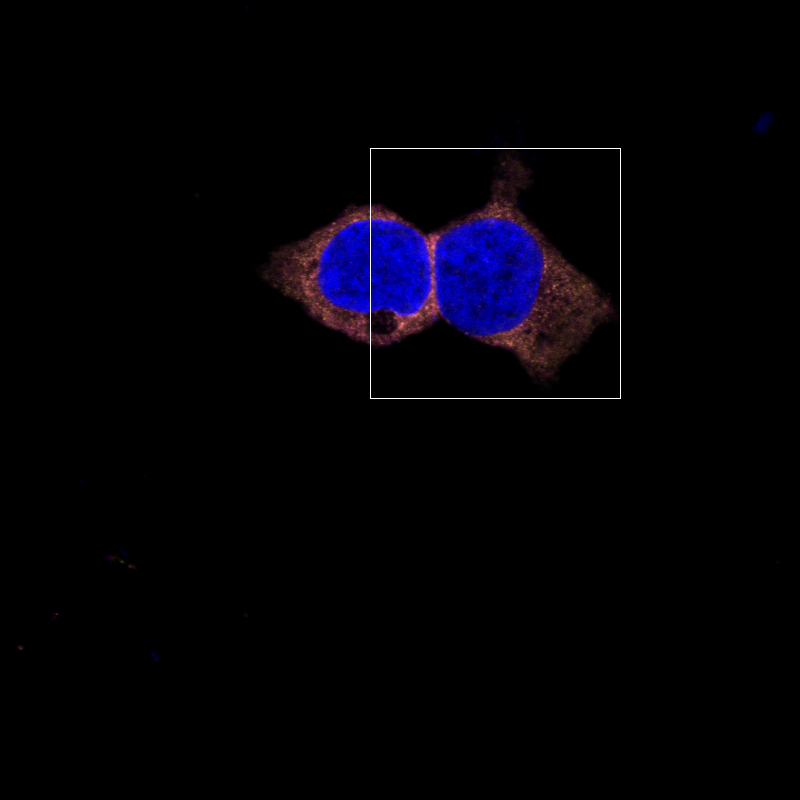

Supplement: Supplementary file 9 — Source data Fig. 6 [file 44318_2024_120_MOESM9_ESM.zip › Figure 6/6J/HAX1-K131R-mCherry/Vector/Merge.tif]

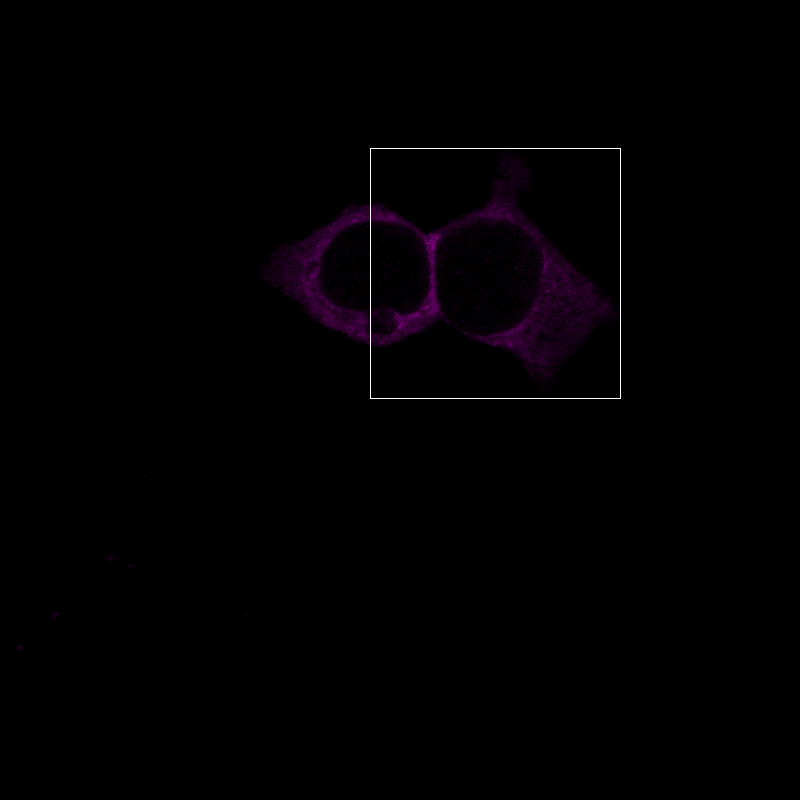

Supplement: Supplementary file 9 — Source data Fig. 6 [file 44318_2024_120_MOESM9_ESM.zip › Figure 6/6J/HAX1-K131R-mCherry/Vector/HAX1.tif]

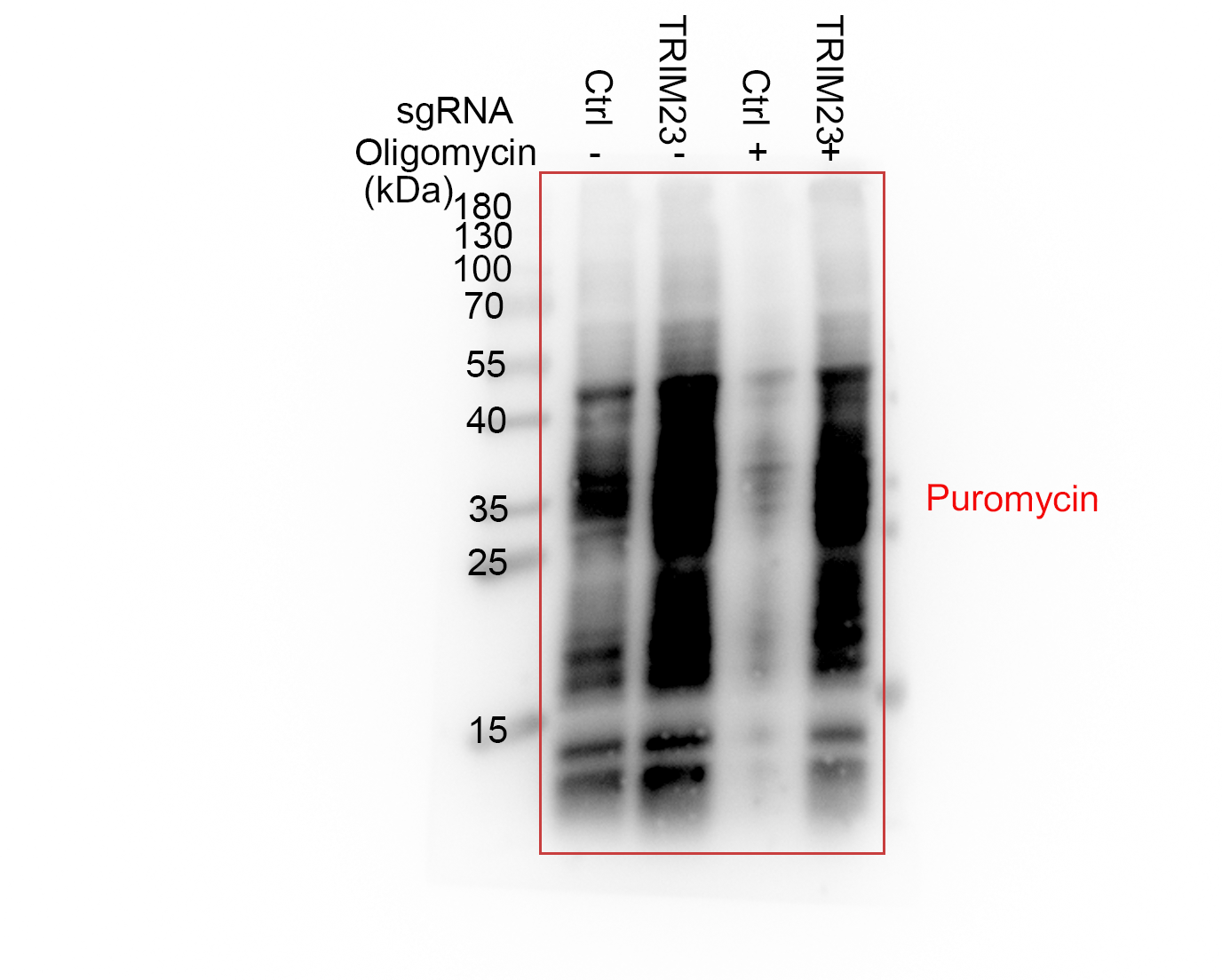

Supplement: Supplementary file 10 — Source data Fig. 7 [file 44318_2024_120_MOESM10_ESM.zip › Figure 7/7D/western-Puromycin.Tif]

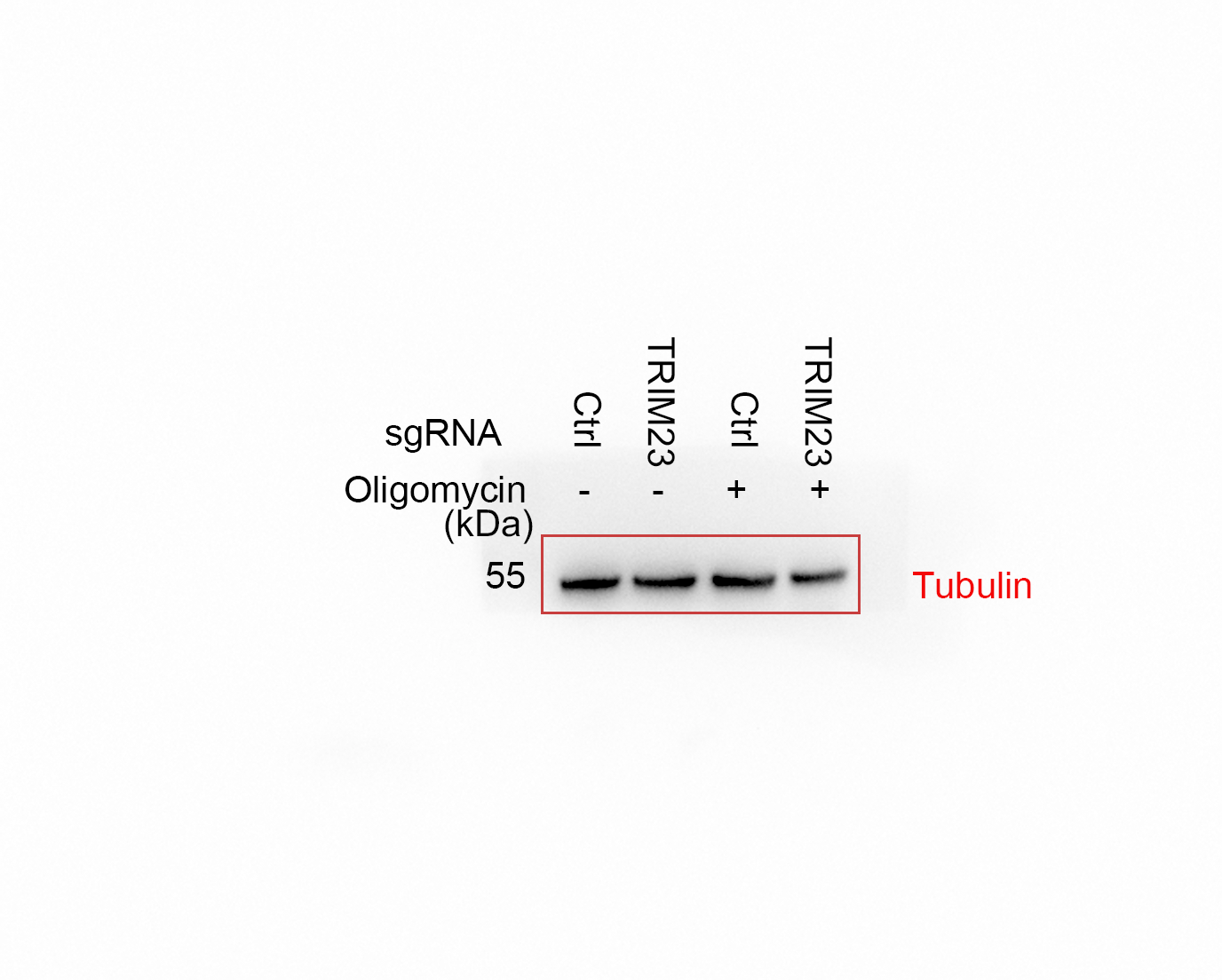

Supplement: Supplementary file 10 — Source data Fig. 7 [file 44318_2024_120_MOESM10_ESM.zip › Figure 7/7D/western-Tubulin.Tif]

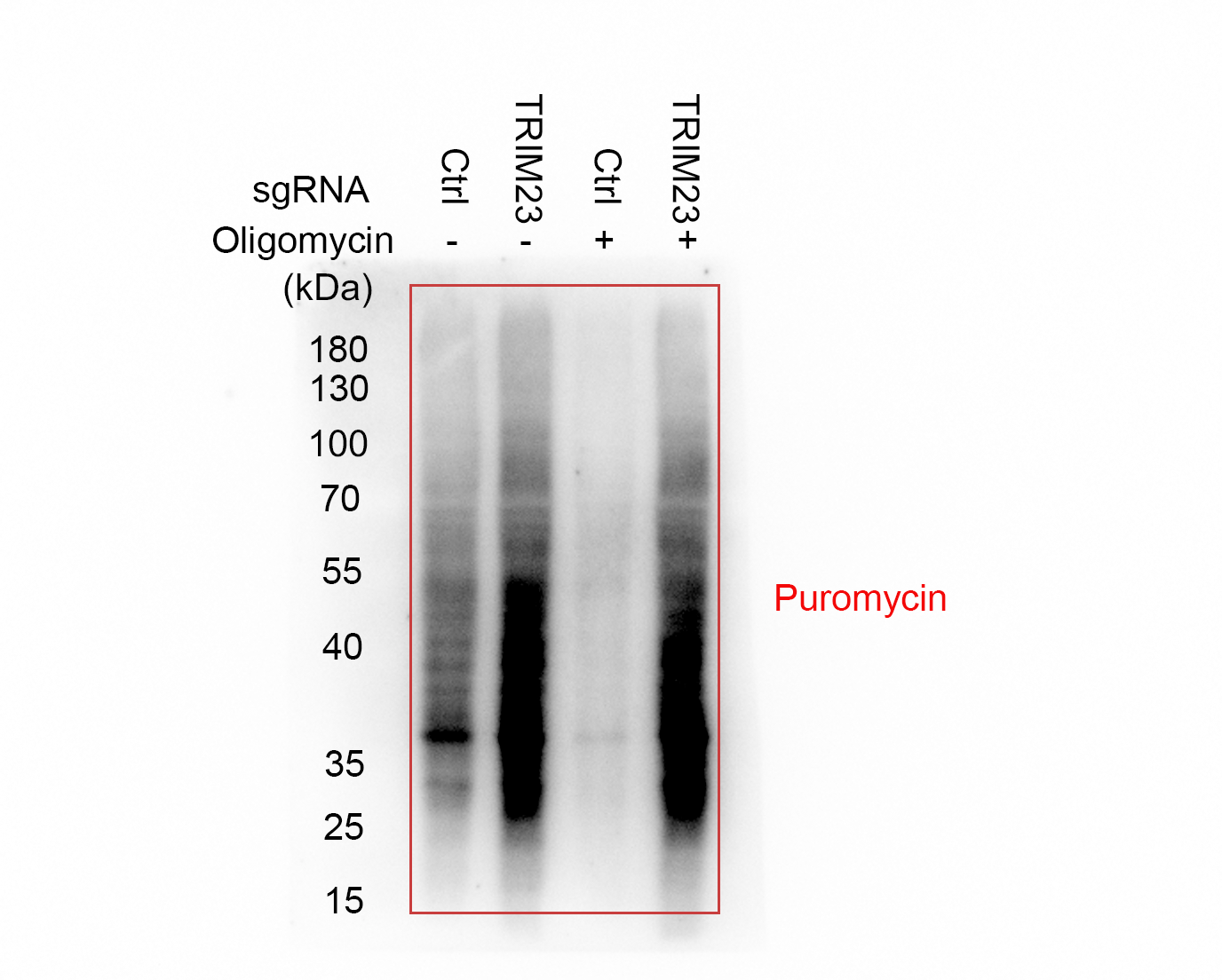

Supplement: Supplementary file 10 — Source data Fig. 7 [file 44318_2024_120_MOESM10_ESM.zip › Figure 7/7C/western-Puromycin.Tif]

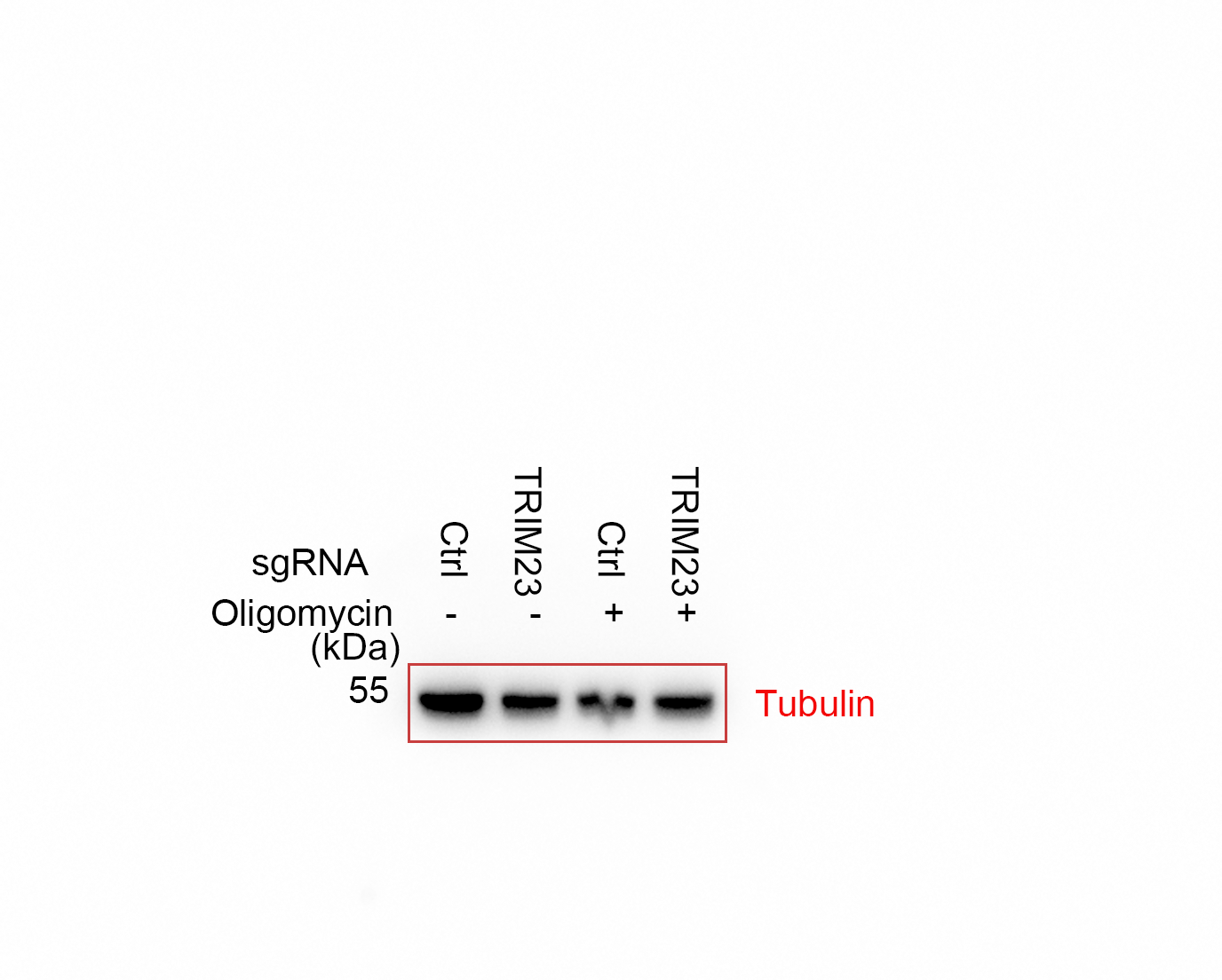

Supplement: Supplementary file 10 — Source data Fig. 7 [file 44318_2024_120_MOESM10_ESM.zip › Figure 7/7C/western-Tubulin.Tif]

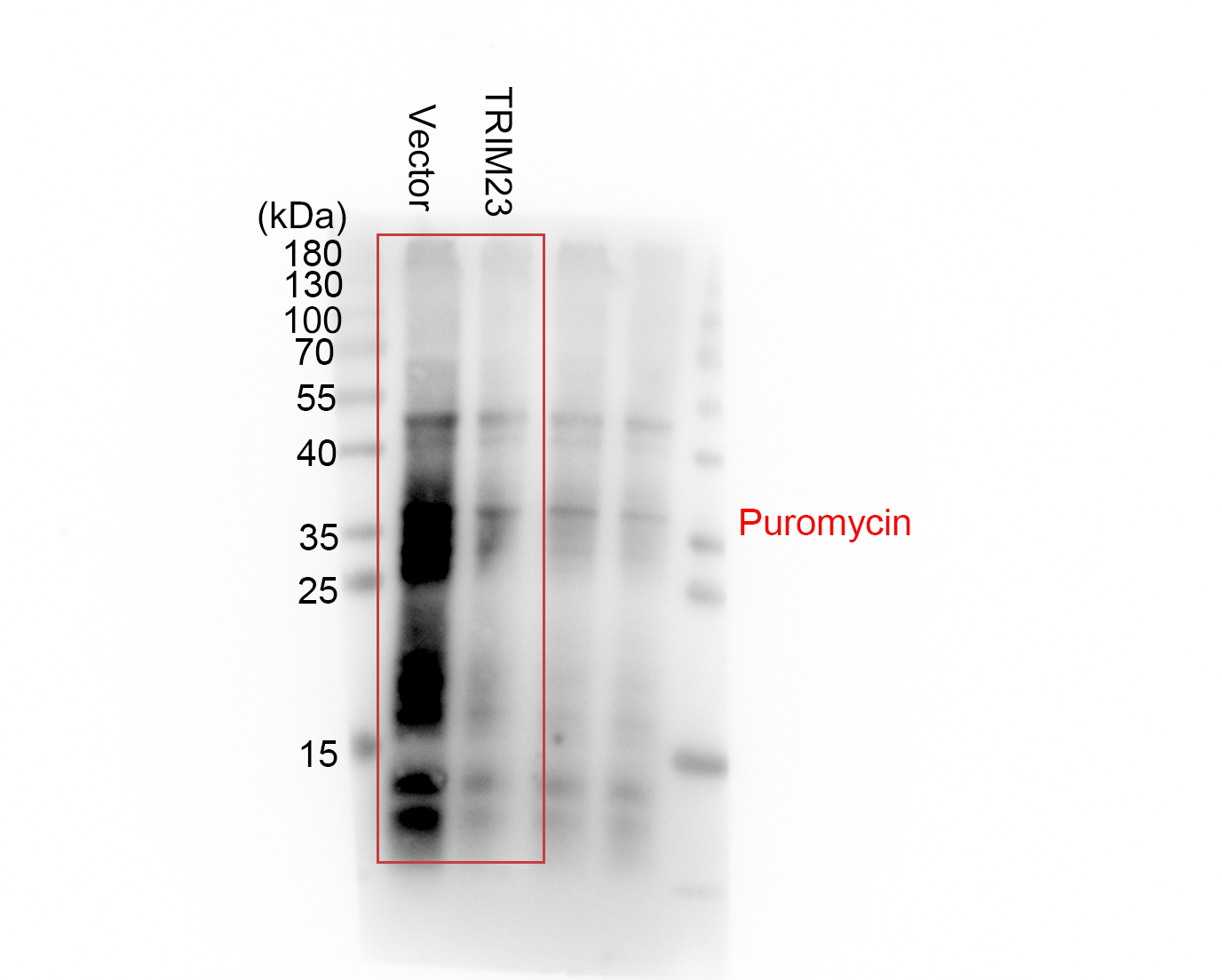

Supplement: Supplementary file 10 — Source data Fig. 7 [file 44318_2024_120_MOESM10_ESM.zip › Figure 7/7B/western-Puromycin.Tif]

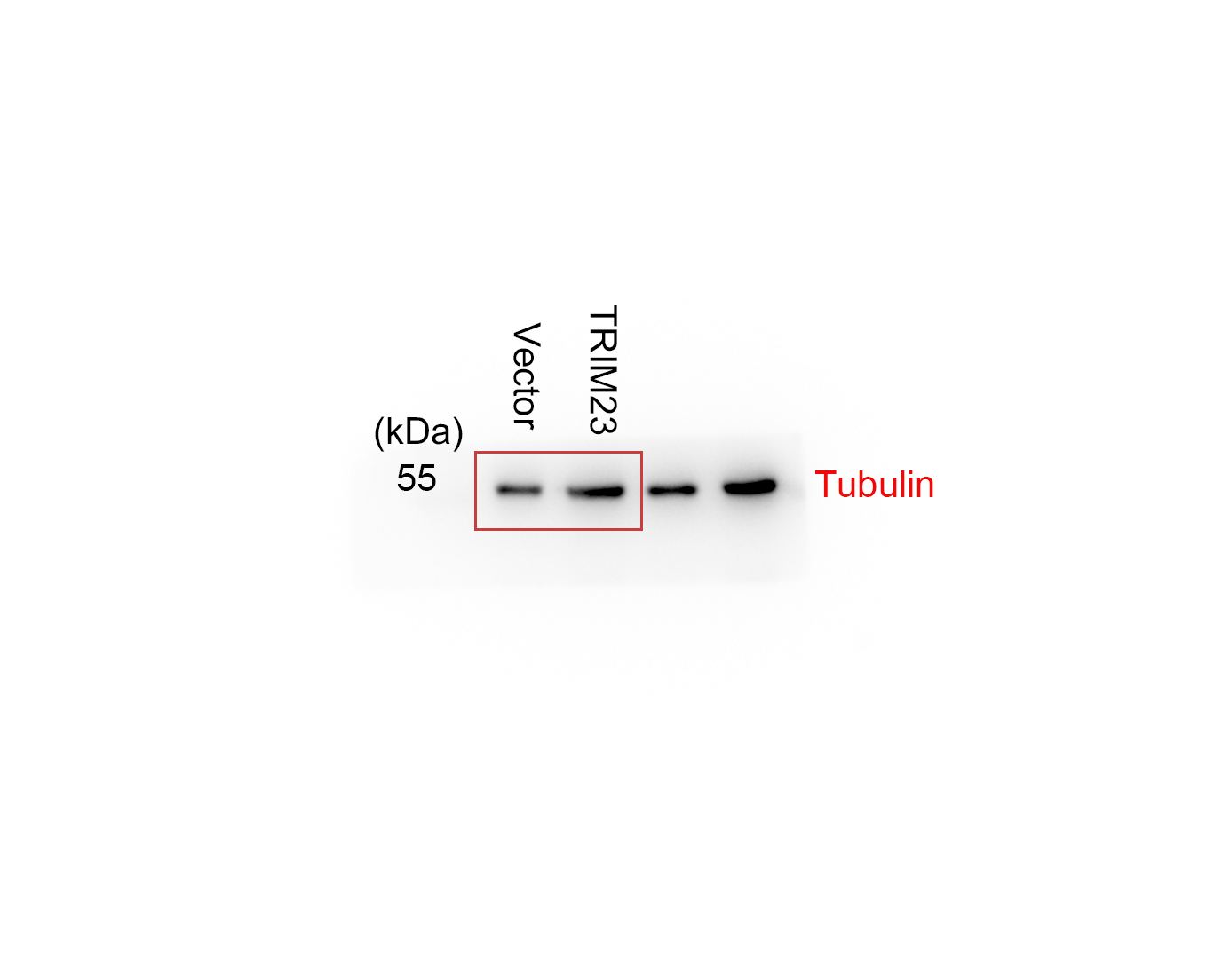

Supplement: Supplementary file 10 — Source data Fig. 7 [file 44318_2024_120_MOESM10_ESM.zip › Figure 7/7B/western-Tubulin.Tif]

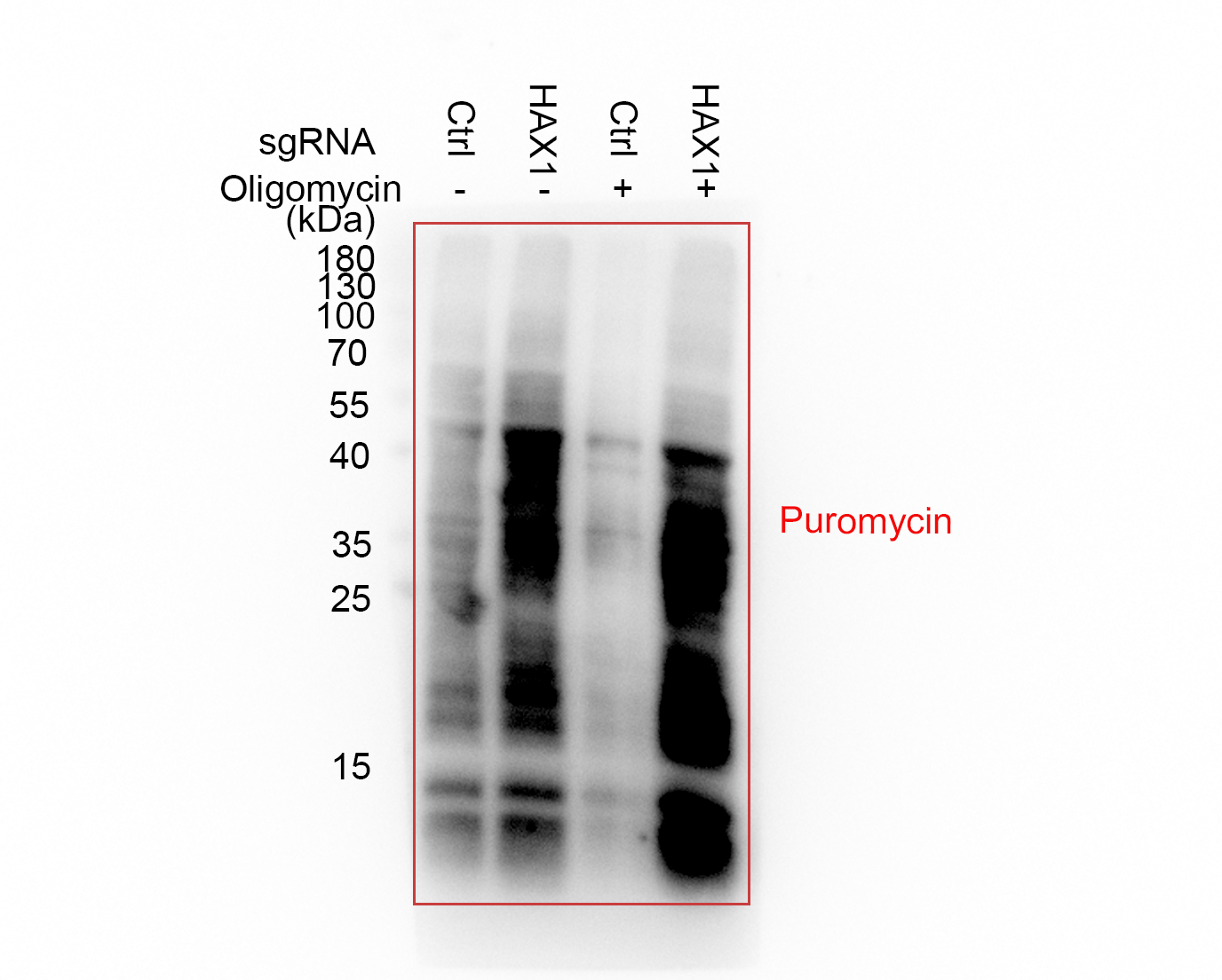

Supplement: Supplementary file 10 — Source data Fig. 7 [file 44318_2024_120_MOESM10_ESM.zip › Figure 7/7E/western-Puromycin.Tif]

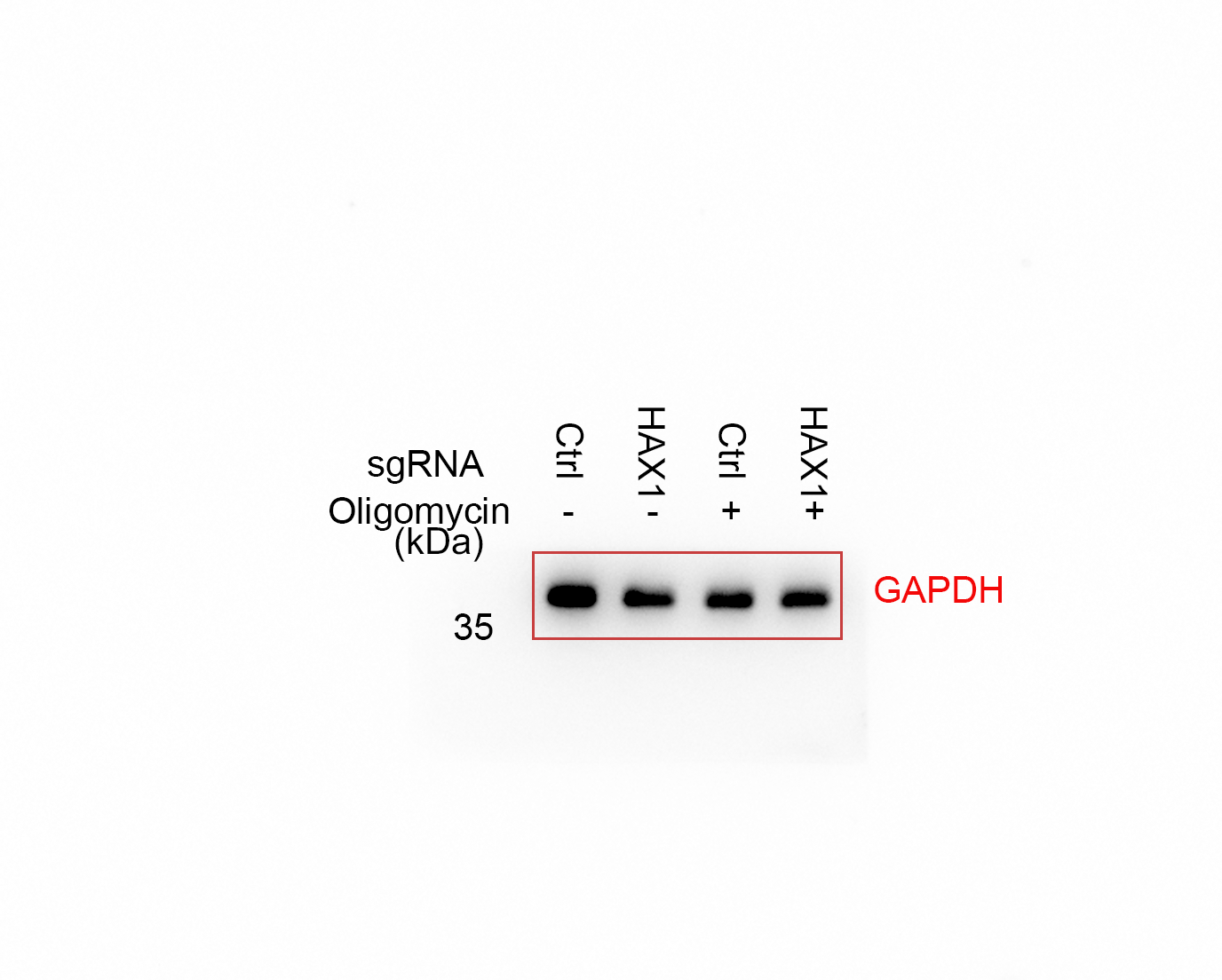

Supplement: Supplementary file 10 — Source data Fig. 7 [file 44318_2024_120_MOESM10_ESM.zip › Figure 7/7E/western-GAPDH.Tif]

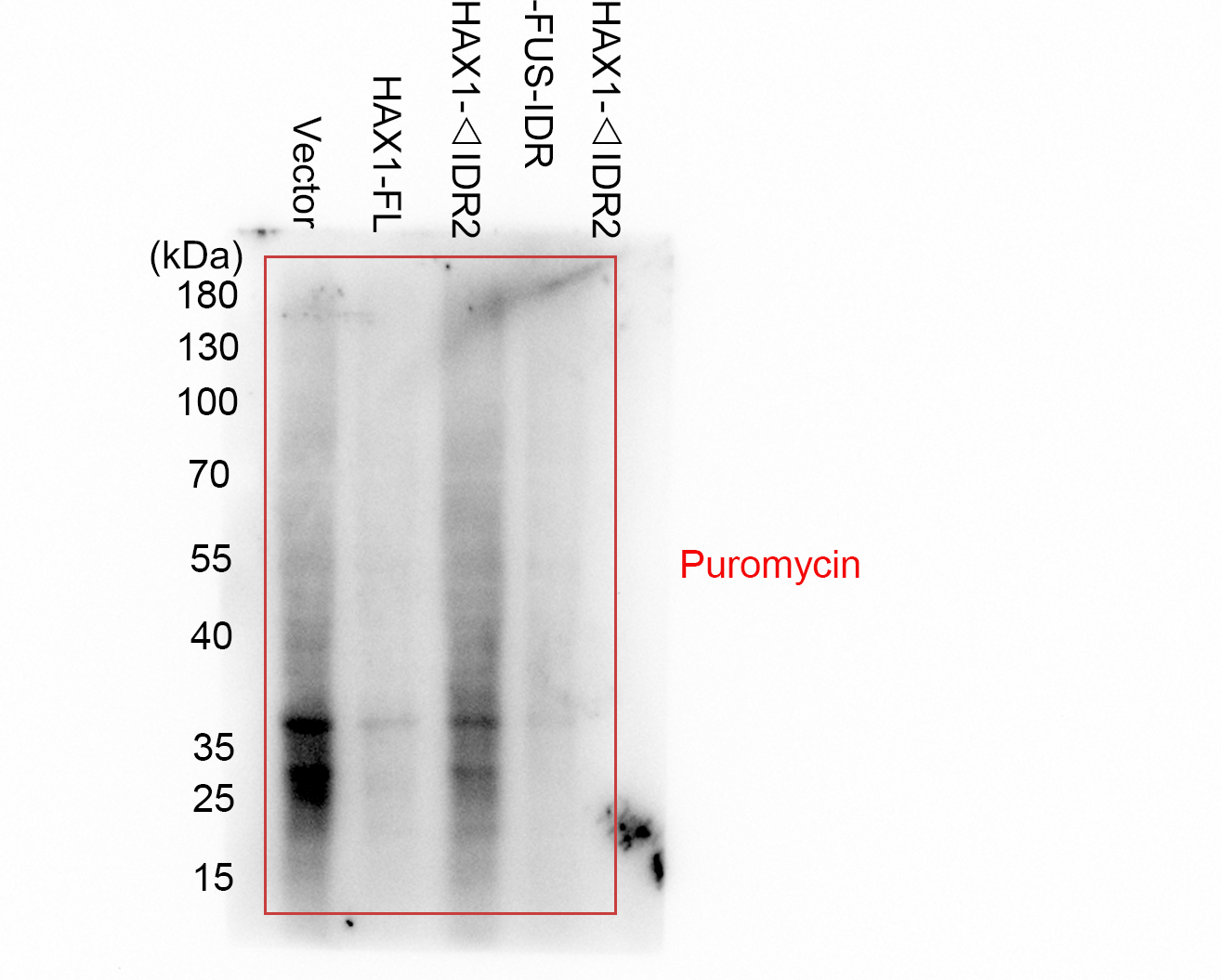

Supplement: Supplementary file 10 — Source data Fig. 7 [file 44318_2024_120_MOESM10_ESM.zip › Figure 7/7G/western-Puromycin.Tif]

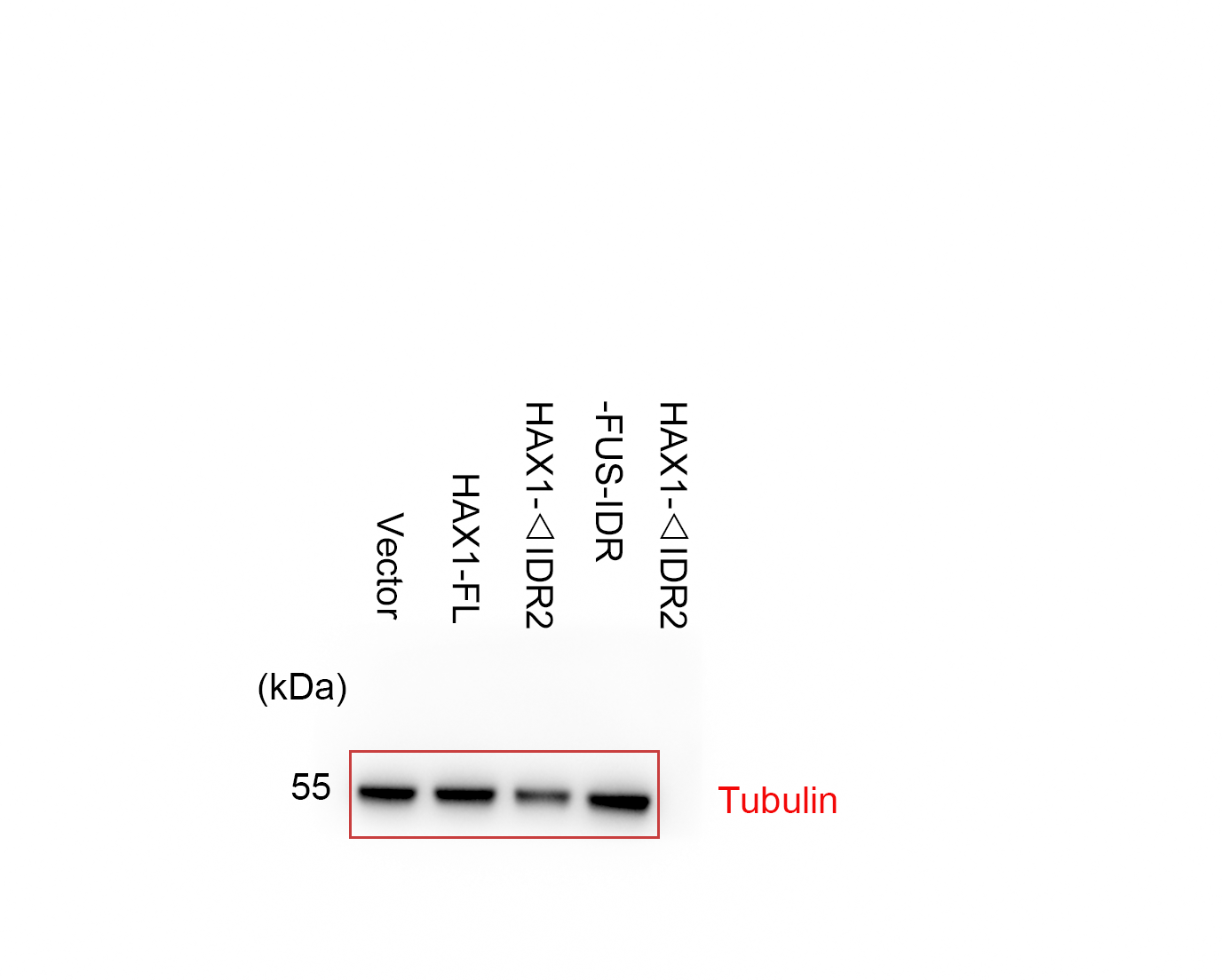

Supplement: Supplementary file 10 — Source data Fig. 7 [file 44318_2024_120_MOESM10_ESM.zip › Figure 7/7G/western-Tubulin.Tif]

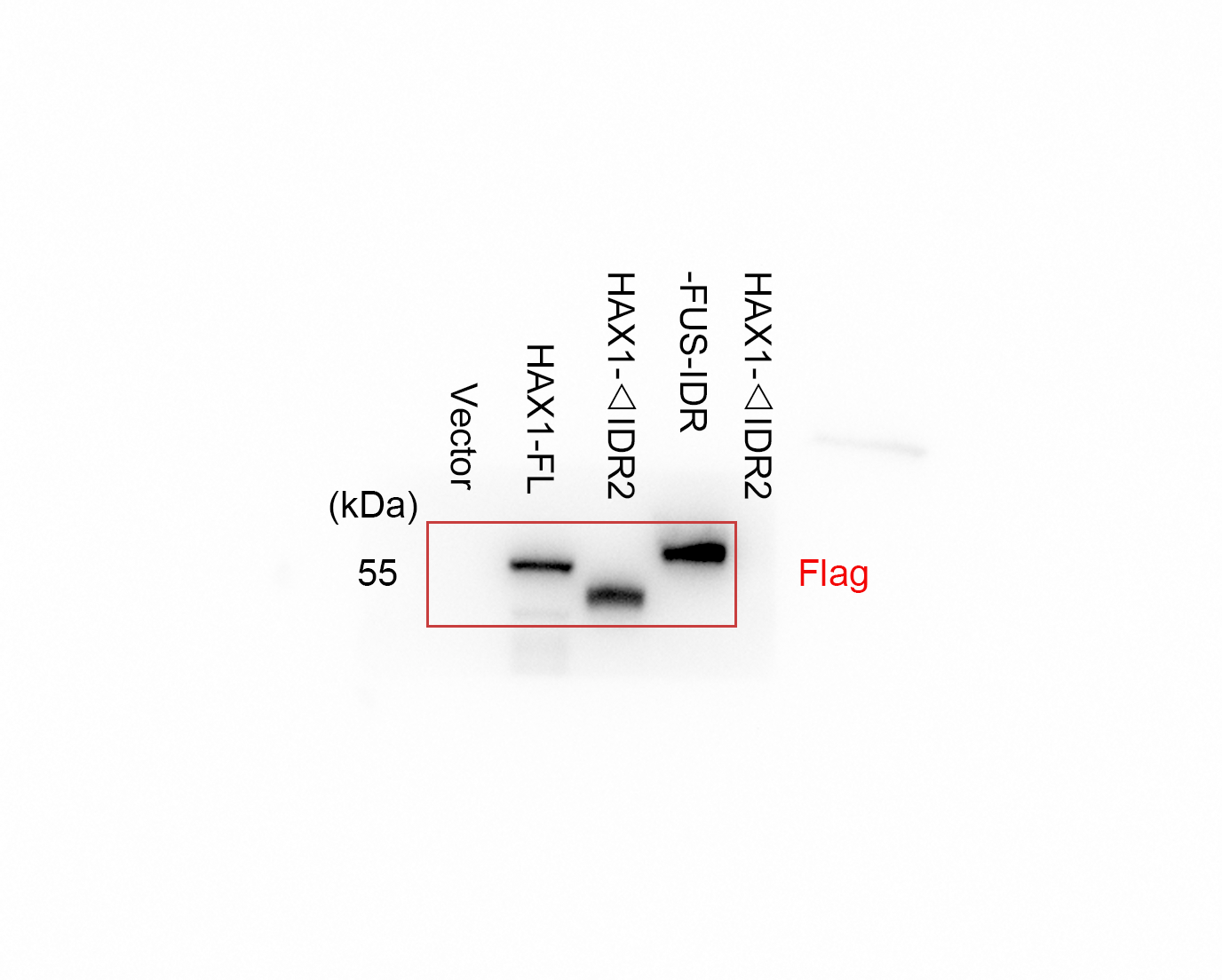

Supplement: Supplementary file 10 — Source data Fig. 7 [file 44318_2024_120_MOESM10_ESM.zip › Figure 7/7G/western-Flag.Tif]

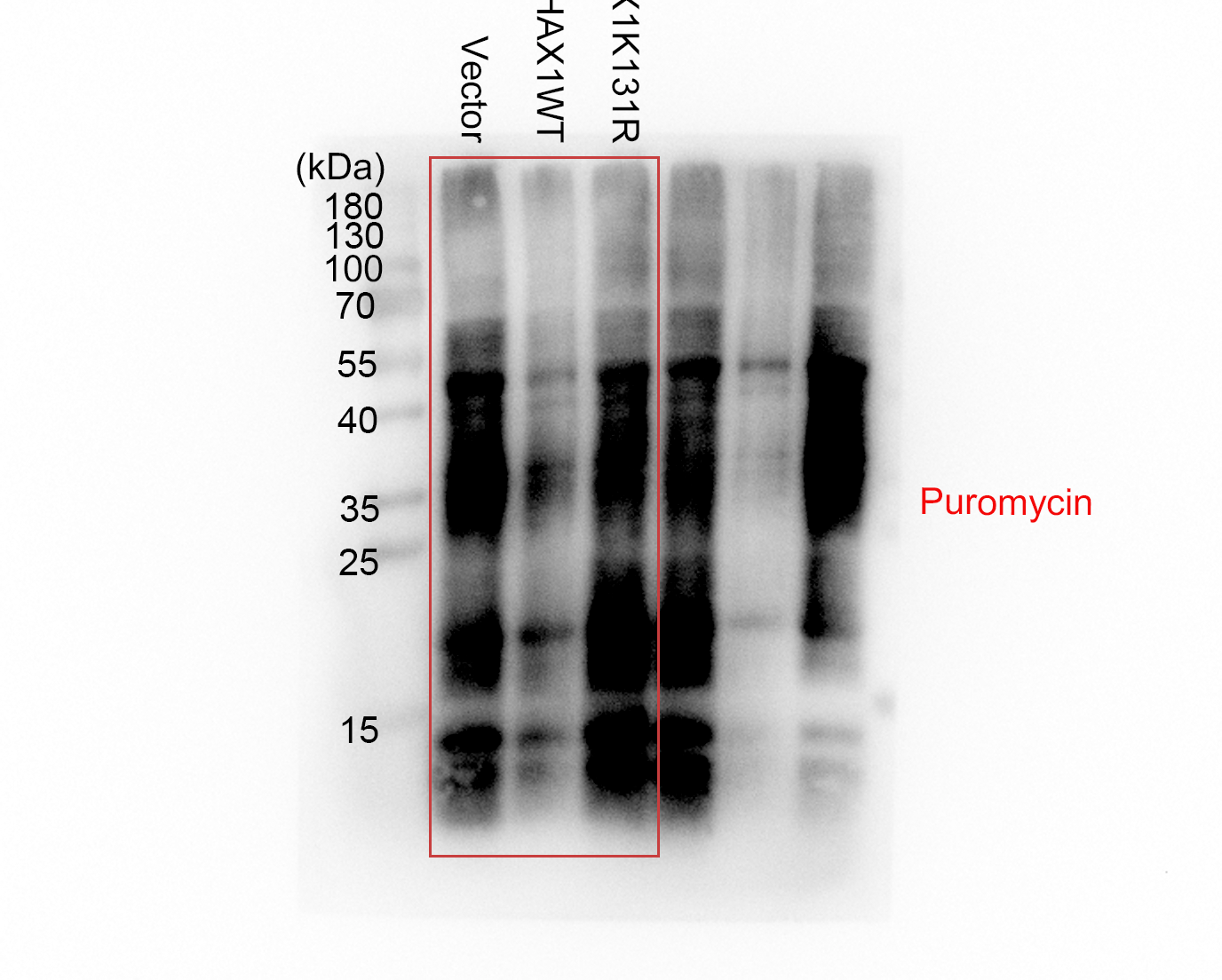

Supplement: Supplementary file 10 — Source data Fig. 7 [file 44318_2024_120_MOESM10_ESM.zip › Figure 7/7F/western-Puromycin.Tif]

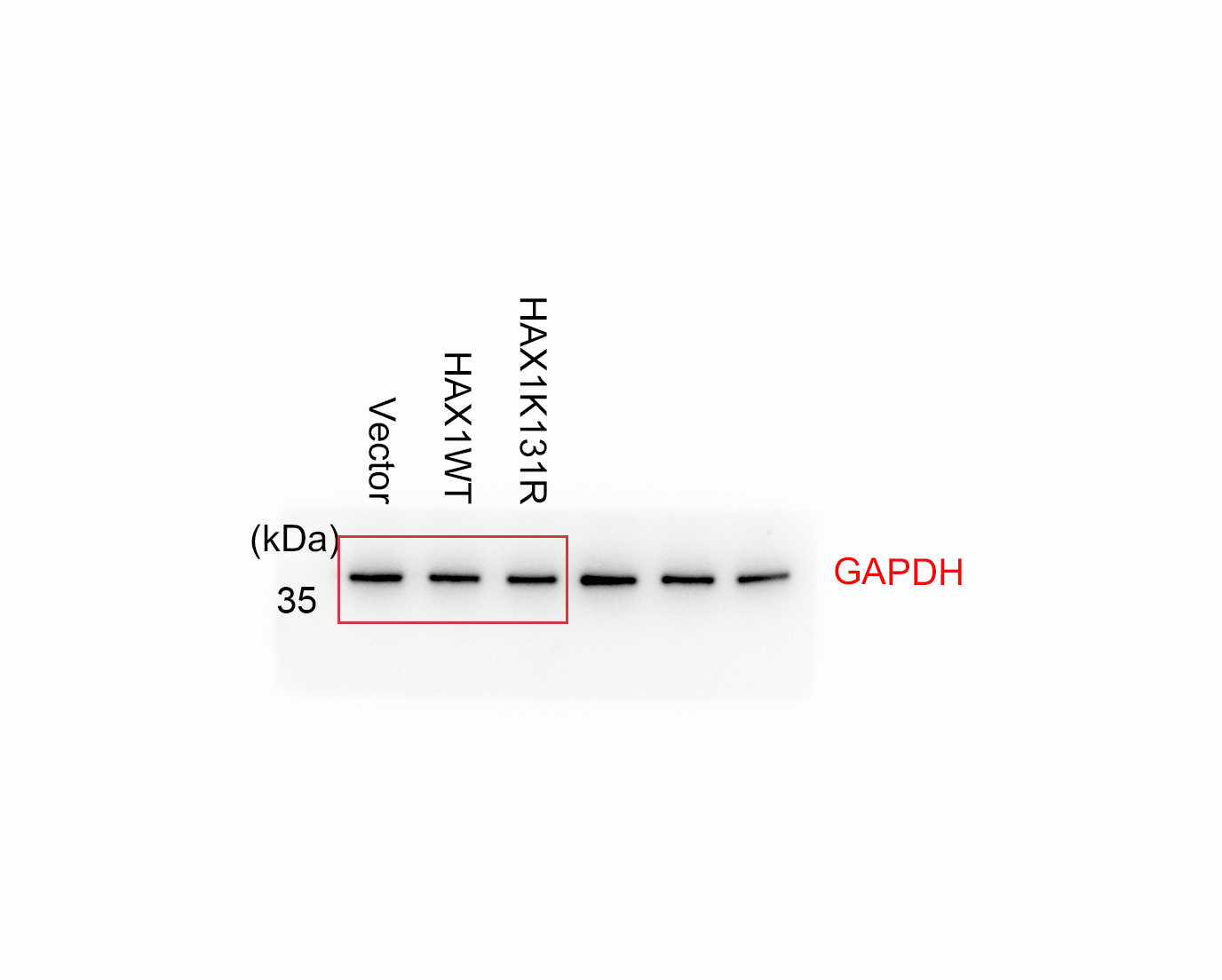

Supplement: Supplementary file 10 — Source data Fig. 7 [file 44318_2024_120_MOESM10_ESM.zip › Figure 7/7F/western-GAPDH.Tif]

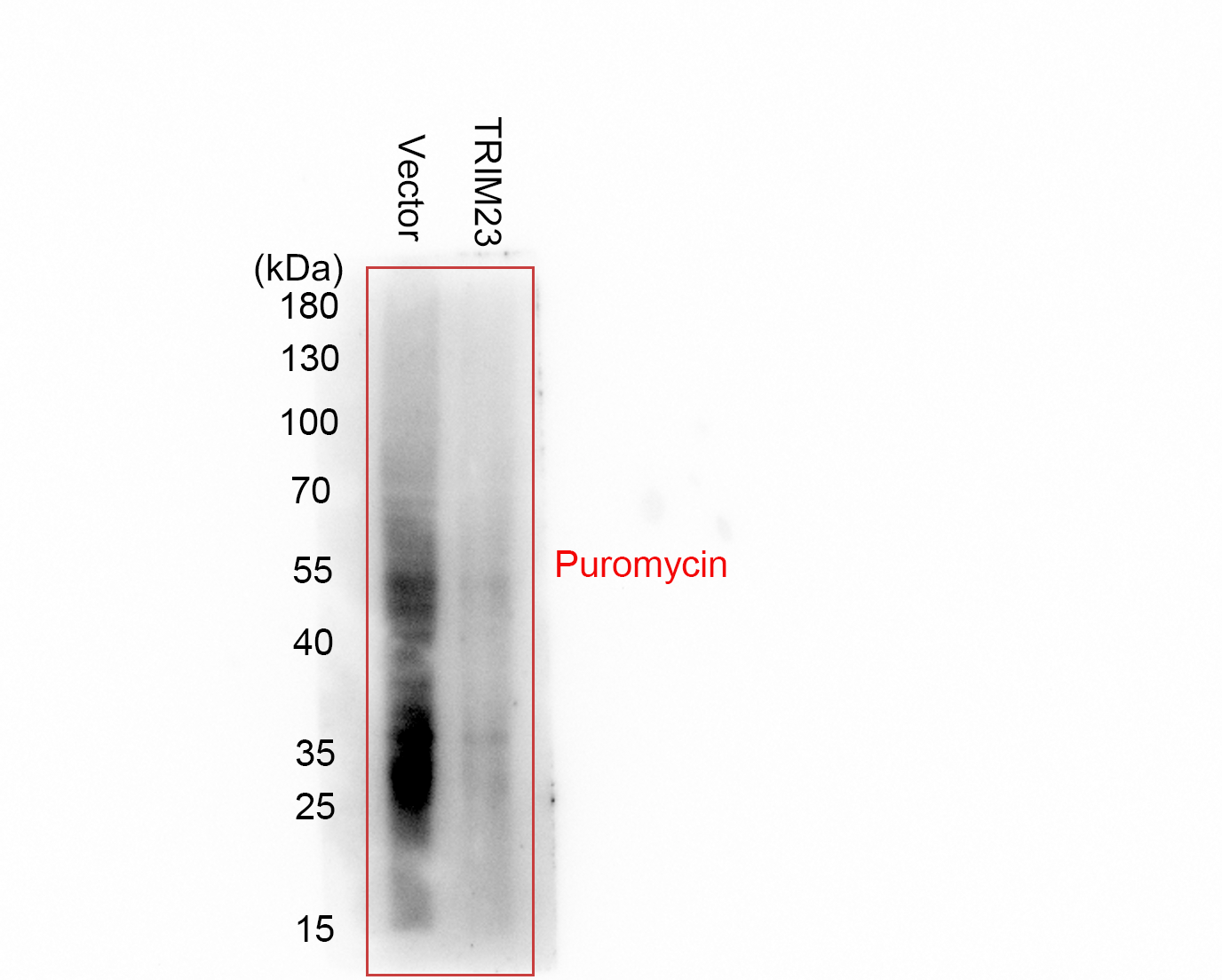

Supplement: Supplementary file 10 — Source data Fig. 7 [file 44318_2024_120_MOESM10_ESM.zip › Figure 7/7A/western-Puromycin.Tif]

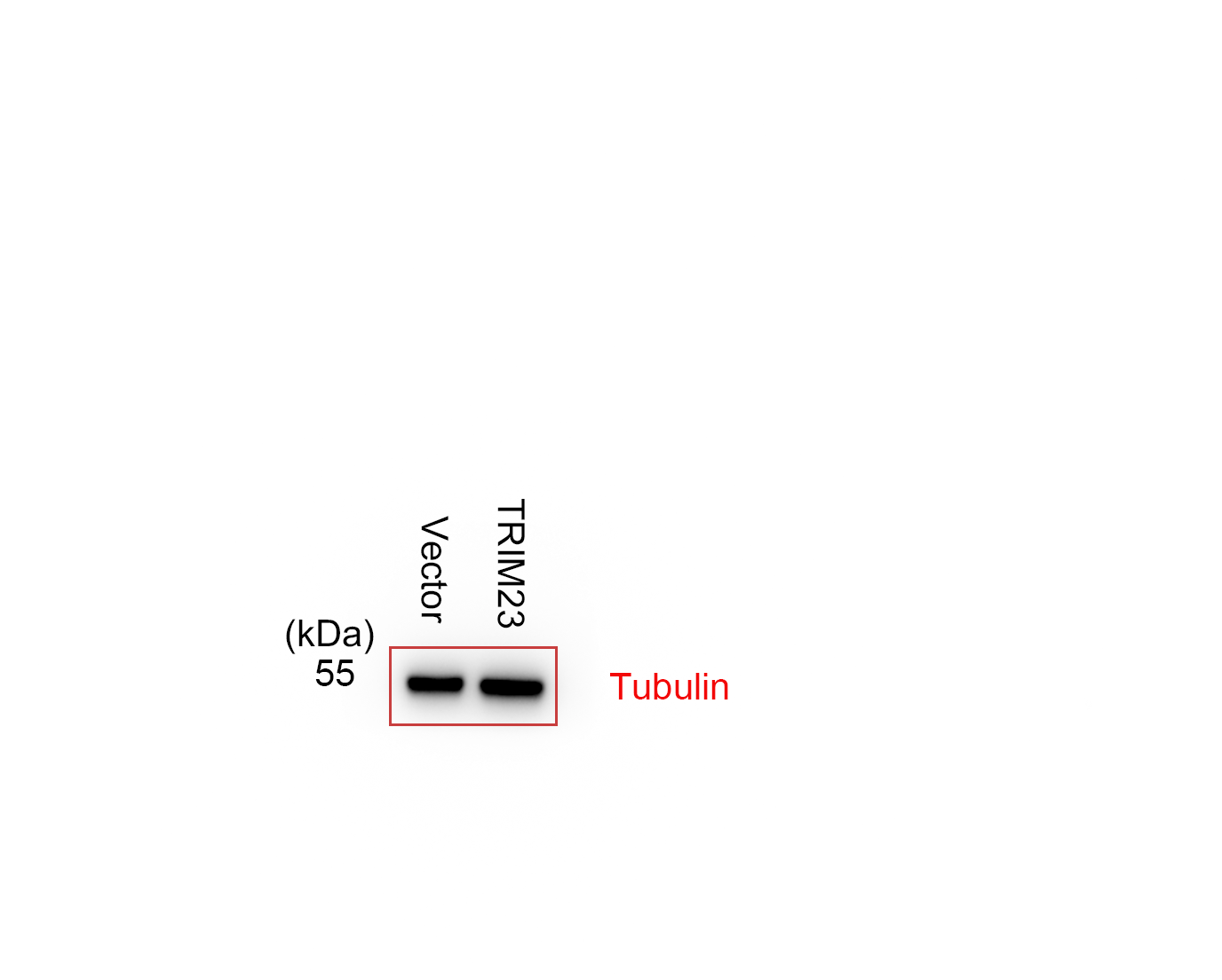

Supplement: Supplementary file 10 — Source data Fig. 7 [file 44318_2024_120_MOESM10_ESM.zip › Figure 7/7A/western-Tubulin.Tif]

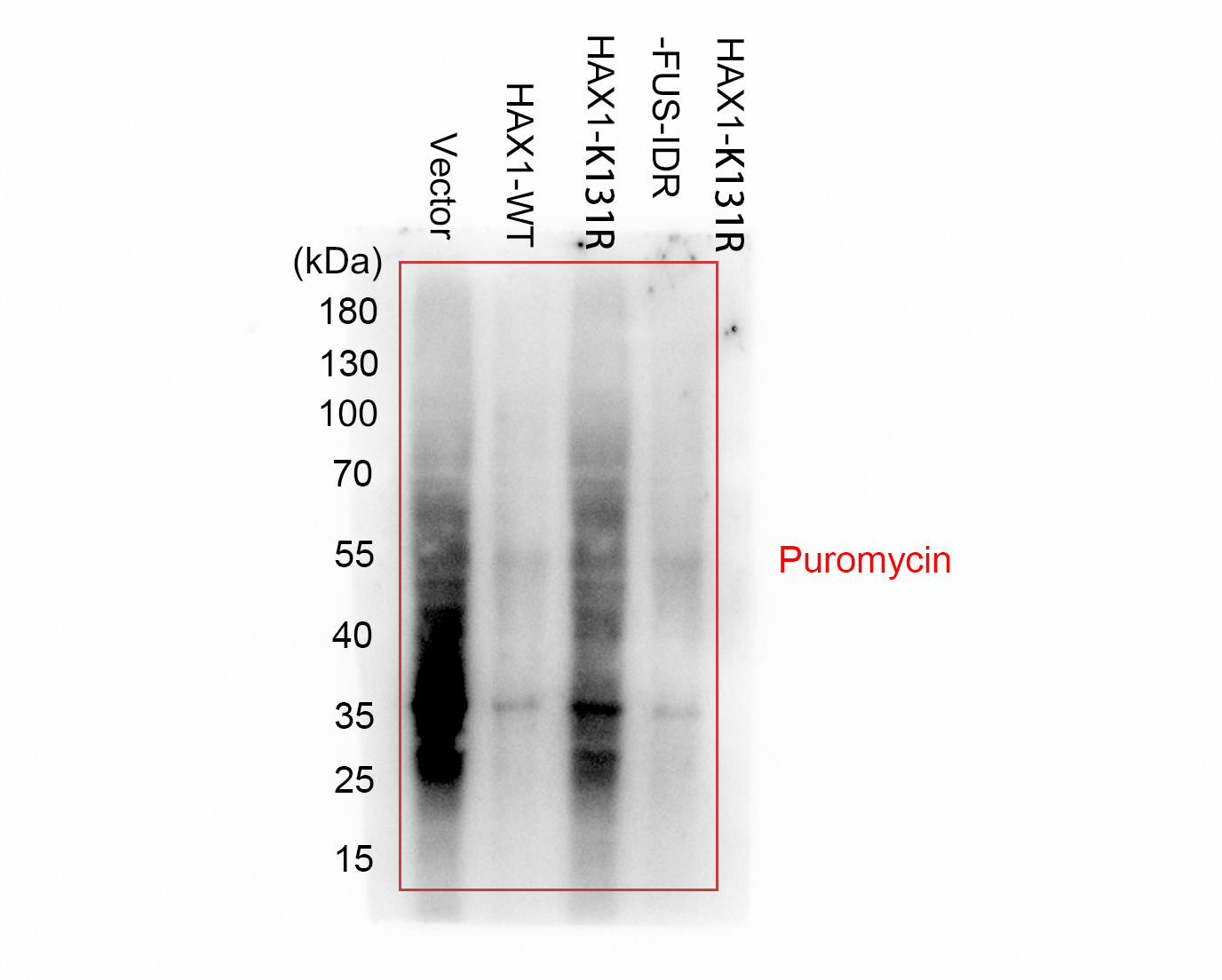

Supplement: Supplementary file 10 — Source data Fig. 7 [file 44318_2024_120_MOESM10_ESM.zip › Figure 7/7H/western-Puromycin.Tif]

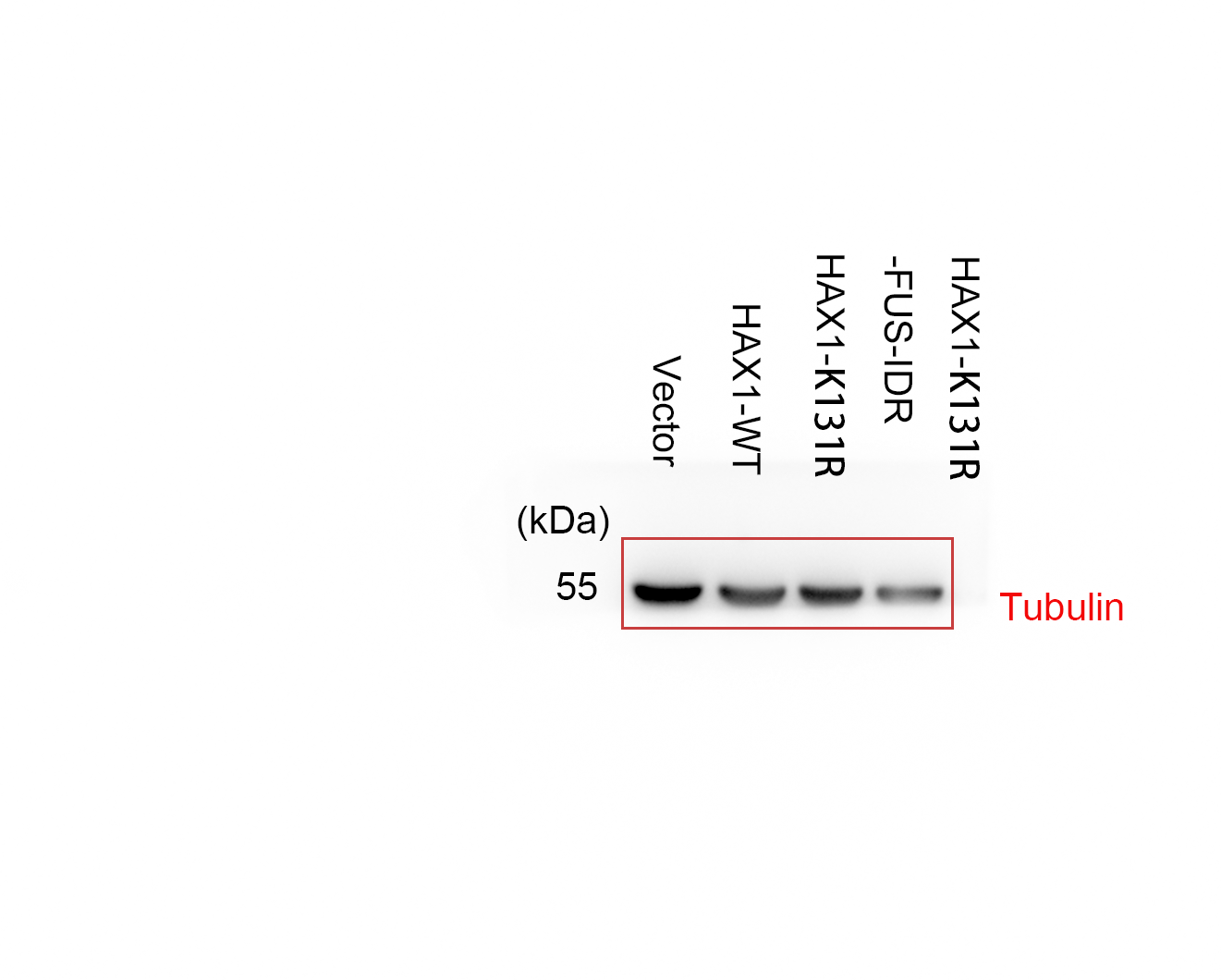

Supplement: Supplementary file 10 — Source data Fig. 7 [file 44318_2024_120_MOESM10_ESM.zip › Figure 7/7H/western-TUBULIN.Tif]

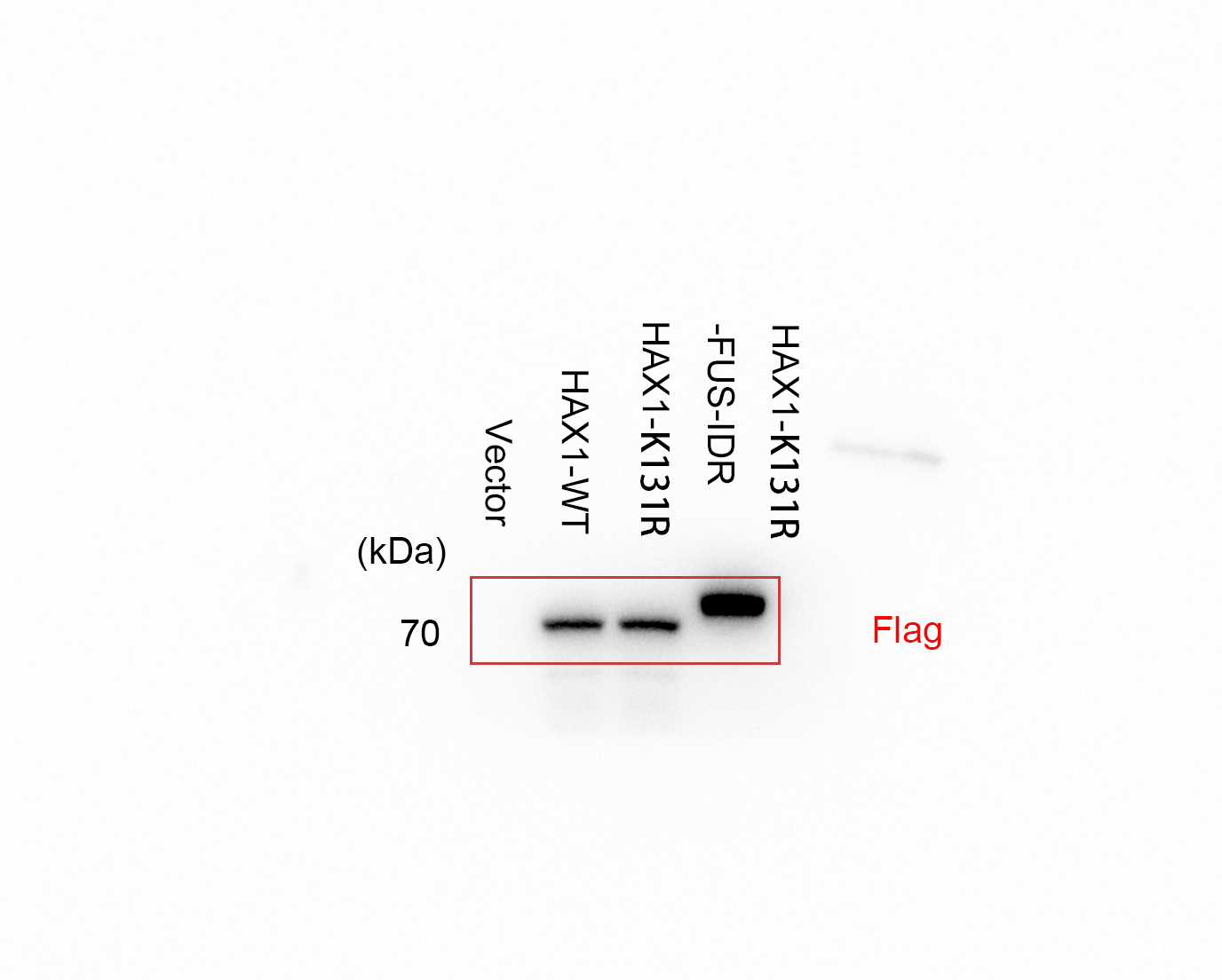

Supplement: Supplementary file 10 — Source data Fig. 7 [file 44318_2024_120_MOESM10_ESM.zip › Figure 7/7H/western-Flag.Tif]

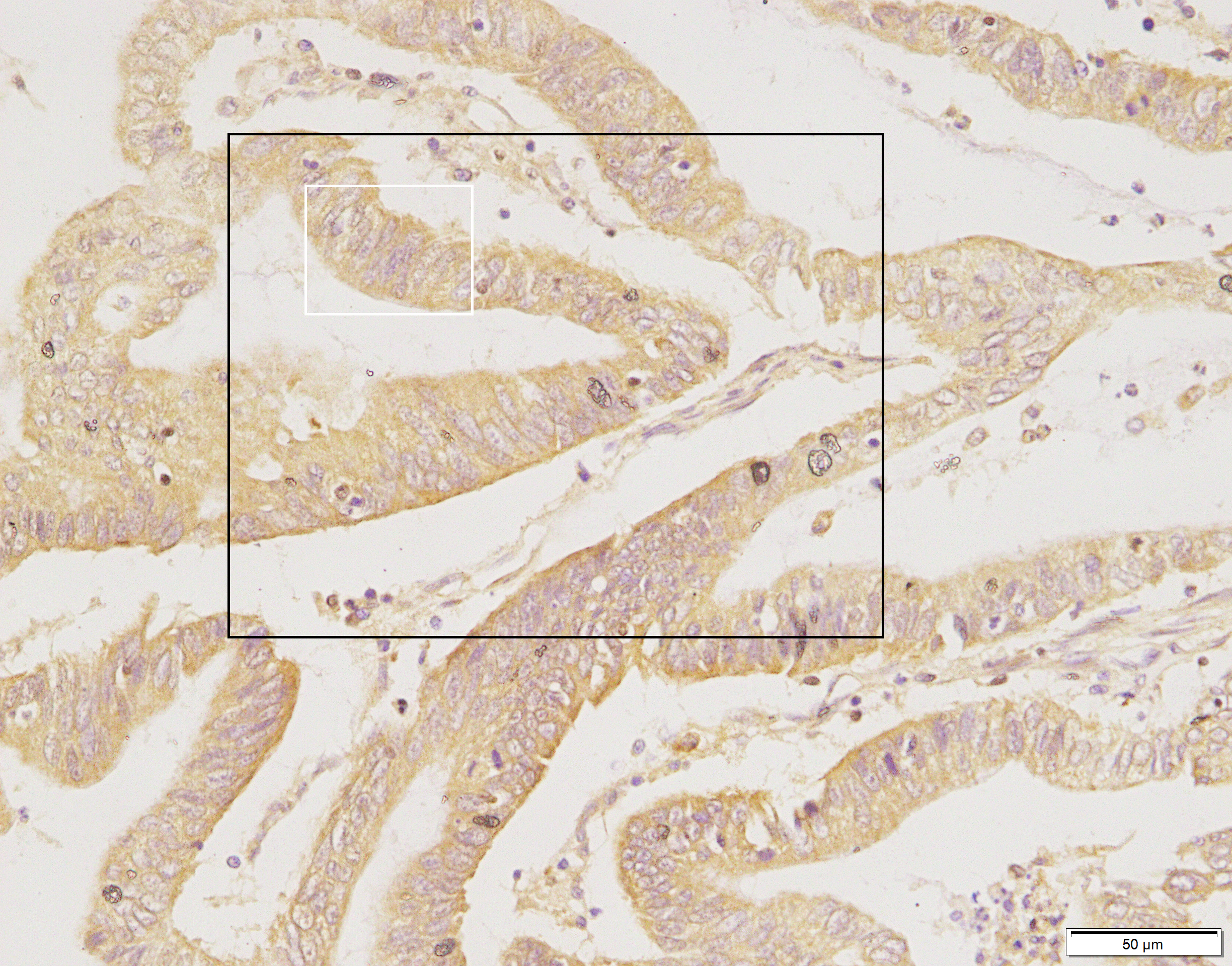

Supplement: Supplementary file 11 — Source data Fig. 8 [file 44318_2024_120_MOESM11_ESM.zip › Figure 8/8A/CRC.tif]

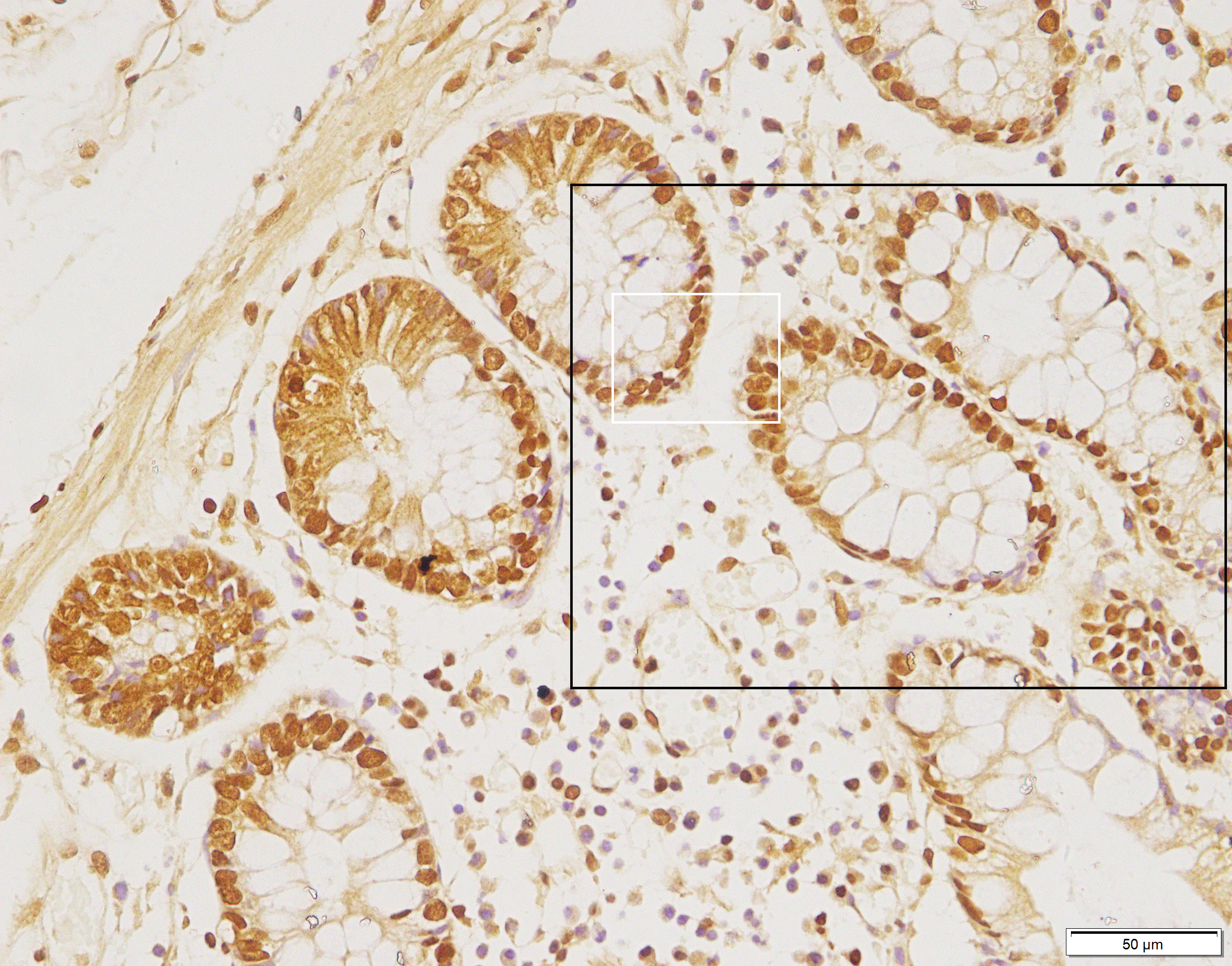

Supplement: Supplementary file 11 — Source data Fig. 8 [file 44318_2024_120_MOESM11_ESM.zip › Figure 8/8A/Normal.tif]

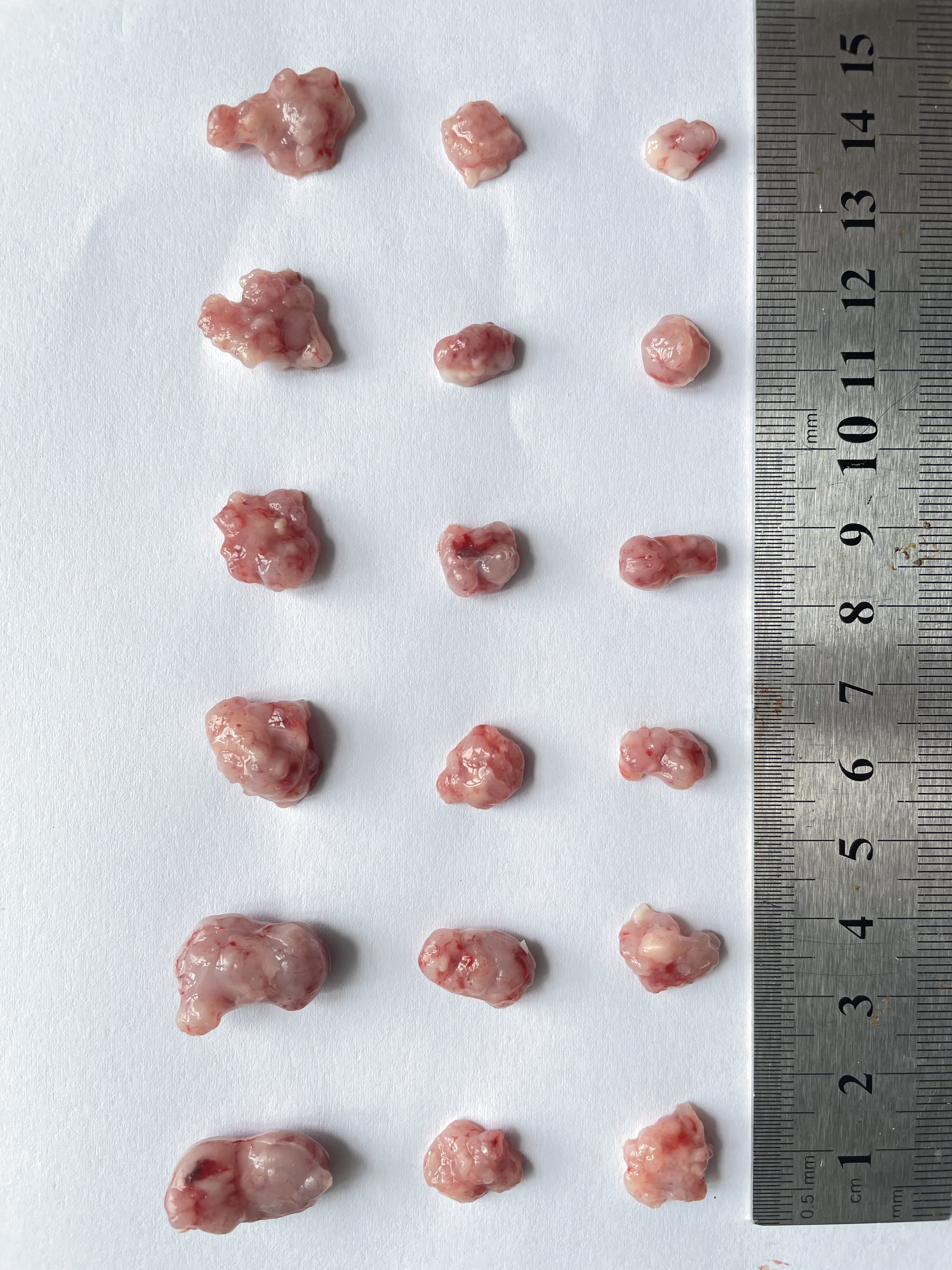

Supplement: Supplementary file 11 — Source data Fig. 8 [file 44318_2024_120_MOESM11_ESM.zip › Figure 8/8J/sgTRIM23.tif]

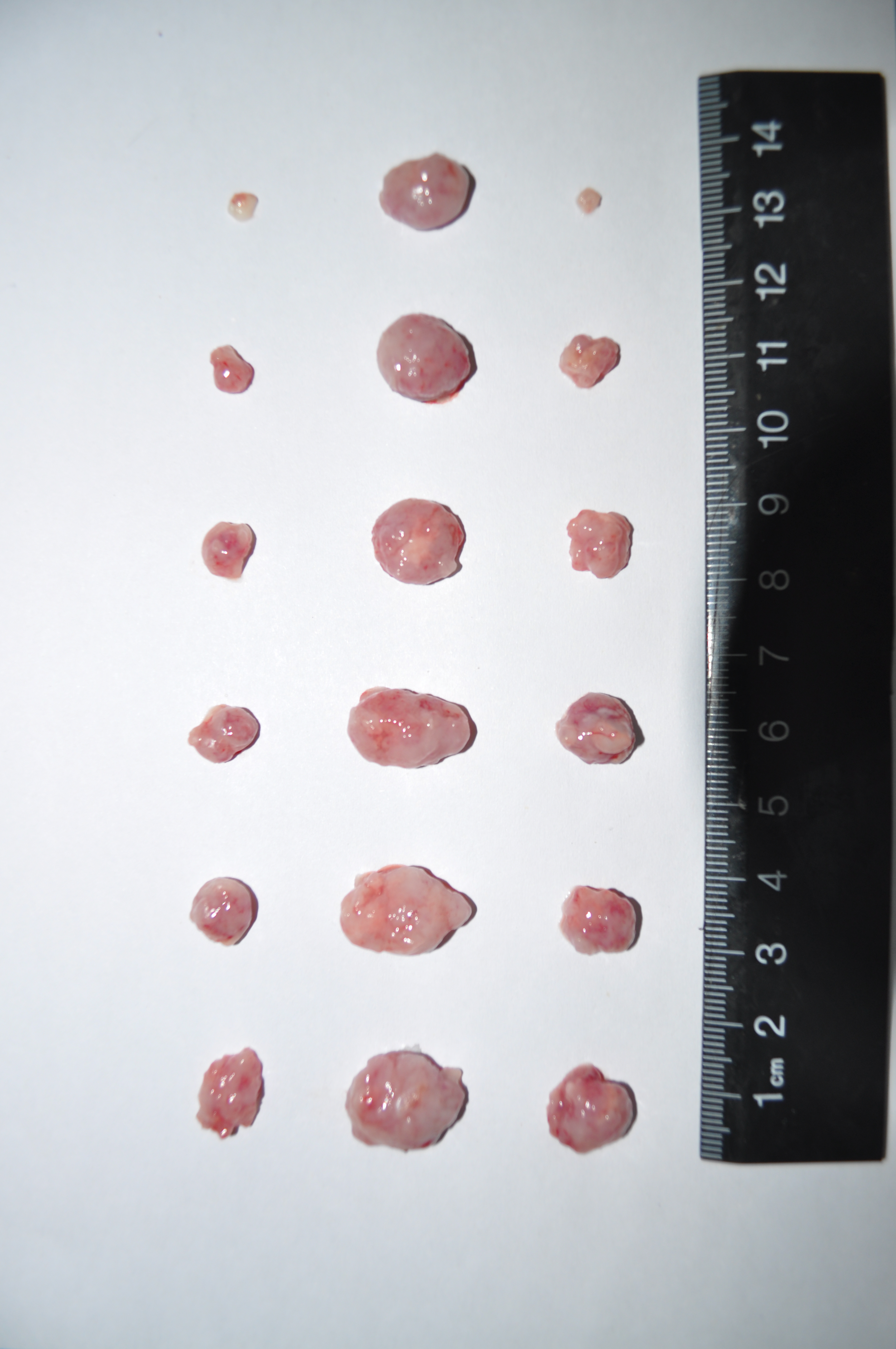

Supplement: Supplementary file 11 — Source data Fig. 8 [file 44318_2024_120_MOESM11_ESM.zip › Figure 8/8M/HAX1.tif]

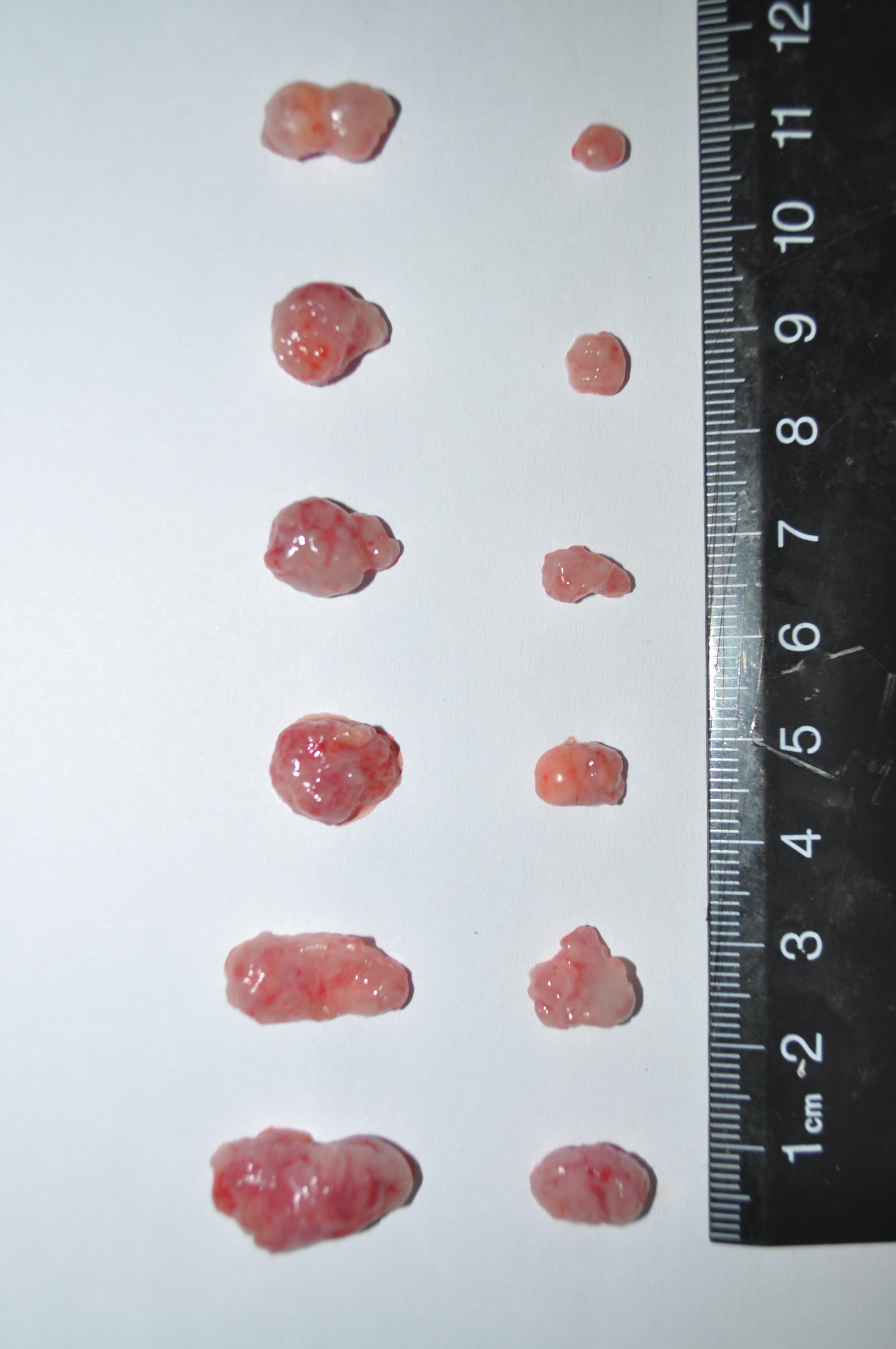

Supplement: Supplementary file 11 — Source data Fig. 8 [file 44318_2024_120_MOESM11_ESM.zip › Figure 8/8L/sgHAX1.tif]

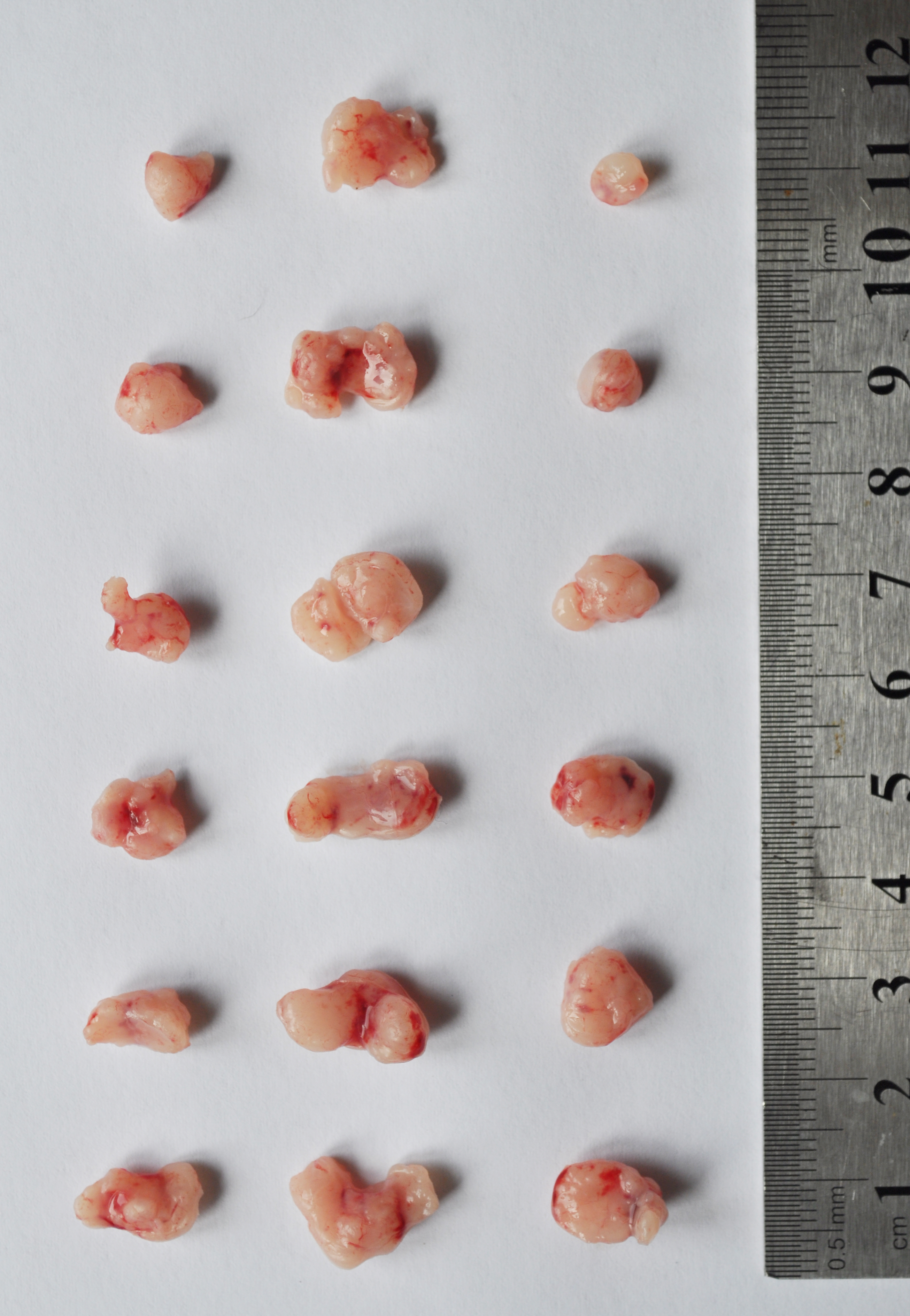

Supplement: Supplementary file 11 — Source data Fig. 8 [file 44318_2024_120_MOESM11_ESM.zip › Figure 8/8K/TRIM23.tif]

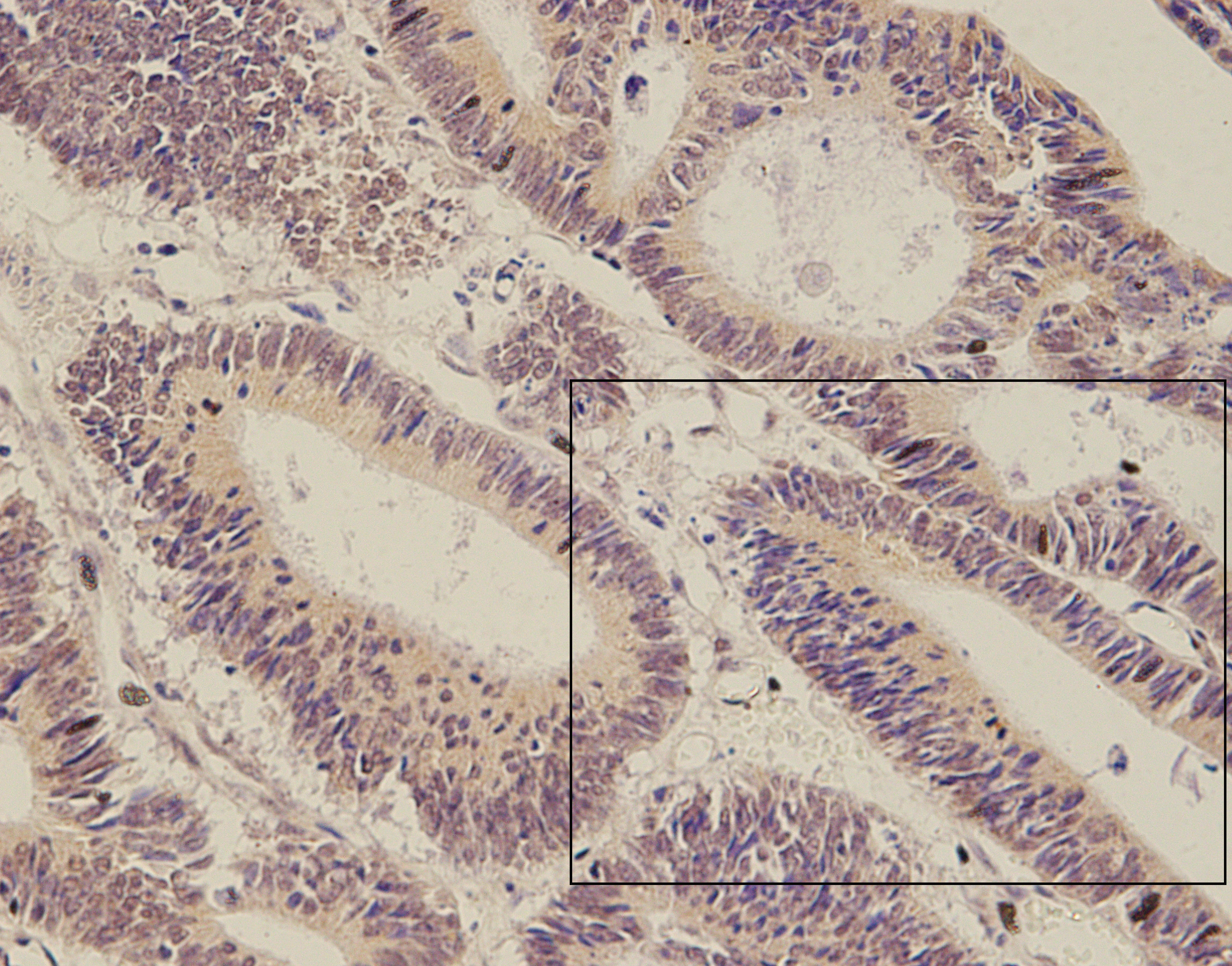

Supplement: Supplementary file 11 — Source data Fig. 8 [file 44318_2024_120_MOESM11_ESM.zip › Figure 8/8D/HAX1L-TRIM23C/TRIM23.tif]

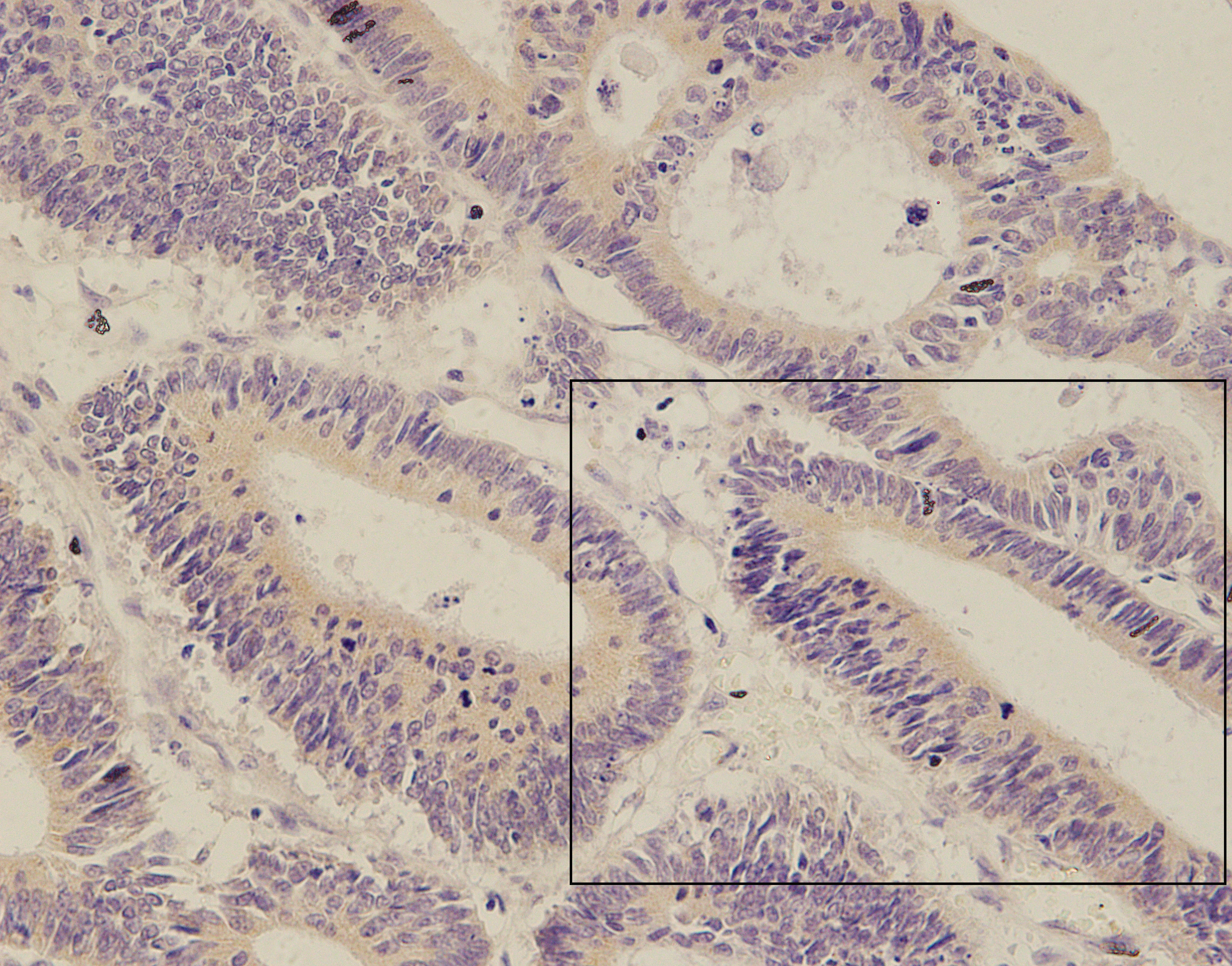

Supplement: Supplementary file 11 — Source data Fig. 8 [file 44318_2024_120_MOESM11_ESM.zip › Figure 8/8D/HAX1L-TRIM23C/HAX1.tif]

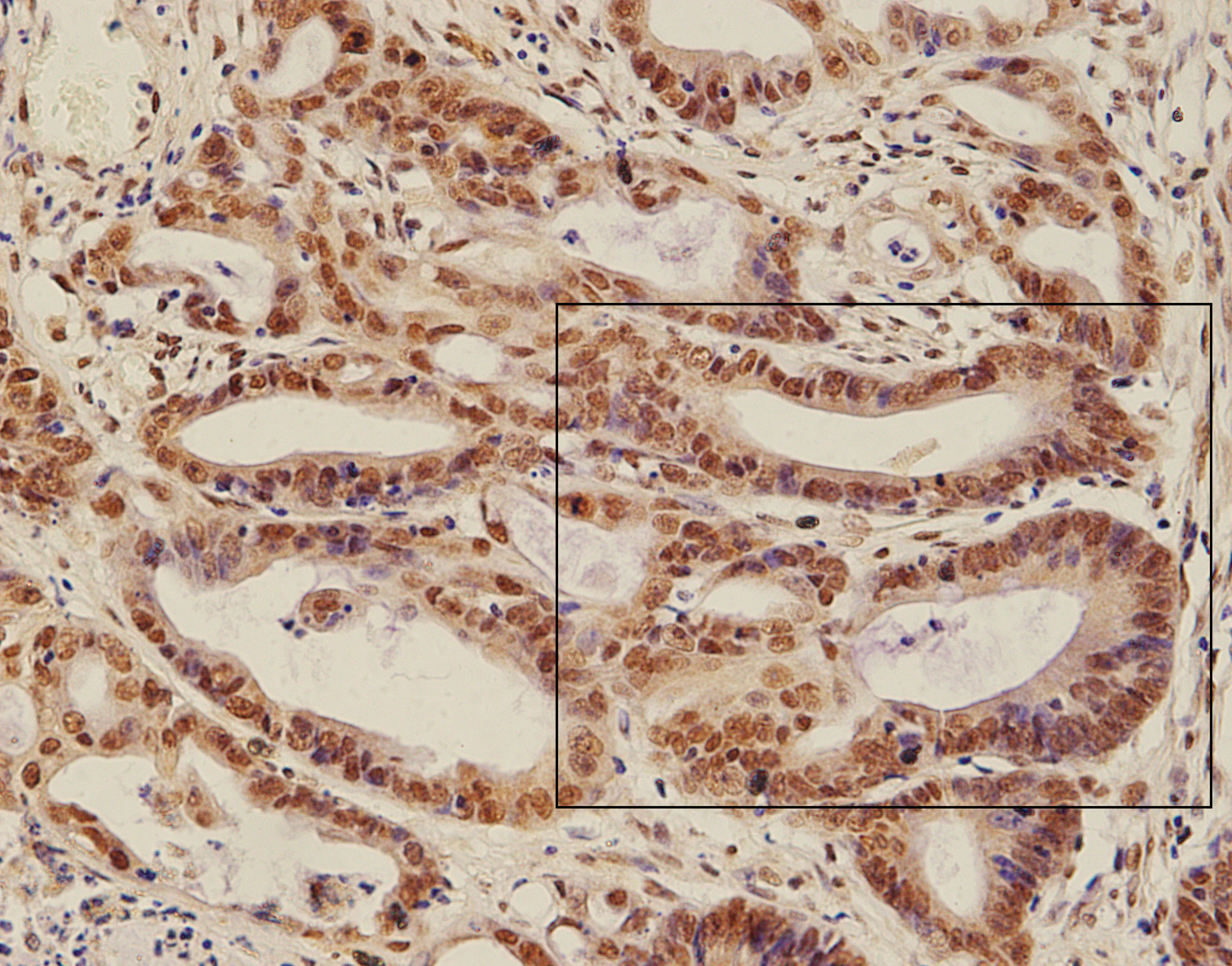

Supplement: Supplementary file 11 — Source data Fig. 8 [file 44318_2024_120_MOESM11_ESM.zip › Figure 8/8D/HAX1H-TRIM23N/TRIM23.tif]

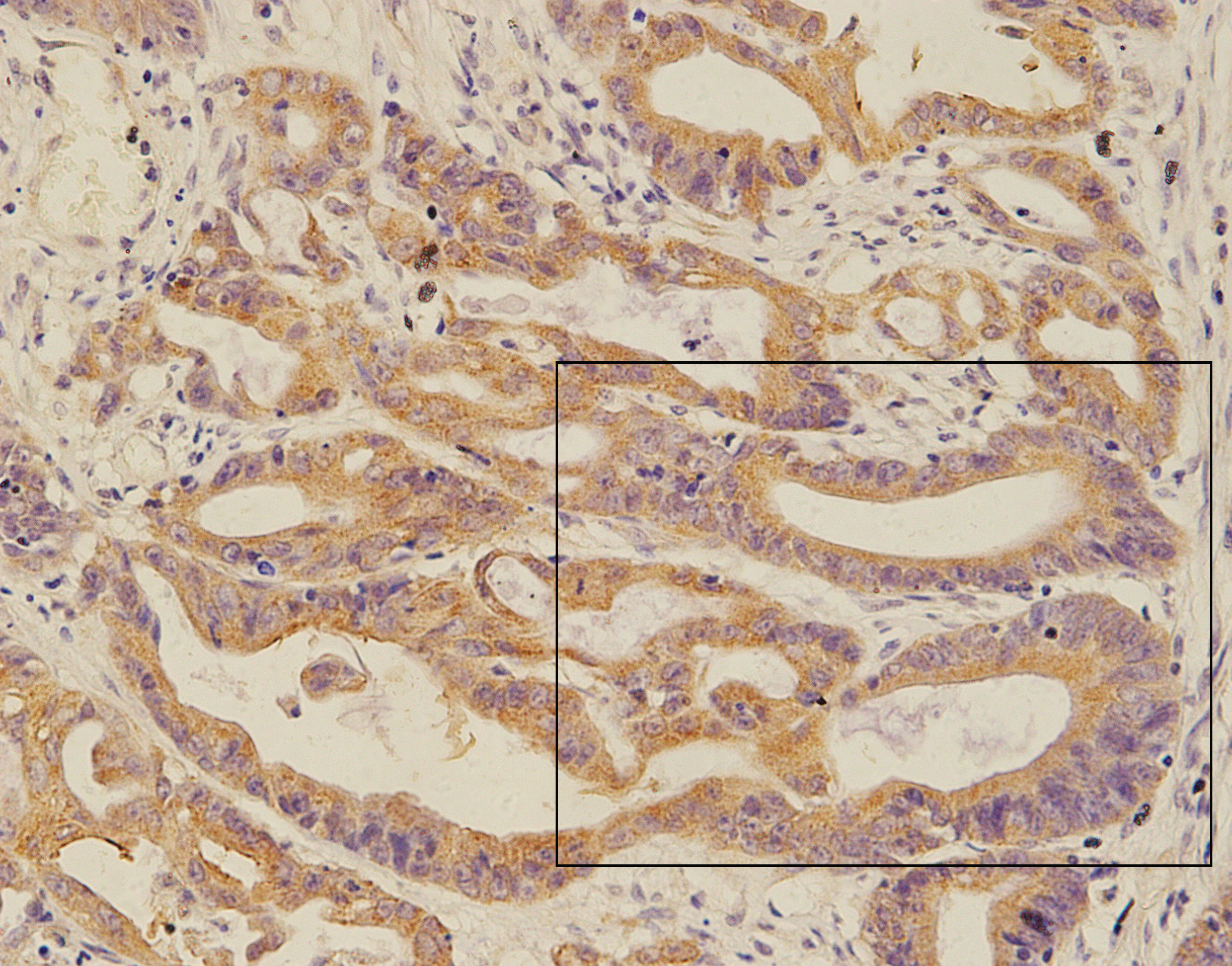

Supplement: Supplementary file 11 — Source data Fig. 8 [file 44318_2024_120_MOESM11_ESM.zip › Figure 8/8D/HAX1H-TRIM23N/HAX1.tif]

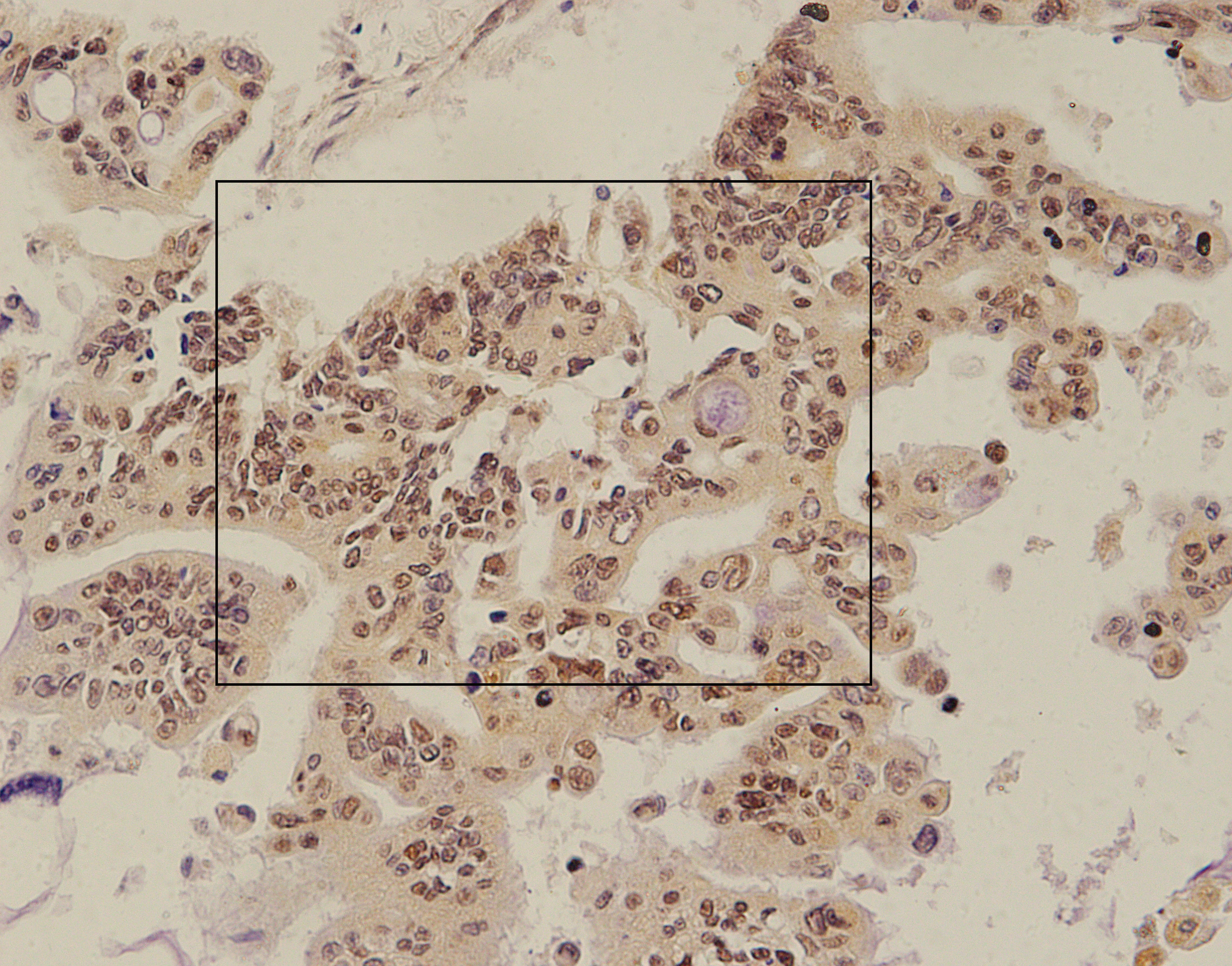

Supplement: Supplementary file 11 — Source data Fig. 8 [file 44318_2024_120_MOESM11_ESM.zip › Figure 8/8D/HAX1L-TRIM23N/TRIM23.tif]

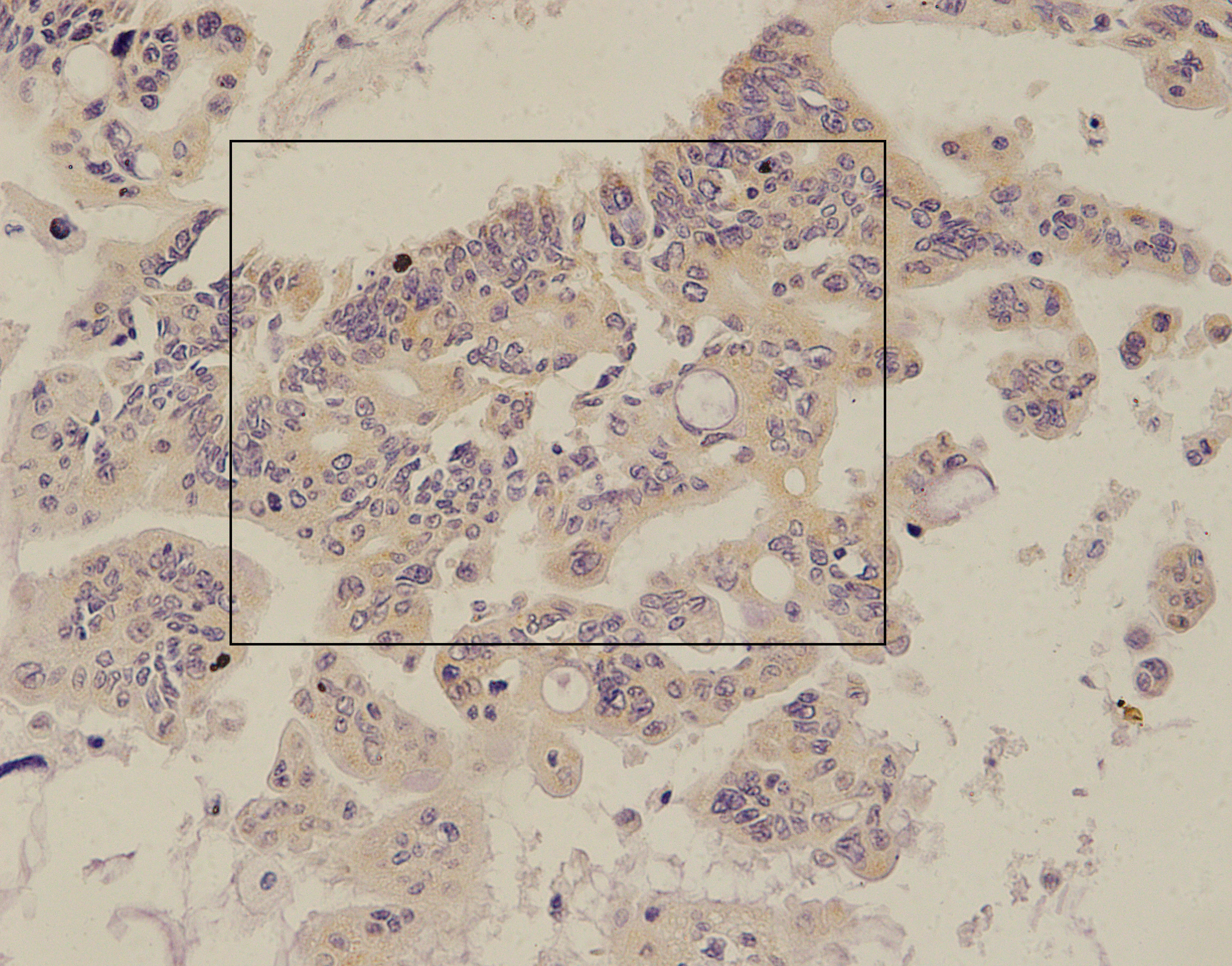

Supplement: Supplementary file 11 — Source data Fig. 8 [file 44318_2024_120_MOESM11_ESM.zip › Figure 8/8D/HAX1L-TRIM23N/HAX1.tif]

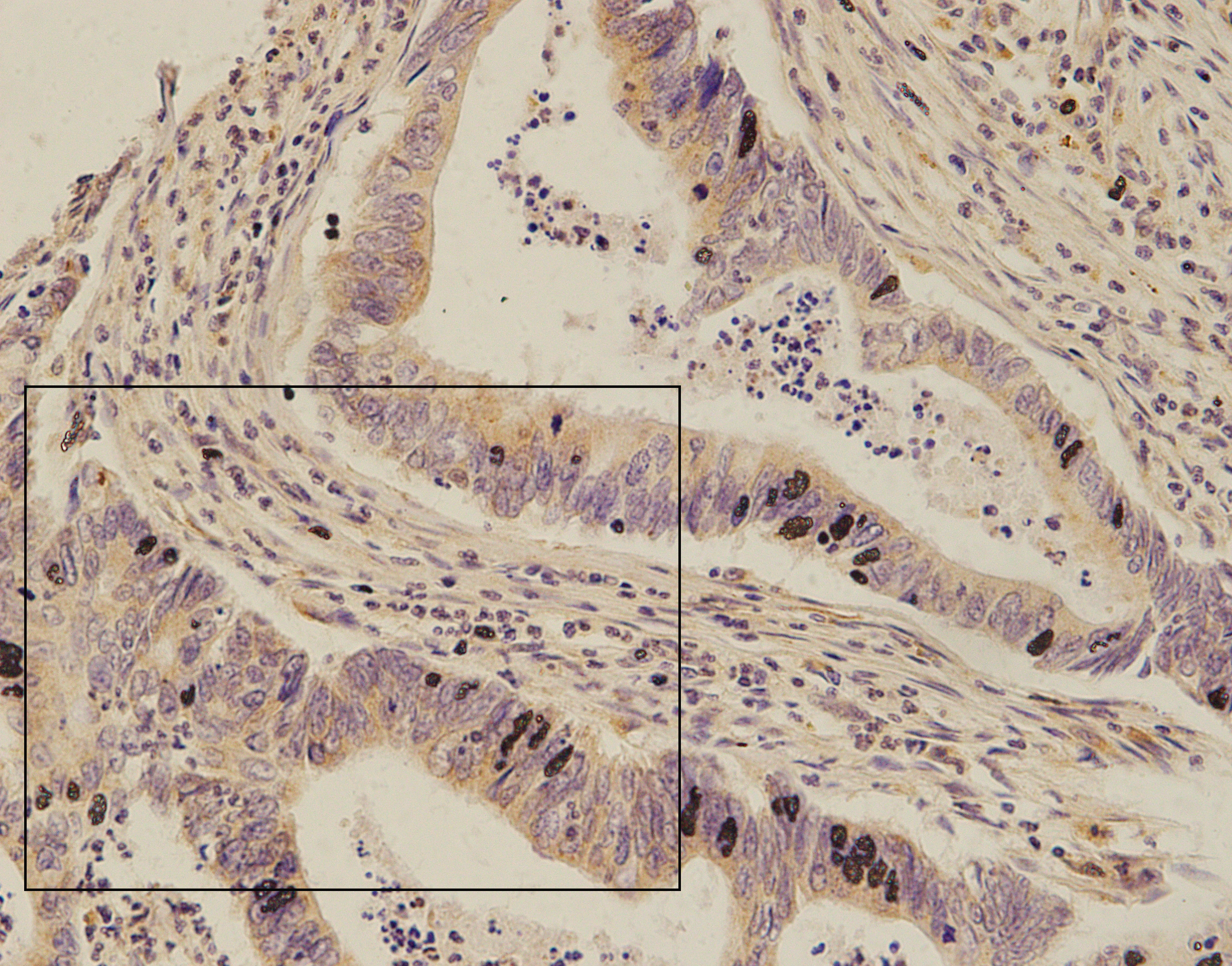

Supplement: Supplementary file 11 — Source data Fig. 8 [file 44318_2024_120_MOESM11_ESM.zip › Figure 8/8D/HAX1H-TRIM23C/TRIM23.tif]

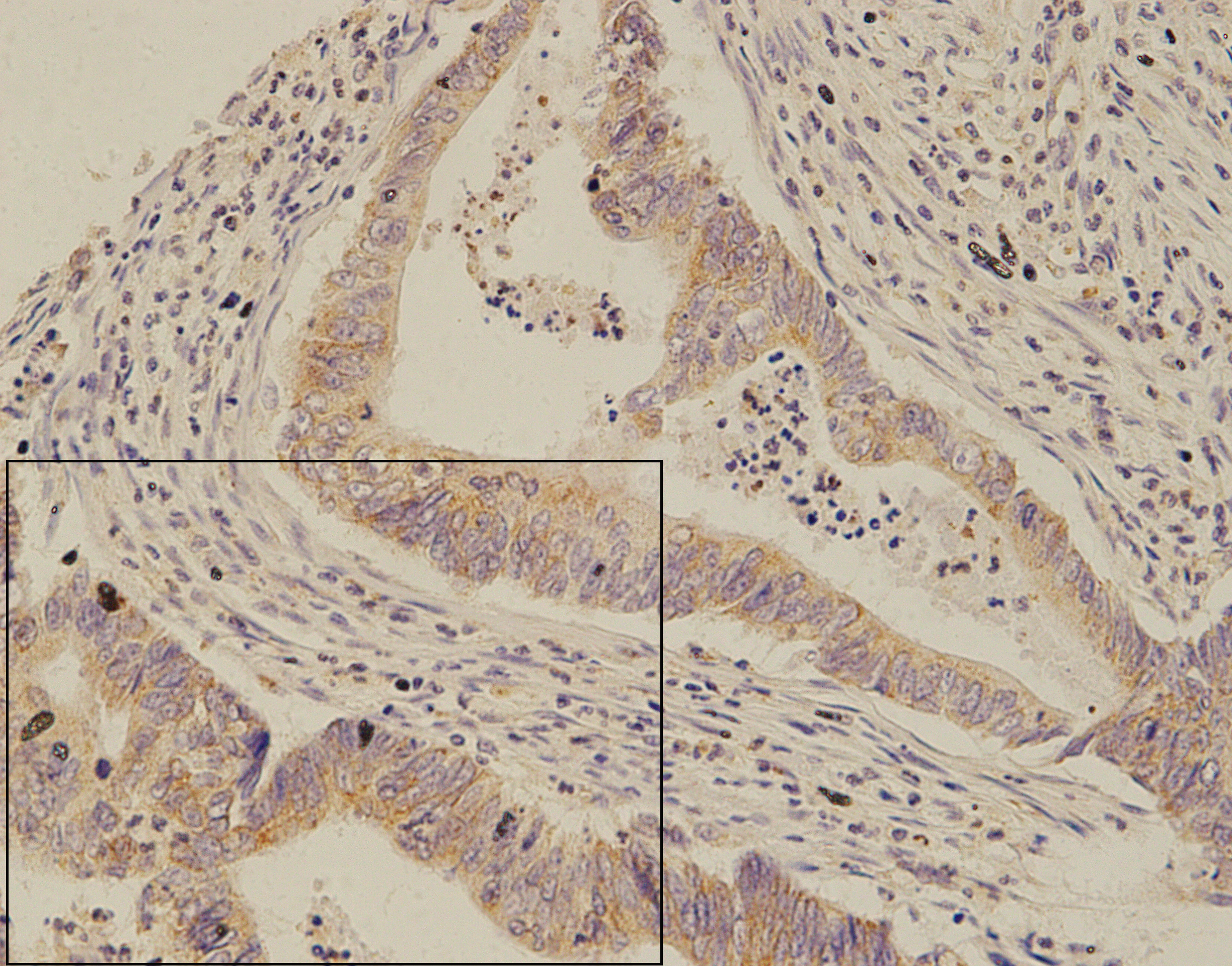

Supplement: Supplementary file 11 — Source data Fig. 8 [file 44318_2024_120_MOESM11_ESM.zip › Figure 8/8D/HAX1H-TRIM23C/HAX1.tif]
