# Supplementary material for: Energy stress promotes P-bodies formation via lysine-63-linked polyubiquitination of HAX1
Source: EMBO J. 2024 May 20;43(13):11. doi: 10.1038/s44318-024-00120-6 (PMC11217408; doi:10.1038/s44318-024-00120-6)
Supplement: Supplementary file 13 — Expanded View Figures [file 44318_2024_120_MOESM13_ESM.pdf]

Expanded View Figures

Figure EV1. ATP regulates P-bodies formation.

(**A**) The cellular ATP content of HEK293T cells treated with 2-DG or/and oligomycin was detected. Three biological replicates are plotted as the mean ± SD. ***$P < 0.001$ (Student's t test). (**B**, **C**) HEK293T cells treated with 2-DG or/and oligomycin (2 h) were exposed to sodium arsenite (2 h) and immunolabeled for TIA1 and G3BP2. Representative images are shown in (**B**). The number of stress granules represented by TIA1/G3BP2 within each cell is plotted in (**C**) ($n = 50$). Scale bar, 5 μm. Error bars indicate SEM. ns: no significance, ****$P < 0.0001$ (two-way ANOVA). (**D**, **E**) RKO cells were treated with oligomycin (1 h) and stained for LSM14A and DCP1A. Representative images are shown in (**D**). The number of P-bodies within each cell is plotted in (**E**) ($n = 50$). Scale bar, 5 μm. Error bars indicate SEM. ****$P < 0.0001$ (Student's t test). (**F**) The number of P-bodies of different sizes in Fig. 1A. ImageJ software was used to determine the particle size of the P-bodies (large, diameter >0.2 μm; small, diameter <0.2 μm). Error bars indicate SD. Red and black lines are used for the comparation of large and small P-bodies. ****$P < 0.0001$ (two-way ANOVA). (**G**) The cellular ATP content of HEK293T cells treated with or without NADH plus CoQ10 (2 h) and exposed to heat shock (42 °C) (1 h). Three biological replicates are plotted as the mean ± SD. ****$P < 0.0001$ (two-way ANOVA). (**H**, **I**) RKO cells were treated with or without NADH plus CoQ10 (2 h) and stained for LSM14A and DCP1A. Representative images are shown in (**H**). The number of P-bodies within each cell is plotted in (**I**) ($n = 50$). Scale bar, 5 μm. Error bars indicate SEM. ****$P < 0.0001$ (Student's t test). (**J**) ATP assay of HEK293T cells exposed to heat shock (42 °C) (1 h) or treated with oligomycin (2 h) in the presence of liposome or ATPosome. Three biological replicates are plotted as the mean ± SD. ****$P < 0.0001$ (two-way ANOVA). (**K**, **L**) The number of P-bodies of different sizes in Fig. 1E (**K**) and Fig. 1G (**L**). Error bars indicate SD. ns: no significance, ***$P < 0.001$, ****$P < 0.0001$ (two-way ANOVA). (**M**, **N**) RKO cells were treated with liposome or ATPsome (1 h) in the presence or absence of oligomycin (2 h) and stained for LSM14A and DCP1A. The cells were imaged (**M**), and the number of P-bodies within each cell was quantified (**N**) ($n = 50$). Scale bar, 5 μm. Error bars indicate SEM. *$P < 0.05$, ****$P < 0.0001$ (two-way ANOVA).

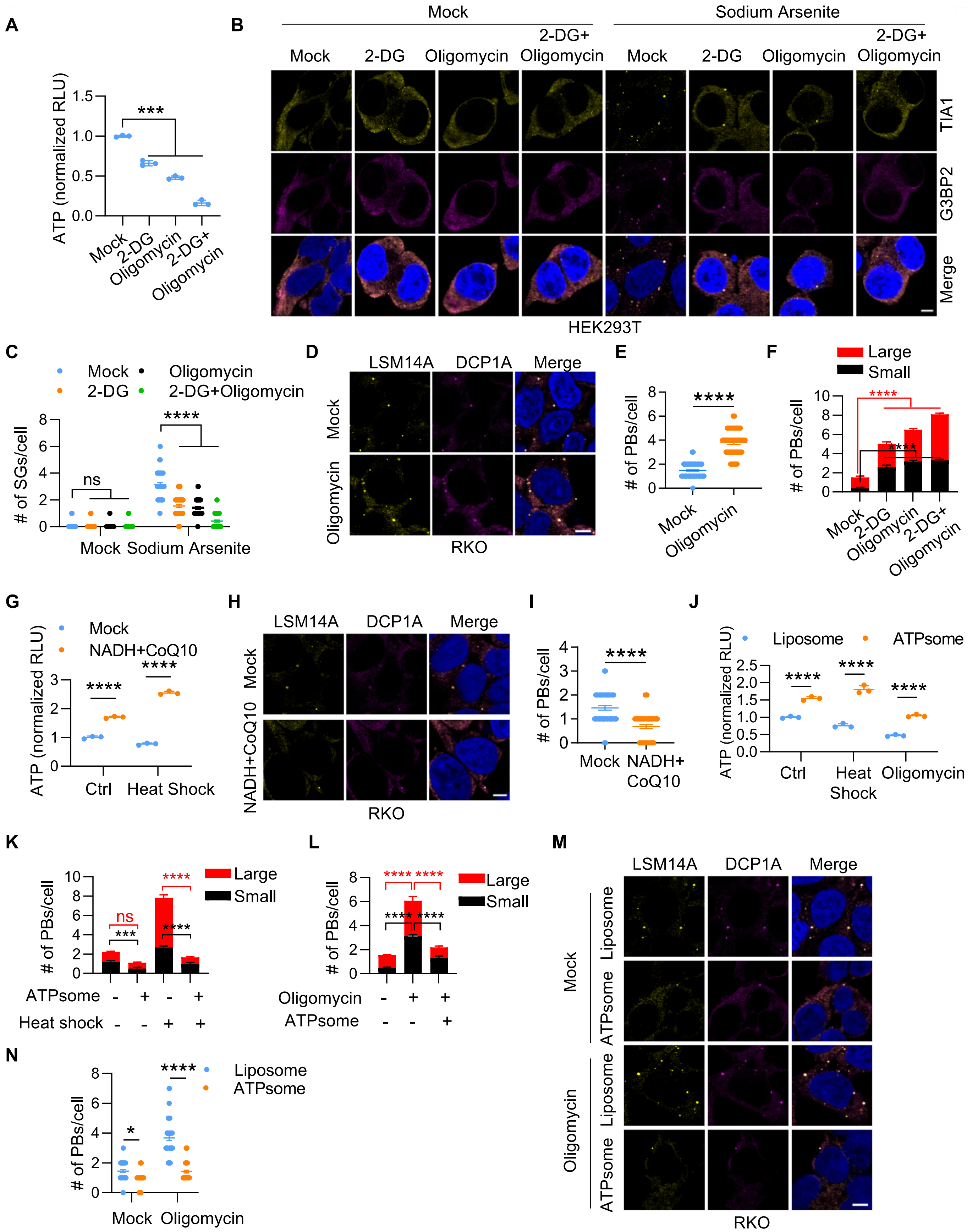
A
B
Mock
Sodium Arsenite
Mock
2-DG
Oligomycin
2-DG+ Oligomycin
TIA1
G3BP2
Merge
HEK293T
ATP (normalized RLU)
C
# of SGs/cell
Mock
2-DG
Oligomycin
2-DG+Oligomycin
ns
****
Sodium Arsenite
D
LSM14A
DCP1A
RKO
E
# of PBs/cell
F
Large
Small
G
NADH+CoQ10
Ctrl
Heat Shock
H
I
NADH+ CoQ10
J
Liposome
ATPsome
K
L
M
N
Heat shock

 © The Author(s)

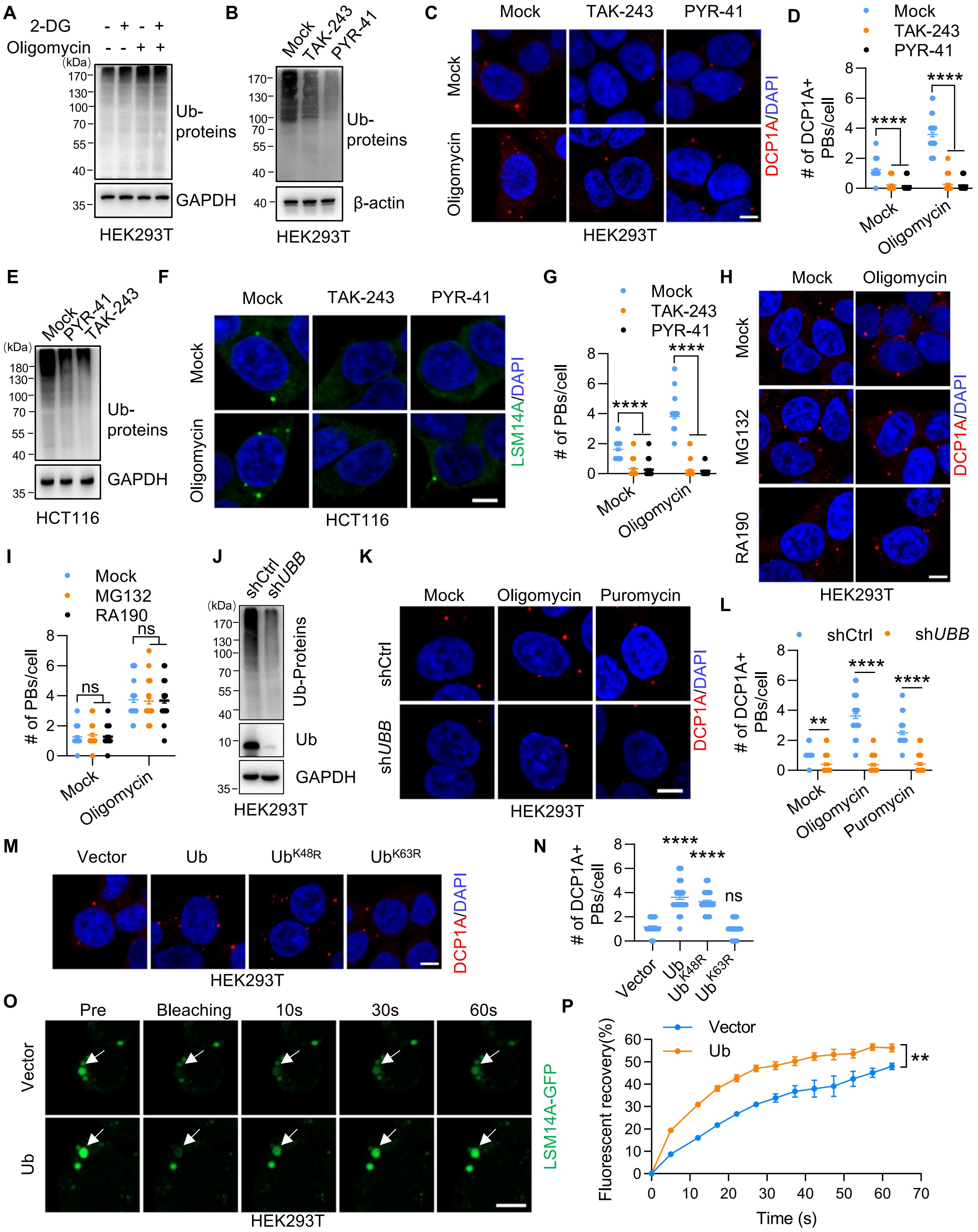
A
2-DG
Oligomycin
Ub-proteins
GAPDH
HEK293T
B
Mock
TAK-243
PYR-41
Ub-proteins
β-actin
HEK293T
C
Mock
TAK-243
PYR-41
Oligomycin
DCP1A/DAPI
HEK293T
D
# of DCP1A+ PBs/cell
E
HCT116
F
LSM14A/DAPI
G
# of PBs/cell
H
MG132
RA190
I
J
shCtrl
shUBB
Ub-Proteins
Ub
K
Puromycin
L
M
Vector
Ub
Ub K48R
Ub K63R
N
ns
O
Pre
Bleaching
10s
30s
60s
LSM14A-GFP
P
Fluorescent recovery(%)
Time (s)

Figure EV2. Ubiquitin regulates oligomycin-induced P-bodies dynamics.

(**A**) HEK293T cells were treated with 2-DG or/and oligomycin for 2 h. The ubiquitination level of the proteins was detected by immunoblotting. (**B**) Immunoblot of HEK293T cells treated with TAK-243 or PYR-41 (2 h). (**C**, **D**) HEK293T cells were treated with TAK-243 or PYR-41 (1 h) in the presence or absence of oligomycin (1 h) and immunolabeled for DCP1A. Representative images are shown in (**C**). Scale bar, 5 μm. The number of P-bodies within each cell is plotted in (**D**) ($n = 50$). Error bars indicate SEM. ****$P < 0.0001$ (two-way ANOVA). (**E**) Immunoblot of HCT116 cells treated with TAK-243 or PYR-41 (2 h). (**F**, **G**) HCT116 cells were treated with TAK-243 or PYR-41 in the presence or absence of oligomycin (1 h) and immunolabeled for LSM14A. Representative images are shown in (**F**). Scale bar, 5 μm. The number of P-bodies within each cell is plotted in (**G**) ($n = 50$). Error bars indicate SEM. ****$P < 0.0001$ (two-way ANOVA). (**H**, **I**) HEK293T cells were treated with MG132 or RA190 (1 h) in the presence or absence of oligomycin (1 h) and immunolabeled for DCP1A. Representative images are shown in (**H**). Scale bar, 5 μm. The number of P-bodies within each cell is plotted in (**I**). Error bars indicate SEM. ns: no significance (two-way ANOVA). (**J**) Immunoblot of control and *UBB*-knockdown HEK293T cells. (**K**, **L**) Control and *UBB*-knockdown HEK293T cells were treated with oligomycin (1 h) or puromycin (1 h), and stained for DCP1A and DAPI. Representative images are shown in (**K**). The number of P-bodies within each cell is plotted in (**L**) ($n = 50$). Error bars indicate SEM. **$P < 0.01$, ****$P < 0.0001$ (two-way ANOVA). (**M**, **N**) HEK293T cells transfected with vector, Ub, Ub^{K48R} or Ub^{K63R} were stained for DCP1A and DAPI. Representative images are shown in (**M**). The number of P-bodies within each cell is plotted in (**N**) ($n = 50$). Scale bar, 5 μm. Error bars indicate SEM. Ns: no significance, ****$P < 0.0001$ (Student's t test). (**O**, **P**) FRAP analysis of GFP-LSM14A in HEK293T cells transfected with vector or Ub. White arrows indicate the target droplets for FRAP. Scale bar, 5 μm. $n = 3$, Error bars indicate SEM. **$P < 0.01$ (two-way ANOVA).

 © The Author(s)

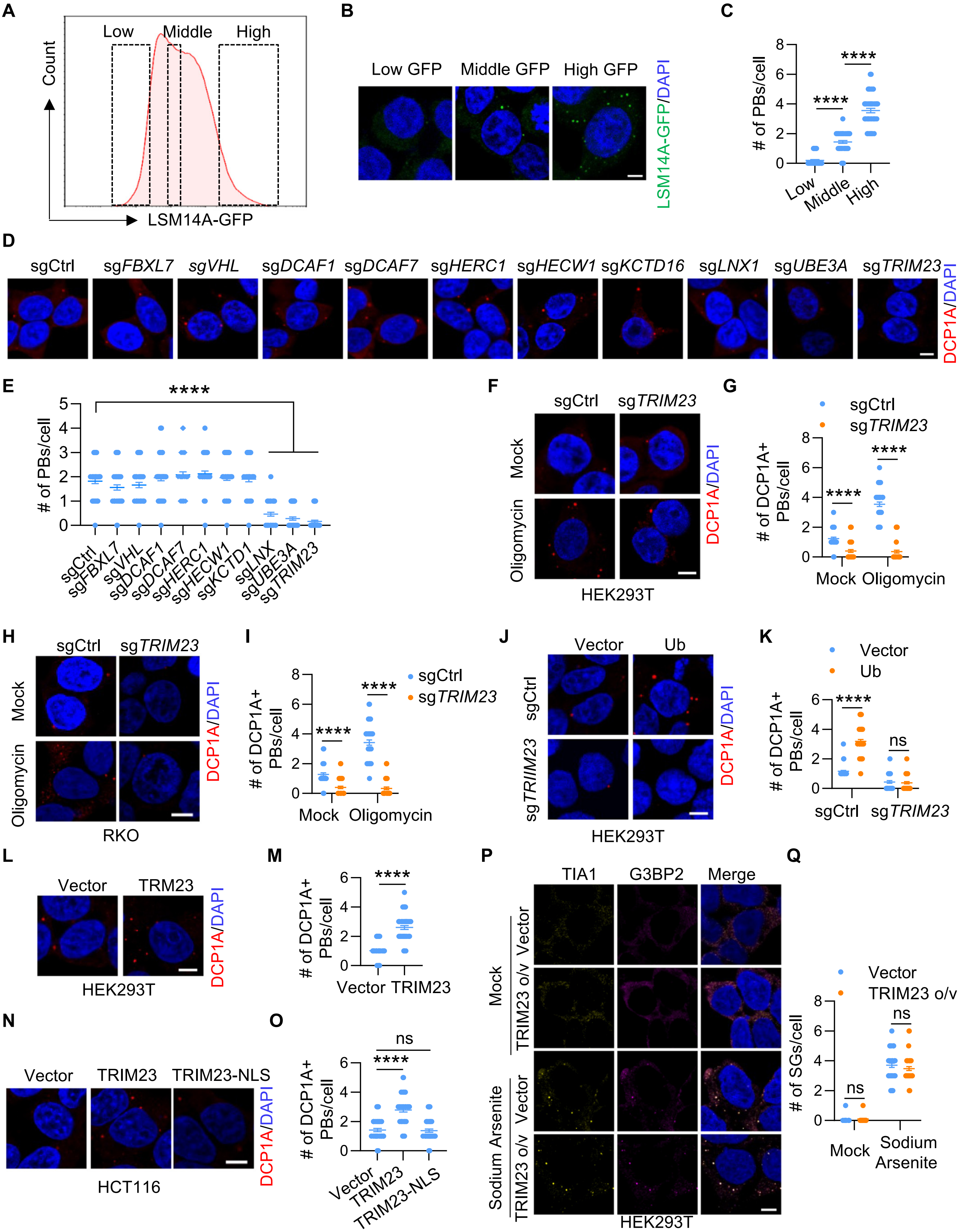
A
Low
Middle
High
Count
LSM14A-GFP
B
Low GFP
Middle GFP
High GFP
LSM14A-GFP/DAPI
C
# of PBs/cell
****
****
Low
Middle
High
D
sgCtrl
sgFBXL7
sgVHL
sgDCAF1
sgDCAF7
sgHERC1
sgHECW1
sgKCTD16
sgLNX1
sgUBE3A
sgTRIM23
DCP1A/DAPI
E
****
# of PBs/cell
sgCtrl
sgFBXL7
sgVHL
sgDCAF1
sgDCAF7
sgHERC1
sgHECW1
sgKCTD1
sgLNX
sgUBE3A
sgTRIM23
F
sgCtrl
sgTRIM23
Mock
Oligomycin
DCP1A/DAPI
HEK293T
G
sgCtrl
sgTRIM23
# of DCP1A+ PBs/cell
****
****
Mock
Oligomycin
H
sgCtrl
sgTRIM23
Mock
Oligomycin
DCP1A/DAPI
RKO
I
sgCtrl
sgTRIM23
# of DCP1A+ PBs/cell
****
****
Mock
Oligomycin
J
Vector
Ub
sgCtrl
sgTRIIM23
DCP1A/DAPI
HEK293T
K
Vector
Ub
# of DCP1A+ PBs/cell
****
ns
sgCtrl
sgTRIM23
L
Vector
TRM23
DCP1A/DAPI
HEK293T
M
# of DCP1A+ PBs/cell
****
Vector
TRIM23
N
Vector
TRIM23
TRIM23-NLS
DCP1A/DAPI
HCT116
O
ns
****
# of DCP1A+ PBs/cell
Vector
TRIM23
TRIM23-NLS
P
TIA1
G3BP2
Merge
Mock
Vector
TRIM23 o/v
Sodium Arsenite
Vector
TRIM23 o/v
HEK293T
Q
Vector
TRIM23 o/v
# of SGs/cell
ns
ns
Mock
Sodium Arsenite

Figure EV3. TRIM23 promotes P-bodies formation.

(**A–C**) HEK293T cells expressing GFP-LSM14A with low, middle, and high fluorescence intensities were isolated by fluorescence-activated cell sorting (**A**). Cells were imaged after sorting (**B**), and the number of P-bodies within each cell from one of two biological replicates is plotted in (**C**) ($n = 50$). Scale bar, 5 μm. Error bars indicate SEM. ****$P < 0.0001$ (Student's t test). (**D**, **E**) HEK293T cells were transfected with LentiCRISPR constructs targeting the indicated genes for 48 h and selected with puromycin for 72 h. Cells were stained for DCP1A and DAPI and imaged (**D**). Scale bar, 5 μm. The number of P-bodies within each cell is plotted in (**E**) ($n = 50$). Error bars indicate SEM. ****$P < 0.0001$ (Student's t test). (**F–K**) *TRIM23* KO HEK293T cells (**F**, **G**, **J**, **K**) or *TRIM23* KO RKO cells (**H**, **I**) were treated with or without oligomycin (1 h) (**F–I**) or transfected with vector or Ub (**J**, **K**) and stained for DCP1A and DAPI. Representative images are shown in (**F**, **H**, **J**). The number of P-bodies within each cell is plotted in (**G**, **I**, **K**) ($n = 50$). Scale bar, 5 μm. Error bars indicate SEM. ns: no significance, ****$P < 0.0001$ (two-way ANOVA). (**L–O**) HEK293T cells transfected with TRIM23 or vector and HCT116 cells transduced with lentivirus expressing control, TRIM23 or TRIM23-NLS were stained for DCP1A and DAPI. Representative images are shown in (**L**, **N**). The number of P-bodies within each cell is plotted in (**M**, **O**) ($n = 50$). Scale bar, 5 μm. Error bars indicate SEM. ns: no significance, ****$P < 0.0001$ (Student's t test). (**P**, **Q**) HEK293T cells transfected with TRIM23 or vector were exposed to sodium arsenite (2 h) and immunolabeled for TIA1 and G3BP2. Representative images are shown in (**P**). The number of stress granules within each cell is plotted in (**Q**) ($n = 50$). Scale bar, 5 μm. Error bars indicate SEM. ns: no significance (two-way ANOVA).

 © The Author(s)

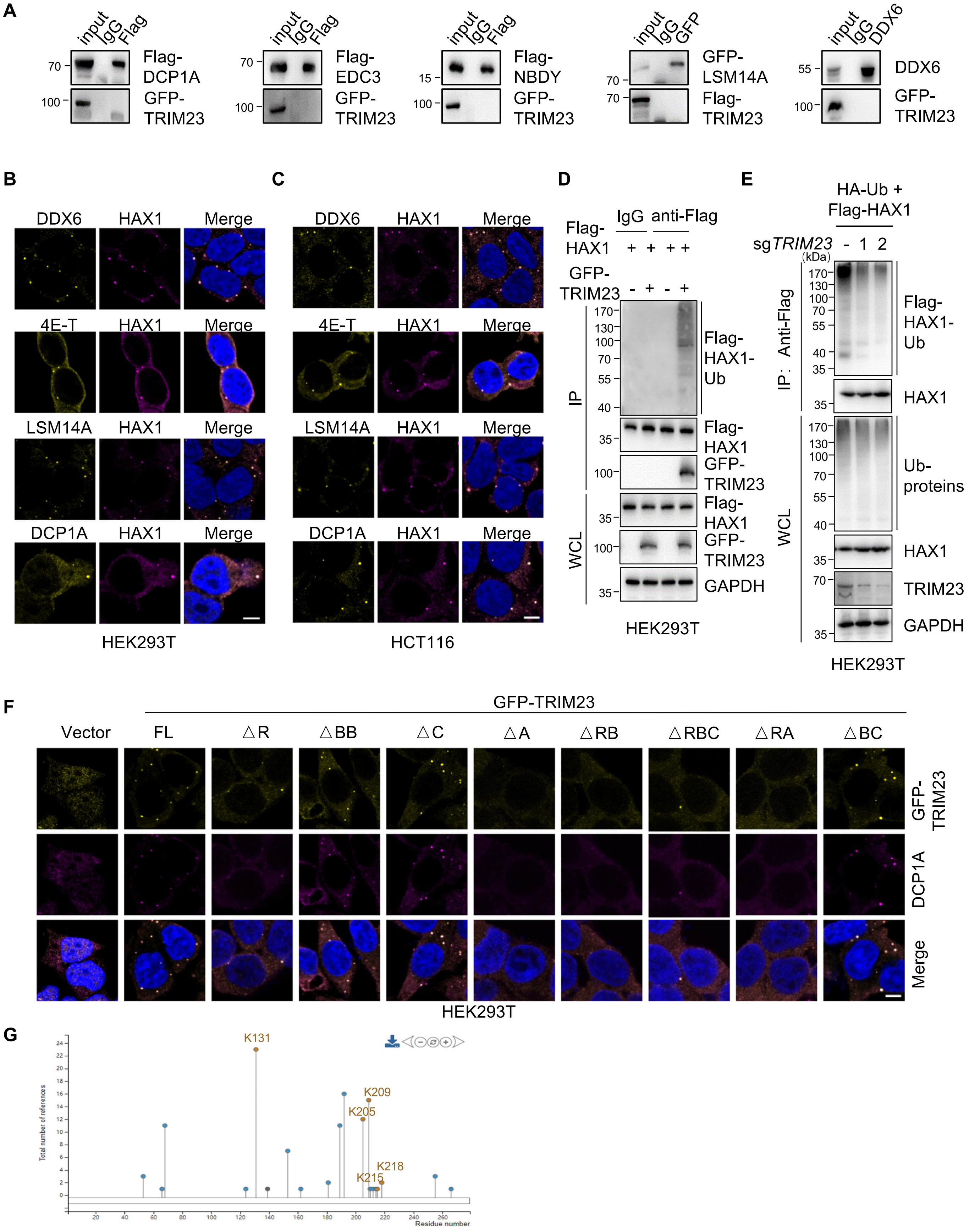
A
input
IgG
Flag
70
100
Flag-DCP1A
GFP-TRIM23
Flag-EDC3
GFP-TRIM23
15
Flag-NBDY
GFP-TRIM23
GFP
GFP-LSM14A
Flag-TRIM23
DDX6
55
DDX6
GFP-TRIM23
B
DDX6 HAX1 Merge
4E-T HAX1 Merge
LSM14A HAX1 Merge
DCP1A HAX1 Merge
HEK293T
C
DDX6 HAX1 Merge
4E-T HAX1 Merge
LSM14A HAX1 Merge
DCP1A HAX1 Merge
HCT116
D
IgG anti-Flag
Flag-HAX1 + + + +
GFP-TRIM23 - + - +
IP
WCL
170
130
100
70
55
40
35
Flag-HAX1-Ub
Flag-HAX1
GFP-TRIM23
GAPDH
HEK293T
E
HA-Ub + Flag-HAX1
sgTRIM23 - 1 2
(kDa)
IP: Anti-Flag
WCL
Flag-HAX1-Ub
HAX1
Ub-proteins
TRIM23
GAPDH
HEK293T
F
GFP-TRIM23
Vector FL △R △BB △C △A △RB △RBC △RA △BC
GFP-TRIM23
DCP1A
Merge
HEK293T
G
K131
K209
K205
K218
K215
Total number of references
Residue number

Figure EV4. TRIM23 interacts with HAX1 to catalyze ubiquitination.

(**A**) HEK293T cells were transfected with GFP-TRIM23 along with flag-DCP1A, flag-EDC3 or flag-NBDY, or GFP-LSM14A with flag-TRIM23, or GFP-TRIM23 alone. Cell lysates were immunoprecipitated using control IgG or the indicated antibodies. IP samples and whole-cell lysates (input) were analyzed via western blotting. (**B**, **C**) Immunofluorescence staining of HEK293T cells (**B**) or HCT116 cells (**C**) for endogenous HAX1 and DDX6, 4E-T, LSM14A, or DCP1A. Scale bar, 5 µm. (**D**) HEK293T cells were transfected with Flag-HAX and GFP-TRIM23. Cell lysates were immunoprecipitated using IgG or anti-Flag antibodies. IP samples and whole-cell lysates (WCLs) were analyzed via western blotting. (**E**) Control and *TRIM23*-knockout HEK293T cells were transfected with HA-Ub and Flag-HAX1. Cell lysates were immunoprecipitated using anti-Flag antibodies. IP samples and WCLs were analyzed using western blotting. (**F**) HEK293T cells were transfected with vector-GFP, TRIM23-GFP-FL or its deletion mutants and stained for DCP1A and DAPI. Scale bar, 5 µm. (**G**) Ubiquitination sites (Lys131/205/209/215/218) of HAX1 reported on PhosphoSitePlus.

 © The Author(s)

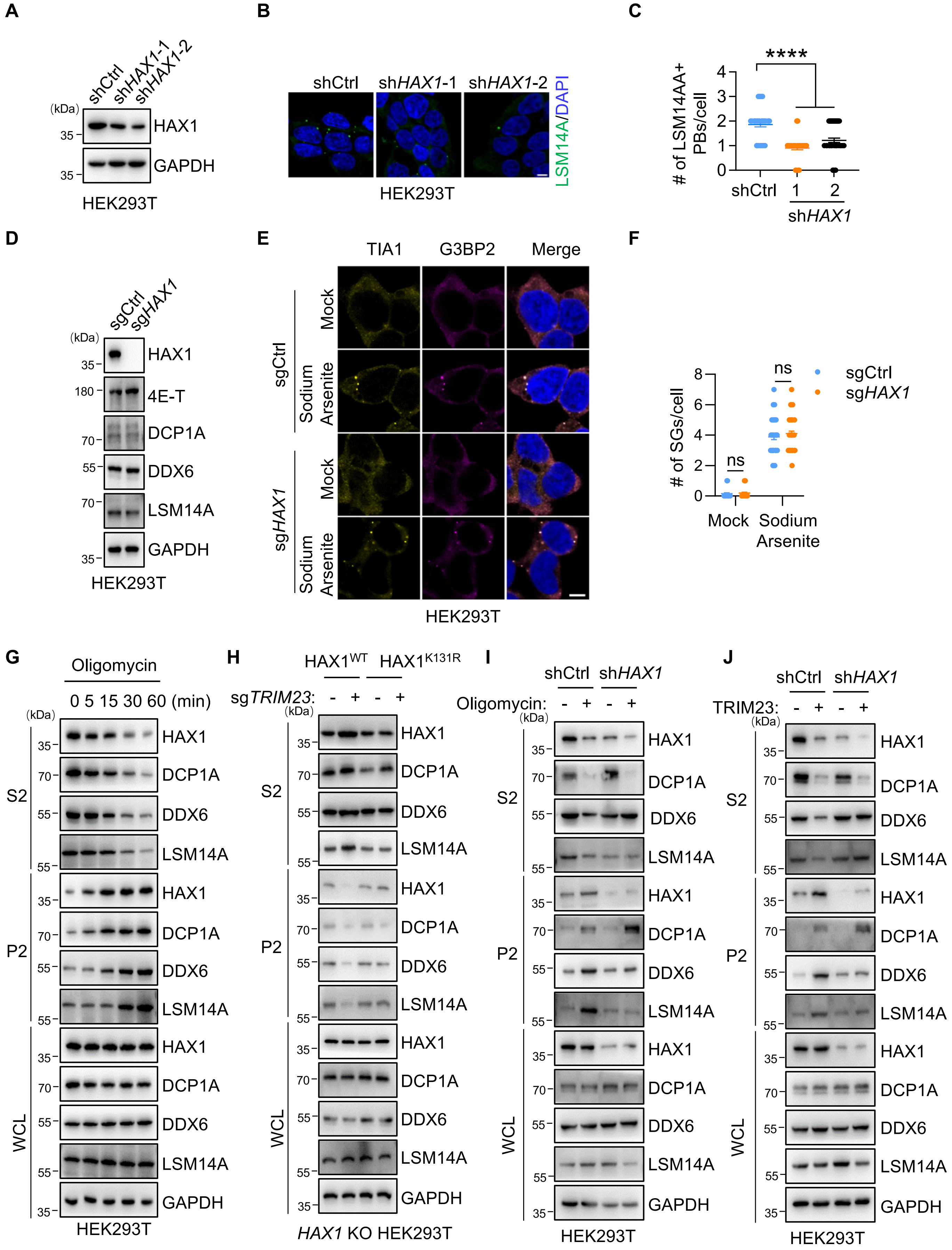
A
shCtrl
shHAX1-1
shHAX1-2
(kDa)
35
HAX1
35
GAPDH
HEK293T
B
shCtrl
shHAX1-1
shHAX1-2
LSM14A/DAPI
HEK293T
C
# of LSM14AA+ PBs/cell
****
0
1
2
3
4
shCtrl
1
2
shHAX1
D
sgCtrl
sgHAX1
(kDa)
35
HAX1
180
4E-T
70
DCP1A
55
DDX6
70
LSM14A
35
GAPDH
HEK293T
E
TIA1
G3BP2
Merge
sgCtrl
Mock
Sodium Arsenite
sgHAX1
Mock
Sodium Arsenite
HEK293T
F
# of SGs/cell
0
2
4
6
8
ns
ns
sgCtrl
sgHAX1
Mock
Sodium Arsenite
G
Oligomycin
0 5 15 30 60 (min)
(kDa)
S2
P2
WCL
HAX1
DCP1A
DDX6
LSM14A
GAPDH
35
70
55
HEK293T
H
HAX1WT
HAX1K131R
sgTRIM23:
- + - +
HAX1 KO HEK293T
I
shCtrl
shHAX1
Oligomycin:
- + - +
HEK293T
J
shCtrl
shHAX1
TRIM23:
- + - +
HEK293T

Figure EV5. The condensation of P-bodies induced by energy stress depends on ubiquitinated HAX1.

(**A**) Immunoblot of HEK293T cells transfected with shCtrl or two independent HAX1 shRNAs. (**B**, **C**) Control and HAX1 knockdown (sh*HAX1*) HEK293T cells were stained for LSM14A and DAPI. Representative images are shown in (**B**). The number of P-bodies within each cell is plotted in (**C**) ($n = 50$). Scale bar, 5 μm. Error bars indicate SEM. *****P* < 0.0001 (Student's t test). (**D**) The expression of HAX1, 4E-T, DCP1A, DDX6, and LSM14A in *HAX1* KO HEK293T cells was detected by immunoblotting. (**E**, **F**) Control and *HAX1* KO HEK293T cells were exposed to sodium arsenite (2 h) and immunolabeled for TIA1 and G3BP2 (**E**). Scale bar, 5 μm. The number of stress granules within each cell is plotted in (**F**) ($n = 50$). Statistical Error bars indicate SEM. Ns: no significance (two-way ANOVA). (**G**) S2 and P2 fractions from HEK293T cells treated with oligomycin for the indicated times were analyzed by immunoblotting. (**H**) S2 and P2 fractions from *HAX1* KO HEK293T cells transfected with $HAX1^{WT}$ or $HAX1^{K131R}$ along with sgCrtl or sg*TRIM23* were analyzed by immunoblotting. (**I**, **J**) S2 and P2 fractions from control and HAX1-knockdown (sh*HAX1*) HEK293T cells with or without oligomycin treatment (1 h) (**I**) or TRIM23 overexpression (**J**) were analyzed by immunoblotting.

© The Author(s)
